# Photoinduced Copper-Catalyzed Enantioselective Allylic C(*sp* ^3^)–H Oxygenation of Acyclic Terminal Olefins Enabled by SphenBOX

**DOI:** 10.1021/jacs.5c06136

**Published:** 2025-06-09

**Authors:** Xu-Kuan Qi, Qiang Dai, Yangyi Gu, Kwan Shing Lau, Herman H. Y. Sung, Ian D. Williams, Zhenyang Lin, Chaoshen Zhang, Jianwei Sun

**Affiliations:** † Department of Chemistry and the Hong Kong Branch of Chinese National Engineering Research Centre for Tissue Restoration & Reconstruction, 58207 The Hong Kong University of Science and Technology, Clear Water Bay, Kowloon 999077, Hong Kong SAR, China; ‡ State Key Laboratory of Microbial Technology, Jiangsu Collaborative Innovation Center of Biomedical Functional Materials, School of Chemistry and Materials Science, Nanjing Normal University, Nanjing 210023, China

## Abstract

Although the copper-catalyzed allylic C­(sp^3^)–H oxygenation (Kharasch–Sosnovsky reaction) is a powerful transformation known for over a half century, its enantioselective version has been developed with drawbacks. High enantioselectivity was not achieved for acyclic terminal olefins and tertiary C–H bonds. Here we have addressed these long-standing challenges enabled by the design of new SphenBOX ligands. When combined with photocatalysis, a range of valuable allylic esters can be obtained directly from terminal olefins and carboxylic acids with high efficiency, regioselectivity, and enantioselectivity. Unprecedented enantioconvergent oxygenation of allylic tertiary C­(sp^3^)–H bonds is demonstrated, allowing construction of quaternary stereocenters with high enantioselectivity. Structural analysis indicates that SphenBOX features a compact chiral pocket that may contribute to its superior performance.

## Graphical abstract

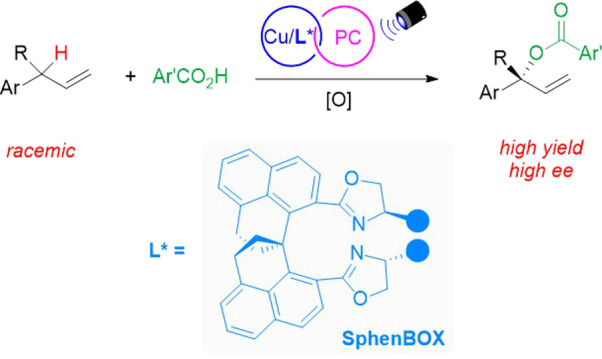

Direct enantioselective functionalization of inert C­(sp^3^)–H bonds is one of the holy grails in organic synthesis, owing to its extraordinary expediency in accessing valuable building blocks from simple substrates.^1^ Specifically, stereocontrolled C–O bond formation is particularly useful in view of the wide presence of oxygenated molecules in natural products and pharmaceuticals.^2–4^ Among them, enantioenriched allylic alcohol derivatives represent privileged structures. Thus, direct allylic C­(sp^3^)–H oxygenation provides the most attractive access in view of step- and atom-economy.^5^ However, intimidating challenges are involved, including the general inertness of C­(sp^3^)–H bonds and the requirement of control over site-selectivity, chemoselectivity (e.g., overoxidation, homocoupling) and enantioselectivity. Consequently, despite the persistent efforts in the past half century, there remain unsolved problems.^5–12^

While attempts by palladium catalysis have proved the feasibility of enantiocontrol for this transformation,^6^ an ideal level of enantioselectivity has not been achieved so far, except for sporadic intramolecular etherification cases.^7^ In 1958, Kharasch and Sosnovsky pioneered the use of copper catalysis for this reaction,^8^ which stimulated substantial efforts in copper-based enantioselective protocols (Scheme 1A).^9–11^ However, for a long time, excellent enantioselectivity has only been achieved for simple symmetric cyclic olefins, where the enantiocontrol benefits from restricted conformation and no site selectivity or *E/Z* control is necessary (Scheme 1B).^9^ Indeed, these protocols proved less effective for acyclic olefins.^10^ This limitation has remained unsolved until very recently.

**Scheme 1:**
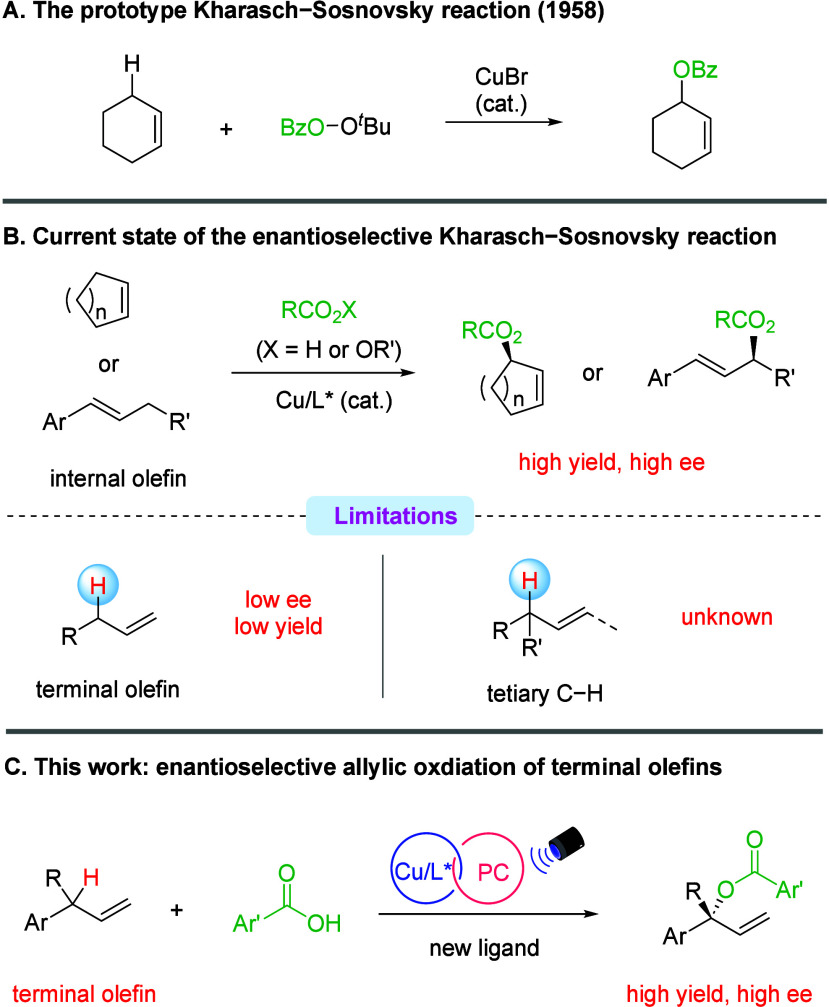
Introduction to Cu-Catalyzed Enantioselective Allylic C­(sp^3^)–H Oxygenation

In 2024, Kramer, Yu, Zhou, and Liu’s laboratories independently reported their breakthroughs in achieving both high efficiency and excellent enantioselectivity for acyclic olefins.^11^ A copper/BOX catalytic system, together with photo irradiation (except for Liu’s system),^11d^ on substituted styrenes constitutes the key features (Scheme 1B).

While these state-of-the-art achievements certainly represent a new milestone, it is worth mentioning that they also share a common limitation, i.e., no success was demonstrated for terminal olefins. Indeed, very low yield and enantioselectivity were explicitly documented in Kramer and Yu’s papers for terminal olefins (details shown in their Supporting Information).^11a,11b^ Moreover, current success has only been achieved for secondary allylic C­(sp^3^)–H bonds, but never tertiary ones. The latter would involve overwhelming steric repulsion during C–O bond formation (in the presence of other less hindered reactive sites or side reaction paths), not to mention the elusive enantiocontrol over the tetrasubstituted stereogenic center. In this context, here we report our progress in addressing these unsolved challenges enabled by new ligand design (Scheme 1C).

Chiral bis­(oxazoline) ligands^13^ have shown exceptional capability in the enantioselective Kharasch–Sosnovsky reactions.^9–11^ We hypothesized that new bis­(oxazoline) ligands with distinct geometry may help address the current limitations. Recently, we have developed a highly rigid spirocyclic skeleton, SPHENOL, which may provide unique geometry for bidentate ligands (Scheme 2).^14^ Thus, SPHENOL was first converted to the corresponding bis­(triflate) and then bis­(nitrile) by Pd-catalyzed cross-coupling. Subsequent reduction by DIBAL-H followed by oxidation delivered the dicarboxylic acid. Finally, condensation with a chiral amino alcohol, followed by cyclization of the amide intermediate provided the bis­(oxazoline) ligand, SphenBOX. Notably, all these steps are operationally simple and high yielding, although a shorter sequence could be envisioned. A series of nine different ligands (**L1**–**L9**) bearing different side arms were obtained.

**Scheme 2:**
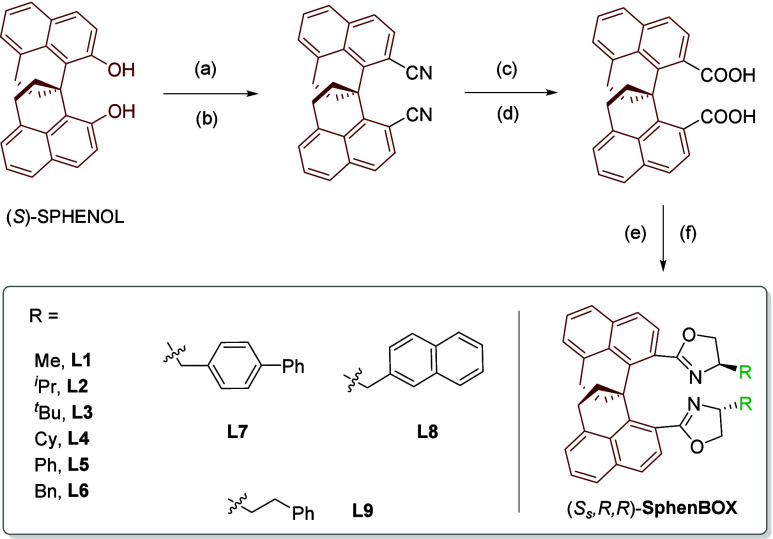
Design of SphenBOX Ligands^a^

a Conditions: (a) Tf_2_O, pyridine, DCM. (b) Zn­(CN)_2_, Pd­(^*t*^Bu_3_P)_2_/dppe, DMF. (c) (i) DIBAL-H, DCM; (ii) HCl (aq). (d) NaClO_2_, NaH_2_PO_4_·2H_2_O, THF/H_2_O/^*t*^BuOH. (e) (i) (COCl)_2_, DMF, DCM; (ii) amino alcohol, Et_3_N, DCM. (f) PPh_3_, Et_3_N, CCl_4_, MeCN.

We began to test our ligands for the model reaction of olefin **1a** and carboxylic acid **2a** (Table 1). Through extensive screening of reaction parameters (details in the SI), we found that the desired enantioselective allylic C­(sp^3^)–H oxygenation could be achieved by a dual copper/iridium photocatalytic system with (*S*_*s*_ *,R,R*)-**L7** as the optimal ligand and 420 nm LED as the light source. The desired allyl ester **3a** was obtained in 87% yield and 94% ee, together with exclusive regioselectivity (*b/l* > 20:1, entry 1).

**Table 1:**
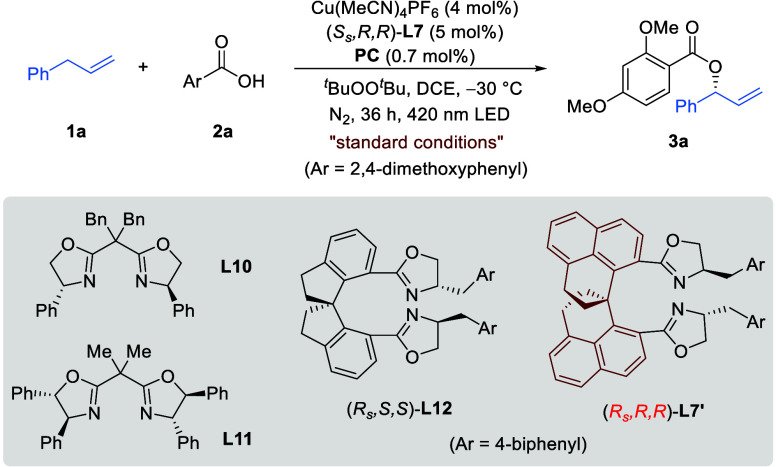
Reaction Condition Optimization^a^

entry	variation from the“standard conditions”	yield^b^ (%)	enantiomeric excess, ee^c^ (%)
1	–^a^	87​	94
2	without Cu(MeCN)_4_PF_6_	<5​	–
3	CuCl as Cu source	<5​	–
4	Cu(MeCN)_4_BF_4_ as Cu source	85​	93
5	without **L7**	46​^d^	0
6	**L1**–**L3** as ligand	<5​	–
7	**L4** as ligand	18​	0
8	**L5** as ligand	36​	–4
9	**L6** as ligand	78​	91
10	**L8** as ligand	83​	92
11	**L9** as ligand	58​	84
12	**L10** as ligand	60​	10
13	**L11** as ligand	56​	19
14	**L12** as ligand	66​	–59
15	(*R*_*s*_,*R*,*R*)-**L7′** as ligand	88​	–20
16	without **PC**	12​	93
17	4CzIPN instead of **PC**	8​	–
18	TXT instead of **PC**	80​	89
19	without ^*t*^BuOO^*t*^Bu	<5​	–
20	without light	<5​	–

a Standard conditions: Cu­(MeCN)_4_PF_6_ (4 μmol), **L7** (5 μmol), **PC** (0.7 μmol), **1a** (0.3 mmol), **2a** (0.1 mmol),^t^BuOO^*t*^Bu (0.4 mmol), DCE (0.25 mL), 6 W 420 nm blue LED, −30 °C, 36 h.

b Yields were determined by^1^H NMR analysis of the crude mixture with CH_2_Br_2_ as an internal standard.

c ee values were determined by chiral HPLC.

d *b*/*l* = 4.5:1. **PC** = Ir­[dF­(CF_3_)­ppy]_2_(dtbbpy)­PF_6_. 4CzIPN = 1,2,3,5-tetrakis­(carbazol-9-yl)-4,6-dicyanobenzene. TXT = thiaxanthenone.

Without Cu­(MeCN)_4_PF_6_ or replacing it with CuCl resulted in no reaction (entries 2 and 3 in Table 1). However, comparable results could be obtained with Cu­(MeCN)_4_BF_4_ (entry 4 in Table 1). A reduced catalyst loading resulted in low yield, but almost no influence on enantioselectivity (see the SI for details). In the absence of a chiral ligand, the reaction proceeded to form racemic **3a** in 46% yield, together with the linear isomer (entry 5 in Table 1). Other SphenBOX ligands were compared. **L1**–**L3** led to no desired product (entry 6 in Table 1). **L4** and **L5** provided **3a** in low yield and almost in racemic form (entries 7 and 8 in Table 1). In contrast, the benzyl- and 2-naphthylmethyl-substituted ligands (**L6** and **L8**) gave both high yield and excellent enantioselectivity (entries 9 and 10 in Table 1), almost comparable to **L7**. However, homobenzyl-substituted **L9** led to obvious decrease in enantioselectivity (entry 11 in Table 1). These results indicated that a suitably extended arene in the side arm was essential to excellent enantiocontrol. We also evaluated the BOX ligands **L10** and **L11**, which proved effective in the previous reported cases for internal olefins,^11^ but they resulted in very low enantioselectivities (10% ee and 19% ee; see entries 12 and 13 in Table 1). We also prepared the SPINOL-derived bis­(oxazoline) **L12** bearing the same side arm on the oxazoline ring as **L7**.^15^ However, the reaction gave only 59% ee (entry 14 in Table 1). These results not only corroborated the distinct reactivities of terminal olefin (vs internal ones) but also highlighted the unique performance of the SPHENOL backbone. Furthermore, the diastereomeric ligand (*R*_*s*_,*R*,*R*)-**L7′** was also evaluated, but dramatic decrease in enantioselectivity was observed (20% ee; see entry 15 in Table 1), suggesting that matching the chirality of the oxazoline motif with the spiro chirality was also essential.

Further control experiments indicated that the iridium photocatalyst was crucial for chemical efficiency. Removing or replacing it with 4CzIPN resulted in dramatically low yield (entries 16 and 17 in Table 1). However, thiaxanthenone (TXT) provided good yield and enantioselectivity (entry 18 in Table 1). Other photocatalysts with a lower triplet state energy resulted in trace product (see the SI for details). Finally, the reaction did not proceed in the absence of the oxidant or light (entries 19 and 20 in Table 1). These results suggest that **PC** serves as an effective triplet state energy transfer reagent in this process.

With **2a** as the partner, various substituted allylbenzenes all reacted to form the corresponding allylic esters with good efficiency and high enantioselectivity (**3a**–**3j**, Scheme 3).^16^ *Meta*- or *para*-substituents on the benzene ring did not affect the excellent enantioselectivity, but an *ortho*-methyl group led to diminished yield and ee (**3j**). This reaction was also applicable to 2-allylnaphthalene (**3k**). Other carboxylic acids (**3l**–**3q**) were also investigated. Benzoic acids bearing an electron-donating substituent at any position could provide good to excellent outcomes (**3l**–**3p**). Unfortunately, substitution with an alkyl group at the allylic position resulted in moderate efficiency and enantioselectivity (**3r** and **3s**).

**Scheme 3:**
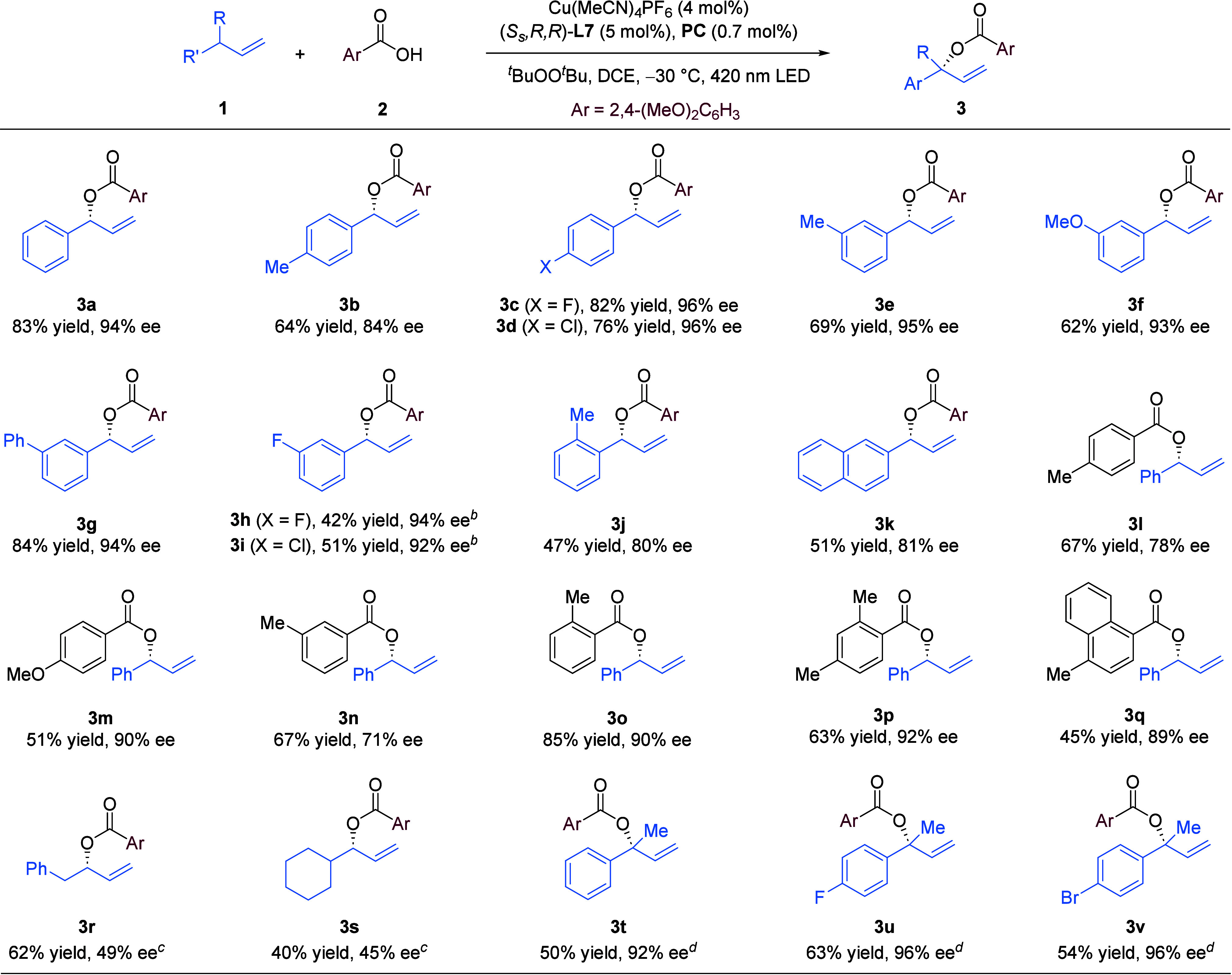
Reaction Scope^a^

a Reaction conditions: Cu­(MeCN)_4_PF_6_ (0.012 mmol), **L7** (0.015 mmol), **PC** (2.1 μmol), **1** (0.9 mmol), **2** (0.3 mmol), ^*t*^BuOO^*t*^Bu (1.2 mmol), DCE (0.75 mL), 6 W 420 nm blue LED, −30 °C, 3–5.5 days. Isolated yield. The ee value was determined by chiral HPLC.
b
**1** (1.8 mmol), Cu­(MeCN)_4_PF_6_ (0.024 mmol), **L7** (0.03 mmol).
c
**1** (1.5 mmol).
d –50 °C for 5 days.

We were also interested in probing the capability of our protocol in generating sterically hindered C–O bonds. It is worth noting that direct enantioconvergent functionalization of an inert tertiary C­(sp^3^)–H bond to form a quaternary stereogenic center remains a largely uncharted territory.^3j,17^ To our delight, our ligand system showed preliminary success in this regard. The corresponding allylic esters **3t**–**3v** were formed with respectable yield and excellent enantioselectivity, which represent the first highly enantioselective tertiary allylic C­(sp^3^)–H bond oxidation.

The enantioenriched products **3a** and **3t** can be easily converted to other chiral building blocks (Scheme 4). For example, hydrolysis of **3a** with NaOMe afforded allylic alcohol **4** in high yield. Hydroboration of the terminal olefin followed by oxidation furnished 1,3-diol monoester **5**. Dihydroxylation with K_2_OsO_2_(OH)_4_ followed by C–C bond cleavage with NaIO_4_ resulted in α-acyloxy aldehyde **6**. Regioselective difluoromethylation was achieved via a photocatalytic system to deliver difluoro ester **7**. Ir-Catalyzed hydroboration of **3t** with HBpin resulted in boronate **8**. Reduction of **3t** by DIBAL-H afforded tertiary allylic alcohol **9**. With the second-generation Hoveyda–Grubbs catalyst, it reacted with ethyl acrylate to give α,β-unsaturated ester **10** bearing a remote tertiary alcohol functionality. Notably, the high enantiopurity remained intact in all these transformations.

**Scheme 4:**
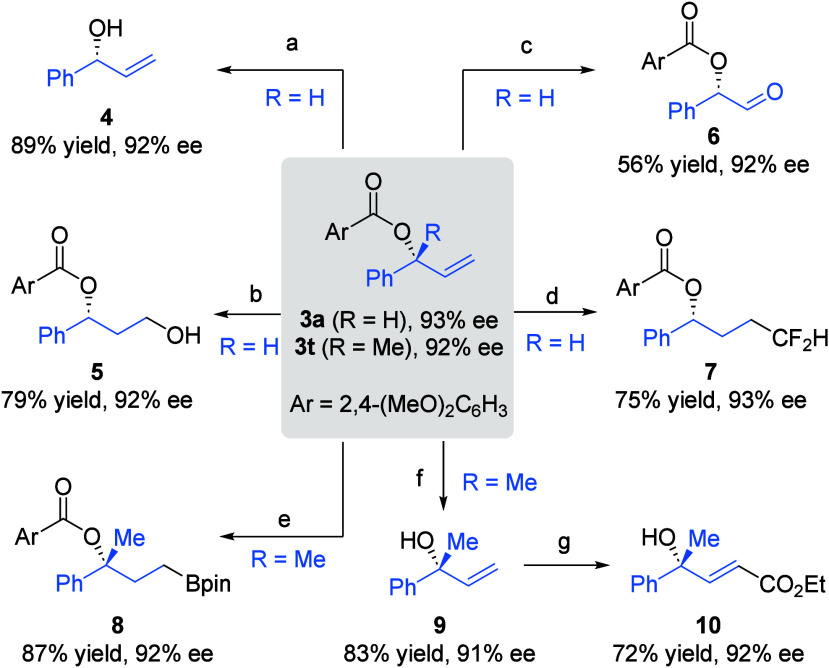
Product Transformations^a^

a Pathway (a): NaOMe, MeOH. Pathway (b): (i) 9-BBN; (ii) NaBO_3_·4H_2_O. Pathway (c): K_2_OsO_2_(OH)_4_, NMO, NaIO_4_, ^*t*^BuOH/H_2_O. Pathway (d): Fe­(acac)_3_, 2,4,6-(^*i*^Pr)_3_C_6_H_2_SH, CF_2_HCO_2_H, DMSO, *hv*. Pathway (e): [Ir­(COD)­Cl]_2_, dppm, HBpin, DCM. Pathway (f): DIBAL-H, DCM. Pathway (g): The second-generation Hoveyda–Grubbs catalyst, ethyl acrylate, DCM.

Control experiments were performed to understand the mechanism (Figure 1). When a radical scavenger (TEMPO or 1,1-diphenylethylene) was added, the desired reaction was inhibited (Figure 1a). The formation of side products **11** and **12** suggested the involvement of allylic and ^*t*^BuO^•^ radicals, respectively. The competition between **1c** and **1c**-*d*_2_ suggested that C–H bond cleavage might be the rate-determining step (*k*_H_/*k*_D_ = 2.6, Figure 1b). Furthermore, the product ee values showed a linear correlation with the ligand ee values, consistent with only one ligand molecule dictating the enantioselectivity (Figure 1c). Further study indicated that the reaction progress required persistent light irradiation, thus excluding a radical chain pathway (Figure 1d).

**Figure 1:**
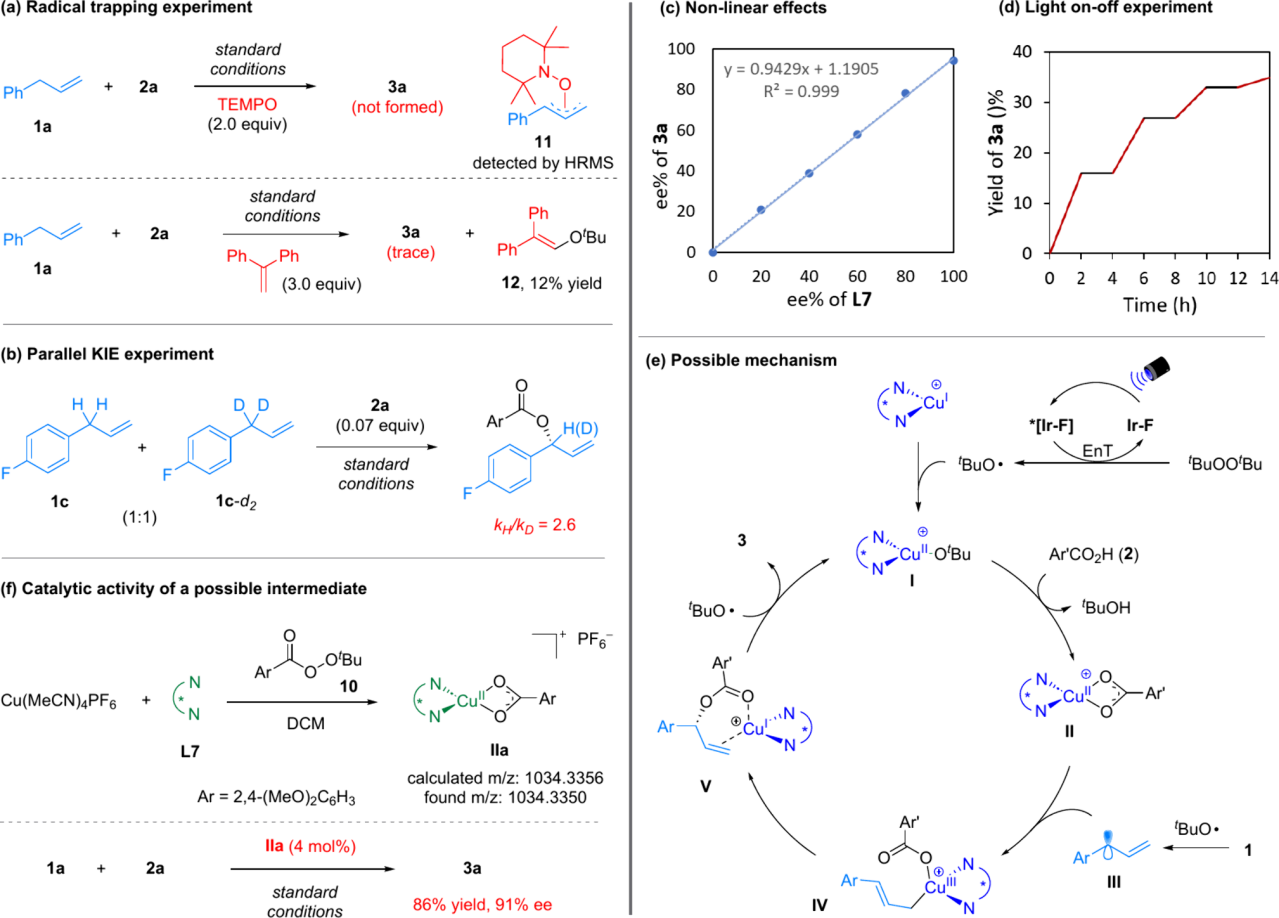
Mechanistic studies and proposed mechanism.

Based on the above results and related literature,^11,18^ we proposed a mechanism (Figure 1e). The cooperation of light and photocatalyst triggers homolytic cleavage of ^*t*^BuOO^*t*^Bu to generate ^*t*^BuO^•^ radical, which oxidizes the Cu­(I)/**L7** complex to form Cu­(II)-complex **I**. Ligand exchange with **2** results in Cu­(II)-complex **II**. Separately, the allylic radical **III** is generated via H-abstraction by ^*t*^BuO^•^, which traps complex **II** to form the key Cu­(III) species **IV**. The copper preferentially binds with the terminal carbon due to steric effect. Subsequent allylic reductive elimination, in a manner like sigmatropic rearrangement,^9a^ forms Cu­(I)-bound allylic ester **V** with enantiocontrol. This explains the high regioselectivity favoring the C–O bond formation at a hindered position, particularly for the tertiary C–H cases. Further oxidation by ^*t*^BuO^•^ regenerates the Cu­(II) complex **I** and releases product **3**.

To gain more insights, the Cu­(II) species **IIa** was synthesized by mixing Cu­(MeCN)_4_BF_4_, **L7**, and peroxide **10**. Delightedly, **IIa** showed comparable catalytic activity in the standard reaction (Figure 1f).

Finally, we were interested in probing the origin of the high performance of our SphenBOX ligands. We managed to obtain X-ray crystallographic structures of **L7**- and **L12**-bound copper complexes (Figure 2). Their structures were compared. In the Cu/SphenBOX complex, the naphthalene rings have a smaller dihedral angle (55.7°). Similarly, the bite angle (94.3°) and the cone angle (205.8°) are both smaller than those in the SPINOL-bis­(oxazoline). In addition, while the two Cu–N bonds are comparably long in these two complexes, the N**···**N distance in **L7**·Cu­(OAc)_2_ is obviously shorter. To make a more intuitive comparison of chiral induction model of **L7·Cu­(OAc)**_**2**_ and **L12·Cu­(OAc)**_**2**_, we have depicted the steric map of the chiral pockets^19^ that may help recognize the prochiral faces of the allylic radical (Figure 3). In the four-quadrant diagrams of the chiral pockets created by **L7·Cu­(OAc)**_**2**_ and **L12·Cu­(OAc)**_**2**_, respectively, the regions with significant steric hindrance are primarily located in the northwest quadrant (NW, 59.0% vs 58.1% buried) and the southeast quadrant (SE, 61.1% vs 58.5% buried), with the former being relatively more crowded. In contrast, the northeast quadrant (NE, 42.3% vs 43.8% buried) and southwest quadrant (SW, 45.6% vs 42.3% buried) of both pockets exhibit less steric hindrance. Notably, the steric hindrance in the southwest quadrant of **L7·Cu­(OAc)**_**2**_ is greater than that of **L12·Cu­(OAc)**_**2**_. Consequently, the steric map analysis indicates a larger buried volume of 52.0% for **L7·Cu­(OAc)**_**2**_, compared to 50.7% for **L12·Cu­(OAc)**_**2**_. Therefore, all the data suggested that the SphenBOX ligand has a more compact pocket, thus creating a tighter coordination environment, which might explain its outstanding performance.

**Figure 2:**
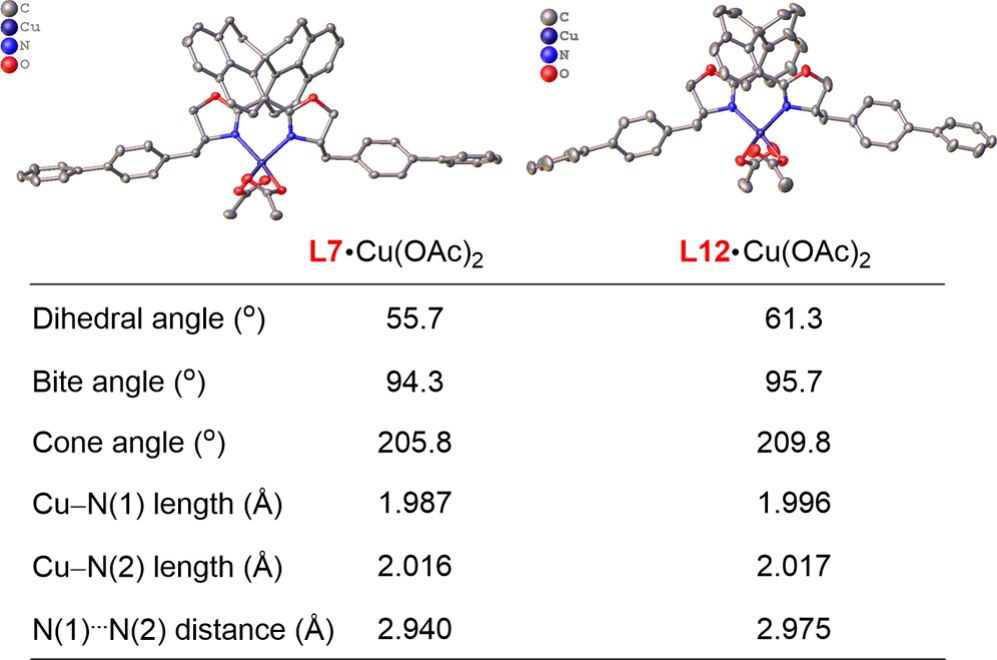
Structures of the **Cu/L7** and **Cu/L12** complexes.

**Figure 3:**
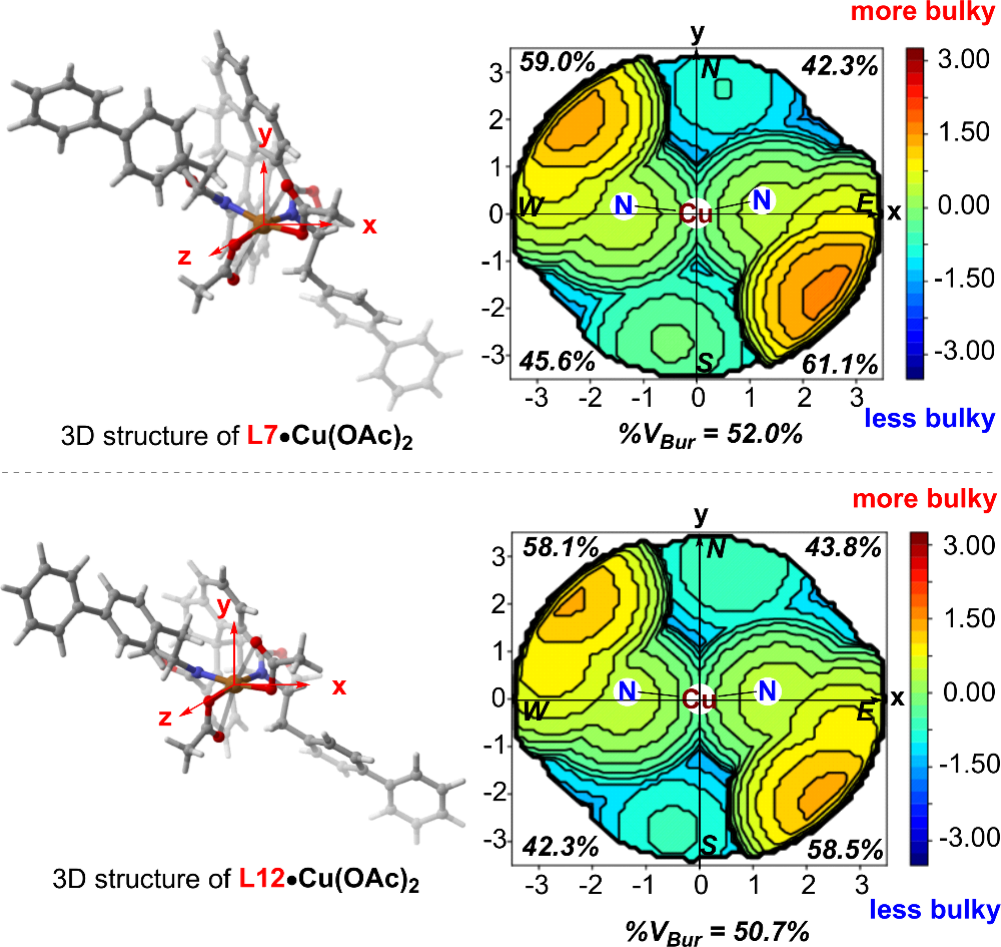
Comparison of the steric maps of **L7·Cu­(OAc)**_**2**_ and **L12·Cu­(OAc)**_**2**_. The orientation is indicated in the left panel. The steric maps are viewed down the *z*-axis on the right panel. The term “%V_Bur_” represents the percentage of buried volume.

In summary, although direct enantioselective allylic C–H oxygenation represents a powerful process receiving tremendous attention for over a half century, current available strategies have been developed with drawbacks, particularly with terminal olefins and tertiary C–H oxidation. Enabled by the design of a family of new rigid chiral SphenBOX ligands, we have addressed these remaining longstanding challenges. A range of highly enantioenriched allylic esters were synthesized directly from acyclic terminal olefins and carboxylic acids. The new SphenBOX ligands showed unique advantages over the known BOX ligands as well as the analogues derived from SPINOL in both chemical efficiency and enantioselectivity. Detailed analysis of their copper complexes indicated that they create a tight coordination environment that contributes to the superior performance. Our protocol is also applicable to the enantioconvergent oxidation of allylic tertiary C–H bonds, leading to sterically hindered oxygenated quaternary stereocenters with high chemoselectivity, regioselectivity, and enantioselectivity. This is not only the first demonstration in allylic oxygenation, but also a rare example of enantioselective functionalization of tertiary C–H bonds in general.

## Supplementary Material



## Notes

Experimental procedures, spectroscopic data for substrates and products, and crystallographic data for **L7·Cu­(OAc)**_**2**_ and **L12·Cu­(OAc)**_**2**_ (PDF)
