# Supplementary material for: Photoinduced Copper-Catalyzed Enantioselective Allylic C(sp 3)–H Oxygenation of Acyclic Terminal Olefins Enabled by SphenBOX
Source: J Am Chem Soc. 2025 Jun 9;147(24):20225–32. doi: 10.1021/jacs.5c06136 (PMC12186482; doi:10.1021/jacs.5c06136)
Supplement: Supplementary file 1 [file ja5c06136_si_001.pdf]

Supporting Information

Photoinduced Copper-Catalyzed Enantioselective Allylic C(sp^3)–H Oxygenation of Acyclic Terminal Olefins Enabled by SphenBOX

Xu-Kuan Qi,[1†] Qiang Dai,[1,2†] Yangyi Gu,[1] Kwan Shing Lau,[1] Herman H. Y. Sung,[1] Ian D. Williams,[1] Zhenyang Lin,[1] Chaoshen Zhang,[1*] and Jianwei Sun[1*]

[1]*Department of Chemistry and the Hong Kong Branch of Chinese National Engineering Research Centre for Tissue Restoration & Reconstruction, The Hong Kong University of Science and Technology, Clear Water Bay, Kowloon, Hong Kong SAR, China*

[2]*State Key Laboratory of Microbial Technology, Jiangsu Collaborative Innovation Center of Biomedical Functional Materials, School of Chemistry and Materials Science, Nanjing Normal University, Nanjing 210023, China.*

[†]*These authors contributed equally to this work.*

Table of Contents

I. General Information **S-2**

II. Synthesis of the Substrates **S-3**

III. Synthesis of the SphenBox Ligands **S-4**

IV. Details of Condition Optimization **S-15**

V. Catalytic Enantioselective Allylic C(*sp*³)–H Oxidation **S-23**

VI. Mechanistic Study **S-44**

VII. Product Derivatizations **S-57**

VIII. Determination of Product Structures **S-64**

IX. References **S-102**

NMR Spectra and HPLC Traces

I. General Information

Flash column chromatography was performed over silica gel (200-300 or 300-400 mesh) purchased from Qingdao Haiyang Co., China. All air or moisture sensitive reactions were conducted in oven-dried glassware or plastic vessel under nitrogen atmosphere using anhydrous solvents. Tetrahydrofuran was distilled from sodium/benzophenone. Anhydrous dichloromethane, acetonitrile, and *n*-hexane were purified by the Innovative® solvent purification system. Other anhydrous solvents were purchased from Sigma-Aldrich®, J&K® and Energy® and used as received. Chemicals were purchased from commercial suppliers, such as Sigma-Aldrich®, J&K®, Energy® and used without further purification unless otherwise stated. Chiral organocatalysts were purchased from Daicel® Chiral Technologies (China) Co., Ltd. and used as received. NMR spectra were recorded with a Bruker AVII, AVIII, 100 MHz (^{13}C NMR) and 376 MHz (^{19}F NMR). Chemical shifts (δ values) were reported in ppm down field from an internal standard (^{1}H NMR: Me_4Si at 0.00 ppm and ^{13}C NMR: $CDCl_3$ at 77.00 ppm, ^{1}H NMR: DMSO-d_6 at 2.50 ppm and ^{13}C NMR: DMSO-d_6 at 39.5 ppm). Data for ^{1}H NMR were recorded as follows: chemical shift (δ, ppm), multiplicity (s = singlet; d = doublet; t = triplet; q = quarter; p = pentet; m = multiplet; br = broad), coupling constant (Hz), integration. Mass spectra were collected on an Agilent GC/MS 5975C system, or a MALDI Micro MX mass spectrometer, or an API QSTAR XL System. Optical rotations were measured on a JASCO P-2000 polarimeter or an AUTOPOL I Automatic polarimeter with $[\alpha]_D$ values reported in degrees; concentration (c) is in 10 mg/mL. Enantioselectivities were recorded on an Agilent HPLC instrument, using a chiral stationary phase column (Daicel CHIRALPAK® AD-3, IC-3, ID-3, IG-3, CHIRALCEL® OD-3, OJ-3). The chiral HPLC methods were calibrated with the corresponding racemic mixtures.

II. Synthesis of the Substrates

The acyclic olefins (**1**)[1-3] and chiral amino alcohol[4] were prepared according to the literature procedure. Unless otherwise noted, carboxylic acids (**2**) were used as received from commercial sources without further purification.

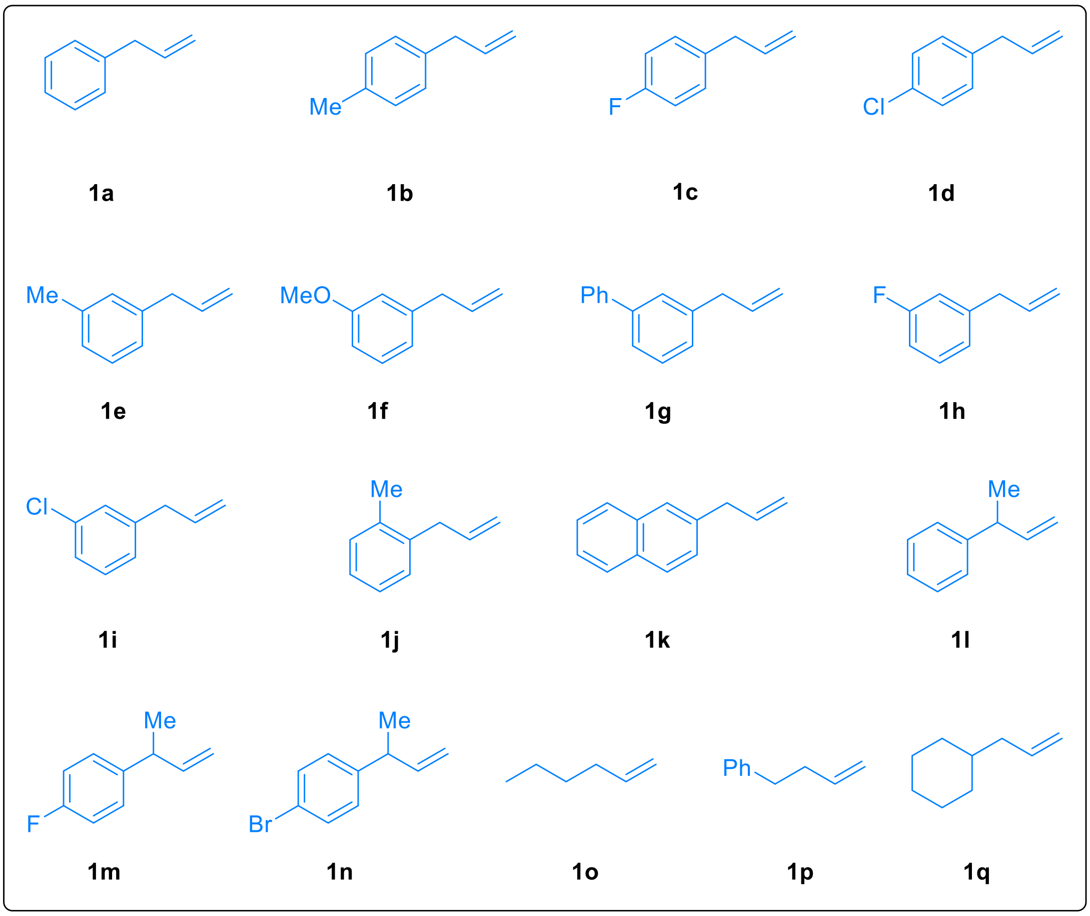

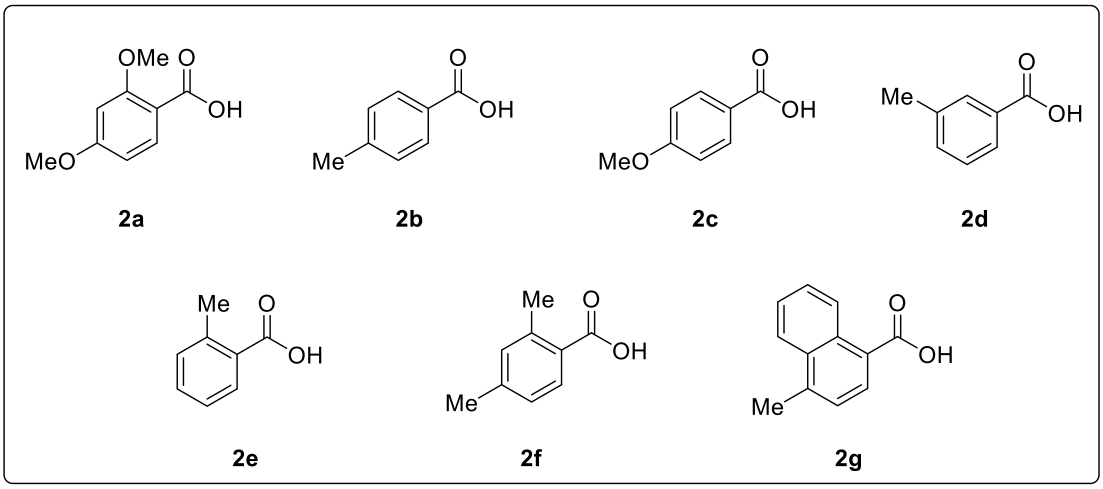

III. Synthesis of the SphenBox Ligands

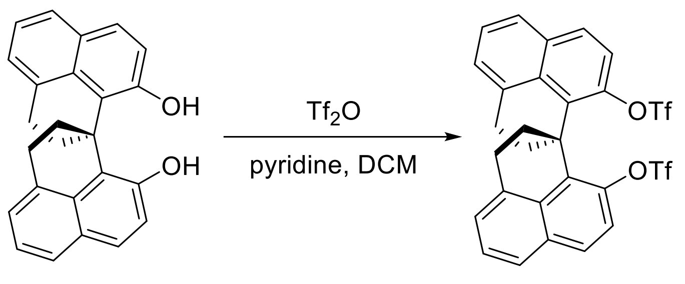

(*S*)-2,2',3,3'-Tetrahydro-1,1'-spirobi[phenalene]-9,9'-diyl bis(trifluoro methanesulfonate) ((*S*)-1). To a solution of *(S)*-SPHENOL (7.05 g, 20.0 mmol) and pyridine (7.2 mL, 88.0 mmol) in CH_2Cl_2 (100 mL), trifluoromethanesulfonic anhydride (7.4 mL, 44.0 mmol) was added slowly at 0 ^{o}C. The mixture was stirred at room temperature for 3 h. After removal of the solvent, the residue was diluted with CH_2Cl_2 (200 mL) and sequentially washed with a HCl solution (5% aq.), a saturated aqueous $NaHCO_3$ solution, and brine. The organic layer was dried over anhydrous $MgSO_4$, filtered, and concentrated under reduced pressure. The residue was purified by silica gel flash chromatography (EtOAc/*n*-hexane = 1:5) to afford *(S)*-**1** (12.14 g, 98% yield) as a white solid.

^{1}H NMR (400 MHz, $CDCl_3$) δ 7.81 (dd, J = 17.4, 8.7 Hz, 4H), 7.55 – 7.46 (m, 2H), 7.44 – 7.35 (m, 4H), 3.47 – 3.28 (m, 2H), 3.17 (dt, J = 16.6, 3.2 Hz, 2H), 2.54 (dd, J = 14.2, 1.9 Hz, 2H), 2.36 (td, J = 14.1, 4.0 Hz, 2H) ppm.

^{13}C NMR (101 MHz, $CDCl_3$) *δ* 143.6, 134.4, 132.8, 132.4, 129.9, 129.1, 126.6, 126.3, 125.9, 118.5, 117.7 (q, *J* = 318.0 Hz), 40.8, 31.5, 26.1 ppm.

^{19}F NMR (377 MHz, $CDCl_3$) δ -75.04 ppm.

HRMS (CI+) Calcd for $C_{27}H_{18}F_6O_6SNa$ $[M+Na]^+$: 639.0347, Found: 639.0347.

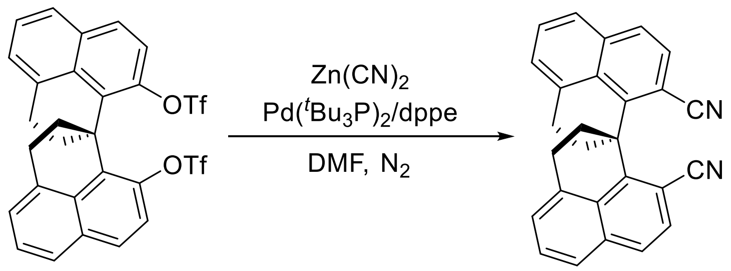

(*S*)-**1** (*S*)-**2**

(*S*)-2,2',3,3'-Tetrahydro-1,1'-spirobi[phenalene]-9,9'-dicarbonitrile ((*S*)-2). Under N_2, a mixture of Pd(P^{t}Bu$_3$)$_2$ (261.6 mg, 0.5 mmol), dppe (398.5 mg, 1.0 mmol) in DMF (15 mL) was stirred at room temperature for 10 min. Then, the bis(triflate) (*S*)-**1** (3.08 g, 5.0 mmol) and Zn(CN)$_2$ (3.6 g, 30.1 mmol) were added. The reaction mixture was stirred at 150 °C for 66 h and then cooled to room temperature. It was diluted with CH_2Cl_2, washed sequentially with an aqueous Na_2CO_3 solution and brine, dried over anhydrous $MgSO_4$, and concentrated. The residue was purified by silica gel flash chromatography (EtOAc/*n*-hexane = 5:1 to 2:1) to afford *(S)*-**2** (1.74 g, 94% yield) as a white solid.

^{1}H NMR (400 MHz, $CDCl_3$) δ 7.87 (d, *J* = 8.5 Hz, 2H), 7.81 (d, *J* = 8.2 Hz, 2H), 7.61 – 7.54 (m, 4H), 7.46 (d, *J* = 7.0 Hz, 2H), 3.47 – 3.39 (m, 2H), 3.26 – 3.20 (m, 2H), 2.69 – 2.57 (m, 4H) ppm.

^{13}C NMR (101 MHz, $CDCl_3$) δ 150.0, 135.2, 134.8, 129.8, 128.5, 128.4, 128.3, 127.0, 126.2, 118.1, 106.9, 45.3, 32.8, 26.4 ppm.

HRMS (CI+) Calcd for $C_{27}H_{18}N_2Na$ [M+Na]$^+$: 393.1362, Found: 393.1361.

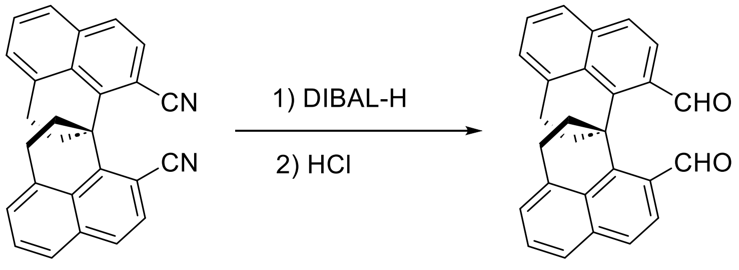

(*S*)-**2** (*S*)-**3**

(*S*)-2,2',3,3'-Tetrahydro-1,1'-spirobi[phenalene]-9,9'-dicarbaldehyde ((*S*)-3). At –78 °C under N_2, a solution of DIBAL-H in *n*-hexane (1.0 M, 15.0 mL, 15.0 mmol)

was added slowly to a solution of *(S)*-**2** (1.85 g, 5.0 mmol) in dry CH_2Cl_2 (50 mL). The reaction was stirred at –78 °C for 1 h and then at 50 °C for 8 h. The reaction mixture was cooled to 0 °C and quenched with dropwise addition of an aqueous HCl solution (30 mL, 2N). The mixture was stirred at room temperature for 12 h and then extracted with CH_2Cl_2. The organic layer was washed with brine, dried over anhydrous $MgSO_4$, and concentrated. The residue was purified by silica gel flash chromatography (DCM/*n*-hexane = 2:1 to 1:1) to afford *(S)*-**3** (1.64 g, 87% yield) as a white solid.

^{1}H NMR (400 MHz, $CDCl_3$) δ 9.57 (s, 2H), 7.89 – 7.77 (m, 6H), 7.58 (t, *J* = 7.2 Hz, 2H), 7.44 (d, *J* = 7.0 Hz, 2H), 3.52 (dt, *J* = 17.6 Hz, *J* = 4.2 Hz, 2H), 3.21 (dt, *J* = 15.5 Hz, *J* = 3.8 Hz, 2H), 2.80 (dq, *J* = 14.4 Hz, *J* = 2.4 Hz, 2H), 2.58 (dt, *J* = 14.4 Hz, *J* = 4.1 Hz, 2H) ppm.

^{13}C NMR (101 MHz, $CDCl_3$) δ 190.3, 150.4, 136.8, 135.5, 128.3 (2C), 127.7, 127.5, 127.2, 126.1, 124.4, 43.5, 36.5, 26.4 ppm.

HRMS (CI+) Calcd for $C_{27}H_{20}NaO_2$ [M+Na]$^+$: 399.1356, Found: 399.1360.

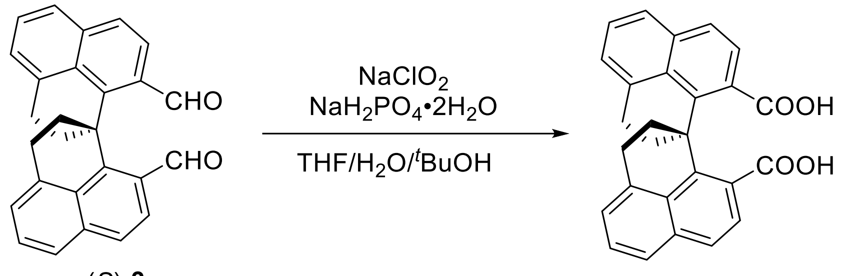

(*S*)-**3** (*S*)-**4**

(*S*)-2,2',3,3'-Tetrahydro-1,1'-spirobi[phenalene]-9,9'-dicarboxylic acid ((*S*)-4). At room temperature, to a solution of aldehyde *(S)*-**3** (1.88 g, 5.0 mmol) in THF (75 mL) were added $NaClO_2$ (2.71 g, 30.0 mmol, 6.0 equiv), 2-methylbut-2-ene (10.7 mL, 75 mmol, 13.0 equiv), and ^{t}BuOH (150 mL). The mixture was stirred vigorously for 5 min, and then a solution of $NaH_2PO_4 \cdot 2H_2O$ (6.24 g, 40.0 mmol, 8.0 equiv) in water (25 mL) was added dropwise. The mixture was heated to 35 °C with vigorous stirring for 12 h with exclusion of light. The mixture was then concentrated before the addition of an aqueous solution of HCl (2 M) to pH =

1, which was then extracted with EtOAc (50 mL x 3). The combined organic layers were dried over anhydrous $MgSO_4$, filtered and concentrated. The residue was purified by silica gel flash chromatography (*n*-hexane/EtOAc/AcOH = 1:1:0.02) to give *(S)*-**4** (1.83 g, 90% yield) as a white foam.

^{1}H NMR (400 MHz, $CDCl_3$) δ 7.68 (d, *J* = 8.6 Hz, 2H), 7.59 (d, *J* = 8.1 Hz, 2H), 7.46 – 7.37 (m, 4H), 7.32 (d, *J* = 7.0 Hz, 2H), 6.33 (s, 2H), 3.36 (td, *J* = 15.2, 14.6, 4.0 Hz, 2H), 3.10 – 2.88 (m, 4H), 2.31 (dd, *J* = 13.3, 3.4 Hz, 2H) ppm.

^{13}C NMR (101 MHz, $CDCl_3$) δ 173.6, 145.0, 135.7, 134.8, 129.8, 127.1, 126.8, 126.4, 125.9 (3C), 44.5, 31.3, 26.7 ppm.

HRMS (CI+) Calcd for $C_{27}H_{19}O_4$ $[M–H]^-$: 407.1289, Found: 407.1283.

General Procedure A.

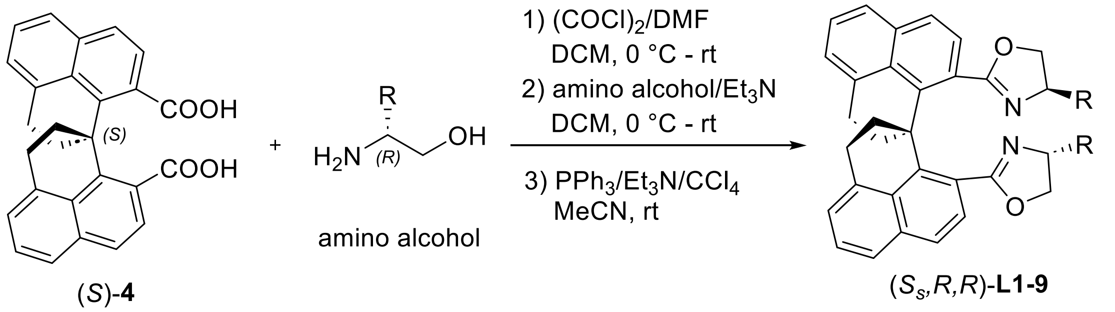

At 0 °C, to a 100-mL Schlenk® flask charged with a suspension of *(S)*-**4** (408 mg, 1.0 mmol) in dry dichloromethane (10 mL) was added three drops of dry DMF followed by dropwise addition of oxalyl chloride (2.7 mL, 5.0 mmol). The resulting mixture was stirred at room temperature. Upon completion (~3 h), the solvent was removed under reduced pressure to afford the crude acyl chloride as a light-yellow solid, which was then re-dissolved in dichloromethane (20 mL). In a separate flask, a stirred solution of the chiral amino alcohol (2.2 mmol) and Et_3N (0.65 mL, 5.0 mmol) in dichloromethane (10 mL) was cooled to 0 °C, to which was added the acyl chloride solution over 10 min under nitrogen. The

reaction mixture was slowly warmed to room temperature and stirred overnight. The reaction mixture was then diluted with dichloromethane (20 mL) and washed by water (30 mL) and brine (30 mL). The organic layer was dried over Na_2SO_4, filtered and concentrated under reduced pressure to afford the crude amide, which was used directly in the next step without any purification.

A mixture of the crude amide, triphenylphosphine (834.8 mg, 3.0 mmol), triethylamine (0.39 mL, 2.8 mmol), and tetrachloromethane (430.7 mg, 2.8 mmol) was dissolved in dry acetonitrile (20 mL) and stirred at room temperature for 36 h. The mixture was concentrated under reduced pressure. The residue was dissolved with CH_2Cl_2 (50 mL), washed with water, dried over anhydrous $MgSO_4$, and then concentrated under reduced pressure. The residue was purified by silica gel flash chromatography to give the pure spiro bis(oxazoline) ligand.

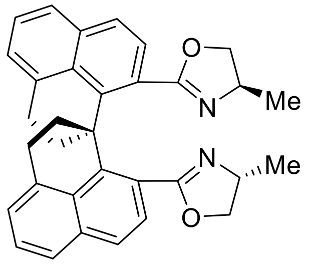

(S_s,*R*,*R*)-**L1**

(*S*)-9,9'-Bis((*R*)-4-methyl-4,5-dihydrooxazol-2-yl)-2,2',3,3'-tetrahydro-1,1'-spirobi[phenalene] ((S_s,*R*,*R*)-L1) was prepared as a white foam according to the General Procedure A (eluent: *n*-hexane/EtOAc = 3:1) in 54% yield (263.2 mg). $[\alpha]_D^{25}$: –298.9 (c = 0.5, $CHCl_3$).

^{1}H NMR (400 MHz, $CDCl_3$) δ 7.68 – 7.66 (m, 4H), 7.45 (t, J = 7.1 Hz, 2H), 7.39 (d, J = 8.4 Hz, 2H), 7.35 (d, J = 6.9 Hz, 2H), 3.70 (dt, J = 13.9 Hz, J = 4.3 Hz, 2H), 3.44 – 3.32 (m, 4H), 3.10 (dd, J = 16.3 Hz, J = 2.2 Hz, 2H), 2.86 (t, J = 8.6 Hz, 2H), 2.79 – 2.69 (m, 2H), 2.47 – 2.43 (m, 2H), 0.84 (d, J = 6.6 Hz, 6H) ppm.

^{13}C NMR (101 MHz, $CDCl_3$) δ 164.3, 144.3, 136.1, 133.9, 130.3, 127.9, 126.1, 125.9,

125.8, 125.4, 125.0, 72.8, 60.7, 44.5, 32.6, 27.0, 20.6 ppm.

HRMS (CI+) Calcd for $C_{33}H_{31}N_2O_2$ $[M+H]^+$: 487.2380, Found: 487.2385.

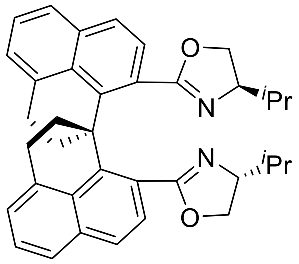

(S_s,*R*,*R*)-**L2**

(*S*)-9,9'-Bis((*R*)-4-isopropyl-4,5-dihydrooxazol-2-yl)-2,2',3,3'-tetrahydro-1,1'-spirobi[phenalene] ((S_s,*R*,*R*)-L2) was prepared as a white foam according to the General Procedure A (eluent: *n*-hexane/EtOAc = 10:1) in 47% yield (252.6 mg). $[\alpha]_D^{25}$: –196.2 (*c* = 0.5, $CHCl_3$).

^{1}H NMR (400 MHz, $CDCl_3$) δ 7.68 – 7.64 (m, 4H), 7.44 (t, *J* = 7.1 Hz, 2H), 7.35 – 7.33 (m, 4H), 3.92 (dt, *J* = 13.7 Hz, *J* = 4.2 Hz, 2H), 3.45 – 3.31 (m, 4H), 3.26 – 3.22 (m, 2H), 3.09 (dd, *J* = 16.2 Hz, *J* = 2.2 Hz, 2H), 2.48-2.44 (m, 2H), 2.11 – 2.04 (m, 2H), 1.35 – 1.27 (m, 2H), 0.60 (d, *J* = 6.7 Hz, 6H), 0.54 (d, *J* = 6.7 Hz, 6H) ppm.

^{13}C NMR (101 MHz, $CDCl_3$) δ 163.7, 144.9, 136.3, 133.9, 130.5, 127.4, 126.0, 125.71, 125.66, 125.0, 124.8, 71.7, 69.1, 44.6, 32.5, 31.7, 27.1, 18.8, 18.0 ppm.

HRMS (CI+) Calcd for $C_{37}H_{39}N_2O_2$ $[M+H]^+$: 543.3006, Found: 543.3013.

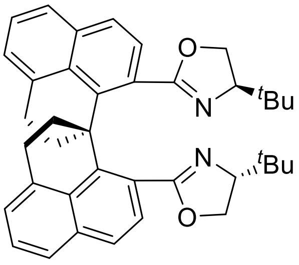

(S_s,*R*,*R*)-**L3**

(*S*)-9,9'-Bis((*R*)-4-(*tert*-butyl)-4,5-dihydrooxazol-2-yl)-2,2',3,3'-tetrahydro-1,1'-spirobi[phenalene] ((S_s,*R*,*R*)-L3) was prepared as a white foam according to the General Procedure A (eluent: *n*-hexane/EtOAc = 10:1) in 44% yield (248.6

mg). $[\alpha]_D^{25}$: –144.4 (c = 0.5, $CHCl_3$).

^{1}H NMR (400 MHz, $CDCl_3$) δ 7.68 – 7.64 (m, 4H), 7.44 (t, J = 7.1 Hz, 2H), 7.33 (d, J = 8.2 Hz, 4H), 4.04 (dt, J = 13.8 Hz, J = 4.2 Hz, 2H), 3.53 (t, J = 8.6 Hz, 2H), 3.41 (dt, J = 15.6 Hz, J = 4.2 Hz, 2H), 3.16 (t, J = 8.8 Hz, 2H), 3.08 (dd, J = 16.1 Hz, J = 2.1 Hz, 2H), 2.47 (dd, J = 13.4 Hz, J = 2.4 Hz, 2H), 1.89 (t, J = 9.8 Hz, 2H), 0.53 (s, 18H) ppm.

^{13}C NMR (101 MHz, $CDCl_3$) δ 163.2, 145.2, 136.5, 133.9, 130.7, 127.1, 126.0, 125.64, 125.57, 125.0, 124.7, 75.1, 67.3, 44.7, 32.5, 32.3, 27.1, 25.6 ppm.

HRMS (CI+) Calcd for $C_{39}H_{43}N_2O_2$ $[M+H]^+$: 571.3319, Found: 571.3327.

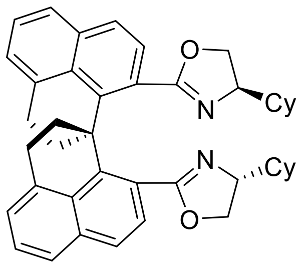

(S_s,R,R)-**L4**

(S)-9,9'-Bis((R)-4-cyclohexyl-4,5-dihydrooxazol-2-yl)-2,2',3,3'-tetrahydro-1,1'-spirobi[phenalene] ((S_s,R,R)-L4) was prepared as a white foam according to the General Procedure A (eluent: n-hexane/EtOAc = 10:1) in 50% yield (313.9 mg). $[\alpha]_D^{25}$: –197.9 (c = 0.5, $CHCl_3$).

^{1}H NMR (400 MHz, $CDCl_3$) δ 7.67 – 7.63 (m, 4H), 7.44 (t, J = 7.0 Hz, 2H), 7.34-7.32 (m, 4H), 3.90 (dt, J = 13.8 Hz, J = 4.1 Hz, 2H), 3.44 – 3.22 (m, 6H), 3.09 (dd, J = 16.2 Hz, J = 2.2 Hz, 2H), 2.45 (dd, J = 13.5 Hz, J = 2.4 Hz, 2H), 2.11 – 2.04 (m, 2H), 1.55 – 1.51 (m, 8H), 1.18 – 0.93 (m, 10H), 0.69 – 0.60 (m, 2H), 0.54 – 0.45 (m, 2H) ppm.

^{13}C NMR (101 MHz, $CDCl_3$) δ 163.6, 144.8, 136.3, 133.9, 130.5, 127.5, 126.0, 125.72, 125.65, 125.2, 124.8, 70.7, 69.3, 44.6, 41.7, 32.5, 29.4, 28.6, 27.1, 26.4, 25.8, 25.7 ppm.

HRMS (CI+) Calcd for $C_{43}H_{47}N_2O_2$ $[M+H]^+$: 623.3632, Found: 623.3638.

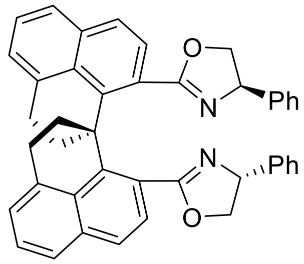

(S_s,R,R)-**L5**

(*S*)-9,9'-Bis((*R*)-4-phenyl-4,5-dihydrooxazol-2-yl)-2,2',3,3'-tetrahydro-1,1'-spirobi[phenalene] ((S_s,*R*,*R*)-L5) was prepared as a white foam according to the General Procedure A (eluent: *n*-hexane/EtOAc = 10:1) in 48% yield (294.4 mg). $[\alpha]_D^{25}$: –198.4 (*c* = 0.5, $CHCl_3$).

^{1}H NMR (400 MHz, $CDCl_3$) *δ* 7.76 – 7.73 (m, 4H), 7.58 – 7.50 (m, 4H), 7.39 (d, *J* = 6.7 Hz, 2H), 7.25 – 7.18 (m, 6H), 6.96 (d, *J* = 7.1 Hz, 4H), 3.92 (t, *J* = 13.5 Hz, 2H), 3.67 – 3.40 (m, 8H), 3.13 (d, *J* = 15.6 Hz, 2H), 2.51 (d, *J* = 13.4 Hz, 2H) ppm.

^{13}C NMR (101 MHz, $CDCl_3$) *δ* 165.5, 144.9 (2C), 136.3, 134.0, 130.5, 128.3, 127.7, 127.1, 126.6, 126.3, 125.9, 125.8, 125.2, 124.8, 73.4, 69.0, 44.7, 32.8, 27.0 ppm.

HRMS (CI+) Calcd for $C_{43}H_{35}N_2O_2$ $[M+H]^+$: 611.2693, Found: 611.2701.

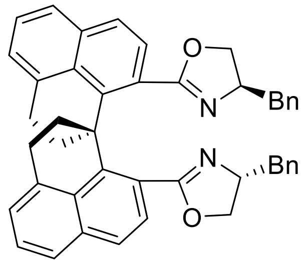

(S_s,R,R)-**L6**

(*S*)-9,9'-Bis((*R*)-4-benzyl-4,5-dihydrooxazol-2-yl)-2,2',3,3'-tetrahydro-1,1'-spirobi[phenalene] ((S_s,*R*,*R*)-L6) was prepared as a white foam from *(S)*-**4** (2.04 g, 5.0 mmol) according to the General Procedure A (eluent: *n*-hexane/EtOAc = 5:1) in 63% yield (1.4764 g). $[\alpha]_D^{25}$: –270.9 (*c* = 0.5, $CHCl_3$).

^{1}H NMR (400 MHz, $CDCl_3$) *δ* 7.71 – 7.67 (m, 4H), 7.48 (t, *J* = 7.1 Hz, 2H), 7.36 (d, *J* = 6.9 Hz, 2H), 7.31 (d, *J* = 8.4 Hz, 2H), 7.26 – 7.15 (m, 6H), 6.90 (d, *J* = 6.7 Hz, 4H), 3.78 (dt, *J* = 13.8 Hz, *J* = 4.3 Hz, 2H), 3.43 (dt, *J* = 15.7 Hz, *J* = 4.2 Hz, 2H),

3.23 (dd, J = 9.4 Hz, J = 8.3 Hz, 2H), 3.14 – 3.07 (m, 4H), 3.01 – 2.93 (m, 2H), 2.73 (dd, J = 13.9 Hz, J = 5.4 Hz, 2H), 2.48 (dd, J = 13.6 Hz, J = 2.5 Hz, 2H), 2.21 (dd, J = 13.9 Hz, J = 8.2 Hz, 2H) ppm.

^{13}C NMR (101 MHz, $CDCl_3$) δ 164.7, 144.5, 138.0, 136.1, 133.9, 130.3, 129.0, 128.1, 127.8, 126.2, 126.0, 125.9, 125.7, 125.1, 125.0, 70.5, 66.4, 44.6, 40.6, 32.5, 27.0 ppm.

HRMS (CI+) Calcd for $C_{45}H_{38}N_2O_2$ $[M+H]^+$: 639.3006, Found: 639.3014.

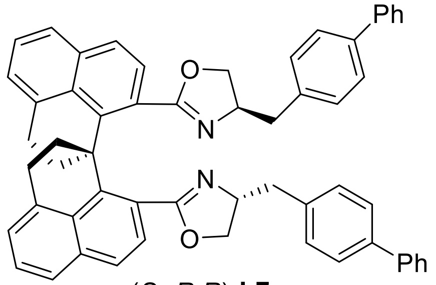

(S_s,R,R)-**L7**

(S)-9,9'-Bis((R)-4-([1,1'-biphenyl]-4-ylmethyl)-4,5-dihydrooxazol-2-yl)-2,2',3,3'-tetrahydro-1,1'-spirobi[phenalene] ((S_s,R,R)-L7) was prepared as a white foam from *(S)*-**4** (2.04 g, 5.0 mmol) according to the General Procedure A (eluent: *n*-hexane/EtOAc = 5:1) in 61% yield (2.41 g). $[\alpha]_D^{25}$: –243.4 (c = 0.5, $CHCl_3$).

^{1}H NMR (400 MHz, $CDCl_3$) δ 7.72 – 7.67 (m, 4H), 7.60 – 7.58 (m, 4H), 7.50 – 7.43 (m, 10H), 7.37 – 7.31 (m, 6H), 6.98 (d, J = 8.2 Hz, 4H), 3.80 (dt, J = 13.8 Hz, J = 4.2 Hz, 2H), 3.44 (dt, J = 15.6 Hz, J = 4.1 Hz, 2H), 3.28 (dd, J = 9.6 Hz, J = 8.2 Hz, 2H), 3.17 – 3.10 (m, 4H), 3.02 – 2.94 (m, 2H), 2.73 (dd, J = 14.0 Hz, J = 5.7 Hz, 2H), 2.45 (dd, J = 13.5 Hz, J = 2.4 Hz, 2H), 2.28 (dd, J = 13.9 Hz, J = 7.8 Hz, 2H) ppm.

^{13}C NMR (101 MHz, $CDCl_3$) δ 164.7, 144.6, 141.0, 139.0, 137.2, 136.1, 134.0, 130.4, 129.4, 128.7, 127.8, 127.0, 126.9, 126.8, 126.2, 125.9, 125.8, 125.1, 124.9, 70.6, 66.4, 44.6, 40.2, 32.5, 27.0 ppm.

HRMS (CI+) Calcd for $C_{57}H_{46}N_2NaO_2$ $[M+Na]^+$: 813.3451, Found: 813.3460.

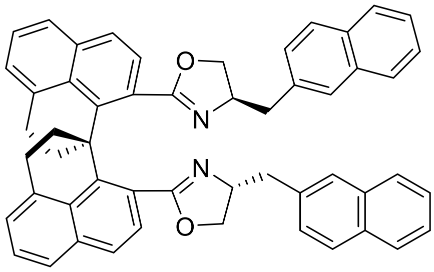

(S_s,R,R)-**L8**

(*S*)-9,9'-Bis((*R*)-4-(naphthalen-2-ylmethyl)-4,5-dihydrooxazol-2-yl)-2,2',3,3'-tetrahydro-1,1'-spirobi[phenalene] ((S_s,*R*,*R*)-L8) was prepared as a white foam from *(S)*-**4** (163.2 mg, 0.4 mmol) according to the General Procedure A (eluent: *n*-hexane/EtOAc = 5:1) in 53% yield (158.1 mg). $[\alpha]_D^{25}$: –228.5 (*c* = 0.5, $CHCl_3$).

^{1}H NMR (400 MHz, $CDCl_3$) *δ* 7.81 – 7.65 (m, 10H), 7.49 – 7.42 (m, 6H), 7.35 (s, 2H), 7.31 (d, *J* = 8.4 Hz, 2H), 7.26 (d, *J* = 6.9 Hz, 2H), 7.04 (dd, *J* = 8.4 Hz, *J* = 1.6 Hz, 2H), 3.80 (dt, *J* = 13.8 Hz, *J* = 4.2 Hz, 2H), 3.41 (dt, *J* = 15.5 Hz, *J* = 4.2 Hz, 2H), 3.26 – 3.21 (m, 2H), 3.15 – 2.97 (m, 6H), 2.86 (dd, *J* = 13.8 Hz, *J* = 5.6 Hz, 2H), 2.48 (dd, *J* = 13.6 Hz, *J* = 2.6 Hz, 2H), 2.36 (dd, *J* = 13.8 Hz, *J* = 8.0 Hz, 2H) ppm.

^{13}C NMR (101 MHz, $CDCl_3$) *δ* 164.8, 144.6, 136.1, 135.7, 134.0, 133.4, 132.1, 130.4, 127.9, 127.66, 127.65, 127.5, 127.4, 127.3, 126.2, 125.9, 125.8, 125.7, 125.3, 125.1, 124.9, 70.6, 66.6, 44.6, 40.8, 32.5, 27.0 ppm.

HRMS (CI+) Calcd for $C_{53}H_{42}N_2NaO_2$ $[M+Na]^+$: 761.3138, Found: 761.3144.

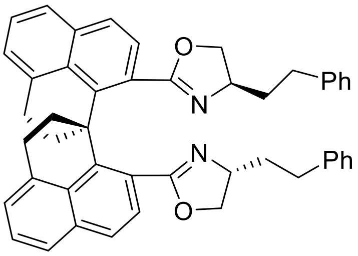

(S_s,R,R)-**L9**

(*S*)-9,9'-Bis((*R*)-4-phenethyl-4,5-dihydrooxazol-2-yl)-2,2',3,3'-tetrahydro-1,1'-spirobi[phenalene] ((S_s,*R*,*R*)-L9) was prepared as a white foam from *(S)*-**4** (163.2 mg, 0.4 mmol) according to the General Procedure A (eluent: *n*-hexane/EtOAc = 5:1) in 77% yield (205.5 mg). $[\alpha]_D^{25}$: –232.6 (*c* = 0.5, $CHCl_3$).

^{1}H NMR (400 MHz, $CDCl_3$) *δ* 7.66 – 7.63 (m, 4H), 7.45 – 7.35 (m, 6H), 7.27 – 7.23

(m, 4H), 7.19 – 7.15 (m, 2H), 7.03 – 7.01 (m, 4H), 3.83 (dt, *J* = 13.8 Hz, *J* = 4.2 Hz, 2H), 3.48 – 3.36 (m, 4H), 3.13 (dd, *J* = 16.4 Hz, *J* = 2.3 Hz, 2H), 3.04 (t, *J* = 8.9 Hz, 2H), 2.62 – 2.40 (m, 6H), 2.26 – 2.19 (m, 2H), 1.59 – 1.50 (m, 2H), 1.39 – 1.30 (m, 2H) ppm.

^{13}C NMR (101 MHz, $CDCl_3$) δ 164.2, 144.7, 141.9, 136.1, 134.0, 130.4, 128.3, 128.2, 127.7, 126.1, 125.9, 125.8, 125.6, 125.0, 124.8, 71.3, 65.3, 44.6, 36.7, 32.5, 32.2, 27.0 ppm.

HRMS (CI+) Calcd for $C_{47}H_{43}N_2O_2$ $[M+H]^+$: 667.3319, Found: 667.3329.

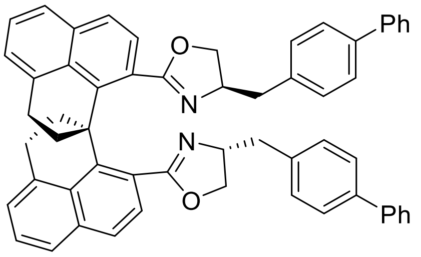

(R_s,*R*,*R*)-**L7'**

(*R*)-9,9'-Bis((*R*)-4-([1,1'-biphenyl]-4-ylmethyl)-4,5-dihydrooxazol-2-yl)-2,2',3,3'-tetrahydro-1,1'-spirobi[phenalene] ((S_s,*R*,*R*)-L7′) was prepared as a white foam from *(R)*-**4** (122.4 mg, 0.3 mmol) according to the General Procedure A (eluent: *n*-hexane/EtOAc = 5:1) in 60% yield (142.2 mg).

$[\alpha]_D^{25}$: +441.7 (*c* = 1, $CHCl_3$).

^{1}H NMR (400 MHz, $CDCl_3$) δ 7.76 (t, *J* = 9.3 Hz, 4H), 7.53 (d, *J* = 7.1 Hz, 4H), 7.49 – 7.36 (m, 14H), 7.31 (t, *J* = 7.3 Hz, 2H), 6.96 (d, *J* = 8.1 Hz, 4H), 3.93 (qd, *J* = 9.1, 5.7 Hz, 2H), 3.71 (td, *J* = 13.9, 4.1 Hz, 2H), 3.55 – 3.42 (m, 2H), 3.39 (t, *J* = 8.9 Hz, 2H), 3.16 (d, *J* = 14.5 Hz, 2H), 2.85 (t, *J* = 8.7 Hz, 2H), 2.56 – 2.43 (m, 2H), 2.08 (dd, *J* = 13.7, 5.5 Hz, 2H), 1.34 (dd, *J* = 13.7, 8.8 Hz, 2H) ppm.

^{13}C NMR (101 MHz, $CDCl_3$) δ 165.6, 143.3, 140.9, 139.0, 137.6, 136.0, 134.3, 130.4, 129.1, 128.7, 127.9, 127.0, 126.9, 126.4, 126.2, 126.1, 125.5 (2C), 71.0, 67.6, 44.7, 39.3, 32.2, 26.9 ppm.**HRMS** (CI+) Calcd for $C_{57}H_{47}N_2O_2$ $[M+H]^+$: 791.3638, Found: 791.3640.

IV. Details of Condition Optimization

Table S1. Screening of Solvents[a]

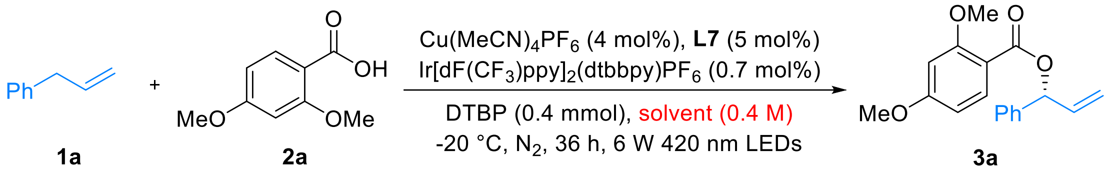

entry	solvent	yield (%)[b]	ee (%)[c]
1	DCE	80	92
2	acetone	42	94
3	DCE/acetone = 1:1	52	93
4	acetone/CF_3CH_2OH = 3:1	82	85
5	MeCN	34	38
6	$PhCF_3$	26	63
7	THF	–	–
8	DMSO	–	–
9	EtOAc	–	–
10	1,4-dioxane	–	–
11	DMF	–	–

[a]Reaction conditions: **1a** (0.3 mmol), **2a** (0.1 mmol), $Cu(MeCN)_4PF_6$ (4 μmol), **L7** (5 μmol), $Ir[dF(CF_3)ppy]_2(dtbbpy)PF_6$ (0.7 μmol), and DTBP (0.4 mmol), solvent (0.25 mL), 6 W 420 nm blue LEDs, −20 °C, 36 h. [b]Yield was determined by ^1H NMR analysis of the crude mixture with CH_2Br_2 as an internal standard. [c]Enantiomeric excess (ee) was determined by HPLC on a chiral stationary phase.

Table S2. Evaluation of Temperature[a]

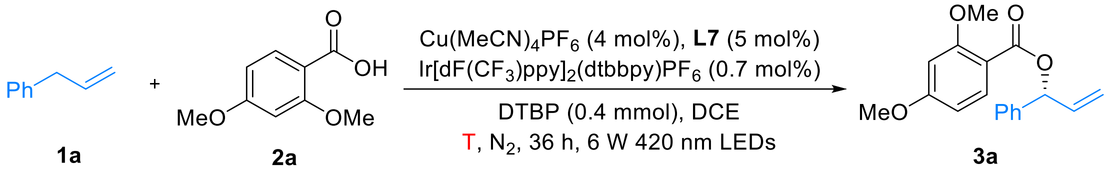

entry	T (°C)	yield (%)[b]	ee (%)[c]
1	0	77	85
2	−10	79	88
3	−20	82	92
4	−30	87	94
5	−40	84	94

[a]Standard reaction condition: **1a** (0.3 mmol), **2a** (0.1 mmol), $Cu(MeCN)_4PF_6$ (4 μmol), **L7** (5 μmol), $Ir[dF(CF_3)ppy]_2(dtbbpy)PF_6$ (0.7 μmol), and DTBP (0.4 mmol), DCE (0.25 mL), 6 W 420 nm blue LEDs, 36 h. [b]Yield was determined by ^1H NMR analysis of the crude mixture with CH_2Br_2 as an internal standard. [c]Enantiomeric excess (ee) was determined by HPLC on a chiral stationary phase.

Table S3. Screening of Chiral Ligands[a]

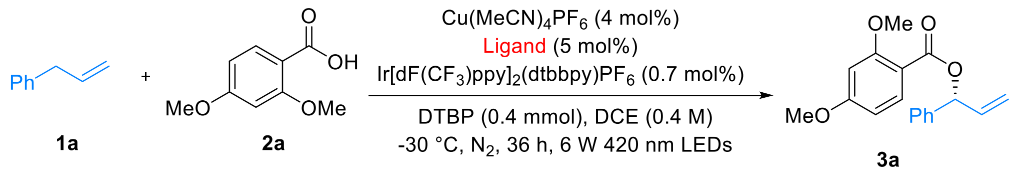

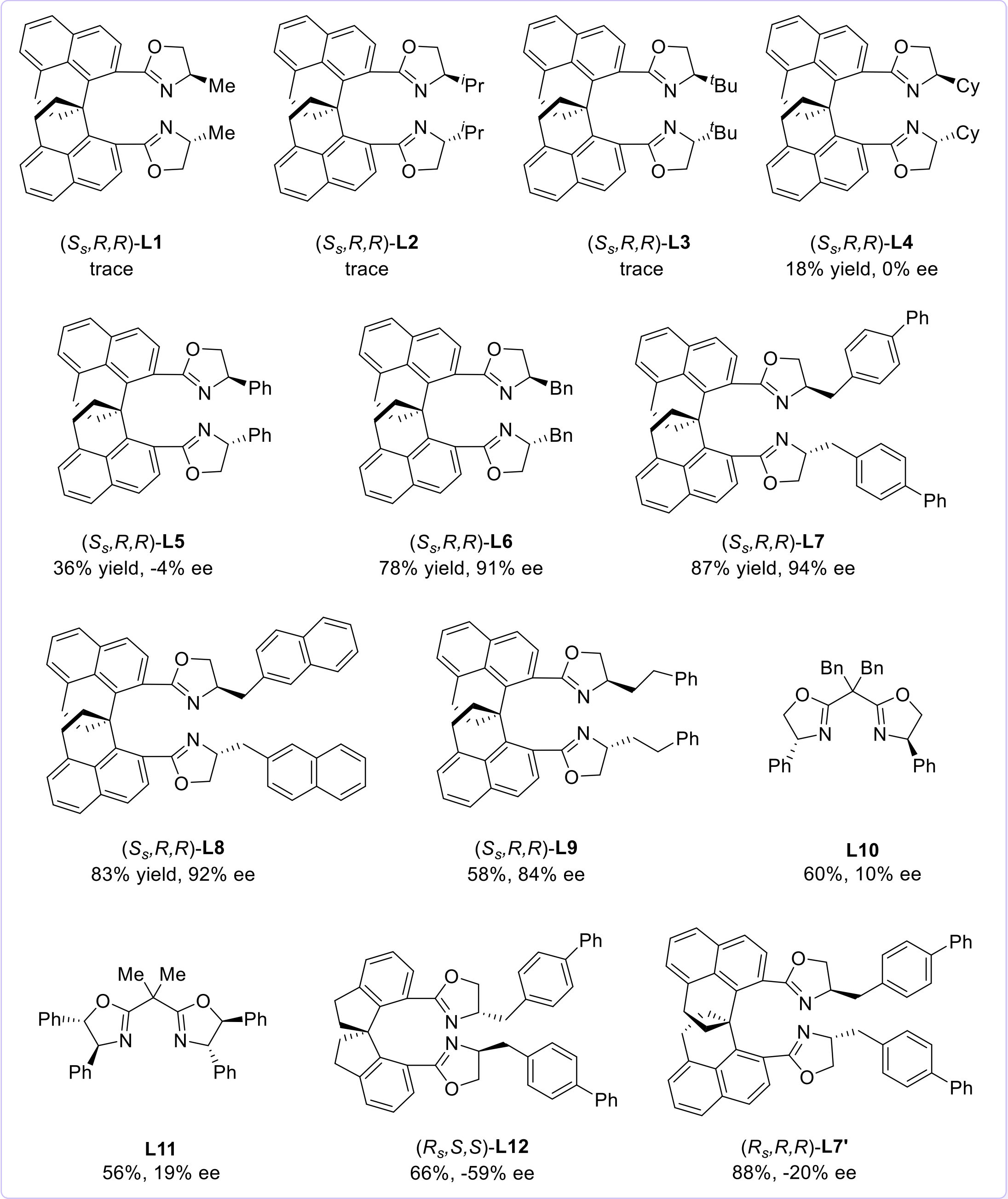

[a]Standard reaction condition: **1a** (0.3 mmol), **2a** (0.1 mmol), $Cu(MeCN)_4PF_6$ (4 μmol), Ligand (5 μmol), $Ir[dF(CF_3)ppy]_2(dtbbpy)PF_6$ (0.7 μmol), and DTBP (0.4 mmol), DCE (0.25 mL), 6 W 420 nm blue LEDs, −30 °C, 36 h. [b]Yield was determined by ^{1}H NMR analysis of the crude mixture with CH_2Br_2 as an

internal standard. [c]Enantiomeric excess (ee) was determined by HPLC on a chiral stationary phase.

Table S4. Evaluation of the Copper Sources[a]

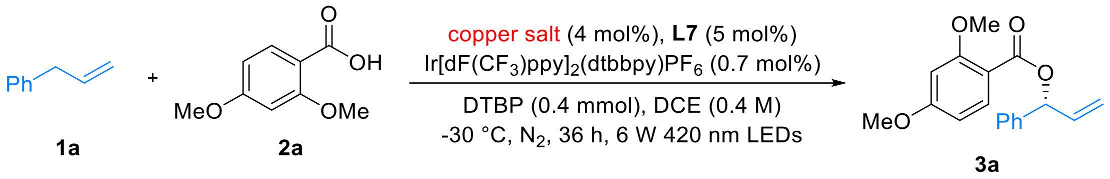

entry	Cu source (4 mol%)	yield (%)[b]	ee (%)[c]
1	$Cu(MeCN)_4PF_6$	87	94
2	$Cu(MeCN)_4BF_4$	85	93
3	CuCl	trace	–
4	CuI	trace	–
5	$Cu(OAc)_2$	trace	–

[a]Standard reaction condition: **1a** (0.3 mmol), **2a** (0.1 mmol), copper salt (4 μmol), **L7** (5 μmol), $Ir[dF(CF_3)ppy]_2(dtbbpy)PF_6$ (0.7 μmol), and DTBP (0.4 mmol), DCE (0.25 mL), 6 W 420 nm blue LEDs, −30 °C, 36 h. [b]Yield was determined by ^1H NMR analysis of the crude mixture with CH_2Br_2 as an internal standard. [c]Enantiomeric excess (ee) was determined by HPLC on a chiral stationary phase.

Table S5. Effect of Catalyst Loading[a]

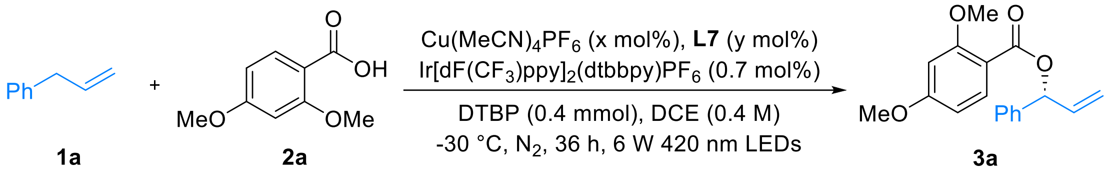

entry	x	y	yield (%)[b]	ee (%)[c]
1	4	5	87	94
2	3	3.8	76	93
3	2	2.5	70	92
4	1	1.3	50	90

[a]Standard reaction condition: **1a** (0.3 mmol), **2a** (0.1 mmol), $Cu(MeCN)_4PF_6$, **L7**, $Ir[dF(CF_3)ppy]_2(dtbbpy)PF_6$ (0.7 µmol), and DTBP (0.4 mmol), DCE (0.25 mL), 6 W 420 nm blue LEDs, −30 °C, 36 h. [b]Yield was determined by ^1H NMR analysis of the crude mixture with CH_2Br_2 as an internal standard. [c]Enantiomeric excess (ee) was determined by HPLC on a chiral stationary phase.

Table S6. Effect of Photocatalysts[a]

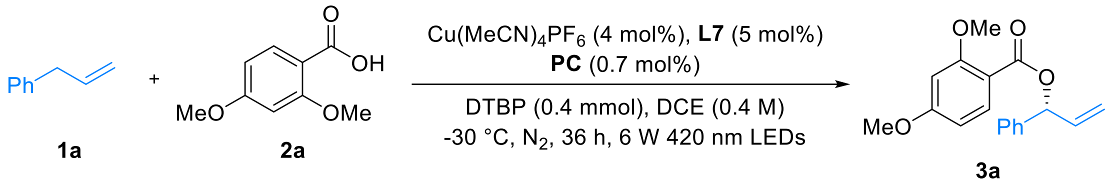

entry	**PC** (0.7 mol%)	E_T(kcal mol^{-1})	yield (%)[b]	ee (%)[c]
1	TXT	65.4	80	89
2	$Ir[dF(CF_3)ppy]_2(dtbbpy)PF_6$	61.8	87	94
3	4CzIPN	53	< 10	–
4	$Ir(ppy)_2(dtbbpy)$ PF_6	49.2	< 10	–
5	$[Ru(bpy)_3](PF_6)_2$	46.5	trace	–
6	$[Mes\text{-}Acr]^+(ClO_4)^-$	44.7	trace	–

[a]Standard reaction condition: **1a** (0.3 mmol), **2a** (0.1 mmol), $Cu(MeCN)_4PF_6$ (4 μmol), **L7** (5 μmol), **PC** (0.7 μmol), and DTBP (0.4 mmol), DCE (0.25 mL), 6 W 420 nm blue LEDs, −30 °C, 36 h. [b]Yield was determined by ^{1}H NMR analysis of the crude mixture with CH_2Br_2 as an internal standard. [c]Enantiomeric excess (ee) was determined by HPLC on a chiral stationary phase. TXT = Thiaxanthone. 4CzIPN = 1,2,3,5-Tetrakis(carbazol-9-yl)-4,6-dicyanobenzene.

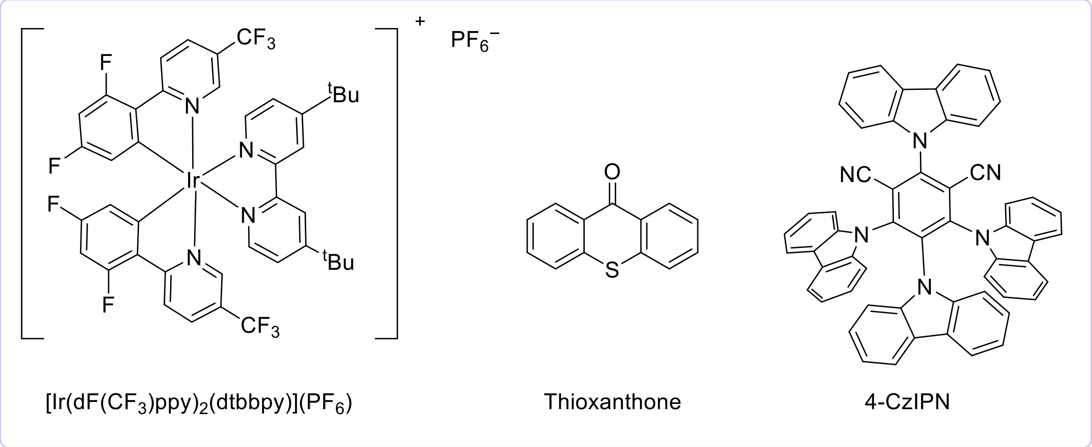

Table S7. Evaluation of the Substrate Loading[a]

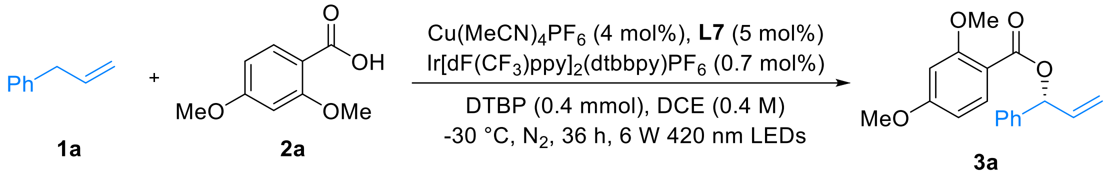

entry	**1a** (mmol)	**2a** (mmol)	DTBP (mmol)	yield (%)[b]	ee (%)[c]
1	0.3	0.1	0.4	87	94
2	0.3	0.1	0.3	86	93
3	0.2	0.1	0.3	72	90
4	0.2	0.1	0.4	66	91
5	0.1	0.15	0.4	20	81

[a]Standard reaction condition: **1a**, **2a**, $Cu(MeCN)_4PF_6$ (4 μmol), **L7** (5 μmol), $Ir[dF(CF_3)ppy]_2(dtbbpy)PF_6$ (0.7 μmol), and DTBP, DCE (0.25 mL), 6 W 420 nm blue LEDs, −30 °C, 36 h. [b]Yield was determined by ^{1}H NMR analysis of the crude mixture with CH_2Br_2 as an internal standard. [c]Enantiomeric excess (ee) was determined by HPLC on a chiral stationary phase.

Table S8. Evaluation of Reaction Concentration[a]

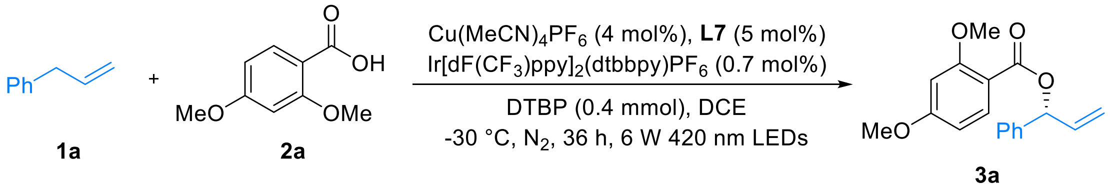

entry	conc.	yield (%)[b]	ee (%)[c]
1	0.5 M	87	94
2	0.4 M	87	94
3	0.3 M	83	94
4	0.2 M	80	94
5	0.1 M	71	94

[a]Standard reaction condition: **1a** (0.3 mmol), **2a** (0.1 mmol), $Cu(MeCN)_4PF_6$ (4 μmol), **L7** (5 μmol), $Ir[dF(CF_3)ppy]_2(dtbbpy)PF_6$ (0.7 μmol), and DTBP (0.4 mmol), DCE, 6 W 420 nm blue LEDs, −30 °C, 36 h. [b]Yield was determined by ^1H NMR analysis of the crude mixture with CH_2Br_2 as an internal standard. [c]Enantiomeric excess (ee) was determined by HPLC on a chiral stationary phase.

Table S9. Control Experiments[a]

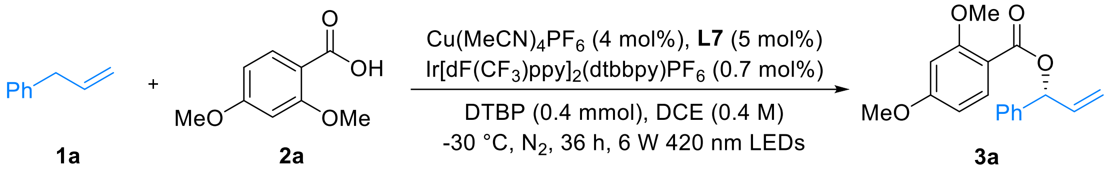

entry	Cu salt	ligand	**PC**	yield (%)[b]	ee (%)[c]
1[d]	√	√	√	ND	–
2	–	√	√	ND	–
3[e]	√	–	√	46	0
4	√	√	–	12	93
5[f]	√	√	√	ND	–

[a]Standard reaction condition: **1a** (0.3 mmol), **2a** (0.1 mmol), $Cu(MeCN)_4PF_6$ (4 μmol), **L7** (5 μmol), $Ir[dF(CF_3)ppy]_2(dtbbpy)PF_6$ (0.7 μmol), and DTBP (0.4 mmol), DCE (0.25 mL), 6 W 420 nm blue LEDs, −30 °C, 36 h. [b]Yield was determined by ^1H NMR analysis of the crude mixture with CH_2Br_2 as an internal standard. [c]Enantiomeric excess (ee) was determined by HPLC on a chiral stationary phase. [d]no DTBP. [e]b/l = 4.5:1, b = branched, l = linear. [f]no light. ND = Not detected. **PC** = $Ir[dF(CF_3)ppy]_2(dtbbpy)PF_6$.

V. Catalytic Enantioselective Allylic C(sp^3)–H Oxidation

General Procedure B

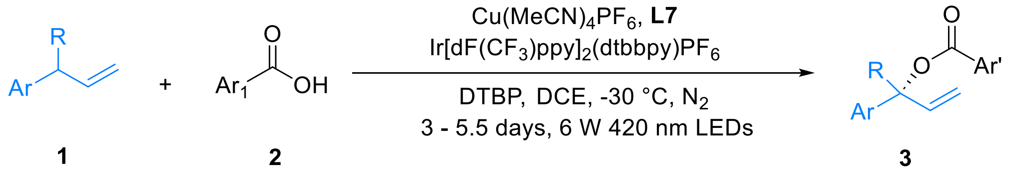

In a glove box, an oven-dried vial was charged with a stir bar, $Cu(MeCN)_4PF_6$ (4.5 mg, 0.012 mmol, 4 mol%), **L7** (11.9 mg, 0.015 mmol, 5 mol%) and DCE (0.75 mL). The mixture was stirred at 40 °C for 60 min before it was added to a separate photoreactor tube charged with the carboxylic acid **2** (0.3 mmol, 1.0 equiv) and the iridium photocatalyst (2.3 mg, 2.1 µmol, 0.7 mol%). Next, the alkene **1** (0.9 mmol, 3.0 equiv) and di-*tert*-butyl peroxide (225 µL, 1.2 mmol, 4.0 equiv) were added. The photoreactor tube was sealed with a rubber stopper and then removed from the glove box. The reaction mixture was stirred at –30 °C (internal temperature) with irradiation by 6 W 420 nm LEDs. After stirring for 3 – 5.5 days, the reaction mixture was quenched by exposure to air and warmed to room temperature. The resulting homogenous solution was transferred to a 50-mL round-bottom flask with the aid of EtOAc (2 x 3 mL). Silica was added to this solution and the volatiles were removed under reduced pressure. The residue was purified by silica gel flash chromatography to give the pure product **3**.

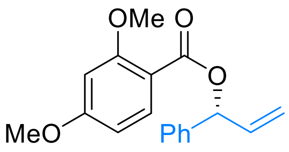

3a

(*R*)-1-Phenylallyl 2,4-dimethoxybenzoate (3a) was prepared as a colorless oil from $Cu(MeCN)_4PF_6$ (4.5 mg, 0.012 mmol, 4 mol%), **L7** (11.9 mg, 0.015 mmol, 5 mol%), $Ir[dF(CF_3)ppy]_2(dtbbpy)PF_6$ (2.3 mg, 2.1 µmol, 0.7 mol%), 2,4-

dimethoxybenzoic acid **2a** (54.6 mg, 0.3 mmol), allylbenzene **1a** (120 μL, 0.9 mmol), and DTBP (225 μL, 1.2 mmol) in DCE (0.75 mL) according to the General Procedure B (–30 °C, 3 d, eluent: 10→25% EtOAc in *n*-hexane) in 83% yield (74.2 mg, 94% ee).

$[\alpha]_D^{25}$: +3.2 (*c* = 1.0, $CHCl_3$). HPLC analysis of the product: Daicel CHIRALPAK® AD-3 column; 20% *i*-PrOH in *n*-hexane; 1.0 mL/min; retention times: 9.8 min (major), 15.6 min (minor).

^{1}H NMR (400 MHz, $CDCl_3$) δ 7.95 (d, *J* = 8.4 Hz, 1H), 7.46 (d, *J* = 7.4 Hz, 2H), 7.36 (t, *J* = 7.4 Hz, 2H), 7.29 (t, *J* = 7.2 Hz, 1H), 6.51 – 6.48 (m, 3H), 6.10 (ddd, *J* = 16.6, 10.4, 5.8 Hz, 1H), 5.49 – 5.19 (m, 2H), 3.89 (s, 3H), 3.84 (s, 3H) ppm.

^{13}C NMR (101 MHz, $CDCl_3$) δ 164.3, 164.3, 161.7, 139.4, 136.7, 133.9, 128.4, 127.9, 127.1, 116.6, 112.2, 104.5, 98.9, 75.9, 55.9, 55.4 ppm.

HRMS (ESI) Calcd for $C_{18}H_{18}NaO_4$ $[M + Na]^+$: 321.1098, found: 321.1104.

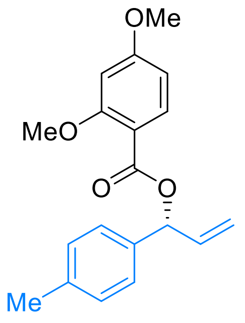

3b

(*R*)-1-(*p*-Tolyl)allyl 2,4-dimethoxybenzoate (3b) was prepared as a colorless oil from $Cu(MeCN)_4PF_6$ (4.5 mg, 0.012 mmol, 4 mol%), **L7** (11.9 mg, 0.015 mmol, 5 mol%), $Ir[dF(CF_3)ppy]_2(dtbbpy)PF_6$ (2.3 mg, 2.1 μmol, 0.7 mol%), 2,4-dimethoxybenzoic acid **2a** (54.6 mg, 0.3 mmol), alkene **1b** (118.8 mg, 0.9 mmol), and DTBP (225 μL, 1.2 mmol) in DCE (0.75 mL) according to the General Procedure B (–30 °C, 3 d, eluent: 10→25% EtOAc in *n*-hexane) in 64% yield (59.9 mg, 84% ee).

$[\alpha]_D^{25}$: +10.2 (*c* = 1.0, $CHCl_3$). HPLC analysis of the product: Daicel

CHIRALPAK® AD-3 column; 20% *i*-PrOH in *n*-hexane; 1.0 mL/min; retention times: 10.6 min (major), 15.8 min (minor).

^{1}H NMR (400 MHz, $CDCl_3$) δ 7.92 (d, *J* = 8.7 Hz, 1H), 7.34 (d, *J* = 8.0 Hz, 2H), 7.16 (d, *J* = 7.9 Hz, 2H), 6.57 – 6.40 (m, 3H), 6.08 (ddd, *J* = 16.4, 10.4, 5.7 Hz, 1H), 5.39 (d, *J* = 17.1 Hz, 1H), 5.24 (d, *J* = 10.5 Hz, 1H), 3.87 (s, 3H), 3.83 (s, 3H), 2.33 (s, 3H) ppm.

^{13}C NMR (101 MHz, $CDCl_3$) δ 164.3 (2C), 161.6, 137.6, 136.8, 136.4, 133.9, 129.1, 127.1, 116.4, 112.3, 104.5, 98.9, 75.9, 55.8, 55.4, 21.1 ppm.

HRMS (ESI) Calcd for $C_{19}H_{20}NaO_4$ $[M + Na]^+$: 335.1259, found: 335.1256.

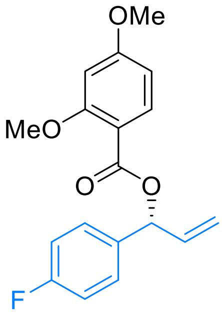

3c

(*R*)-1-(4-Fluorophenyl)allyl 2,4-dimethoxybenzoate (3c) was prepared as a colorless oil from $Cu(MeCN)_4PF_6$ (4.5 mg, 0.012 mmol, 4 mol%), **L7** (11.9 mg, 0.015 mmol, 5 mol%), $Ir[dF(CF_3)ppy]_2(dtbbpy)PF_6$ (2.3 mg, 2.1 μmol, 0.7 mol%), 2,4-dimethoxybenzoic acid **2a** (54.6 mg, 0.3 mmol), alkene **1c** (122.4 mg, 0.9 mmol), and DTBP (225 μL, 1.2 mmol) in DCE (0.75 mL) according to the General Procedure B (–30 °C, 3 d, eluent: 10→25% EtOAc in *n*-hexane) in 82% yield (77.7 mg, 96% ee).

$[\alpha]_D^{25}$: +2.5 (*c* = 1.0, $CHCl_3$). HPLC analysis of the product: Daicel CHIRALPAK® AD-3 column; 20% *i*-PrOH in *n*-hexane; 1.0 mL/min; retention times: 9.9 min (major), 15.3 min (minor).

^{1}H NMR (400 MHz, $CDCl_3$) δ 7.92 (d, *J* = 8.5 Hz, 1H), 7.42 (dd, *J* = 8.6, 5.4 Hz, 2H), 7.04 (t, *J* = 8.7 Hz, 2H), 6.54 – 6.42 (m, 3H), 6.15 – 6.00 (m, 1H), 5.39 (dt, *J* =

17.1, 1.3 Hz, 1H), 5.27 (dt, *J* = 10.5, 1.3 Hz, 1H), 3.88 (s, 3H), 3.84 (s, 3H) ppm.

^{19}F NMR (376 MHz, $CDCl_3$) δ -114.35 ppm.

^{13}C NMR (101 MHz, $CDCl_3$) δ 164.5, 164.3, 162.4 (d, *J* = 246.3 Hz), 161.7, 136.5, 135.3 (d, *J* = 3.1 Hz), 134.0, 129.0 (d, *J* = 8.2 Hz), 116.8, 115.3 (d, *J* = 21.5 Hz), 112.1, 104.6, 99.0, 75.3, 55.9, 55.5 ppm.

HRMS (ESI) Calcd for $C_{18}H_{17}FNaO_4$ $[M + Na]^+$: 339.1009, found: 339.1006.

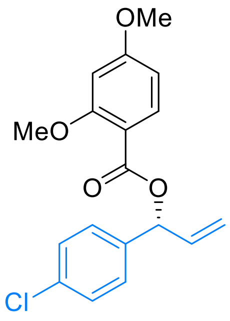

3d

(*R*)-1-(4-Chlorophenyl)allyl 2,4-dimethoxybenzoate (3d) was prepared as a colorless oil from $Cu(MeCN)_4PF_6$ (4.5 mg, 0.012 mmol, 4 mol%), **L7** (11.9 mg, 0.015 mmol, 5 mol%), $Ir[dF(CF_3)ppy]_2(dtbbpy)PF_6$ (2.3 mg, 2.1 µmol, 0.7 mol%), 2,4-dimethoxybenzoic acid **2a** (54.6 mg, 0.3 mmol), alkene **1d** (136.8 mg, 0.9 mmol), and DTBP (225 µL, 1.2 mmol) in DCE (0.75 mL) according to the General Procedure B (–30 °C, 3 d, eluent: 10→25% EtOAc in *n*-hexane) in 76% yield (75.7 mg, 96% ee).

$[\alpha]_D^{25}$: –10.5 (*c* = 1.0, $CHCl_3$). HPLC analysis of the product: Daicel CHIRALPAK® AD-3 column; 20% *i*-PrOH in *n*-hexane; 1.0 mL/min; retention times: 10.5 min (major), 16.2 min (minor).

^{1}H NMR (400 MHz, $CDCl_3$) δ 7.92 (d, *J* = 8.4 Hz, 1H), 7.39 (d, *J* = 8.4 Hz, 2H), 7.32 (d, *J* = 8.2 Hz, 2H), 6.55 – 6.39 (m, 3H), 6.13 – 5.98 (m, 1H), 5.45 – 5.35 (m, 1H), 5.32 – 5.20 (m, 1H), 3.88 (s, 3H), 3.84 (s, 3H) ppm.

^{13}C NMR (101 MHz, $CDCl_3$) δ 164.5, 164.2, 161.7, 137.9, 136.2, 133.9, 133.7, 128.6, 128.5, 117.0, 111.9, 104.6, 98.9, 75.2, 55.8, 55.4 ppm.

HRMS (ESI) Calcd for $C_{18}H_{17}ClNaO_4$ [M + Na]$^+$: 355.0713, found: 355.0711.

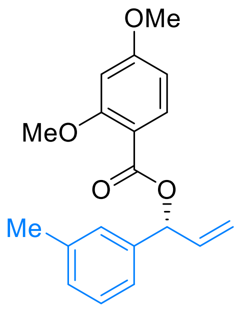

3e

(*R*)-1-(*m*-Tolyl)allyl 2,4-dimethoxybenzoate (3e) was prepared as a colorless oil from $Cu(MeCN)_4PF_6$ (4.5 mg, 0.012 mmol, 4 mol%), **L7** (11.9 mg, 0.015 mmol, 5 mol%), Ir[dF(CF_3)ppy]$_2$(dtbbpy)PF_6 (2.3 mg, 2.1 μmol, 0.7 mol%), 2,4-dimethoxybenzoic acid **2a** (54.6 mg, 0.3 mmol), alkene **1e** (118.8 mg, 0.9 mmol), and DTBP (225 μL, 1.2 mmol) in DCE (0.75 mL) according to the General Procedure B (–30 °C, 3 d, eluent: 10→20% EtOAc in *n*-hexane) in 69% yield (64.6 mg, 95% ee).

$[\alpha]_D^{25}$: +4.5 (*c* = 1.0, $CHCl_3$). HPLC analysis of the product: Daicel CHIRALPAK® AD-3 column; 20% *i*-PrOH in *n*-hexane; 1.0 mL/min; retention times: 8.1 min (major), 11.7 min (minor).

^{1}H NMR (400 MHz, $CDCl_3$) δ 7.94 (d, *J* = 8.5 Hz, 1H), 7.25 (d, *J* = 5.2 Hz, 3H), 7.10 (dd, *J* = 5.6, 2.5 Hz, 1H), 6.56 – 6.41 (m, 3H), 6.15 – 6.00 (m, 1H), 5.40 (d, *J* = 17.1 Hz, 1H), 5.25 (d, *J* = 10.4 Hz, 1H), 3.89 (s, 3H), 3.84 (s, 3H), 2.35 (s, 3H) ppm.

^{13}C NMR (101 MHz, $CDCl_3$) δ 164.3 (2C), 161.7, 139.3, 138.0, 136.7, 134.0, 128.6, 128.3, 127.8, 124.2, 116.5, 112.3, 104.5, 98.9, 76.0, 55.8, 55.4, 21.4 ppm.

HRMS (ESI) Calcd for $C_{19}H_{20}NaO_4$ [M + Na]$^+$: 335.1259, found: 335.1257.

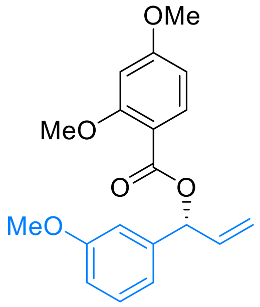

3f

(*R*)-1-(3-Methoxyphenyl)allyl 2,4-dimethoxybenzoate (3f) was prepared as a colorless oil from $Cu(MeCN)_4PF_6$ (4.5 mg, 0.012 mmol, 4 mol%), **L7** (11.9 mg, 0.015 mmol, 5 mol%), $Ir[dF(CF_3)ppy]_2(dtbbpy)PF_6$ (2.3 mg, 2.1 µmol, 0.7 mol%), 2,4-dimethoxybenzoic acid **2a** (54.6 mg, 0.3 mmol), alkene **1f** (133.2 mg, 0.9 mmol), and DTBP (225 µL, 1.2 mmol) in DCE (0.75 mL) according to the General Procedure B (–30 °C, 3 d, eluent: 10→25% EtOAc in *n*-hexane) in 62% yield (61.0 mg, 93% ee).

$[\alpha]_D^{25}$: –10.9 (c = 1.0, $CHCl_3$). HPLC analysis of the product: Daicel CHIRALPAK® AD-3 column; 20% *i*-PrOH in *n*-hexane; 1.0 mL/min; retention times: 12.2 min (major), 16.6 min (minor).

^{1}H NMR (400 MHz, $CDCl_3$) δ 7.94 (d, *J* = 8.4 Hz, 1H), 7.27 (t, *J* = 7.9 Hz, 1H), 7.07 – 6.99 (m, 2H), 6.86 – 6.80 (m, 1H), 6.53 – 6.42 (m, 3H), 6.08 (ddd, *J* = 17.1, 10.4, 5.8 Hz, 1H), 5.41 (dt, *J* = 17.1, 1.4 Hz, 1H), 5.26 (dt, *J* = 10.4, 1.3 Hz, 1H), 3.89 (s, 3H), 3.84 (s, 3H), 3.80 (s, 3H) ppm.

^{13}C NMR (101 MHz, $CDCl_3$) δ 164.3, 164.2, 161.7, 159.6, 141.0, 136.5, 133.9, 129.5, 119.4, 116.7, 113.2, 112.8, 112.1, 104.5, 98.9, 75.8, 55.8, 55.4, 55.2 ppm.

HRMS (ESI) Calcd for $C_{19}H_{20}NaO_5$ $[M + Na]^+$: 351.1208, found: 351.1211.

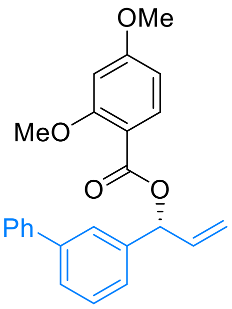

3g

(*R*)-1-([1,1'-Biphenyl]-3-yl)allyl 2,4-dimethoxybenzoate (3g) was prepared as a colorless oil from Cu(MeCN)$_4$PF$_6$ (4.5 mg, 0.012 mmol, 4 mol%), **L7** (11.9 mg, 0.015 mmol, 5 mol%), Ir[dF(CF$_3$)ppy]$_2$(dtbbpy)PF$_6$ (2.3 mg, 2.1 μmol, 0.7 mol%), 2,4-dimethoxybenzoic acid **2a** (54.6 mg, 0.3 mmol), alkene **1g** (174.6 mg, 0.9 mmol), and DTBP (225 μL, 1.2 mmol) in DCE (0.75 mL) according to the General Procedure B (–30 °C, 3 d, eluent: 10→25% EtOAc in *n*-hexane) in 84% yield (94.2 mg, 94% ee).

$[\alpha]_D^{25}$: –5.3 (*c* = 1.0, CHCl$_3$). HPLC analysis of the product: Daicel CHIRALPAK® AD-3 column; 20% *i*-PrOH in *n*-hexane; 1.0 mL/min; retention times: 11.2 min (major), 15.4 min (minor).

^{1}H NMR (400 MHz, CDCl$_3$) δ 7.96 (d, *J* = 8.5 Hz, 1H), 7.68 (s, 1H), 7.61 – 7.57 (m, 2H), 7.52 (ddd, *J* = 5.1, 3.3, 1.9 Hz, 1H), 7.47 – 7.41 (m, 4H), 7.34 (t, *J* = 7.3 Hz, 1H), 6.57 – 6.46 (m, 3H), 6.13 (ddd, *J* = 16.8, 10.4, 5.8 Hz, 1H), 5.45 (d, *J* = 17.1 Hz, 1H), 5.29 (d, *J* = 10.4 Hz, 1H), 3.88 (s, 3H), 3.84 (s, 3H) ppm.

^{13}C NMR (101 MHz, CDCl$_3$) δ 164.4 (2C), 161.7, 141.5, 141.0, 139.9, 136.6, 134.0, 128.9, 128.7, 127.3, 127.2, 126.7, 126.1, 126.0, 116.8, 112.2, 104.5, 98.9, 76.0, 55.9, 55.5 ppm.

HRMS (ESI) Calcd for $C_{24}H_{22}NaO_4$ [M + Na]$^+$: 397.1416, found: 397.1418.

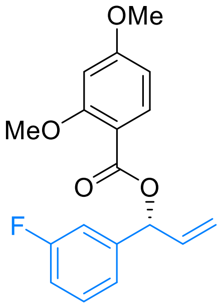

3h

(*R*)-1-(3-Fluorophenyl)allyl 2,4-dimethoxybenzoate (3h) was prepared as a colorless oil from $Cu(MeCN)_4PF_6$ (9 mg, 0.012 mmol, 8 mol%), **L7** (23.8 mg, 0.015 mmol, 9 mol%), $Ir[dF(CF_3)ppy]_2(dtbbpy)PF_6$ (2.3 mg, 2.1 μmol, 0.7 mol%), 2,4-dimethoxybenzoic acid **2a** (54.6 mg, 0.3 mmol), alkene **1h** (244.8 mg, 1.8 mmol), and DTBP (225 μL, 1.2 mmol) in DCE (0.75 mL) according to the General Procedure B (–30 °C, 5 d, eluent: 10→25% EtOAc in *n*-hexane) in 42% yield (39.8 mg, 94% ee).

$[\alpha]_D^{25}$: –8.1 (*c* = 1.0, $CHCl_3$). HPLC analysis of the product: Daicel CHIRALPAK® AD-3 column; 20% *i*-PrOH in *n*-hexane; 1.0 mL/min; retention times: 9.2 min (major), 11.4 min (minor).

^{1}H NMR (400 MHz, $CDCl_3$) δ 7.94 (d, *J* = 8.6 Hz, 1H), 7.32 (td, *J* = 8.1, 5.9 Hz, 1H), 7.24 – 7.17 (m, 2H), 7.01 – 6.95 (m, 1H), 6.53 – 6.48 (m, 2H), 6.46 (d, *J* = 5.9 Hz, 1H), 6.06 (ddd, *J* = 17.1, 10.4, 5.9 Hz, 1H), 5.42 (dt, *J* = 17.1, 1.3 Hz, 1H), 5.29 (dt, *J* = 10.4, 1.3 Hz, 1H), 3.90 (s, 3H), 3.86 (s, 3H) ppm.

^{19}F NMR (377 MHz, $CDCl_3$) δ -112.84 ppm.

^{13}C NMR (101 MHz, $CDCl_3$) δ 164.5, 164.3, 162.9 (d, *J* = 245.8 Hz), 161.7, 142.0 (d, *J* = 7.1 Hz), 136.2, 134.0, 129.9 (d, *J* = 8.2 Hz), 122.7 (d, *J* = 2.9 Hz), 117.3, 114.7 (d, *J* = 21.1 Hz), 113.9 (d, *J* = 22.3 Hz), 111.9, 104.6, 99.0, 75.3 (d, *J* = 1.8 Hz), 55.9, 55.5 ppm.

HRMS (ESI) Calcd for $C_{18}H_{17}FNaO_4$ $[M + Na]^+$: 339.1009, found: 339.1003.

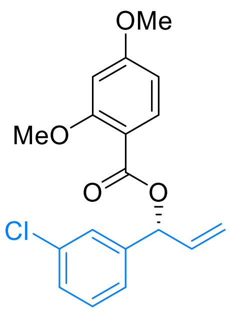

3i

(*R*)-1-(3-Chlorophenyl)allyl 2,4-dimethoxybenzoate (3i) was prepared as a colorless oil from $Cu(MeCN)_4PF_6$ (9 mg, 0.012 mmol, 8 mol%), **L7** (23.8 mg, 0.015 mmol, 9 mol%), $Ir[dF(CF_3)ppy]_2(dtbbpy)PF_6$ (2.3 mg, 2.1 μmol, 0.7 mol%), 2,4-dimethoxybenzoic acid **2a** (54.6 mg, 0.3 mmol), alkene **1i** (273.6 mg, 1.8 mmol), and DTBP (225 μL, 1.2 mmol) in DCE (0.75 mL) according to the General Procedure B (–30 °C, 5 d, eluent: 10→25% EtOAc in *n*-hexane) in 51% yield (50.8 mg, 92% ee).

$[\alpha]_D^{25}$: –4.2 (*c* = 1.0, $CHCl_3$). HPLC analysis of the product: Daicel CHIRALPAK® AD-3 column; 20% *i*-PrOH in *n*-hexane; 1.0 mL/min; retention times: 9.3 min (major), 10.8 min (minor).

^{1}H NMR (400 MHz, $CDCl_3$) δ 7.94 (d, *J* = 8.6 Hz, 1H), 7.48 (s, 1H), 7.33 – 7.26 (m, 3H), 6.54 – 6.47 (m, 2H), 6.44 (d, *J* = 5.9 Hz, 1H), 6.12 – 5.98 (m, 1H), 5.42 (d, *J* = 17.1 Hz, 1H), 5.29 (d, *J* = 10.4 Hz, 1H), 3.91 (s, 3H), 3.86 (s, 3H) ppm.

^{13}C NMR (101 MHz, $CDCl_3$) δ 164.5, 164.3, 161.7, 141.5, 136.1, 134.3, 134.0, 129.7, 128.0, 127.2, 125.3, 117.3, 111.9, 104.6, 98.9, 75.3, 55.9, 55.5 ppm.

HRMS (ESI) Calcd for $C_{18}H_{17}ClNaO_4$ $[M + Na]^+$: 355.0713, found: 355.0714.

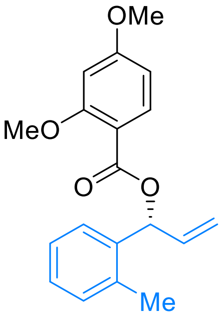

3j

(*R*)-1-(*o*-Tolyl)allyl 2,4-dimethoxybenzoate (3j) was prepared as a colorless oil from $Cu(MeCN)_4PF_6$ (4.5 mg, 0.012 mmol, 4 mol%), **L7** (11.9 mg, 0.015 mmol, 5 mol%), $Ir[dF(CF_3)ppy]_2(dtbbpy)PF_6$ (2.3 mg, 2.1 μmol, 0.7 mol%), 2,4-dimethoxybenzoic acid **2a** (54.6 mg, 0.3 mmol), alkene **1j** (118.8 mg, 0.9 mmol), and DTBP (225 μL, 1.2 mmol) in DCE (0.75 mL) according to the General Procedure B (–30 °C, 3 d, eluent: 10→20% EtOAc in *n*-hexane) in 47% yield (44.0 mg, 80% ee).

$[\alpha]_D^{25}$: –50.9 (*c* = 1.0, $CHCl_3$). HPLC analysis of the product: Daicel CHIRALPAK® AD-3 column; 20% *i*-PrOH in *n*-hexane; 1.0 mL/min; retention times: 7.9 min (major), 9.4 min (minor).

^{1}H NMR (400 MHz, $CDCl_3$) δ 7.96 (d, *J* = 8.6 Hz, 1H), 7.53 – 7.44 (m, 1H), 7.24 – 7.14 (m, 3H), 6.65 (dt, *J* = 5.4, 1.3 Hz, 1H), 6.55 – 6.44 (m, 2H), 6.08 (ddd, *J* = 17.1, 10.4, 5.5 Hz, 1H), 5.36 – 5.19 (m, 2H), 3.88 (s, 3H), 3.84 (s, 3H), 2.43 (s, 3H) ppm.

^{13}C NMR (101 MHz, $CDCl_3$) δ 164.3 (2C), 161.7, 137.5, 136.0, 135.7, 133.9, 130.4, 127.7, 127.0, 126.1, 116.6, 112.1, 104.5, 98.9, 73.3, 55.8, 55.4, 19.2 ppm.

HRMS (ESI) Calcd for $C_{19}H_{20}NaO_4$ $[M + Na]^+$: 335.1259, found: 335.1260.

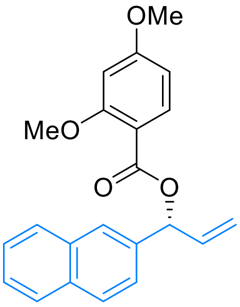

3k

(*R*)-1-(Naphthalen-2-yl)allyl 2,4-dimethoxybenzoate (3k) was prepared as a colorless oil from $Cu(MeCN)_4PF_6$ (4.5 mg, 0.012 mmol, 4 mol%), **L7** (11.9 mg, 0.015 mmol, 5 mol%), $Ir[dF(CF_3)ppy]_2(dtbbpy)PF_6$ (2.3 mg, 2.1 μmol, 0.7 mol%), 2,4-dimethoxybenzoic acid **2a** (54.6 mg, 0.3 mmol), alkene **1k** (151.2 mg, 0.9 mmol), and DTBP (225 μL, 1.2 mmol) in DCE (0.75 mL) according to the General Procedure B (–30 °C, 3 d, eluent: 10→25% EtOAc in *n*-hexane) in 51% yield (53.2 mg, 81% ee).

$[\alpha]_D^{25}$: –12.5 (*c* = 1.0, $CHCl_3$). HPLC analysis of the product: Daicel CHIRALPAK® IC-3 column; 20% *i*-PrOH in *n*-hexane; 1.0 mL/min; retention times: 10.2 min (major), 15.1 min (minor).

^{1}H NMR (400 MHz, $CDCl_3$) δ 7.96 (d, *J* = 8.7 Hz, 1H), 7.92 (s, 1H), 7.85 – 7.80 (m, 3H), 7.56 (dd, *J* = 8.5, 1.7 Hz, 1H), 7.49 – 7.45 (m, 2H), 6.65 (d, *J* = 5.6 Hz, 1H), 6.53 – 6.46 (m, 2H), 6.17 (ddd, *J* = 17.1, 10.5, 5.7 Hz, 1H), 5.46 (dt, *J* = 17.1, 1.4 Hz, 1H), 5.31 (dt, *J* = 10.5, 1.3 Hz, 1H), 3.90 (s, 3H), 3.84 (s, 3H) ppm.

^{13}C NMR (101 MHz, CDCl3) δ 164.4 (2C), 161.7, 136.7, 136.6, 134.0, 133.2, 133.0, 128.2, 128.1, 127.6, 126.3, 126.1 (2C), 125.1, 116.9, 112.2, 104.6, 98.9, 76.1, 55.9, 55.5 ppm.

HRMS (ESI) Calcd for $C_{22}H_{20}NaO_4$ $[M + Na]^+$: 371.1259, found: 371.1253.

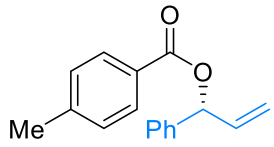

3l

(*R*)-1-Phenylallyl 4-methylbenzoate (3l) was prepared as a colorless oil from $Cu(MeCN)_4PF_6$ (4.5 mg, 0.012 mmol, 4 mol%), **L7** (11.9 mg, 0.015 mmol, 5 mol%), $Ir[dF(CF_3)ppy]_2(dtbbpy)PF_6$ (2.3 mg, 2.1 μmol, 0.7 mol%), 4-methylbenzoic acid **2b** (40.8 mg, 0.3 mmol), allylbenzene **1a** (120 μL, 0.9 mmol), and DTBP (225 μL, 1.2 mmol) in DCE (0.75 mL) according to the General Procedure B (–30 °C, 3.5 d, eluent: 0→10% EtOAc in *n*-hexane) in 67% yield (50.7 mg, 78% ee).

$[\alpha]_D^{25}$: –11.5 (c = 1.0, $CHCl_3$). HPLC analysis of the product: Daicel CHIRALPAK® AD-3 column; 1% *i*-PrOH in *n*-hexane; 1.0 mL/min; retention times: 15.9 min (major), 22.9 min (minor).

^{1}H NMR (400 MHz, $CDCl_3$) δ 7.99 (d, J = 8.2 Hz, 2H), 7.47 – 7.42 (m, 2H), 7.40 – 7.34 (m, 2H), 7.34 – 7.28 (m, 1H), 7.24 (d, J = 8.3 Hz, 2H), 6.50 (d, J = 5.8 Hz, 1H), 6.12 (ddd, J = 17.1, 10.4, 5.8 Hz, 1H), 5.39 (d, J = 17.2 Hz, 1H), 5.29 (d, J = 10.5 Hz, 1H), 2.40 (s, 3H) ppm.

^{13}C NMR (101 MHz, $CDCl_3$) δ 165.5, 143.7, 139.0, 136.4, 129.7, 129.1, 128.5, 128.1, 127.5, 127.1, 116.9, 76.4, 21.7 ppm.

HRMS (ESI) Calcd for $C_{17}H_{16}NaO_2$ $[M + Na]^+$: 275.1048, found: 275.1043.

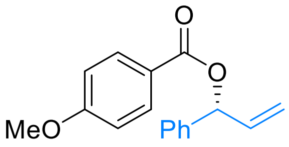

3m

(*R*)-1-Phenylallyl 4-methoxybenzoate (3m) was prepared as a colorless oil from $Cu(MeCN)_4PF_6$ (4.5 mg, 0.012 mmol, 4 mol%), **L7** (11.9 mg, 0.015 mmol, 5 mol%), $Ir[dF(CF_3)ppy]_2(dtbbpy)PF_6$ (2.3 mg, 2.1 μmol, 0.7 mol%), 4-methoxybenzoic acid **2c** (45.6 mg, 0.3 mmol), allylbenzene **1a** (120 μL, 0.9 mmol), and DTBP (225 μL, 1.2 mmol) in DCE (0.75 mL) according to the General

Procedure B (–30 °C, 4 d, eluent: 0→15% EtOAc in *n*-hexane) in 51% yield (41.0 mg, 90% ee).

$[\alpha]_D^{25}$: –24.2 (*c* = 1.0, $CHCl_3$). HPLC analysis of the product: Daicel CHIRALPAK® AD-3 column; 2% *i*-PrOH in *n*-hexane; 1.0 mL/min; retention times: 21.3 min (major), 28.7 min (minor).

^{1}H NMR (400 MHz, $CDCl_3$) δ 8.06 (d, *J* = 9.0 Hz, 2H), 7.47 – 7.42 (m, 2H), 7.40 – 7.34 (m, 2H), 7.33 – 7.28 (m, 1H), 6.96 – 6.89 (m, 2H), 6.49 (d, *J* = 5.8 Hz, 1H), 6.12 (ddd, *J* = 17.1, 10.4, 5.8 Hz, 1H), 5.38 (dt, *J* = 17.2, 1.3 Hz, 1H), 5.28 (dt, *J* = 10.4, 1.3 Hz, 1H), 3.85 (s, 3H) ppm.

^{13}C NMR (101 MHz, $CDCl_3$) δ 165.2, 163.4, 139.1, 136.5, 131.7, 128.5, 128.1, 127.1, 122.6, 116.8, 113.6, 76.3, 55.4 ppm.

HRMS (ESI) Calcd for $C_{17}H_{16}NaO_3$ $[M + Na]^+$: 291.0997, found: 291.0992.

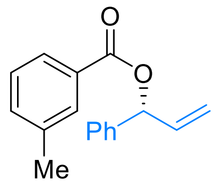

3n

(*R*)-1-Phenylallyl 3-methylbenzoate (3n) was prepared as a colorless oil from $Cu(MeCN)_4PF_6$ (4.5 mg, 0.012 mmol, 4 mol%), **L7** (11.9 mg, 0.015 mmol, 5 mol%), $Ir[dF(CF_3)ppy]_2(dtbbpy)PF_6$ (2.3 mg, 2.1 μmol, 0.7 mol%), 3-methylbenzoic acid **2d** (40.8 mg, 0.3 mmol), allylbenzene **1a** (120 μL, 0.9 mmol), and DTBP (225 μL, 1.2 mmol) in DCE (0.75 mL) according to the General Procedure B (–30 °C, 3.5 d, eluent: 0→10% EtOAc in *n*-hexane) in 67% yield (50.7 mg, 71% ee).

$[\alpha]_D^{25}$: –5.6 (*c* = 1.0, $CHCl_3$). HPLC analysis of the product: Daicel CHIRALPAK® AD-3 column; 5% *i*-PrOH in *n*-hexane; 1.0 mL/min; retention times: 5.5 min (major), 6.4 min (minor).

^{1}H NMR (400 MHz, $CDCl_3$) δ 7.95 – 7.86 (m, 2H), 7.48 – 7.43 (m, 2H), 7.40 – 7.29 (m, 5H), 6.51 (d, *J* = 5.8 Hz, 1H), 6.13 (ddd, *J* = 17.0, 10.4, 5.8 Hz, 1H), 5.39 (d, *J* = 17.2 Hz, 1H), 5.30 (d, *J* = 10.4 Hz, 1H), 2.40 (s, 3H) ppm.

^{13}C NMR (101 MHz, $CDCl_3$) δ 165.6, 139.0, 138.2, 136.3, 133.8, 130.2 (2C), 128.6, 128.3, 128.1, 127.1, 126.8, 117.0, 76.5, 21.3 ppm.

HRMS (ESI) Calcd for $C_{17}H_{16}NaO_2$ $[M + Na]^+$: 275.1048, found: 275.1050.

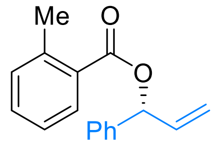

3o

(*R*)-1-Phenylallyl 2-methylbenzoate (3o) was prepared as a colorless oil from $Cu(MeCN)_4PF_6$ (4.5 mg, 0.012 mmol, 4 mol%), **L7** (11.9 mg, 0.015 mmol, 5 mol%), $Ir[dF(CF_3)ppy]_2(dtbbpy)PF_6$ (2.3 mg, 2.1 μmol, 0.7 mol%), 2-methylbenzoic acid **2e** (40.8 mg, 0.3 mmol), allylbenzene **1a** (120 μL, 0.9 mmol), and DTBP (225 μL, 1.2 mmol) in DCE (0.75 mL) according to the General Procedure B (–30 °C, 3.5 d, eluent: 0→10% EtOAc in *n*-hexane) in 85% yield (64.3 mg, 90% ee).

$[\alpha]_D^{25}$: +12.9 (*c* = 1.0, $CHCl_3$). HPLC analysis of the product: Daicel CHIRALPAK® AD-3 column; 1% *i*-PrOH in *n*-hexane; 1.0 mL/min; retention times: 5.6 min (major), 7.7 min (minor).

^{1}H NMR (400 MHz, $CDCl_3$) δ 8.00 (d, *J* = 8.0 Hz, 1H), 7.47 – 7.42 (m, 2H), 7.38 (td, *J* = 7.3, 6.1 Hz, 3H), 7.33 – 7.28 (m, 1H), 7.24 (t, *J* = 7.9 Hz, 2H), 6.49 (d, *J* = 5.9 Hz, 1H), 6.13 (ddd, *J* = 17.1, 10.4, 5.9 Hz, 1H), 5.38 (d, *J* = 17.2 Hz, 1H), 5.29 (d, *J* = 10.4 Hz, 1H), 2.59 (s, 3H) ppm.

^{13}C NMR (101 MHz, $CDCl_3$) δ 166.3, 140.4, 139.0, 136.4, 132.0, 131.7, 130.6, 129.5, 128.6, 128.1, 127.1, 125.7, 117.0, 76.6, 21.8 ppm.

HRMS (ESI) Calcd for $C_{17}H_{16}NaO_2$ $[M + Na]^+$: 275.1048, found: 275.1054.

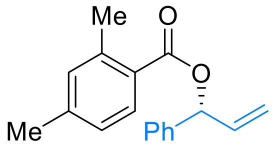

3p

(*R*)-1-Phenylallyl 2,4-dimethylbenzoate (3p) was prepared as a colorless oil

from $Cu(MeCN)_4PF_6$ (4.5 mg, 0.012 mmol, 4 mol%), **L7** (11.9 mg, 0.015 mmol, 5 mol%), $Ir[dF(CF_3)ppy]_2(dtbbpy)PF_6$ (2.3 mg, 2.1 μmol, 0.7 mol%), 2-methylbenzoic acid **2f** (45.0 mg, 0.3 mmol), allylbenzene **1a** (120 μL, 0.9 mmol), and DTBP (225 μL, 1.2 mmol) in DCE (0.75 mL) according to the General Procedure B (–30 °C, 3.5 d, eluent: 10→25% EtOAc in *n*-hexane) in 63% yield (50.3 mg, 92% ee).

$[\alpha]_D^{25}$: +1.1 (*c* = 1.0, $CHCl_3$). HPLC analysis of the product: Daicel CHIRALPAK® AD-3 column; 5% *i*-PrOH in *n*-hexane; 1.0 mL/min; retention times: 4.8 min (major), 6.3 min (minor).

^{1}H NMR (400 MHz, $CDCl_3$) δ 7.93 (d, *J* = 8.4 Hz, 1H), 7.47 – 7.42 (m, 2H), 7.39 – 7.34 (m, 2H), 7.32 – 7.28 (m, 1H), 7.05 (d, *J* = 5.7 Hz, 2H), 6.48 (d, *J* = 5.8 Hz, 1H), 6.12 (ddd, *J* = 17.1, 10.4, 5.9 Hz, 1H), 5.37 (dt, *J* = 17.1, 1.3 Hz, 1H), 5.28 (dt, *J* = 10.4, 1.3 Hz, 1H), 2.57 (s, 3H), 2.34 (s, 3H) ppm.

^{13}C NMR (101 MHz, $CDCl_3$) δ 166.3, 142.6, 140.6, 139.2, 136.6, 132.5, 130.9, 128.5, 128.0, 127.1, 126.6, 126.4, 116.8, 76.4, 21.9, 21.4 ppm.

HRMS (ESI) Calcd for $C_{18}H_{18}NaO_2$ $[M + Na]^+$: 2889.1204, found: 289.1202.

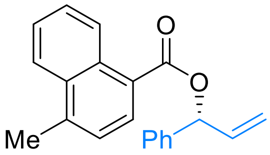

3q

(*R*)-1-Phenylallyl 4-methyl-1-naphthoate (3q) was prepared as a colorless oil from $Cu(MeCN)_4PF_6$ (4.5 mg, 0.012 mmol, 4 mol%), **L7** (11.9 mg, 0.015 mmol, 5 mol%), $Ir[dF(CF_3)ppy]_2(dtbbpy)PF_6$ (2.3 mg, 2.1 μmol, 0.7 mol%), 2-methylbenzoic acid **2g** (55.8 mg, 0.3 mmol), allylbenzene **1a** (120 μL, 0.9 mmol), and DTBP (225 μL, 1.2 mmol) in DCE (0.75 mL) according to the General Procedure B (–30 °C, 3.5 d, eluent: 0→10% EtOAc in *n*-hexane) in 45% yield (40.8 mg, 89% ee).

$[\alpha]_D^{25}$: +38.7 (*c* = 1.0, $CHCl_3$). HPLC analysis of the product: Daicel

CHIRALPAK® AD-3 column; 1% *i*-PrOH in *n*-hexane; 1.0 mL/min; retention times: 9.2 min (major), 11.8 min (minor).

^{1}H NMR (400 MHz, $CDCl_3$) δ 8.99 (dd, *J* = 7.9, 1.8 Hz, 1H), 8.18 (d, *J* = 7.4 Hz, 1H), 8.03 (dd, *J* = 7.6, 1.9 Hz, 1H), 7.57 (dddd, *J* = 14.7, 8.2, 6.8, 1.5 Hz, 2H), 7.52 – 7.46 (m, 2H), 7.42 – 7.29 (m, 4H), 6.60 (d, *J* = 5.9 Hz, 1H), 6.18 (ddd, *J* = 17.0, 10.4, 5.9 Hz, 1H), 5.44 (dt, *J* = 17.2, 1.3 Hz, 1H), 5.32 (dt, *J* = 10.5, 1.2 Hz, 1H), 2.73 (s, 3H) ppm.

^{13}C NMR (101 MHz, $CDCl_3$) δ 166.4, 140.5, 139.1, 136.5, 132.8, 131.5, 130.1, 128.6, 128.1, 127.3, 127.2, 126.3, 126.0, 125.5, 125.3, 124.4, 117.1, 76.6, 20.1 ppm.

HRMS (ESI) Calcd for $C_{21}H_{18}NaO_3$ $[M + Na]^+$: 325.1204, found: 325.1200.

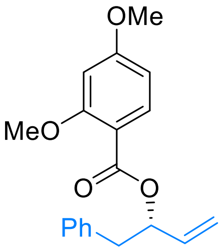

3r

(*S*)-1-Phenylbut-3-en-2-yl 2,4-dimethoxybenzoate (3r) was prepared as a colorless oil from $Cu(MeCN)_4PF_6$ (4.5 mg, 0.012 mmol, 4 mol%), **L7** (11.9 mg, 0.015 mmol, 5 mol%), $Ir[dF(CF_3)ppy]_2(dtbbpy)PF_6$ (2.3 mg, 2.1 μmol, 0.7 mol%), 2,4-dimethoxybenzoic acid **2a** (54.6 mg, 0.3 mmol), alkene **1p** (198.0 mg, 1.5 mmol), and DTBP (225 μL, 1.2 mmol) in DCE (0.75 mL) according to the General Procedure B (–30 °C, 3 d, eluent: 10→25% EtOAc in *n*-hexane) in 62% yield (58.0 mg, 49% ee).

$[\alpha]_D^{25}$: +1.6 (*c* = 1.0, $CHCl_3$). HPLC analysis of the product: Daicel CHIRALPAK® ID-3 column; 10% *i*-PrOH in *n*-hexane; 1.0 mL/min; retention times: 19.3 min (minor), 23.7 min (major).

^{1}H NMR (400 MHz, $CDCl_3$) δ 7.82 (d, *J* = 9.3 Hz, 1H), 7.27 (d, *J* = 5.3 Hz, 4H), 7.22 – 7.18 (m, 1H), 6.47 (dd, *J* = 6.4, 2.4 Hz, 2H), 5.89 (ddd, *J* = 17.0, 10.6, 6.0 Hz,

1H), 5.68 (q, J = 6.5 Hz, 1H), 5.34 – 5.23 (m, 1H), 5.16 (d, J = 10.6 Hz, 1H), 3.86 (s, 3H), 3.83 (s, 3H), 3.11 (dd, J = 13.7, 6.8 Hz, 1H), 2.98 (dd, J = 13.7, 6.5 Hz, 1H) ppm.

^{13}C NMR (101 MHz, $CDCl_3$) δ 164.5, 164.3, 161.6, 137.2, 136.0, 133.7, 129.7, 128.2, 126.5, 116.7, 112.4, 104.5, 99.0, 74.9, 55.9, 55.5, 41.0 ppm.

HRMS (ESI) Calcd for $C_{19}H_{20}NaO_4$ $[M + Na]^+$: 335.1259, found: 335.1255.

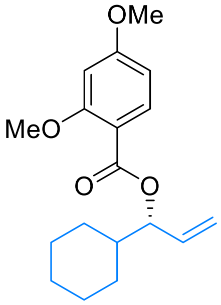

3s

(*R*)-1-Cyclohexylallyl 2,4-dimethoxybenzoate (3s) was prepared as a colorless oil from $Cu(MeCN)_4PF_6$ (4.5 mg, 0.012 mmol, 4 mol%), **L7** (11.9 mg, 0.015 mmol, 5 mol%), $Ir[dF(CF_3)ppy]_2(dtbbpy)PF_6$ (2.3 mg, 2.1 μmol, 0.7 mol%), 2,4-dimethoxybenzoic acid **2a** (54.6 mg, 0.3 mmol), alkene **1q** (186.0 mg, 1.5 mmol), and DTBP (225 μL, 1.2 mmol) in DCE (0.75 mL) according to the General Procedure B (–30 °C, 3 d, eluent: 10→25% EtOAc in *n*-hexane) in 40% yield (39.2 mg, 45% ee).

$[\alpha]_D^{25}$: +9.0 (c = 1.0, $CHCl_3$). HPLC analysis of the product: Daicel CHIRALPAK® IC-3 column; 10% *i*-PrOH in *n*-hexane; 1.0 mL/min; retention times: 10.4 min (major), 16.6 min (minor).

^{1}H NMR (400 MHz, $CDCl_3$) δ 7.89 (d, J = 8.4 Hz, 1H), 6.52 – 6.47 (m, 2H), 5.85 (ddd, J = 17.3, 10.5, 6.4 Hz, 1H), 5.36 – 5.26 (m, 2H), 5.20 (d, J = 10.6 Hz, 1H), 3.88 (s, 3H), 3.85 (s, 3H), 1.84 (d, J = 12.9 Hz, 1H), 1.78 – 1.74 (m, 2H), 1.67 (d, J = 10.0 Hz, 3H), 1.26 – 1.06 (m, 5H) ppm.

^{13}C NMR (101 MHz, $CDCl_3$) δ 164.8, 164.1, 161.5, 135.4, 133.7, 117.1, 112.8, 104.5, 99.0, 78.6, 55.9, 55.5, 41.8, 28.8, 28.3, 26.5, 26.1 ppm.

HRMS (ESI) Calcd for $C_{18}H_{24}NaO_4$ $[M + Na]^+$: 327.1572, found: 327.1568.

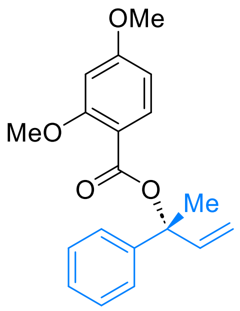

3t

(*R*)-2-Phenylbut-3-en-2-yl 2,4-dimethoxybenzoate (3t) was prepared as a colorless oil from $Cu(MeCN)_4PF_6$ (4.5 mg, 0.012 mmol, 4 mol%), **L7** (11.9 mg, 0.015 mmol, 5 mol%), $Ir[dF(CF_3)ppy]_2(dtbbpy)PF_6$ (2.3 mg, 2.1 μmol, 0.7 mol%), 2,4-dimethoxybenzoic acid **2a** (54.6 mg, 0.3 mmol), alkene **1l** (118.8 mg, 0.9 mmol), and DTBP (225 μL, 1.2 mmol) in DCE (0.75 mL) according to the General Procedure B (–30 °C, 5 d, eluent: 10→25% EtOAc in *n*-hexane) in 50% yield (46.8 mg, 92% ee).

$[\alpha]_D^{25}$: +3.7 (*c* = 1.0, $CHCl_3$). HPLC analysis of the product: Daicel CHIRALPAK® IC-3 column; 20% *i*-PrOH in *n*-hexane; 1.0 mL/min; retention times: 9.3 min (minor), 10.1 min (major).

^{1}H NMR (400 MHz, $CDCl_3$) δ 7.94 (d, *J* = 8.6 Hz, 1H), 7.52 – 7.44 (m, 2H), 7.33 (t, *J* = 7.6 Hz, 2H), 7.26 – 7.22 (m, 1H), 6.56 – 6.44 (m, 2H), 6.33 (dd, *J* = 17.4, 10.8 Hz, 1H), 5.40 (dd, *J* = 17.4, 0.9 Hz, 1H), 5.25 (dd, *J* = 10.8, 0.9 Hz, 1H), 3.88 (s, 3H), 3.85 (s, 3H), 2.02 (s, 3H) ppm.

^{13}C NMR (101 MHz, $CDCl_3$) δ 164.1, 163.9, 161.5, 144.2, 142.1, 134.0, 128.1, 127.0, 125.3, 113.9, 113.3, 104.4, 98.9, 83.3, 55.8, 55.4, 25.6 ppm.

HRMS (ESI) Calcd for $C_{19}H_{20}NaO_4$ $[M + Na]^+$: 335.1259, found: 335.1254.

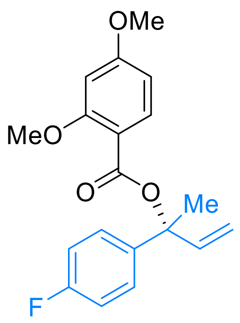

3u

(*R*)-2-(4-Fluorophenyl)but-3-en-2-yl 2,4-dimethoxybenzoate (3u) was prepared as a colorless oil from $Cu(MeCN)_4PF_6$ (4.5 mg, 0.012 mmol, 4 mol%), **L7** (11.9 mg, 0.015 mmol, 5 mol%), $Ir[dF(CF_3)ppy]_2(dtbbpy)PF_6$ (2.3 mg, 2.1 μmol, 0.7 mol%), 2,4-dimethoxybenzoic acid **2a** (54.6 mg, 0.3 mmol), alkene **1m** (135.0 mg, 0.9 mmol), and DTBP (225 μL, 1.2 mmol) in DCE (0.75 mL) according to the General Procedure B (–30 °C, 5 d, eluent: 10→25% EtOAc in *n*-hexane) in 63% yield (62.4 mg, 96% ee).

$[\alpha]_D^{25}$: +2.2 (*c* = 1.0, $CHCl_3$). HPLC analysis of the product: Daicel CHIRALPAK® AD-3 column; 20% *i*-PrOH in *n*-hexane; 1.0 mL/min; retention times: 8.9 min (major), 15.5 min (minor).

^{1}H NMR (400 MHz, $CDCl_3$) δ 7.92 (d, *J* = 8.6 Hz, 1H), 7.52 – 7.40 (m, 2H), 7.05 – 6.97 (m, 2H), 6.55 – 6.42 (m, 2H), 6.30 (dd, *J* = 17.4, 10.8 Hz, 1H), 5.39 (dd, *J* = 17.4, 0.9 Hz, 1H), 5.26 (dd, *J* = 10.8, 0.9 Hz, 1H), 3.88 (s, 3H), 3.85 (s, 3H), 2.00 (s, 3H) ppm.

^{19}F NMR (376 MHz, $CDCl_3$) δ -116.09 ppm.

^{13}C NMR (101 MHz, $CDCl_3$) δ 164.2, 163.9, 161.8 (d, *J* = 245.4 Hz), 161.5, 142.0, 139.9 (d, *J* = 3.2 Hz), 134.0, 127.2 (d, *J* = 8.1 Hz), 114.9 (d, *J* = 21.4 Hz), 114.0, 113.1, 104.5, 98.9, 82.9, 55.8, 55.5, 25.6 ppm.

HRMS (ESI) Calcd for $C_{19}H_{19}FNaO_4$ $[M + Na]^+$: 353.1165, found: 353.1158.

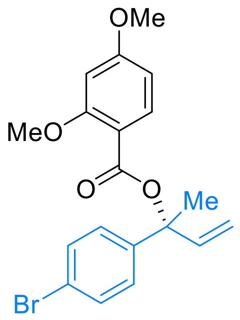

3v

(*R*)-2-(4-Bromophenyl)but-3-en-2-yl 2,4-dimethoxybenzoate (3v) was prepared as a colorless oil from $Cu(MeCN)_4PF_6$ (4.5 mg, 0.012 mmol, 4 mol%), **L7** (11.9 mg, 0.015 mmol, 5 mol%), $Ir[dF(CF_3)ppy]_2(dtbbpy)PF_6$ (2.3 mg, 2.1 μmol, 0.7 mol%), 2,4-dimethoxybenzoic acid **2a** (54.6 mg, 0.3 mmol), alkene **1n** (189.0 mg, 0.9 mmol), and DTBP (225 μL, 1.2 mmol) in DCE (0.75 mL) according to the General Procedure B (–30 °C, 5 d, eluent: 10→25% EtOAc in *n*-hexane) in 54% yield (63.2 mg, 96% ee).

$[\alpha]_D^{25}$: –3.6 (*c* = 1.0, $CHCl_3$). HPLC analysis of the product: Daicel CHIRALPAK® IC-3 column; 20% *i*-PrOH in *n*-hexane; 1.0 mL/min; retention times: 9.0 min (minor), 9.7 min (major).

^{1}H NMR (400 MHz, $CDCl_3$) δ 7.91 (d, *J* = 8.6 Hz, 1H), 7.48 – 7.42 (m, 2H), 7.38 – 7.32 (m, 2H), 6.56 – 6.43 (m, 2H), 6.29 (dd, *J* = 17.4, 10.8 Hz, 1H), 5.40 (dd, *J* = 17.4, 0.9 Hz, 1H), 5.26 (dd, *J* = 10.8, 0.9 Hz, 1H), 3.88 (s, 3H), 3.85 (s, 3H), 1.98 (s, 3H) ppm.

^{13}C NMR (101 MHz, $CDCl_3$) δ 164.3, 163.8, 161.5, 143.3, 141.6, 134.0, 131.3, 127.3, 121.0, 114.3, 113.0, 104.5, 98.9, 82.8, 55.8, 55.5, 25.5 ppm.

HRMS (ESI) Calcd for $C_{19}H_{19}BrNaO_4$ $[M + Na]^+$: 413.0364, found: 413.0366.

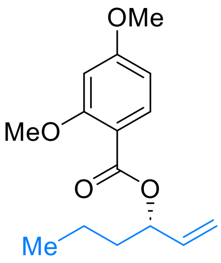

3w

(*S*)-Hex-1-en-3-yl 2,4-dimethoxybenzoate (3w) was prepared as a colorless oil from Cu(MeCN)$_4$PF$_6$ (4.5 mg, 0.012 mmol, 4 mol%), **L7** (11.9 mg, 0.015 mmol, 5 mol%), Ir[dF(CF$_3$)ppy]$_2$(dtbbpy)PF$_6$ (2.3 mg, 2.1 μmol, 0.7 mol%), 2,4-dimethoxybenzoic acid **2a** (54.6 mg, 0.3 mmol), alkene **1o** (126.0 mg, 1.5 mmol), and DTBP (225 μL, 1.2 mmol) in DCE (0.75 mL) according to the General Procedure B (–30 °C, 3 d, eluent: 10→25% EtOAc in *n*-hexane) in 68% yield (53.9 mg, 19% ee).

$[\alpha]_D^{25}$: +4.9 (*c* = 1.0, CHCl$_3$). HPLC analysis of the product: Daicel CHIRALPAK® IC-3 column; 10% *i*-PrOH in *n*-hexane; 1.0 mL/min; retention times: 10.7 min (major), 14.4 min (minor).

^{1}H NMR (400 MHz, CDCl$_3$) δ 7.91 – 7.83 (m, 1H), 6.53 – 6.45 (m, 2H), 5.88 (ddd, *J* = 16.9, 10.5, 6.1 Hz, 1H), 5.47 (q, *J* = 6.0 Hz, 1H), 5.32 (dt, *J* = 17.2, 1.4 Hz, 1H), 5.17 (dt, *J* = 10.5, 1.3 Hz, 1H), 3.88 (s, 3H), 3.84 (s, 3H), 1.77 – 1.63 (m, 2H), 1.49 – 1.37 (m, 2H), 0.94 (t, *J* = 7.4 Hz, 3H) ppm.

^{13}C NMR (101 MHz, CDCl$_3$) δ 164.8, 164.2, 161.5, 137.0, 133.6, 116.2, 112.7, 104.5, 99.0, 74.3, 55.9, 55.5, 36.5, 18.4, 13.9 ppm.

HRMS (ESI) Calcd for $C_{15}H_{20}NaO_4$ $[M + Na]^+$: 287.1259, found: 2287.1263.

VI. Mechanistic Study

(1) Radical trapping experiment with TEMPO

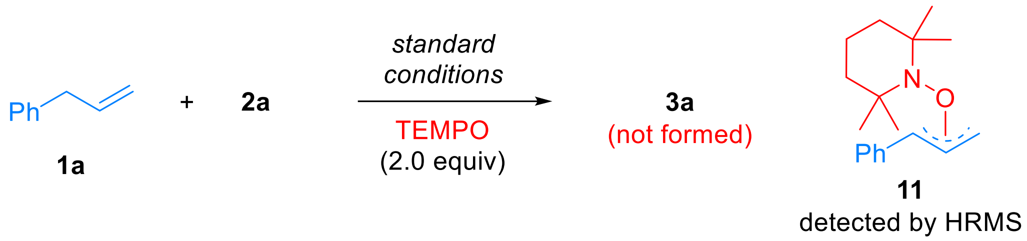

In a glove box, an oven-dried vial was charged with a stir bar, $Cu(MeCN)_4PF_6$ (1.5 mg, 4 μmol, 4 mol%), **L7** (4 mg, 5 μmol, 5 mol%) and DCE (0.25 mL). The mixture was stirred at 40 °C for 60 min before it was added to a separate photoreactor tube charged with the carboxylic acid **2a** (0.1 mmol, 1.0 equiv), TEMPO (31.2 mg, 0.2 mmol, 2.0 equiv) and the iridium photocatalyst (0.7 mg, 0.7 μmol, 0.7 mol%). Next, the alkene **1a** (0.3 mmol, 3.0 equiv) and di-*tert*-butyl peroxide (75 μL, 0.4 mmol, 4.0 equiv) were added. The photoreactor tube was sealed with a rubber stopper and then removed from the glove box. The reaction mixture was stirred at –30 °C (internal temperature) with irradiation by 6 W 420 nm LEDs. After stirring for 36 h, the reaction mixture was quenched by exposure to air and warmed to room temperature. The reaction mixture was filtered through a short pad of silica. The filtrate was directly analyzed by HRMS, which indicated the presence of a compound consistent with **11**.

HRMS (ESI) Calcd for $C_{18}H_{28}NO$ $[M + H]^+$: 274.2171, found: 274.2169.

(2) Radical trapping experiment with 1,1-diphenylethylene

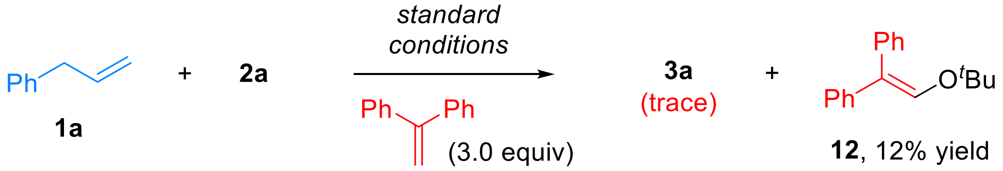

In a glove box, an oven-dried vial was charged with a stir bar, $Cu(MeCN)_4PF_6$ (1.5 mg, 4 μmol, 4 mol%), **L7** (4 mg, 5 μmol, 5 mol%) and DCE (0.25 mL). The mixture was stirred at 40 °C for 60 min before it was added to a separate photoreactor tube charged with the carboxylic acid **2a** (0.1 mmol, 1.0 equiv) and the iridium photocatalyst (0.7 mg, 0.7 μmol, 0.7 mol%). Next, the alkene **1a** (0.3 mmol, 3.0 equiv), 1.1-diphenylethylene (54.0 mg, 0.3 mmol, 3.0 equiv) and di-*tert*-butyl peroxide (75 μL, 0.4 mmol, 4.0 equiv) were added. The photoreactor tube was sealed with a rubber stopper and then removed from the glove box. The reaction mixture was stirred at –30 °C (internal temperature) with irradiation by 6 W 420 nm LEDs. After stirring for 36 h, the reaction mixture was quenched by exposure to air and warmed to room temperature. The resulting homogenous solution was transferred to a 50-mL round-bottom flask with the aid of EtOAc (2 x 3 mL). Silica was added to this solution and the volatiles were removed under reduced pressure. Purification by silica gel flash chromatography (eluent: *n*-hexane/EtOAc = 50:1 → 20:1) to afford the pure product **12** (6.1 mg, 12% yield).

^{1}H NMR (400 MHz, $CDCl_3$) δ 7.44 (dd, *J* = 8.3, 1.2 Hz, 2H), 7.29 (td, *J* = 6.9, 6.3, 1.3 Hz, 4H), 7.24 – 7.16 (m, 4H), 6.71 (s, 1H), 1.38 (s, 9H) ppm.

^{13}C NMR (101 MHz, $CDCl_3$) δ 141.4, 139.8, 138.1, 129.8, 128.6, 128.2, 127.7, 126.2, 126.0, 120.2, 77.4, 28.1 ppm.

HRMS (ESI) for **12**: m/z calculated for $C_{18}H_{20}NaO$ $[M + Na]^+$: 275.1412, found: 275.1412.

(3) KIE experiments

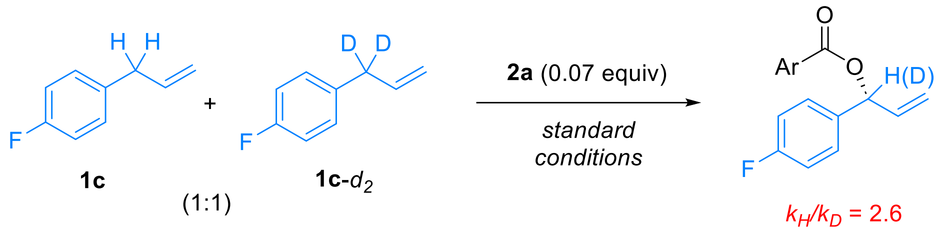

In a glove box, an oven-dried vial was charged with a stir bar, $Cu(MeCN)_4PF_6$ (1.5 mg, 4 μmol, 4 mol%), **L7** (4 mg, 5 μmol, 5 mol%) and DCE (0.25 mL). The mixture was stirred at 40 °C for 60 min before it was added to a separate photoreactor tube charged with the carboxylic acid **2a** (0.1 mmol, 1.0 equiv), TEMPO (31.2 mg, 0.2 mmol, 2.0 equiv) and the iridium photocatalyst (0.7 mg, 0.7 μmol, 0.7 mol%). Next, the alkene **1c** (0.15 mmol, 1.5 equiv), **1c-*d*$_2$** (0.15 mmol, 1.5 equiv) and di-*tert*-butyl peroxide (75 μL, 0.4 mmol, 4.0 equiv) were added. The photoreactor tube was sealed with a rubber stopper and then removed from the glove box. The reaction mixture was stirred at –30 °C (internal temperature) with irradiation by 6 W 420 nm LEDs. After stirring for 36 h, the reaction mixture was quenched by exposure to air and warmed to room temperature. The resulting homogenous solution was transferred to a 50-mL round-bottom flask with the aid of EtOAc (2 x 3 mL). Silica was added to this solution and the volatiles were removed under reduced pressure. The residue was purified by silica gel flash chromatography (eluent: *n*-hexane/EtOAc = 10:1 → 5:1) to afford **3c** and **3c-*d*$_1$**. Integration in the ^1H NMR spectrum indicated the ratio of these two compounds as 0.72 : 0.28, suggesting the KIE value was 2.6.

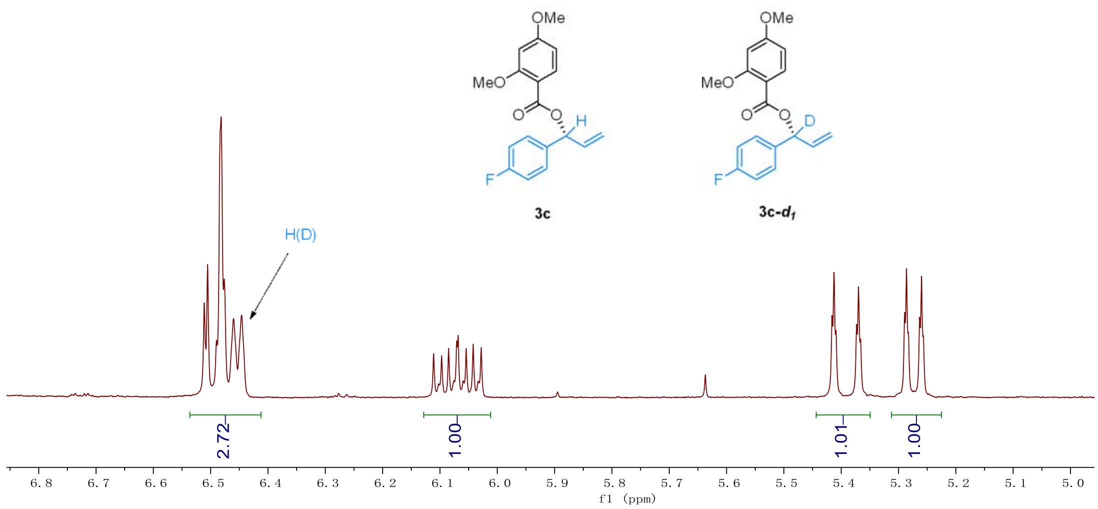

Figure S1. ^{1}H NMR spectra of the mixture of **3c** and **3c-*d*₁** in $CDCl_3$

(4) Non-linear effects

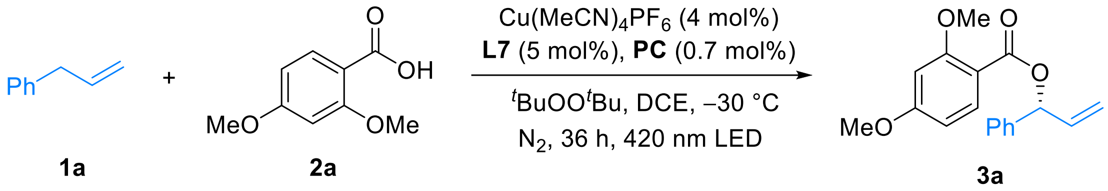

entry	ee% of **L7**	ee% of **3a**
1	0	0
2	20	21
3	40	39
4	60	58
5	80	78
6	100	94

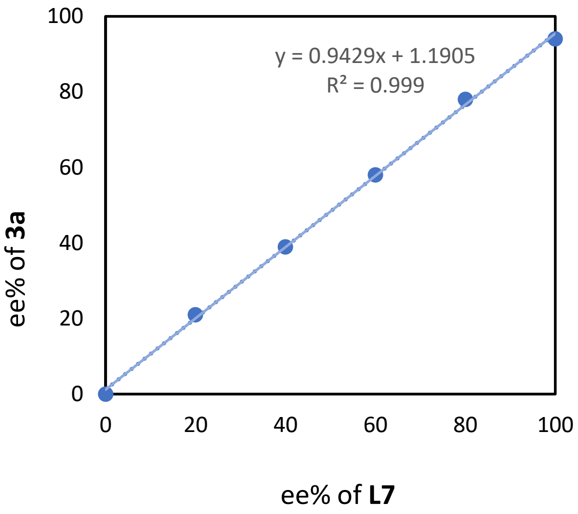

Figure S2. Non-linear effects between the catalyst and product.

(5) Light on-off experiments

In a glove box, an oven-dried vial was charged with a stir bar, $Cu(MeCN)_4PF_6$ (1.5 mg, 4 μmol, 4 mol%), **L7** (4 mg, 5 μmol, 5 mol%) and DCE (0.25 mL). The mixture was stirred at 40 °C for 60 min before it was added to a separate photoreactor tube charged with the carboxylic acid **2a** (0.1 mmol, 1.0 equiv) and the iridium photocatalyst (0.7 mg, 0.7 μmol, 0.7 mol%). Next, the alkene **1a** (0.3 mmol, 3.0 equiv), CH_2Br_2 (7 μL, 0.1 mmol) and di-*tert*-butyl peroxide (75 μL, 0.4 mmol, 4.0 equiv) were added. The photoreactor tube was sealed with a rubber stopper and then removed from the glove box. The reaction mixture was stirred at –30 °C (internal temperature) with irradiation by 6 W 420 nm LEDs. After 2 h, the light was turned off, the photoreaction tube was removed and an aliquot of the reaction mixture (20 μL) was taken out by micro syringe. The yield of **3a** was determined by ^{1}H NMR analysis with CH_2Br_2 as an internal standard. The major reaction mixture was then stirred in the absence of light for an additional 2 h. The same operation was repeated.

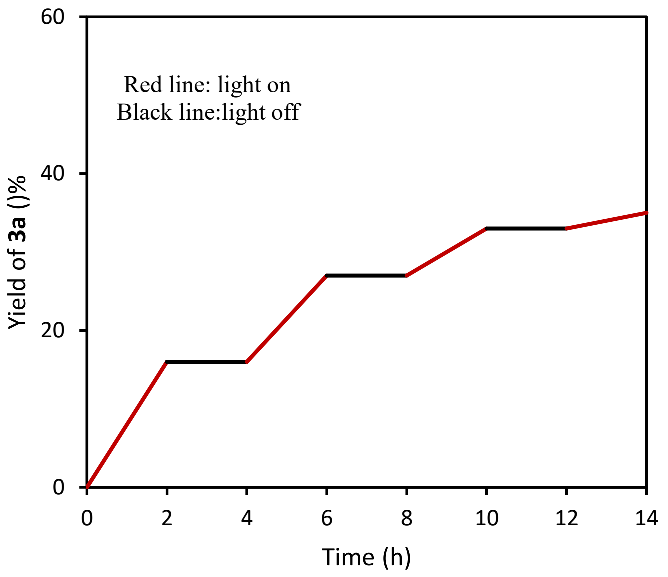

Figure S3. Light on-off experiment.

(6) Catalytic activity of intermediate IIa

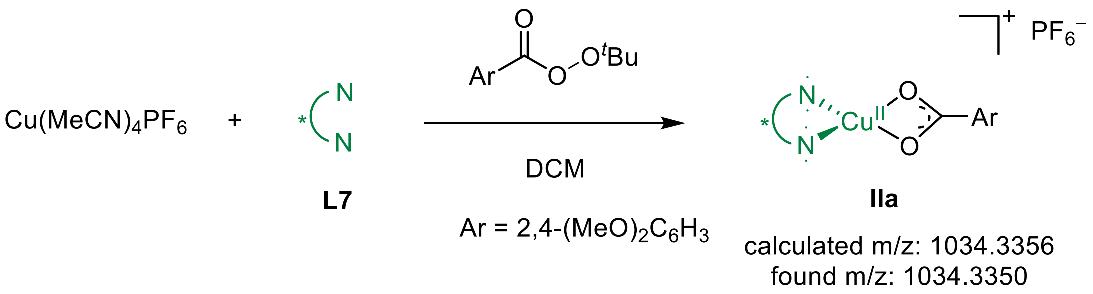

In a glove box, an oven-dried 4-mL vial was charged with a stir bar, $Cu(MeCN)_4PF_6$ (18.6 mg, 0.05 mmol), **L7** (40 mg, 0.05 mmol), and DCE (1.0 mL). The mixture was stirred at 40 ^{o}C for 60 min. Then, *tert*-butyl 2,4-dimethoxybenzoperoxoate (15.2 mg, 0.06 mmol) was added and stirred at room temperature for 30 min to form a homogeneous green solution. Then, additional *n*-hexane (5 mL) was added slowly. The vial was sealed and placed in a refrigerator overnight to produce a green solid precipitate. The solvent was removed under reduced pressure to give intermediate **IIa** as a green solid.

HRMS (ESI) for **IIa**: m/z calculated for $C_{66}H_{55}CuN_2O_6$ $[M]^+$: 1034.3356, found: 1034.3350.

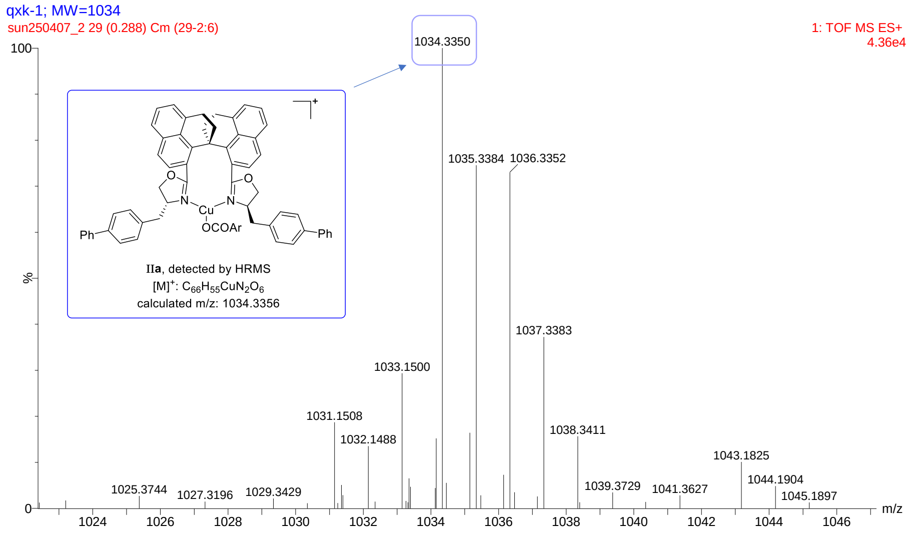

Figure S4. HRMS of **IIa.**

1a + **2a** —— **IIa** (4 mol%), *standard conditions* ——→ **3a** 86% yield, 91% ee

In a glove box, an oven-dried vial was charged with a stir bar, **IIa** (5.5 mg, 4 μmol, 4 mol%), carboxylic acid **2a** (0.1 mmol, 1.0 equiv), the iridium photocatalyst (0.7 mg, 0.7 μmol, 0.7 mol%), and DCE (0.25 mL). Next, the alkene **1a** (0.3 mmol, 3.0 equiv) and di-*tert*-butyl peroxide (75 μL, 0.4 mmol, 4.0 equiv) were added. The photoreactor tube was sealed with a rubber stopper and then removed from the glove box. The reaction mixture was stirred at –30 °C with irradiation by 6 W 420 nm LEDs. After stirring for 36 h, the reaction mixture was quenched by exposure to air and warmed to room temperature. The reaction mixture was filtered through a short pad of silica. The yield of **3a** was determined by ^{1}H NMR analysis with CH_2Br_2 (7 μL, 0.1 mmol) as an internal standard.

(7) Preparation of the Cu/L7 Complex

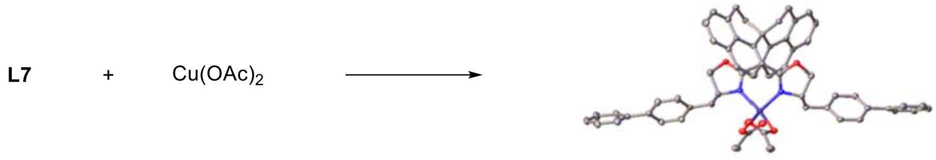

L7·$Cu(OAc)_2$

In a glove box, an oven-dried 4-mL vial was charged with a stir bar, $Cu(OAc)_2$ (1.8 mg, 10 μmol), **L7** (8 mg, 10 μmol), and DCM (0.3 mL). The mixture was stirred at 40 °C for 60 min. Then, *n*-hexane (2 mL) was added slowly. The vial was sealed and placed in the refrigerator overnight. Green crystaline precipitate was collected and subjected to X-ray diffraction. The structural parameters were measured by the following method.

Dihedral angle: The .cif file was opened in Olex2 software. The two naphthalene rings were selected to define the two planes. The dihedral angle of these two planes was analyzed by Olex2.

Bite angle: The angle of three connected atoms, N−Cu−N.

Cone angle: Cu was defined as the vertex, and the outermost edge of the ligand moiety was set to define the cone. The angle of this cone was analyzed by Olex2.

(8) Preparation of the Cu/L12 Complex

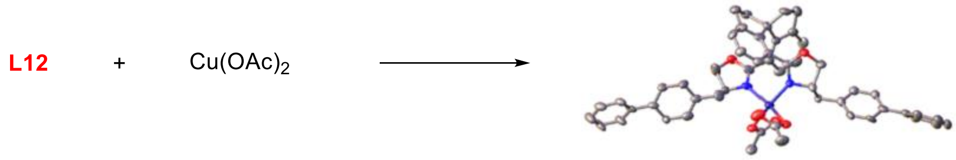

L12·$Cu(OAc)_2$

In a glove box, an oven-dried 4-mL vial was charged with a stir bar, $Cu(OAc)_2$ (1.8 mg, 10 μmol), **L12** (7 mg, 10 μmol), and DCM (0.3 mL). The mixture was stirred at 40 °C for 60 min. Then, *n*-hexane (2 mL) was added slowly. The vial was sealed and placed in the refrigerator overnight. Green crystaline

precipitate was collected and subjected to X-ray diffraction. The structural parameters were measured using the same method described above.

(9) Comparison of Steric Maps of L7·Cu(OAc)$_2$ and L12·Cu(OAc)$_2$

L7·Cu(OAc)$_2$ and **L12·Cu(OAc)$_2$** were exported from Olex2, respectively. Then, the steric maps of the two complexes were obtained by following the steps shown in the following URL.

https://www.aocdweb.com/OMtools/sambvca2.0/index.html

Step 1 : Load file - **L7·Cu(OAc)$_2$** exported from Olex2.xyz

Step 2: Select the atoms coordinated to the center of the sphere – Cu atom

Step 3: Select the atoms for z axis definition – N(1), N(2) atoms (Z-negative)

Step 4: Select the atoms for xz-plane definition – N(1)

Step 5: Select the atoms to be deleted – the atoms of Cu(OAc)$_2$

Step 6: Check the chosen orientation – default

Step 7: Select the atomic radii – default (Bondi radii scaled by 1.17)

Step 8: Sphere radius – default (3.5)

Step 9: Distance of the coordination point from the center of the sphere – default (0.0)

Step 10: Mesh spacing for numerical integration – default (0.10)

Step 11: Check the box below to include H atoms in the calculations – default

Step 12: Input completed – submit, and then obtain SambVca – Result of **L7·Cu(OAc)$_2$**

SambVca - Result

122

L7·Cu(OAc)$_2$ exported from Olex2

Cu	-0.3897	-4.3952	-19.0981
O	-2.2313	-3.7206	-18.9345
O	3.6888	-5.0156	-19.9806
O	1.3874	-1.2589	-16.9850

O	-0.3494	-6.7555	-17.8553
O	-1.0122	-6.0783	-19.8503
O	-1.6427	-3.0504	-20.9576
N	1.4683	-4.8330	-19.7476
C	-1.9338	-3.8478	-9.3101
H	-1.6024	-4.6960	-9.5836
N	0.2086	-2.9377	-17.8868
C	2.6224	-4.7047	-19.2094
C	-2.1269	-2.8411	-10.2678
C	1.0336	-5.5569	-23.5019
C	4.4502	-0.9440	-17.4373
C	4.1361	-4.4120	-13.7386
H	3.8551	-5.1089	-13.1586
C	6.2209	-1.7755	-19.7522
H	6.4822	-0.9620	-19.2515
H	7.0409	-2.1751	-20.1358
C	5.7664	-0.9292	-22.0649
H	6.7072	-0.8639	-22.1858
C	2.8727	-5.4407	-15.5912
H	2.5799	-6.1336	-15.0112
C	3.6911	-3.3590	-17.3385
C	2.9548	-4.4304	-17.7831
C	-0.7247	-3.9787	-15.8000
H	-1.4594	-4.4689	-16.2456
H	0.0800	-4.5560	-15.8078
C	-0.8172	-6.9968	-18.9632
C	1.8268	-7.5481	-27.2446
C	5.5753	-2.7655	-18.8014
H	6.1912	-2.9392	-18.0461
H	5.4265	-3.6214	-19.2757
C	4.0793	-3.3279	-15.9437
C	-2.6235	-1.6114	-9.8311
H	-2.7749	-0.9164	-10.4611
C	1.6109	-1.1210	-21.5708
H	1.0407	-0.8975	-22.2974
C	-2.6726	-2.7969	-12.7313
H	-3.5106	-2.3949	-12.5371
C	1.1512	-2.0871	-18.0244
C	5.3470	-2.3839	-14.1056
H	5.9106	-1.7017	-13.7623
C	4.2256	-2.2499	-18.2476
C	3.8641	-1.4775	-20.6656
C	1.0634	-1.4657	-20.3705
H	0.1199	-1.4721	-20.2665
C	1.3530	-7.0097	-28.4405
H	0.8323	-6.2159	-28.4289
C	3.5693	-0.6379	-22.9695
H	3.0003	-0.3942	-23.6906
C	-1.7864	-3.0897	-11.6864
C	3.6799	-4.3880	-15.0817
C	-0.2194	-3.9437	-13.3264
H	0.6284	-4.3238	-13.5199
C	1.4899	-4.8452	-24.6113
H	1.6438	-3.9111	-24.5369
C	-0.4334	-2.6918	-16.5633
H	-1.2876	-2.1880	-16.6989
C	-2.4866	-3.1675	-20.0561
C	1.8952	-1.8164	-19.2760

C	0.7897	-4.8452	-22.1845
H	-0.1629	-4.9368	-21.9290
H	0.9890	-3.8809	-22.2866
C	-2.3409	-3.0900	-14.0466
H	-2.9602	-2.8895	-14.7391
C	3.2938	-1.8772	-19.4042
C	2.5164	-5.4629	-16.8981
H	1.9739	-6.1687	-17.2281
C	0.8371	-6.9212	-23.6386
H	0.5142	-7.4213	-22.8982
C	2.8955	-9.3106	-28.5023
H	3.4326	-10.0941	-28.5184
C	1.7215	-5.4831	-25.8244
H	2.0123	-4.9763	-26.5737
C	4.9749	-3.4459	-13.2717
H	5.3064	-3.4910	-12.3827
C	3.1640	-5.1863	-21.3316
H	3.5864	-5.9579	-21.7851
H	3.3065	-4.3720	-21.8764
C	-2.2181	-3.6284	-7.9740
H	-2.0820	-4.3242	-7.3417
C	0.5844	-1.7828	-15.8850
H	0.1321	-1.0489	-15.3969
H	1.1449	-2.2934	-15.2493
C	-0.5651	-3.6597	-12.0084
H	0.0520	-3.8593	-11.3139
C	4.9153	-2.3046	-15.4104
C	1.6425	-7.6329	-29.6542
H	1.3029	-7.2707	-30.4638

C	-2.6937	-2.4115	-7.5557
H	-2.8824	-2.2647	-6.6363
C	1.6706	-5.4352	-21.0919
H	1.5071	-6.4210	-21.0370
C	-1.1143	-3.6726	-14.3692
C	5.2838	-1.3957	-20.8659
C	3.0043	-1.0915	-21.7490
C	2.5831	-8.7022	-27.2947
H	2.8973	-9.0889	-26.4853
C	1.1038	-7.5739	-24.8370
H	0.9897	-8.5161	-24.8945
C	2.4182	-8.7667	-29.6864
H	2.6256	-9.1790	-30.5170
C	4.9223	-0.5476	-23.1144
H	5.2924	-0.2240	-23.9278
C	1.5337	-6.8622	-25.9541
C	-2.9007	-1.3901	-8.4844
H	-3.2306	-0.5462	-8.1985
C	-3.9168	-2.6807	-20.2616
H	-4.4783	-3.4291	-20.5519
H	-3.9277	-1.9791	-20.9459
H	-4.2631	-2.3199	-19.4187
C	-1.2214	-8.3888	-19.3867
H	-0.6840	-8.6660	-20.1576
H	-2.1708	-8.3936	-19.6321
H	-1.0739	-9.0123	-18.6446
C	5.3715	-1.1523	-16.2563
H	6.2897	-1.3256	-16.5822
H	5.3920	-0.3288	-15.7064

H	4.8386	-0.2544	-18.0332
H	3.5778	-0.6075	-17.1129

SambVca - Result

110

L12·Cu(OAc)₂ exported from Olex2

Cu	4.8162	4.8874	3.0612
O	8.6608	5.8599	4.4846
O	6.8108	1.4595	1.7165
O	4.0790	6.6547	3.5179
O	4.9757	6.9612	1.5282
O	3.0078	4.2494	2.5711
O	3.2315	3.7735	4.7107
N	6.5269	5.4431	3.9733
N	5.5782	3.2654	2.1816
C	9.4841	2.7989	3.2786
C	10.0173	1.6148	2.4147
H	10.7501	1.1431	2.8858
H	9.2913	0.9677	2.2270
C	10.5253	2.2373	1.1252
H	11.4898	2.4521	1.1894
H	10.3811	1.6303	0.3568
C	9.6998	3.4956	0.9859
C	9.1237	3.8172	2.2265
C	10.5750	3.2661	4.2974
H	10.4800	4.2314	4.4962
H	11.4832	3.1012	3.9406
C	10.3245	2.4240	5.5572
H	10.8290	1.5725	5.5243
H	10.5802	2.9208	6.3757
C	8.8280	2.1783	5.5118
C	8.3624	2.3966	4.2192
C	7.7459	5.4005	3.6156
C	8.3184	4.9471	2.3254
C	8.0677	5.7299	1.1925
H	7.4852	6.4786	1.2451
C	8.6838	5.3981	-0.0142
H	8.5397	5.9438	-0.7786
C	9.4971	4.2897	-0.1135
H	9.9150	4.0768	-0.9396
C	7.9579	6.1553	5.7059
H	8.1621	5.4812	6.4023
H	8.1999	7.0536	6.0455
C	6.4811	6.0986	5.3032
H	6.1180	7.0260	5.2128
C	5.6575	5.3155	6.3236
H	6.0289	4.4019	6.4114
H	4.7256	5.2360	5.9993
C	6.4241	2.3996	2.5881
C	6.9816	2.2522	3.9570
C	6.1283	1.9094	4.9877
H	5.1948	1.8449	4.8231
C	6.6176	1.6587	6.2568
H	6.0220	1.3988	6.9500
C	7.9699	1.7848	6.5228
H	8.3063	1.6023	7.3929
C	6.2125	1.8128	0.4488
H	6.8887	2.1829	-0.1707

H	5.7886	1.0238	0.0268	H	2.7512	3.1428	-1.0083
C	5.1682	2.8650	0.8038	C	2.9178	2.6719	-2.9814
H	4.2674	2.4323	0.8430	H	2.0204	2.3768	-3.0824
C	5.1248	4.0146	-0.1889	C	3.7733	2.6552	-4.0777
H	4.4989	4.7099	0.1352	C	5.0946	3.0315	-3.8833
H	6.0243	4.4214	-0.2656	H	5.7008	3.0067	-4.6143
C	5.6591	5.9845	7.6729	C	5.5453	3.4456	-2.6320
C	6.3678	5.4328	8.7336	H	6.4586	3.6807	-2.5177
H	6.8554	4.6279	8.6031	C	3.2604	2.2604	-5.4170
C	6.3733	6.0429	9.9840	C	2.4811	1.1310	-5.5901
H	6.8594	5.6461	10.6975	H	2.3005	0.5685	-4.8467
C	5.6738	7.2312	10.2038	C	1.9547	0.8049	-6.8436
C	4.9915	7.7868	9.1186	H	1.4158	0.0280	-6.9453
H	4.5122	8.5992	9.2319	C	2.2156	1.5986	-7.9172
C	5.0052	7.1668	7.8777	H	1.8603	1.3764	-8.7685
H	4.5482	7.5747	7.1516	C	2.9999	2.7322	-7.7748
C	5.6208	7.8587	11.5528	H	3.1835	3.2850	-8.5260
C	5.6395	7.0844	12.7082	C	3.5181	3.0627	-6.5228
H	5.7154	6.1404	12.6329	H	4.0536	3.8415	-6.4258
C	5.5503	7.6561	13.9619	C	4.3582	7.3902	2.5104
H	5.5751	7.1098	14.7387	C	3.8734	8.8166	2.5697
C	5.4248	9.0201	14.0797	H	3.9575	9.1527	3.4868
H	5.3545	9.4200	14.9386	H	4.4140	9.3712	1.9687
C	5.4025	9.8118	12.9392	H	2.9347	8.8539	2.3005
H	5.3098	10.7536	13.0206	C	2.5404	3.8282	3.6811
C	5.5123	9.2435	11.6859	C	1.1052	3.3485	3.6745
H	5.5158	9.7959	10.9130	H	0.5114	4.1029	3.4758
C	4.6750	3.5197	-1.5442	H	0.9954	2.6575	2.9891
C	3.3596	3.1158	-1.7373	H	0.8804	2.9787	4.5525

VII. Product Derivatizations

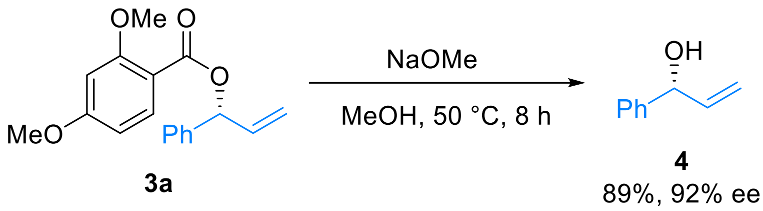

(*R*)-1-Phenylprop-2-en-1-ol (4) was synthesized according to a modified literature procedure.[5] To a solution of **3a** (59.6 mg, 0.2 mmol, 1.0 equiv) in MeOH (2.0 mL) was added NaOMe (32.4 mg, 0.6 mmol, 3.0 equiv). The reaction mixture was stirred at 50 °C for 8 h before it was diluted with water (10 mL) and extracted with EtOAc (3 x 10 mL). The combined organic layers were dried over anhydrous $MgSO_4$ and filtered. Silica was added to this solution and the volatiles were removed under reduced pressure. The residue was purified by silica gel flash chromatography (eluent: EtOAc/*n*-hexane = 1:10 → 1:3) to afford the desired product as a pale-yellow oil (23.9 mg, 89% yield, 92% ee).

$[\alpha]_D^{25}$: +4.2 (c = 0.5, Ph). HPLC analysis of the product: Daicel CHIRALPAK® OD-3 column; 5% *i*-PrOH in *n*-hexane; 1.0 mL/min; retention times: 9.7 min (major), 12.2 min (minor).

^{1}H NMR (400 MHz, $CDCl_3$) δ 7.38 – 7.33 (m, 4H), 7.29 (dp, J = 8.2, 2.8 Hz, 1H), 6.10 – 5.98 (m, 1H), 5.38 – 5.30 (m, 1H), 5.23 – 5.13 (m, 2H), 2.09 (s, 1H) ppm.

^{13}C NMR (101 MHz, $CDCl_3$) δ 142.5, 140.2, 128.5, 127.7, 126.3, 115.1, 75.3 ppm.

HRMS (ESI) Calcd for $C_9H_{11}O$ $[M + H]^+$: 134.0732, found: 134.0736.

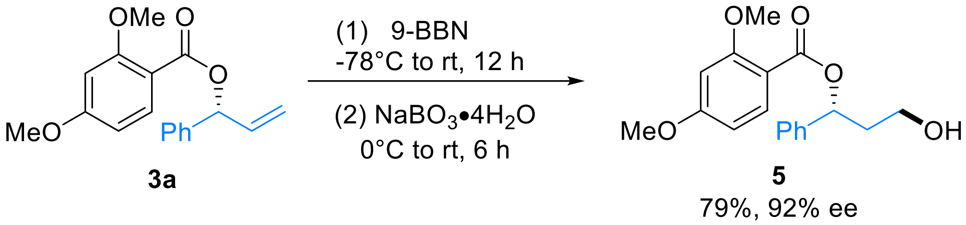

(*R*)-3-Hydroxy-1-phenylpropyl 2,4-dimethoxybenzoate (5) was synthesized according to a modified literature procedure.[6] At –78 °C, to a solution of **3a** (59.6 mg, 0.2 mmol, 1.0 equiv) in dry THF (1.0 mL) was added a solution of 9-BBN in THF (1.2 mL, 0.5 M, 0.6 mmol, 3.0 equiv). The reaction mixture was stirred for 1 h at –78 °C and then allowed to warm slowly to room temperature and kept stirring for 12 h before it was cooled to 0 °C and treated with water (2.0 mL) and $NaBO_3 \cdot 4H_2O$ (461.6 mg, 3.0 mmol, 15.0 equiv). The reaction mixture was allowed to warm to room temperature and kept stirring for 6 h. The reaction mixture was diluted with water (20 mL) and extracted with EtOAc (3 x 20 mL). The combined organic layers were dried over anhydrous $MgSO_4$ and filtered. Silica was added to this solution and the volatiles were removed under reduced pressure. The residue was purified by silica gel flash chromatography (eluent: EtOAc/*n*-hexane = 1:5 → 1:1) to afford the desired product **5** as a colorless oil (50.0 mg, 79% yield, 92% ee).

$[\alpha]_D^{24}$: –31.5 (*c* = 1.0, $CHCl_3$). HPLC analysis of the product: Daicel CHIRALPAK® AD-3 column; 30% *i*-PrOH in *n*-hexane; 1.0 mL/min; retention times: 10.1 min (major), 19.4 min (minor).

^{1}H NMR (400 MHz, $CDCl_3$) δ 7.90 (d, *J* = 8.6 Hz, 1H), 7.41 (d, *J* = 7.1 Hz, 2H), 7.35 (t, *J* = 7.4 Hz, 2H), 7.29 (d, *J* = 7.2 Hz, 1H), 6.55 – 6.46 (m, 2H), 6.24 – 6.13 (m, 1H), 3.90 (s, 3H), 3.85 (s, 3H), 3.75 (t, *J* = 4.9 Hz, 2H), 3.01(s, 1H), 2.24 – 2.13 (m, 2H) ppm.

^{13}C NMR (101 MHz, $CDCl_3$) δ 165.5, 164.6, 161.1, 140.7, 134.5, 128.5, 127.7, 126.1, 111.8, 104.8, 98.9, 74.6, 59.3, 55.8, 55.5, 39.7 ppm.

HRMS (ESI) Calcd for $C_{18}H_{20}NaO_5$ $[M + Na]^+$: 339.1208, found: 339.1218.

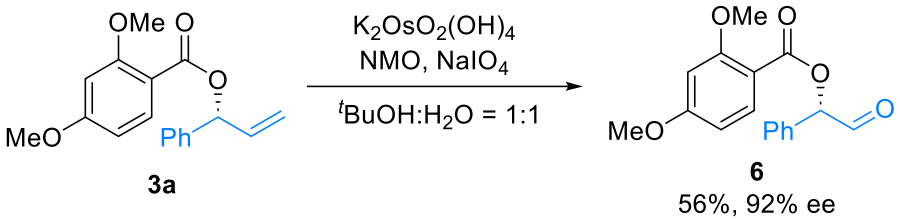

(*S*)-2-Oxo-1-phenylethyl 2,4-dimethoxybenzoate (6) was synthesized according to a modified literature procedure.[7] A mixture of **3a** (59.6 mg, 0.2 mmol, 1.0 equiv), ^{t}BuOH (1.2 mL), H_2O (1.2 mL), $K_2OsO_2(OH)_4$ (1.2 mg, 3.2 μmol, 0.016 equiv), NMO (70.3 mg, 0.6 mmol, 3.0 equiv), and $NaIO_4$ (128.3 mg, 0.6 mmol, 3.0 equiv) was stirred at room temperature for 3 h. The mixture was diluted with water (3 mL) and extracted with diethyl ether (3 x 3 mL). The combined organic layers were dried over anhydrous $MgSO_4$ and filtered. Silica was added to this solution and the volatiles were removed under reduced pressure. The residue was purified by silica gel flash chromatography (eluent: EtOAc/*n*-hexane = 1:4 → 1:1) to afford **6** as a colorless oil (33.6 mg, 56% yield, 92% ee).

$[\alpha]_D^{24}$: +24.7 (*c* = 1.0, $CHCl_3$). HPLC analysis of the product: Daicel CHIRALPAK® AD-3 column; 30% *i*-PrOH in *n*-hexane; 1.0 mL/min; retention times: 14.9 min (major), 16.9 min (minor).

^{1}H NMR (400 MHz, $CDCl_3$) δ 9.66 (s, 1H), 8.05 (d, *J* = 8.7 Hz, 1H), 7.51 (dd, *J* = 8.1, 1.3 Hz, 2H), 7.47 – 7.37 (m, 3H), 6.57 – 6.47 (m, 2H), 6.19 (s, 1H), 3.92 (s, 3H), 3.87 (s, 3H) ppm.

^{13}C NMR (101 MHz, $CDCl_3$) δ 195.0, 165.0, 164.3, 162.1, 134.4, 132.0, 129.1, 129.0, 127.7, 110.6, 104.8, 98.9, 80.2, 55.9, 55.5 ppm.

HRMS (ESI) Calcd for $C_{17}H_{16}NaO_5$ $[M + Na]^+$: 323.0895, found: 323.0899.

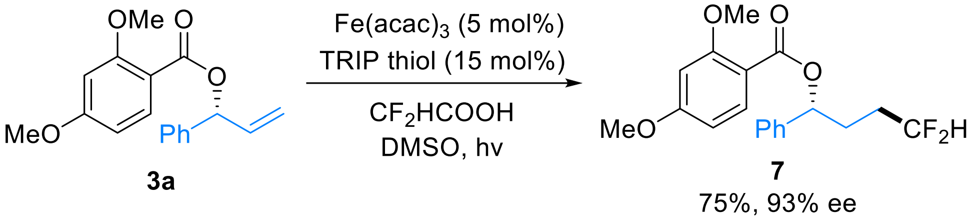

(*R*)-4,4-Difluoro-1-phenylbutyl 2,4-dimethoxybenzoate (7) was synthesized according to a modified literature procedure.[8] To an oven-dried 4-mL vial charged with a stir bar and $Fe(acac)_3$ (3.5 mg, 0.01 mmol, 5% mmol) were added dry dimethyl sulfoxide (2 mL, 0.1 M) and alkene **3a** (59.6 mg, 0.2 mmol, 1.0 equiv), 2,4,6-triisopropylbenzenethiol (TRIP, 8 μL, 0.04 mmol, 20% mmol) and difluoroacetic acid (96 mg, 1.0 mmol, 5.0 equiv). The vial was sealed, evacuated and backfilled with N_2 three times. The mixture was stirred and irradiated using a 12 W 390 nm LED lamp for 48 h until the reaction was complete (monitored by TLC). Then, the mixture was quenched by a saturated aqueous solution of $NaHCO_3$ (5 mL). EtOAc (5 mL) was added and the layers were separated. The aqueous layer was extracted with EtOAc (2 x 5 mL). The combined organic layers were dried over anhydrous $MgSO_4$ and filtered. Silica was added to this solution and the volatiles were removed under reduced pressure. The crude product was extracted and purified by silica gel flash chromatography (eluent: EtOAc/*n*-hexane = 1:10 → 1:5) to give the product (52.5 mg, 75% yield, 93% ee) as a colorless oil.

$[\alpha]_D^{24}$: –24.2 (*c* = 0.5, $CHCl_3$). HPLC analysis of the product: Daicel CHIRALPAK® AD-3 column; 20% *i*-PrOH in *n*-hexane; 1.0 mL/min; retention times: 11.2 min (major), 24.3 min (minor).

^{1}H NMR (400 MHz, $CDCl_3$) δ 7.90 (d, *J* = 8.6 Hz, 1H), 7.40 (d, *J* = 7.0 Hz, 2H), 7.35 (t, *J* = 7.4 Hz, 2H), 7.29 (d, *J* = 7.2 Hz, 1H), 6.55 – 6.46 (m, 2H), 6.05 – 5.66 (m, 2H), 3.89 (s, 3H), 3.85 (s, 3H), 2.24 – 1.87 (m, 4H) ppm.

^{19}F NMR (377 MHz, $CDCl_3$) δ -116.11 (dt, *J* = 56.9, 17.3 Hz) ppm.

^{13}C NMR (101 MHz, $CDCl_3$) δ 164.7, 164.4, 161.6, 140.2, 133.9, 128.5, 127.9, 126.3,

116.9 (t, J = 239.0 Hz), 112.0, 104.6, 98.9, 74.7, 55.8, 55.5, 30.3 (t, J = 21.4 Hz), 29.1 (t, J = 5.4 Hz) ppm.

HRMS (ESI) Calcd for $C_{19}H_{20}F_2NaO_4$ $[M + Na]^+$: 373.1227, found: 373.1233.

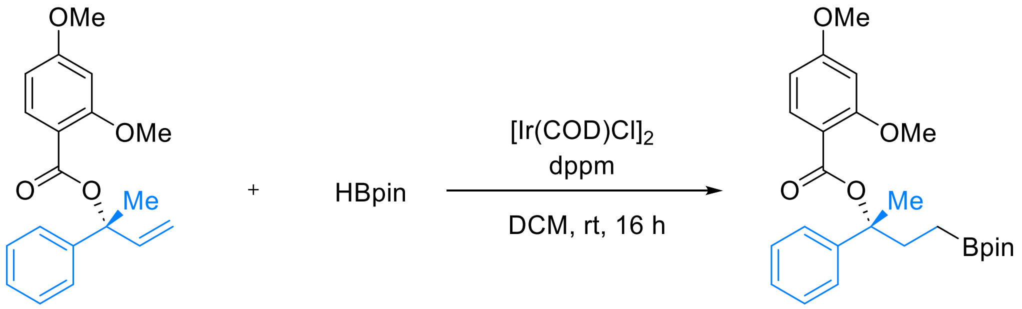

3t

8
87%, 92% ee

(*R*)-2-Phenyl-4-(4,4,5,5-tetramethyl-1,3,2-dioxaborolan-2-yl)butan-2-yl 2,4-dimethoxybenzoate (8) was synthesized according to a modified literature procedure.[9] Under N_2, an oven-dried 4-mL vial was charged with a stir bar, $[Ir(COD)Cl]_2$ (6.7 mg, 0.01 mmol, 5 mol%), bis(diphenylphosphino)methane (dppm, 7.7 mg, 0.02 mmol, 10 mol%) and DCM (2.0 mL). After stirring for 10 min at room temperature, HBpin (30.7 mg, 0.24 mmol, 1.2 equiv) and **3t** (62.4 mg, 0.2 mmol, 1.0 equiv) were added sequentially. The mixture was stirred at room temperature for 16 h and then filtered through a short pad of silica. The filtrate was concentrated and subjected to silica gel flash chromatography (eluent: EtOAc/*n*-hexane = 1:10 → 1:5) to give pure boronate **8** (76.6 mg, 87% yield, 92% ee) as a colorless oil.

Note: The product should be quickly separated and concentrated by rotary evaporation (water bath temperature < 40 °C).

$[\alpha]_D^{24}$: –16.9 (c = 1.0, $CHCl_3$). HPLC analysis of the product: Daicel CHIRALPAK® ID-3 column; 20% *i*-PrOH in *n*-hexane; 1.0 mL/min; retention times: 9.1 min (major), 11.3 min (minor).

^{1}H NMR (400 MHz, $CDCl_3$) δ 7.95 (d, J = 8.6 Hz, 1H), 7.38 (d, J = 7.2 Hz, 2H),

7.29 (t, J = 7.7 Hz, 2H), 7.20 (t, J = 7.3 Hz, 1H), 6.58 – 6.36 (m, 2H), 3.86 (d, J = 4.0 Hz, 6H), 2.24 – 2.06 (m, 2H), 1.97 (s, 3H), 1.21 (s, 12H), 0.86 – 0.70 (m, 2H) ppm.

^{13}C NMR (101 MHz, $CDCl_3$) δ 164.1, 164.0, 161.5, 145.2, 133.9, 128.0, 126.5, 124.9, 113.6, 104.3, 98.9, 84.7, 83.0, 55.7, 55.5, 37.5, 24.8, 24.8, 24.6 ppm.

HRMS (ESI) Calcd for $C_{25}H_{33}BNaO_6$ [M + Na]$^+$: 463.2268, found: 463.2268.

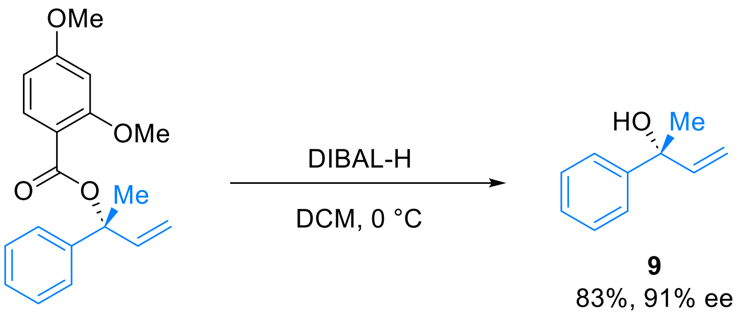

3t

9
83%, 91% ee

(*R*)-2-Phenylbut-3-en-2-ol (9). At 0 °C under N_2, a solution of DIBAL-H in *n*-hexane (1.0 M, 0.6 mL, 0.6 mmol, 2.0 equiv) was added slowly to a solution of **3t** (93.6 mg, 0.3 mmol, 1.0 equiv) in dry CH_2Cl_2 (2 mL). The mixture was stirred at 0 °C for 2 h, at which point TLC analysis indicated reaction completion. A saturated NH_4Cl solution (0.3 mL) was added dropwise at 0 °C, followed by an aqueous NaOH solution (10%) with stirring until the reaction was free of colloidal precipitate. DCM (3 x 3 mL) was added to extract the reaction mixture. The organic phases were combined and washed with brine, dried over anhydrous $MgSO_4$ and concentrated. The crude product was purified by silica gel flash chromatography (eluent: EtOAc/*n*-hexane = 1:10 → 1:7) to give the alcohol **9** (36.8 mg, 83% yield, 91% ee) as a colorless oil.

$[\alpha]_D^{24}$: +26.3 (c = 1.0, $CHCl_3$). HPLC analysis of the product: Daicel CHIRALCEL® OJ-3 column; 10% *i*-PrOH in *n*-hexane; 1.0 mL/min; retention times: 11.2 min (minor), 14.9 min (major).

^{1}H NMR (400 MHz, $CDCl_3$) δ 7.50 (dd, J = 8.4, 1.2 Hz, 2H), 7.37 (td, J = 6.9, 6.4, 1.7 Hz, 2H), 7.30 – 7.26 (m, 1H), 6.20 (dd, J = 17.3, 10.6 Hz, 1H), 5.32 (dd, J = 17.3, 1.0 Hz, 1H), 5.17 (dd, J = 10.6, 1.1 Hz, 1H), 2.08 (s, 1H), 1.68 (s, 3H) ppm.

^{13}**C NMR** (101 MHz, $CDCl_3$) δ 146.3, 144.8, 128.2, 126.9, 125.1, 112.3, 74.7, 29.3 ppm.

This is a known compound.

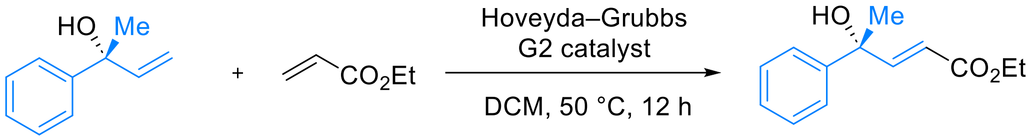

9

10
72%, 92% ee

Ethyl (*R*,*E*)-4-hydroxy-4-phenylpent-2-enoate (10) was synthesized according to a modified literature procedure.[10] Under N_2, the second-generation Hoveyda–Grubbs catalyst (6.26 mg, 0.01 mmol) and ethyl acrylate (84 mg, 0.84 mmol, 6.0 equiv) were added to an oven-dried 50-mL round-bottom flask. Then, a solution of **9** (20.7 mg, 0.14 mmol, 1.0 equiv) in DCM (0.5 mL) was added. The resulting mixture was allowed to stir at 50 °C for 12 h. The reaction mixture was filtered through a short pad of silica. The crude product was purified by silica gel flash chromatography (eluent: EtOAc/*n*-hexane = 1:10 → 1:5) to give ester **10** (22.2 mg, 72% yield, 92% ee) as a colorless oil.

$[\alpha]_D^{24}$: +10.6 (c = 0.5, $CHCl_3$). HPLC analysis of the product: Daicel CHIRALCEL® OD-3 column; 5% *i*-PrOH in *n*-hexane; 1.0 mL/min; retention times: 16.3 min (major), 24.8 min (minor).

^{1}**H NMR** (400 MHz, $CDCl_3$) δ 7.48 – 7.42 (m, 2H), 7.35 (t, J = 7.5 Hz, 2H), 7.30 – 7.25 (m, 1H), 7.15 (d, J = 15.6 Hz, 1H), 6.10 (d, J = 15.6 Hz, 1H), 4.18 (q, J = 7.1 Hz, 2H), 2.25 (s, 1H), 1.71 (s, 3H), 1.28 (t, J = 7.1 Hz, 3H) ppm.

^{13}**C NMR** (101 MHz, $CDCl_3$) δ 166.7, 153.1, 144.7, 128.5, 127.5, 125.1, 118.5, 74.2, 60.5, 29.0, 14.2 ppm.

HRMS (ESI) Calcd for $C_{13}H_{16}NaO_3$ $[M + Na]^+$: 243.0997, found: 243.1007.

VIII. Determination of Product Structures

(1) Determination of the product absolute configuration

The absolute stereochemistry of the product was determined by comparison of the optical rotation values with the literature values.

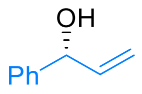

4

(*R*)-1-Phenylprop-2-en-1-ol. The literature value for (*S*)-**4** in 97% ee was reported to be $[\alpha]_D^{25}$: –5.9 (*c* = 1.73, benzene).[11] The measured value of our product in 92% ee is $[\alpha]_D^{25}$: +4.2 (*c* = 0.5, benzene). Thus, the absolute configuration of our product was assigned to be *R*.

(2) Determination of the structures of the copper complexes

The structures of the **Cu/L7** and **Cu/L12** complexes were determined by X-ray crystallography. Single crystals of the **Cu/L7** and **Cu/L12** complexes were obtained from dichloromethane and *n*-hexane by vapor deposition. The X-ray data has been deposited at the Cambridge Crystallographic Data Center (CCDC 2435187 for **Cu/L7** and CCDC 2435188 for **Cu/L12**).

A suitable crystal of **Cu/L7** was selected and mounted on a Rigaku-Oxford Diffraction Supernova diffractometer operating with a micro-focus Cu-K*α* source and Atlas detector with a MiTeGenTM loop. The crystal was kept at 100.00(10) K during data collection. Using Olex2,[12] the structure was solved with the SHELXT structure solution program using intrinsic phasing and refined with the SHELXL through least squares minimization.[13]

A suitable crystal of **Cu/L12** was selected and mounted on a Bruker Incoatec ImuS Ag Diamond II with PHOTO III diffractometer with a MiTeGen™ loop. The crystal was kept at 173.0(1) K during data collection. Using Olex2,[12] the structure was solved with the SHELXT[13] structure solution program using intrinsic phasing and refined with the olex2 via Gauss-Newton minimization.[14] The solvent molecules (i.e. DCM) were refined with free variables to obtain their optimum occupancies for disorder parts, as a result of a non-integer empirical formula.

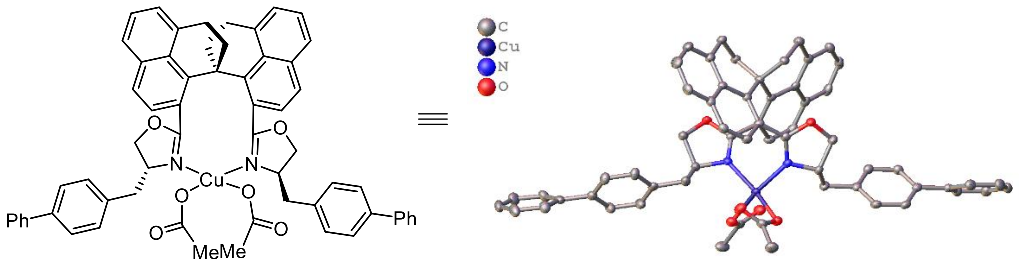

Table S10. Crystal Data and Structure Refinement for Cu/L7.

Identification code	**Cu/L7**
Empirical formula	$C_{123}H_{106}Cl_2Cu_2N_4O_{12}$
Formula weight	2030.09
Temperature/K	100.00(10)
Crystal system	orthorhombic
Space group	$P2_12_12_1$
a/Å	17.5483(2)
b/Å	18.4369(2)
c/Å	30.5799(3)
α/°	90
β/°	90
γ/°	90
Volume/Å^3	9893.7(2)
Z	4
ρ_{calc}g/cm^3	1.363
μ/mm^{-1}	1.572
F(000)	4240.0

Crystal size/mm^3	0.15 × 0.15 × 0.04
Radiation	Cu Kα (λ = 1.54184)
2Θ range for data collection/°	5.598 to 149.78
Index ranges	-21 ≤ h ≤ 17, -17 ≤ k ≤ 22, -29 ≤ l ≤ 37
Reflections collected	33529
Independent reflections	18049 [R_{int} = 0.0330, R_{sigma} = 0.0485]
Data/restraints/parameters	18049/0/1292
Goodness-of-fit on F^2	1.030
Final R indexes [I>=2σ (I)]	R_1 = 0.0392, wR_2 = 0.0965
Final R indexes [all data]	R_1 = 0.0429, wR_2 = 0.0984
Largest diff. peak/hole / e Å^{-3}	0.55/-0.71
Flack parameter	-0.025(9)

Table S11. Fractional Atomic Coordinates (×10^4) and Equivalent Isotropic Displacement Parameters (Å^2×10^3) for Cu/L7. U_{eq} is defined as 1/3 of the trace of the orthogonalised U_{IJ} tensor.

Atom	*x*	*y*	*z*	U(eq)
Cu1	-222.1(3)	-2383.9(3)	-6245.3(2)	19.07(11)
Cu2	4934.5(3)	2957.3(3)	-6208.2(2)	18.02(11)
Cl3	2169.8(8)	-1375.2(10)	-8525.6(5)	63.1(4)
Cl5	2628.9(10)	-215.0(10)	-9124.8(7)	78.0(5)
O6	-1271.5(14)	-2018.0(15)	-6191.8(8)	24.3(5)
O10	5910.4(14)	2635.4(16)	-5946.3(8)	24.7(5)
O61	3777.5(15)	991.7(14)	-5985.3(8)	21.0(5)
O119	2102.1(14)	-2720.4(15)	-6533.9(8)	22.3(5)
O98	790.6(15)	-682.8(14)	-5554.3(8)	21.1(5)
O37	2816.4(14)	3440.0(15)	-6787.9(8)	23.3(5)
O136	-199.1(17)	-3664.1(17)	-5838.9(9)	31.6(6)
O138	-576.8(15)	-3296.8(16)	-6491.3(8)	26.0(6)
O8	-936.1(16)	-1654.5(18)	-6853.4(9)	32.9(7)
N122	836.7(17)	-2621.4(17)	-6457.7(9)	19.5(6)
C114	-1102(2)	-2087(2)	-3044.5(12)	24.4(8)
N40	4010.6(17)	3271.1(16)	-6556.3(9)	17.5(6)
N58	4461.1(16)	2017.3(17)	-6008.5(8)	16.7(6)

O142	5344.1(15)	3937.6(15)	-6317.5(8)	25.3(6)
N101	118.9(17)	-1593.4(16)	-5849.2(9)	18.5(6)
O140	4710.9(18)	4042.0(17)	-5696.2(9)	34.4(7)
C20	2983(2)	1638(2)	-7318.2(12)	22.3(7)
C118	1494.4(19)	-2551.8(19)	-6281.7(11)	18.4(7)
C48	4314(2)	4449(2)	-9067.0(11)	21.2(7)
C14	2353(2)	1794(2)	-6568.0(12)	19.7(7)
C109	-1212(2)	-1541(2)	-3357.7(11)	21.1(7)
C127	589(2)	-3014(2)	-7685.4(11)	23.2(7)
O12	5941.8(17)	2206(2)	-6614.9(10)	43.8(9)
C84	2536(2)	-512(2)	-5702.2(11)	20.2(7)
C16	3799(2)	1608.6(19)	-6677.3(11)	17.4(7)
C34	3073(2)	3169(2)	-5702.3(12)	21.5(7)
C117	2357(2)	-2393(2)	-4492.7(11)	27.4(8)
C88	3545(2)	-963(2)	-6459.2(11)	21.7(7)
C27	2133(2)	1938(2)	-5745.0(12)	22.0(7)
C90	3286(2)	-504(2)	-7215.5(13)	28.2(8)
C79	1637(2)	-2951(2)	-5098.5(12)	24.9(8)
C60	4070(2)	1076(2)	-5538.5(11)	22.6(8)
C76	2103.4(19)	-1821.9(19)	-5669.9(11)	17.5(7)
C77	1683.8(19)	-2403(2)	-5815.3(11)	19.0(7)
C59	4657(2)	1687.3(19)	-5577.3(10)	18.0(7)
C29	1267(3)	1126(2)	-5367.1(15)	33.3(10)
C63	5521(2)	1223(2)	-4014.6(11)	21.4(7)
C64	4749(2)	1401(2)	-4052.0(11)	23.4(7)
C102	-413(2)	-2158(2)	-5166.8(11)	23.4(8)
C15	3071(2)	1704.1(19)	-6849.4(12)	18.9(7)
C65	4463(2)	1712(2)	-4429.5(11)	23.2(8)
C137	-465.7(19)	-3795(2)	-6201.2(13)	23.2(7)
C38	3283(2)	3638(2)	-7159.7(12)	25.3(8)
C130	1041(2)	-4094(2)	-8909.3(12)	24.9(8)
C33	2766(2)	2900(2)	-5324.8(12)	25.6(8)
C89	3177.1(19)	-1500(2)	-6148.3(11)	19.9(7)
C81	2324.6(19)	-1805(2)	-5213.8(11)	19.8(7)
C110	-1495(2)	-874(2)	-3214.9(13)	27.2(8)

C69	5836(2)	868(2)	-3618.2(11)	21.2(7)
C62	4648(2)	2220.6(19)	-5198.6(11)	21.2(7)
C94	918(2)	-608(2)	-7053.9(12)	22.3(7)
C108	-1523(2)	-1517(2)	-4163.3(12)	22.0(7)
C31	1941(3)	2014(2)	-4949.7(14)	34.2(10)
C28	2488.4(19)	2199.2(19)	-6137.6(11)	19.2(7)
C97	656(2)	-1132(2)	-5894.2(11)	19.0(7)
C115	3047(2)	-1293(2)	-4612.7(12)	25.9(8)
C32	2292(2)	2284(2)	-5335.4(12)	26.2(8)
C57	4005.7(18)	1587.1(19)	-6205.9(11)	17.5(6)
C23	1714(2)	2195(2)	-6824.6(13)	24.1(8)
C75	2408(2)	-1220.3(19)	-5967.2(10)	17.0(7)
C35	2933.4(19)	2821(2)	-6106.4(11)	19.0(7)
C11	6225(2)	2261(2)	-6248.1(12)	26.3(8)
C86	2202(2)	-801.4(19)	-6757.9(11)	18.4(7)
C42	4363(2)	4219(2)	-8599.7(11)	20.4(7)
C95	606(2)	-795(2)	-6661.4(12)	22.5(7)
C39	4079(2)	3724(2)	-6961.3(11)	19.2(7)
C74	5659(2)	1134(2)	-3198.7(12)	25.6(8)
C131	771(3)	-3802(2)	-9300.4(13)	31.0(9)
C46	4598(2)	4485(2)	-7835.9(12)	23.2(8)
C92	2034(2)	-346(2)	-7511.3(12)	26.8(8)
C103	-1018(2)	-1675.8(19)	-3821.6(12)	21.1(7)
C49	3818(3)	4987(2)	-9205.5(14)	32.4(9)
C67	5706(2)	1699(2)	-4743.6(11)	23.9(8)
C18	4355(2)	1406(2)	-7391.6(12)	23.7(8)
C44	4428(2)	3257(2)	-8064.3(12)	22.7(7)
C53	4775(2)	4101(2)	-9375.0(11)	23.3(7)
C80	2097(2)	-2380(2)	-4931.9(11)	21.2(7)
C105	-125(2)	-2139(2)	-4357.9(11)	22.1(7)
C126	849(2)	-2628(2)	-8048.2(12)	26.4(8)
C100	-247(2)	-1460(2)	-5416.4(10)	19.3(7)
C7	-1417(2)	-1718(2)	-6558.6(12)	24.0(8)
C96	1080(2)	-985.2(19)	-6303.5(11)	18.2(7)
C123	450(2)	-2628(2)	-7254.6(11)	24.8(8)

C66	4938(2)	1871.8(19)	-4781.6(10)	19.9(7)
C107	-1334(2)	-1676(2)	-4593.4(12)	23.5(8)
C43	4320(2)	3489(2)	-8490.2(12)	22.4(7)
C85	1877(2)	-1018.2(19)	-6345.4(11)	17.6(7)
C78	1434(2)	-2963(2)	-5525.9(12)	22.4(7)
C128	477(2)	-3754(2)	-7730.1(12)	30.5(9)
C134	1650(3)	-5050(3)	-9320.6(15)	36.9(10)
C125	981(2)	-2974(2)	-8444.9(12)	27.7(8)
C116	2835(2)	-1869(2)	-4340.0(12)	26.8(8)
C17	4436(2)	1477(2)	-6954.1(12)	21.1(7)
C120	1803(2)	-2813(2)	-6975.7(12)	24.9(8)
C41	4714(2)	3474(2)	-7266.2(11)	22.9(7)
C83	3061(2)	-625(2)	-5316.0(12)	24.8(8)
C113	-1264(2)	-1968(2)	-2607.6(12)	28.4(8)
C72	6422(3)	196(3)	-2873.5(14)	36.6(10)
C99	333(2)	-967(2)	-5194.6(12)	24.5(8)
C73	5964(2)	797(3)	-2829.0(12)	33.1(10)
C36	3297(2)	3168(2)	-6490.8(11)	18.5(7)
C19	3629(2)	1475(2)	-7584.5(12)	25.1(8)
C104	-322(2)	-1985(2)	-3926.9(11)	22.6(7)
C141	5082(2)	4308(2)	-5996.2(11)	25.6(8)
C68	5995(2)	1379(2)	-4367.3(12)	23.8(8)
C21	2258(2)	1707(2)	-7525.7(13)	28.4(9)
C82	2801(2)	-1250(2)	-5039.4(12)	22.0(7)
C132	936(3)	-4140(3)	-9697.3(13)	35.3(10)
C112	-1535(2)	-1308(3)	-2470.8(12)	30.1(9)
C121	952(2)	-2948(2)	-6897.3(11)	22.0(7)
C106	-635(2)	-1992(2)	-4698.9(11)	21.8(7)
C87	3011(2)	-757(2)	-6823.4(12)	22.5(7)
C26	1605(2)	1347(2)	-5748.0(14)	27.1(8)
C52	4748(2)	4297(2)	-9814.6(12)	26.1(8)
C22	1552(2)	1846(2)	-7267.1(14)	32.0(9)
C24	2094(2)	1007(2)	-6479.4(12)	23.5(8)
C45	4587(2)	3751(2)	-7729.4(11)	21.0(7)
C93	1712(2)	-592(2)	-7112.2(11)	20.9(7)

C135	1472(3)	-4720(2)	-8925.7(13)	30.8(9)
C30	1434(3)	1454(2)	-4965.9(15)	37.3(11)
C143	5255(3)	5115(2)	-6009.2(17)	43.2(11)
C71	6599(3)	-74(3)	-3282.9(15)	35.8(10)
C70	6308(2)	266(2)	-3653.6(12)	27.0(8)
C50	3787(3)	5177(3)	-9647.3(14)	38.1(10)
C129	629(2)	-4108(2)	-8122.0(12)	28.5(9)
C56	3548(3)	1386(2)	-8045.1(12)	33.2(10)
C51	4251(3)	4837(3)	-9948.8(13)	34.0(10)
C47	4484(2)	4723(2)	-8261.7(12)	23.1(7)
C54	2208(3)	1612(2)	-7975.4(14)	37.0(10)
C55	2853(3)	1456(3)	-8233.6(13)	41.2(11)
C133	1378(3)	-4755(3)	-9707.8(14)	36.4(10)
C91	2805(3)	-297(3)	-7558.7(13)	34.6(10)
C124	874(2)	-3722(2)	-8487.3(12)	23.4(8)
C111	-1653(3)	-754(2)	-2774.5(14)	31.7(9)
C13	6962(2)	1882(3)	-6132.3(16)	39.1(10)
C25	1410(2)	977(2)	-6173.6(14)	28.0(8)
C9	-2232(2)	-1454(3)	-6625.8(14)	32.8(9)
C139	-696(3)	-4550(2)	-6339.7(16)	36.4(10)
C4	2915(3)	-972(3)	-8813(2)	61.1(15)

Table S12. Anisotropic Displacement Parameters (Å^2×10^3) for Cu/L7. The Anisotropic displacement factor exponent takes the form: -2 π^2[h^{2}a*^{2}U$_{11}$+2hka*b*U$_{12}$+⋯].

Atom	U_{11}	U_{22}	U_{33}	U_{23}	U_{13}	U_{12}
Cu1	14.1(2)	24.1(3)	18.9(2)	1.5(2)	-1.20(19)	-2.1(2)
Cu2	16.0(2)	20.6(2)	17.5(2)	1.5(2)	-1.52(18)	-1.5(2)
Cl3	37.7(6)	77.9(10)	73.7(9)	-24.5(8)	5.2(6)	-2.1(7)
Cl5	63.0(10)	59.1(9)	111.8(14)	0.1(9)	-1.8(9)	25.9(8)
O6	16.8(11)	32.3(14)	23.7(11)	2.9(12)	-1.9(10)	-0.9(11)
O10	18.1(12)	28.1(14)	28.0(12)	-0.3(11)	-4.1(10)	-4.6(12)
O61	22.3(13)	18.5(13)	22.1(12)	3.3(10)	-6.5(10)	-3.6(11)
O119	14.8(11)	27.5(14)	24.6(12)	-2.4(10)	-3.4(9)	0.1(11)
O98	20.0(12)	20.6(13)	22.6(11)	-0.3(10)	2.3(10)	-3.4(11)
O37	15.8(12)	26.9(14)	27.2(12)	9.1(11)	0.1(10)	3.1(11)

O136	27.5(14)	40.7(17)	26.6(12)	4.8(12)	-1.2(11)	-7.7(14)
O138	20.5(13)	30.6(15)	26.9(12)	1.3(11)	-4.0(10)	-1.2(12)
O8	24.5(14)	45.5(18)	28.7(13)	6.8(13)	2.1(11)	-2.4(14)
N122	18.2(14)	21.3(15)	19.1(13)	0.3(12)	-1.5(11)	-4.4(13)
C114	20.0(16)	28(2)	24.6(16)	-2.5(16)	-3.0(13)	3.1(17)
N40	16.8(14)	18.9(15)	16.8(12)	1.6(11)	1.1(11)	-2.6(13)
N58	16.3(13)	18.7(14)	15.1(12)	1.3(11)	-2.3(10)	-0.1(13)
O142	22.0(12)	25.9(13)	28.0(13)	3.9(11)	-0.7(10)	-3.8(11)
N101	15.7(14)	22.5(15)	17.3(12)	4.4(11)	0.4(11)	0.2(13)
O140	39.8(17)	40.0(17)	23.2(12)	-0.3(12)	2.9(12)	-6.8(15)
C20	24.3(18)	17.1(17)	25.6(17)	2.8(14)	-8.1(15)	-0.6(16)
C118	14.8(14)	15.4(16)	25.2(16)	1.8(13)	1.1(13)	0.1(14)
C48	19.7(17)	20.9(18)	23.1(16)	2.3(14)	-1.5(14)	-4.1(15)
C14	13.8(16)	20.2(18)	25.0(16)	2.8(14)	-2.4(13)	1.5(14)
C109	16.4(16)	22.2(19)	24.6(17)	0.5(14)	-1.6(13)	-1.2(15)
C127	14.5(15)	33(2)	21.9(16)	0.2(16)	-2.5(13)	-0.3(16)
O12	26.1(14)	79(3)	26.6(14)	-9.3(15)	1.2(11)	-7.4(17)
C84	18.3(17)	19.3(17)	23.1(16)	-1.0(14)	0.0(14)	-2.1(15)
C16	18.8(16)	15.4(17)	17.8(15)	-0.8(13)	-1.6(13)	-2.1(14)
C34	17.6(16)	21.4(18)	25.6(17)	-1.2(14)	1.6(14)	0.8(15)
C117	26.4(18)	38(2)	18.2(16)	7.0(16)	4.1(14)	8.9(19)
C88	16.3(16)	25.1(19)	23.7(16)	1.8(14)	1.8(13)	-0.6(16)
C27	16.8(16)	18.6(18)	30.6(18)	2.9(14)	3.2(14)	6.4(15)
C90	22.7(18)	36(2)	26.1(18)	2.1(16)	4.4(15)	-5.3(18)
C79	19.5(17)	28(2)	27.3(17)	10.1(16)	4.7(14)	2.9(17)
C60	26.3(18)	22.5(18)	18.9(15)	4.3(14)	-6.6(14)	-5.9(16)
C76	11.4(15)	17.9(17)	23.2(16)	2.4(13)	0.1(12)	1.7(14)
C77	12.1(14)	21.6(18)	23.3(16)	5.7(14)	-1.2(12)	0.8(15)
C59	20.1(16)	19.5(17)	14.3(14)	2.9(12)	-3.5(12)	3.7(15)
C29	28(2)	22(2)	49(2)	6.7(18)	15.7(19)	3.4(18)
C63	26.6(19)	19.2(18)	18.4(15)	0.5(13)	-3.2(14)	-2.6(16)
C64	24.5(18)	26.0(19)	19.8(15)	0.8(14)	2.9(14)	1.3(17)
C102	24.3(18)	22.3(19)	23.7(16)	0.9(14)	4.5(13)	-4.8(16)
C15	18.3(17)	13.1(16)	25.1(17)	2.1(13)	-4.6(14)	0.3(14)
C65	22.3(18)	25.5(19)	21.9(16)	-1.6(14)	-2.8(14)	3.6(17)

C137	12.2(15)	27.3(19)	30.1(17)	-1.3(16)	-0.7(14)	2.2(14)
C38	19.2(17)	31(2)	25.7(17)	11.9(16)	2.7(14)	1.8(17)
C130	21.0(17)	29(2)	24.3(17)	-1.3(15)	-0.7(14)	-8.4(17)
C33	24.7(18)	28(2)	23.6(16)	-2.2(15)	4.0(14)	5.2(18)
C89	14.8(15)	22.7(18)	22.4(16)	1.7(14)	-1.2(13)	0.6(15)
C81	12.3(15)	25.3(19)	21.7(16)	3.9(14)	0.9(12)	5.0(14)
C110	25.3(19)	20.9(19)	35(2)	-3.3(16)	0.7(16)	2.1(17)
C69	18.0(16)	23.7(19)	22.1(16)	3.3(14)	-2.4(13)	-6.3(16)
C62	26.3(18)	17.1(18)	20.1(15)	-0.3(13)	-5.1(13)	0.1(15)
C94	23.5(18)	21.1(19)	22.4(16)	3.1(14)	-5.1(14)	1.7(16)
C108	14.9(16)	23.0(19)	28.1(17)	2.2(15)	2.7(14)	-1.4(15)
C31	43(2)	30(2)	29.3(19)	2.8(17)	17.3(17)	8(2)
C28	12.3(14)	21.5(18)	23.7(16)	1.7(13)	0.6(12)	6.0(14)
C97	14.0(15)	22.2(18)	20.8(15)	3.2(14)	-2.3(13)	3.3(15)
C115	23.9(18)	29(2)	24.7(17)	-3.4(15)	-5.2(15)	3.9(17)
C32	26.9(19)	25(2)	26.2(17)	3.1(15)	8.0(15)	7.5(17)
C57	13.2(14)	17.9(16)	21.3(15)	0.4(14)	-1.8(13)	2.5(13)
C23	16.4(16)	21.2(19)	34.6(19)	5.1(15)	-6.1(14)	1.4(15)
C75	15.5(16)	18.0(17)	17.4(15)	2.1(13)	-2.2(12)	-2.9(14)
C35	13.8(15)	20.7(18)	22.5(16)	1.6(13)	3.6(12)	2.0(14)
C11	19.8(16)	35(2)	24.1(16)	-1.8(16)	-0.6(14)	-4.7(16)
C86	17.6(16)	16.1(16)	21.4(15)	0.2(13)	0.1(13)	-2.3(15)
C42	16.8(16)	21.9(18)	22.5(16)	0.6(14)	0.2(13)	-1.0(15)
C95	17.0(16)	25.2(19)	25.5(17)	1.5(15)	-2.8(14)	1.1(16)
C39	19.7(17)	18.9(18)	19.0(15)	2.0(13)	2.2(13)	-1.4(15)
C74	25.6(18)	29(2)	22.4(16)	-0.8(15)	-0.5(15)	-5.3(17)
C131	39(2)	27(2)	27.3(18)	2.1(16)	1.5(17)	-3.9(19)
C46	22.4(18)	22.7(19)	24.6(17)	-1.8(14)	1.1(14)	-0.8(16)
C92	34(2)	29(2)	17.1(16)	4.0(14)	-3.2(15)	-2.9(18)
C103	19.8(16)	16.2(17)	27.4(17)	0.4(14)	2.3(14)	-0.8(15)
C49	34(2)	32(2)	31(2)	2.6(17)	1.4(17)	8(2)
C67	22.1(18)	31(2)	18.5(15)	2.0(15)	1.4(14)	-4.1(17)
C18	28.0(19)	19.9(18)	23.3(16)	2.1(14)	2.1(15)	0.9(17)
C44	23.6(18)	20.4(18)	24.0(16)	0.8(14)	3.8(14)	2.1(16)
C53	21.5(17)	24.6(19)	23.8(16)	1.3(14)	-1.3(14)	-3.6(17)

C80	17.0(16)	25.2(19)	21.4(16)	5.4(14)	2.0(12)	5.9(16)
C105	20.5(17)	20.5(17)	25.3(16)	1.1(14)	1.0(14)	1.2(15)
C126	30.1(19)	26.1(19)	23.0(16)	0.8(15)	2.3(14)	7.0(18)
C100	16.4(15)	23.5(18)	18.2(14)	0.5(13)	2.1(13)	1.5(15)
C7	19.9(17)	29(2)	22.8(16)	0.5(15)	-4.3(14)	-2.4(17)
C96	15.3(15)	16.4(16)	22.9(16)	3.9(13)	0.2(13)	-1.0(14)
C123	18.1(16)	32(2)	23.9(16)	-4.3(16)	-1.0(13)	2.8(17)
C66	25.4(18)	17.2(16)	17.1(14)	-1.8(12)	-4.5(13)	-1.2(15)
C107	16.9(16)	28(2)	25.6(17)	4.3(15)	-3.0(14)	-4.8(16)
C43	22.1(17)	23.1(19)	22.1(16)	-1.3(14)	0.9(14)	-0.6(16)
C85	17.7(16)	14.5(16)	20.5(15)	1.9(13)	-1.2(13)	-0.6(14)
C78	16.6(16)	18.9(18)	31.7(18)	4.6(15)	-1.4(13)	-2.7(16)
C128	31(2)	38(2)	22.1(17)	0.8(16)	1.7(15)	-11.4(19)
C134	38(2)	31(2)	42(2)	-12.9(19)	1.6(19)	1(2)
C125	32(2)	29(2)	21.9(16)	2.9(16)	2.5(15)	2.2(19)
C116	27.3(19)	34(2)	19.3(16)	-1.8(15)	-2.5(14)	9.5(18)
C17	18.6(17)	17.0(17)	27.7(17)	1.3(14)	-0.2(14)	2.5(15)
C120	18.8(17)	32(2)	23.9(17)	-4.1(15)	-1.0(13)	-1.3(17)
C41	21.6(17)	25.7(19)	21.3(15)	4.1(14)	1.4(14)	4.0(16)
C83	19.2(17)	28(2)	27.5(18)	-1.7(16)	-2.9(14)	-2.0(16)
C113	22.0(17)	38(2)	25.3(17)	0.0(17)	-4.1(14)	1.6(19)
C72	31(2)	51(3)	28.7(19)	18.3(19)	-8.4(17)	-2(2)
C99	23.9(18)	27(2)	22.4(16)	0.9(14)	4.3(14)	-4.2(17)
C73	31(2)	50(3)	18.8(16)	5.1(17)	-0.9(15)	-11(2)
C36	19.3(16)	17.4(17)	18.9(15)	0.0(13)	-0.1(13)	1.3(15)
C19	36(2)	18.5(18)	20.4(16)	1.5(14)	-4.0(15)	0.2(17)
C104	20.9(17)	22.5(18)	24.2(16)	1.4(14)	-3.4(13)	1.4(16)
C141	24.3(18)	29(2)	23.4(16)	1.2(14)	-6.9(15)	0.2(17)
C68	18.3(17)	30(2)	22.7(16)	-0.9(15)	-2.8(14)	-0.8(16)
C21	33(2)	22.2(19)	30.5(19)	3.0(15)	-15.7(17)	-1.6(18)
C82	17.2(16)	24.2(19)	24.6(17)	-0.2(14)	-1.2(14)	4.8(16)
C132	50(3)	35(2)	20.6(17)	1.2(17)	2.0(17)	-12(2)
C112	22.4(18)	45(3)	22.4(17)	-8.6(17)	-3.0(14)	-1.0(19)
C121	18.3(16)	25.4(19)	22.4(16)	-4.2(15)	-0.3(13)	-0.5(17)
C106	21.8(17)	20.6(18)	23.0(16)	1.0(14)	2.0(14)	-4.5(16)

C87	22.1(18)	22.2(18)	23.3(16)	1.2(14)	0.8(14)	-1.6(16)
C26	19.3(17)	21.1(19)	41(2)	5.9(16)	6.4(16)	3.8(16)
C52	25.7(19)	29(2)	23.7(16)	-3.8(15)	-0.5(15)	-5.9(18)
C22	25.1(19)	31(2)	40(2)	7.0(18)	-15.9(17)	-2.7(18)
C24	19.1(17)	19.9(18)	31.6(18)	3.0(15)	-4.4(15)	-1.9(16)
C45	19.0(16)	24.6(19)	19.4(15)	1.6(14)	2.5(13)	2.7(15)
C93	25.0(18)	17.3(17)	20.5(16)	1.9(13)	-0.3(14)	0.6(16)
C135	34(2)	31(2)	27.8(18)	-5.9(16)	-7.6(16)	-2.4(19)
C30	44(3)	30(2)	37(2)	8.7(18)	22(2)	5(2)
C143	52(3)	24(2)	54(3)	-3.3(19)	-2(2)	-4(2)
C71	28(2)	35(2)	44(2)	13(2)	-7.6(18)	1(2)
C70	26.6(19)	29(2)	25.2(17)	2.9(15)	-2.7(15)	1.1(18)
C50	41(2)	40(3)	34(2)	13.3(19)	-3.5(19)	10(2)
C129	34(2)	30(2)	22.1(17)	-0.3(15)	-0.9(15)	-9.6(19)
C56	52(3)	28(2)	19.9(17)	2.2(15)	-1.9(18)	4(2)
C51	41(2)	40(3)	21.6(17)	7.3(17)	-8.6(17)	-8(2)
C47	25.7(18)	17.2(17)	26.4(17)	0.4(14)	1.6(14)	0.1(16)
C54	44(3)	33(2)	33(2)	1.9(18)	-21.1(19)	0(2)
C55	64(3)	41(3)	18.3(17)	0.0(17)	-14(2)	3(3)
C133	42(2)	38(2)	28.9(19)	-13.2(18)	9.1(18)	-11(2)
C91	38(2)	43(3)	22.7(17)	6.8(17)	5.2(17)	-10(2)
C124	19.2(17)	28(2)	22.8(16)	0.3(15)	-2.5(14)	-0.2(16)
C111	31(2)	28(2)	36(2)	-10.2(17)	1.2(17)	2.0(19)
C13	24(2)	43(3)	50(3)	-6(2)	1.2(18)	4(2)
C25	16.0(16)	22.0(19)	46(2)	7.5(17)	1.9(16)	-2.9(15)
C9	21.9(19)	38(2)	38(2)	3.3(18)	-6.0(17)	2.8(19)
C139	28(2)	23(2)	58(3)	-6.1(19)	-2.1(19)	-2.3(18)
C4	37(3)	52(3)	94(5)	-1(3)	-2(3)	10(3)

Table S13. Bond Lengths for Cu/L7.

Atom	Atom	Length/Å	Atom	Atom	Length/Å
Cu1	O6	1.968(3)	C102	C106	1.514(5)
Cu1	O138	1.946(3)	C65	C66	1.392(5)
Cu1	N122	2.016(3)	C137	C139	1.512(6)
Cu1	N101	1.987(3)	C38	C39	1.531(5)

Cu2	O10	1.981(3)	C130	C131	1.394(6)
Cu2	N40	2.024(3)	C130	C135	1.383(6)
Cu2	N58	2.016(3)	C130	C124	1.490(5)
Cu2	O142	1.974(3)	C33	C32	1.409(6)
Cl3	C4	1.742(6)	C89	C75	1.548(5)
Cl5	C4	1.763(6)	C81	C80	1.425(5)
O6	C7	1.276(5)	C81	C82	1.424(5)
O10	C11	1.278(5)	C110	C111	1.393(6)
O61	C60	1.468(4)	C69	C74	1.407(5)
O61	C57	1.349(4)	C69	C70	1.390(6)
O119	C118	1.352(4)	C62	C66	1.516(5)
O119	C120	1.460(4)	C94	C95	1.363(5)
O98	C97	1.350(4)	C94	C93	1.405(5)
O98	C99	1.460(4)	C108	C103	1.400(5)
O37	C38	1.448(4)	C108	C107	1.387(5)
O37	C36	1.337(4)	C31	C32	1.420(5)
O136	C137	1.227(5)	C31	C30	1.364(7)
O138	C137	1.291(5)	C28	C35	1.390(5)
O8	C7	1.241(5)	C97	C96	1.481(5)
N122	C118	1.280(4)	C115	C116	1.399(6)
N122	C121	1.487(4)	C115	C82	1.376(5)
C114	C109	1.403(5)	C23	C22	1.526(6)
C114	C113	1.384(5)	C75	C85	1.531(5)
N40	C39	1.498(4)	C35	C36	1.484(5)
N40	C36	1.282(5)	C11	C13	1.512(6)
N58	C59	1.493(4)	C86	C85	1.441(5)
N58	C57	1.278(5)	C86	C87	1.435(5)
O142	C141	1.282(5)	C86	C93	1.437(5)
N101	C97	1.277(5)	C42	C43	1.389(5)
N101	C100	1.492(4)	C42	C47	1.407(5)
O140	C141	1.227(5)	C95	C96	1.418(5)
C20	C15	1.447(5)	C39	C41	1.524(5)
C20	C19	1.427(6)	C74	C73	1.396(6)
C20	C21	1.427(5)	C131	C132	1.395(6)
C118	C77	1.490(5)	C46	C45	1.391(5)

C48	C42	1.493(5)	C46	C47	1.389(5)
C48	C49	1.386(6)	C92	C93	1.420(5)
C48	C53	1.397(5)	C92	C91	1.364(6)
C14	C15	1.534(5)	C103	C104	1.387(5)
C14	C28	1.532(5)	C49	C50	1.397(6)
C14	C23	1.557(5)	C67	C66	1.390(5)
C14	C24	1.545(5)	C67	C68	1.389(5)
C109	C110	1.397(6)	C18	C17	1.352(5)
C109	C103	1.480(5)	C18	C19	1.410(6)
C127	C126	1.395(5)	C44	C43	1.384(5)
C127	C123	1.517(5)	C44	C45	1.399(5)
C127	C128	1.385(6)	C53	C52	1.393(5)
O12	C11	1.231(5)	C105	C104	1.391(5)
C84	C75	1.554(5)	C105	C106	1.400(5)
C84	C83	1.512(5)	C126	C125	1.390(5)
C16	C15	1.393(5)	C100	C99	1.524(5)
C16	C57	1.487(4)	C7	C9	1.524(5)
C16	C17	1.423(5)	C96	C85	1.406(5)
C34	C33	1.367(5)	C123	C121	1.523(5)
C34	C35	1.414(5)	C107	C106	1.397(5)
C117	C80	1.419(5)	C128	C129	1.391(6)
C117	C116	1.362(6)	C134	C135	1.387(6)
C88	C89	1.517(5)	C134	C133	1.387(7)
C88	C87	1.505(5)	C125	C124	1.398(6)
C27	C28	1.436(5)	C120	C121	1.533(5)
C27	C32	1.432(5)	C41	C45	1.522(5)
C27	C26	1.432(6)	C83	C82	1.501(5)
C90	C87	1.374(5)	C113	C112	1.372(6)
C90	C91	1.400(6)	C72	C73	1.375(7)
C79	C80	1.420(6)	C72	C71	1.383(7)
C79	C78	1.355(5)	C19	C56	1.425(5)
C60	C59	1.531(5)	C141	C143	1.520(6)
C76	C77	1.374(5)	C21	C22	1.492(6)
C76	C81	1.448(5)	C21	C54	1.389(6)
C76	C75	1.530(5)	C132	C133	1.374(7)

C77	C78	1.428(5)	C112	C111	1.396(6)
C59	C62	1.519(5)	C26	C25	1.508(6)
C29	C26	1.369(6)	C52	C51	1.386(6)
C29	C30	1.399(7)	C24	C25	1.522(5)
C63	C64	1.398(5)	C71	C70	1.392(6)
C63	C69	1.483(5)	C50	C51	1.380(7)
C63	C68	1.393(5)	C129	C124	1.393(5)
C64	C65	1.383(5)	C56	C55	1.355(7)
C102	C100	1.524(5)	C54	C55	1.410(7)

Table S14. Bond Angles for Cu/L7.

Atom	Atom	Atom	Angle/°	Atom	Atom	Atom	Angle/°
O6	Cu1	N122	164.46(11)	C85	C75	C89	110.0(3)
O6	Cu1	N101	88.84(12)	C34	C35	C36	115.0(3)
O138	Cu1	O6	91.68(12)	C28	C35	C34	122.1(3)
O138	Cu1	N122	88.99(12)	C28	C35	C36	122.9(3)
O138	Cu1	N101	165.09(11)	O10	C11	C13	116.7(3)
N101	Cu1	N122	94.48(12)	O12	C11	O10	121.9(4)
O10	Cu2	N40	172.09(11)	O12	C11	C13	121.4(4)
O10	Cu2	N58	88.64(11)	C87	C86	C85	122.0(3)
N58	Cu2	N40	94.30(12)	C87	C86	C93	118.1(3)
O142	Cu2	O10	91.60(11)	C93	C86	C85	119.8(3)
O142	Cu2	N40	86.61(12)	C43	C42	C48	120.2(3)
O142	Cu2	N58	171.26(11)	C43	C42	C47	118.1(3)
C7	O6	Cu1	105.2(2)	C47	C42	C48	121.6(3)
C11	O10	Cu2	104.1(2)	C94	C95	C96	120.5(3)
C57	O61	C60	106.0(3)	N40	C39	C38	101.4(3)
C118	O119	C120	105.7(3)	N40	C39	C41	113.3(3)
C97	O98	C99	105.3(3)	C41	C39	C38	113.2(3)
C36	O37	C38	105.7(3)	C73	C74	C69	120.0(4)
C137	O138	Cu1	107.5(2)	C130	C131	C132	120.3(4)
C118	N122	Cu1	132.4(2)	C47	C46	C45	121.7(4)
C118	N122	C121	107.4(3)	C91	C92	C93	120.5(4)
C121	N122	Cu1	120.3(2)	C108	C103	C109	122.4(3)
C113	C114	C109	121.1(4)	C104	C103	C109	119.6(3)

C39	N40	Cu2	122.0(2)	C104	C103	C108	118.0(3)
C36	N40	Cu2	131.1(2)	C48	C49	C50	120.0(4)
C36	N40	C39	106.9(3)	C68	C67	C66	121.4(3)
C59	N58	Cu2	121.5(2)	C17	C18	C19	120.0(4)
C57	N58	Cu2	130.4(2)	C43	C44	C45	121.0(4)
C57	N58	C59	108.0(3)	C52	C53	C48	120.8(4)
C141	O142	Cu2	103.1(2)	C117	C80	C79	120.7(4)
C97	N101	Cu1	130.2(2)	C117	C80	C81	119.6(4)
C97	N101	C100	107.7(3)	C79	C80	C81	119.6(3)
C100	N101	Cu1	122.2(2)	C104	C105	C106	120.5(3)
C19	C20	C15	119.9(3)	C125	C126	C127	121.0(4)
C19	C20	C21	118.2(3)	N101	C100	C102	112.7(3)
C21	C20	C15	121.9(4)	N101	C100	C99	101.9(3)
O119	C118	C77	114.4(3)	C99	C100	C102	114.1(3)
N122	C118	O119	116.6(3)	O6	C7	C9	116.4(3)
N122	C118	C77	128.5(3)	O8	C7	O6	122.9(4)
C49	C48	C42	122.1(3)	O8	C7	C9	120.7(3)
C49	C48	C53	119.1(3)	C95	C96	C97	113.8(3)
C53	C48	C42	118.8(3)	C85	C96	C97	124.7(3)
C15	C14	C23	111.1(3)	C85	C96	C95	121.5(3)
C15	C14	C24	103.9(3)	C127	C123	C121	110.4(3)
C28	C14	C15	114.0(3)	C65	C66	C62	122.7(3)
C28	C14	C23	108.2(3)	C67	C66	C65	117.8(3)
C28	C14	C24	110.7(3)	C67	C66	C62	119.5(3)
C24	C14	C23	108.8(3)	C108	C107	C106	121.1(3)
C114	C109	C103	120.2(3)	C44	C43	C42	121.3(4)
C110	C109	C114	117.9(3)	C86	C85	C75	119.2(3)
C110	C109	C103	121.9(3)	C96	C85	C75	123.2(3)
C126	C127	C123	120.2(4)	C96	C85	C86	117.5(3)
C128	C127	C126	118.1(4)	C79	C78	C77	120.4(4)
C128	C127	C123	121.7(4)	C127	C128	C129	121.4(4)
C83	C84	C75	112.3(3)	C135	C134	C133	119.6(4)
C15	C16	C57	126.4(3)	C126	C125	C124	120.7(4)
C15	C16	C17	121.1(3)	C117	C116	C115	119.8(3)
C17	C16	C57	112.4(3)	C18	C17	C16	121.5(4)

C33	C34	C35	120.4(4)	O119	C120	C121	103.0(3)
C116	C117	C80	120.8(4)	C45	C41	C39	111.1(3)
C87	C88	C89	111.3(3)	C82	C83	C84	111.2(3)
C32	C27	C28	119.8(3)	C112	C113	C114	120.5(4)
C26	C27	C28	122.1(4)	C73	C72	C71	120.7(4)
C26	C27	C32	118.0(3)	O98	C99	C100	104.3(3)
C87	C90	C91	122.3(4)	C72	C73	C74	120.1(4)
C78	C79	C80	120.5(3)	O37	C36	C35	115.4(3)
O61	C60	C59	103.9(3)	N40	C36	O37	117.1(3)
C77	C76	C81	118.2(3)	N40	C36	C35	127.5(3)
C77	C76	C75	124.1(3)	C18	C19	C20	119.9(3)
C81	C76	C75	117.6(3)	C18	C19	C56	119.5(4)
C76	C77	C118	124.9(3)	C56	C19	C20	120.6(4)
C76	C77	C78	121.8(3)	C103	C104	C105	121.5(3)
C78	C77	C118	113.1(3)	O142	C141	C143	115.5(4)
N58	C59	C60	102.3(3)	O140	C141	O142	123.4(4)
N58	C59	C62	114.0(3)	O140	C141	C143	121.1(4)
C62	C59	C60	114.2(3)	C67	C68	C63	120.8(4)
C26	C29	C30	121.8(4)	C20	C21	C22	121.3(3)
C64	C63	C69	122.1(3)	C54	C21	C20	119.1(4)
C68	C63	C64	117.8(3)	C54	C21	C22	119.5(4)
C68	C63	C69	120.1(3)	C81	C82	C83	121.2(3)
C65	C64	C63	121.2(3)	C115	C82	C81	119.8(4)
C106	C102	C100	110.6(3)	C115	C82	C83	118.9(4)
C20	C15	C14	118.5(3)	C133	C132	C131	120.4(4)
C16	C15	C20	117.5(3)	C113	C112	C111	119.8(4)
C16	C15	C14	123.7(3)	N122	C121	C123	114.4(3)
C64	C65	C66	121.1(4)	N122	C121	C120	102.0(3)
O136	C137	O138	122.6(4)	C123	C121	C120	112.8(3)
O136	C137	C139	122.4(4)	C105	C106	C102	120.0(3)
O138	C137	C139	115.0(3)	C107	C106	C102	121.9(3)
O37	C38	C39	103.3(3)	C107	C106	C105	118.0(3)
C131	C130	C124	120.0(4)	C90	C87	C88	120.8(4)
C135	C130	C131	118.5(4)	C90	C87	C86	119.3(4)
C135	C130	C124	121.5(4)	C86	C87	C88	119.9(3)

C34	C33	C32	120.4(4)	C27	C26	C25	119.8(3)
C88	C89	C75	112.2(3)	C29	C26	C27	120.1(4)
C80	C81	C76	119.4(3)	C29	C26	C25	120.1(4)
C82	C81	C76	122.3(3)	C51	C52	C53	119.6(4)
C82	C81	C80	118.2(3)	C21	C22	C23	112.8(3)
C111	C110	C109	120.9(4)	C25	C24	C14	112.0(3)
C74	C69	C63	120.7(4)	C46	C45	C44	117.7(3)
C70	C69	C63	120.7(3)	C46	C45	C41	122.8(3)
C70	C69	C74	118.7(4)	C44	C45	C41	119.5(3)
C66	C62	C59	111.3(3)	C94	C93	C86	119.5(3)
C95	C94	C93	121.0(3)	C94	C93	C92	120.7(3)
C107	C108	C103	120.8(3)	C92	C93	C86	119.7(4)
C30	C31	C32	121.2(4)	C130	C135	C134	121.4(4)
C27	C28	C14	119.2(3)	C31	C30	C29	119.8(4)
C35	C28	C14	123.2(3)	C72	C71	C70	119.5(4)
C35	C28	C27	117.6(3)	C69	C70	C71	121.0(4)
O98	C97	C96	116.8(3)	C51	C50	C49	120.6(4)
N101	C97	O98	117.1(3)	C128	C129	C124	120.7(4)
N101	C97	C96	125.7(3)	C55	C56	C19	119.9(4)
C82	C115	C116	121.7(4)	C50	C51	C52	120.0(4)
C33	C32	C27	119.6(3)	C46	C47	C42	120.1(4)
C33	C32	C31	121.3(4)	C21	C54	C55	121.9(4)
C31	C32	C27	119.1(4)	C56	C55	C54	120.3(4)
O61	C57	C16	115.7(3)	C132	C133	C134	119.8(4)
N58	C57	O61	117.0(3)	C92	C91	C90	120.0(4)
N58	C57	C16	126.4(3)	C125	C124	C130	120.5(4)
C22	C23	C14	112.3(3)	C129	C124	C130	121.4(4)
C76	C75	C84	110.5(3)	C129	C124	C125	118.1(4)
C76	C75	C89	106.0(3)	C110	C111	C112	119.8(4)
C76	C75	C85	114.4(3)	C26	C25	C24	109.6(3)
C89	C75	C84	109.9(3)	Cl3	C4	Cl5	113.4(3)
C85	C75	C84	106.1(3)				

Table S15. Torsion Angles for Cu/L7.

A	B	C	D	Angle/°	A	B	C	D	Angle/°

Cu1	O6	C7	O8	-4.5(5)
Cu1	O6	C7	C9	173.8(3)
Cu1	O138	C137	O136	1.7(4)
Cu1	O138	C137	C139	-178.9(3)
Cu1	N122	C118	O119	-175.2(2)
Cu1	N122	C118	C77	13.8(6)
Cu1	N122	C121	C123	40.9(4)
Cu1	N122	C121	C120	163.0(2)
Cu1	N101	C97	O98	-175.7(2)
Cu1	N101	C97	C96	12.3(5)
Cu1	N101	C100	C102	42.3(4)
Cu1	N101	C100	C99	165.0(2)
Cu2	O10	C11	O12	4.7(5)
Cu2	O10	C11	C13	-175.0(3)
Cu2	N40	C39	C38	165.6(2)
Cu2	N40	C39	C41	44.0(4)
Cu2	N40	C36	O37	-179.2(2)
Cu2	N40	C36	C35	5.1(6)
Cu2	N58	C59	C60	170.6(2)
Cu2	N58	C59	C62	46.7(4)
Cu2	N58	C57	O61	-179.6(2)
Cu2	N58	C57	C16	11.1(5)
Cu2	O142	C141	O140	4.1(4)
Cu2	O142	C141	C143	-175.5(3)
O61	C60	C59	N58	16.0(4)
O61	C60	C59	C62	139.7(3)
O119	C118	C77	C76	66.7(5)
O119	C118	C77	C78	-108.6(4)
O119	C120	C121	N122	22.3(4)
O119	C120	C121	C123	145.5(3)
O98	C97	C96	C95	-116.4(4)
O98	C97	C96	C85	63.0(5)
O37	C38	C39	N40	22.6(4)
O37	C38	C39	C41	144.3(3)
N122	C118	C77	C76	-122.1(4)
C57	N58	C59	C62	-136.5(3)
C57	C16	C15	C20	-174.1(3)
C57	C16	C15	C14	-0.9(6)
C57	C16	C17	C18	173.7(3)
C23	C14	C15	C20	-30.3(5)
C23	C14	C15	C16	156.5(3)
C23	C14	C28	C27	98.0(4)
C23	C14	C28	C35	-81.1(4)
C23	C14	C24	C25	-65.6(4)
C75	C84	C83	C82	-51.8(4)
C75	C76	C77	C118	3.0(6)
C75	C76	C77	C78	177.9(3)
C75	C76	C81	C80	-175.8(3)
C75	C76	C81	C82	0.6(5)
C35	C34	C33	C32	-1.5(6)
C42	C48	C49	C50	-179.2(4)
C42	C48	C53	C52	179.8(3)
C95	C94	C93	C86	2.3(6)
C95	C94	C93	C92	-175.3(4)
C95	C96	C85	C75	180.0(3)
C95	C96	C85	C86	4.3(5)
C39	N40	C36	O37	2.8(4)
C39	N40	C36	C35	-172.9(3)
C39	C41	C45	C46	64.7(5)
C39	C41	C45	C44	-113.4(4)
C74	C69	C70	C71	0.4(6)
C131	C130	C135	C134	1.2(7)
C131	C130	C124	C125	-47.0(6)
C131	C130	C124	C129	134.7(4)
C131	C132	C133	C134	1.1(7)
C103	C109	C110	C111	-178.2(4)
C103	C108	C107	C106	-0.5(6)
C49	C48	C42	C43	129.5(4)
C49	C48	C42	C47	-53.2(6)
C49	C48	C53	C52	0.9(6)

N122	C118	C77	C78	62.6(5)	C49	C50	C51	C52	0.7(7)
C114	C109	C110	C111	0.8(6)	C18	C19	C56	C55	-179.7(4)
C114	C109	C103	C108	130.8(4)	C53	C48	C42	C43	-49.4(5)
C114	C109	C103	C104	-47.7(5)	C53	C48	C42	C47	127.9(4)
C114	C113	C112	C111	0.4(6)	C53	C48	C49	C50	-0.3(6)
N40	C39	C41	C45	159.4(3)	C53	C52	C51	C50	-0.1(7)
N58	C59	C62	C66	-173.6(3)	C80	C117	C116	C115	2.8(6)
N101	C97	C96	C95	55.6(5)	C80	C79	C78	C77	0.3(6)
N101	C97	C96	C85	-125.0(4)	C80	C81	C82	C115	2.0(5)
N101	C100	C99	O98	18.9(4)	C80	C81	C82	C83	-179.7(3)
C20	C19	C56	C55	0.0(6)	C126	C127	C123	C121	-119.9(4)
C20	C21	C22	C23	23.1(5)	C126	C127	C128	C129	0.9(6)
C20	C21	C54	C55	-0.6(7)	C126	C125	C124	C130	-178.1(4)
C118	O119	C120	C121	-20.1(4)	C126	C125	C124	C129	0.2(6)
C118	N122	C121	C123	-139.5(3)	C100	N101	C97	O98	3.3(4)
C118	N122	C121	C120	-17.4(4)	C100	N101	C97	C96	-168.8(3)
C118	C77	C78	C79	172.5(3)	C100	C102	C106	C105	-107.2(4)
C48	C42	C43	C44	174.5(3)	C100	C102	C106	C107	71.3(5)
C48	C42	C47	C46	-174.0(4)	C123	C127	C126	C125	179.8(3)
C48	C49	C50	C51	-0.5(7)	C123	C127	C128	C129	-177.9(4)
C48	C53	C52	C51	-0.7(6)	C66	C67	C68	C63	-0.1(6)
C14	C28	C35	C34	-177.5(3)	C107	C108	C103	C109	-177.6(4)
C14	C28	C35	C36	2.4(5)	C107	C108	C103	C104	1.0(6)
C14	C23	C22	C21	-48.9(4)	C43	C42	C47	C46	3.4(5)
C14	C24	C25	C26	-57.8(4)	C43	C44	C45	C46	2.8(5)
C109	C114	C113	C112	-0.1(6)	C43	C44	C45	C41	-179.0(3)
C109	C110	C111	C112	-0.6(6)	C85	C86	C87	C88	2.0(6)
C109	C103	C104	C105	178.2(4)	C85	C86	C87	C90	-176.0(4)
C127	C126	C125	C124	-1.5(6)	C85	C86	C93	C94	-0.6(5)
C127	C123	C121	N122	-178.0(3)	C85	C86	C93	C92	177.0(3)
C127	C123	C121	C120	66.0(4)	C78	C79	C80	C117	-175.3(4)
C127	C128	C129	C124	-2.2(7)	C78	C79	C80	C81	2.1(6)
C84	C75	C85	C86	92.1(4)	C128	C127	C126	C125	0.9(6)
C84	C75	C85	C96	-83.5(4)	C128	C127	C123	C121	58.9(5)
C84	C83	C82	C81	22.1(5)	C128	C129	C124	C130	179.9(4)

C84	C83	C82	C115	-159.5(3)
C34	C33	C32	C27	0.0(6)
C34	C33	C32	C31	-177.3(4)
C34	C35	C36	O37	-115.5(4)
C34	C35	C36	N40	60.3(5)
C88	C89	C75	C84	-61.7(4)
C88	C89	C75	C76	178.9(3)
C88	C89	C75	C85	54.7(4)
C27	C28	C35	C34	3.4(5)
C27	C28	C35	C36	-176.6(3)
C27	C26	C25	C24	31.1(5)
C60	O61	C57	N58	7.2(4)
C60	O61	C57	C16	177.6(3)
C60	C59	C62	C66	69.2(4)
C76	C77	C78	C79	-3.0(6)
C76	C81	C80	C117	175.5(3)
C76	C81	C80	C79	-1.9(5)
C76	C81	C82	C115	-174.5(3)
C76	C81	C82	C83	3.9(5)
C76	C75	C85	C86	-145.8(3)
C76	C75	C85	C96	38.6(5)
C77	C76	C81	C80	-0.6(5)
C77	C76	C81	C82	175.8(3)
C77	C76	C75	C84	155.5(3)
C77	C76	C75	C89	-85.5(4)
C77	C76	C75	C85	35.8(5)
C59	N58	C57	O61	3.9(4)
C59	N58	C57	C16	-165.4(3)
C59	C62	C66	C65	-109.6(4)
C59	C62	C66	C67	70.3(4)
C29	C26	C25	C24	-150.1(4)
C63	C64	C65	C66	-1.4(6)
C63	C69	C74	C73	-179.6(4)
C63	C69	C70	C71	-179.3(4)
C64	C63	C69	C74	-47.6(5)
C128	C129	C124	C125	1.6(6)
C116	C117	C80	C79	176.1(4)
C116	C117	C80	C81	-1.3(6)
C116	C115	C82	C81	-0.6(6)
C116	C115	C82	C83	-179.0(4)
C17	C16	C15	C20	2.3(5)
C17	C16	C15	C14	175.5(3)
C17	C16	C57	O61	-105.8(4)
C17	C16	C57	N58	63.6(5)
C17	C18	C19	C20	1.5(6)
C17	C18	C19	C56	-178.9(4)
C120	O119	C118	N122	10.2(4)
C120	O119	C118	C77	-177.6(3)
C83	C84	C75	C76	56.0(4)
C83	C84	C75	C89	-60.6(4)
C83	C84	C75	C85	-179.5(3)
C113	C114	C109	C110	-0.4(6)
C113	C114	C109	C103	178.6(4)
C113	C112	C111	C110	0.0(6)
C72	C71	C70	C69	-0.7(7)
C99	O98	C97	N101	9.7(4)
C99	O98	C97	C96	-177.5(3)
C73	C72	C71	C70	-0.3(7)
C36	O37	C38	C39	-21.9(4)
C36	N40	C39	C38	-16.1(4)
C36	N40	C39	C41	-137.7(3)
C19	C20	C15	C14	-173.2(3)
C19	C20	C15	C16	0.4(5)
C19	C20	C21	C22	177.5(4)
C19	C20	C21	C54	0.2(6)
C19	C18	C17	C16	1.2(6)
C19	C56	C55	C54	-0.4(7)
C104	C105	C106	C102	179.6(3)
C104	C105	C106	C107	1.1(6)
C68	C63	C64	C65	1.1(6)

C64	C63	C69	C70	132.1(4)	C68	C63	C69	C74	133.2(4)
C64	C63	C68	C67	-0.3(6)	C68	C63	C69	C70	-47.1(5)
C64	C65	C66	C62	-179.2(3)	C68	C67	C66	C65	-0.1(6)
C64	C65	C66	C67	0.9(6)	C68	C67	C66	C62	180.0(4)
C102	C100	C99	O98	140.7(3)	C21	C20	C15	C14	4.7(5)
C15	C20	C19	C18	-2.3(6)	C21	C20	C15	C16	178.3(4)
C15	C20	C19	C56	178.1(4)	C21	C20	C19	C18	179.7(4)
C15	C20	C21	C22	-0.5(6)	C21	C20	C19	C56	0.1(6)
C15	C20	C21	C54	-177.7(4)	C21	C54	C55	C56	0.7(7)
C15	C14	C28	C27	-137.8(3)	C82	C81	C80	C117	-1.0(5)
C15	C14	C28	C35	43.1(5)	C82	C81	C80	C79	-178.5(3)
C15	C14	C23	C22	52.3(4)	C82	C115	C116	C117	-1.8(6)
C15	C14	C24	C25	175.9(3)	C121	N122	C118	O119	5.3(4)
C15	C16	C57	O61	70.8(5)	C121	N122	C118	C77	-165.7(4)
C15	C16	C57	N58	-119.8(4)	C106	C102	C100	N101	168.7(3)
C15	C16	C17	C18	-3.2(6)	C106	C102	C100	C99	53.2(4)
C38	O37	C36	N40	12.9(4)	C106	C105	C104	C103	-0.7(6)
C38	O37	C36	C35	-170.8(3)	C87	C88	C89	C75	-54.5(4)
C38	C39	C41	C45	44.6(4)	C87	C90	C91	C92	-0.4(7)
C130	C131	C132	C133	-1.2(7)	C87	C86	C85	C75	-1.1(5)
C33	C34	C35	C28	-0.3(6)	C87	C86	C85	C96	174.7(3)
C33	C34	C35	C36	179.8(3)	C87	C86	C93	C94	-178.0(4)
C89	C88	C87	C90	-156.1(4)	C87	C86	C93	C92	-0.4(5)
C89	C88	C87	C86	25.9(5)	C26	C27	C28	C14	-5.4(5)
C89	C75	C85	C86	-26.7(4)	C26	C27	C28	C35	173.7(3)
C89	C75	C85	C96	157.7(3)	C26	C27	C32	C33	-175.4(4)
C81	C76	C77	C118	-171.9(3)	C26	C27	C32	C31	2.0(6)
C81	C76	C77	C78	3.1(5)	C26	C29	C30	C31	0.6(7)
C81	C76	C75	C84	-29.6(4)	C22	C21	C54	C55	-177.9(4)
C81	C76	C75	C89	89.4(4)	C24	C14	C15	C20	86.5(4)
C81	C76	C75	C85	-149.3(3)	C24	C14	C15	C16	-86.7(4)
C110	C109	C103	C108	-50.2(5)	C24	C14	C28	C27	-21.1(4)
C110	C109	C103	C104	131.3(4)	C24	C14	C28	C35	159.8(3)
C69	C63	C64	C65	-178.1(4)	C24	C14	C23	C22	-61.4(4)
C69	C63	C68	C67	178.9(4)	C45	C46	C47	C42	-0.8(6)

C69	C74	C73	C72	-1.6(6)	C45	C44	C43	C42	-0.2(6)
C94	C95	C96	C97	176.6(4)	C93	C94	C95	C96	-0.6(6)
C94	C95	C96	C85	-2.8(6)	C93	C86	C85	C75	-178.4(3)
C108	C103	C104	C105	-0.4(6)	C93	C86	C85	C96	-2.6(5)
C108	C107	C106	C102	-179.0(4)	C93	C86	C87	C88	179.4(3)
C108	C107	C106	C105	-0.6(6)	C93	C86	C87	C90	1.3(6)
C28	C14	C15	C20	-152.9(3)	C93	C92	C91	C90	1.3(7)
C28	C14	C15	C16	33.9(5)	C135	C130	C131	C132	0.1(6)
C28	C14	C23	C22	178.3(3)	C135	C130	C124	C125	131.7(4)
C28	C14	C24	C25	53.1(4)	C135	C130	C124	C129	-46.5(6)
C28	C27	C32	C33	3.1(5)	C135	C134	C133	C132	0.1(7)
C28	C27	C32	C31	-179.4(4)	C30	C29	C26	C27	-1.3(6)
C28	C27	C26	C29	-178.6(4)	C30	C29	C26	C25	179.9(4)
C28	C27	C26	C25	0.3(6)	C30	C31	C32	C27	-2.8(6)
C28	C35	C36	O37	64.6(5)	C30	C31	C32	C33	174.6(4)
C28	C35	C36	N40	-119.6(4)	C71	C72	C73	C74	1.4(7)
C97	O98	C99	C100	-17.7(4)	C70	C69	C74	C73	0.7(6)
C97	N101	C100	C102	-136.7(3)	C47	C42	C43	C44	-2.9(6)
C97	N101	C100	C99	-14.0(4)	C47	C46	C45	C44	-2.3(6)
C97	C96	C85	C75	0.6(6)	C47	C46	C45	C41	179.6(3)
C97	C96	C85	C86	-175.0(3)	C54	C21	C22	C23	-159.7(4)
C32	C27	C28	C14	176.1(3)	C133	C134	C135	C130	-1.3(7)
C32	C27	C28	C35	-4.8(5)	C91	C90	C87	C88	-179.0(4)
C32	C27	C26	C29	0.0(6)	C91	C90	C87	C86	-1.0(7)
C32	C27	C26	C25	178.8(3)	C91	C92	C93	C94	176.7(4)
C32	C31	C30	C29	1.5(7)	C91	C92	C93	C86	-0.9(6)
C57	O61	C60	C59	-14.4(4)	C124	C130	C131	C132	178.9(4)
C57	N58	C59	C60	-12.6(4)	C124	C130	C135	C134	-177.6(4)

Table S16. Hydrogen Atom Coordinates (Å×10[4]) and Isotropic Displacement Parameters (Å[2]×10[3]) for Cu/L7.

Atom	*x*	*y*	*z*	U(eq)
H114	-913.15	-2547.07	-3133.94	29
H84A	2757.32	-137.96	-5897.08	24
H84B	2038.82	-329.49	-5596.12	24

H34	3382.01	3591.26	-5693.02	26
H117	2196.83	-2771.04	-4303.03	33
H88A	3693.91	-521.79	-6295.47	26
H88B	4012.29	-1179.74	-6584.65	26
H90	3822.14	-468.55	-7255.03	34
H79	1470.18	-3326.78	-4908.85	30
H60A	4312.82	622.06	-5435.37	27
H60B	3656.61	1210.04	-5333.9	27
H59	5176.82	1467.51	-5597.85	22
H29	909.25	738.99	-5375.29	40
H64	4415.5	1307.34	-3814.02	28
H10A	-831.62	-2423.91	-5312.5	28
H10B	45.6	-2471.12	-5169.33	28
H65	3934.64	1818.58	-4448.81	28
H38A	3106.6	4098.82	-7291.9	30
H38B	3277.14	3253.5	-7385.38	30
H33	2872.13	3130.76	-5053.76	31
H89A	3528.09	-1594.21	-5901.31	24
H89B	3092.35	-1964.24	-6303.4	24
H110	-1581.3	-497.03	-3420.9	33
H62A	4971.83	2643.4	-5271.61	25
H62B	4121.55	2397.19	-5152.17	25
H94	593.07	-486.8	-7291.52	27
H108	-2000.54	-1299	-4099.77	26
H31	2061.13	2228.12	-4675.58	41
H115	3368.19	-922.97	-4500.45	31
H23A	1241.2	2194.39	-6647.72	29
H23B	1867.2	2706.56	-6870.25	29
H95	68.32	-798.46	-6627.39	27
H39	4164.19	4241.89	-6878.35	23
H74	5332.83	1541.25	-3167.13	31
H131	474.31	-3371.42	-9296.6	37
H46	4685.96	4831.18	-7611.62	28
H92	1709.75	-213.82	-7747.13	32
H49	3499.25	5226.25	-8999.98	39

H67	6040.08	1802.06	-4979.93	29
H18	4787.41	1309.42	-7569.2	28
H44	4394.24	2754.2	-7998.45	27
H53	5111.28	3727.14	-9283.4	28
H105	358.13	-2345.19	-4421.17	27
H126	936.72	-2121.33	-8023.85	32
H100	-733.72	-1186.74	-5460.76	23
H12A	-92.83	-2677.7	-7171.06	30
H12B	563.6	-2104.99	-7288	30
H107	-1686.87	-1567.23	-4819.88	28
H43	4214.69	3142.27	-8711.97	27
H78	1124.82	-3345.87	-5633.81	27
H128	293.03	-4025.24	-7487.97	37
H134	1956.1	-5474.95	-9325.87	44
H125	1146.72	-2699.12	-8689.92	33
H116	3023.91	-1893.5	-4049.28	32
H17	4929.73	1438.56	-6828.63	25
H12C	2043.72	-3231.5	-7123.98	30
H12D	1884.24	-2371.34	-7153.86	30
H41A	5209.57	3654.66	-7156.19	27
H41B	4733.58	2937.19	-7269.06	27
H83A	3584.23	-718.99	-5422.57	30
H83B	3072.69	-178.32	-5136.2	30
H113	-1186.46	-2345.39	-2400.84	34
H72	6617.85	-36.29	-2620.14	44
H99A	75.28	-568.94	-5034.98	29
H99B	652.41	-1243.93	-4986.72	29
H73	5855.36	983.98	-2546.29	40
H104	29.64	-2093.26	-3699.78	27
H68	6522.38	1266.36	-4350.34	29
H132	742.48	-3943.54	-9962.04	42
H112	-1642.57	-1228.37	-2170.15	36
H121	858.83	-3482.71	-6879.37	26
H52	5067.23	4060.95	-10021.18	31
H22A	1282.14	1380.49	-7219.98	38

H22B	1211.07	2166.81	-7437.42	38
H24A	1959.21	771.81	-6760.15	28
H24B	2520.66	731.46	-6348.23	28
H135	1651.07	-4929.75	-8661.02	37
H30	1195.05	1288.07	-4705.76	45
H14A	5745.66	5207.33	-5867.11	65
H14B	4852.44	5381.2	-5854.95	65
H14C	5276.94	5278.49	-6313.97	65
H71	6917.11	-488.1	-3311.09	43
H70	6434.48	83.97	-3935.01	32
H50	3443.01	5542.95	-9741.19	46
H129	563.99	-4619.06	-8140.81	34
H56	3981.25	1278.1	-8219.78	40
H51	4229.88	4973.52	-10248.06	41
H47	4488.69	5227.84	-8324.76	28
H54	1724.25	1652.66	-8112.93	44
H55	2800.46	1399.02	-8540.93	49
H133	1496.19	-4978.6	-9979.43	44
H91	3015.92	-121.51	-7824.68	41
H111	-1841	-296.27	-2681.01	38
H13D	7366.99	2243.05	-6096.3	59
H13E	7100.19	1544.83	-6366.89	59
H13F	6896.18	1612.95	-5858.48	59
H25A	969.1	1222.19	-6311.37	34
H25B	1268.5	465.55	-6117.65	34
H9A	-2552	-1859.93	-6720.73	49
H9B	-2238.22	-1073.44	-6849.56	49
H9C	-2429.38	-1258.28	-6350.14	49
H13A	-389.77	-4700.34	-6591.77	55
H13B	-1237.06	-4552.62	-6419.93	55
H13C	-612	-4888.2	-6097.02	55
H4A	3144.96	-1336.79	-9010.6	73
H4B	3312.42	-817.55	-8602.91	73

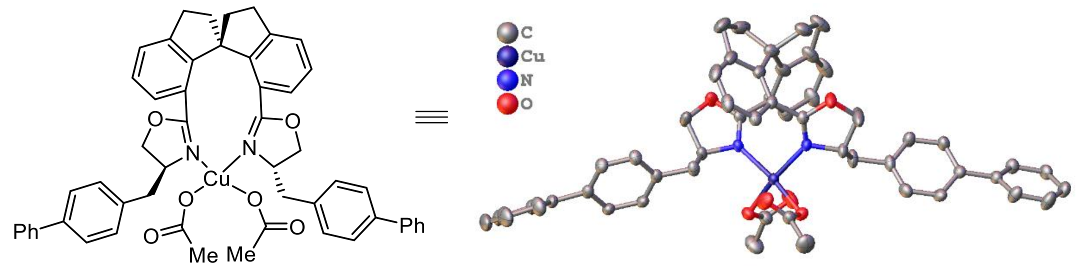

Table S17. Crystal Data and Structure Refinement for Cu/L12.

Identification code	**Cu/L12**
Empirical formula	$C_{54.74}H_{51.48}Cl_{3.48}CuN_2O_6$
Formula weight	1020.097
Temperature/K	173.0(1)
Crystal system	orthorhombic
Space group	$P2_12_12$
a/Å	17.1617(8)
b/Å	18.6752(7)
c/Å	15.6497(7)
α/°	90
β/°	90
γ/°	90
Volume/Å^3	5015.7(4)
Z	4
ρ_{calc}g/cm^3	1.351
μ/mm^{-1}	0.356
F(000)	2123.1
Crystal size/mm^3	0.33 × 0.29 × 0.232
Radiation	Ag Kα (λ = 0.56086)
2Θ range for data collection/°	3.74 to 42.16
Index ranges	$-22 \le h \le 21$, $-23 \le k \le 23$, $-20 \le l \le 18$
Reflections collected	71834
Independent reflections	11082 [R_{int} = 0.0317, R_{sigma} = 0.0206]
Data/restraints/parameters	11082/73/653
Goodness-of-fit on F^2	1.042
Final R indexes [I>=2σ (I)]	R_1 = 0.0391, wR_2 = 0.1083
Final R indexes [all data]	R_1 = 0.0416, wR_2 = 0.1102
Largest diff. peak/hole / e Å^{-3}	0.99/-0.49

Flack parameter 0.004(3)

Table S18. Fractional Atomic Coordinates (×10[4]) and Equivalent Isotropic Displacement Parameters (Å[2]×10[3]) for Cu/L12. U_{eq} is defined as 1/3 of the trace of the orthogonalised U_{IJ} tensor.

Atom	*x*	*y*	*z*	U(eq)
Cu1	2806.38(17)	2617.06(14)	1956.10(16)	23.78(7)
O1	5046.6(12)	3137.8(11)	2865.6(12)	38.3(4)
O2	3968.6(13)	781.5(10)	1096.8(13)	39.4(5)
O11	2376.8(11)	3563.4(10)	2247.9(12)	32.2(4)
O12	2899.3(17)	3727.5(14)	976.5(13)	55.4(6)
O21	1752.6(11)	2275.4(10)	1642.9(11)	32.9(4)
O22	1883.0(14)	2020.6(14)	3010.1(14)	52.8(6)
N1	3803.2(13)	2914.6(11)	2538.9(13)	26.3(4)
N2	3250.4(13)	1748.5(10)	1394.0(13)	25.3(4)
C1	5526.3(17)	1498.7(15)	2095(2)	39.7(6)
C2	5837(2)	864.7(18)	1543(3)	56.2(9)
C3	6133(2)	1198(2)	719(3)	60.5(10)
C4	5652.0(17)	1871.8(17)	630(2)	41.6(7)
C5	5316.3(15)	2044.0(14)	1422.7(18)	31.8(5)
C6	6162.0(19)	1748.9(18)	2746(2)	48.4(8)
C7	6016(2)	1298(2)	3551(3)	60.5(10)
C8	5144(2)	1166.4(16)	3522(2)	46.2(8)
C9	4872.7(17)	1283.3(14)	2696(2)	37.2(6)
C10	4513.5(15)	2891.8(13)	2310.3(16)	27.5(5)
C11	4847.1(14)	2649.0(14)	1485.9(15)	27.2(4)
C12	4701.0(17)	3068.2(14)	762.0(17)	32.6(5)
C13	5060(2)	2890.5(18)	-9.1(18)	42.1(7)
C14	5533.9(19)	2297(2)	-72.5(19)	47.1(7)
C15	4637(2)	3296(3)	3646(2)	70.7(14)
C16	3776.5(16)	3265.6(14)	3388.7(16)	31.0(5)
C17	3296.6(19)	2846.3(16)	4040.7(17)	39.3(6)
C20	3743.3(15)	1284.9(12)	1653.8(17)	28.3(5)

C21	4068.1(16)	1206.0(13)	2528.5(18)	31.9(6)
C22	3570.9(19)	1022.4(14)	3187.1(19)	38.6(6)
C23	3856(2)	888.2(17)	3998(2)	46.1(7)
C24	4644(2)	955.7(18)	4168(2)	54.1(9)
C25	3620(2)	970.7(17)	286.8(19)	43.9(7)
C26	3011.5(15)	1534.1(12)	513.6(15)	26.8(5)
C27	2986.2(17)	2149.7(13)	-120.7(16)	31.4(5)
C31	3297.5(17)	3204.5(15)	4902.9(16)	33.0(6)
C32	3710.5(19)	2909.1(15)	5580.7(18)	37.5(6)
C33	3713.7(18)	3235.8(14)	6379.7(17)	34.6(6)
C34	3306.1(15)	3872.1(13)	6520.1(16)	27.7(5)
C35	2908.5(16)	4169.6(15)	5826.7(16)	34.2(5)
C36	2916.5(17)	3837.6(16)	5033.8(17)	37.0(6)
C37	3275.2(15)	4208.1(14)	7382.1(16)	29.3(5)
C38	3286.1(17)	3793.5(14)	8120.4(18)	35.1(5)
C39	3234.1(18)	4099.6(16)	8921.5(17)	36.7(6)
C40	3161(2)	4830.0(17)	8996.8(18)	42.0(7)
C41	3148(2)	5253.9(17)	8268(2)	51.3(8)
C42	3212(2)	4949.6(16)	7467.2(18)	41.4(7)
C43	2724.1(17)	1884.7(12)	-986.7(15)	28.7(5)
C44	1957.6(16)	1668.4(14)	-1110.1(17)	31.5(5)
C45	1700.2(16)	1430.7(14)	-1905.1(18)	33.4(5)
C46	2198.7(18)	1421.8(13)	-2605.6(15)	30.8(5)
C47	2968.6(17)	1623.3(14)	-2481.4(16)	33.6(6)
C48	3231.2(17)	1845.0(14)	-1681.8(17)	33.2(5)
C49	1899.8(17)	1210.4(16)	-3461.4(17)	35.8(6)
C50	1445.7(19)	605.6(17)	-3572(2)	41.8(7)
C51	1139(2)	431(2)	-4373(2)	54.7(9)
C52	1291(2)	856(3)	-5059(2)	64.3(11)
C53	1748(3)	1463(3)	-4968(2)	65.5(11)
C54	2050(2)	1640(2)	-4168(2)	53.4(9)
C61	2539.5(17)	3957.2(14)	1604.1(18)	34.1(5)
C62	2257(3)	4721.0(16)	1642(3)	58.5(9)
C71	1480.3(17)	2049.9(16)	2352.2(18)	35.9(6)
C72	644(2)	1793(2)	2348(2)	57.5(9)

Cl1A	4856(3)	4870(2)	1860.8(15)	83.4(12)
Cl2A	5102(3)	4944(4)	43.3(16)	92.2(16)
C1S	5519(3)	4955(6)	1042(2)	78(2)
Cl3	1130.8(8)	4601.8(7)	3942.5(8)	71.9(3)
Cl4	46.1(17)	3555(2)	3384(3)	101.9(11)
Cl4A	216(4)	3284(5)	3763(6)	92(2)
Cl5	-179(3)	5522(3)	1257(3)	100.7(15)
Cl6	268(3)	4019(3)	1533(3)	92.8(13)
C2S	1047(3)	3704(3)	3649(4)	74.3(11)
C3S	-106(9)	4800(5)	1963(7)	83(3)

Table S19. Anisotropic Displacement Parameters (Å^2×10^3) for Cu/L12. The Anisotropic displacement factor exponent takes the form: -2π^2[h^{2}a*^{2}U$_{11}$+2hka*b*U$_{12}$+…].

Atom	U_{11}	U_{22}	U_{33}	U_{12}	U_{13}	U_{23}
Cu1	26.67(14)	26.16(12)	18.51(12)	1.81(11)	-1.40(11)	-0.49(10)
O1	29.5(10)	54.2(12)	31.2(10)	-10.7(8)	2.4(8)	-13.2(8)
O2	52.0(12)	27.7(9)	38.5(10)	10.2(8)	-14.3(9)	-4.7(8)
O11	34.4(10)	31.4(8)	30.9(9)	5.1(7)	4.4(7)	1.3(7)
O12	68.2(16)	70.7(15)	27.2(10)	20.2(14)	8.5(11)	6.8(10)
O21	30.1(9)	42.0(10)	26.6(8)	1.3(8)	-3.5(7)	1.4(8)
O22	48.5(12)	78.0(15)	31.9(10)	-13.9(11)	-7.7(10)	12.5(11)
N1	29.2(11)	28.5(9)	21.2(9)	0.0(8)	1.1(8)	-4.3(8)
N2	29.0(11)	25.1(9)	21.8(9)	0.3(8)	-4.1(8)	-0.4(7)
C1	33.2(14)	32.1(13)	53.7(18)	2.4(10)	-9.6(13)	-3.3(12)
C2	40.4(17)	40.0(16)	88(3)	11.6(13)	-9.7(18)	-13.4(17)
C3	51(2)	62(2)	69(2)	16.5(17)	6.0(18)	-27.1(19)
C4	29.1(14)	46.7(16)	49.0(17)	-2.5(12)	4.4(12)	-20.2(14)
C5	27.6(13)	30.9(12)	37.0(13)	-3.1(10)	-0.1(11)	-7.6(10)
C6	33.8(15)	44.2(15)	67(2)	0.5(12)	-15.7(14)	-1.9(15)
C7	50(2)	59(2)	72(3)	-4.6(16)	-34.7(19)	14.7(19)
C8	50.3(19)	38.3(14)	50.1(18)	-2.8(13)	-23.2(15)	11.8(13)
C9	37.6(15)	25.3(11)	48.6(16)	2.7(10)	-13.8(12)	4.8(11)

C10	31.0(13)	25.5(10)	26.1(11)	-3.1(9)	0.1(10)	0.7(9)
C11	26.5(11)	29.5(10)	25.7(11)	-5.3(10)	2.3(9)	-3.6(10)
C12	34.0(14)	35.2(12)	28.7(12)	-6.6(10)	1.9(10)	-0.7(10)
C13	45.9(17)	53.9(16)	26.4(13)	-16.5(14)	5.4(12)	-1.9(12)
C14	42.7(16)	63.9(19)	34.8(14)	-10.8(15)	14.4(12)	-17.1(14)
C15	37.0(18)	132(4)	43.1(19)	-14(2)	3.9(15)	-43(2)
C16	34.4(14)	34.9(12)	23.7(11)	-4.8(10)	0.9(10)	-8.2(10)
C17	53.3(18)	42.2(14)	22.4(12)	-14.7(13)	0.9(12)	-5.9(10)
C20	31.0(13)	21.2(10)	32.8(12)	-2.6(9)	-5.3(10)	0.0(9)
C21	35.5(14)	22.4(10)	38.0(14)	-0.2(9)	-14.0(11)	3.9(10)
C22	43.8(16)	33.1(13)	38.8(15)	-5.7(11)	-10.9(12)	5.9(11)
C23	61(2)	42.7(15)	34.6(15)	-10.1(14)	-13.0(14)	15.0(12)
C24	70(2)	47.4(17)	45.1(17)	-8.8(16)	-29.6(17)	18.1(14)
C25	61(2)	40.0(14)	31.2(14)	20.2(14)	-9.1(13)	-10.5(11)
C26	34.4(14)	23.8(10)	22.1(11)	-0.1(9)	-5.8(9)	-1.9(8)
C27	43.5(16)	24.2(10)	26.6(11)	-3.1(9)	-5.0(10)	1.3(9)
C31	39.2(15)	39.1(13)	20.8(11)	-11.6(11)	1.9(10)	-2.3(10)
C32	47.8(17)	34.0(12)	30.8(13)	4.4(12)	-1.9(12)	-3.8(11)
C33	42.2(16)	34.3(13)	27.1(12)	2.4(11)	-5.1(11)	-0.9(10)
C34	26.6(12)	32.1(12)	24.4(12)	-1.7(9)	3.1(10)	0.9(9)
C35	30.6(14)	44.5(14)	27.6(12)	6.5(11)	5.0(10)	2.5(10)
C36	34.9(15)	50.4(15)	25.6(12)	0.3(12)	-2.3(11)	4.9(11)
C37	28.7(13)	35.0(12)	24.2(11)	0.7(10)	2.9(10)	0.1(10)
C38	43.4(15)	32.8(12)	29.0(13)	0.1(11)	-0.2(12)	3.1(11)
C39	41.8(16)	45.7(15)	22.6(12)	1.0(12)	0.0(11)	4.1(11)
C40	49.7(17)	48.9(17)	27.4(13)	6.9(13)	4.8(12)	-5.6(12)
C41	74(2)	38.6(15)	40.7(16)	10.5(15)	13.2(15)	-5.1(12)
C42	59.2(19)	36.5(13)	28.5(13)	11.4(13)	10.9(13)	4.1(11)
C43	38.9(14)	22.5(10)	24.6(11)	1.5(10)	-4.2(10)	0.5(8)
C44	32.7(14)	36.9(13)	25.0(12)	9.6(10)	0.5(10)	-1.3(10)
C45	30.0(13)	39.4(13)	30.6(12)	8.7(10)	-1.0(11)	-5.6(11)
C46	39.8(14)	28.0(11)	24.5(11)	6.0(11)	-2.5(11)	0.1(9)
C47	37.7(15)	38.0(13)	25.2(12)	-2.8(11)	4.8(10)	2.4(10)
C48	36.8(14)	33.2(12)	29.7(12)	-6.5(11)	0.1(11)	2.5(10)
C49	37.2(14)	44.8(14)	25.3(12)	10.2(11)	-2.9(10)	-6.4(11)

C50	43.4(16)	46.8(16)	35.0(14)	7.2(13)	-5.5(13)	-10.7(12)
C51	48.8(19)	71(2)	44.3(18)	9.8(17)	-9.0(15)	-28.1(17)
C52	60(2)	100(3)	32.2(17)	18(2)	-11.1(16)	-23.2(19)
C53	70(3)	101(3)	25.2(15)	4(2)	-2.5(16)	3.1(18)
C54	58(2)	72(2)	29.4(14)	-4.2(17)	-2.1(14)	4.6(14)
C61	37.0(14)	34.1(13)	31.2(12)	3.9(11)	-4.1(11)	-0.8(10)
C62	77(3)	33.4(14)	65(2)	6.1(16)	-3(2)	4.3(14)
C71	32.6(14)	42.1(14)	32.9(13)	-2.3(11)	-2.8(11)	1.3(11)
C72	38.5(18)	82(3)	52.0(19)	-18.2(17)	-2.5(15)	7.7(19)
Cl1A	102(4)	75(3)	73.5(12)	34(2)	-15.1(16)	-13.8(13)
Cl2A	111(3)	65.5(19)	100.0(16)	-15(2)	62(3)	-1(2)
C1S	72(3)	73(3)	91(3)	2.7(14)	-4.8(14)	0.6(14)
Cl3	67.8(7)	82.9(7)	65.1(7)	12.8(5)	-3.3(5)	-6.5(5)
Cl4	66.9(12)	104.7(17)	134(2)	-3.6(8)	4.9(9)	-19.0(11)
Cl4A	66(3)	115(4)	93(4)	-19.2(19)	33.0(19)	-33(2)
Cl5	93(3)	127(3)	82(2)	-10.6(15)	3.1(16)	16.3(14)
Cl6	71(2)	116(3)	91(2)	-4.4(14)	-3.0(15)	-21.1(14)
C2S	66.8(17)	81.2(17)	75(2)	2.5(8)	14.2(10)	-7.3(9)
C3S	79(5)	107(3)	62(3)	-5.5(15)	8.6(17)	-2.2(13)

Table S20. Bond Lengths for Cu/L12.

Atom	Atom	Length/Å	Atom	Atom	Length/Å
Cu1	O11	1.9686(18)	C23	C24	1.383(5)
Cu1	O21	1.9793(19)	C25	C26	1.524(4)
Cu1	N1	2.017(2)	C26	C27	1.520(3)
Cu1	N2	1.996(2)	C27	C43	1.511(3)
O1	C10	1.343(3)	C31	C32	1.390(4)
O1	C15	1.439(4)	C31	C36	1.367(4)
O2	C20	1.339(3)	C32	C33	1.391(4)
O2	C25	1.446(3)	C33	C34	1.396(4)
O11	C61	1.278(3)	C34	C35	1.397(4)
O12	C61	1.237(4)	C34	C37	1.489(3)
O21	C71	1.276(3)	C35	C36	1.387(4)

O22	C71	1.241(4)	C37	C38	1.391(4)
N1	C10	1.271(3)	C37	C42	1.395(4)
N1	C16	1.483(3)	C38	C39	1.381(4)
N2	C20	1.277(3)	C39	C40	1.375(4)
N2	C26	1.492(3)	C40	C41	1.389(4)
C1	C2	1.560(4)	C41	C42	1.380(4)
C1	C5	1.508(4)	C43	C44	1.390(4)
C1	C6	1.564(4)	C43	C48	1.395(4)
C1	C9	1.518(4)	C44	C45	1.393(4)
C2	C3	1.520(6)	C45	C46	1.391(4)
C3	C4	1.511(5)	C46	C47	1.387(4)
C4	C5	1.405(4)	C46	C49	1.488(3)
C4	C14	1.370(5)	C47	C48	1.393(4)
C5	C11	1.391(4)	C49	C50	1.383(5)
C6	C7	1.535(5)	C49	C54	1.390(4)
C7	C8	1.518(5)	C50	C51	1.398(4)
C8	C9	1.392(4)	C51	C52	1.362(6)
C8	C24	1.383(5)	C52	C53	1.386(7)
C9	C21	1.413(4)	C53	C54	1.394(5)
C10	C11	1.483(3)	C61	C62	1.508(4)
C11	C12	1.400(4)	C71	C72	1.513(4)
C12	C13	1.395(4)	Cl1A	C1S	1.7218(10)
C13	C14	1.379(5)	Cl2A	C1S	1.7183(10)
C15	C16	1.532(5)	Cl3	C2S	1.745(6)
C16	C17	1.527(4)	Cl4	C2S	1.790(6)
C17	C31	1.506(3)	Cl4A	C2S	1.638(7)
C20	C21	1.485(4)	Cl5	C3S	1.748(8)
C21	C22	1.381(4)	Cl6	C3S	1.730(8)
C22	C23	1.383(4)			

Table S21. Bond Angles for Cu/L12.

Atom	Atom	Atom	Angle/˚	Atom	Atom	Atom	Angle/˚
O21	Cu1	O11	90.27(8)	C22	C21	C20	118.7(2)

N1	Cu1	O11	88.02(8)	C23	C22	C21	120.7(3)
N1	Cu1	O21	167.38(8)	C24	C23	C22	120.4(3)
N2	Cu1	O11	167.05(8)	C23	C24	C8	119.5(3)
N2	Cu1	O21	88.73(8)	C26	C25	O2	104.4(2)
N2	Cu1	N1	95.70(8)	C25	C26	N2	102.3(2)
C15	O1	C10	106.7(2)	C27	C26	N2	114.07(19)
C25	O2	C20	106.2(2)	C27	C26	C25	112.9(2)
C61	O11	Cu1	104.59(16)	C43	C27	C26	110.28(19)
C71	O21	Cu1	103.04(16)	C32	C31	C17	120.5(3)
C10	N1	Cu1	132.60(17)	C36	C31	C17	121.2(3)
C16	N1	Cu1	120.07(17)	C36	C31	C32	118.2(2)
C16	N1	C10	107.3(2)	C33	C32	C31	120.9(3)
C20	N2	Cu1	131.57(17)	C34	C33	C32	120.8(2)
C26	N2	Cu1	121.33(15)	C35	C34	C33	117.4(2)
C26	N2	C20	107.1(2)	C37	C34	C33	121.3(2)
C5	C1	C2	102.0(3)	C37	C34	C35	121.2(2)
C6	C1	C2	110.4(3)	C36	C35	C34	120.8(3)
C6	C1	C5	114.8(2)	C35	C36	C31	121.7(3)
C9	C1	C2	113.2(3)	C38	C37	C34	121.2(2)
C9	C1	C5	115.8(2)	C42	C37	C34	120.5(2)
C9	C1	C6	101.0(3)	C42	C37	C38	118.3(2)
C3	C2	C1	105.8(3)	C39	C38	C37	121.5(2)
C4	C3	C2	103.7(3)	C40	C39	C38	119.6(3)
C5	C4	C3	109.4(3)	C41	C40	C39	119.8(3)
C14	C4	C3	129.6(3)	C42	C41	C40	120.6(3)
C14	C4	C5	121.0(3)	C41	C42	C37	120.1(3)
C4	C5	C1	111.3(3)	C44	C43	C27	120.1(2)
C11	C5	C1	129.6(2)	C48	C43	C27	122.1(2)
C11	C5	C4	119.1(3)	C48	C43	C44	117.8(2)
C7	C6	C1	104.9(3)	C45	C44	C43	121.1(2)
C8	C7	C6	103.0(3)	C46	C45	C44	120.9(3)
C9	C8	C7	109.4(3)	C47	C46	C45	118.2(2)
C24	C8	C7	129.5(3)	C49	C46	C45	120.1(3)
C24	C8	C9	121.0(3)	C49	C46	C47	121.8(2)
C8	C9	C1	111.7(3)	C48	C47	C46	121.0(2)

C21	C9	C1	129.4(3)	C47	C48	C43	121.0(3)
C21	C9	C8	118.9(3)	C50	C49	C46	121.6(3)
N1	C10	O1	117.4(2)	C54	C49	C46	120.0(3)
C11	C10	O1	113.9(2)	C54	C49	C50	118.4(3)
C11	C10	N1	128.7(2)	C51	C50	C49	121.0(3)
C10	C11	C5	122.3(2)	C52	C51	C50	119.9(4)
C12	C11	C5	120.0(2)	C53	C52	C51	120.3(3)
C12	C11	C10	117.6(2)	C54	C53	C52	119.8(4)
C13	C12	C11	119.2(3)	C53	C54	C49	120.6(4)
C14	C13	C12	120.9(3)	O12	C61	O11	122.4(2)
C13	C14	C4	119.7(3)	C62	C61	O11	116.3(3)
C16	C15	O1	103.9(2)	C62	C61	O12	121.3(3)
C15	C16	N1	102.8(2)	O22	C71	O21	122.2(3)
C17	C16	N1	112.9(2)	C72	C71	O21	116.6(3)
C17	C16	C15	111.3(3)	C72	C71	O22	121.2(3)
C31	C17	C16	111.7(2)	Cl2A	C1S	Cl1A	113.6(2)
N2	C20	O2	117.4(2)	Cl4	C2S	Cl3	106.9(3)
C21	C20	O2	115.0(2)	Cl4A	C2S	Cl3	120.2(4)
C21	C20	N2	127.5(2)	Cl4A	C2S	Cl4	27.7(4)
C20	C21	C9	121.8(3)	Cl6	C3S	Cl5	115.6(7)
C22	C21	C9	119.4(3)				

Table 22. Torsion Angles for Cu/L12.

A	B	C	D	Angle/°	A	B	C	D	Angle/°
Cu1	O11	C61	O12	-0.6(2)	C8	C9	C21	C22	0.5(3)
Cu1	O11	C61	C62	178.5(2)	C8	C24	C23	C22	0.7(4)
Cu1	O21	C71	O22	-6.2(2)	C9	C1	C5	C11	39.8(3)
Cu1	O21	C71	C72	175.9(2)	C9	C8	C24	C23	-2.7(4)
Cu1	N1	C10	O1	-179.8(2)	C9	C21	C22	C23	-2.4(3)
Cu1	N1	C10	C11	2.5(3)	C10	O1	C15	C16	-12.4(2)
Cu1	N1	C16	C15	172.0(3)	C10	N1	C16	C15	-10.6(3)
Cu1	N1	C16	C17	51.9(2)	C10	N1	C16	C17	-130.6(2)
Cu1	N2	C20	O2	-175.5(2)	C10	C11	C12	C13	174.6(2)

Cu1	N2	C20	C21	8.7(3)	C11	C5	C4	C14	1.1(3)
Cu1	N2	C26	C25	167.4(2)	C11	C10	O1	C15	-175.6(3)
Cu1	N2	C26	C27	45.2(2)	C11	C10	N1	C16	-174.5(3)
O1	C10	N1	C16	3.2(3)	C11	C12	C13	C14	2.1(3)
O1	C10	C11	C5	68.2(2)	C15	C16	C17	C31	63.4(3)
O1	C10	C11	C12	-109.2(2)	C16	C17	C31	C32	-108.2(3)
O1	C15	C16	N1	13.7(4)	C16	C17	C31	C36	69.6(3)
O1	C15	C16	C17	134.8(3)	C17	C31	C32	C33	-179.9(3)
O2	C20	N2	C26	5.0(3)	C17	C31	C36	C35	179.5(3)
O2	C20	C21	C9	66.1(3)	C20	O2	C25	C26	-13.7(2)
O2	C20	C21	C22	-111.2(3)	C20	N2	C26	C25	-13.0(2)
O2	C25	C26	N2	15.9(3)	C20	N2	C26	C27	-135.3(2)
O2	C25	C26	C27	138.9(2)	C20	C21	C22	C23	174.9(3)
N1	C10	O1	C15	6.4(3)	C21	C9	C8	C24	2.0(3)
N1	C10	C11	C5	-114.0(3)	C21	C20	O2	C25	-177.7(3)
N1	C10	C11	C12	68.5(3)	C21	C20	N2	C26	-170.8(3)
N1	C16	C17	C31	178.5(2)	C21	C22	C23	C24	1.8(4)
N2	C20	O2	C25	6.0(3)	C25	C26	C27	C43	64.9(2)
N2	C20	C21	C9	-118.0(3)	C26	C27	C43	C44	70.1(3)
N2	C20	C21	C22	64.8(3)	C26	C27	C43	C48	-109.7(2)
N2	C26	C27	C43	-178.9(2)	C27	C43	C44	C45	179.2(2)
C1	C2	C3	C4	-27.1(3)	C27	C43	C48	C47	-177.7(2)
C1	C5	C4	C3	-1.3(3)	C31	C32	C33	C34	-0.4(4)
C1	C5	C4	C14	179.8(2)	C31	C36	C35	C34	1.3(3)
C1	C5	C11	C10	5.5(3)	C32	C31	C36	C35	-2.6(3)
C1	C5	C11	C12	-177.1(3)	C32	C33	C34	C35	-1.0(3)
C1	C6	C7	C8	-30.2(3)	C32	C33	C34	C37	177.1(3)
C1	C9	C8	C7	0.3(3)	C33	C32	C31	C36	2.2(4)
C1	C9	C8	C24	-179.1(3)	C33	C34	C35	C36	0.6(3)
C1	C9	C21	C20	4.7(3)	C33	C34	C37	C38	-32.3(3)
C1	C9	C21	C22	-178.1(3)	C33	C34	C37	C42	149.5(3)
C2	C1	C5	C4	-15.4(3)	C34	C37	C38	C39	-178.0(3)
C2	C1	C5	C11	163.1(2)	C34	C37	C42	C41	177.0(3)
C2	C1	C6	C7	-90.3(3)	C35	C34	C37	C38	145.7(3)
C2	C1	C9	C8	99.1(3)	C35	C34	C37	C42	-32.5(3)

C2	C1	C9	C21	-82.2(3)	C36	C35	C34	C37	-177.5(3)
C2	C3	C4	C5	18.0(3)	C37	C38	C39	C40	0.7(4)
C2	C3	C4	C14	-163.3(3)	C37	C42	C41	C40	1.4(4)
C3	C2	C1	C5	26.0(3)	C38	C37	C42	C41	-1.3(3)
C3	C2	C1	C6	-96.5(3)	C38	C39	C40	C41	-0.6(4)
C3	C2	C1	C9	151.1(3)	C39	C38	C37	C42	0.2(4)
C3	C4	C5	C11	180.0(2)	C39	C40	C41	C42	-0.5(4)
C3	C4	C14	C13	179.4(4)	C43	C44	C45	C46	-1.8(3)
C4	C5	C1	C6	104.0(3)	C43	C48	C47	C46	-1.3(3)
C4	C5	C1	C9	-138.8(2)	C44	C43	C48	C47	2.6(3)
C4	C5	C11	C10	-176.0(2)	C44	C45	C46	C47	3.0(3)
C4	C5	C11	C12	1.4(3)	C44	C45	C46	C49	-176.1(2)
C4	C14	C13	C12	0.4(4)	C45	C44	C43	C48	-1.0(3)
C5	C1	C6	C7	155.1(3)	C45	C46	C47	C48	-1.5(3)
C5	C1	C9	C8	-143.6(3)	C45	C46	C49	C50	-47.3(3)
C5	C1	C9	C21	35.1(3)	C45	C46	C49	C54	130.3(3)
C5	C4	C14	C13	-2.0(3)	C46	C49	C50	C51	177.1(3)
C5	C11	C12	C13	-2.9(3)	C46	C49	C54	C53	-177.4(3)
C6	C1	C5	C11	-77.4(3)	C47	C46	C49	C50	133.6(3)
C6	C1	C9	C8	-19.0(3)	C47	C46	C49	C54	-48.8(3)
C6	C1	C9	C21	159.7(2)	C48	C47	C46	C49	177.6(2)
C6	C7	C8	C9	19.0(3)	C49	C50	C51	C52	0.5(4)
C6	C7	C8	C24	-161.6(3)	C49	C54	C53	C52	0.2(4)
C7	C6	C1	C9	29.8(3)	C50	C49	C54	C53	0.2(4)
C7	C8	C9	C21	-178.6(3)	C50	C51	C52	C53	-0.1(4)
C7	C8	C24	C23	178.1(4)	C51	C50	C49	C54	-0.5(4)
C8	C9	C21	C20	-176.7(2)	C51	C52	C53	C54	-0.2(5)

Table S23. Hydrogen Atom Coordinates (Å×10^4) and Isotropic Displacement Parameters (Å^2×10^3) for Cu/L12.

Atom	*x*	*y*	*z*	U(eq)
H2a	6264(2)	612.1(18)	1844(3)	67.4(11)
H2b	5414(2)	518.2(18)	1423(3)	67.4(11)

H3a	6695(2)	1313(2)	760(3)	72.6(12)
H3b	6049(2)	873(2)	228(3)	72.6(12)
H6a	6106.6(19)	2265.8(18)	2873(2)	58.1(9)
H6b	6691.2(19)	1660.6(18)	2518(2)	58.1(9)
H7a	6310(2)	842(2)	3530(3)	72.6(12)
H7b	6165(2)	1564(2)	4074(3)	72.6(12)
H12	4361.6(17)	3469.1(14)	795.6(17)	39.2(7)
H13	4976(2)	3182.7(18)	-497.5(18)	50.5(8)
H14	5777.4(19)	2183(2)	-600.4(19)	56.5(9)
H15a	4756(2)	2935(3)	4091(2)	84.9(16)
H15b	4778(2)	3777(3)	3863(2)	84.9(16)
H16	3564.9(16)	3762.2(14)	3330.9(16)	37.2(6)
H17a	3513.0(19)	2357.1(16)	4096.8(17)	47.2(8)
H17b	2753.6(19)	2803.7(16)	3833.5(17)	47.2(8)
H22	3027.0(19)	987.9(14)	3081.9(19)	46.3(7)
H23	3509(2)	749.0(17)	4441(2)	55.3(9)
H24	4840(2)	858.0(18)	4724(2)	65.0(11)
H25a	4014(2)	1168.9(17)	-109.1(19)	52.7(9)
H25b	3373(2)	548.2(17)	17.1(19)	52.7(9)
H26	2486.6(15)	1302.4(12)	538.7(15)	32.1(6)
H27a	2621.5(17)	2522.0(13)	86.4(16)	37.7(7)
H27b	3510.3(17)	2367.5(13)	-169.7(16)	37.7(7)
H32	3994.6(19)	2478.1(15)	5497.3(18)	45.0(7)
H33	3996.9(18)	3023.3(14)	6835.6(17)	41.5(7)
H35	2629.2(16)	4604.6(15)	5899.1(16)	41.1(7)
H36	2650.2(17)	4056.0(16)	4569.8(17)	44.3(7)
H38	3330.3(17)	3288.0(14)	8072.3(18)	42.1(7)
H39	3248.6(18)	3807.1(16)	9417.9(17)	44.0(7)
H40	3120(2)	5044.1(17)	9545.6(18)	50.4(8)
H41	3094(2)	5758.2(17)	8320(2)	61.5(10)
H42	3214(2)	5245.4(16)	6973.3(18)	49.7(8)
H44	1603.1(16)	1682.9(14)	-644.3(17)	37.8(7)
H45	1177.3(16)	1272.7(14)	-1969.6(18)	40.0(6)
H47	3321.8(17)	1610.0(14)	-2948.5(16)	40.4(7)
H48	3763.4(17)	1970.9(14)	-1608.8(17)	39.9(7)

H50	1340.5(19)	304.4(17)	-3097(2)	50.1(8)
H51	825(2)	15(2)	-4438(2)	65.7(11)
H52	1084(2)	737(3)	-5603(2)	77.1(13)
H53	1855(3)	1759(3)	-5448(2)	78.6(13)
H54	2362(2)	2057(2)	-4106(2)	64.0(10)
H62a	2306(18)	4901(6)	2228(5)	87.8(14)
H62b	2572(12)	5018(4)	1258(16)	87.8(14)
H62c	1710(6)	4741(3)	1470(20)	87.8(14)
H72a	298(2)	2197(4)	2221(19)	86.2(14)
H72b	580(4)	1423(11)	1910(13)	86.2(14)
H72c	513(6)	1595(15)	2909(6)	86.2(14)
H1Sa	5807(3)	5410(6)	1115(2)	94(3)
H1Sb	5901(3)	4559(6)	1081(2)	94(3)
H2Sa	1384(3)	3600(3)	3151(4)	89.2(13)
H2Sb	1206(3)	3390(3)	4128(4)	89.2(13)
H2Sc	1196(3)	3668(3)	3039(4)	89.2(13)
H2Sd	1443(3)	3434(3)	3978(4)	89.2(13)
H3Sa	228(9)	4945(5)	2449(7)	99(3)
H3Sb	-631(9)	4696(5)	2193(7)	99(3)

Table S24. Atomic Occupancy for Cu/L12.

**Atom**	***Occupancy***	**Atom**	***Occupancy***	**Atom**	***Occupancy***
Cl1A	0.500000	Cl2A	0.500000	C1S	0.500000
H1Sa	0.500000	H1Sb	0.500000	Cl3	0.894010
Cl4	0.624(9)	Cl4A	0.270(9)	Cl5	0.334(3)
Cl6	0.334(3)	C2S	0.894010	H2Sa	0.624(9)
H2Sb	0.624(9)	H2Sc	0.270(9)	H2Sd	0.270(9)
C3S	0.334(3)	H3Sa	0.334(3)	H3Sb	0.334(3)

IX. References

(1) Lin, S.; Song, C.-X.; Cai, G.-X.; Wang, W.-H.; Shi, Z.-J. Intra/Intermolecular Direct Allylic Alkylation via Pd(II)-Catalyzed Allylic C-H Activation. *J. Am. Chem. Soc.* **2008**, *130*, 12901–12903.

(2) Rong, Z.-Q.; Zhang, Y.; Chua, R.-H.; Pan, H.-J.; Zhao, Y. Dynamic Kinetic Asymmetric Amination of Alcohols: From A Mixture of Four Isomers to Diastereo- and Enantiopure α-Branched Amines. *J. Am. Chem. Soc.* **2015**, *137*, 4944–4947.

(3) Vemula, S. R.; Kumar, D.; Cook, G. R. Palladium-Catalyzed Allylic Amidation with N-Heterocycles via sp^3 C–H Oxidation. *ACS Catal.* **2016**, *6*, 5295–5301.

(4) Liu, Z.-C.; Wang, Z.-Q.; Zhang, X.; Yin, L. Copper(I)-catalyzed asymmetric alkylation of α-imino-esters. *Nat. Commun.* **2023**, *14*, 2187.

(5) Tan, G.; Das, M.; Keum, H.; Bellotti, P.; Daniliuc, C.; Glorius, F. Photochemical Single-Step Synthesis of β-Amino Acid Derivatives from Alkenes and (Hetero)arenes. *Nat. Chem.* **2022**, *14*, 1174–1184.

(6) Zhu, M.; Wang, P.; Zhang, Q.; Tang, W.; Zi, W. Diastereodivergent Aldol-Type Coupling of Alkoxyallenes with Pentafluorophenyl Esters Enabled by Synergistic Palladium/Chiral Lewis Base Catalysis. *Angew. Chem., Int. Ed.* **2022**, *61*, e202207621.

(7) Lang, K.; Li, C.; Kim, I.; Zhang, X.-P. Enantioconvergent Amination of Racemic Tertiary C-H Bonds. *J. Am. Chem. Soc.* **2020**, *142*, 20902–20911.

(8) Qi, X.-K.; Yao, L.-J.; Zheng, M.-J.; Zhao, L.; Yang, C.; Guo, L.; Xia, W. Photoinduced Hydrodifluoromethylation and Hydromethylation of Alkenes Enabled by Ligand-to-Iron Charge Transfer Mediated Decarboxylation. *ACS Catal.* **2024**, *14*, 1300–1310.

(9) Xiong, F.-Y.; Pan, S.-R.; Wang, B.-C.; Li, Y.-J.; Shi, J.-W.; Xiao, W.-J.; Lu, L.-Q. Construction of Sulfur(IV)–Chiral Six-Membered Heterocycles by Pd-

Catalyzed Asymmetric (4+2) Dipolar Cyclization. *Eur. J. Org. Chem.* **2024**, *28*, e202401007.

(10) Song, C.; Zhang, H.-H.; Yu, S. Regio- and Enantioselective Decarboxylative Allylic Benzylation Enabled by Dual Palladium/Photoredox Catalysis. *ACS Catal.* **2022**, *12*, 1428−1432.

(11) Lyothier, I.; Defieber, C.; Carreira, E. M. Iridium-Catalyzed Enantioselective Synthesis of Allylic Alcohols: Silanolates as Hydroxide Equivalents. *Angew. Chem., Int. Ed.* **2006**, *45*, 6204−6207.

(12) Dolomanov, O. V.; Bourhis, L. J.; Gildea, R. J.; Howard, J. A. K.; Puschmann, H. OLEX2: A Complete Structure Solution, Refinement and Analysis Program. *J. Appl. Cryst.* **2009**, *42*, 339−341.

(13) Sheldrick, G. M. SHELXT – Integrated Space-Group and Crystal Structure Determination. *Acta Cryst.* **2015**, *71*, 3−8.

(14) Bourhis, L. J.; Dolomanov, O. V.; Gildea, R. J.; Howard, J. A. K.; Puschmann, H. The Anatomy of a Comprehensive Constrained, Restrained Refinement Program for The Modern Computing Environment - Olex2 Dissected. *Acta Cryst.* **2015**, *71*, 59−75.

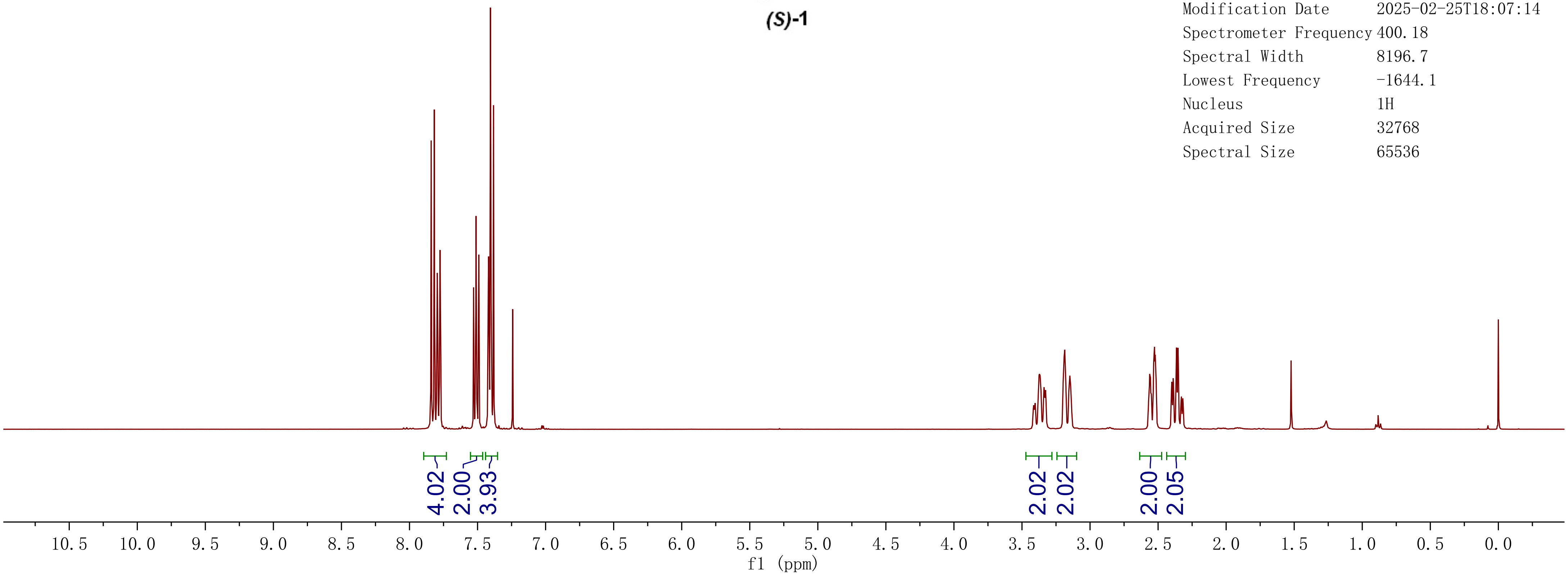

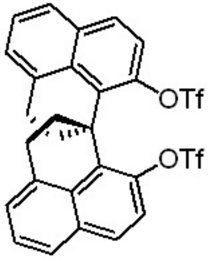

(S)-**1**

Parameter	Value
Title	qxk-132h.6.fid
Origin	Bruker BioSpin GmbH
Owner	nmr
Instrument	AvanceNeo 400MHz
Solvent	CDCl3
Temperature	296.0
Pulse Sequence	zg30
Experiment	1D
Number of Scans	16
Receiver Gain	101.0
Relaxation Delay	1.0000
Pulse Width	8.0000
Acquisition Time	3.9977
Acquisition Date	2025-02-25T18:07:22
Modification Date	2025-02-25T18:07:14
Spectrometer Frequency	400.18
Spectral Width	8196.7
Lowest Frequency	-1644.1
Nucleus	1H
Acquired Size	32768
Spectral Size	65536

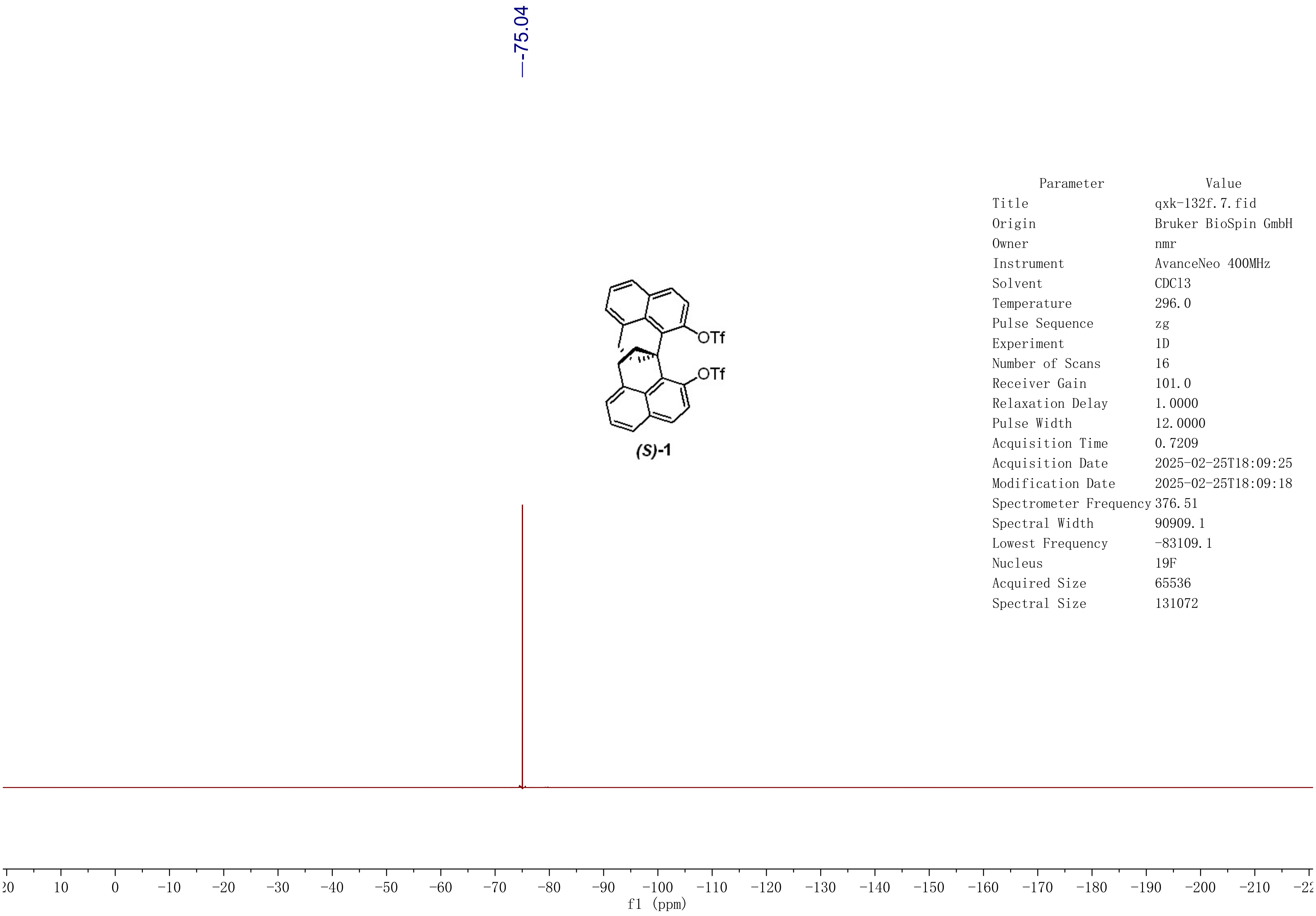

(S)-1

Parameter	Value
Title	qxk-132f.7.fid
Origin	Bruker BioSpin GmbH
Owner	nmr
Instrument	AvanceNeo 400MHz
Solvent	CDCl3
Temperature	296.0
Pulse Sequence	zg
Experiment	1D
Number of Scans	16
Receiver Gain	101.0
Relaxation Delay	1.0000
Pulse Width	12.0000
Acquisition Time	0.7209
Acquisition Date	2025-02-25T18:09:25
Modification Date	2025-02-25T18:09:18
Spectrometer Frequency	376.51
Spectral Width	90909.1
Lowest Frequency	-83109.1
Nucleus	19F
Acquired Size	65536
Spectral Size	131072

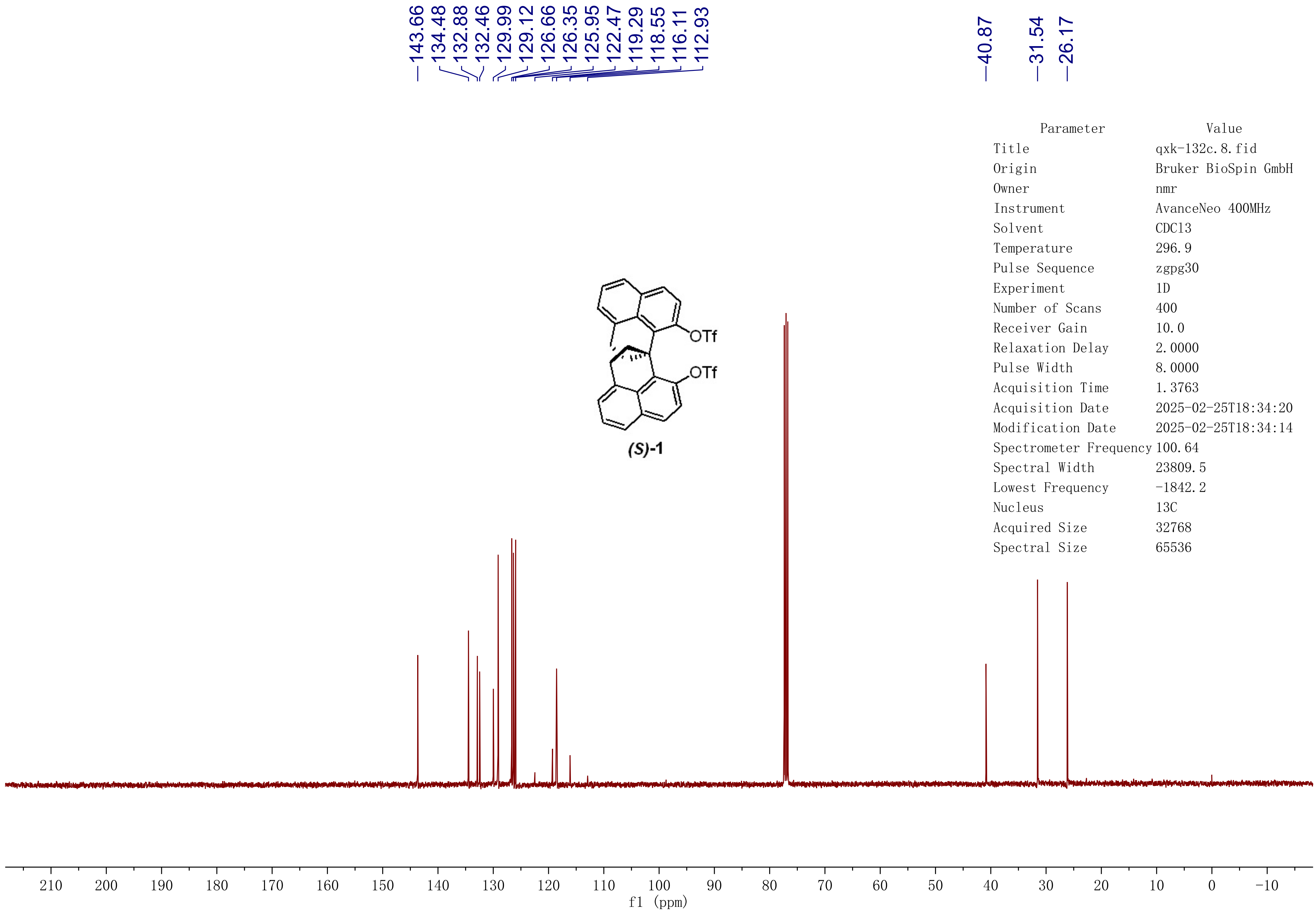

(*S*)-1

Parameter	Value
Title	qxk-132c.8.fid
Origin	Bruker BioSpin GmbH
Owner	nmr
Instrument	AvanceNeo 400MHz
Solvent	CDCl3
Temperature	296.9
Pulse Sequence	zgpg30
Experiment	1D
Number of Scans	400
Receiver Gain	10.0
Relaxation Delay	2.0000
Pulse Width	8.0000
Acquisition Time	1.3763
Acquisition Date	2025-02-25T18:34:20
Modification Date	2025-02-25T18:34:14
Spectrometer Frequency	100.64
Spectral Width	23809.5
Lowest Frequency	-1842.2
Nucleus	13C
Acquired Size	32768
Spectral Size	65536

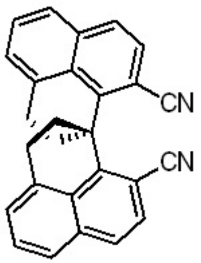

(S)-2

Parameter	Value
Title	dq-1-148-a-400m-h.1.1.1r
Origin	Bruker BioSpin GmbH
Owner	nmr
Instrument	spect
Solvent	CDCl3
Temperature	297.8
Pulse Sequence	zg30
Experiment	1D
Number of Scans	16
Receiver Gain	71.0
Relaxation Delay	1.0000
Pulse Width	14.5000
Acquisition Time	4.0894
Acquisition Date	2023-10-17T11:27:00
Modification Date	2023-10-17T11:27:42
Spectrometer Frequency	400.13
Spectral Width	8012.8
Lowest Frequency	-1545.3
Nucleus	1H
Acquired Size	32768
Spectral Size	65536

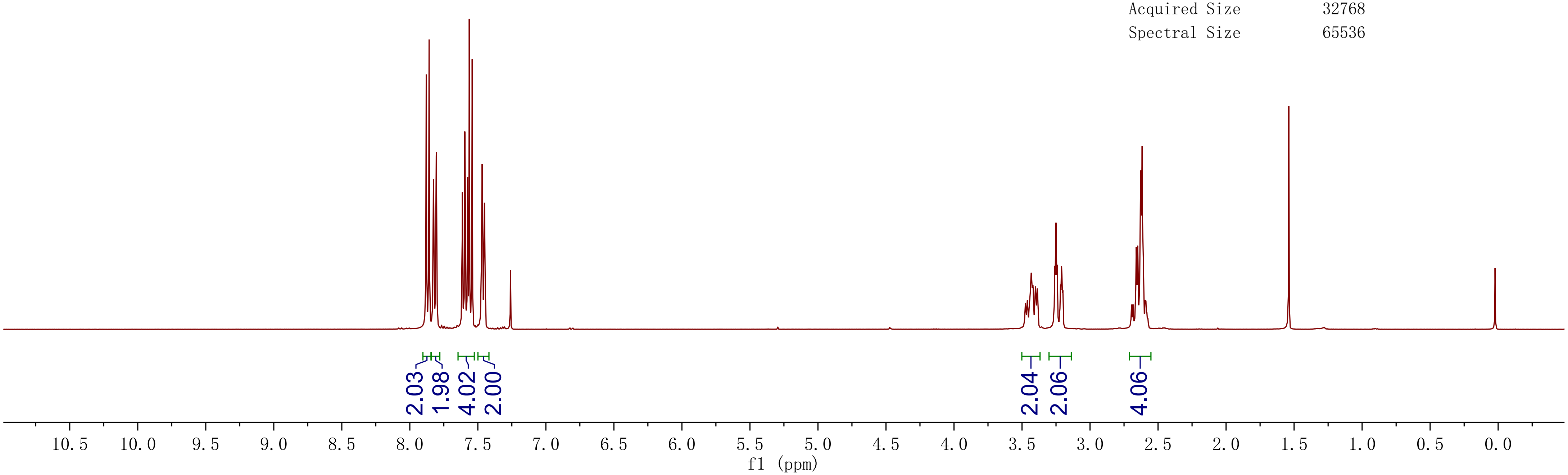

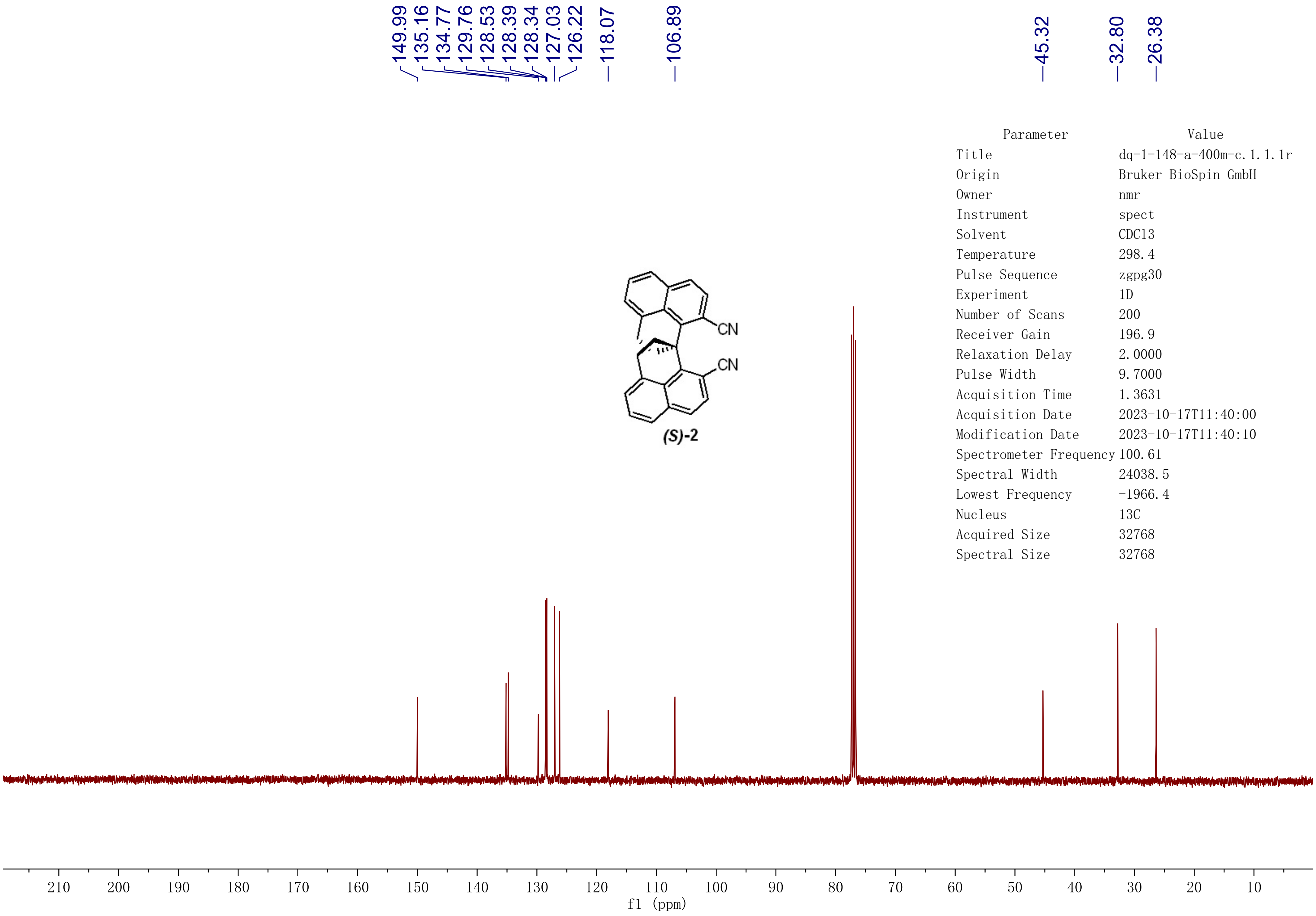

(S)-2

Parameter	Value
Title	dq-1-148-a-400m-c.1.1.1r
Origin	Bruker BioSpin GmbH
Owner	nmr
Instrument	spect
Solvent	CDCl3
Temperature	298.4
Pulse Sequence	zgpg30
Experiment	1D
Number of Scans	200
Receiver Gain	196.9
Relaxation Delay	2.0000
Pulse Width	9.7000
Acquisition Time	1.3631
Acquisition Date	2023-10-17T11:40:00
Modification Date	2023-10-17T11:40:10
Spectrometer Frequency	100.61
Spectral Width	24038.5
Lowest Frequency	-1966.4
Nucleus	13C
Acquired Size	32768
Spectral Size	32768

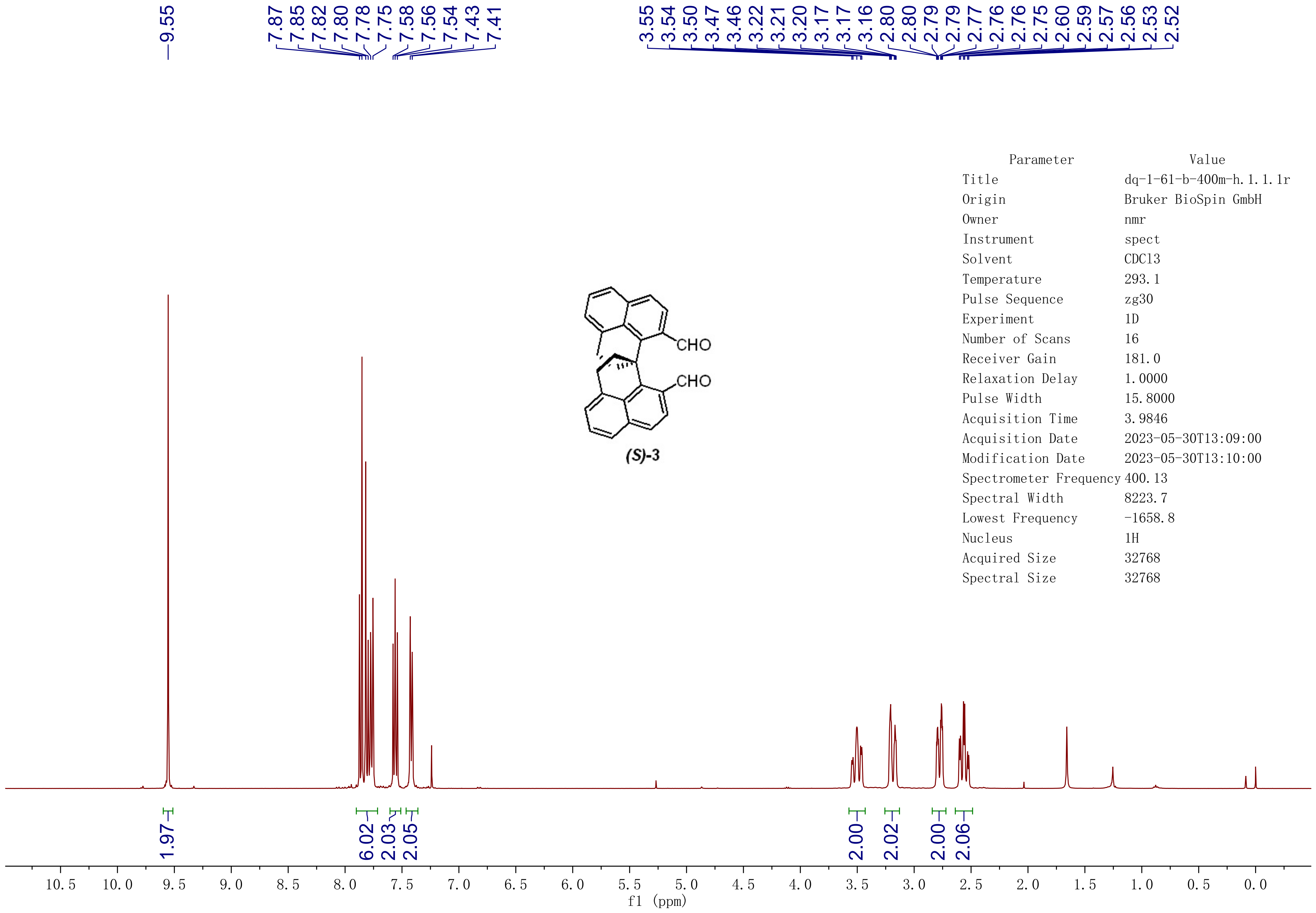

Parameter	Value
Title	dq-1-61-b-400m-h.1.1.1r
Origin	Bruker BioSpin GmbH
Owner	nmr
Instrument	spect
Solvent	CDCl3
Temperature	293.1
Pulse Sequence	zg30
Experiment	1D
Number of Scans	16
Receiver Gain	181.0
Relaxation Delay	1.0000
Pulse Width	15.8000
Acquisition Time	3.9846
Acquisition Date	2023-05-30T13:09:00
Modification Date	2023-05-30T13:10:00
Spectrometer Frequency	400.13
Spectral Width	8223.7
Lowest Frequency	-1658.8
Nucleus	1H
Acquired Size	32768
Spectral Size	32768

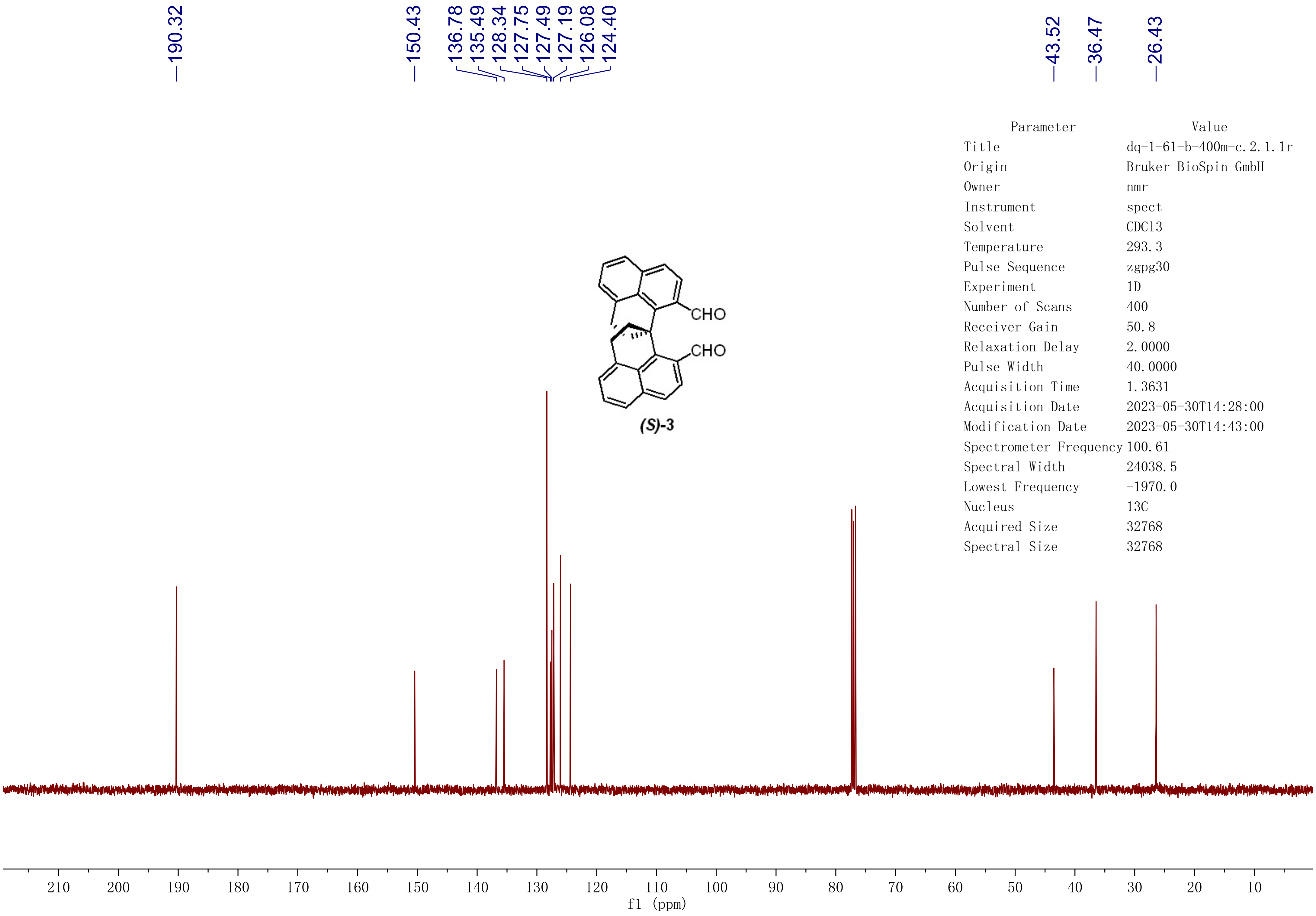

(S)-3

Parameter	Value
Title	dq-1-61-b-400m-c.2.1.1r
Origin	Bruker BioSpin GmbH
Owner	nmr
Instrument	spect
Solvent	CDCl3
Temperature	293.3
Pulse Sequence	zgpg30
Experiment	1D
Number of Scans	400
Receiver Gain	50.8
Relaxation Delay	2.0000
Pulse Width	40.0000
Acquisition Time	1.3631
Acquisition Date	2023-05-30T14:28:00
Modification Date	2023-05-30T14:43:00
Spectrometer Frequency	100.61
Spectral Width	24038.5
Lowest Frequency	-1970.0
Nucleus	13C
Acquired Size	32768
Spectral Size	32768

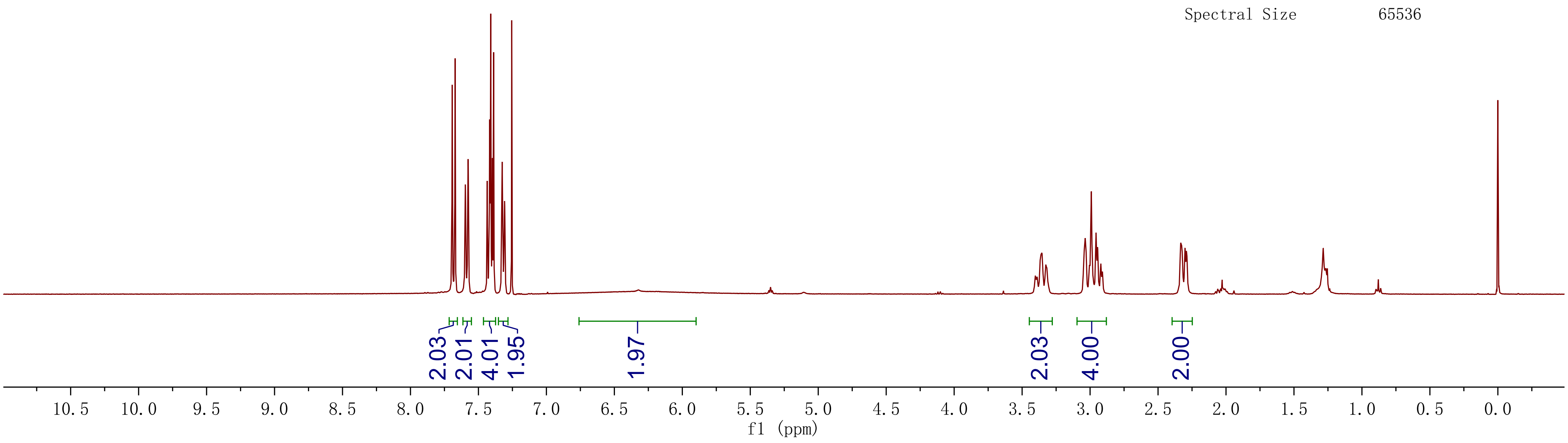

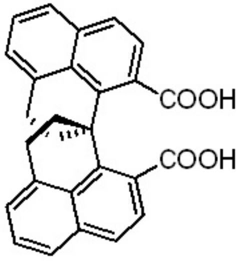

(*S*)-**4**

Parameter	Value
Title	qxk-COOH-3-4.1.fid
Origin	Bruker BioSpin GmbH
Owner	nmr
Instrument	spect
Solvent	CDCl3
Temperature	296.3
Pulse Sequence	zg30
Experiment	1D
Number of Scans	16
Receiver Gain	142.9
Relaxation Delay	1.0000
Pulse Width	14.5000
Acquisition Time	4.0894
Acquisition Date	2025-05-25T14:41:23
Modification Date	2025-05-25T14:41:26
Spectrometer Frequency	400.13
Spectral Width	8012.8
Lowest Frequency	-1547.0
Nucleus	1H
Acquired Size	32768
Spectral Size	65536

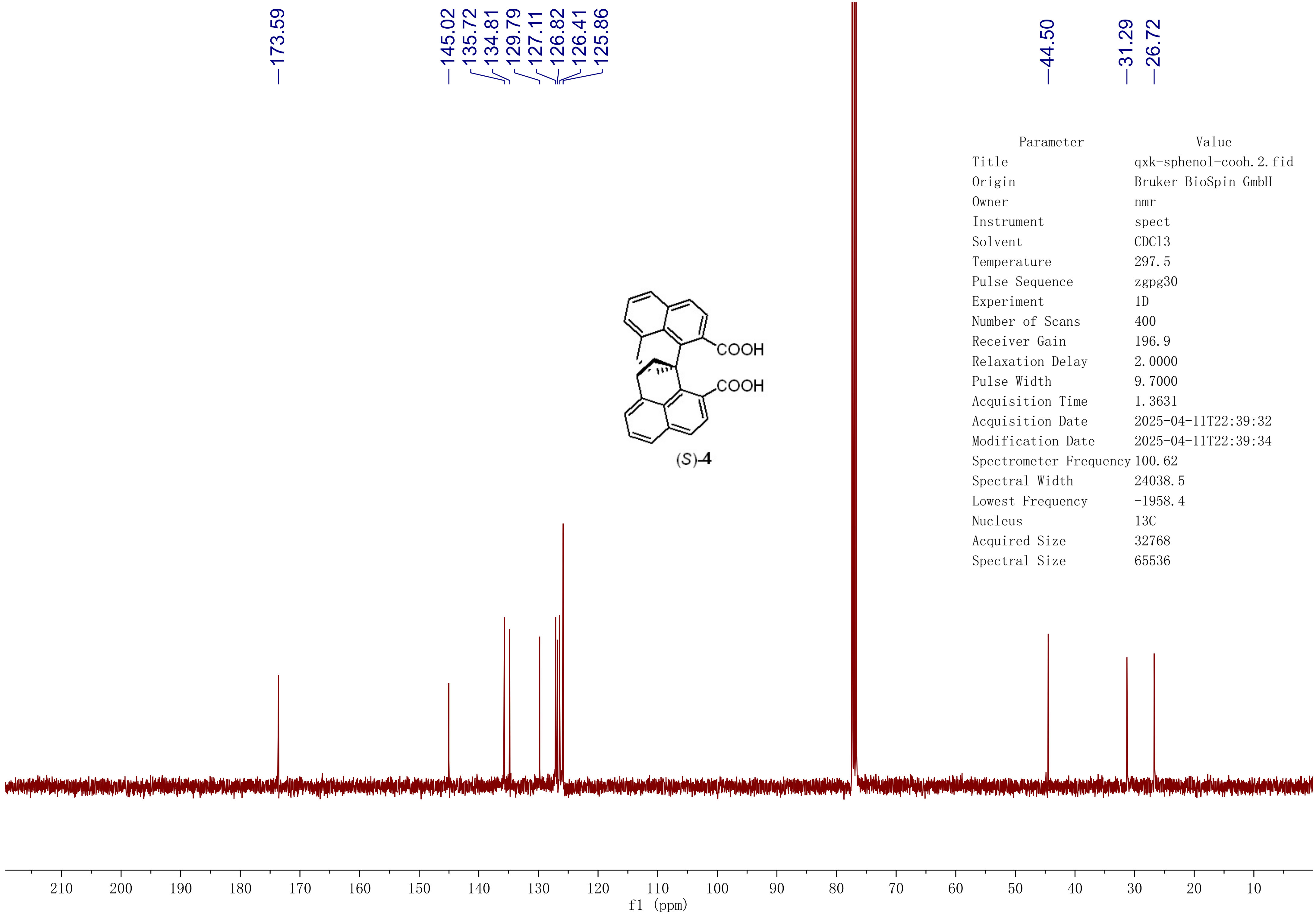

173.59
145.02
135.72
134.81
129.79
127.11
126.82
126.41
125.86
44.50
31.29
26.72
COOH
COOH
(S)-4
Parameter Value
Title qxk-sphenol-cooh.2.fid
Origin Bruker BioSpin GmbH
Owner nmr
Instrument spect
Solvent CDCl3
Temperature 297.5
Pulse Sequence zgpg30
Experiment 1D
Number of Scans 400
Receiver Gain 196.9
Relaxation Delay 2.0000
Pulse Width 9.7000
Acquisition Time 1.3631
Acquisition Date 2025-04-11T22:39:32
Modification Date 2025-04-11T22:39:34
Spectrometer Frequency 100.62
Spectral Width 24038.5
Lowest Frequency -1958.4
Nucleus 13C
Acquired Size 32768
Spectral Size 65536
210 200 190 180 170 160 150 140 130 120 110 100 90 80 70 60 50 40 30 20 10
f1 (ppm)

Parameter	Value
Title	dq-1-103-A-400m-h.1.fid
Origin	Bruker BioSpin GmbH
Owner	nmr
Instrument	AvanceNeo 400MHz
Solvent	CDCl3
Temperature	296.4
Pulse Sequence	zg30
Experiment	1D
Number of Scans	16
Receiver Gain	101.0
Relaxation Delay	1.0000
Pulse Width	8.0000
Acquisition Time	3.9977
Acquisition Date	2023-07-28T19:06:40
Modification Date	2023-07-28T19:06:36
Spectrometer Frequency	400.18
Spectral Width	8196.7
Lowest Frequency	-1637.1
Nucleus	1H
Acquired Size	32768
Spectral Size	65536

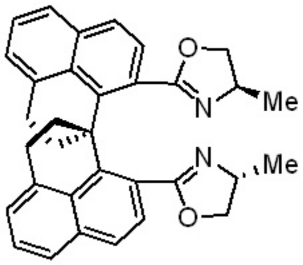

(S_S,R,R)-**L1**

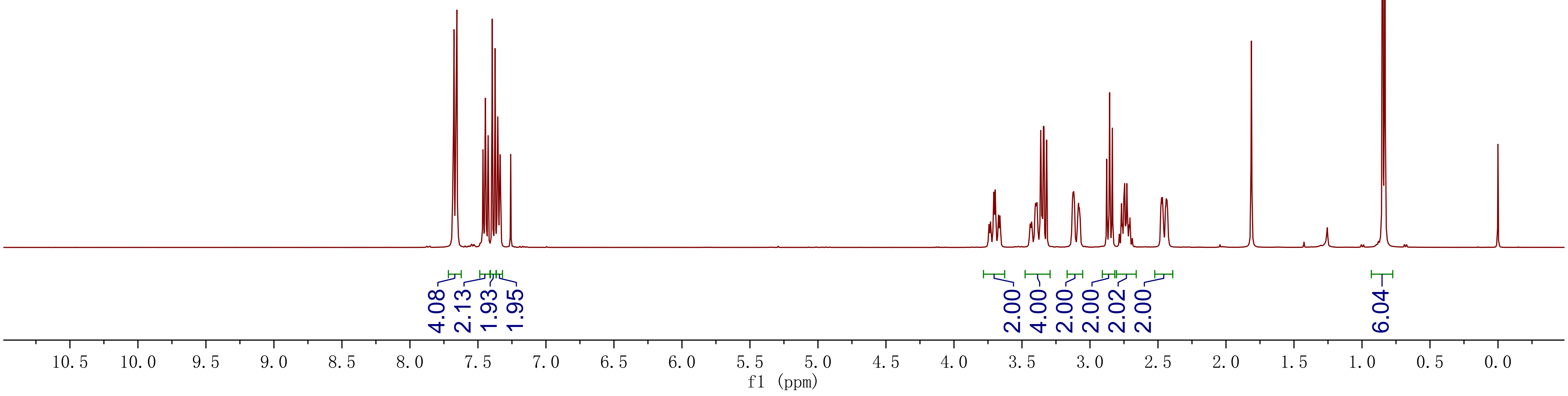

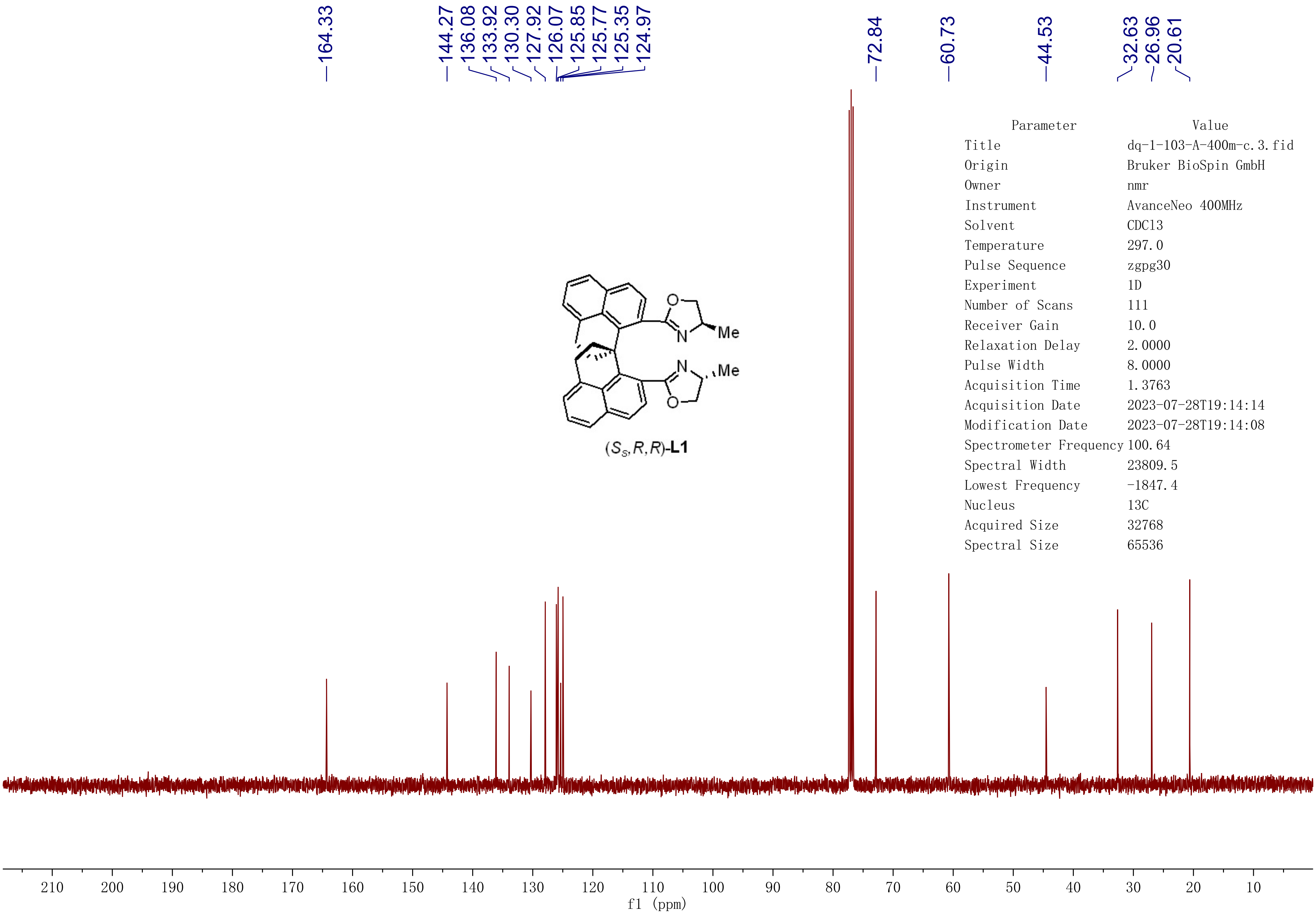
164.33
144.27
136.08
133.92
130.30
127.92
126.07
125.85
125.77
125.35
124.97
72.84
60.73
44.53
32.63
26.96
20.61
Parameter
Value
Title
dq-1-103-A-400m-c.3.fid
Origin
Bruker BioSpin GmbH
Owner
nmr
Instrument
AvanceNeo 400MHz
Solvent
CDCl3
Temperature
297.0
Pulse Sequence
zgpg30
Experiment
1D
Number of Scans
111
Receiver Gain
10.0
Relaxation Delay
2.0000
Pulse Width
8.0000
Acquisition Time
1.3763
Acquisition Date
2023-07-28T19:14:14
Modification Date
2023-07-28T19:14:08
Spectrometer Frequency
100.64
Spectral Width
23809.5
Lowest Frequency
-1847.4
Nucleus
13C
Acquired Size
32768
Spectral Size
65536
O
N
Me
N
Me
O
(S_S,R,R)-L1
210
200
190
180
170
160
150
140
130
120
110
100
90
80
70
60
50
40
30
20
10
f1 (ppm)

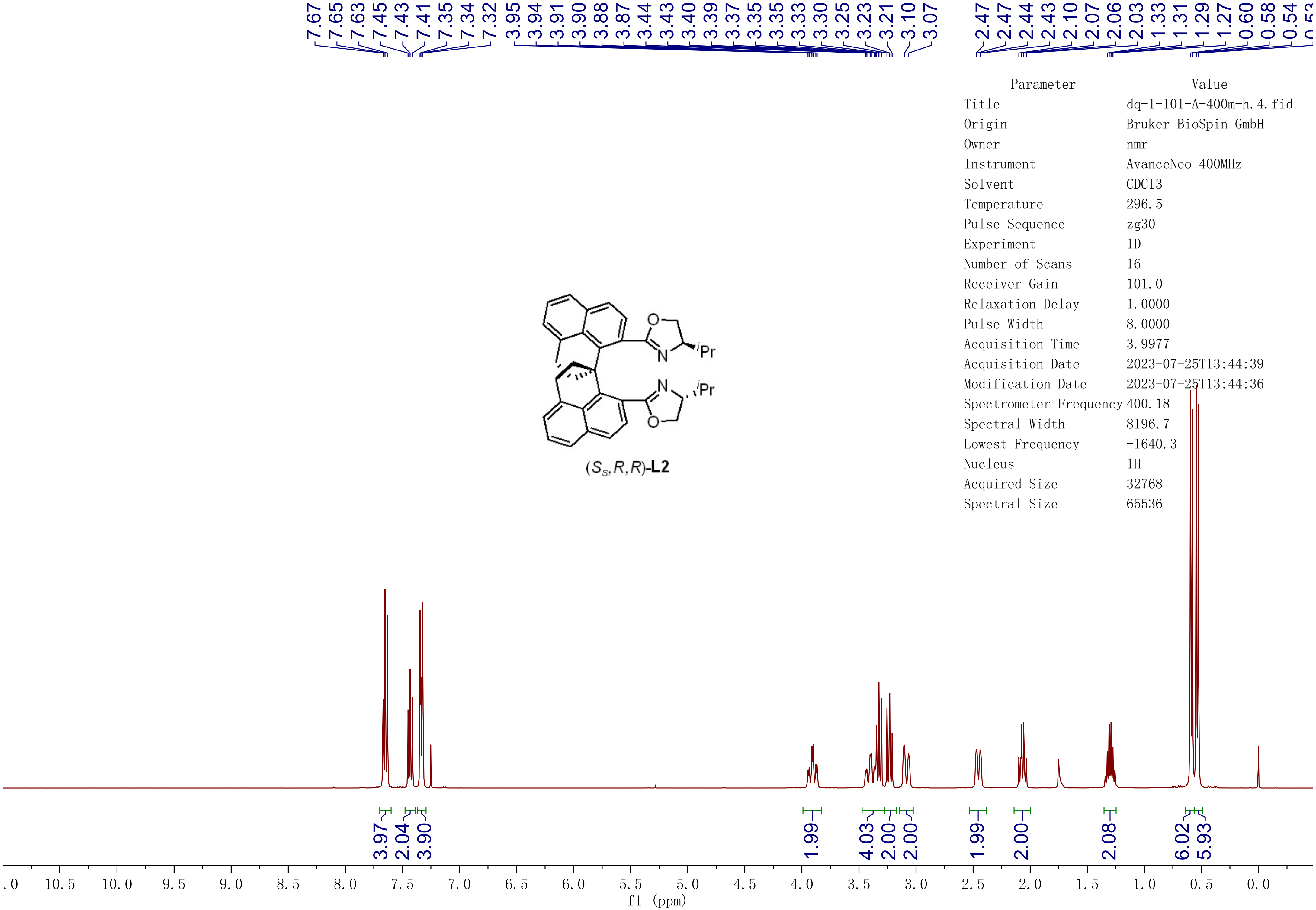

Parameter	Value
Title	dq-1-101-A-400m-h.4.fid
Origin	Bruker BioSpin GmbH
Owner	nmr
Instrument	AvanceNeo 400MHz
Solvent	CDCl3
Temperature	296.5
Pulse Sequence	zg30
Experiment	1D
Number of Scans	16
Receiver Gain	101.0
Relaxation Delay	1.0000
Pulse Width	8.0000
Acquisition Time	3.9977
Acquisition Date	2023-07-25T13:44:39
Modification Date	2023-07-25T13:44:36
Spectrometer Frequency	400.18
Spectral Width	8196.7
Lowest Frequency	-1640.3
Nucleus	1H
Acquired Size	32768
Spectral Size	65536

(S_S,R,R)-**L2**

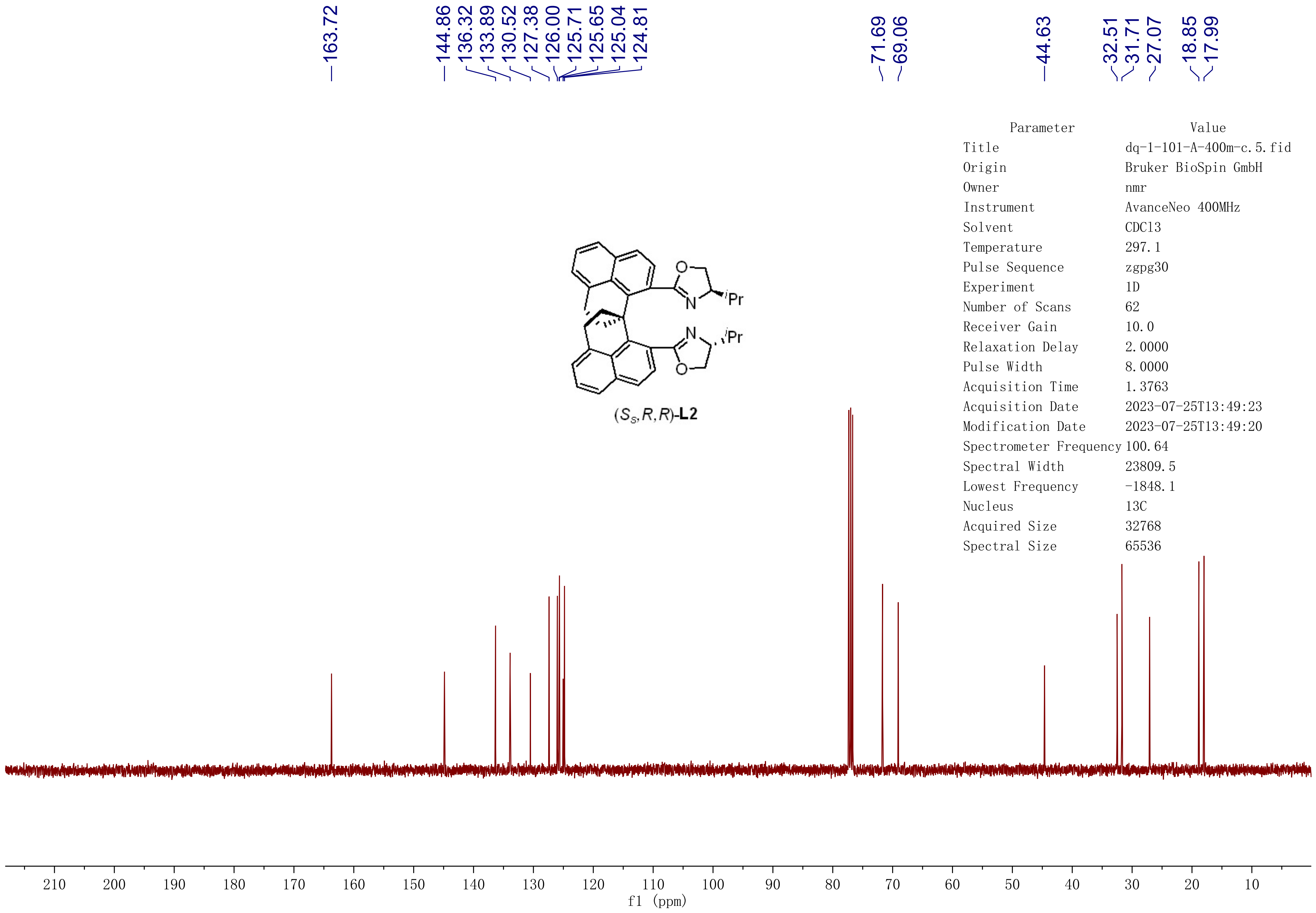

(S_S,R,R)-**L2**

Parameter	Value
Title	dq-1-101-A-400m-c.5.fid
Origin	Bruker BioSpin GmbH
Owner	nmr
Instrument	AvanceNeo 400MHz
Solvent	CDCl3
Temperature	297.1
Pulse Sequence	zgpg30
Experiment	1D
Number of Scans	62
Receiver Gain	10.0
Relaxation Delay	2.0000
Pulse Width	8.0000
Acquisition Time	1.3763
Acquisition Date	2023-07-25T13:49:23
Modification Date	2023-07-25T13:49:20
Spectrometer Frequency	100.64
Spectral Width	23809.5
Lowest Frequency	-1848.1
Nucleus	13C
Acquired Size	32768
Spectral Size	65536

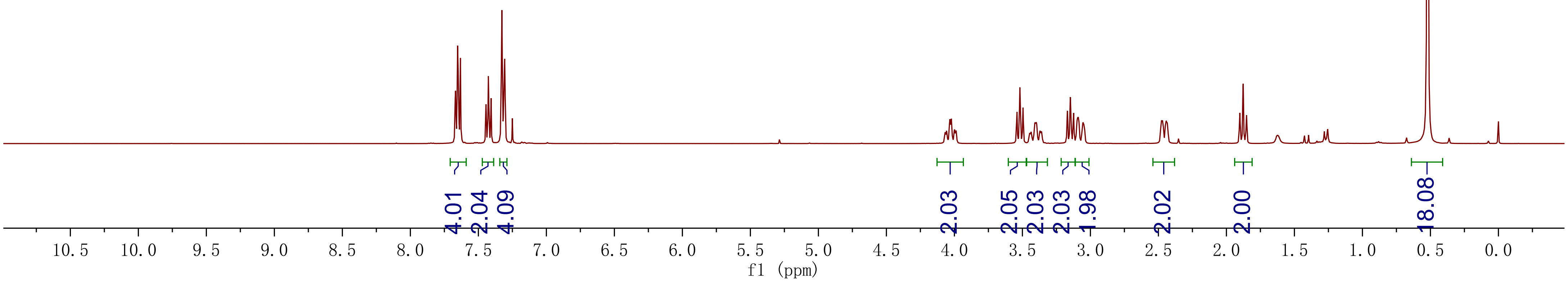

Parameter	Value
Title	dq-1-101-B-400m-h.7.fid
Origin	Bruker BioSpin GmbH
Owner	nmr
Instrument	AvanceNeo 400MHz
Solvent	CDCl3
Temperature	296.5
Pulse Sequence	zg30
Experiment	1D
Number of Scans	16
Receiver Gain	101.0
Relaxation Delay	1.0000
Pulse Width	8.0000
Acquisition Time	3.9977
Acquisition Date	2023-07-25T13:54:00
Modification Date	2023-07-25T13:53:58
Spectrometer Frequency	400.18
Spectral Width	8196.7
Lowest Frequency	-1640.7
Nucleus	1H
Acquired Size	32768
Spectral Size	65536

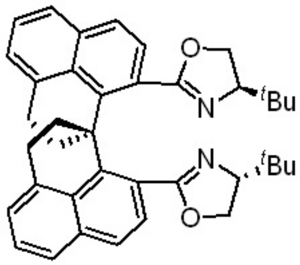

(S_S,R,R)-**L3**

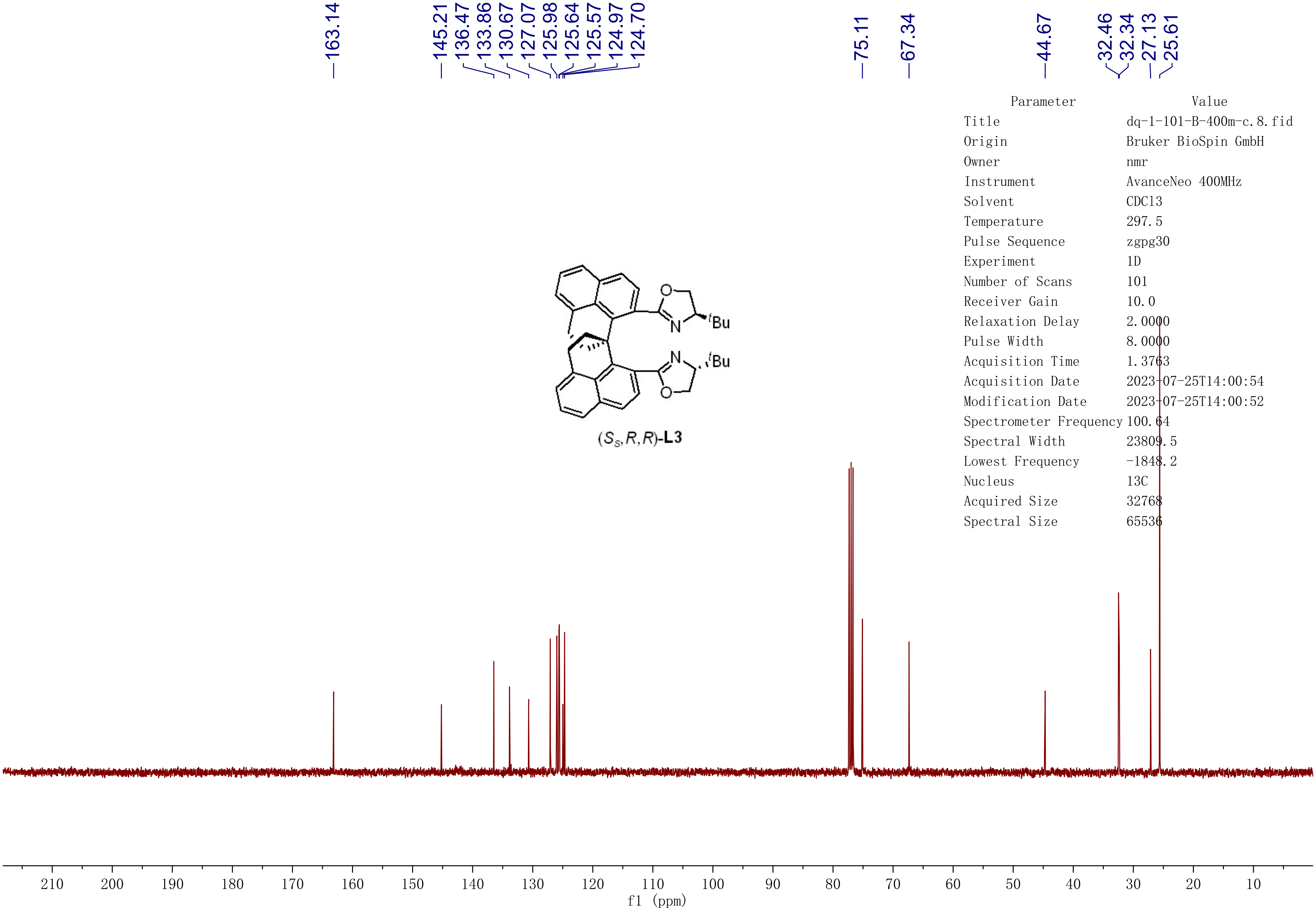

(S_S,R,R)-**L3**

Parameter	Value
Title	dq-1-101-B-400m-c.8.fid
Origin	Bruker BioSpin GmbH
Owner	nmr
Instrument	AvanceNeo 400MHz
Solvent	CDCl3
Temperature	297.5
Pulse Sequence	zgpg30
Experiment	1D
Number of Scans	101
Receiver Gain	10.0
Relaxation Delay	2.0000
Pulse Width	8.0000
Acquisition Time	1.3763
Acquisition Date	2023-07-25T14:00:54
Modification Date	2023-07-25T14:00:52
Spectrometer Frequency	100.64
Spectral Width	23809.5
Lowest Frequency	-1848.2
Nucleus	13C
Acquired Size	32768
Spectral Size	65536

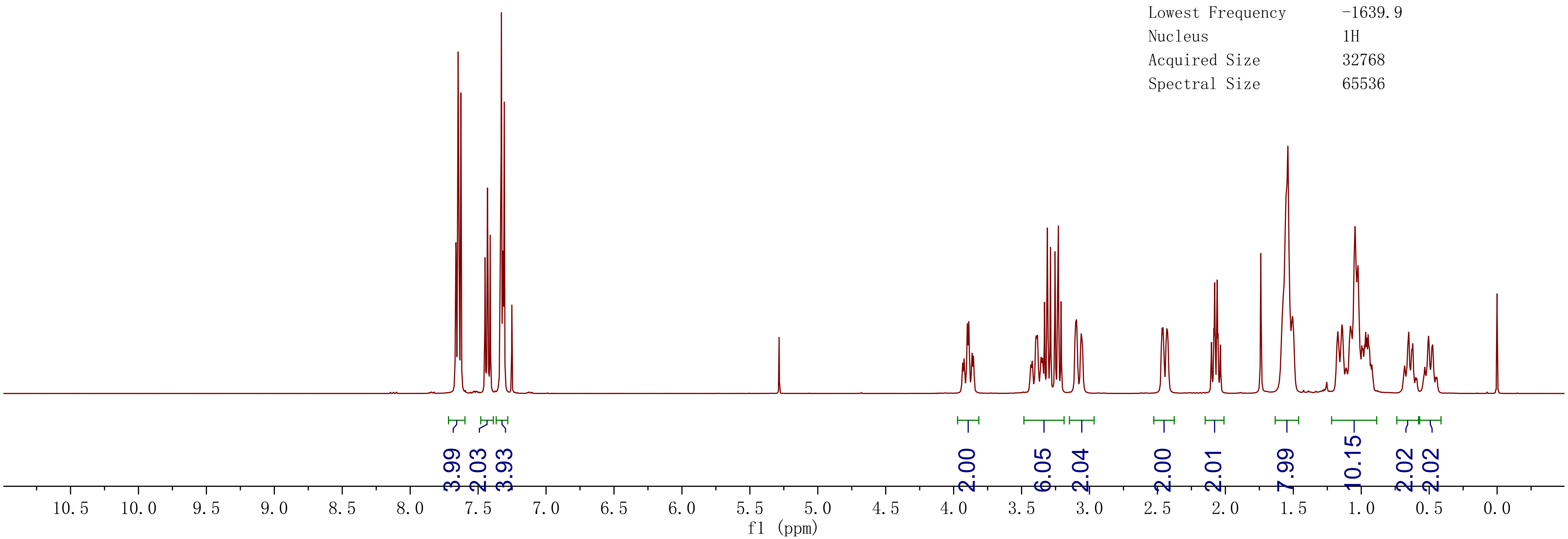

Parameter	Value
Title	dq-1-103-B-400m-h.1.fid
Origin	Bruker BioSpin GmbH
Owner	nmr
Instrument	AvanceNeo 400MHz
Solvent	CDCl3
Temperature	297.6
Pulse Sequence	zg30
Experiment	1D
Number of Scans	16
Receiver Gain	101.0
Relaxation Delay	1.0000
Pulse Width	8.0000
Acquisition Time	3.9977
Acquisition Date	2023-07-29T15:44:18
Modification Date	2023-07-29T15:44:12
Spectrometer Frequency	400.18
Spectral Width	8196.7
Lowest Frequency	-1639.9
Nucleus	1H
Acquired Size	32768
Spectral Size	65536

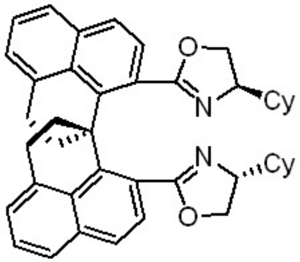

(S_S,R,R)-**L4**

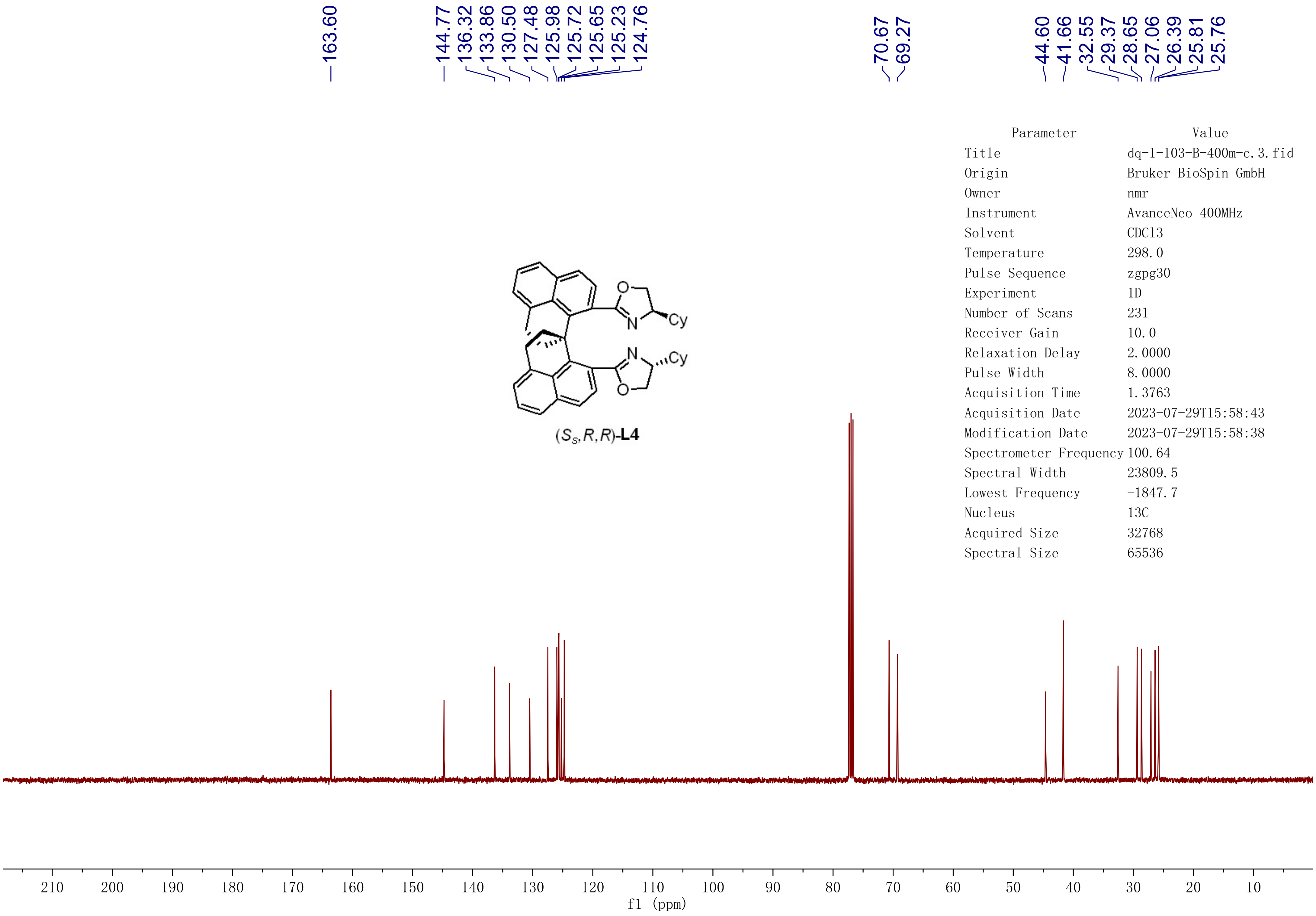

(S_S,R,R)-**L4**

Parameter	Value
Title	dq-1-103-B-400m-c.3.fid
Origin	Bruker BioSpin GmbH
Owner	nmr
Instrument	AvanceNeo 400MHz
Solvent	CDCl3
Temperature	298.0
Pulse Sequence	zgpg30
Experiment	1D
Number of Scans	231
Receiver Gain	10.0
Relaxation Delay	2.0000
Pulse Width	8.0000
Acquisition Time	1.3763
Acquisition Date	2023-07-29T15:58:43
Modification Date	2023-07-29T15:58:38
Spectrometer Frequency	100.64
Spectral Width	23809.5
Lowest Frequency	-1847.7
Nucleus	13C
Acquired Size	32768
Spectral Size	65536

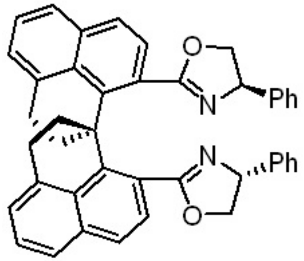

(S_S,R,R)-**L5**

Parameter	Value
Title	dq-1-100-A-400m-h.4.fid
Origin	Bruker BioSpin GmbH
Owner	nmr
Instrument	AvanceNeo 400MHz
Solvent	CDCl3
Temperature	296.3
Pulse Sequence	zg30
Experiment	1D
Number of Scans	16
Receiver Gain	101.0
Relaxation Delay	1.0000
Pulse Width	8.0000
Acquisition Time	3.9977
Acquisition Date	2023-07-25T13:35:10
Modification Date	2023-07-25T13:35:08
Spectrometer Frequency	400.18
Spectral Width	8196.7
Lowest Frequency	-1653.9
Nucleus	1H
Acquired Size	32768
Spectral Size	65536

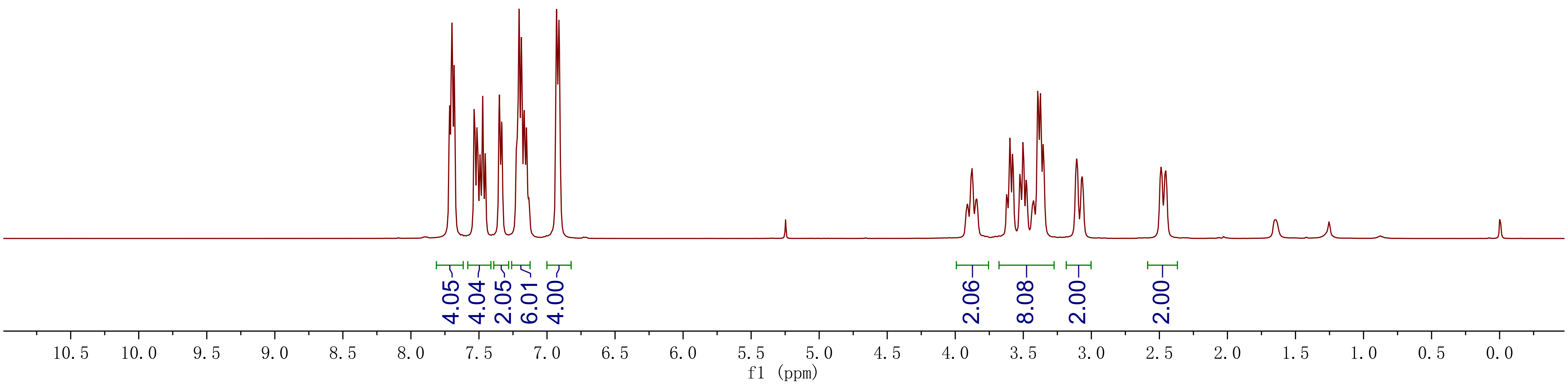

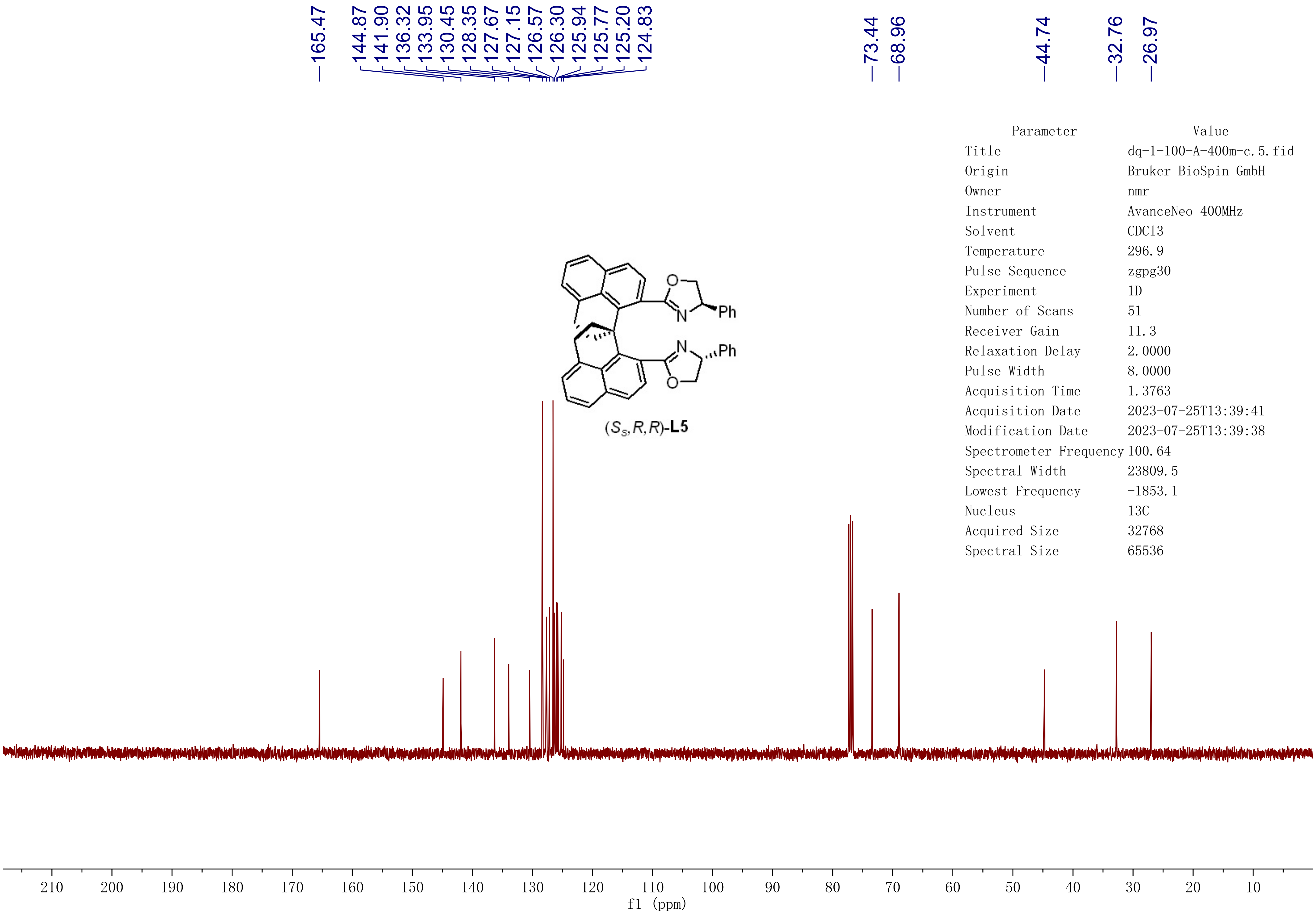

Parameter	Value
Title	dq-1-100-A-400m-c.5.fid
Origin	Bruker BioSpin GmbH
Owner	nmr
Instrument	AvanceNeo 400MHz
Solvent	CDCl3
Temperature	296.9
Pulse Sequence	zgpg30
Experiment	1D
Number of Scans	51
Receiver Gain	11.3
Relaxation Delay	2.0000
Pulse Width	8.0000
Acquisition Time	1.3763
Acquisition Date	2023-07-25T13:39:41
Modification Date	2023-07-25T13:39:38
Spectrometer Frequency	100.64
Spectral Width	23809.5
Lowest Frequency	-1853.1
Nucleus	13C
Acquired Size	32768
Spectral Size	65536

Parameter	Value
Title	dq-1-103-C-400m-h.1.fid
Origin	Bruker BioSpin GmbH
Owner	nmr
Instrument	AvanceNeo 400MHz
Solvent	CDCl3
Temperature	296.8
Pulse Sequence	zg30
Experiment	1D
Number of Scans	16
Receiver Gain	101.0
Relaxation Delay	1.0000
Pulse Width	8.0000
Acquisition Time	3.9977
Acquisition Date	2023-07-29T20:28:56
Modification Date	2023-07-29T20:28:50
Spectrometer Frequency	400.18
Spectral Width	8196.7
Lowest Frequency	-1647.5
Nucleus	1H
Acquired Size	32768
Spectral Size	65536

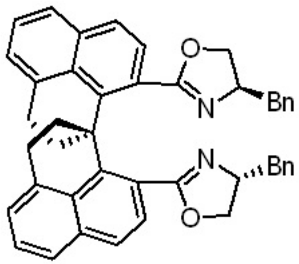

(S_S,R,R)-**L6**

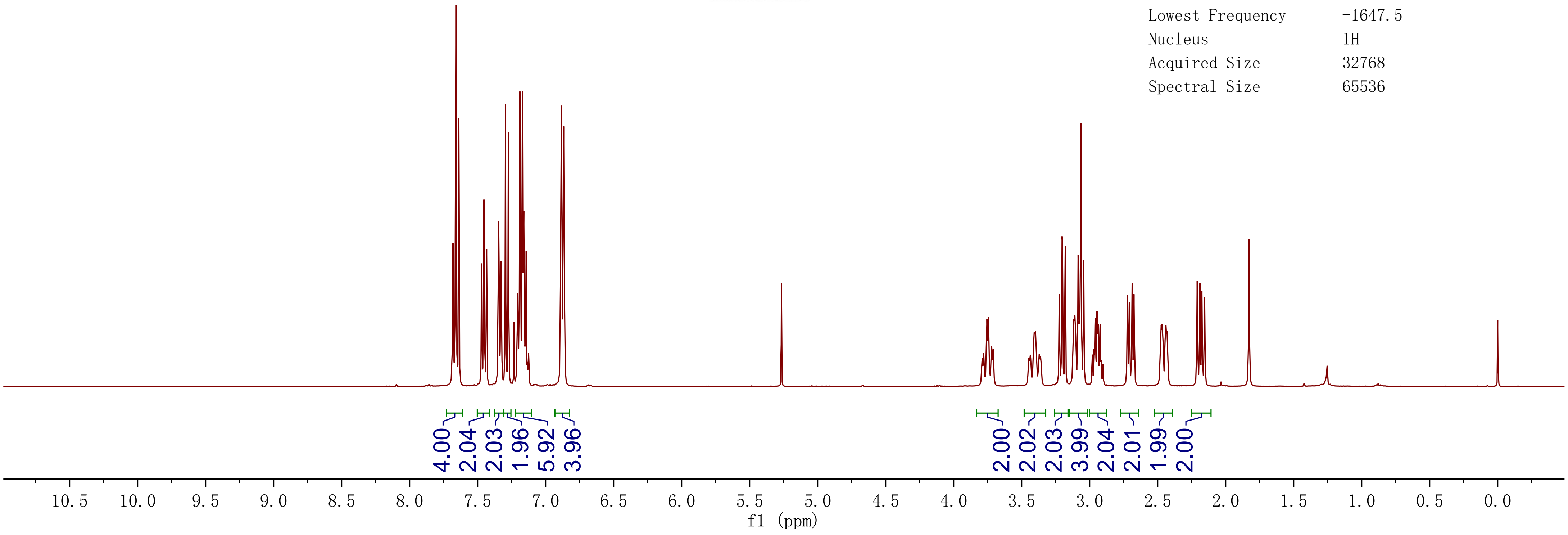

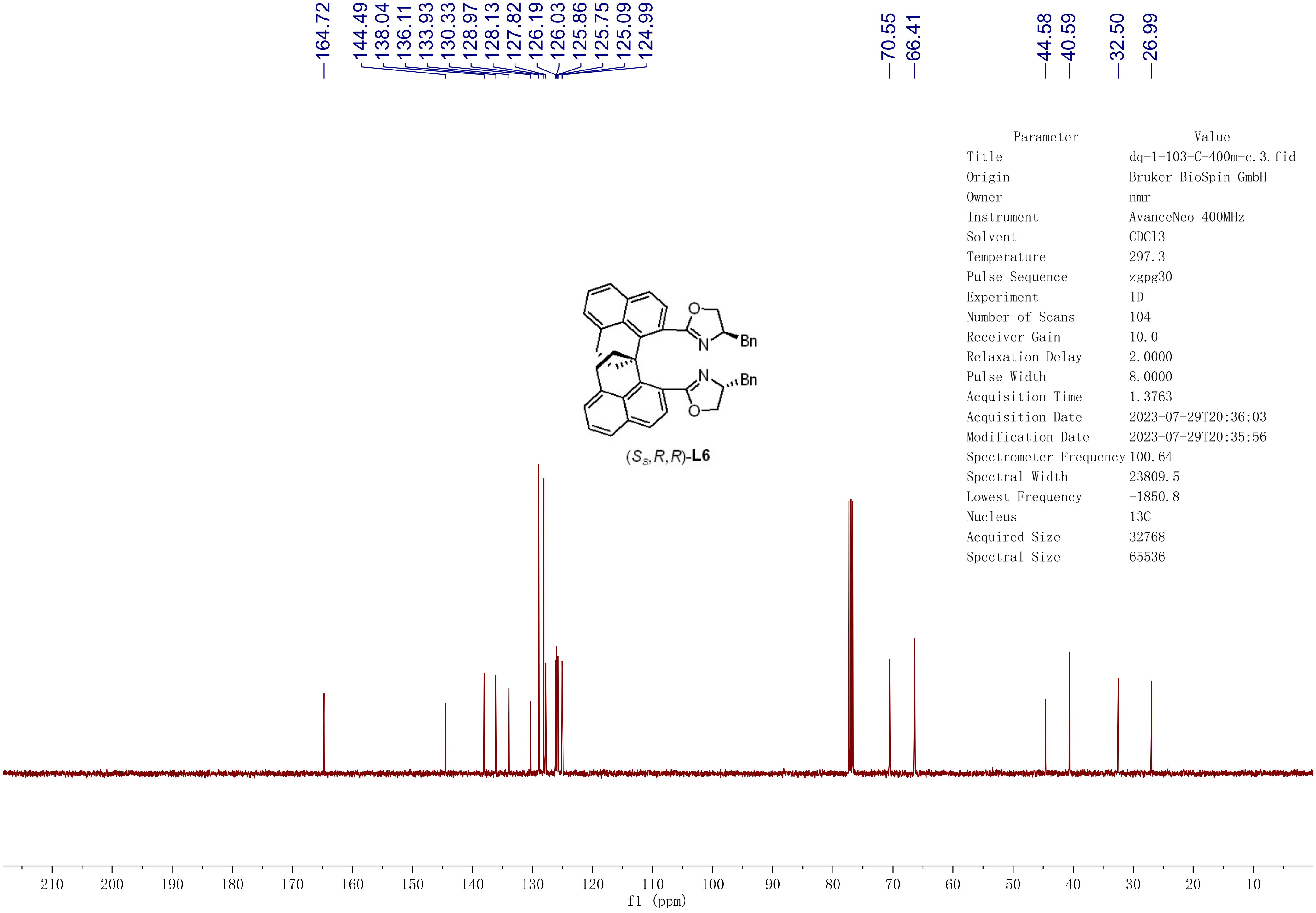

(S_S,R,R)-**L6**

Parameter	Value
Title	dq-1-103-C-400m-c.3.fid
Origin	Bruker BioSpin GmbH
Owner	nmr
Instrument	AvanceNeo 400MHz
Solvent	CDCl3
Temperature	297.3
Pulse Sequence	zgpg30
Experiment	1D
Number of Scans	104
Receiver Gain	10.0
Relaxation Delay	2.0000
Pulse Width	8.0000
Acquisition Time	1.3763
Acquisition Date	2023-07-29T20:36:03
Modification Date	2023-07-29T20:35:56
Spectrometer Frequency	100.64
Spectral Width	23809.5
Lowest Frequency	-1850.8
Nucleus	13C
Acquired Size	32768
Spectral Size	65536

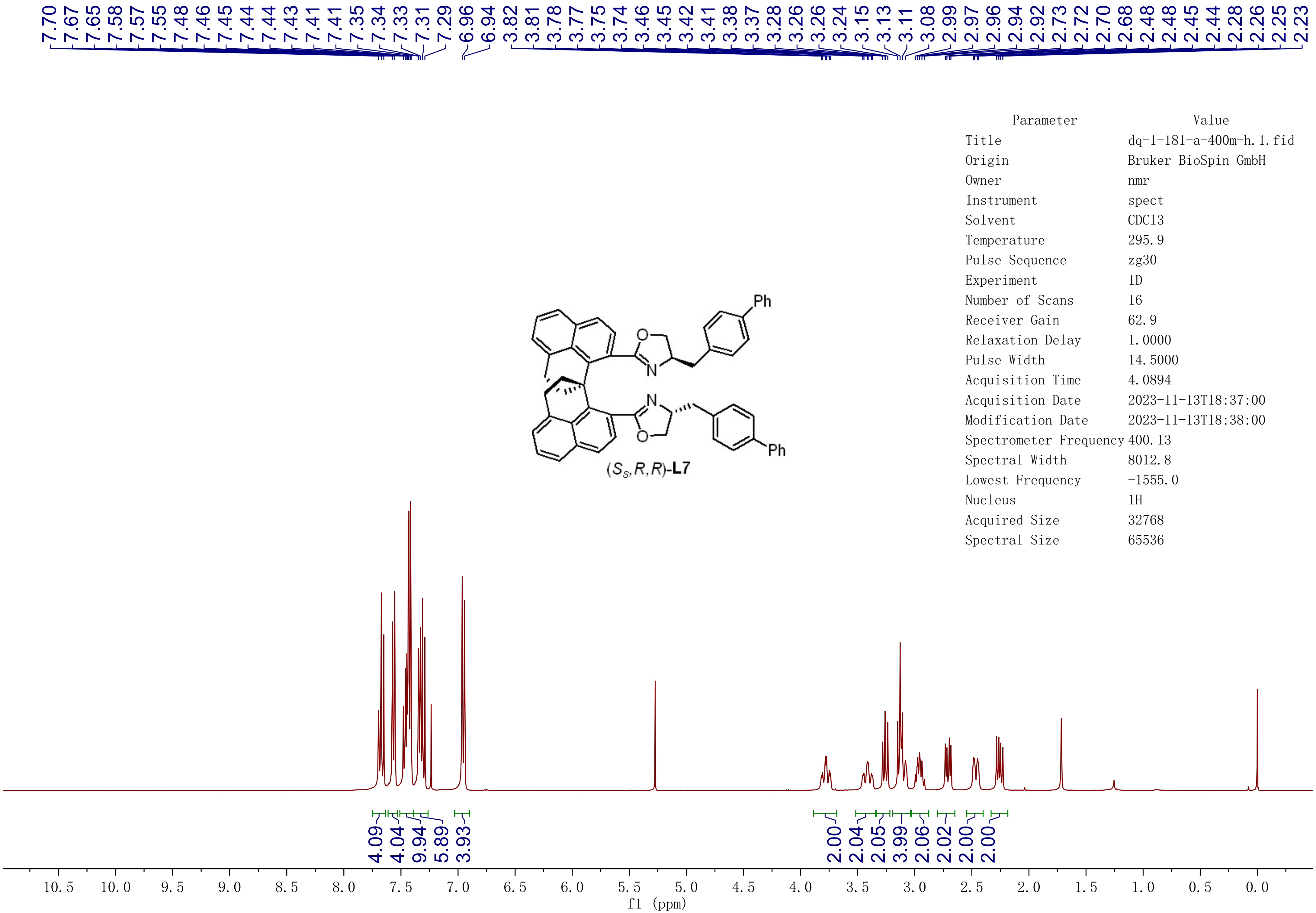

(S_S,*R*,*R*)-**L7**

Parameter	Value
Title	dq-1-181-a-400m-h.1.fid
Origin	Bruker BioSpin GmbH
Owner	nmr
Instrument	spect
Solvent	CDCl3
Temperature	295.9
Pulse Sequence	zg30
Experiment	1D
Number of Scans	16
Receiver Gain	62.9
Relaxation Delay	1.0000
Pulse Width	14.5000
Acquisition Time	4.0894
Acquisition Date	2023-11-13T18:37:00
Modification Date	2023-11-13T18:38:00
Spectrometer Frequency	400.13
Spectral Width	8012.8
Lowest Frequency	-1555.0
Nucleus	1H
Acquired Size	32768
Spectral Size	65536

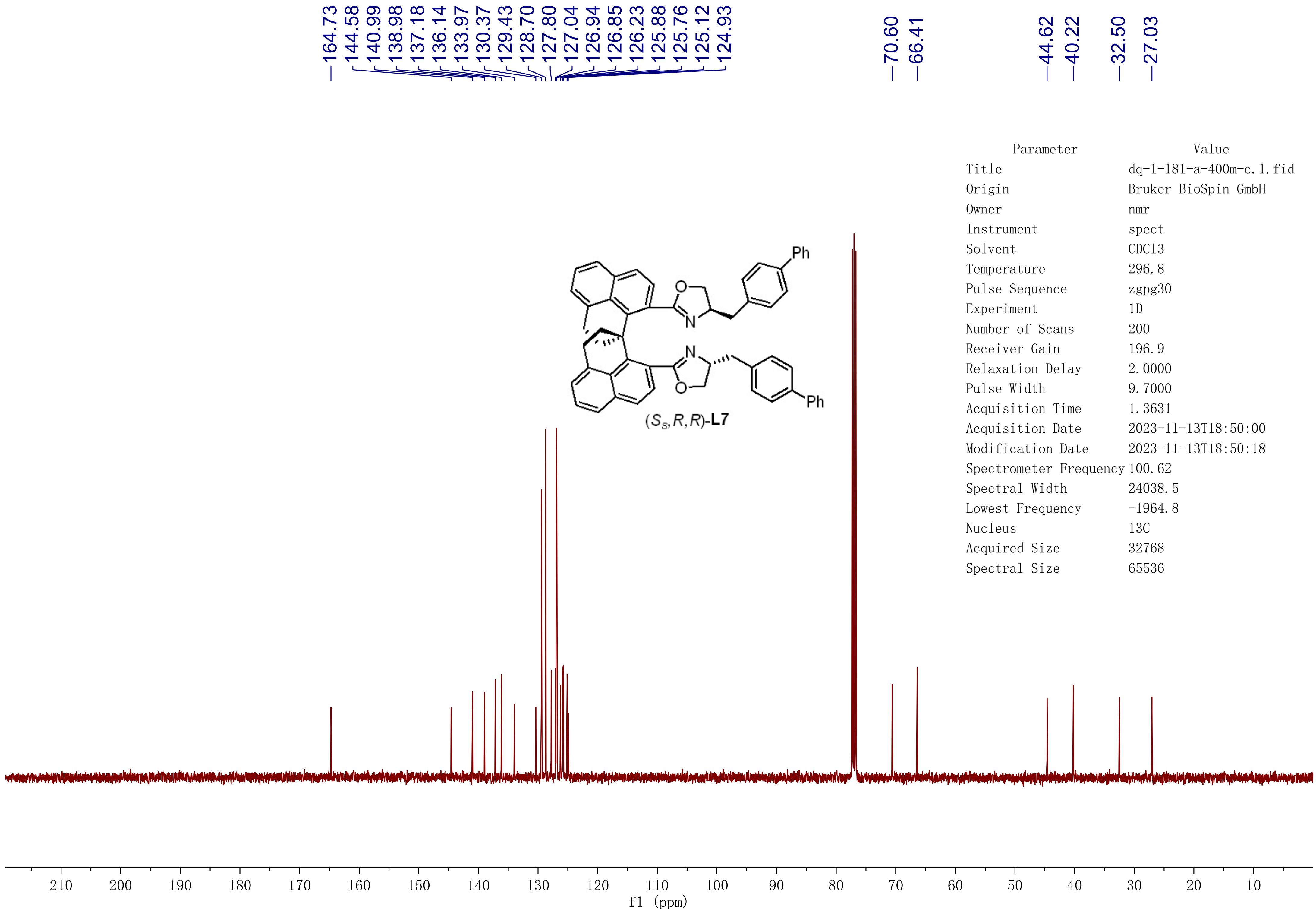

Parameter	Value
Title	dq-1-181-a-400m-c.1.fid
Origin	Bruker BioSpin GmbH
Owner	nmr
Instrument	spect
Solvent	CDCl3
Temperature	296.8
Pulse Sequence	zgpg30
Experiment	1D
Number of Scans	200
Receiver Gain	196.9
Relaxation Delay	2.0000
Pulse Width	9.7000
Acquisition Time	1.3631
Acquisition Date	2023-11-13T18:50:00
Modification Date	2023-11-13T18:50:18
Spectrometer Frequency	100.62
Spectral Width	24038.5
Lowest Frequency	-1964.8
Nucleus	13C
Acquired Size	32768
Spectral Size	65536

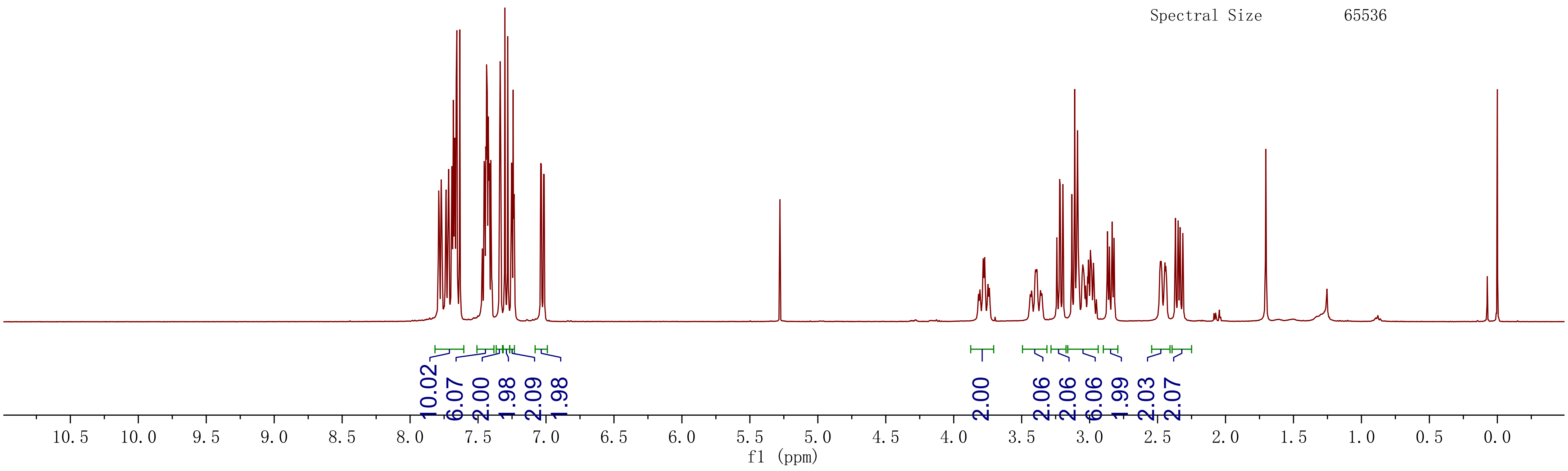

Parameter	Value
Title	dq-1-181-b-400m-h.1.fid
Origin	Bruker BioSpin GmbH
Owner	nmr
Instrument	spect
Solvent	CDCl3
Temperature	295.9
Pulse Sequence	zg30
Experiment	1D
Number of Scans	16
Receiver Gain	82.9
Relaxation Delay	1.0000
Pulse Width	14.5000
Acquisition Time	4.0894
Acquisition Date	2023-11-13T18:55:00
Modification Date	2023-11-13T18:55:12
Spectrometer Frequency	400.13
Spectral Width	8012.8
Lowest Frequency	-1552.6
Nucleus	1H
Acquired Size	32768
Spectral Size	65536

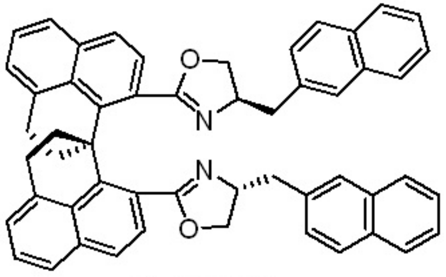

(S_S,R,R)-**L8**

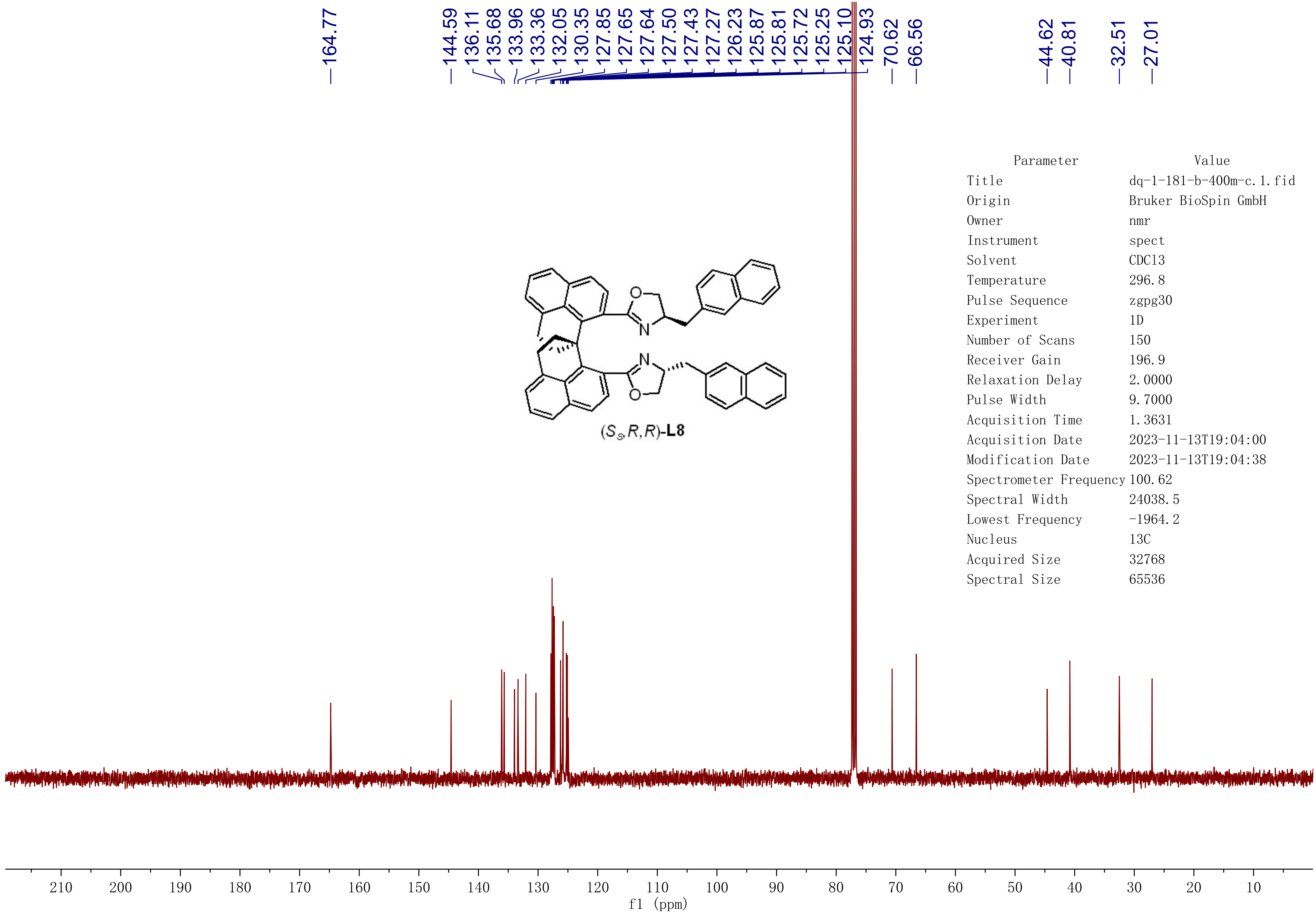
164.77
144.59
136.11
135.68
133.96
133.36
132.05
130.35
127.85
127.65
127.64
127.50
127.43
127.27
126.23
125.87
125.81
125.72
125.25
125.10
124.93
70.62
66.56
44.62
40.81
32.51
27.01
Parameter Value
Title dq-1-181-b-400m-c.1.fid
Origin Bruker BioSpin GmbH
Owner nmr
Instrument spect
Solvent CDCl3
Temperature 296.8
Pulse Sequence zgpg30
Experiment 1D
Number of Scans 150
Receiver Gain 196.9
Relaxation Delay 2.0000
Pulse Width 9.7000
Acquisition Time 1.3631
Acquisition Date 2023-11-13T19:04:00
Modification Date 2023-11-13T19:04:38
Spectrometer Frequency 100.62
Spectral Width 24038.5
Lowest Frequency -1964.2
Nucleus 13C
Acquired Size 32768
Spectral Size 65536
O
N
N
O
(S_S,R,R)-L8
210 200 190 180 170 160 150 140 130 120 110 100 90 80 70 60 50 40 30 20 10
f1 (ppm)

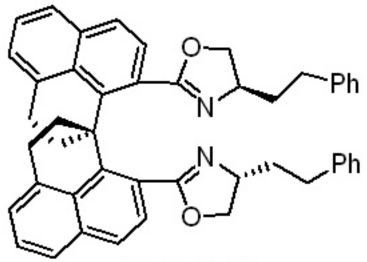

(S_S,R,R)-**L9**

Parameter	Value
Title	dq-1-181-c-400m-h.1.fid
Origin	Bruker BioSpin GmbH
Owner	nmr
Instrument	spect
Solvent	CDCl3
Temperature	295.9
Pulse Sequence	zg30
Experiment	1D
Number of Scans	16
Receiver Gain	71.0
Relaxation Delay	1.0000
Pulse Width	14.5000
Acquisition Time	4.0894
Acquisition Date	2023-11-13T19:09:00
Modification Date	2023-11-13T19:09:30
Spectrometer Frequency	400.13
Spectral Width	8012.8
Lowest Frequency	-1551.8
Nucleus	1H
Acquired Size	32768
Spectral Size	65536

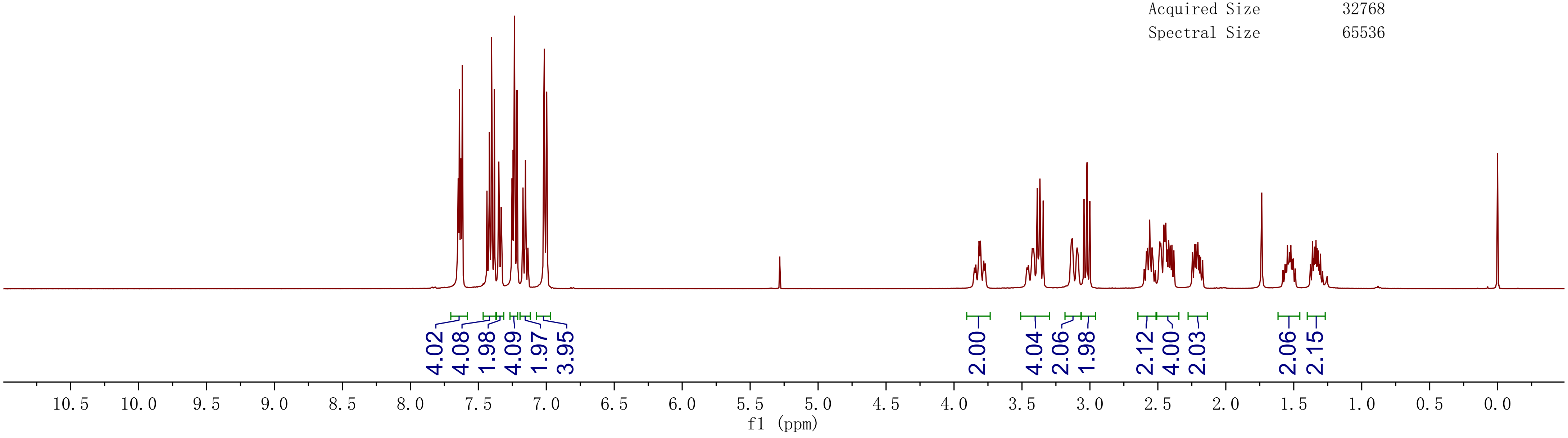

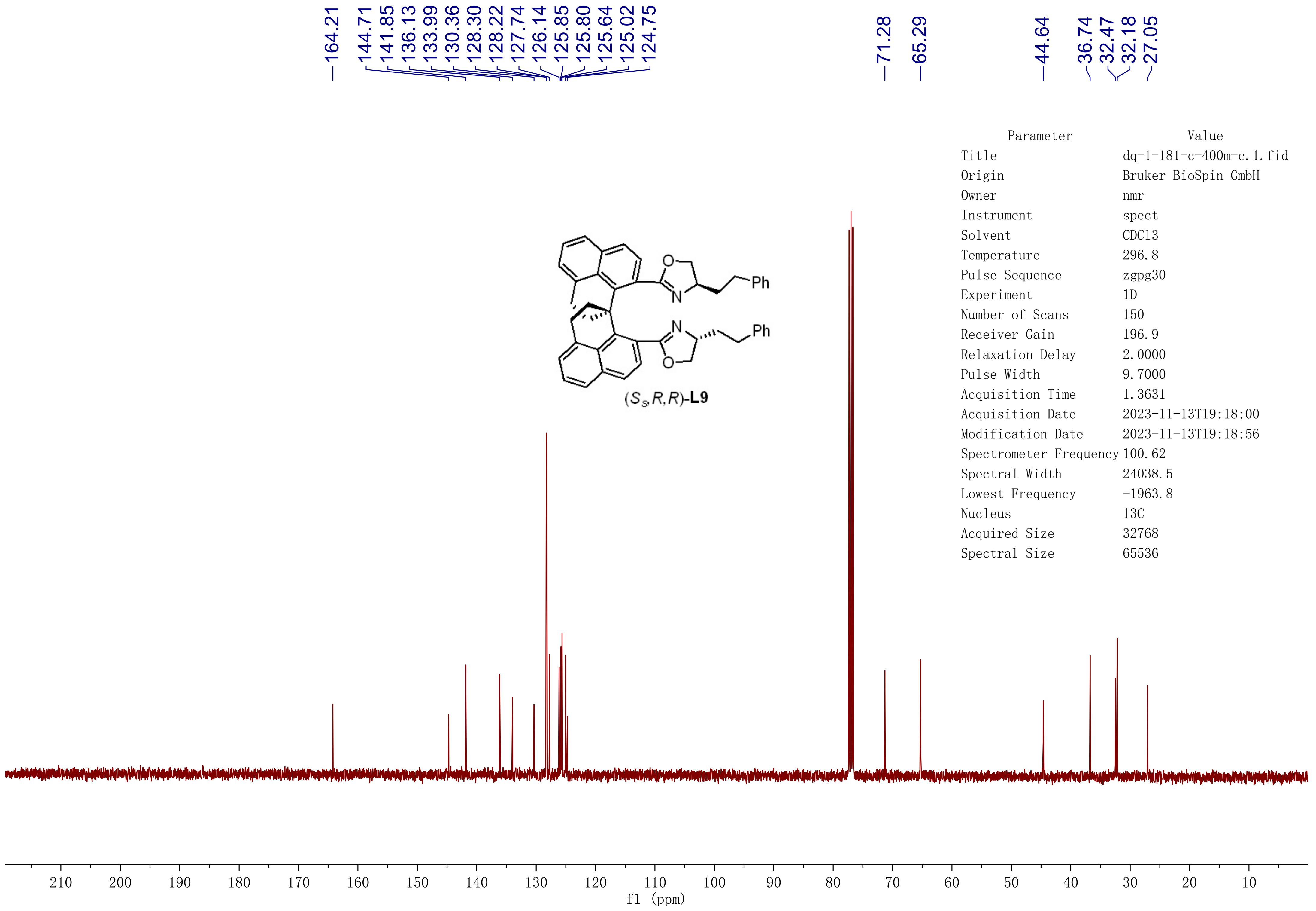

Parameter	Value
Title	dq-1-181-c-400m-c.1.fid
Origin	Bruker BioSpin GmbH
Owner	nmr
Instrument	spect
Solvent	CDCl3
Temperature	296.8
Pulse Sequence	zgpg30
Experiment	1D
Number of Scans	150
Receiver Gain	196.9
Relaxation Delay	2.0000
Pulse Width	9.7000
Acquisition Time	1.3631
Acquisition Date	2023-11-13T19:18:00
Modification Date	2023-11-13T19:18:56
Spectrometer Frequency	100.62
Spectral Width	24038.5
Lowest Frequency	-1963.8
Nucleus	13C
Acquired Size	32768
Spectral Size	65536

Parameter	Value
Title	qxk-RRRR-Bn-Ph.1.fid
Origin	Bruker BioSpin GmbH
Owner	nmr
Instrument	spect
Solvent	CDCl3
Temperature	292.0
Pulse Sequence	zg30
Experiment	1D
Number of Scans	16
Receiver Gain	203.0
Relaxation Delay	1.0000
Pulse Width	15.8000
Acquisition Time	3.9846
Acquisition Date	2025-04-07T19:15:37
Modification Date	2025-04-07T19:15:38
Spectrometer Frequency	400.13
Spectral Width	8223.7
Lowest Frequency	-1659.1
Nucleus	1H
Acquired Size	32768
Spectral Size	65536

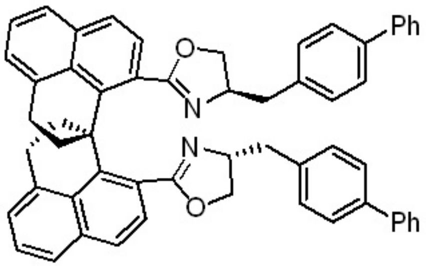

(R_S,R,R)-**L7'**

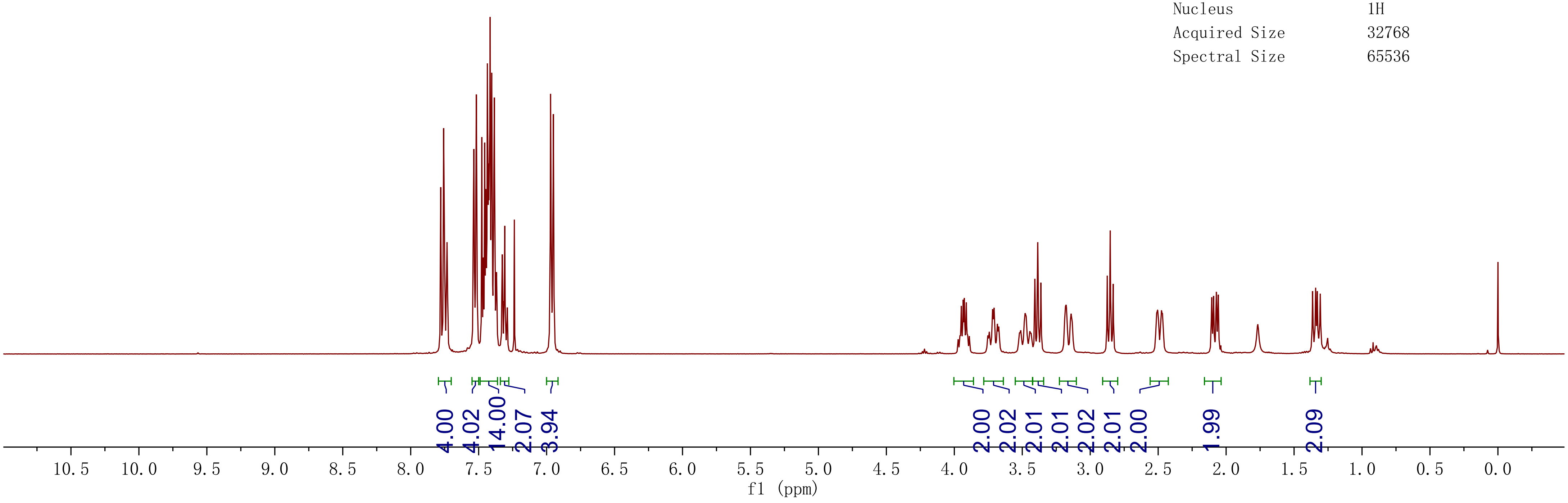

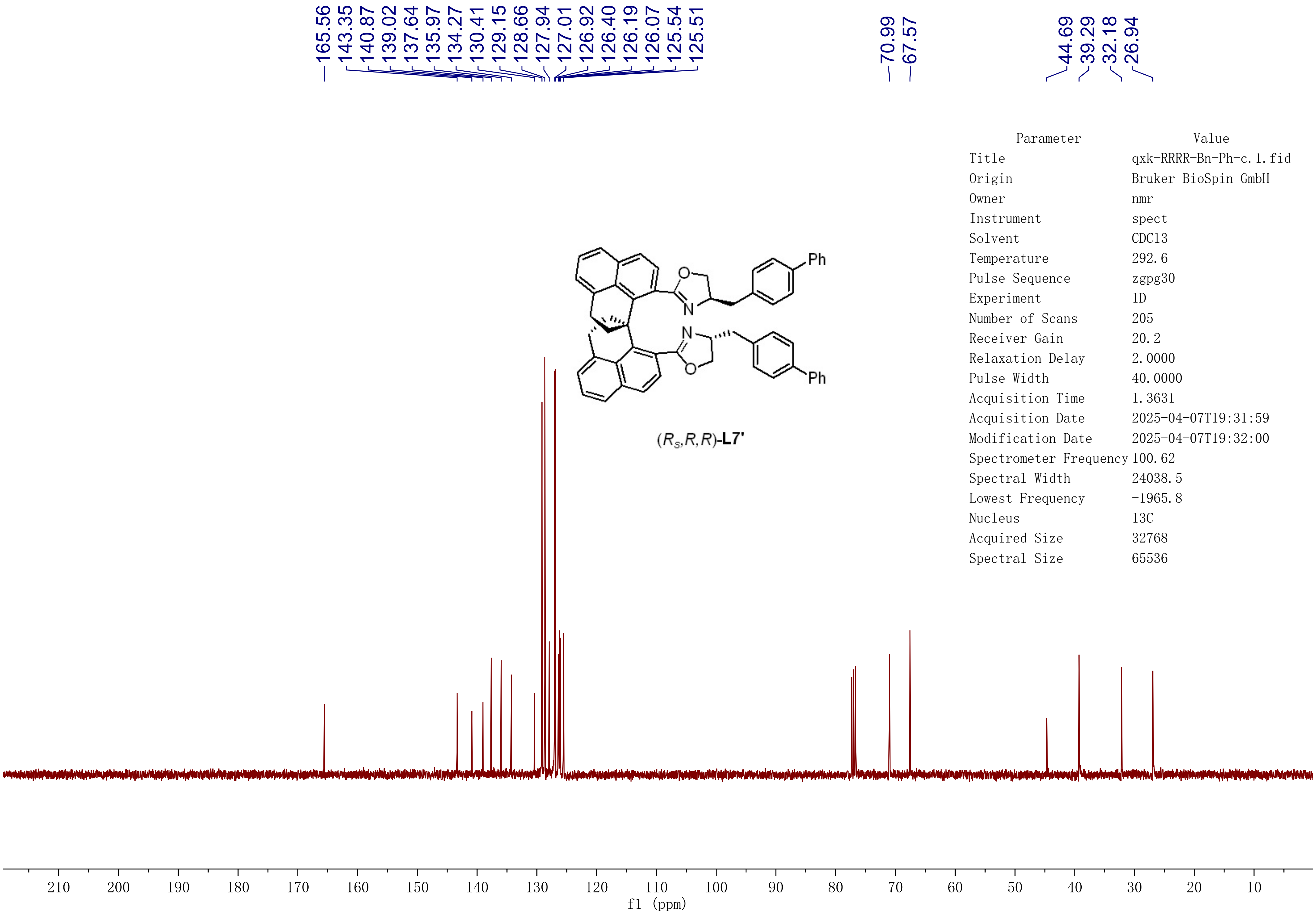

(R_S,R,R)-**L7'**

Parameter	Value
Title	qxk-RRRR-Bn-Ph-c.1.fid
Origin	Bruker BioSpin GmbH
Owner	nmr
Instrument	spect
Solvent	CDCl3
Temperature	292.6
Pulse Sequence	zgpg30
Experiment	1D
Number of Scans	205
Receiver Gain	20.2
Relaxation Delay	2.0000
Pulse Width	40.0000
Acquisition Time	1.3631
Acquisition Date	2025-04-07T19:31:59
Modification Date	2025-04-07T19:32:00
Spectrometer Frequency	100.62
Spectral Width	24038.5
Lowest Frequency	-1965.8
Nucleus	13C
Acquired Size	32768
Spectral Size	65536

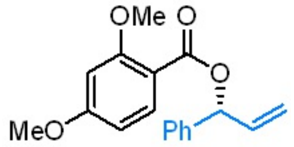

3a

Parameter	Value
Title	qxk-84-1h.1.fid
Origin	Bruker BioSpin GmbH
Owner	nmr
Instrument	spect
Solvent	CDCl3
Temperature	296.3
Pulse Sequence	zg30
Experiment	1D
Number of Scans	16
Receiver Gain	71.0
Relaxation Delay	1.0000
Pulse Width	14.5000
Acquisition Time	4.0894
Acquisition Date	2024-12-31T23:16:45
Modification Date	2024-12-31T23:16:48
Spectrometer Frequency	400.13
Spectral Width	8012.8
Lowest Frequency	-1545.4
Nucleus	1H
Acquired Size	32768
Spectral Size	65536

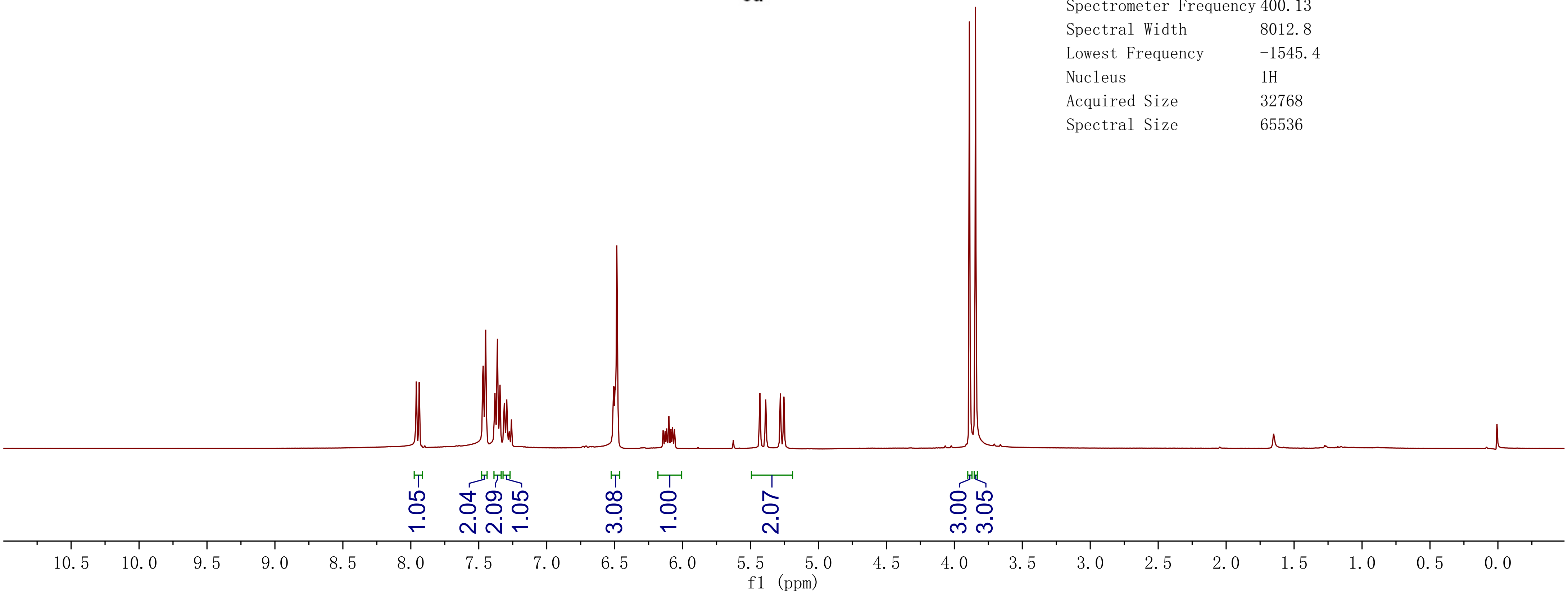

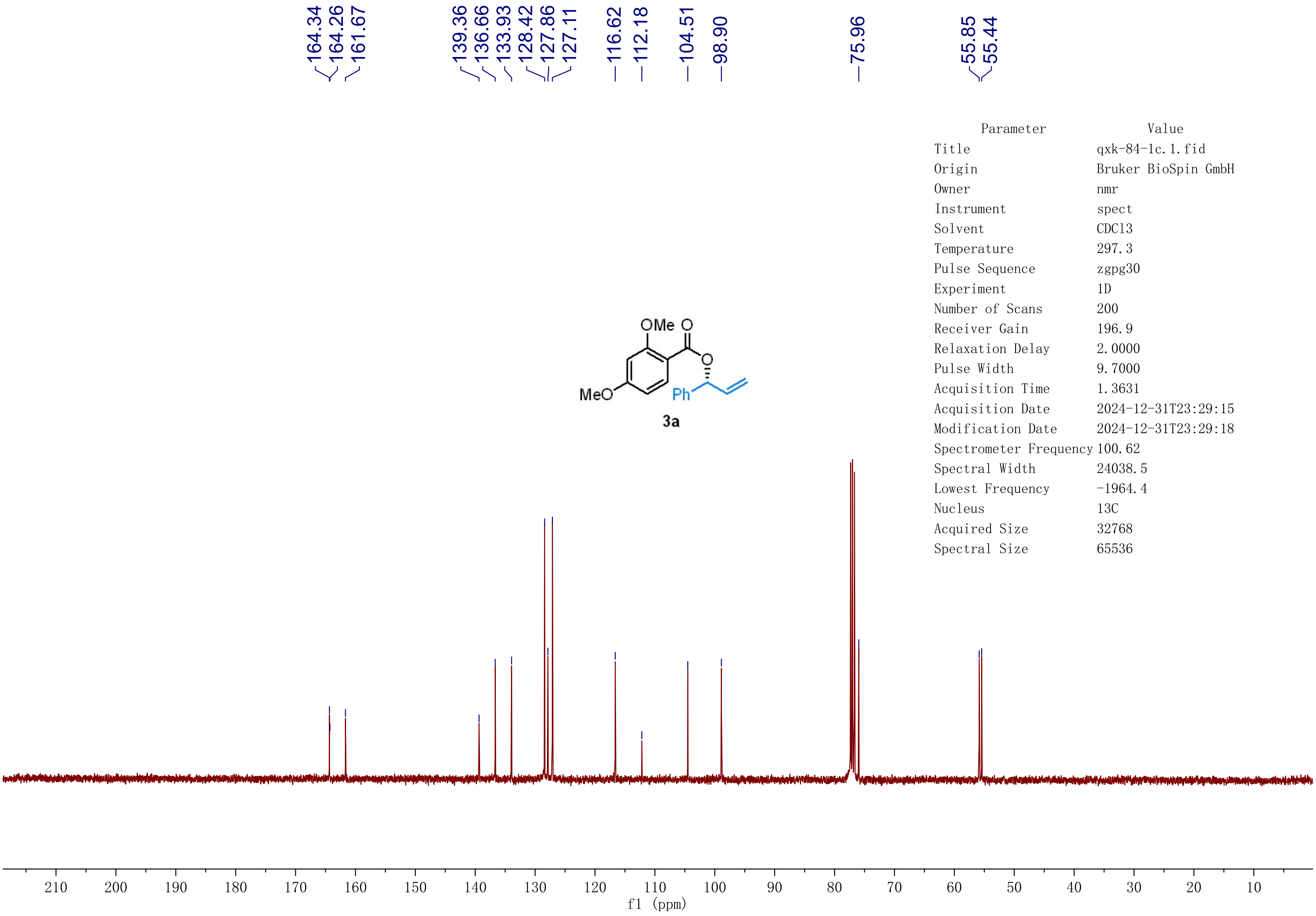

3a

Parameter	Value
Title	qxk-84-1c.1.fid
Origin	Bruker BioSpin GmbH
Owner	nmr
Instrument	spect
Solvent	CDCl3
Temperature	297.3
Pulse Sequence	zgpg30
Experiment	1D
Number of Scans	200
Receiver Gain	196.9
Relaxation Delay	2.0000
Pulse Width	9.7000
Acquisition Time	1.3631
Acquisition Date	2024-12-31T23:29:15
Modification Date	2024-12-31T23:29:18
Spectrometer Frequency	100.62
Spectral Width	24038.5
Lowest Frequency	-1964.4
Nucleus	13C
Acquired Size	32768
Spectral Size	65536

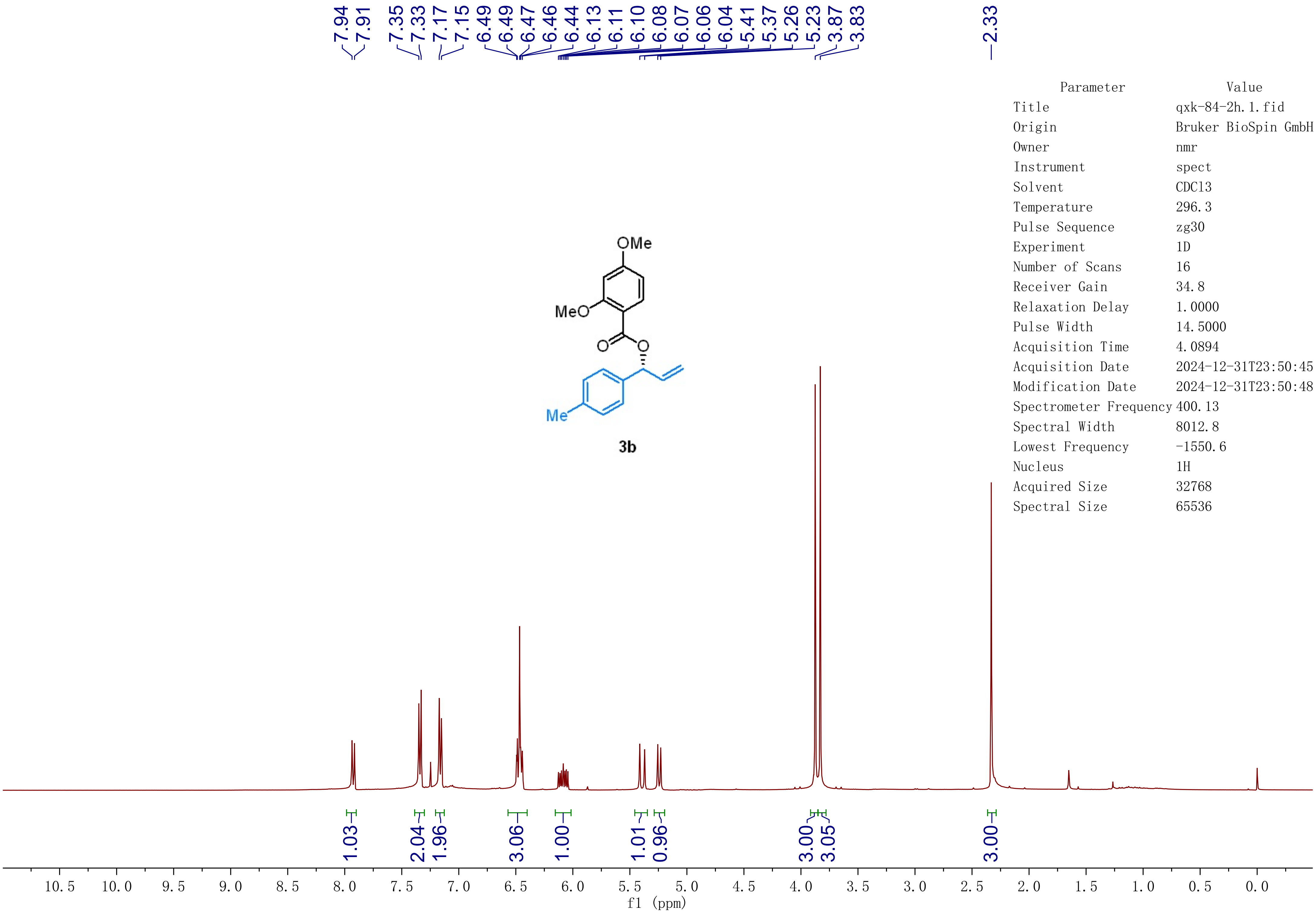

Parameter	Value
Title	qxk-84-2h.1.fid
Origin	Bruker BioSpin GmbH
Owner	nmr
Instrument	spect
Solvent	CDCl3
Temperature	296.3
Pulse Sequence	zg30
Experiment	1D
Number of Scans	16
Receiver Gain	34.8
Relaxation Delay	1.0000
Pulse Width	14.5000
Acquisition Time	4.0894
Acquisition Date	2024-12-31T23:50:45
Modification Date	2024-12-31T23:50:48
Spectrometer Frequency	400.13
Spectral Width	8012.8
Lowest Frequency	-1550.6
Nucleus	1H
Acquired Size	32768
Spectral Size	65536

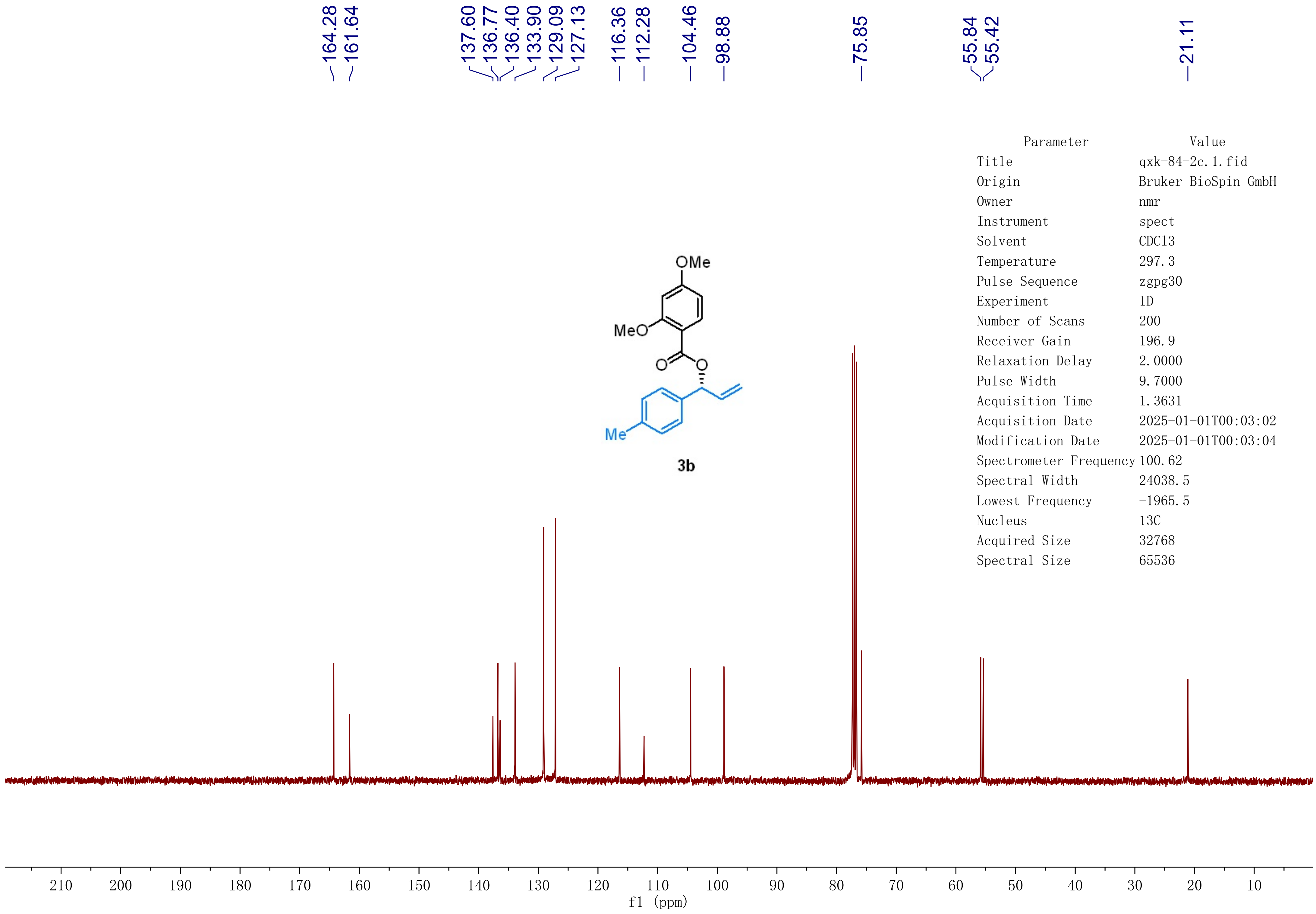

Parameter	Value
Title	qxk-84-2c.1.fid
Origin	Bruker BioSpin GmbH
Owner	nmr
Instrument	spect
Solvent	CDCl3
Temperature	297.3
Pulse Sequence	zgpg30
Experiment	1D
Number of Scans	200
Receiver Gain	196.9
Relaxation Delay	2.0000
Pulse Width	9.7000
Acquisition Time	1.3631
Acquisition Date	2025-01-01T00:03:02
Modification Date	2025-01-01T00:03:04
Spectrometer Frequency	100.62
Spectral Width	24038.5
Lowest Frequency	-1965.5
Nucleus	13C
Acquired Size	32768
Spectral Size	65536

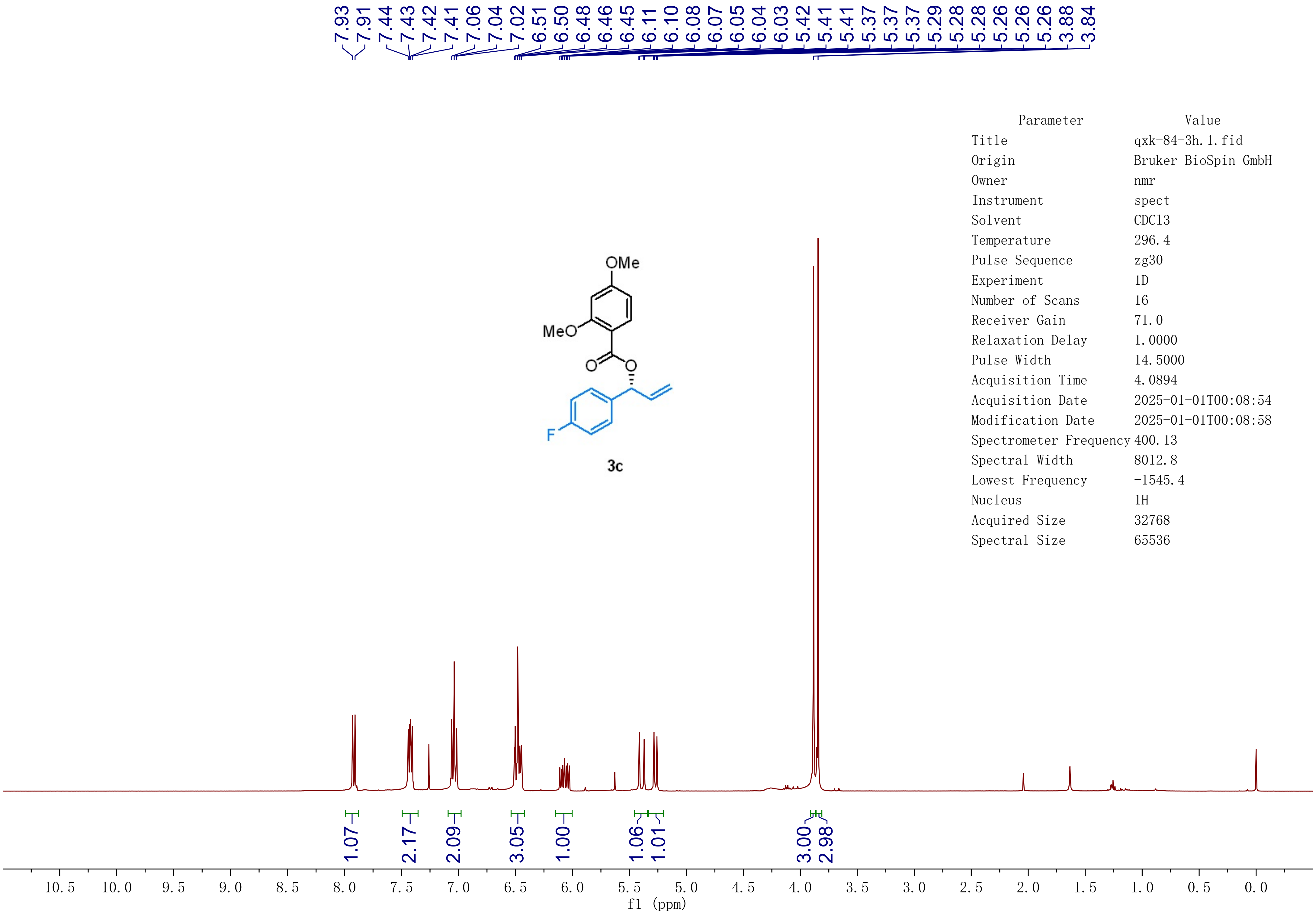

3c

Parameter	Value
Title	qxk-84-3h.1.fid
Origin	Bruker BioSpin GmbH
Owner	nmr
Instrument	spect
Solvent	CDCl3
Temperature	296.4
Pulse Sequence	zg30
Experiment	1D
Number of Scans	16
Receiver Gain	71.0
Relaxation Delay	1.0000
Pulse Width	14.5000
Acquisition Time	4.0894
Acquisition Date	2025-01-01T00:08:54
Modification Date	2025-01-01T00:08:58
Spectrometer Frequency	400.13
Spectral Width	8012.8
Lowest Frequency	-1545.4
Nucleus	1H
Acquired Size	32768
Spectral Size	65536

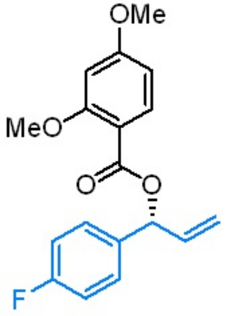

3c

Parameter	Value
Title	qxk-84-3f.1.fid
Origin	Bruker BioSpin GmbH
Owner	nmr
Instrument	spect
Solvent	CDCl3
Temperature	296.8
Pulse Sequence	zgflqn
Experiment	1D
Number of Scans	16
Receiver Gain	196.9
Relaxation Delay	1.0000
Pulse Width	14.7000
Acquisition Time	0.7340
Acquisition Date	2025-01-01T00:22:44
Modification Date	2025-01-01T00:22:48
Spectrometer Frequency	376.46
Spectral Width	89285.7
Lowest Frequency	-82292.5
Nucleus	19F
Acquired Size	65536
Spectral Size	131072

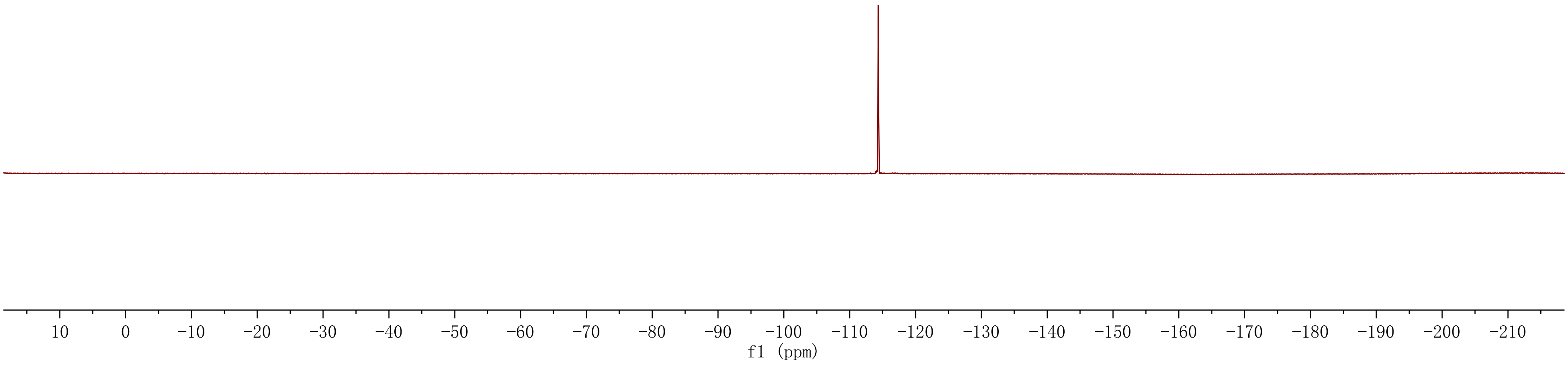

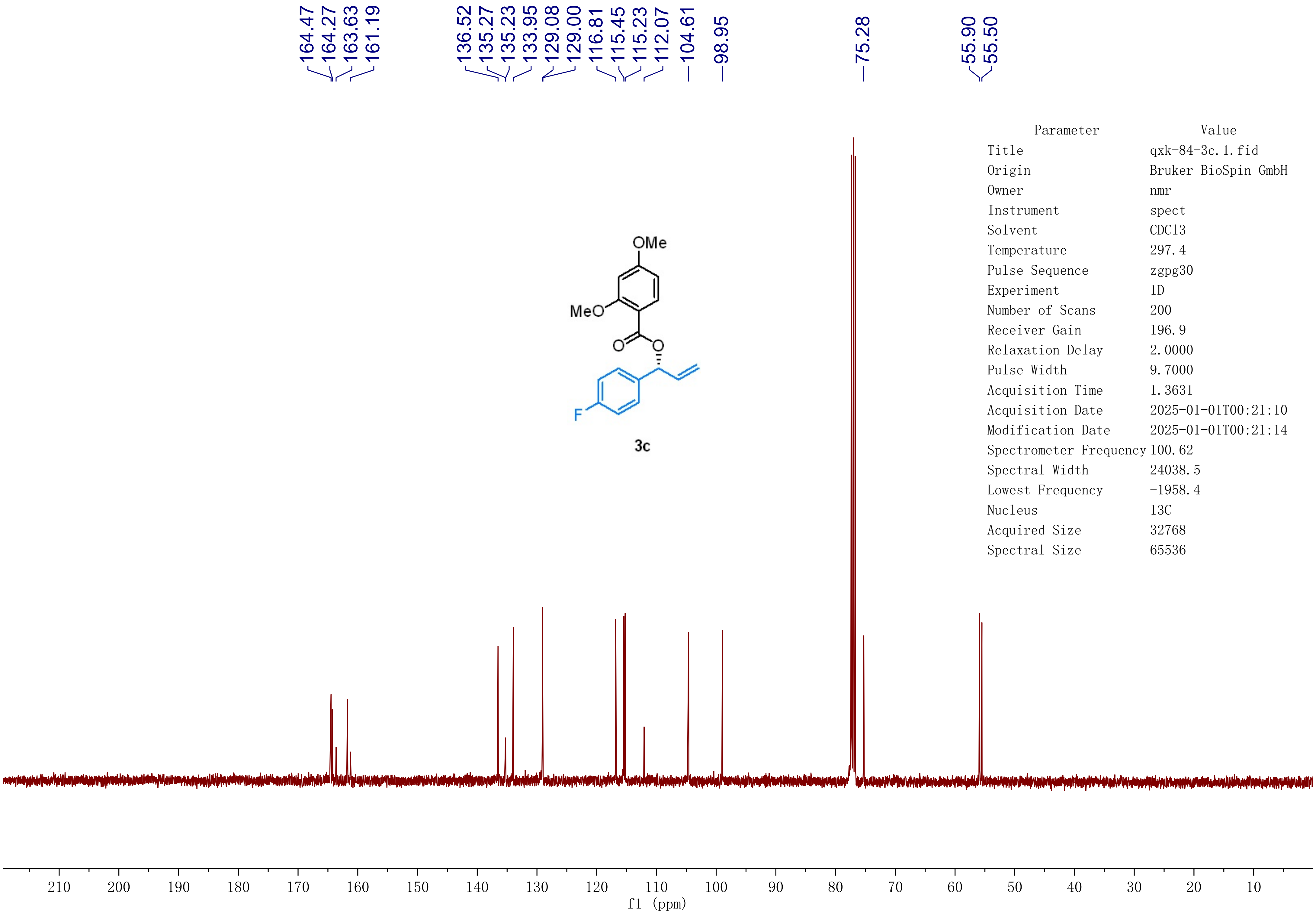

3c

Parameter	Value
Title	qxk-84-3c.1.fid
Origin	Bruker BioSpin GmbH
Owner	nmr
Instrument	spect
Solvent	CDCl3
Temperature	297.4
Pulse Sequence	zgpg30
Experiment	1D
Number of Scans	200
Receiver Gain	196.9
Relaxation Delay	2.0000
Pulse Width	9.7000
Acquisition Time	1.3631
Acquisition Date	2025-01-01T00:21:10
Modification Date	2025-01-01T00:21:14
Spectrometer Frequency	100.62
Spectral Width	24038.5
Lowest Frequency	-1958.4
Nucleus	13C
Acquired Size	32768
Spectral Size	65536

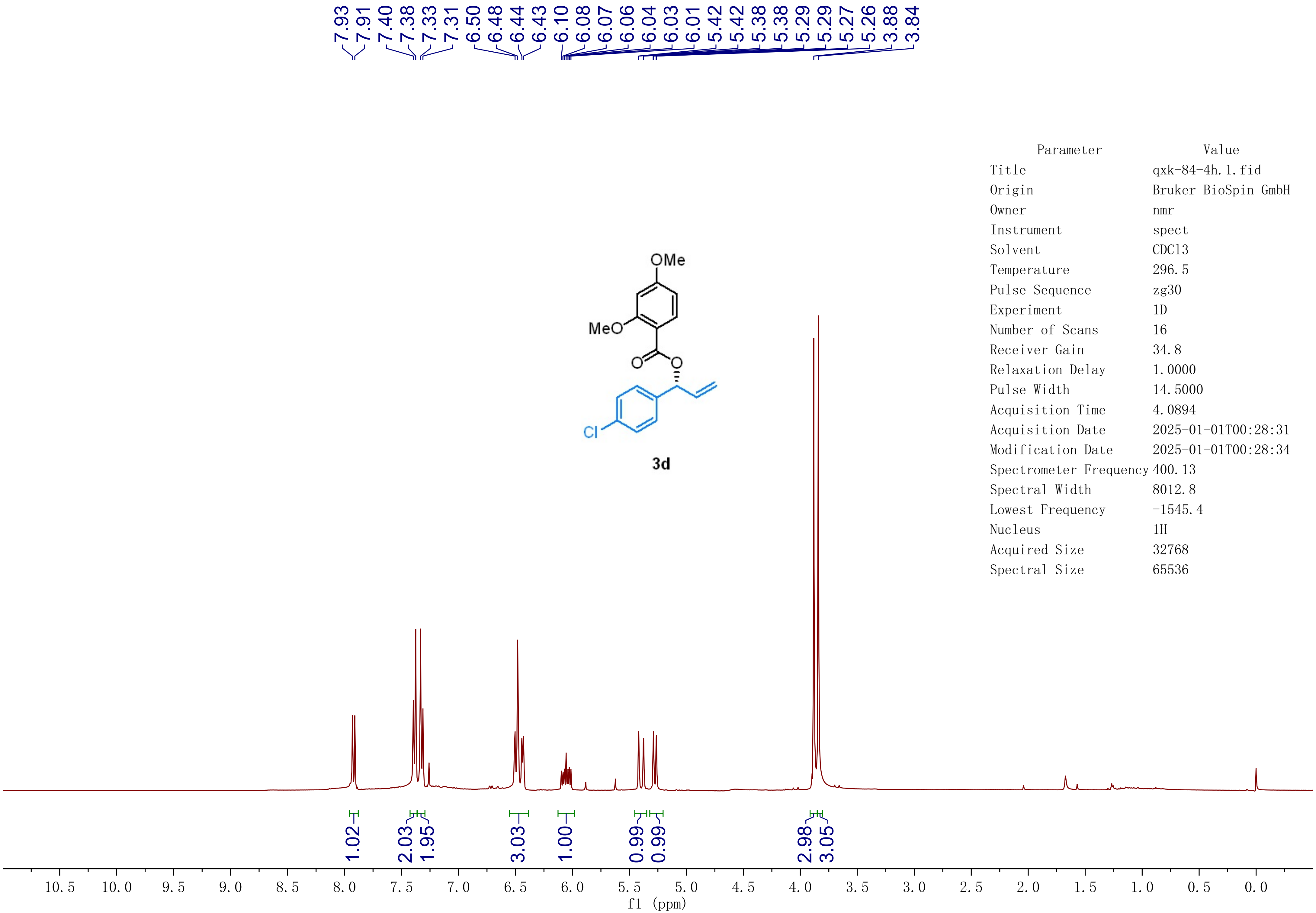

Parameter	Value
Title	qxk-84-4h.1.fid
Origin	Bruker BioSpin GmbH
Owner	nmr
Instrument	spect
Solvent	CDCl3
Temperature	296.5
Pulse Sequence	zg30
Experiment	1D
Number of Scans	16
Receiver Gain	34.8
Relaxation Delay	1.0000
Pulse Width	14.5000
Acquisition Time	4.0894
Acquisition Date	2025-01-01T00:28:31
Modification Date	2025-01-01T00:28:34
Spectrometer Frequency	400.13
Spectral Width	8012.8
Lowest Frequency	-1545.4
Nucleus	1H
Acquired Size	32768
Spectral Size	65536

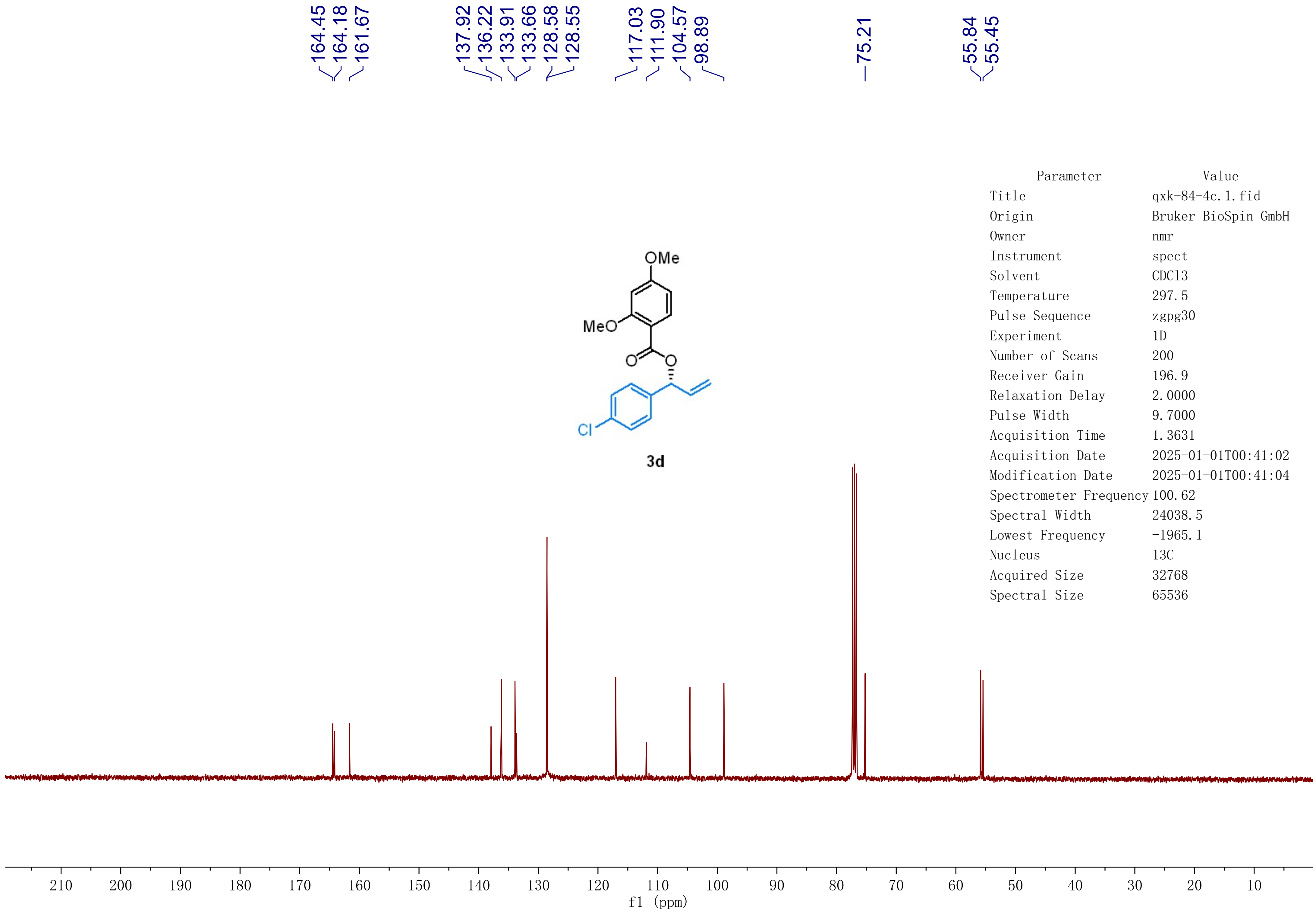

3d

Parameter	Value
Title	qxk-84-4c.1.fid
Origin	Bruker BioSpin GmbH
Owner	nmr
Instrument	spect
Solvent	CDCl3
Temperature	297.5
Pulse Sequence	zgpg30
Experiment	1D
Number of Scans	200
Receiver Gain	196.9
Relaxation Delay	2.0000
Pulse Width	9.7000
Acquisition Time	1.3631
Acquisition Date	2025-01-01T00:41:02
Modification Date	2025-01-01T00:41:04
Spectrometer Frequency	100.62
Spectral Width	24038.5
Lowest Frequency	-1965.1
Nucleus	13C
Acquired Size	32768
Spectral Size	65536

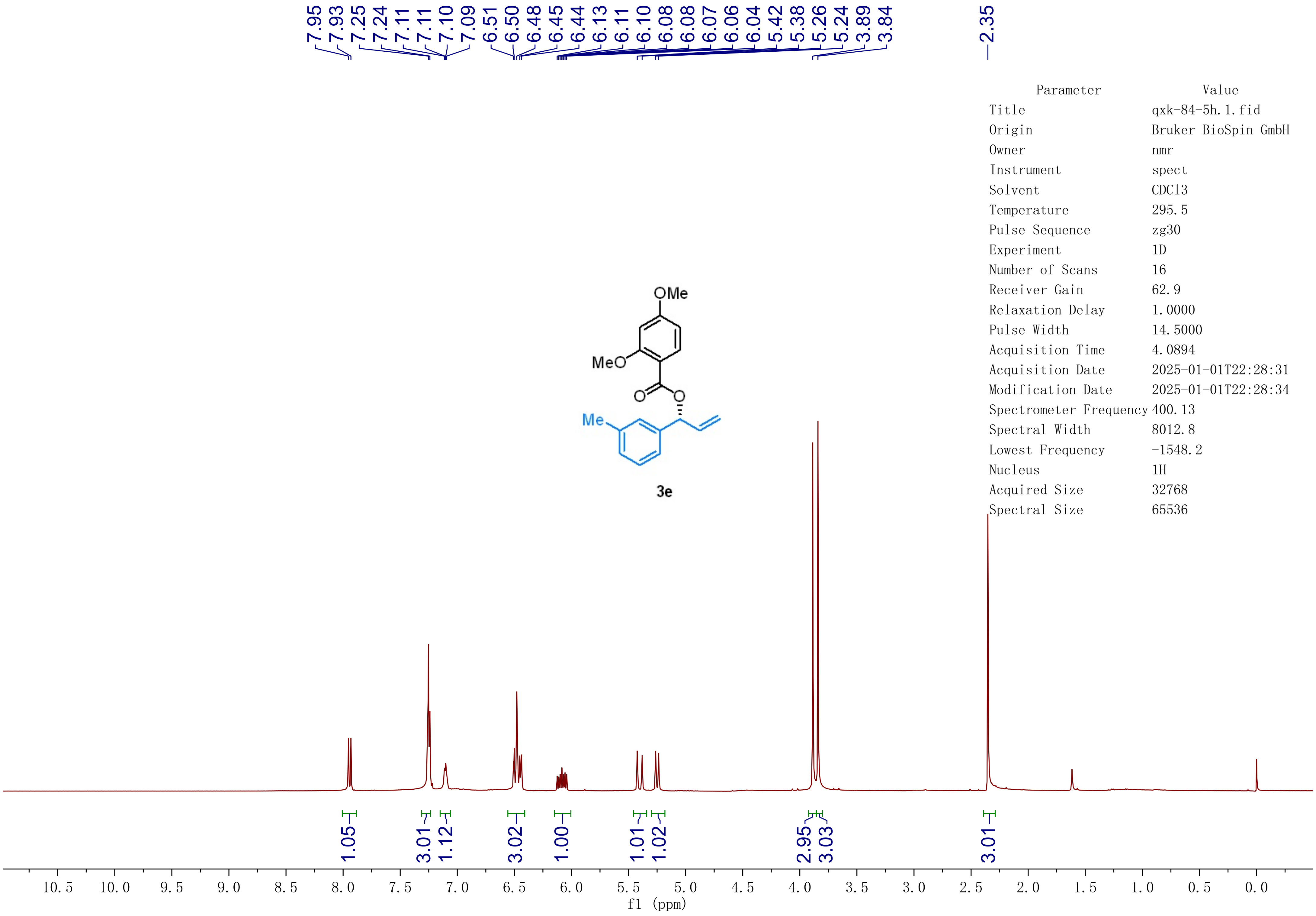

3e

Parameter	Value
Title	qxk-84-5h.1.fid
Origin	Bruker BioSpin GmbH
Owner	nmr
Instrument	spect
Solvent	CDCl3
Temperature	295.5
Pulse Sequence	zg30
Experiment	1D
Number of Scans	16
Receiver Gain	62.9
Relaxation Delay	1.0000
Pulse Width	14.5000
Acquisition Time	4.0894
Acquisition Date	2025-01-01T22:28:31
Modification Date	2025-01-01T22:28:34
Spectrometer Frequency	400.13
Spectral Width	8012.8
Lowest Frequency	-1548.2
Nucleus	1H
Acquired Size	32768
Spectral Size	65536

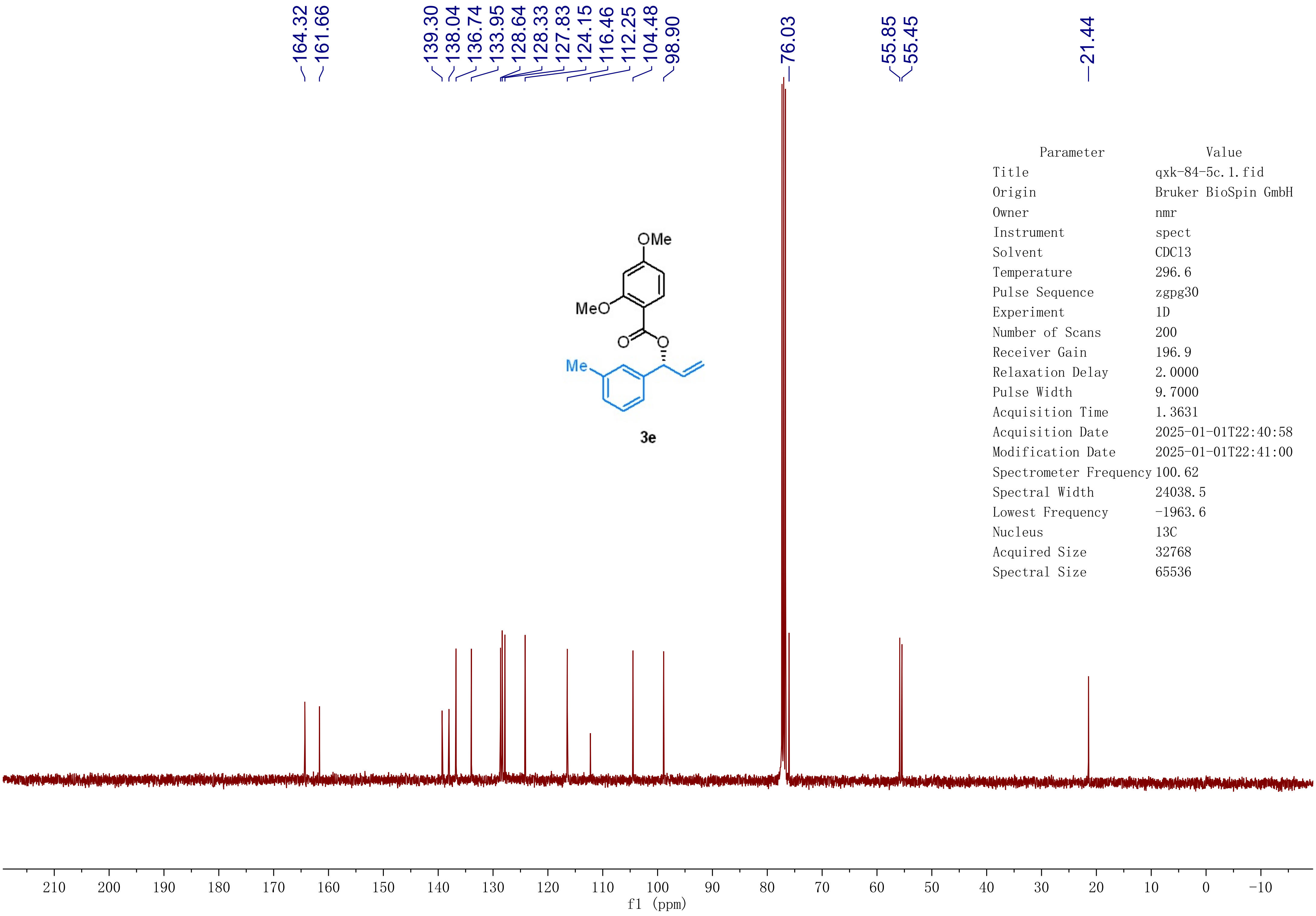

3e

Parameter	Value
Title	qxk-84-5c.1.fid
Origin	Bruker BioSpin GmbH
Owner	nmr
Instrument	spect
Solvent	CDCl3
Temperature	296.6
Pulse Sequence	zgpg30
Experiment	1D
Number of Scans	200
Receiver Gain	196.9
Relaxation Delay	2.0000
Pulse Width	9.7000
Acquisition Time	1.3631
Acquisition Date	2025-01-01T22:40:58
Modification Date	2025-01-01T22:41:00
Spectrometer Frequency	100.62
Spectral Width	24038.5
Lowest Frequency	-1963.6
Nucleus	13C
Acquired Size	32768
Spectral Size	65536

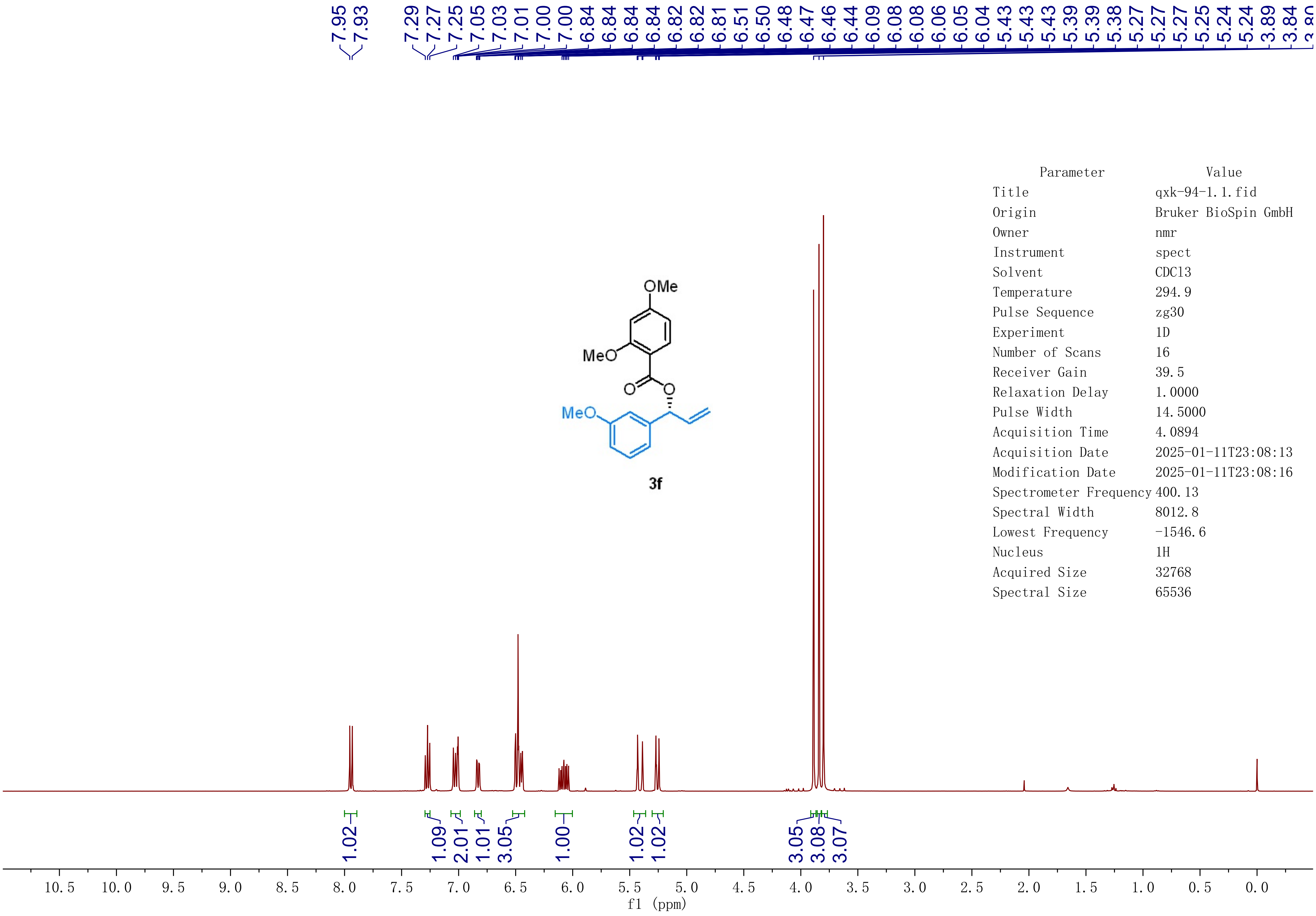

3f

Parameter	Value
Title	qxk-94-1.1.fid
Origin	Bruker BioSpin GmbH
Owner	nmr
Instrument	spect
Solvent	CDCl3
Temperature	294.9
Pulse Sequence	zg30
Experiment	1D
Number of Scans	16
Receiver Gain	39.5
Relaxation Delay	1.0000
Pulse Width	14.5000
Acquisition Time	4.0894
Acquisition Date	2025-01-11T23:08:13
Modification Date	2025-01-11T23:08:16
Spectrometer Frequency	400.13
Spectral Width	8012.8
Lowest Frequency	-1546.6
Nucleus	1H
Acquired Size	32768
Spectral Size	65536

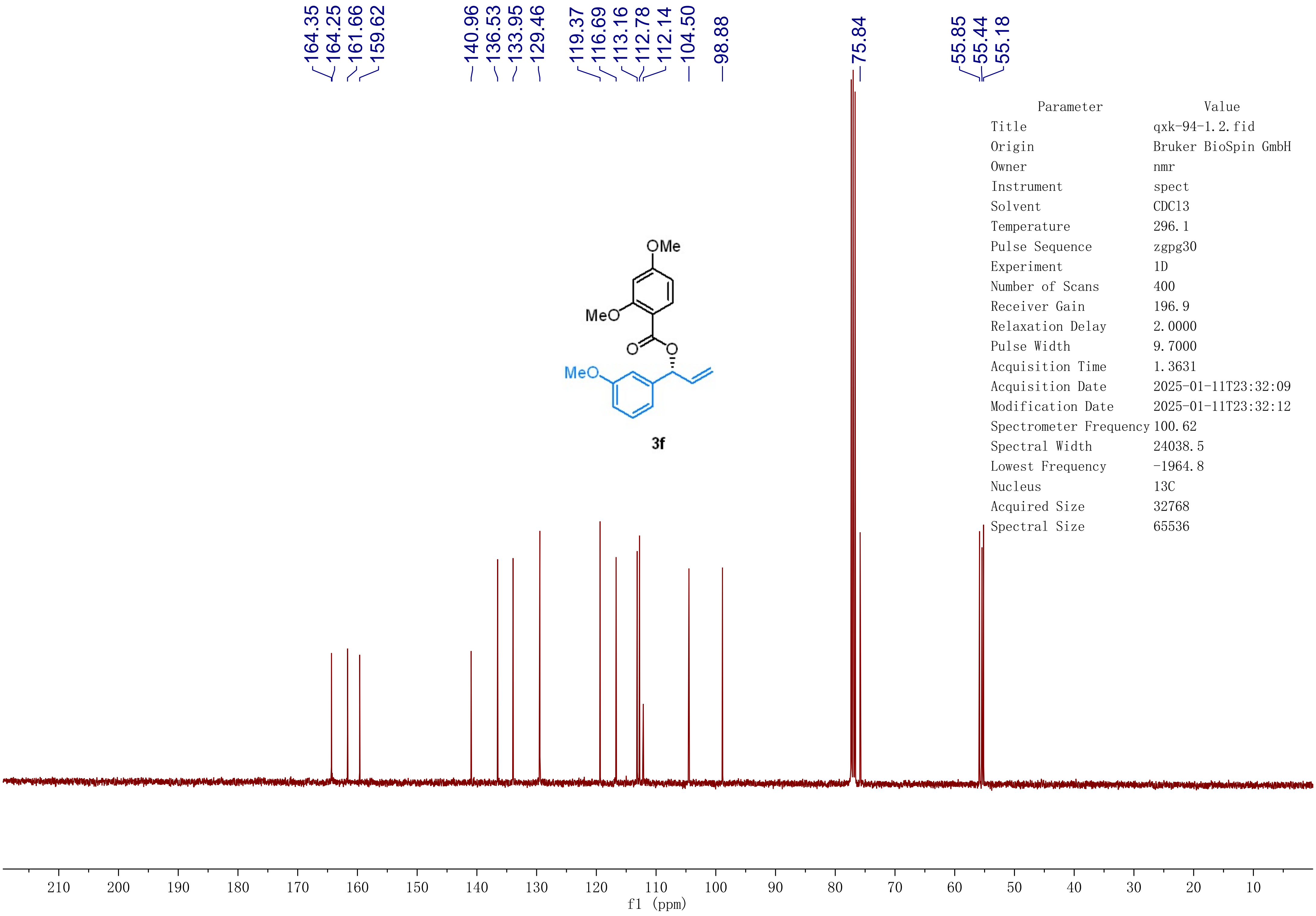

Parameter	Value
Title	qxk-94-1.2.fid
Origin	Bruker BioSpin GmbH
Owner	nmr
Instrument	spect
Solvent	CDCl3
Temperature	296.1
Pulse Sequence	zgpg30
Experiment	1D
Number of Scans	400
Receiver Gain	196.9
Relaxation Delay	2.0000
Pulse Width	9.7000
Acquisition Time	1.3631
Acquisition Date	2025-01-11T23:32:09
Modification Date	2025-01-11T23:32:12
Spectrometer Frequency	100.62
Spectral Width	24038.5
Lowest Frequency	-1964.8
Nucleus	13C
Acquired Size	32768
Spectral Size	65536

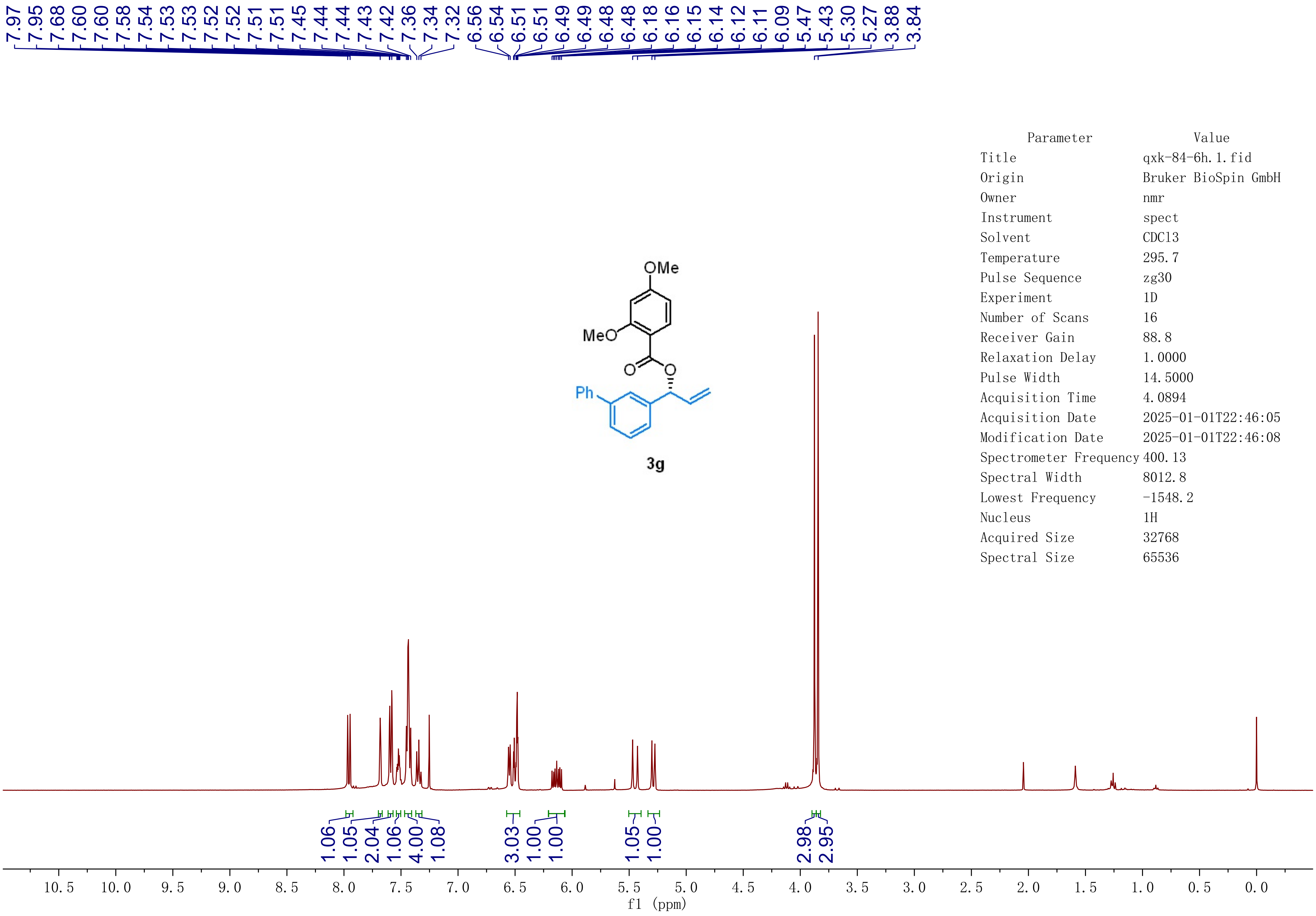

3g

Parameter	Value
Title	qxk-84-6h.1.fid
Origin	Bruker BioSpin GmbH
Owner	nmr
Instrument	spect
Solvent	CDCl3
Temperature	295.7
Pulse Sequence	zg30
Experiment	1D
Number of Scans	16
Receiver Gain	88.8
Relaxation Delay	1.0000
Pulse Width	14.5000
Acquisition Time	4.0894
Acquisition Date	2025-01-01T22:46:05
Modification Date	2025-01-01T22:46:08
Spectrometer Frequency	400.13
Spectral Width	8012.8
Lowest Frequency	-1548.2
Nucleus	1H
Acquired Size	32768
Spectral Size	65536

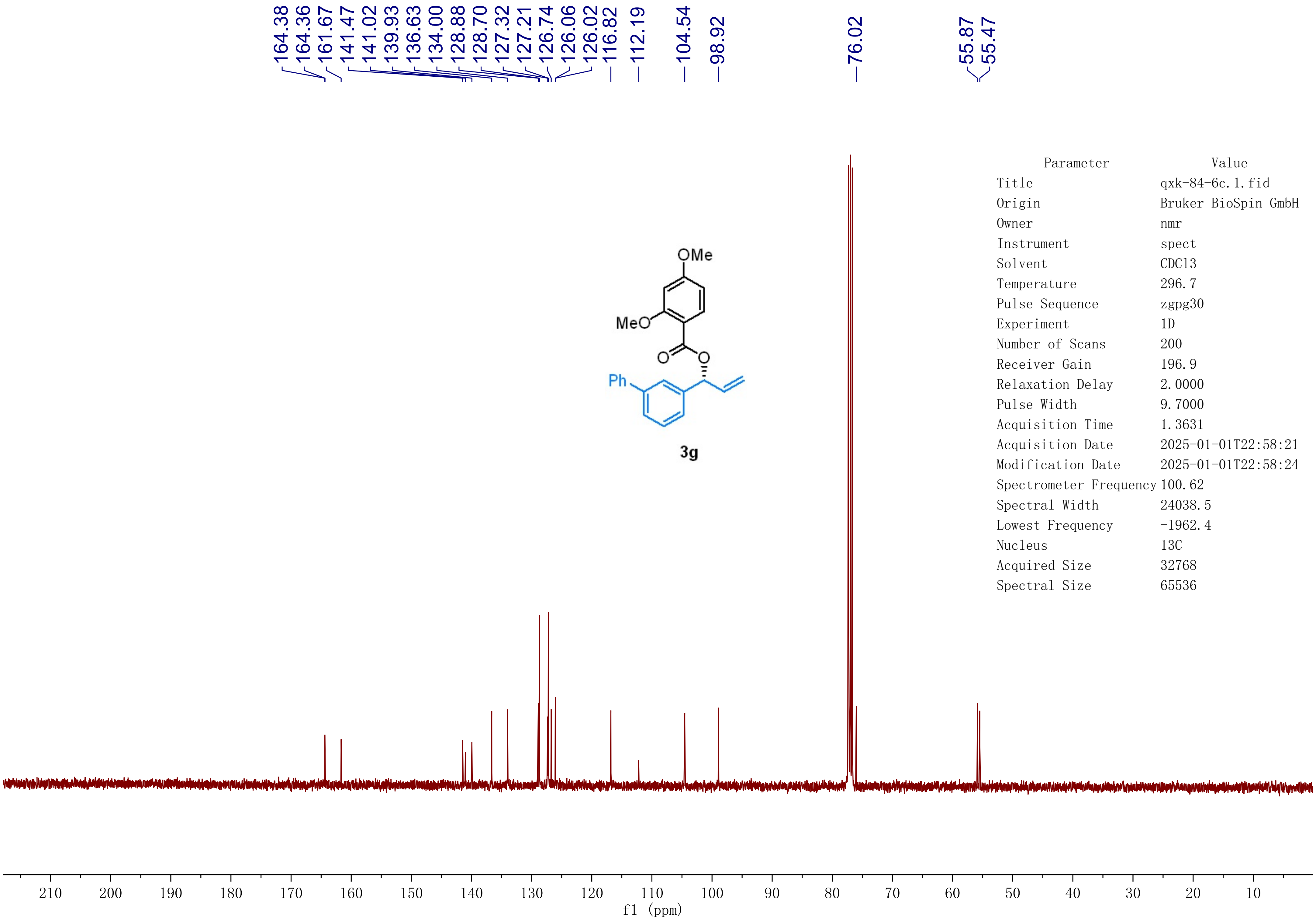

3g

Parameter	Value
Title	qxk-84-6c.1.fid
Origin	Bruker BioSpin GmbH
Owner	nmr
Instrument	spect
Solvent	CDCl3
Temperature	296.7
Pulse Sequence	zgpg30
Experiment	1D
Number of Scans	200
Receiver Gain	196.9
Relaxation Delay	2.0000
Pulse Width	9.7000
Acquisition Time	1.3631
Acquisition Date	2025-01-01T22:58:21
Modification Date	2025-01-01T22:58:24
Spectrometer Frequency	100.62
Spectral Width	24038.5
Lowest Frequency	-1962.4
Nucleus	13C
Acquired Size	32768
Spectral Size	65536

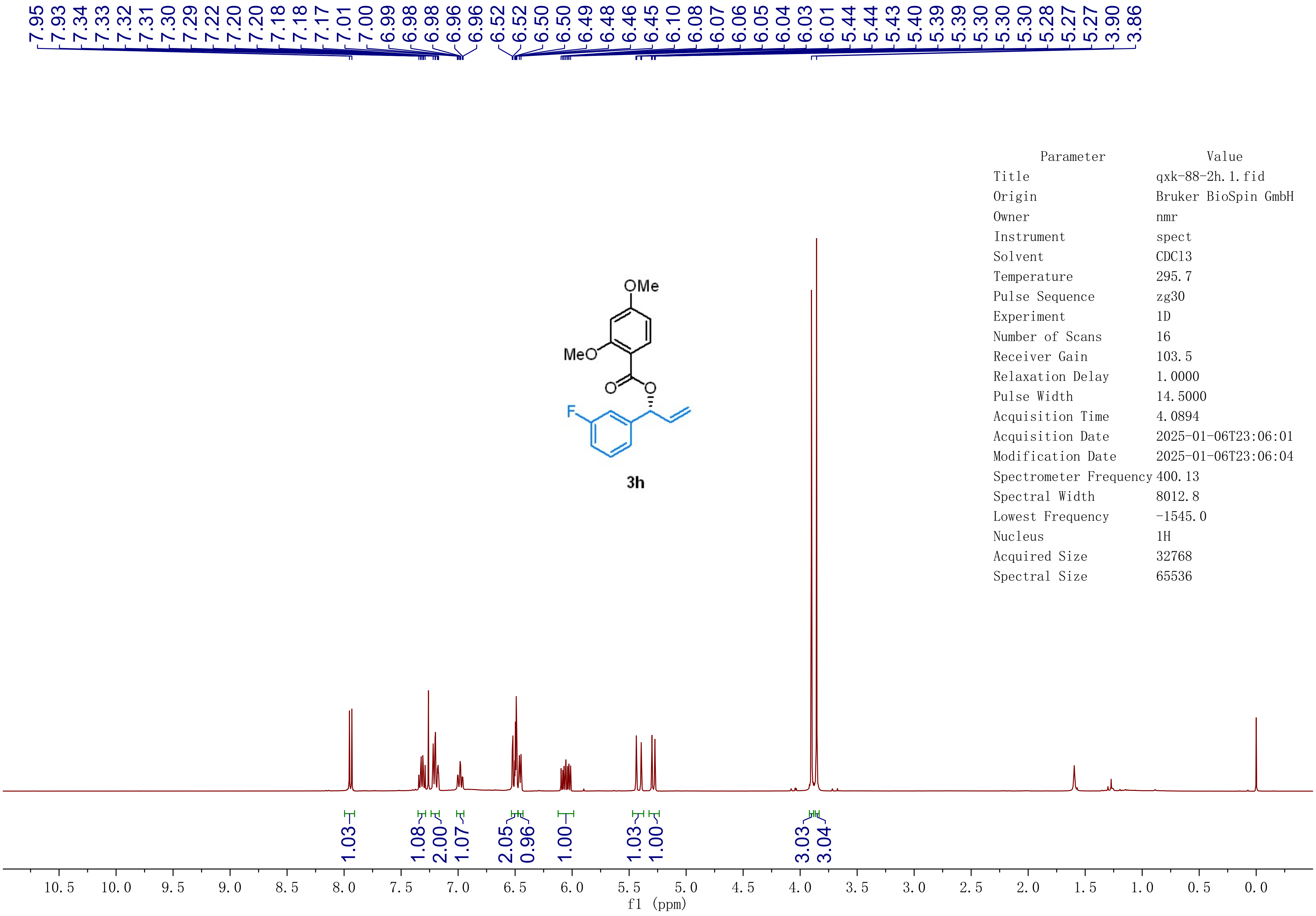

3h

Parameter	Value
Title	qxk-88-2h.1.fid
Origin	Bruker BioSpin GmbH
Owner	nmr
Instrument	spect
Solvent	CDCl3
Temperature	295.7
Pulse Sequence	zg30
Experiment	1D
Number of Scans	16
Receiver Gain	103.5
Relaxation Delay	1.0000
Pulse Width	14.5000
Acquisition Time	4.0894
Acquisition Date	2025-01-06T23:06:01
Modification Date	2025-01-06T23:06:04
Spectrometer Frequency	400.13
Spectral Width	8012.8
Lowest Frequency	-1545.0
Nucleus	1H
Acquired Size	32768
Spectral Size	65536

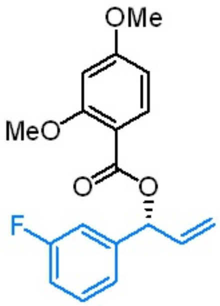

3h

Parameter	Value
Title	qxk-88-2f.1.fid
Origin	Bruker BioSpin GmbH
Owner	nmr
Instrument	AvanceNeo 400MHz
Solvent	CDCl3
Temperature	296.0
Pulse Sequence	zg
Experiment	1D
Number of Scans	16
Receiver Gain	101.0
Relaxation Delay	1.0000
Pulse Width	12.0000
Acquisition Time	0.7209
Acquisition Date	2025-01-08T09:31:07
Modification Date	2025-01-08T09:31:06
Spectrometer Frequency	376.51
Spectral Width	90909.1
Lowest Frequency	-83109.1
Nucleus	19F
Acquired Size	65536
Spectral Size	131072

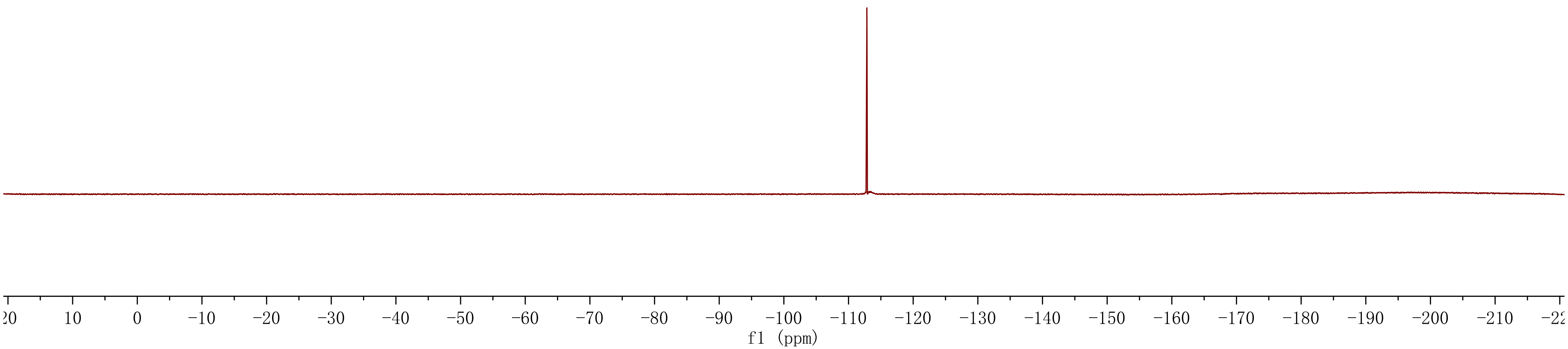

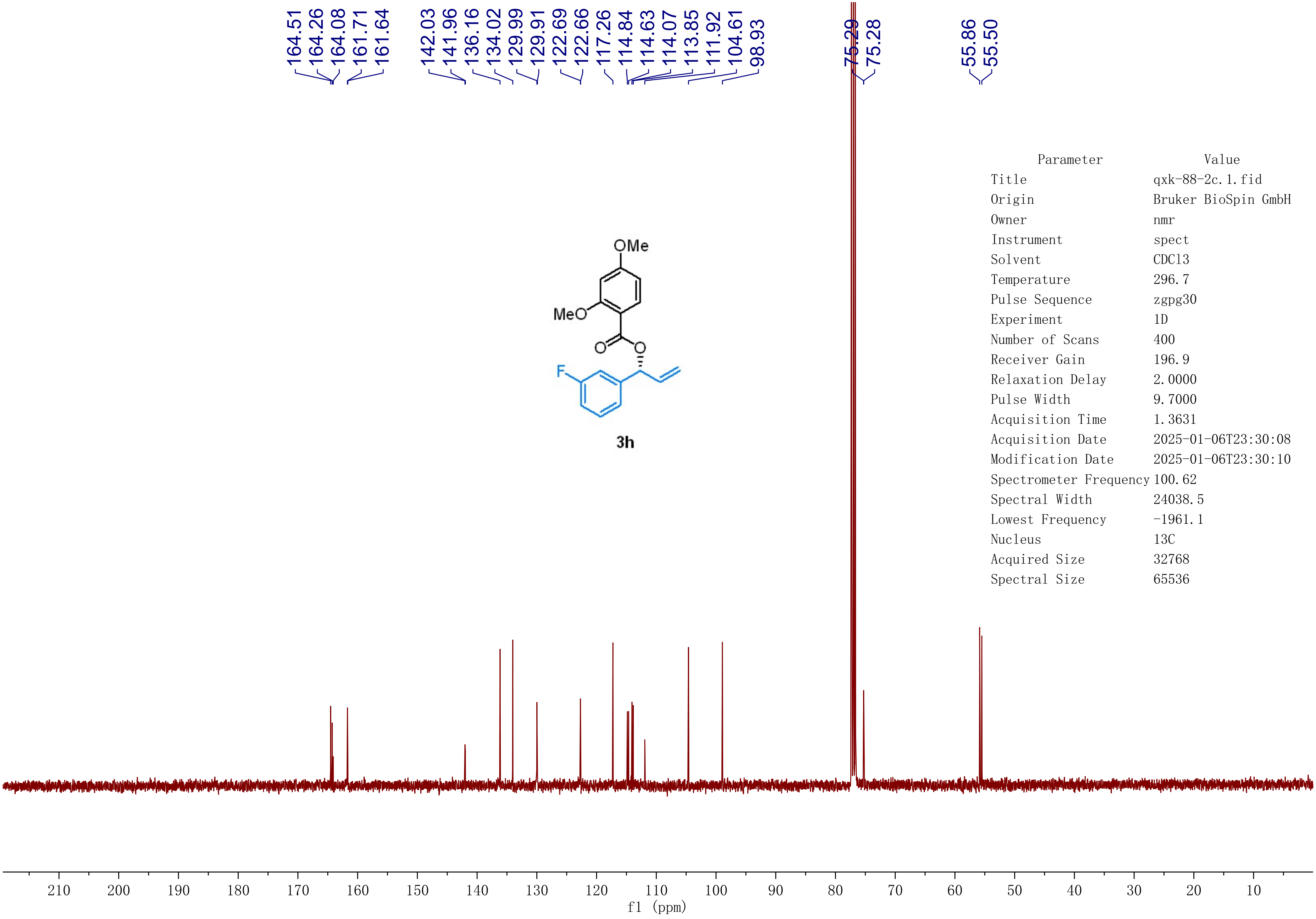

3h

Parameter	Value
Title	qxk-88-2c.1.fid
Origin	Bruker BioSpin GmbH
Owner	nmr
Instrument	spect
Solvent	CDCl3
Temperature	296.7
Pulse Sequence	zgpg30
Experiment	1D
Number of Scans	400
Receiver Gain	196.9
Relaxation Delay	2.0000
Pulse Width	9.7000
Acquisition Time	1.3631
Acquisition Date	2025-01-06T23:30:08
Modification Date	2025-01-06T23:30:10
Spectrometer Frequency	100.62
Spectral Width	24038.5
Lowest Frequency	-1961.1
Nucleus	13C
Acquired Size	32768
Spectral Size	65536

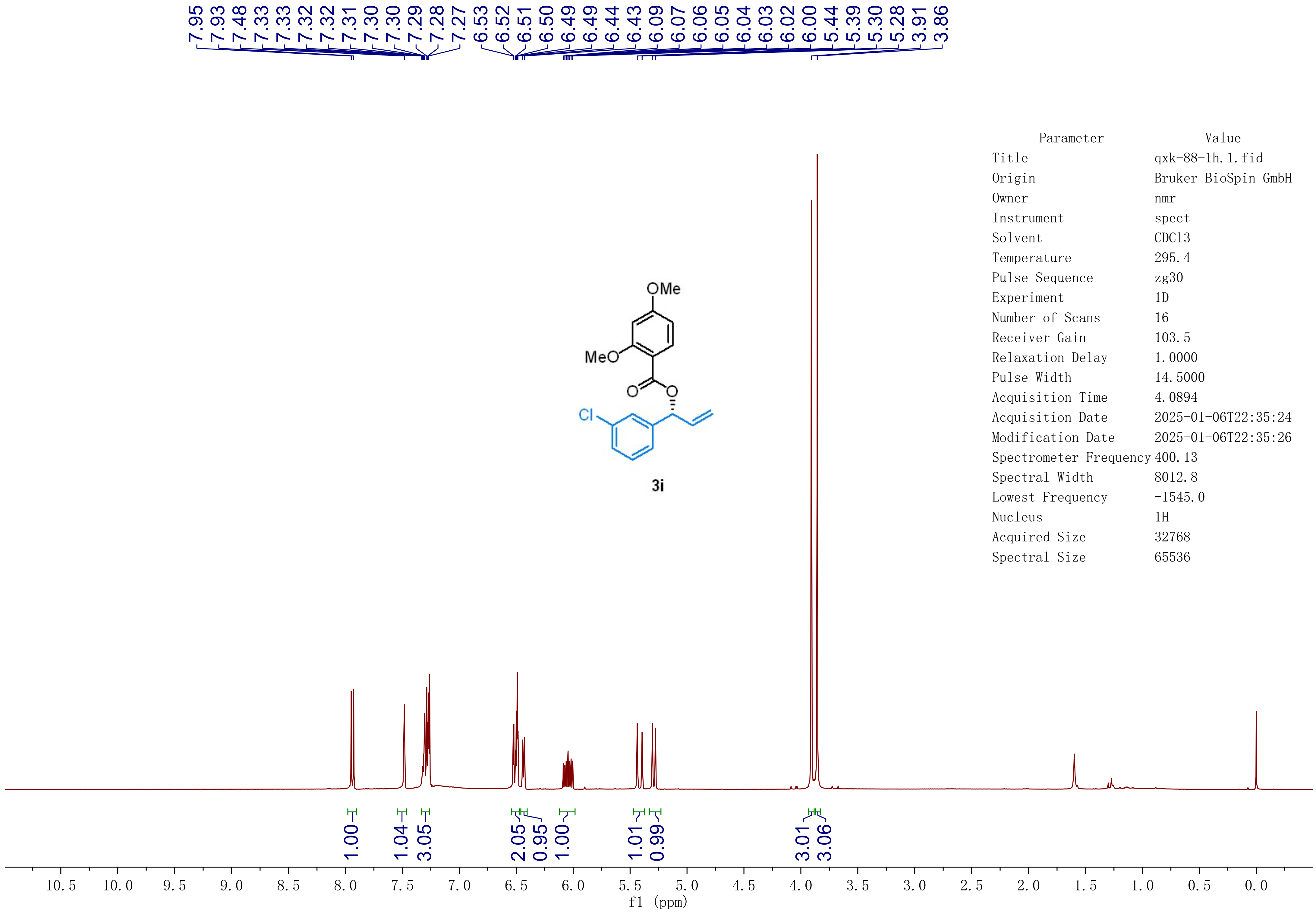

3i

Parameter	Value
Title	qxk-88-1h.1.fid
Origin	Bruker BioSpin GmbH
Owner	nmr
Instrument	spect
Solvent	CDCl3
Temperature	295.4
Pulse Sequence	zg30
Experiment	1D
Number of Scans	16
Receiver Gain	103.5
Relaxation Delay	1.0000
Pulse Width	14.5000
Acquisition Time	4.0894
Acquisition Date	2025-01-06T22:35:24
Modification Date	2025-01-06T22:35:26
Spectrometer Frequency	400.13
Spectral Width	8012.8
Lowest Frequency	-1545.0
Nucleus	1H
Acquired Size	32768
Spectral Size	65536

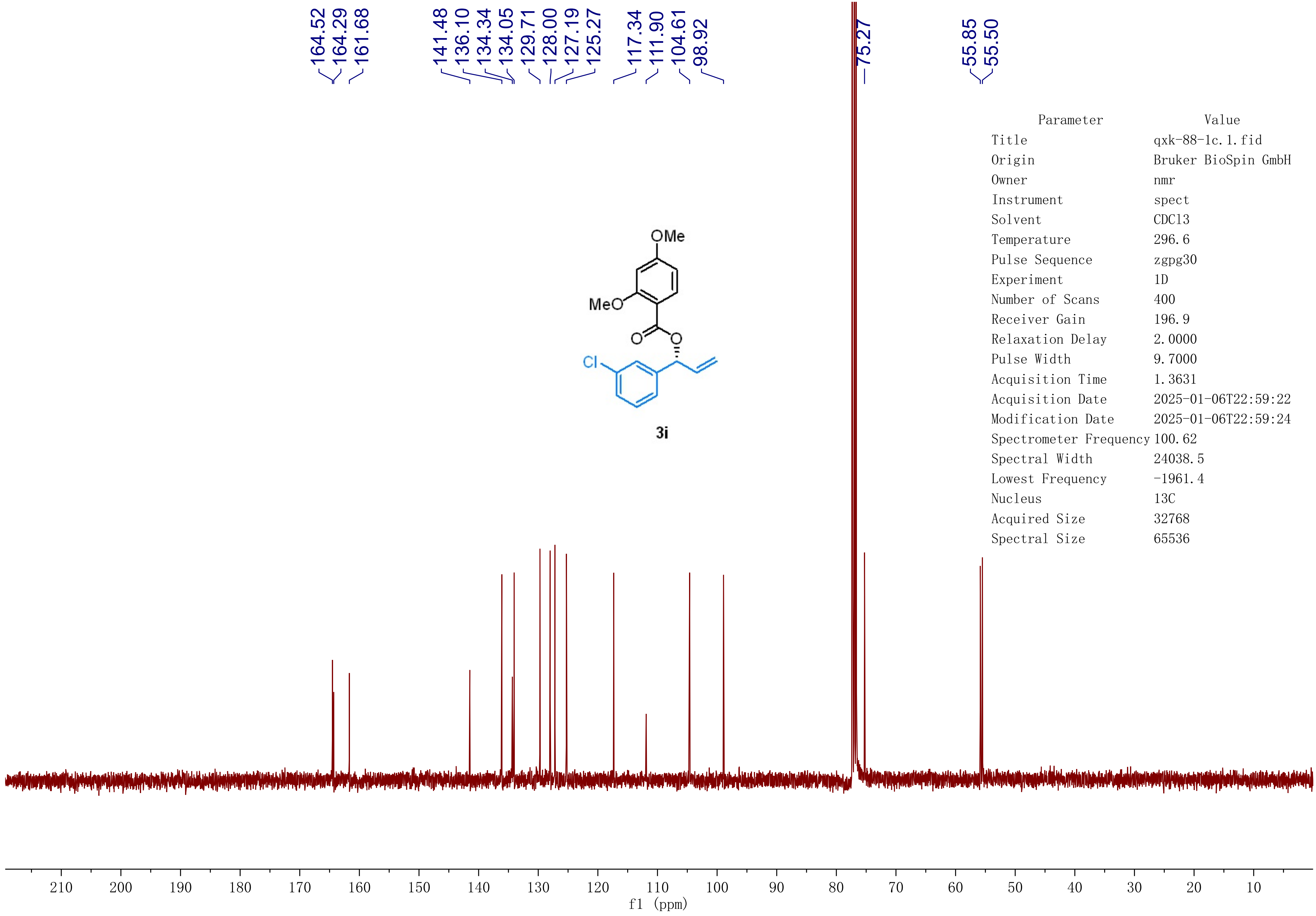

164.52
164.29
161.68
141.48
136.10
134.34
134.05
129.71
128.00
127.19
125.27
117.34
111.90
104.61
98.92
75.27
55.85
55.50
OMe
MeO
O
O
Cl
3i
Parameter Value
Title qxk-88-1c.1.fid
Origin Bruker BioSpin GmbH
Owner nmr
Instrument spect
Solvent CDCl3
Temperature 296.6
Pulse Sequence zgpg30
Experiment 1D
Number of Scans 400
Receiver Gain 196.9
Relaxation Delay 2.0000
Pulse Width 9.7000
Acquisition Time 1.3631
Acquisition Date 2025-01-06T22:59:22
Modification Date 2025-01-06T22:59:24
Spectrometer Frequency 100.62
Spectral Width 24038.5
Lowest Frequency -1961.4
Nucleus 13C
Acquired Size 32768
Spectral Size 65536
210 200 190 180 170 160 150 140 130 120 110 100 90 80 70 60 50 40 30 20 10
f1 (ppm)

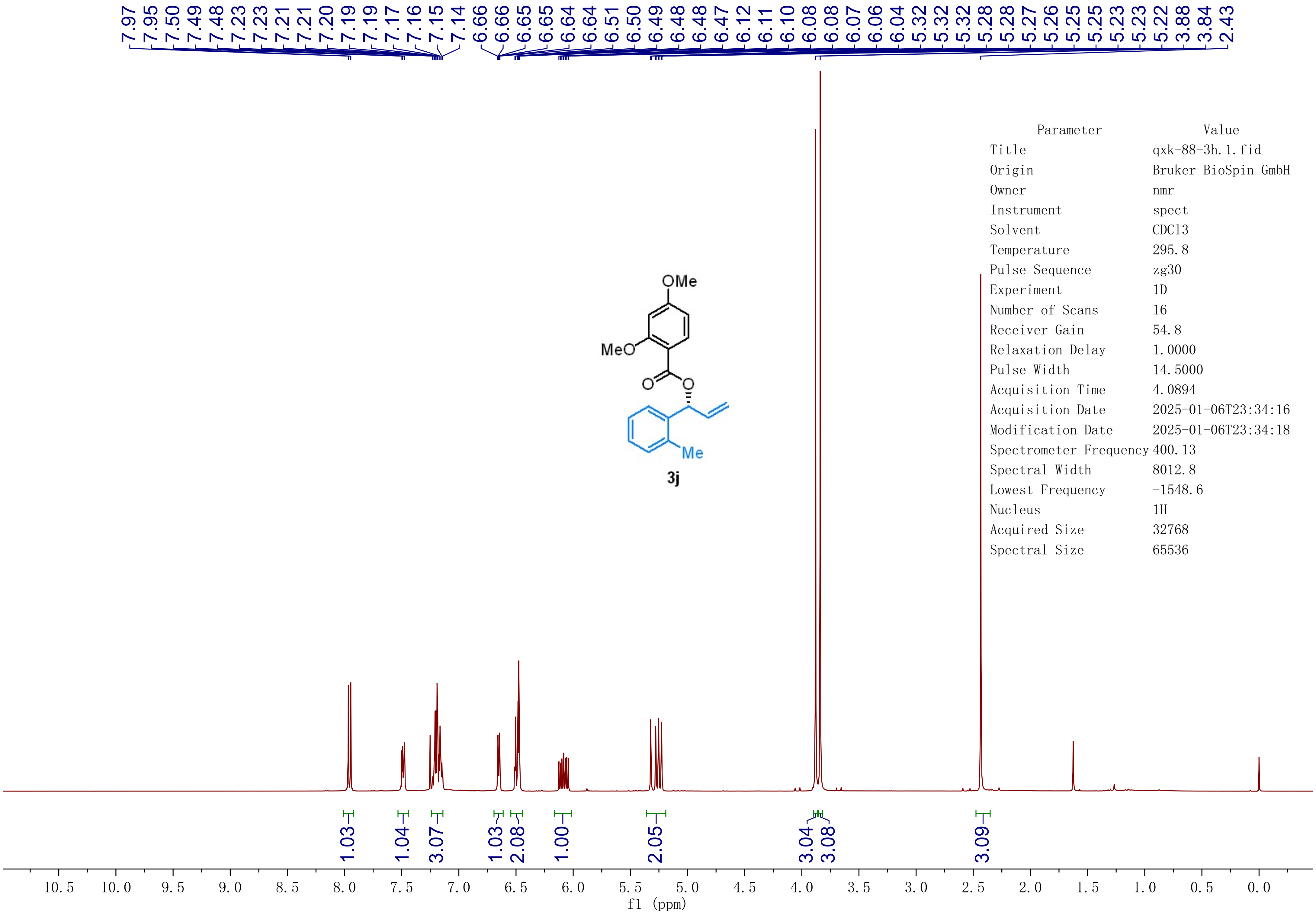
7.97 7.95 7.50 7.49 7.48 7.23 7.23 7.21 7.21 7.20 7.19 7.19 7.17 7.16 7.15 7.14 6.66 6.66 6.65 6.65 6.64 6.64 6.51 6.50 6.49 6.48 6.48 6.47 6.12 6.11 6.10 6.08 6.08 6.07 6.06 6.04 5.32 5.32 5.32 5.28 5.28 5.27 5.26 5.25 5.25 5.23 5.23 5.22 3.88 3.84 2.43
Parameter Value
Title qxk-88-3h.1.fid
Origin Bruker BioSpin GmbH
Owner nmr
Instrument spect
Solvent CDCl3
Temperature 295.8
Pulse Sequence zg30
Experiment 1D
Number of Scans 16
Receiver Gain 54.8
Relaxation Delay 1.0000
Pulse Width 14.5000
Acquisition Time 4.0894
Acquisition Date 2025-01-06T23:34:16
Modification Date 2025-01-06T23:34:18
Spectrometer Frequency 400.13
Spectral Width 8012.8
Lowest Frequency -1548.6
Nucleus 1H
Acquired Size 32768
Spectral Size 65536
OMe
MeO
O
O
Me
3j
1.03 1.04 3.07 1.03 2.08 1.00 2.05 3.04 3.08 3.09
10.5 10.0 9.5 9.0 8.5 8.0 7.5 7.0 6.5 6.0 5.5 5.0 4.5 4.0 3.5 3.0 2.5 2.0 1.5 1.0 0.5 0.0
f1 (ppm)

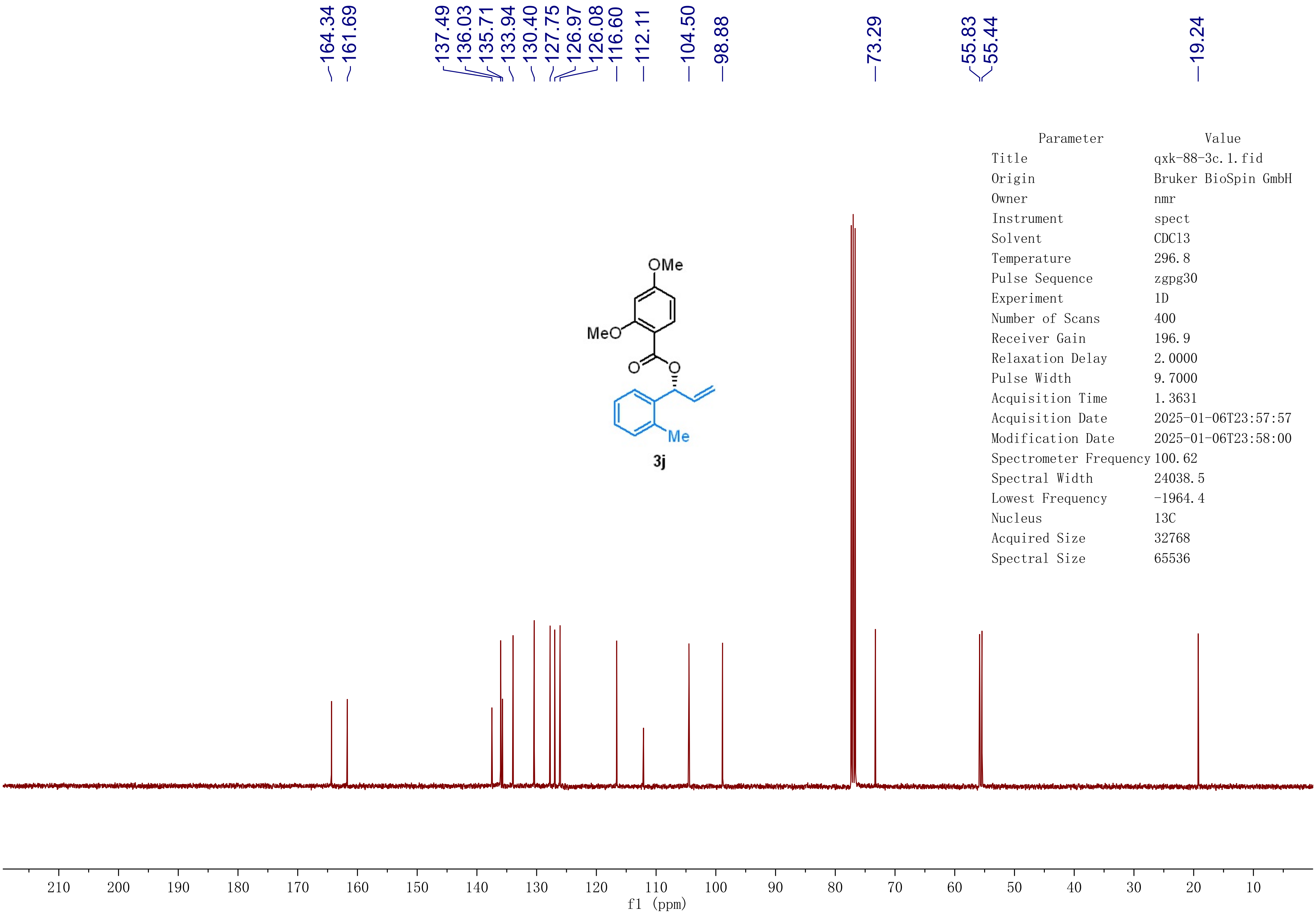

3j

Parameter	Value
Title	qxk-88-3c.1.fid
Origin	Bruker BioSpin GmbH
Owner	nmr
Instrument	spect
Solvent	CDCl3
Temperature	296.8
Pulse Sequence	zgpg30
Experiment	1D
Number of Scans	400
Receiver Gain	196.9
Relaxation Delay	2.0000
Pulse Width	9.7000
Acquisition Time	1.3631
Acquisition Date	2025-01-06T23:57:57
Modification Date	2025-01-06T23:58:00
Spectrometer Frequency	100.62
Spectral Width	24038.5
Lowest Frequency	-1964.4
Nucleus	13C
Acquired Size	32768
Spectral Size	65536

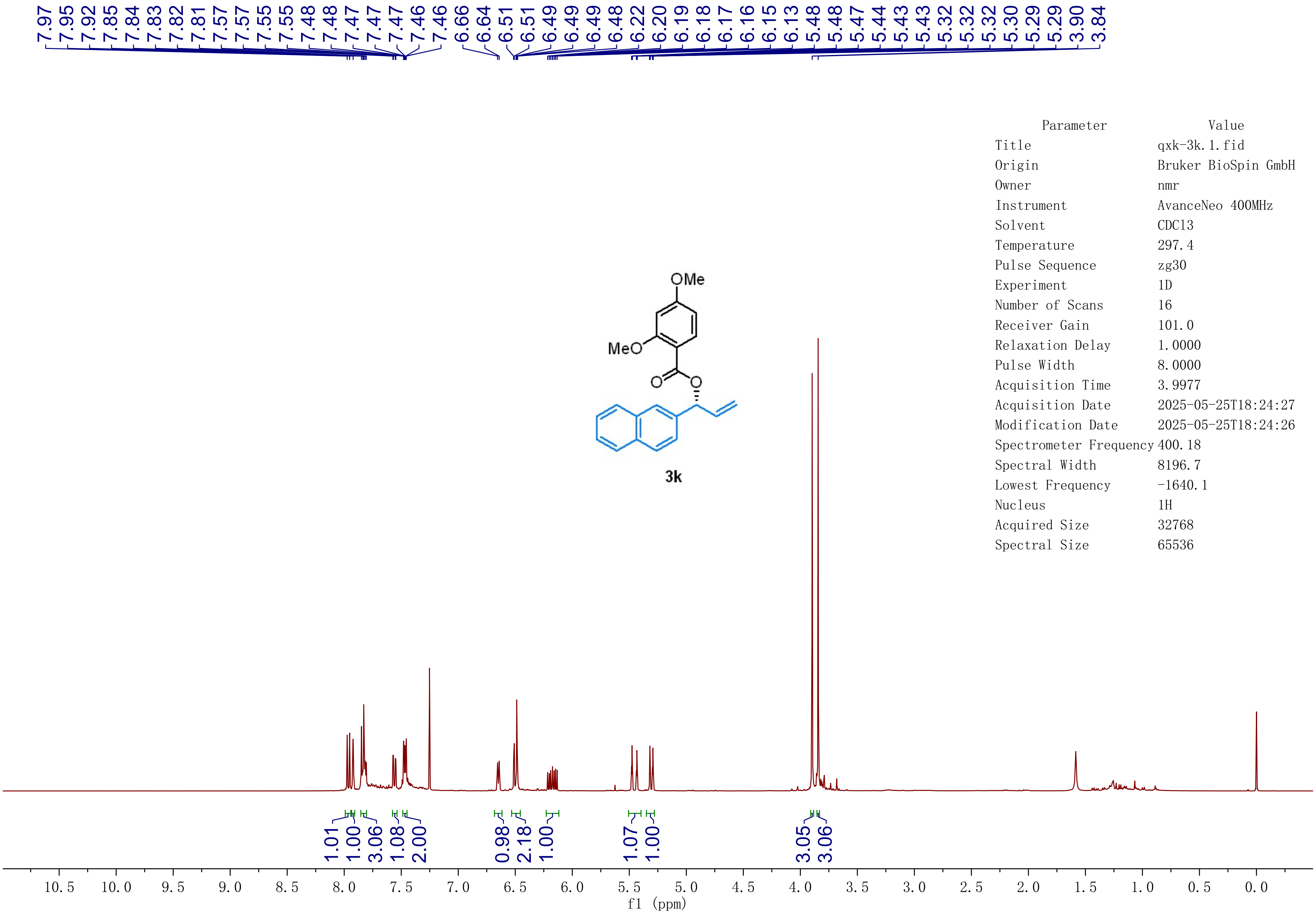

Parameter	Value
Title	qxk-3k.1.fid
Origin	Bruker BioSpin GmbH
Owner	nmr
Instrument	AvanceNeo 400MHz
Solvent	CDCl3
Temperature	297.4
Pulse Sequence	zg30
Experiment	1D
Number of Scans	16
Receiver Gain	101.0
Relaxation Delay	1.0000
Pulse Width	8.0000
Acquisition Time	3.9977
Acquisition Date	2025-05-25T18:24:27
Modification Date	2025-05-25T18:24:26
Spectrometer Frequency	400.18
Spectral Width	8196.7
Lowest Frequency	-1640.1
Nucleus	1H
Acquired Size	32768
Spectral Size	65536

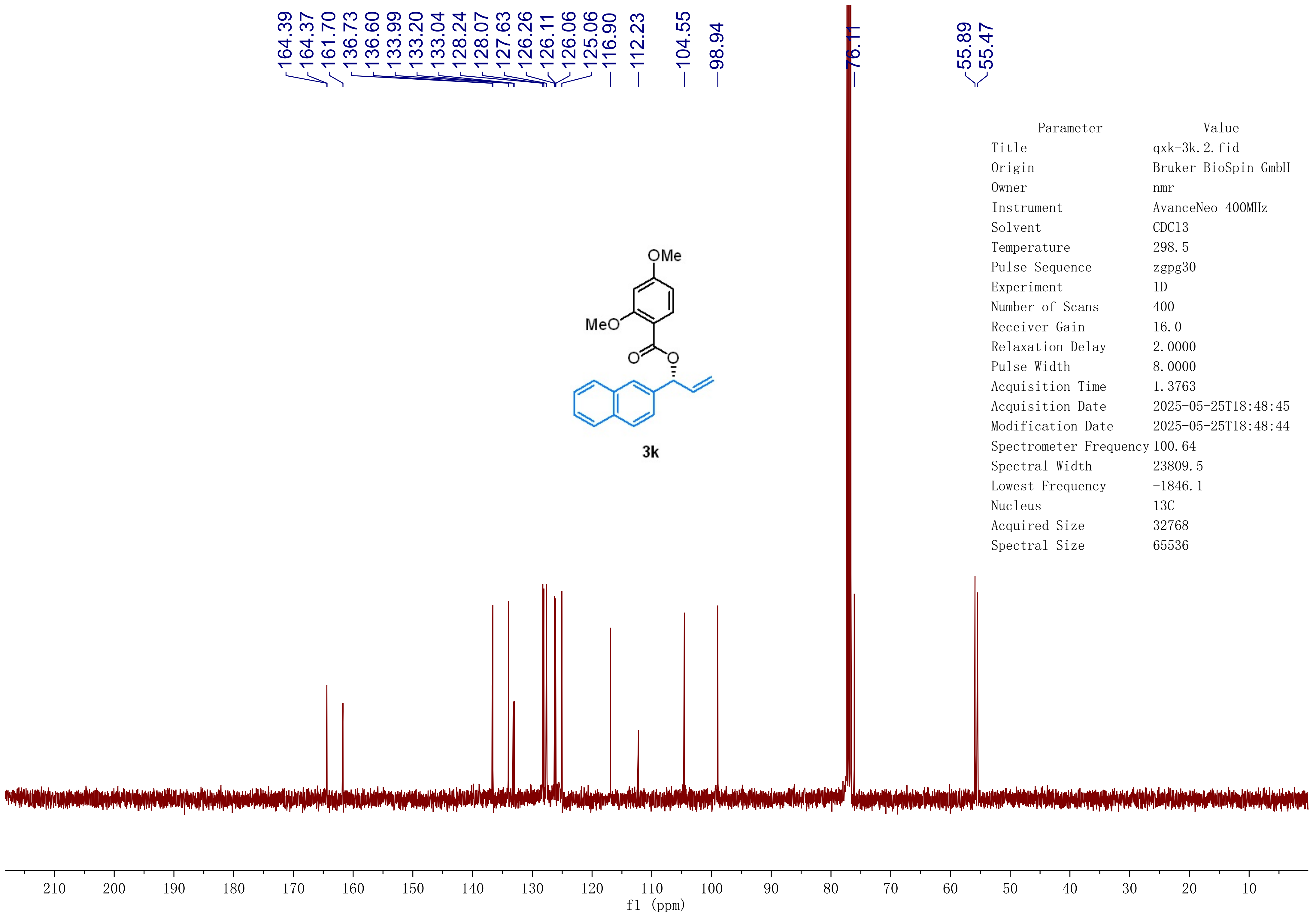
164.39
164.37
161.70
136.73
136.60
133.99
133.20
133.04
128.24
128.07
127.63
126.26
126.11
126.06
125.06
116.90
112.23
104.55
98.94
76.11
55.89
55.47
OMe
MeO
O
O
3k
Parameter Value
Title qxk-3k.2.fid
Origin Bruker BioSpin GmbH
Owner nmr
Instrument AvanceNeo 400MHz
Solvent CDCl3
Temperature 298.5
Pulse Sequence zgpg30
Experiment 1D
Number of Scans 400
Receiver Gain 16.0
Relaxation Delay 2.0000
Pulse Width 8.0000
Acquisition Time 1.3763
Acquisition Date 2025-05-25T18:48:45
Modification Date 2025-05-25T18:48:44
Spectrometer Frequency 100.64
Spectral Width 23809.5
Lowest Frequency -1846.1
Nucleus 13C
Acquired Size 32768
Spectral Size 65536
210 200 190 180 170 160 150 140 130 120 110 100 90 80 70 60 50 40 30 20 10
f1 (ppm)

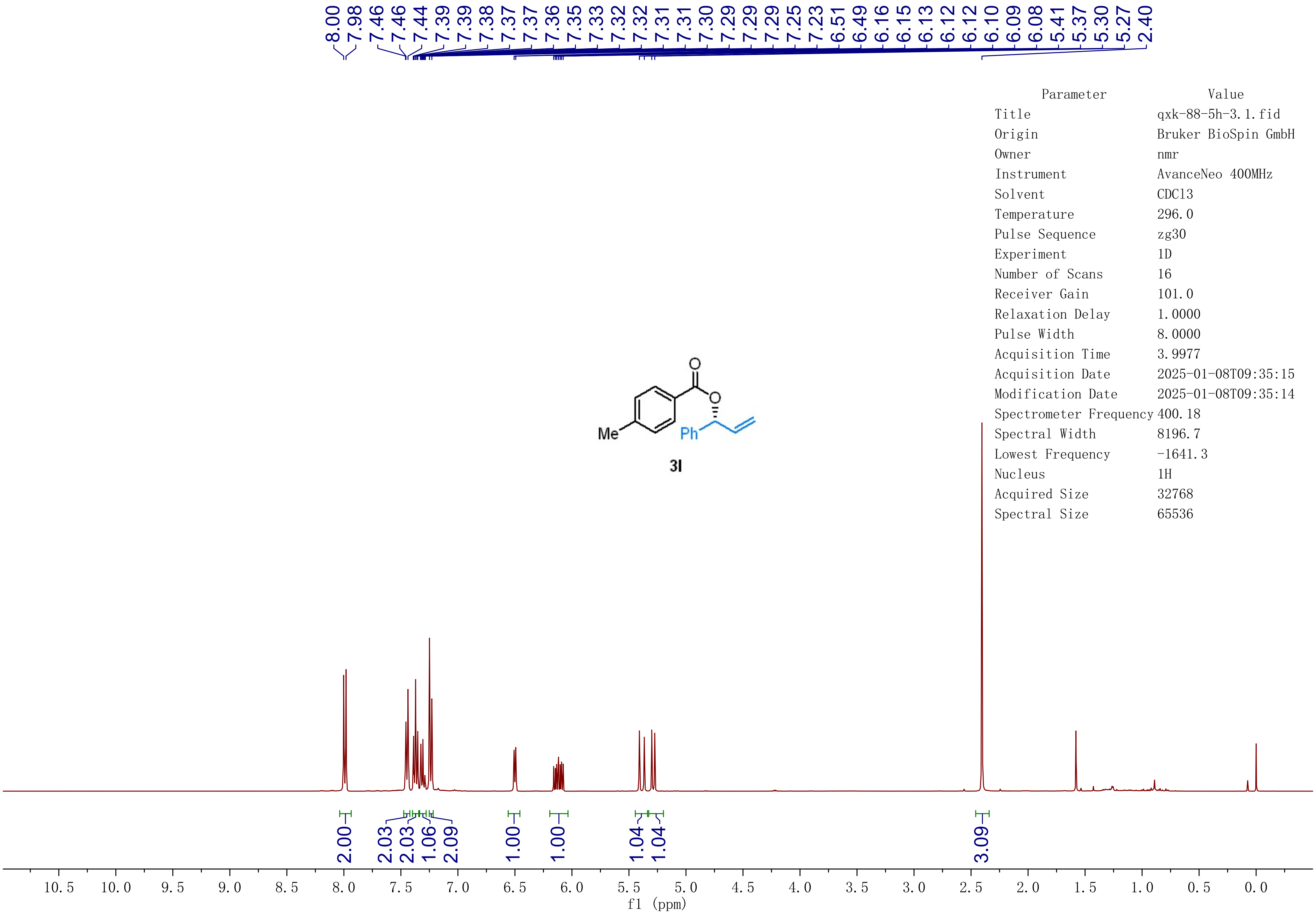

Parameter	Value
Title	qxk-88-5h-3.1.fid
Origin	Bruker BioSpin GmbH
Owner	nmr
Instrument	AvanceNeo 400MHz
Solvent	CDCl3
Temperature	296.0
Pulse Sequence	zg30
Experiment	1D
Number of Scans	16
Receiver Gain	101.0
Relaxation Delay	1.0000
Pulse Width	8.0000
Acquisition Time	3.9977
Acquisition Date	2025-01-08T09:35:15
Modification Date	2025-01-08T09:35:14
Spectrometer Frequency	400.18
Spectral Width	8196.7
Lowest Frequency	-1641.3
Nucleus	1H
Acquired Size	32768
Spectral Size	65536

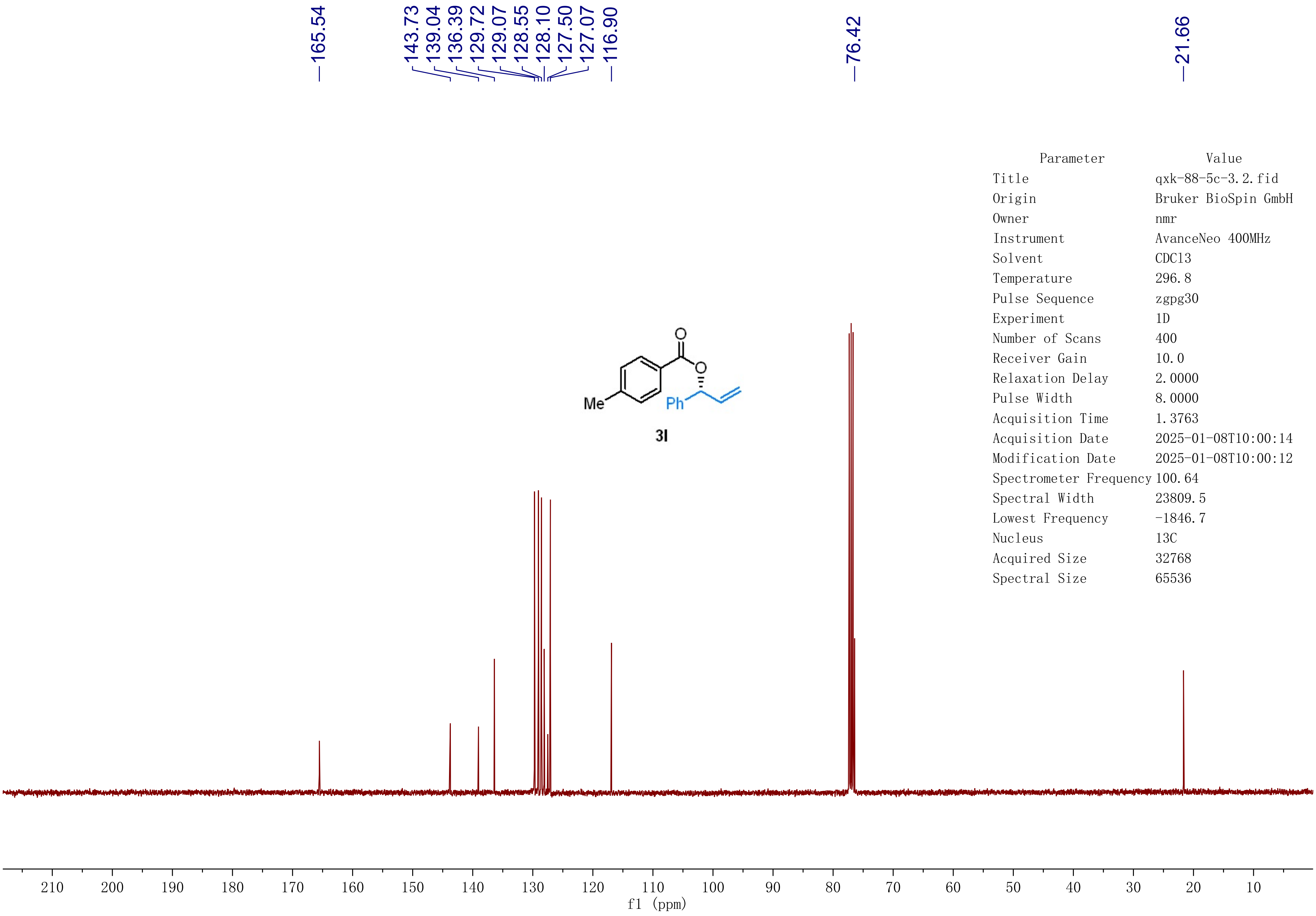

3l

Parameter	Value
Title	qxk-88-5c-3.2.fid
Origin	Bruker BioSpin GmbH
Owner	nmr
Instrument	AvanceNeo 400MHz
Solvent	CDCl3
Temperature	296.8
Pulse Sequence	zgpg30
Experiment	1D
Number of Scans	400
Receiver Gain	10.0
Relaxation Delay	2.0000
Pulse Width	8.0000
Acquisition Time	1.3763
Acquisition Date	2025-01-08T10:00:14
Modification Date	2025-01-08T10:00:12
Spectrometer Frequency	100.64
Spectral Width	23809.5
Lowest Frequency	-1846.7
Nucleus	13C
Acquired Size	32768
Spectral Size	65536

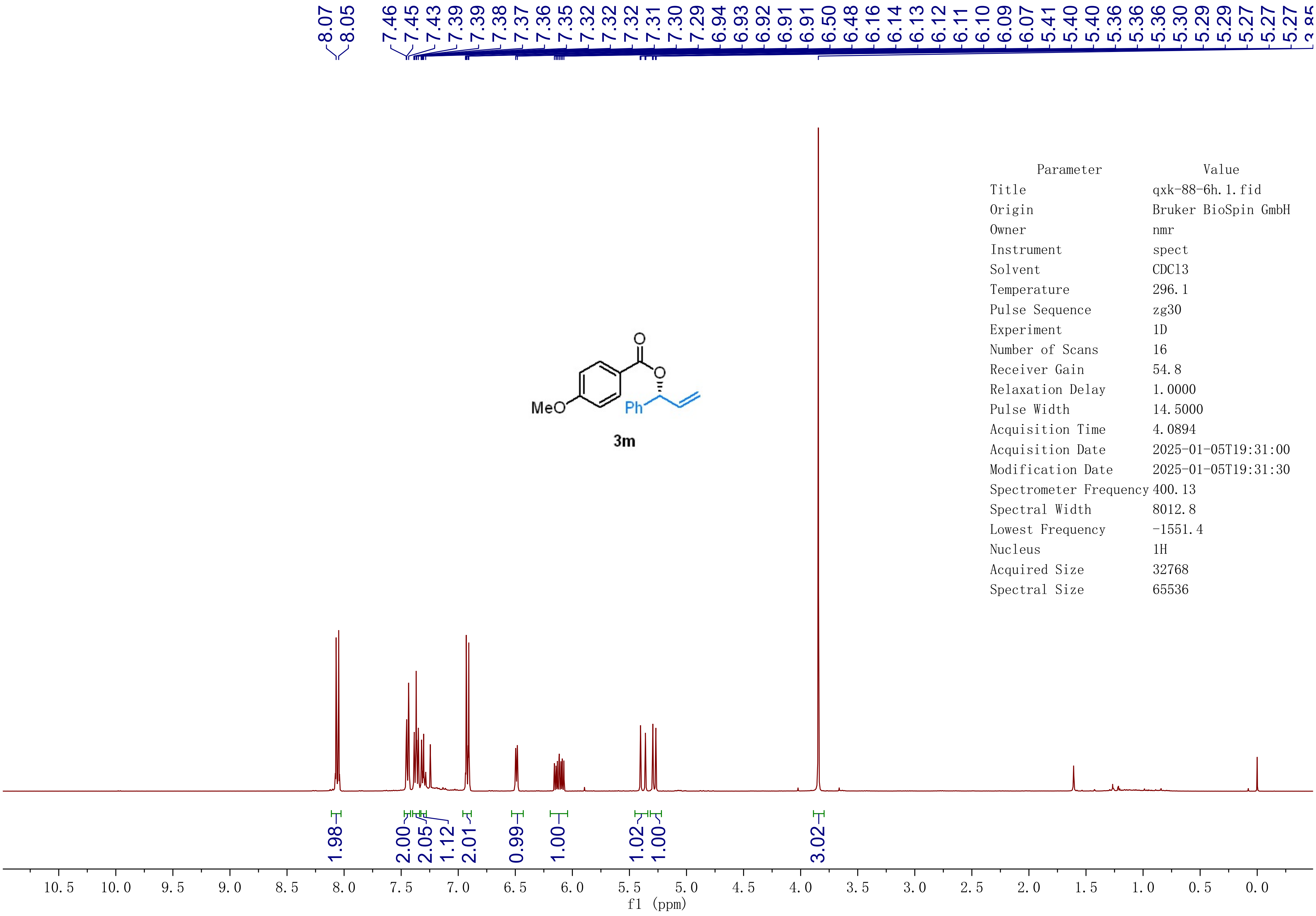

Parameter	Value
Title	qxk-88-6h.1.fid
Origin	Bruker BioSpin GmbH
Owner	nmr
Instrument	spect
Solvent	CDCl3
Temperature	296.1
Pulse Sequence	zg30
Experiment	1D
Number of Scans	16
Receiver Gain	54.8
Relaxation Delay	1.0000
Pulse Width	14.5000
Acquisition Time	4.0894
Acquisition Date	2025-01-05T19:31:00
Modification Date	2025-01-05T19:31:30
Spectrometer Frequency	400.13
Spectral Width	8012.8
Lowest Frequency	-1551.4
Nucleus	1H
Acquired Size	32768
Spectral Size	65536

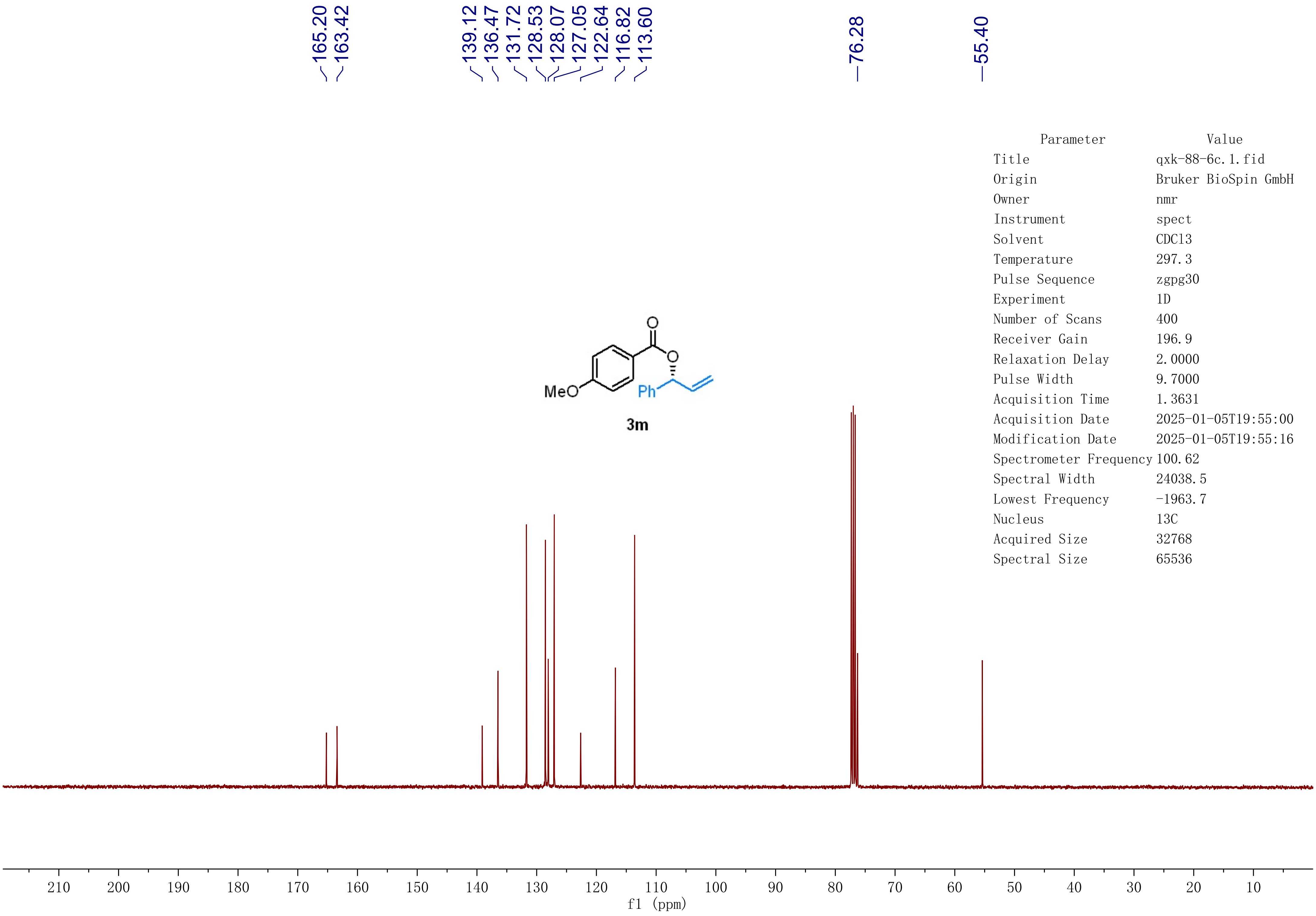

3m

Parameter	Value
Title	qxk-88-6c.1.fid
Origin	Bruker BioSpin GmbH
Owner	nmr
Instrument	spect
Solvent	CDCl3
Temperature	297.3
Pulse Sequence	zgpg30
Experiment	1D
Number of Scans	400
Receiver Gain	196.9
Relaxation Delay	2.0000
Pulse Width	9.7000
Acquisition Time	1.3631
Acquisition Date	2025-01-05T19:55:00
Modification Date	2025-01-05T19:55:16
Spectrometer Frequency	100.62
Spectral Width	24038.5
Lowest Frequency	-1963.7
Nucleus	13C
Acquired Size	32768
Spectral Size	65536

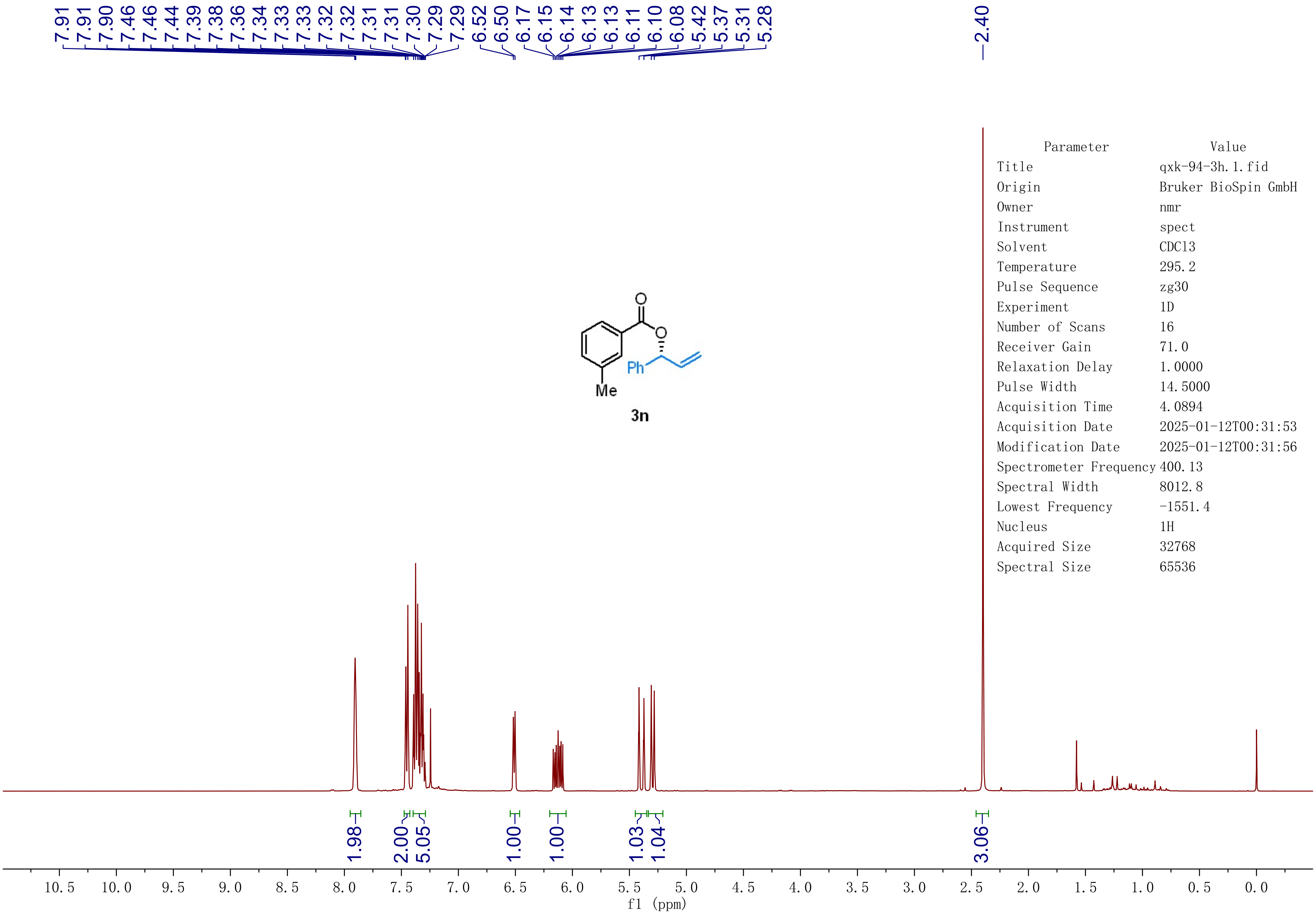

3n

Parameter	Value
Title	qxk-94-3h.1.fid
Origin	Bruker BioSpin GmbH
Owner	nmr
Instrument	spect
Solvent	CDCl3
Temperature	295.2
Pulse Sequence	zg30
Experiment	1D
Number of Scans	16
Receiver Gain	71.0
Relaxation Delay	1.0000
Pulse Width	14.5000
Acquisition Time	4.0894
Acquisition Date	2025-01-12T00:31:53
Modification Date	2025-01-12T00:31:56
Spectrometer Frequency	400.13
Spectral Width	8012.8
Lowest Frequency	-1551.4
Nucleus	1H
Acquired Size	32768
Spectral Size	65536

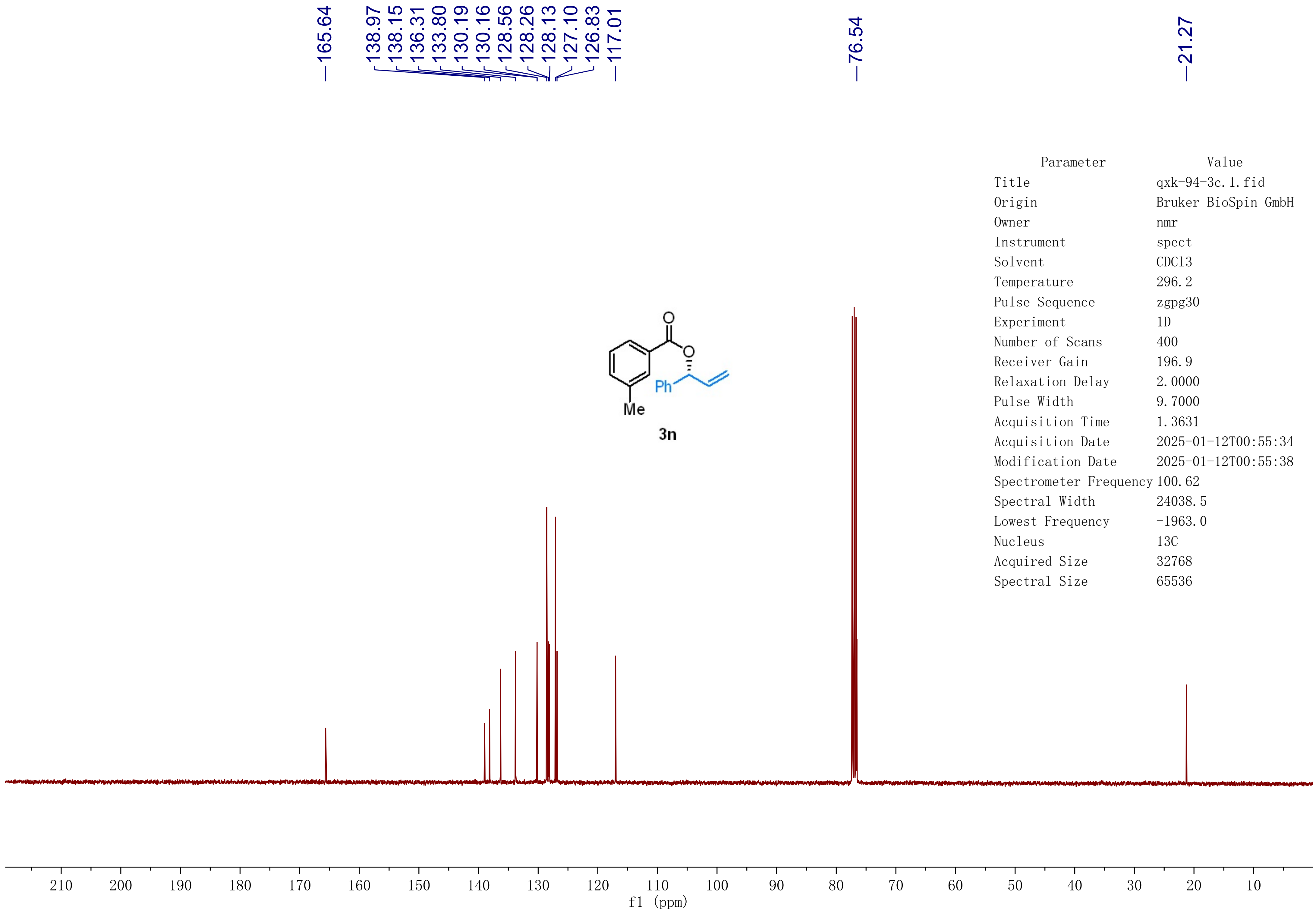

3n

Parameter	Value
Title	qxk-94-3c.1.fid
Origin	Bruker BioSpin GmbH
Owner	nmr
Instrument	spect
Solvent	CDCl3
Temperature	296.2
Pulse Sequence	zgpg30
Experiment	1D
Number of Scans	400
Receiver Gain	196.9
Relaxation Delay	2.0000
Pulse Width	9.7000
Acquisition Time	1.3631
Acquisition Date	2025-01-12T00:55:34
Modification Date	2025-01-12T00:55:38
Spectrometer Frequency	100.62
Spectral Width	24038.5
Lowest Frequency	-1963.0
Nucleus	13C
Acquired Size	32768
Spectral Size	65536

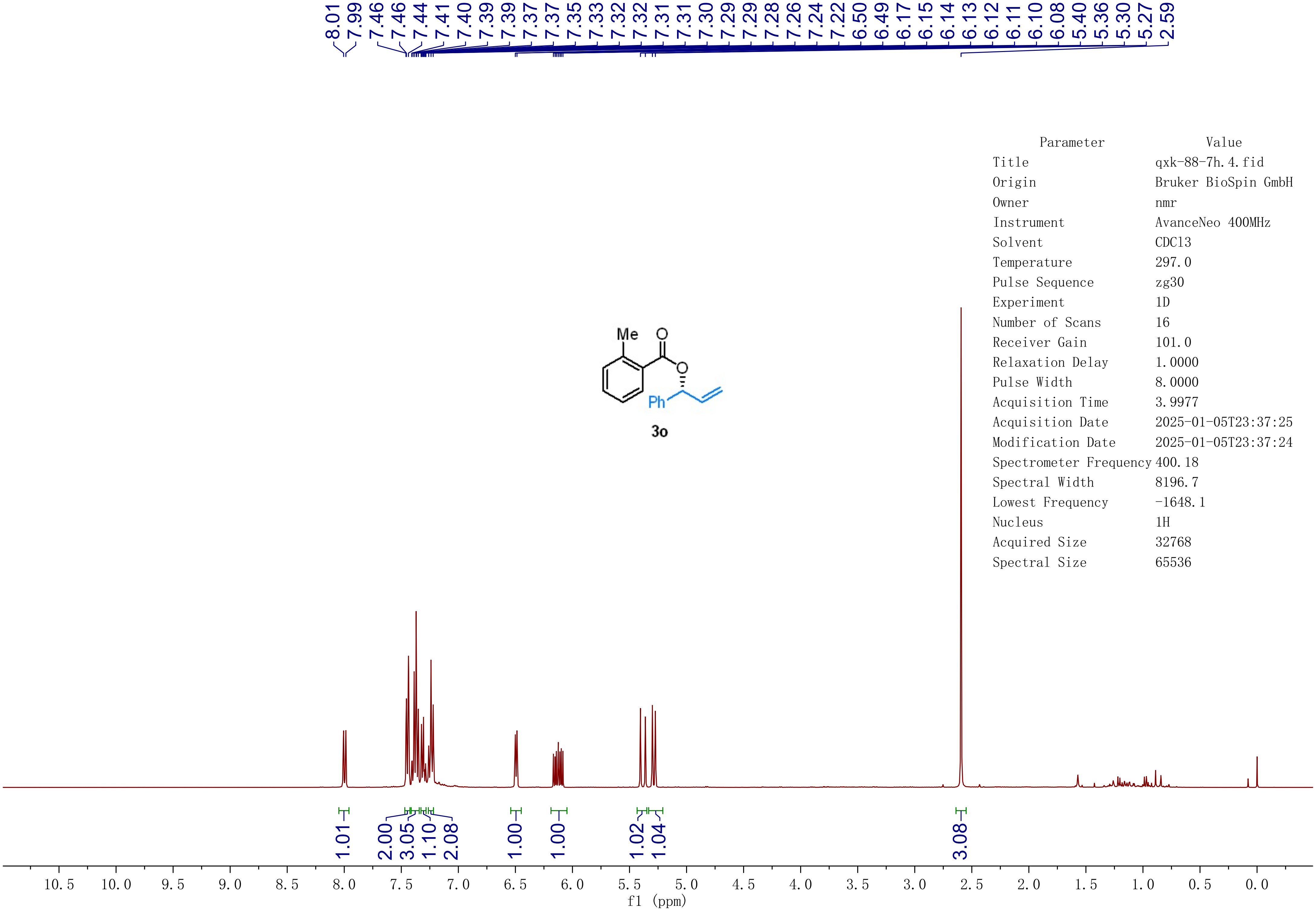

3o

Parameter	Value
Title	qxk-88-7h.4.fid
Origin	Bruker BioSpin GmbH
Owner	nmr
Instrument	AvanceNeo 400MHz
Solvent	CDCl3
Temperature	297.0
Pulse Sequence	zg30
Experiment	1D
Number of Scans	16
Receiver Gain	101.0
Relaxation Delay	1.0000
Pulse Width	8.0000
Acquisition Time	3.9977
Acquisition Date	2025-01-05T23:37:25
Modification Date	2025-01-05T23:37:24
Spectrometer Frequency	400.18
Spectral Width	8196.7
Lowest Frequency	-1648.1
Nucleus	1H
Acquired Size	32768
Spectral Size	65536

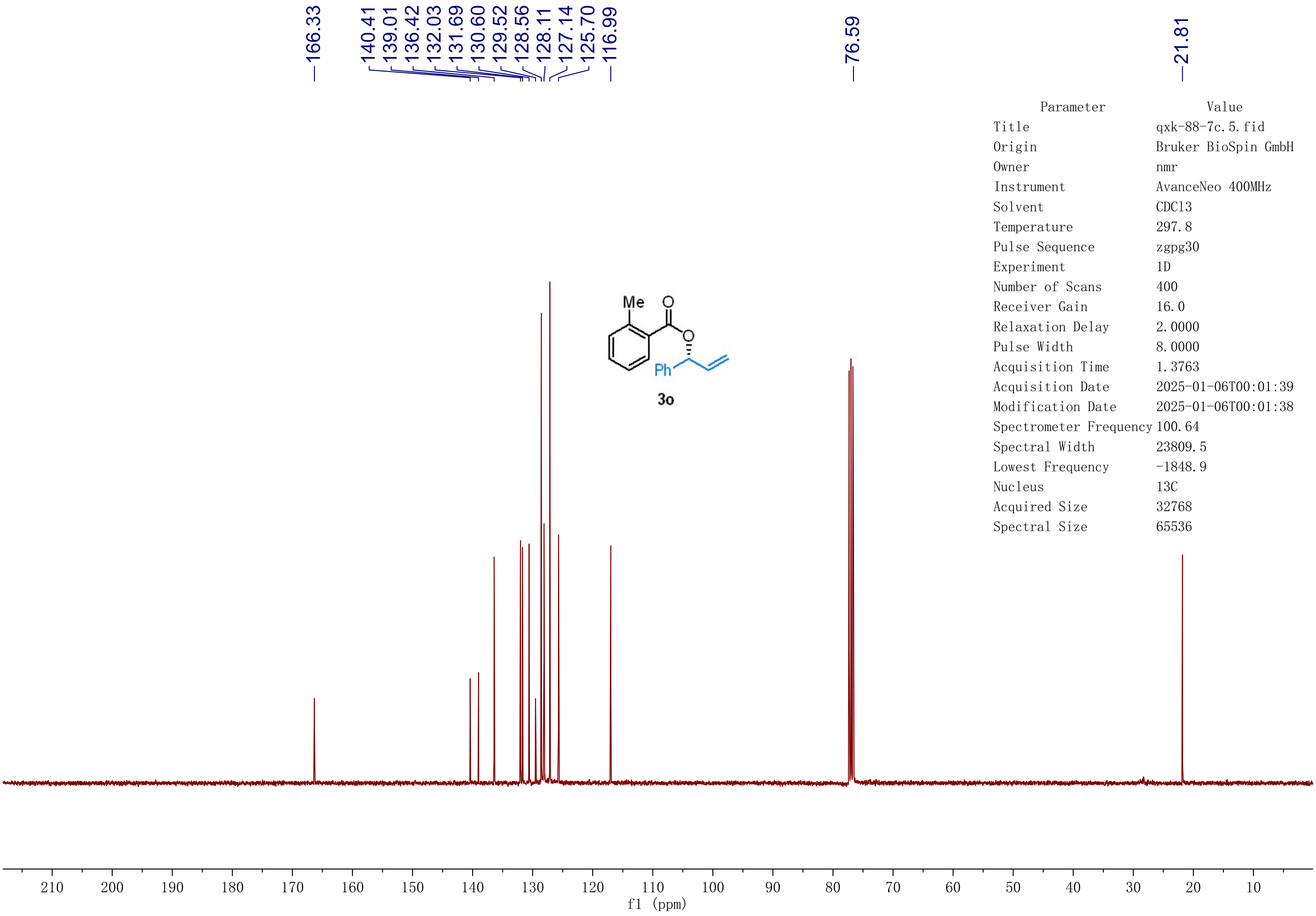

Parameter	Value
Title	qxk-88-7c.5.fid
Origin	Bruker BioSpin GmbH
Owner	nmr
Instrument	AvanceNeo 400MHz
Solvent	CDCl3
Temperature	297.8
Pulse Sequence	zgpg30
Experiment	1D
Number of Scans	400
Receiver Gain	16.0
Relaxation Delay	2.0000
Pulse Width	8.0000
Acquisition Time	1.3763
Acquisition Date	2025-01-06T00:01:39
Modification Date	2025-01-06T00:01:38
Spectrometer Frequency	100.64
Spectral Width	23809.5
Lowest Frequency	-1848.9
Nucleus	13C
Acquired Size	32768
Spectral Size	65536

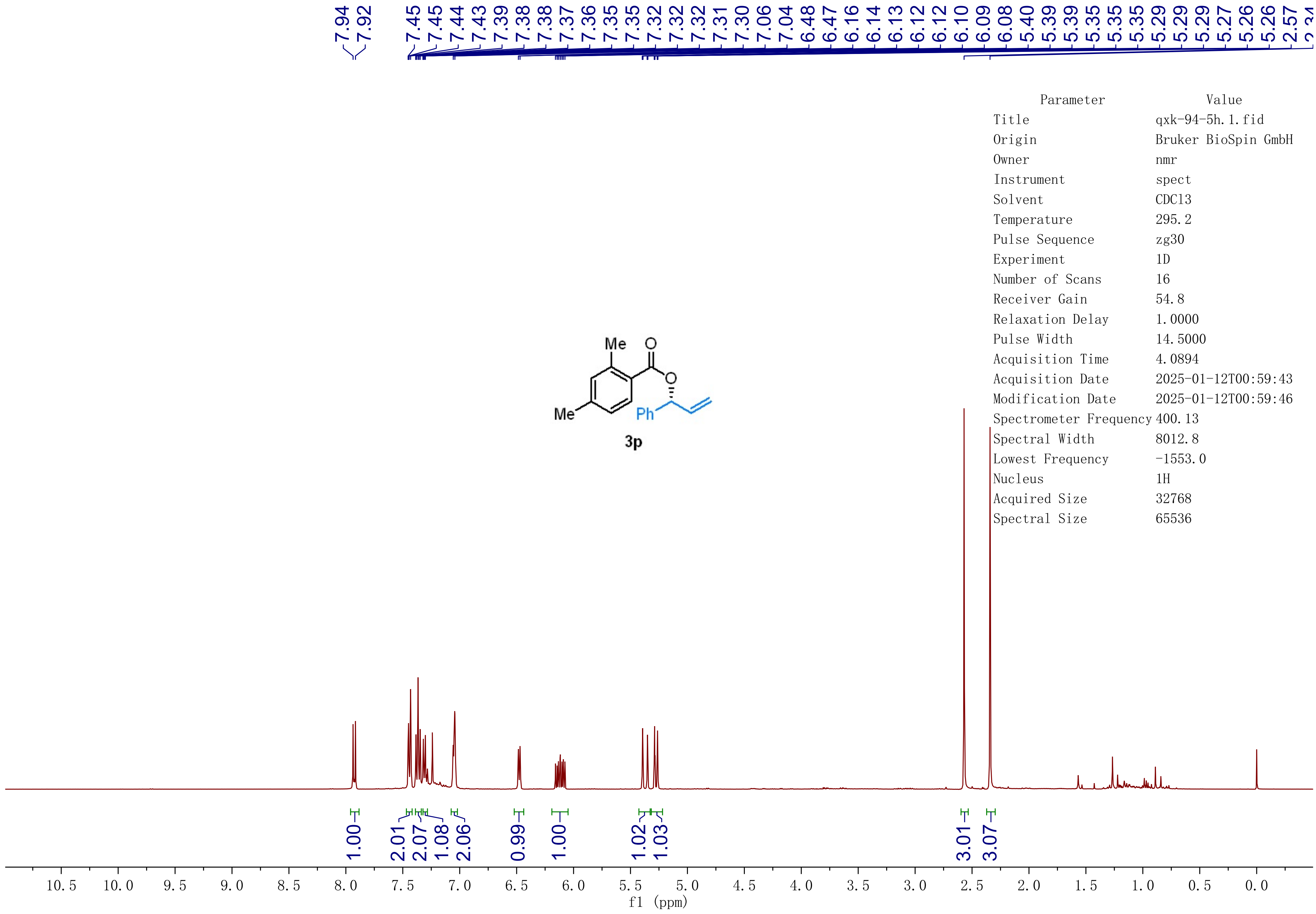
7.94
7.92
7.45
7.45
7.44
7.43
7.39
7.38
7.38
7.37
7.36
7.35
7.35
7.32
7.32
7.32
7.31
7.30
7.06
7.04
6.48
6.47
6.16
6.14
6.13
6.12
6.12
6.10
6.09
6.08
5.40
5.39
5.39
5.35
5.35
5.35
5.29
5.29
5.29
5.27
5.26
5.26
2.57
Parameter Value
Title qxk-94-5h.1.fid
Origin Bruker BioSpin GmbH
Owner nmr
Instrument spect
Solvent CDCl3
Temperature 295.2
Pulse Sequence zg30
Experiment 1D
Number of Scans 16
Receiver Gain 54.8
Relaxation Delay 1.0000
Pulse Width 14.5000
Acquisition Time 4.0894
Acquisition Date 2025-01-12T00:59:43
Modification Date 2025-01-12T00:59:46
Spectrometer Frequency 400.13
Spectral Width 8012.8
Lowest Frequency -1553.0
Nucleus 1H
Acquired Size 32768
Spectral Size 65536
Me
O
O
Me
Ph
3p
1.00
2.01
2.07
1.08
2.06
0.99
1.00
1.02
1.03
3.01
3.07
10.5 10.0 9.5 9.0 8.5 8.0 7.5 7.0 6.5 6.0 5.5 5.0 4.5 4.0 3.5 3.0 2.5 2.0 1.5 1.0 0.5 0.0
f1 (ppm)

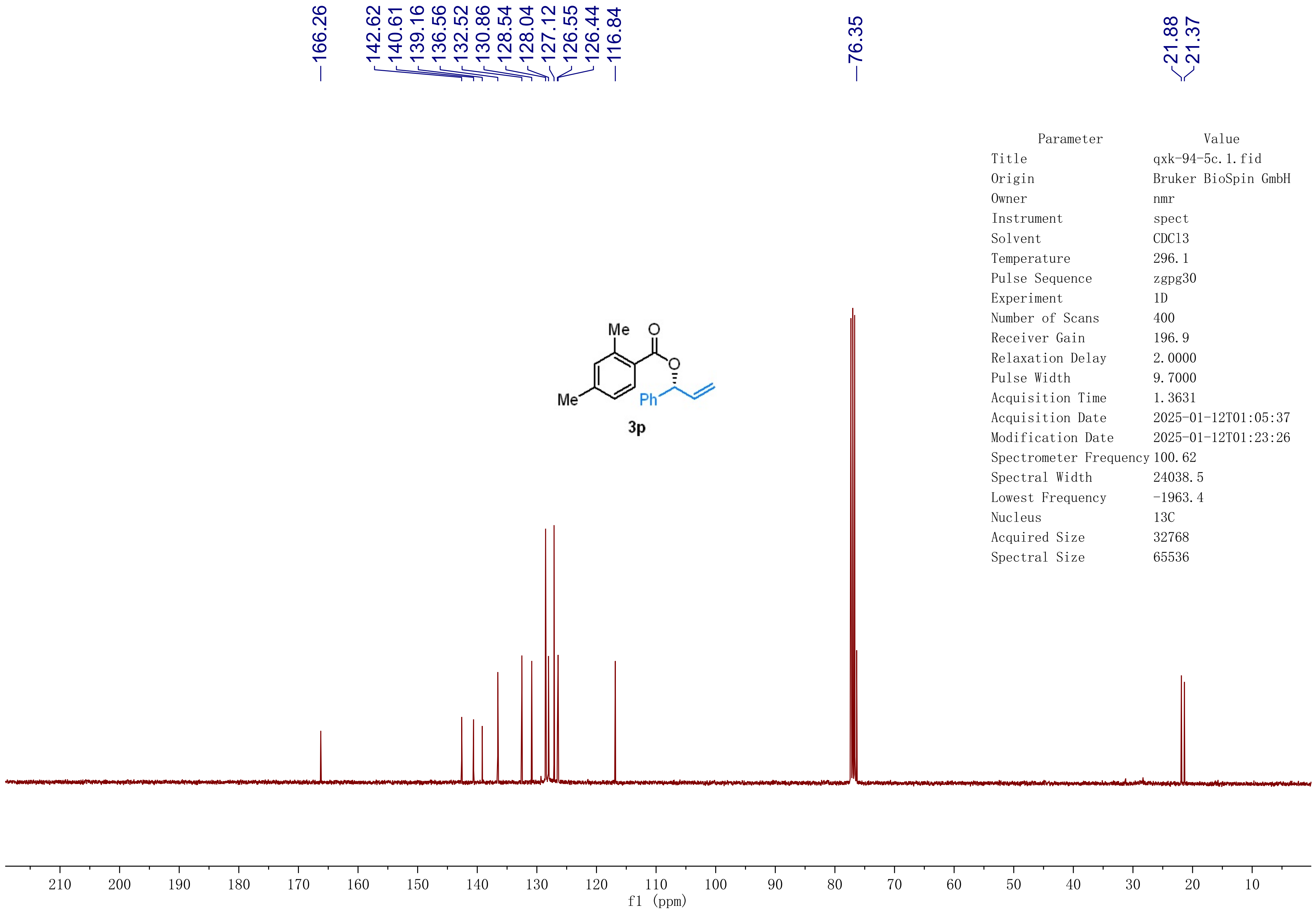

3p

Parameter	Value
Title	qxk-94-5c.1.fid
Origin	Bruker BioSpin GmbH
Owner	nmr
Instrument	spect
Solvent	CDCl3
Temperature	296.1
Pulse Sequence	zgpg30
Experiment	1D
Number of Scans	400
Receiver Gain	196.9
Relaxation Delay	2.0000
Pulse Width	9.7000
Acquisition Time	1.3631
Acquisition Date	2025-01-12T01:05:37
Modification Date	2025-01-12T01:23:26
Spectrometer Frequency	100.62
Spectral Width	24038.5
Lowest Frequency	-1963.4
Nucleus	13C
Acquired Size	32768
Spectral Size	65536

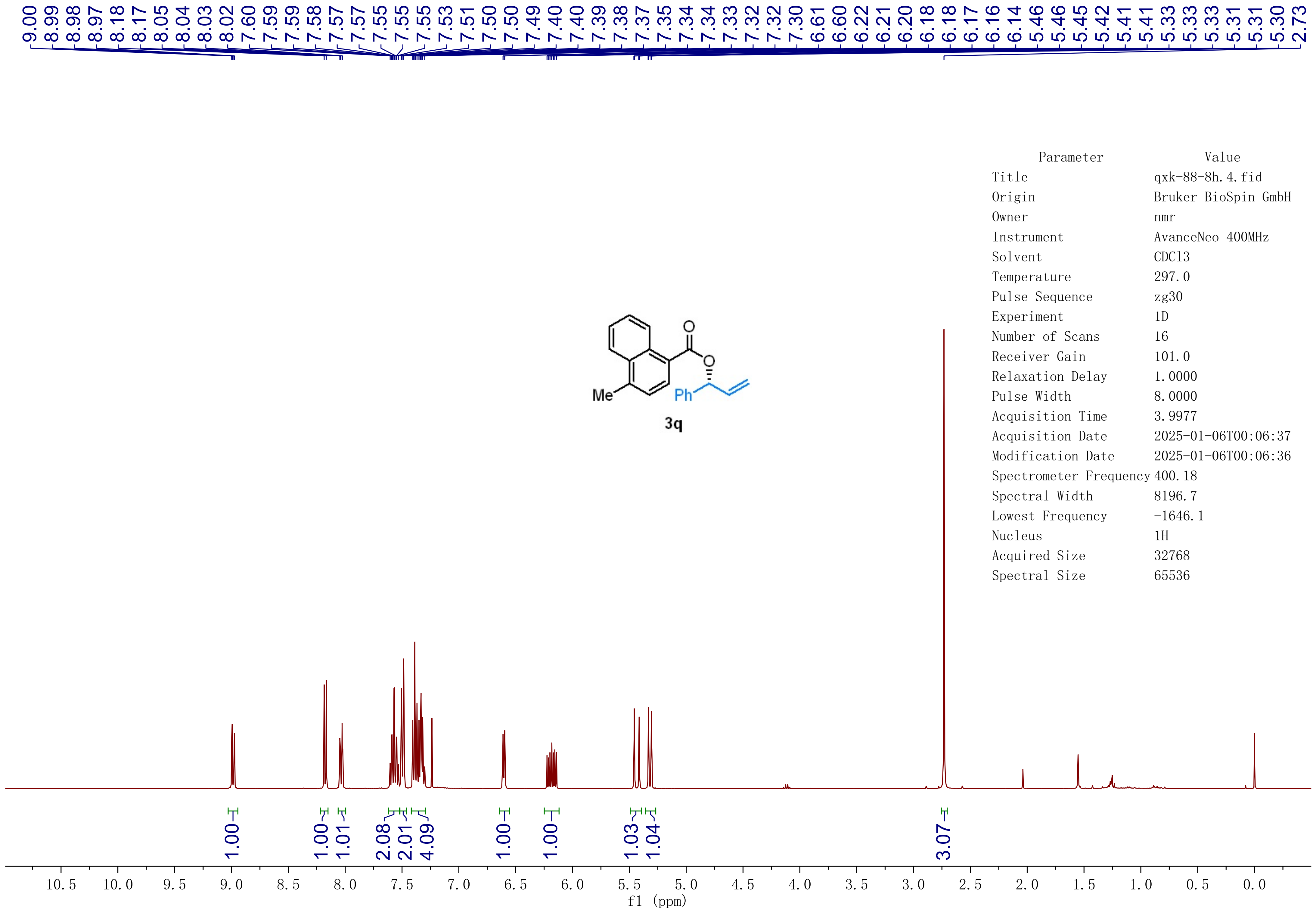

9.00 8.99 8.98 8.97 8.18 8.17 8.05 8.04 8.03 8.02 7.60 7.59 7.59 7.58 7.57 7.57 7.55 7.55 7.55 7.53 7.51 7.50 7.50 7.49 7.40 7.40 7.39 7.38 7.37 7.35 7.34 7.34 7.33 7.32 7.32 7.30 6.61 6.60 6.22 6.21 6.20 6.18 6.18 6.17 6.16 6.14 5.46 5.46 5.45 5.42 5.41 5.41 5.33 5.33 5.33 5.31 5.31 5.30 2.73
O
O
Me
Ph
3q
Parameter Value
Title qxk-88-8h.4.fid
Origin Bruker BioSpin GmbH
Owner nmr
Instrument AvanceNeo 400MHz
Solvent CDCl3
Temperature 297.0
Pulse Sequence zg30
Experiment 1D
Number of Scans 16
Receiver Gain 101.0
Relaxation Delay 1.0000
Pulse Width 8.0000
Acquisition Time 3.9977
Acquisition Date 2025-01-06T00:06:37
Modification Date 2025-01-06T00:06:36
Spectrometer Frequency 400.18
Spectral Width 8196.7
Lowest Frequency -1646.1
Nucleus 1H
Acquired Size 32768
Spectral Size 65536
1.00 1.00 1.01 2.08 2.01 4.09 1.00 1.00 1.03 1.04 3.07
10.5 10.0 9.5 9.0 8.5 8.0 7.5 7.0 6.5 6.0 5.5 5.0 4.5 4.0 3.5 3.0 2.5 2.0 1.5 1.0 0.5 0.0
f1 (ppm)

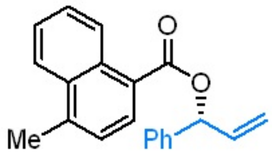

3q

Parameter	Value
Title	qxk-88-8c.5.fid
Origin	Bruker BioSpin GmbH
Owner	nmr
Instrument	AvanceNeo 400MHz
Solvent	CDCl3
Temperature	297.8
Pulse Sequence	zgpg30
Experiment	1D
Number of Scans	400
Receiver Gain	16.0
Relaxation Delay	2.0000
Pulse Width	8.0000
Acquisition Time	1.3763
Acquisition Date	2025-01-06T00:30:52
Modification Date	2025-01-06T00:30:54
Spectrometer Frequency	100.64
Spectral Width	23809.5
Lowest Frequency	-1847.5
Nucleus	13C
Acquired Size	32768
Spectral Size	65536

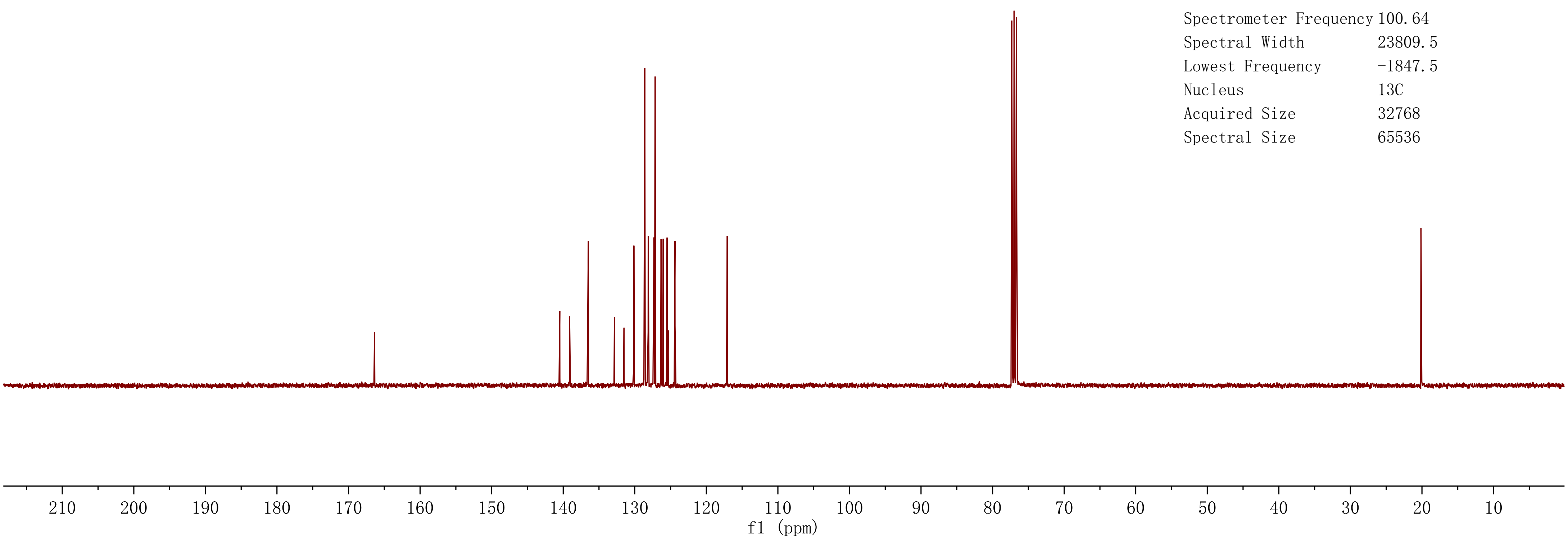

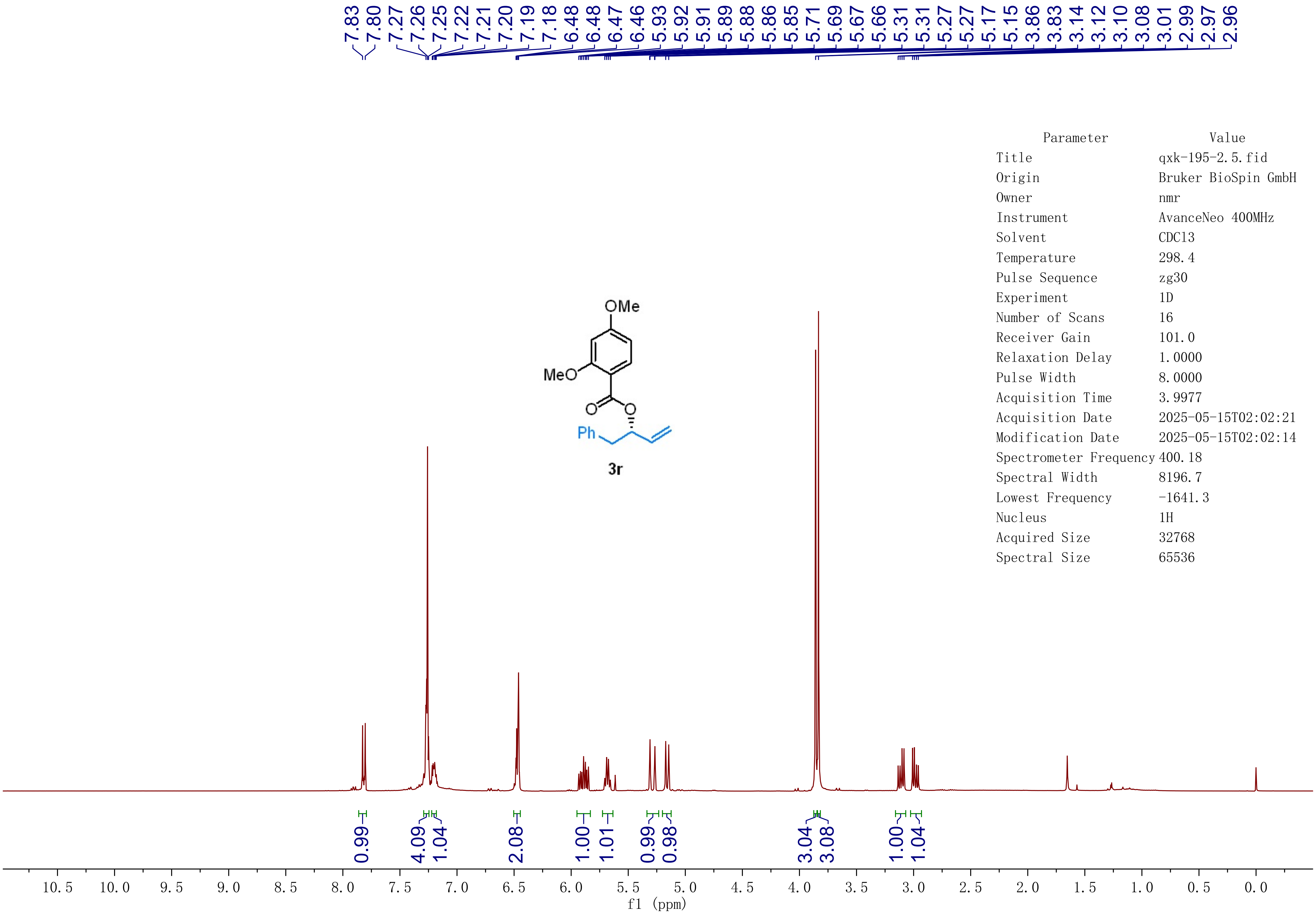

Parameter	Value
Title	qxk-195-2.5.fid
Origin	Bruker BioSpin GmbH
Owner	nmr
Instrument	AvanceNeo 400MHz
Solvent	CDCl3
Temperature	298.4
Pulse Sequence	zg30
Experiment	1D
Number of Scans	16
Receiver Gain	101.0
Relaxation Delay	1.0000
Pulse Width	8.0000
Acquisition Time	3.9977
Acquisition Date	2025-05-15T02:02:21
Modification Date	2025-05-15T02:02:14
Spectrometer Frequency	400.18
Spectral Width	8196.7
Lowest Frequency	-1641.3
Nucleus	1H
Acquired Size	32768
Spectral Size	65536

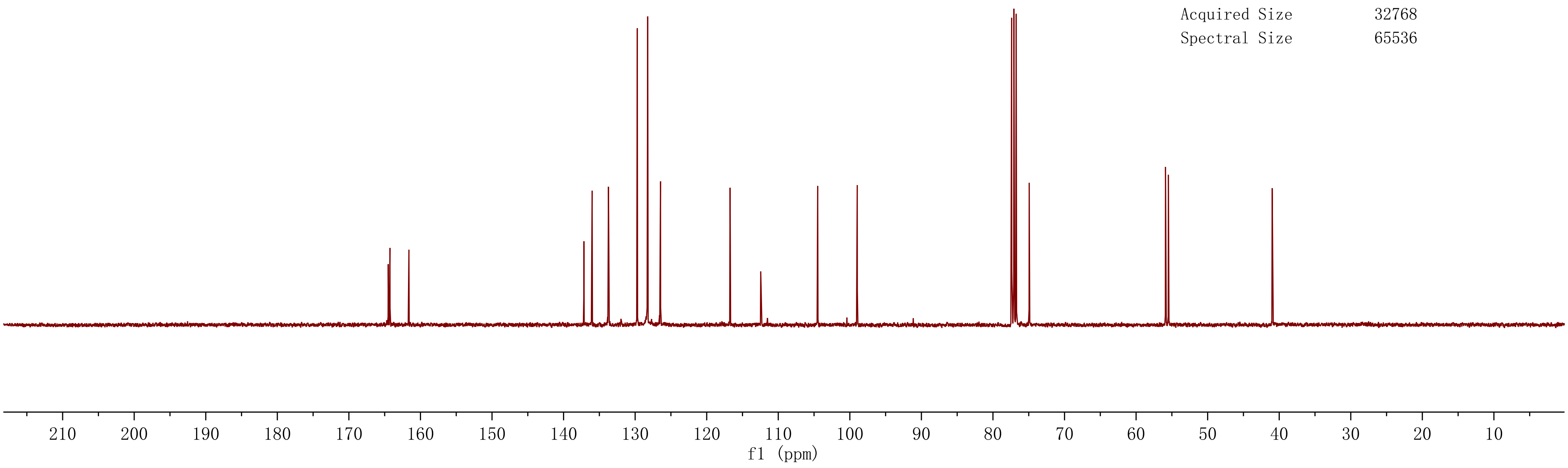

Parameter	Value
Title	qxk-195-2.6.fid
Origin	Bruker BioSpin GmbH
Owner	nmr
Instrument	AvanceNeo 400MHz
Solvent	CDCl3
Temperature	299.1
Pulse Sequence	zgpg30
Experiment	1D
Number of Scans	400
Receiver Gain	16.0
Relaxation Delay	2.0000
Pulse Width	8.0000
Acquisition Time	1.3763
Acquisition Date	2025-05-15T02:26:43
Modification Date	2025-05-15T02:26:36
Spectrometer Frequency	100.64
Spectral Width	23809.5
Lowest Frequency	-1842.2
Nucleus	13C
Acquired Size	32768
Spectral Size	65536

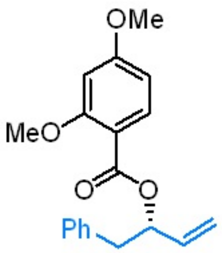

3r

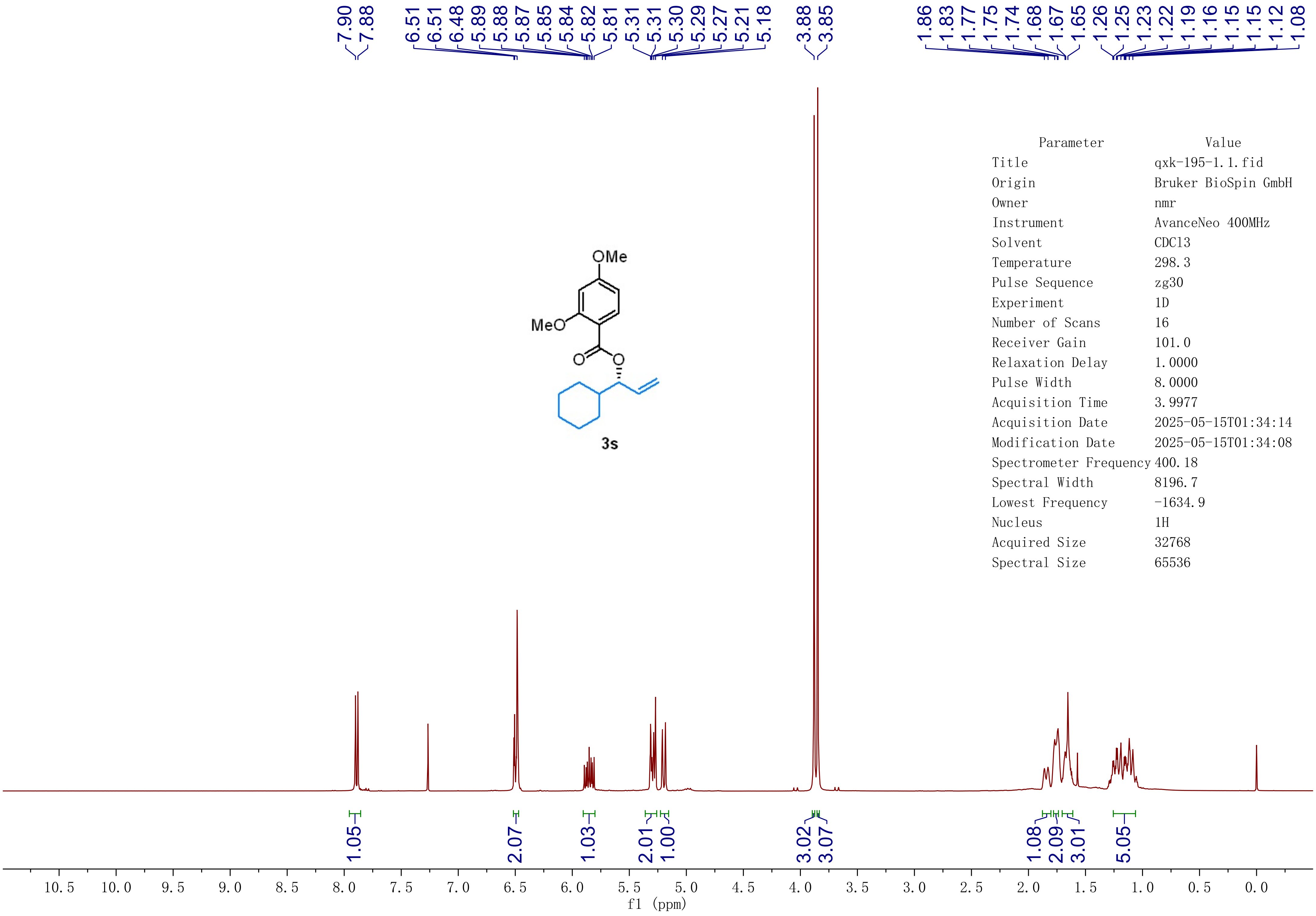

Parameter	Value
Title	qxk-195-1.1.fid
Origin	Bruker BioSpin GmbH
Owner	nmr
Instrument	AvanceNeo 400MHz
Solvent	CDCl3
Temperature	298.3
Pulse Sequence	zg30
Experiment	1D
Number of Scans	16
Receiver Gain	101.0
Relaxation Delay	1.0000
Pulse Width	8.0000
Acquisition Time	3.9977
Acquisition Date	2025-05-15T01:34:14
Modification Date	2025-05-15T01:34:08
Spectrometer Frequency	400.18
Spectral Width	8196.7
Lowest Frequency	-1634.9
Nucleus	1H
Acquired Size	32768
Spectral Size	65536

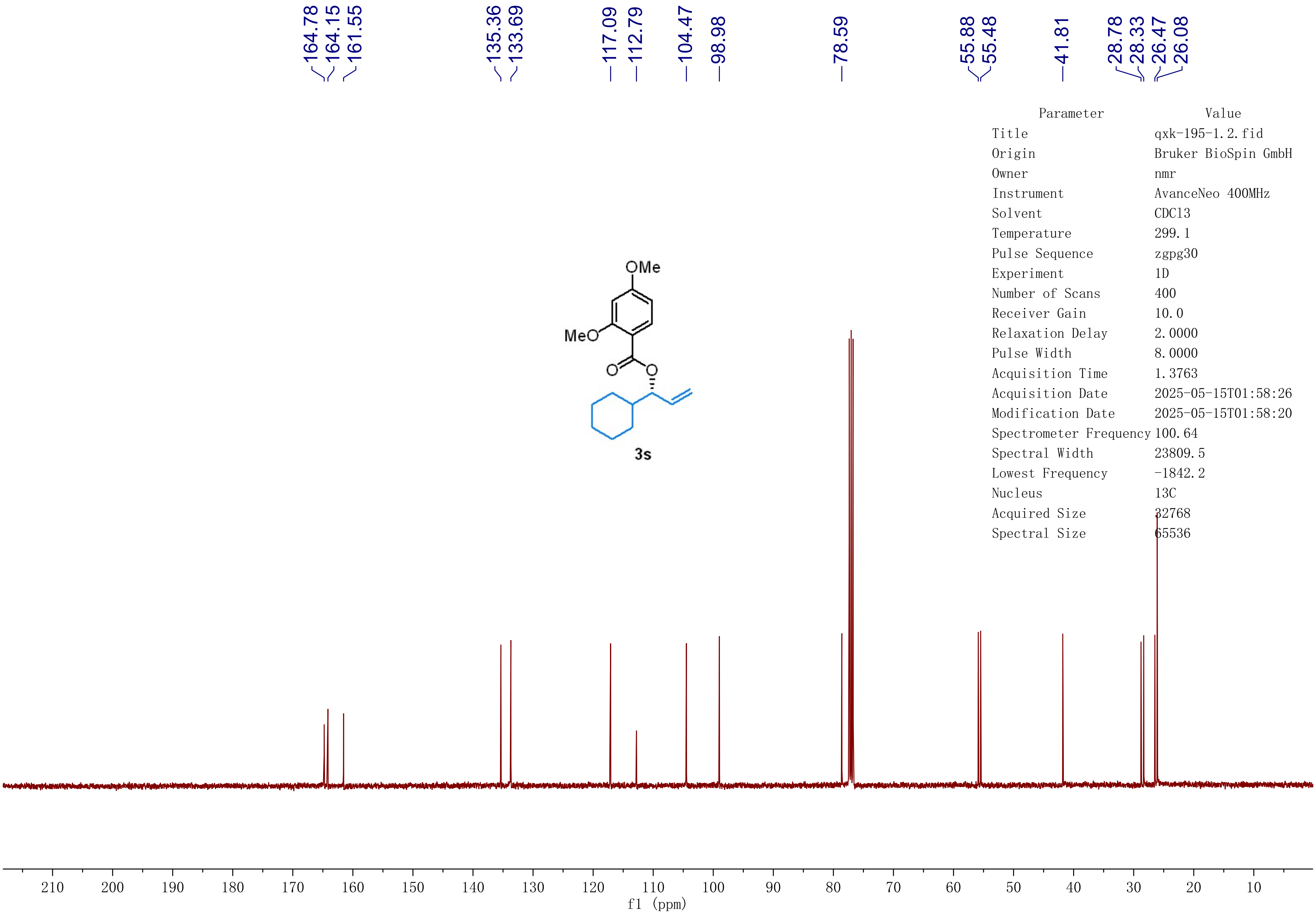

3s

Parameter	Value
Title	qxk-195-1.2.fid
Origin	Bruker BioSpin GmbH
Owner	nmr
Instrument	AvanceNeo 400MHz
Solvent	CDCl3
Temperature	299.1
Pulse Sequence	zgpg30
Experiment	1D
Number of Scans	400
Receiver Gain	10.0
Relaxation Delay	2.0000
Pulse Width	8.0000
Acquisition Time	1.3763
Acquisition Date	2025-05-15T01:58:26
Modification Date	2025-05-15T01:58:20
Spectrometer Frequency	100.64
Spectral Width	23809.5
Lowest Frequency	-1842.2
Nucleus	13C
Acquired Size	32768
Spectral Size	65536

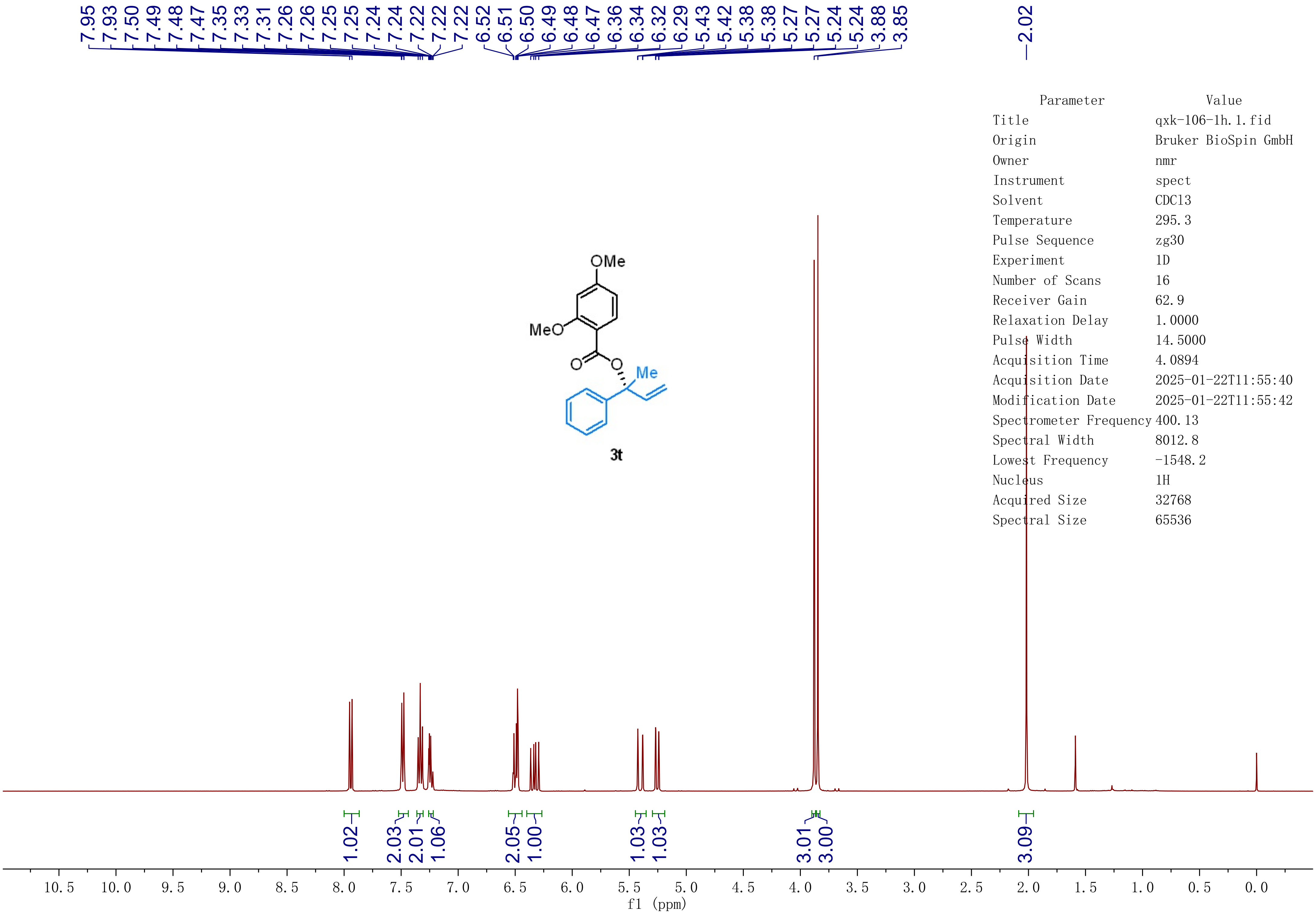

Parameter	Value
Title	qxk-106-1h.1.fid
Origin	Bruker BioSpin GmbH
Owner	nmr
Instrument	spect
Solvent	CDCl3
Temperature	295.3
Pulse Sequence	zg30
Experiment	1D
Number of Scans	16
Receiver Gain	62.9
Relaxation Delay	1.0000
Pulse Width	14.5000
Acquisition Time	4.0894
Acquisition Date	2025-01-22T11:55:40
Modification Date	2025-01-22T11:55:42
Spectrometer Frequency	400.13
Spectral Width	8012.8
Lowest Frequency	-1548.2
Nucleus	1H
Acquired Size	32768
Spectral Size	65536

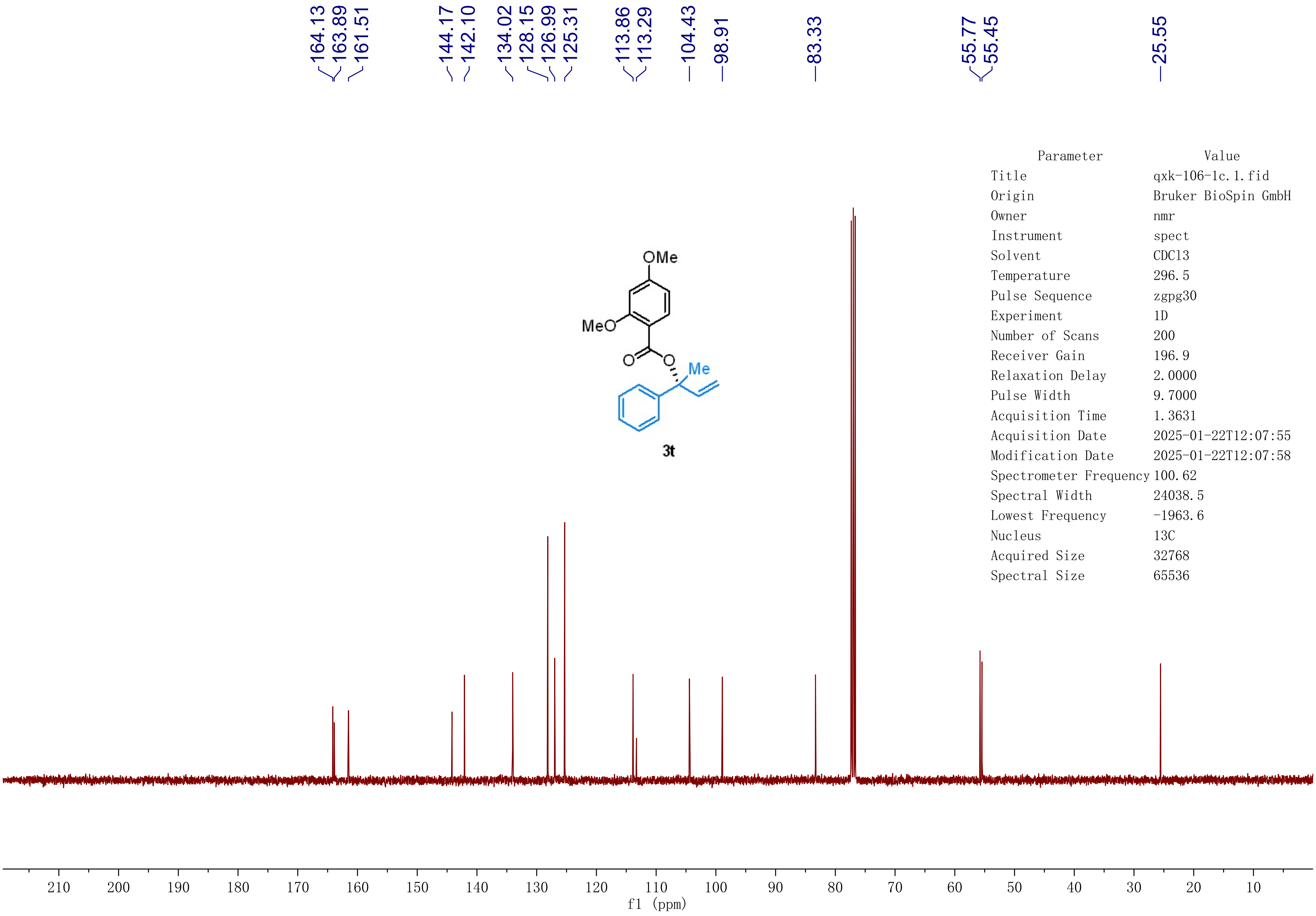

Parameter	Value
Title	qxk-106-1c.1.fid
Origin	Bruker BioSpin GmbH
Owner	nmr
Instrument	spect
Solvent	CDCl3
Temperature	296.5
Pulse Sequence	zgpg30
Experiment	1D
Number of Scans	200
Receiver Gain	196.9
Relaxation Delay	2.0000
Pulse Width	9.7000
Acquisition Time	1.3631
Acquisition Date	2025-01-22T12:07:55
Modification Date	2025-01-22T12:07:58
Spectrometer Frequency	100.62
Spectral Width	24038.5
Lowest Frequency	-1963.6
Nucleus	13C
Acquired Size	32768
Spectral Size	65536

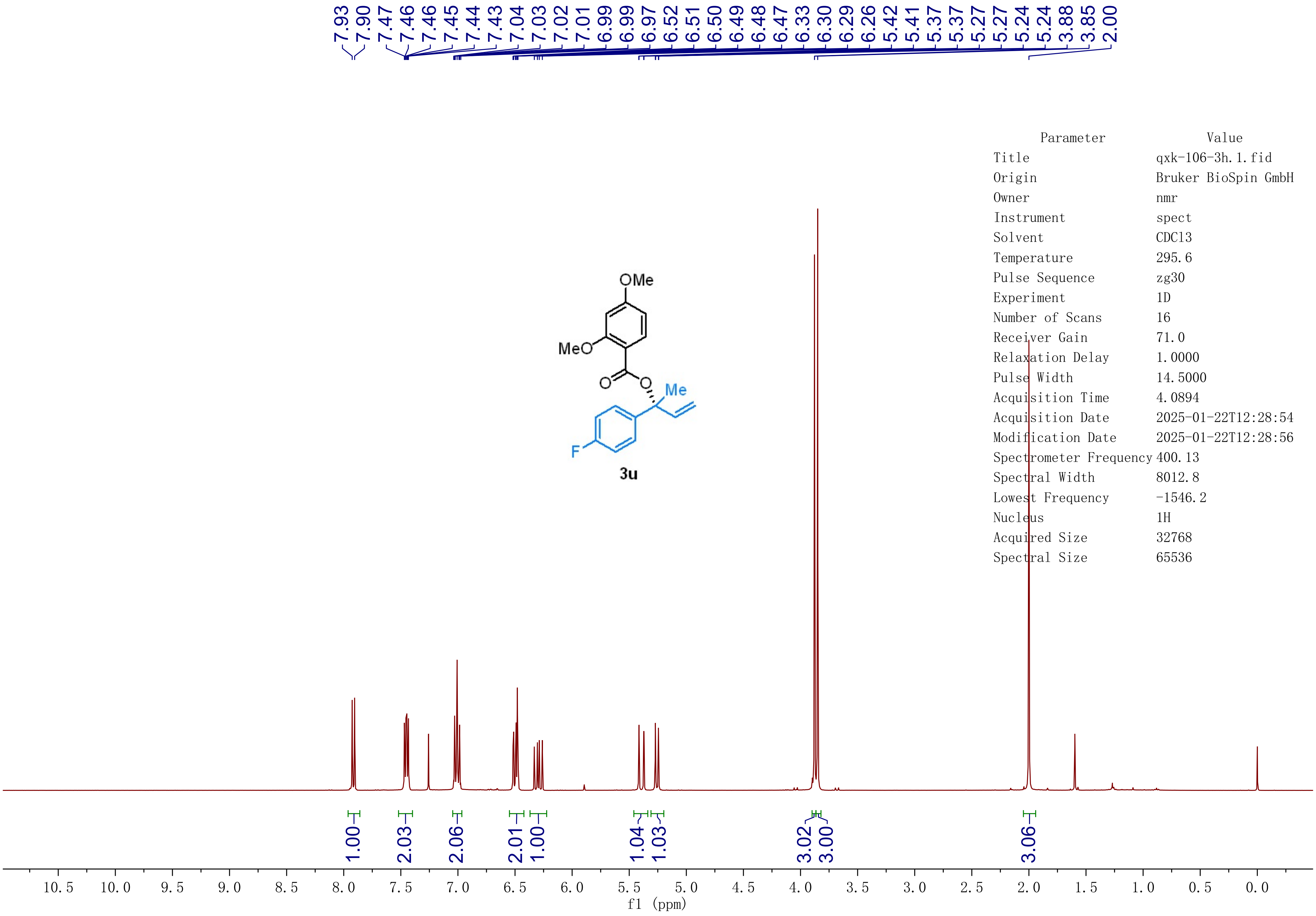

Parameter	Value
Title	qxk-106-3h.1.fid
Origin	Bruker BioSpin GmbH
Owner	nmr
Instrument	spect
Solvent	CDCl3
Temperature	295.6
Pulse Sequence	zg30
Experiment	1D
Number of Scans	16
Receiver Gain	71.0
Relaxation Delay	1.0000
Pulse Width	14.5000
Acquisition Time	4.0894
Acquisition Date	2025-01-22T12:28:54
Modification Date	2025-01-22T12:28:56
Spectrometer Frequency	400.13
Spectral Width	8012.8
Lowest Frequency	-1546.2
Nucleus	1H
Acquired Size	32768
Spectral Size	65536

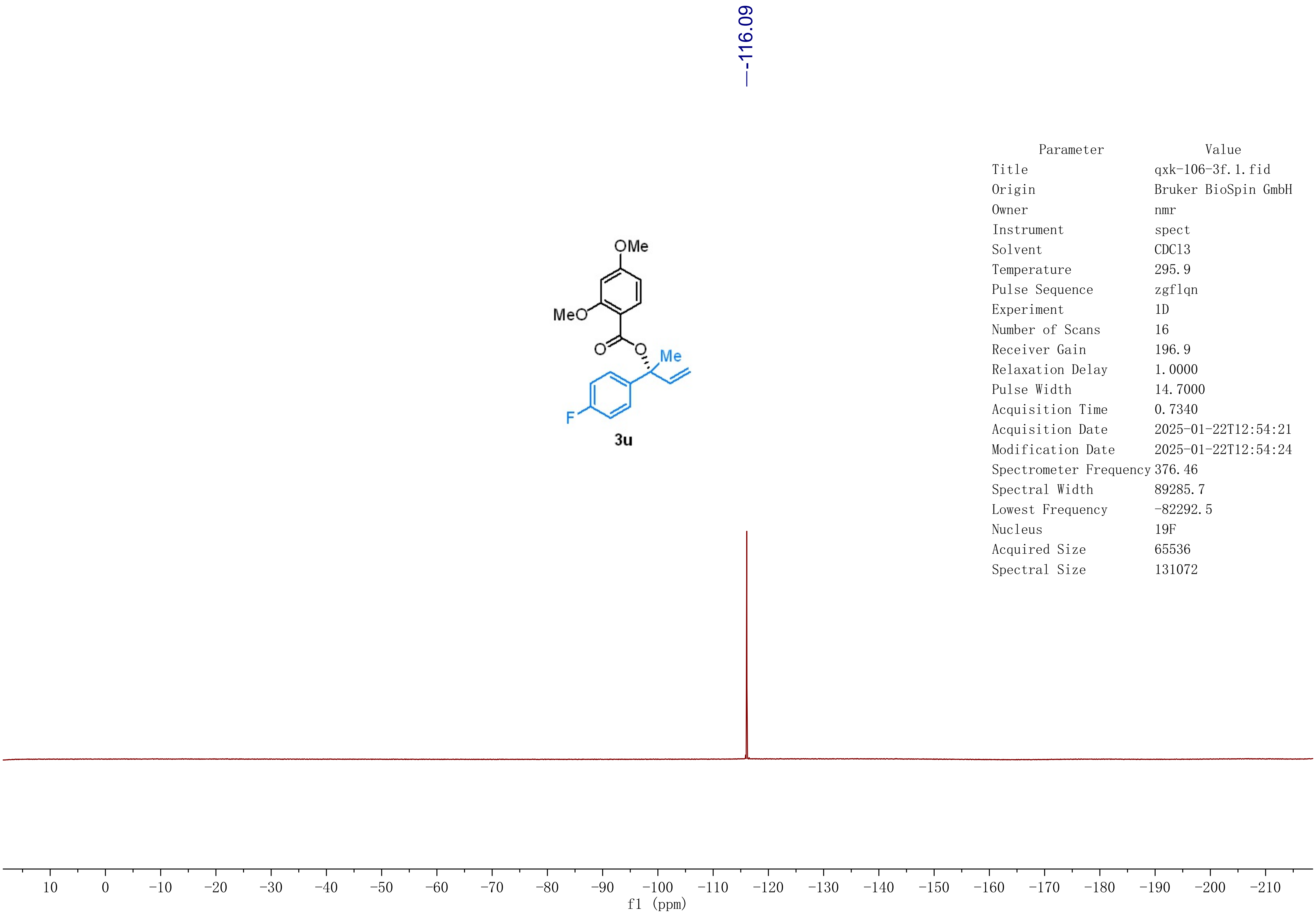

Parameter	Value
Title	qxk-106-3f.1.fid
Origin	Bruker BioSpin GmbH
Owner	nmr
Instrument	spect
Solvent	CDCl3
Temperature	295.9
Pulse Sequence	zgflqn
Experiment	1D
Number of Scans	16
Receiver Gain	196.9
Relaxation Delay	1.0000
Pulse Width	14.7000
Acquisition Time	0.7340
Acquisition Date	2025-01-22T12:54:21
Modification Date	2025-01-22T12:54:24
Spectrometer Frequency	376.46
Spectral Width	89285.7
Lowest Frequency	-82292.5
Nucleus	19F
Acquired Size	65536
Spectral Size	131072

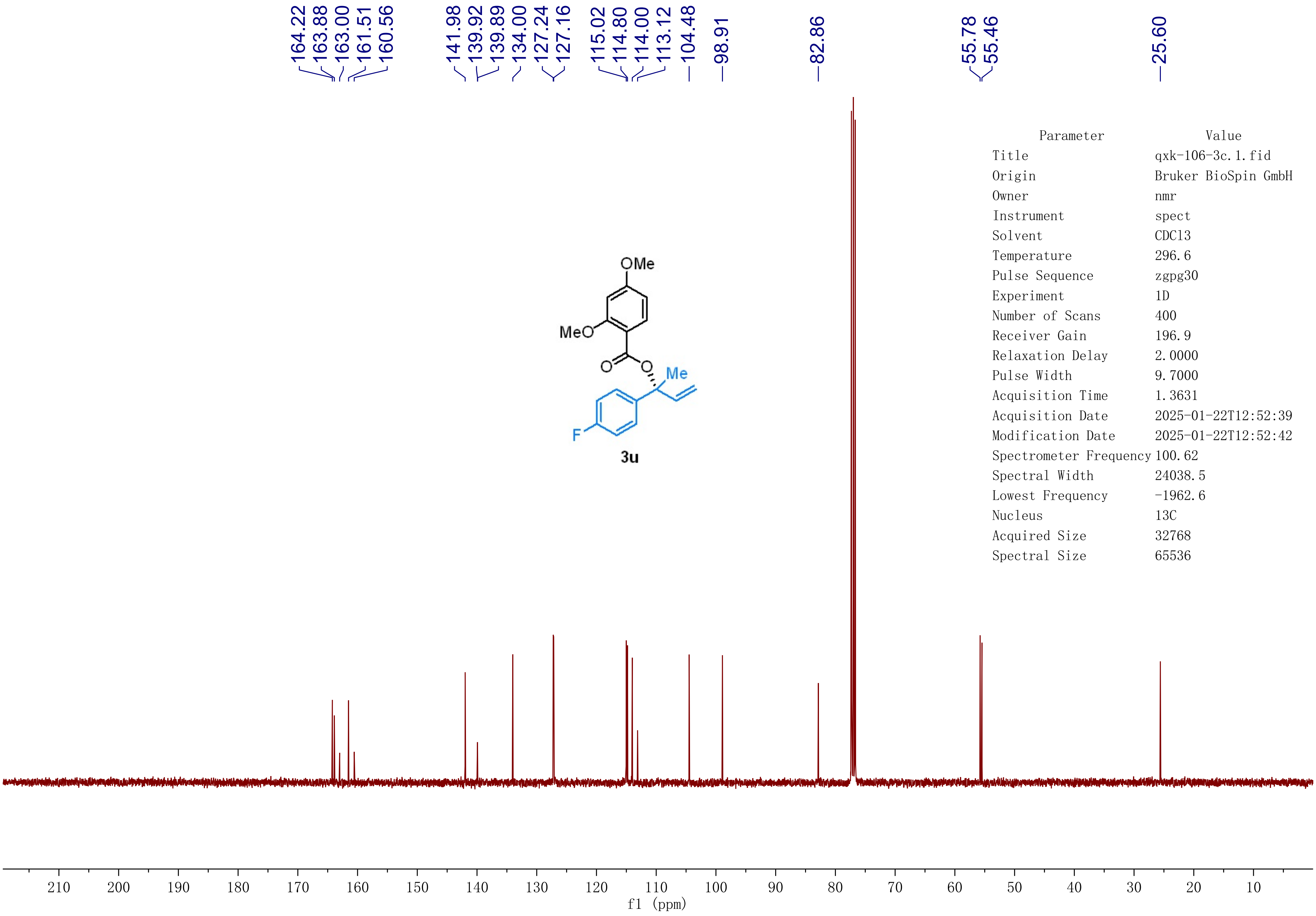
164.22
163.88
163.00
161.51
160.56
141.98
139.92
139.89
134.00
127.24
127.16
115.02
114.80
114.00
113.12
104.48
98.91
82.86
55.78
55.46
25.60
OMe
MeO
O
O
Me
F
3u
f1 (ppm)
Parameter Value
Title qxk-106-3c.1.fid
Origin Bruker BioSpin GmbH
Owner nmr
Instrument spect
Solvent CDCl3
Temperature 296.6
Pulse Sequence zgpg30
Experiment 1D
Number of Scans 400
Receiver Gain 196.9
Relaxation Delay 2.0000
Pulse Width 9.7000
Acquisition Time 1.3631
Acquisition Date 2025-01-22T12:52:39
Modification Date 2025-01-22T12:52:42
Spectrometer Frequency 100.62
Spectral Width 24038.5
Lowest Frequency -1962.6
Nucleus 13C
Acquired Size 32768
Spectral Size 65536

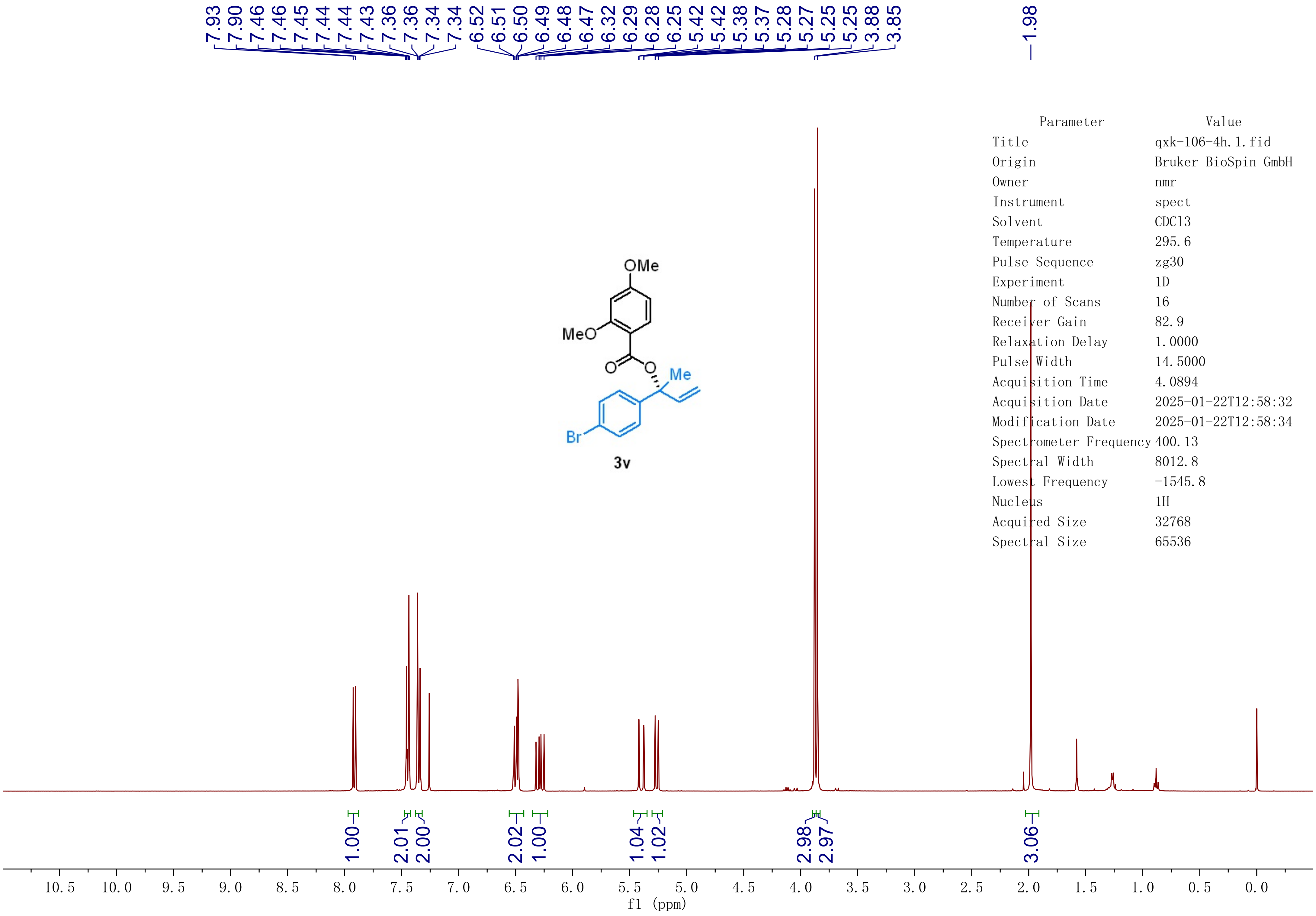

3v

Parameter	Value
Title	qxk-106-4h.1.fid
Origin	Bruker BioSpin GmbH
Owner	nmr
Instrument	spect
Solvent	CDCl3
Temperature	295.6
Pulse Sequence	zg30
Experiment	1D
Number of Scans	16
Receiver Gain	82.9
Relaxation Delay	1.0000
Pulse Width	14.5000
Acquisition Time	4.0894
Acquisition Date	2025-01-22T12:58:32
Modification Date	2025-01-22T12:58:34
Spectrometer Frequency	400.13
Spectral Width	8012.8
Lowest Frequency	-1545.8
Nucleus	1H
Acquired Size	32768
Spectral Size	65536

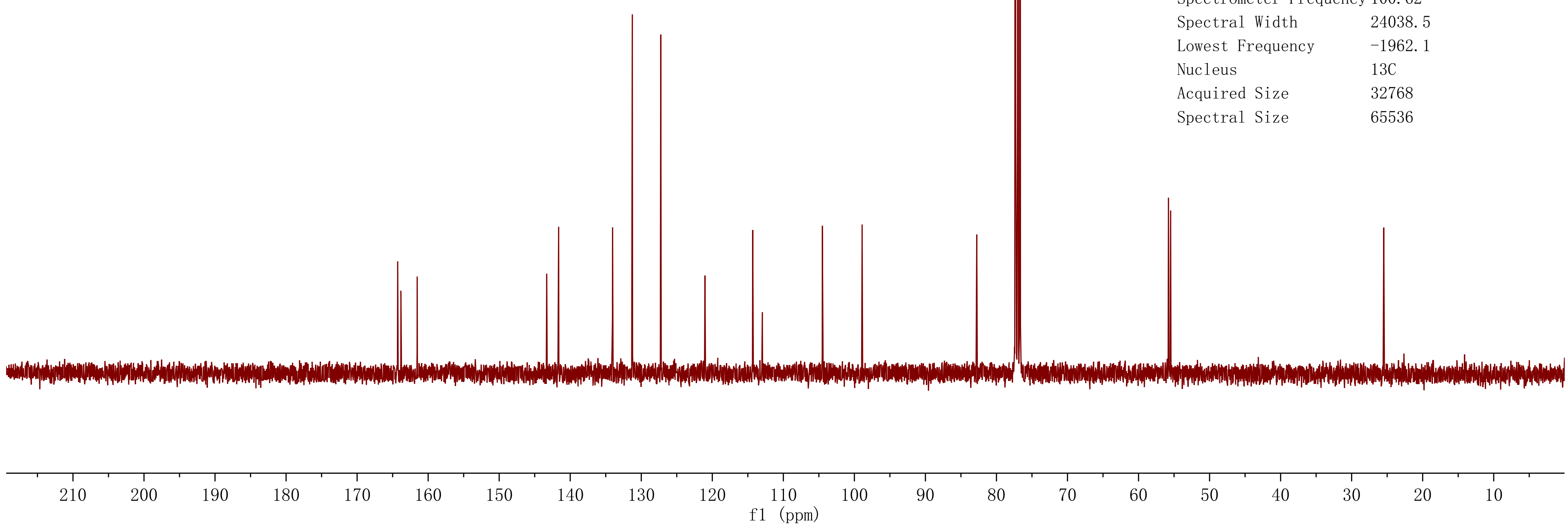

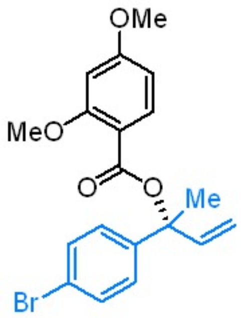

3v

Parameter	Value
Title	qxk-106-4c.1.fid
Origin	Bruker BioSpin GmbH
Owner	nmr
Instrument	spect
Solvent	CDCl3
Temperature	296.5
Pulse Sequence	zgpg30
Experiment	1D
Number of Scans	200
Receiver Gain	196.9
Relaxation Delay	2.0000
Pulse Width	9.7000
Acquisition Time	1.3631
Acquisition Date	2025-01-22T13:11:04
Modification Date	2025-01-22T13:11:06
Spectrometer Frequency	100.62
Spectral Width	24038.5
Lowest Frequency	-1962.1
Nucleus	13C
Acquired Size	32768
Spectral Size	65536

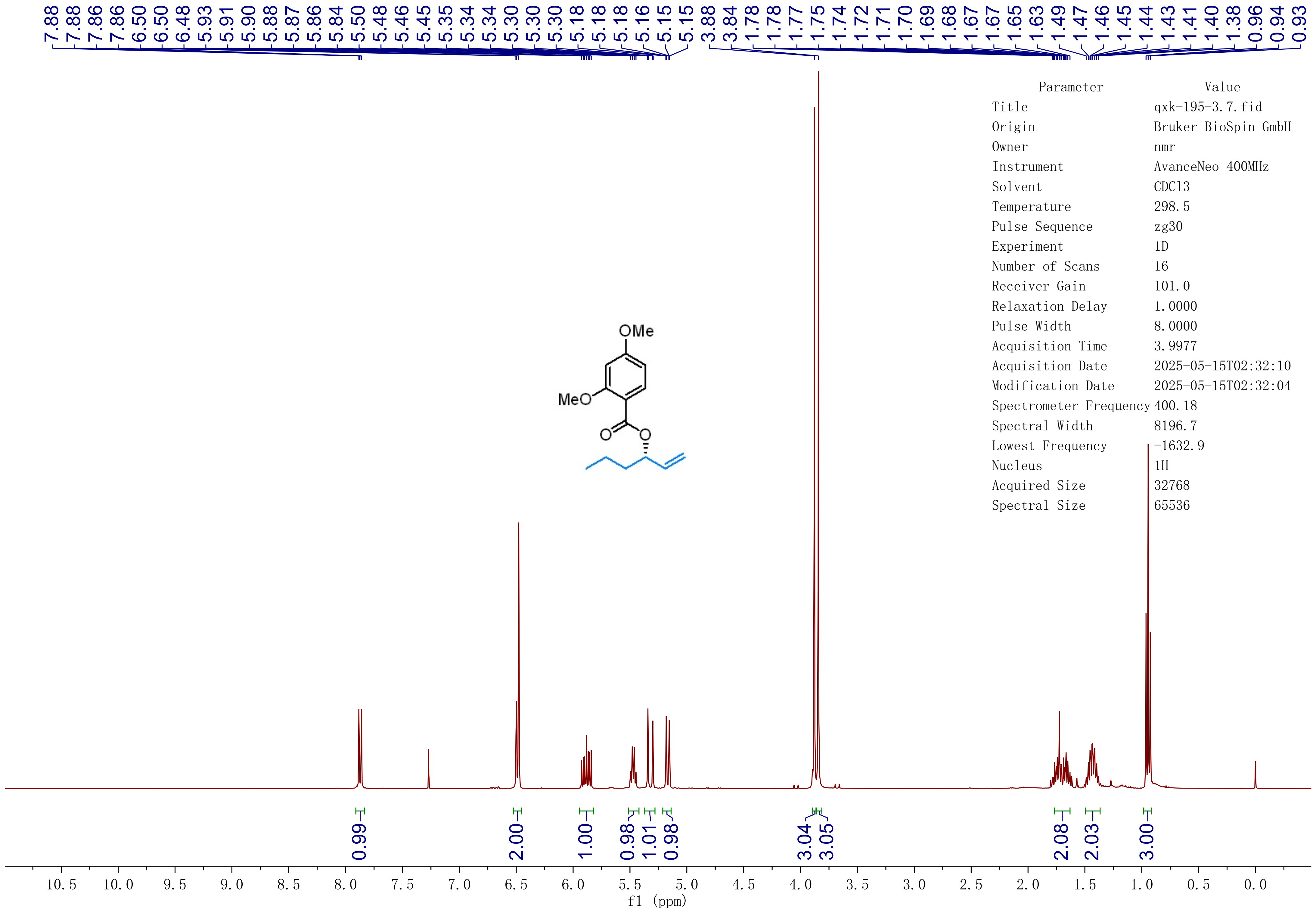

Parameter	Value
Title	qxk-195-3.7.fid
Origin	Bruker BioSpin GmbH
Owner	nmr
Instrument	AvanceNeo 400MHz
Solvent	CDCl3
Temperature	298.5
Pulse Sequence	zg30
Experiment	1D
Number of Scans	16
Receiver Gain	101.0
Relaxation Delay	1.0000
Pulse Width	8.0000
Acquisition Time	3.9977
Acquisition Date	2025-05-15T02:32:10
Modification Date	2025-05-15T02:32:04
Spectrometer Frequency	400.18
Spectral Width	8196.7
Lowest Frequency	-1632.9
Nucleus	1H
Acquired Size	32768
Spectral Size	65536

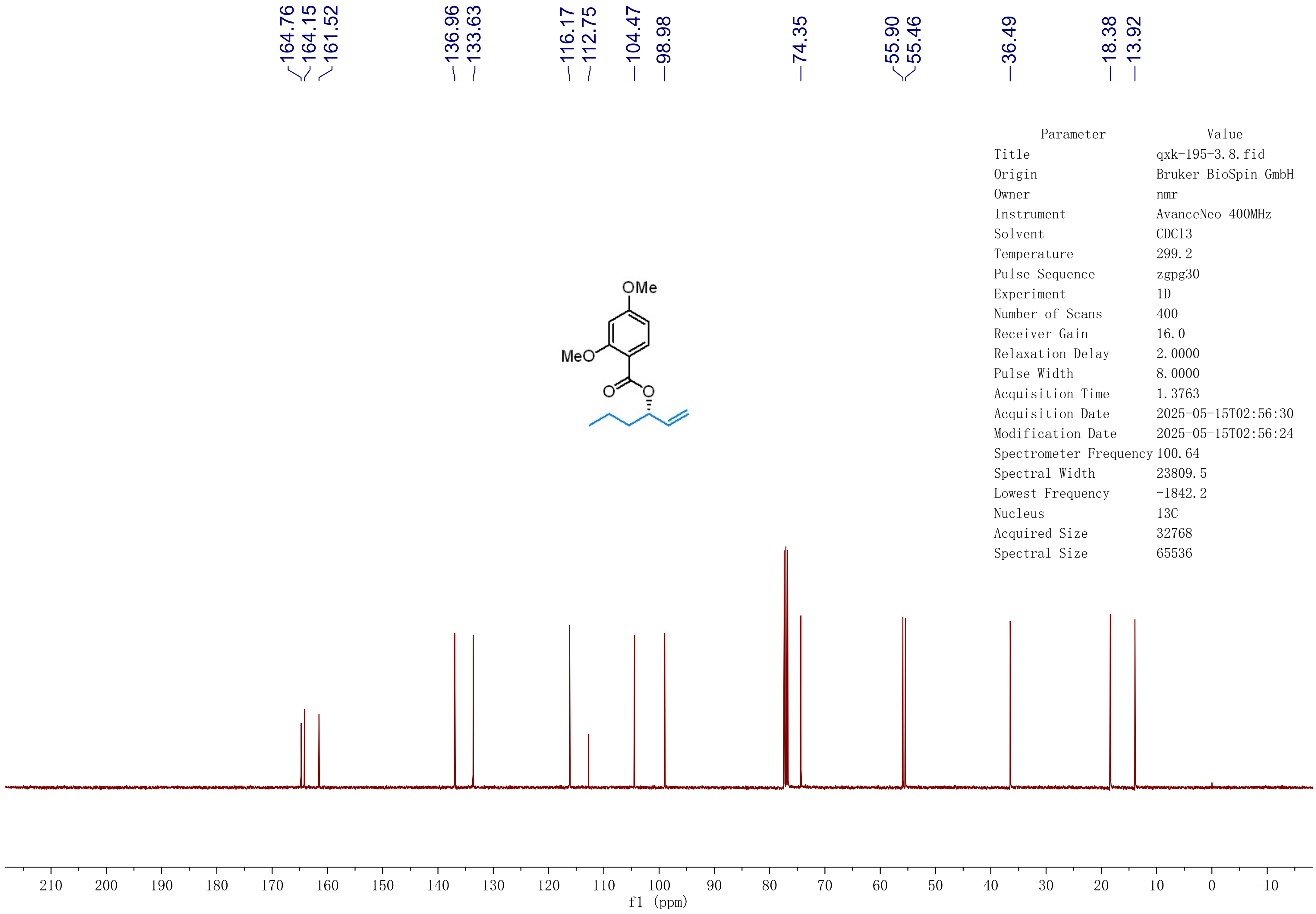

Parameter	Value
Title	qxk-195-3.8.fid
Origin	Bruker BioSpin GmbH
Owner	nmr
Instrument	AvanceNeo 400MHz
Solvent	CDCl3
Temperature	299.2
Pulse Sequence	zgpg30
Experiment	1D
Number of Scans	400
Receiver Gain	16.0
Relaxation Delay	2.0000
Pulse Width	8.0000
Acquisition Time	1.3763
Acquisition Date	2025-05-15T02:56:30
Modification Date	2025-05-15T02:56:24
Spectrometer Frequency	100.64
Spectral Width	23809.5
Lowest Frequency	-1842.2
Nucleus	13C
Acquired Size	32768
Spectral Size	65536

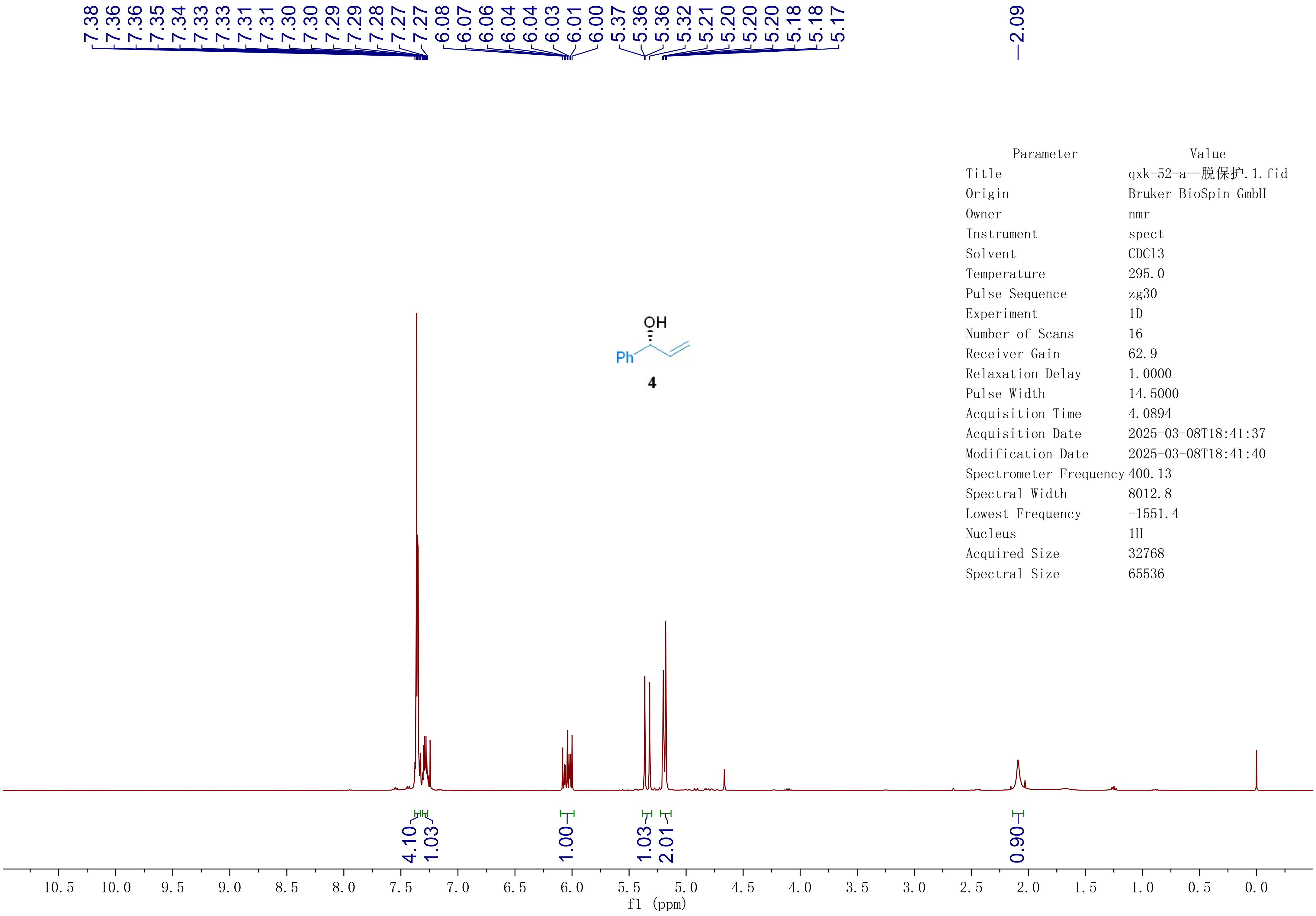

4

Parameter	Value
Title	qxk-52-a--脱保护.1.fid
Origin	Bruker BioSpin GmbH
Owner	nmr
Instrument	spect
Solvent	CDCl3
Temperature	295.0
Pulse Sequence	zg30
Experiment	1D
Number of Scans	16
Receiver Gain	62.9
Relaxation Delay	1.0000
Pulse Width	14.5000
Acquisition Time	4.0894
Acquisition Date	2025-03-08T18:41:37
Modification Date	2025-03-08T18:41:40
Spectrometer Frequency	400.13
Spectral Width	8012.8
Lowest Frequency	-1551.4
Nucleus	1H
Acquired Size	32768
Spectral Size	65536

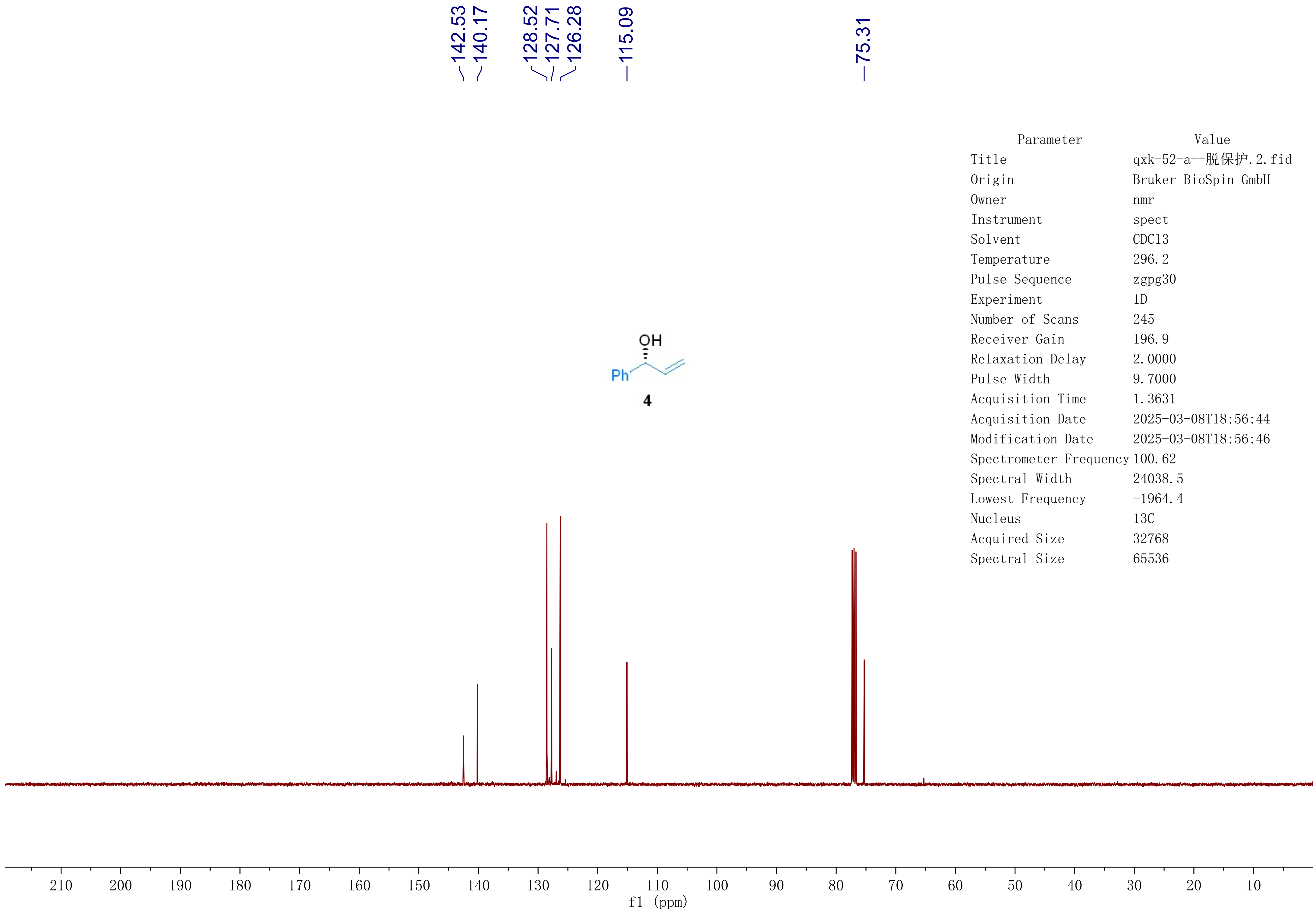

4

Parameter	Value
Title	qxk-52-a--脱保护.2.fid
Origin	Bruker BioSpin GmbH
Owner	nmr
Instrument	spect
Solvent	CDCl3
Temperature	296.2
Pulse Sequence	zgpg30
Experiment	1D
Number of Scans	245
Receiver Gain	196.9
Relaxation Delay	2.0000
Pulse Width	9.7000
Acquisition Time	1.3631
Acquisition Date	2025-03-08T18:56:44
Modification Date	2025-03-08T18:56:46
Spectrometer Frequency	100.62
Spectral Width	24038.5
Lowest Frequency	-1964.4
Nucleus	13C
Acquired Size	32768
Spectral Size	65536

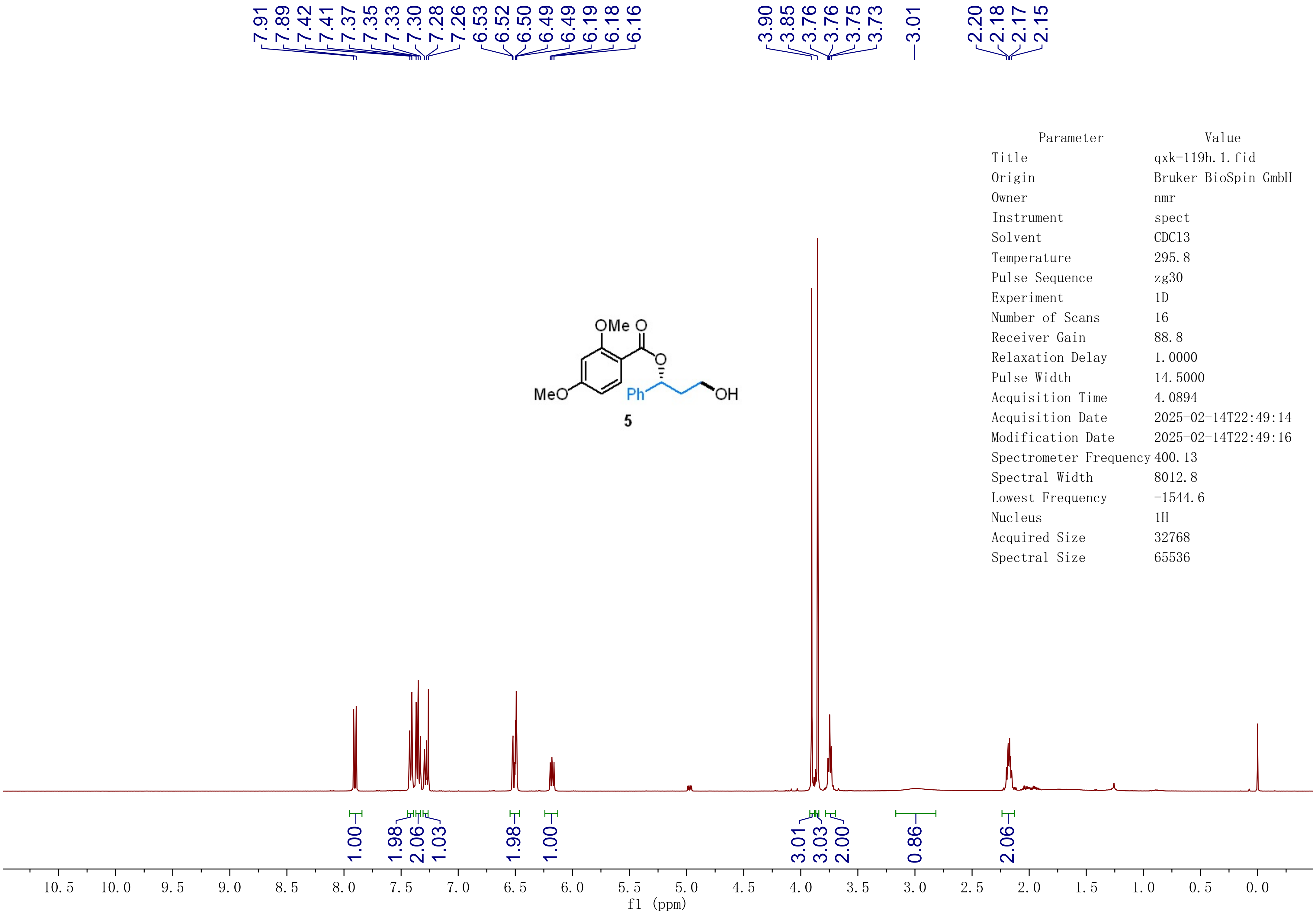

Parameter	Value
Title	qxk-119h.1.fid
Origin	Bruker BioSpin GmbH
Owner	nmr
Instrument	spect
Solvent	CDCl3
Temperature	295.8
Pulse Sequence	zg30
Experiment	1D
Number of Scans	16
Receiver Gain	88.8
Relaxation Delay	1.0000
Pulse Width	14.5000
Acquisition Time	4.0894
Acquisition Date	2025-02-14T22:49:14
Modification Date	2025-02-14T22:49:16
Spectrometer Frequency	400.13
Spectral Width	8012.8
Lowest Frequency	-1544.6
Nucleus	1H
Acquired Size	32768
Spectral Size	65536

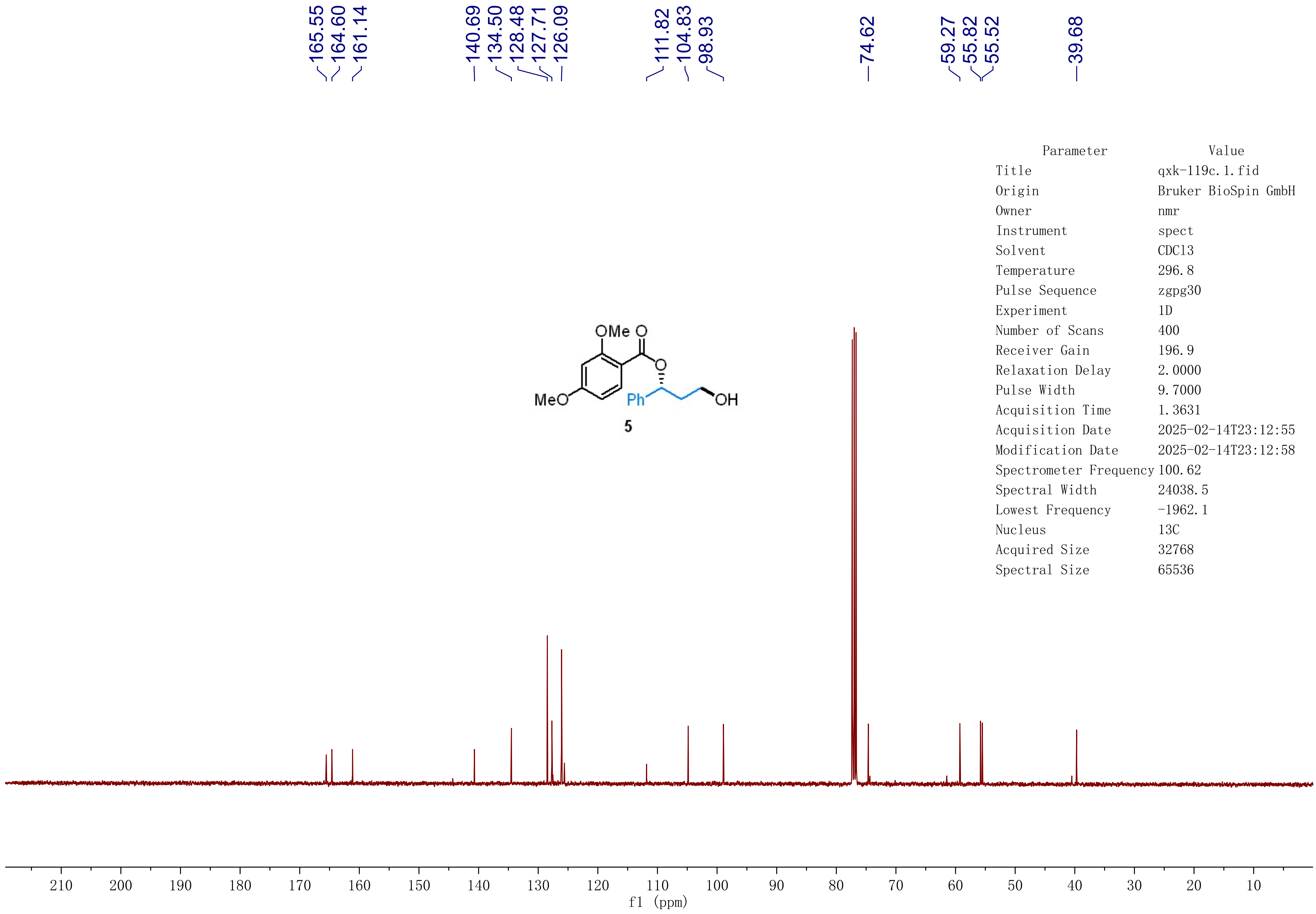

Parameter	Value
Title	qxk-119c.1.fid
Origin	Bruker BioSpin GmbH
Owner	nmr
Instrument	spect
Solvent	CDCl3
Temperature	296.8
Pulse Sequence	zgpg30
Experiment	1D
Number of Scans	400
Receiver Gain	196.9
Relaxation Delay	2.0000
Pulse Width	9.7000
Acquisition Time	1.3631
Acquisition Date	2025-02-14T23:12:55
Modification Date	2025-02-14T23:12:58
Spectrometer Frequency	100.62
Spectral Width	24038.5
Lowest Frequency	-1962.1
Nucleus	13C
Acquired Size	32768
Spectral Size	65536

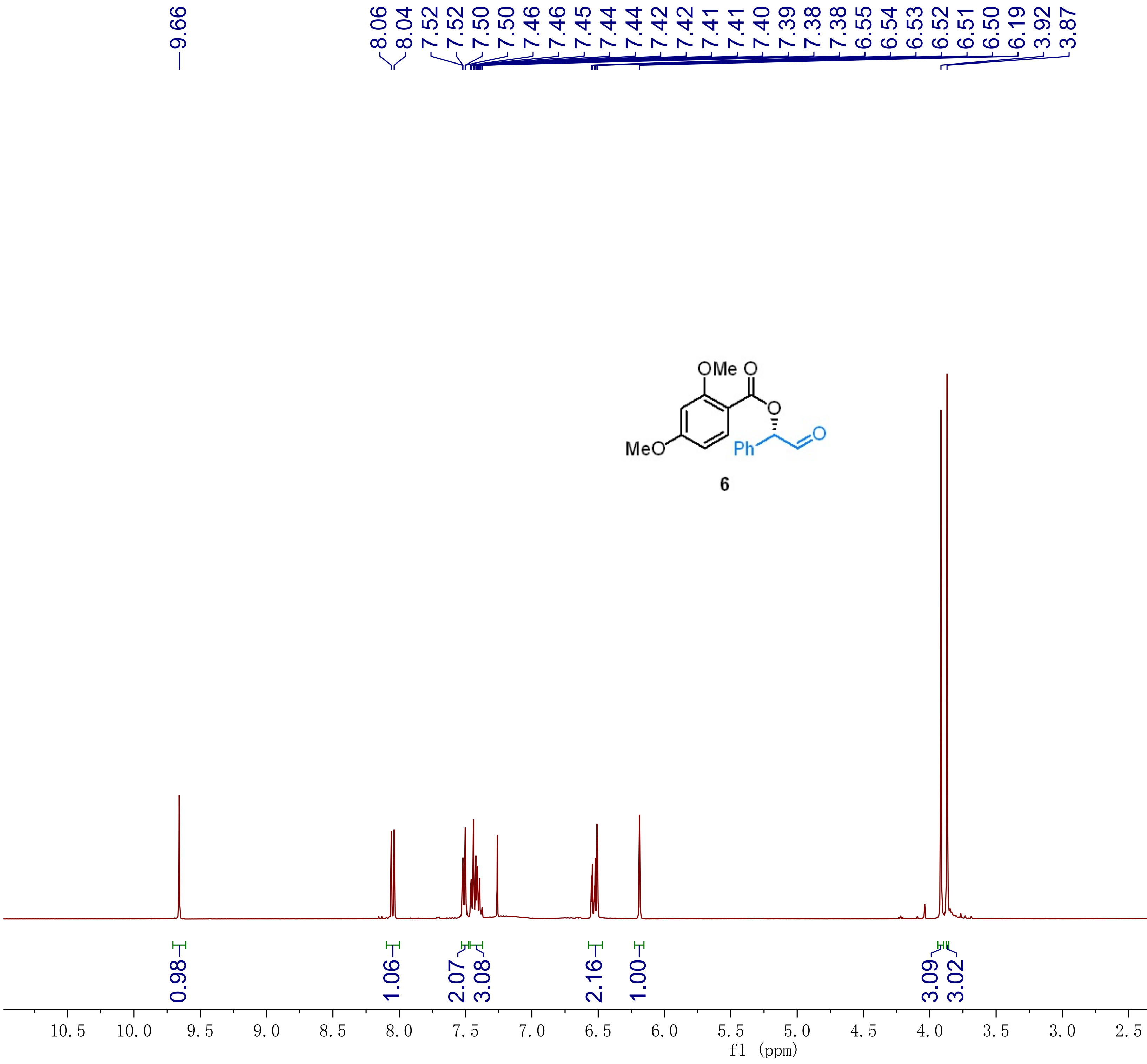

Parameter	Value
Title	qxk-118h-1.2.fid
Origin	Bruker BioSpin GmbH
Owner	nmr
Instrument	spect
Solvent	CDCl3
Temperature	292.6
Pulse Sequence	zg30
Experiment	1D
Number of Scans	16
Receiver Gain	287.0
Relaxation Delay	1.0000
Pulse Width	15.8000
Acquisition Time	3.9846
Acquisition Date	2025-02-15T18:01:40
Modification Date	2025-02-15T18:01:42
Spectrometer Frequency	400.13
Spectral Width	8223.7
Lowest Frequency	-1649.5
Nucleus	1H
Acquired Size	32768
Spectral Size	65536

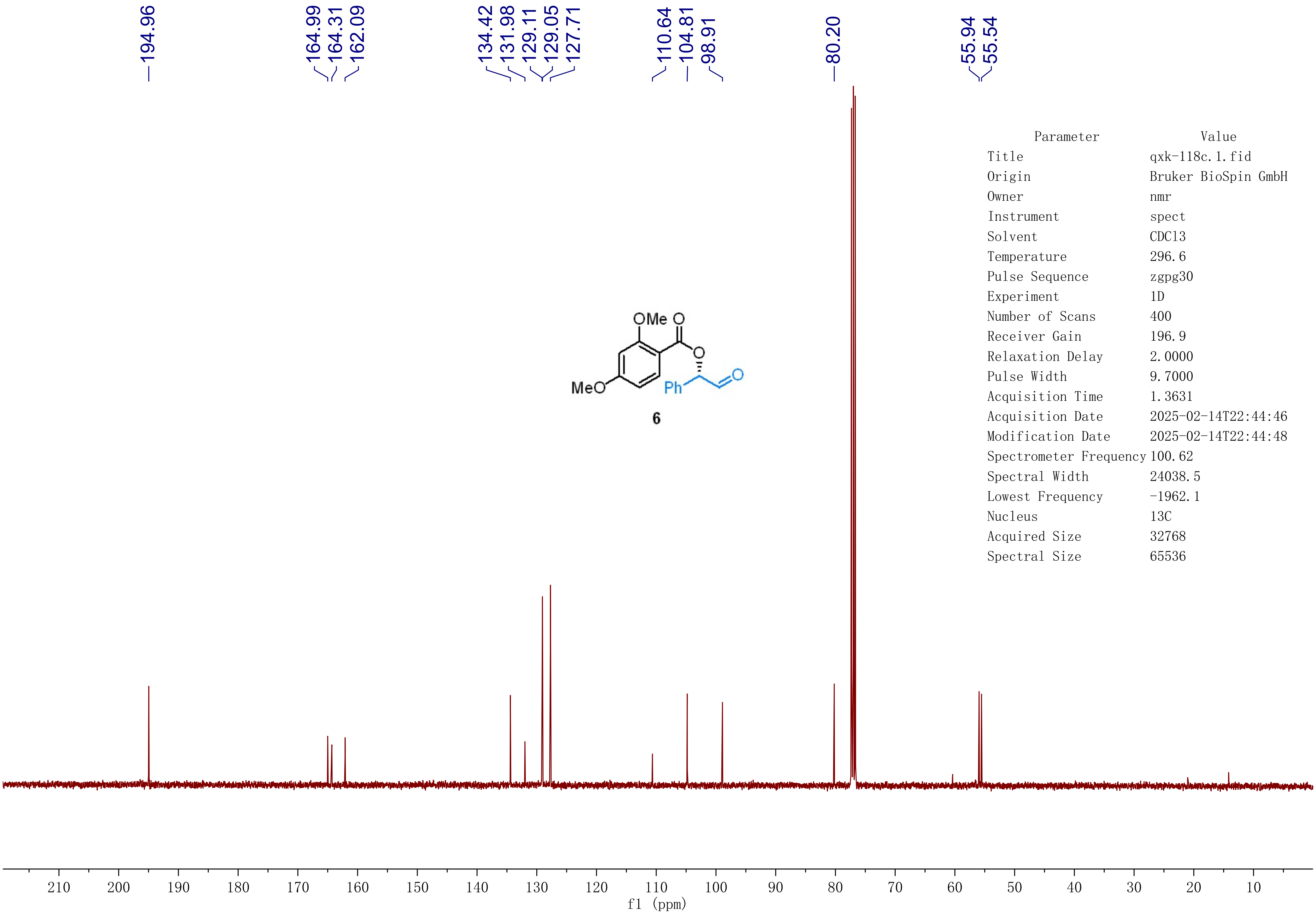

194.96
164.99
164.31
162.09
134.42
131.98
129.11
129.05
127.71
110.64
104.81
98.91
80.20
55.94
55.54
OMe O
O
MeO
Ph
O
6
Parameter Value
Title qxk-118c.1.fid
Origin Bruker BioSpin GmbH
Owner nmr
Instrument spect
Solvent CDCl3
Temperature 296.6
Pulse Sequence zgpg30
Experiment 1D
Number of Scans 400
Receiver Gain 196.9
Relaxation Delay 2.0000
Pulse Width 9.7000
Acquisition Time 1.3631
Acquisition Date 2025-02-14T22:44:46
Modification Date 2025-02-14T22:44:48
Spectrometer Frequency 100.62
Spectral Width 24038.5
Lowest Frequency -1962.1
Nucleus 13C
Acquired Size 32768
Spectral Size 65536
210 200 190 180 170 160 150 140 130 120 110 100 90 80 70 60 50 40 30 20 10
f1 (ppm)

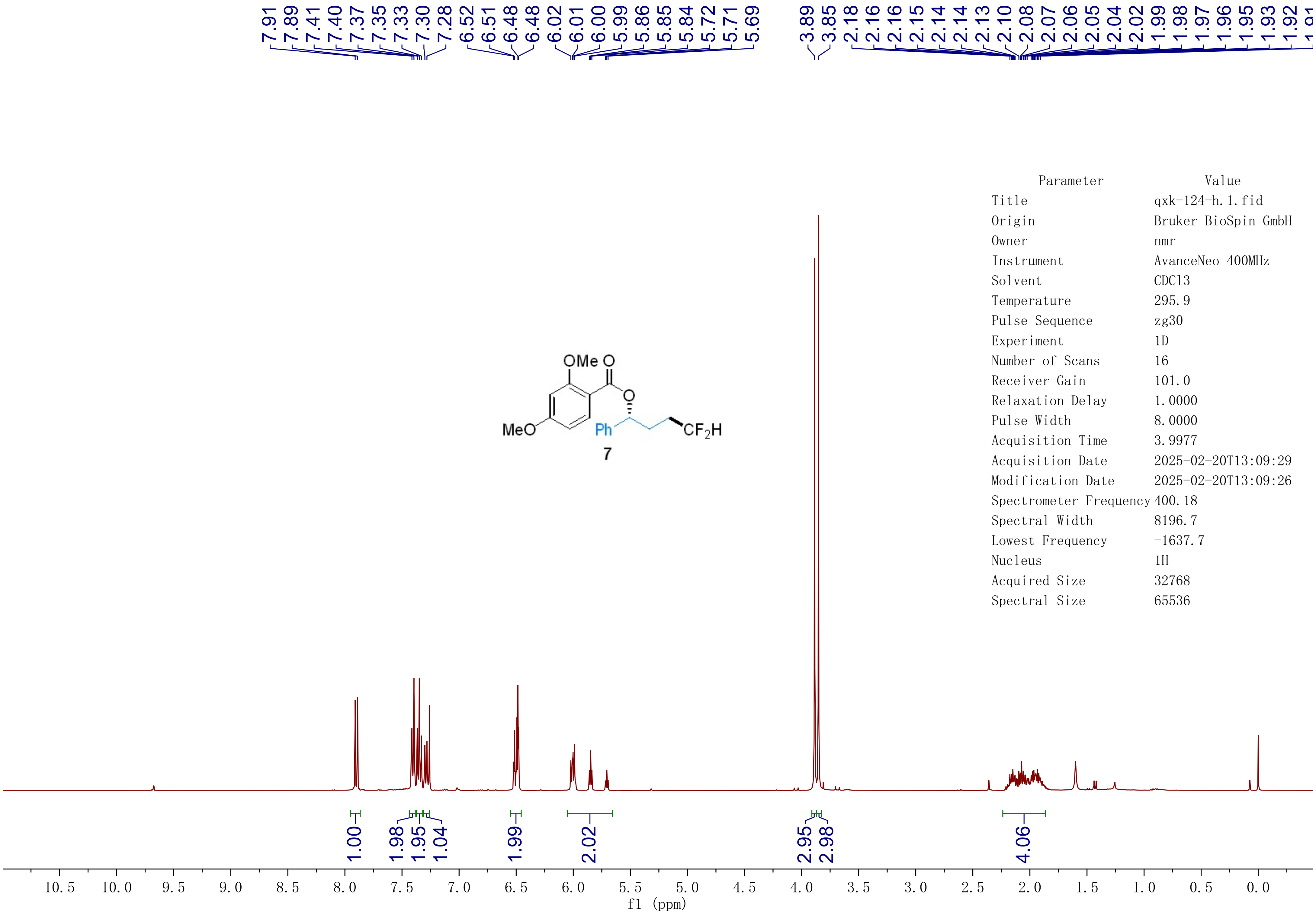

Parameter	Value
Title	qxk-124-h.1.fid
Origin	Bruker BioSpin GmbH
Owner	nmr
Instrument	AvanceNeo 400MHz
Solvent	CDCl3
Temperature	295.9
Pulse Sequence	zg30
Experiment	1D
Number of Scans	16
Receiver Gain	101.0
Relaxation Delay	1.0000
Pulse Width	8.0000
Acquisition Time	3.9977
Acquisition Date	2025-02-20T13:09:29
Modification Date	2025-02-20T13:09:26
Spectrometer Frequency	400.18
Spectral Width	8196.7
Lowest Frequency	-1637.7
Nucleus	1H
Acquired Size	32768
Spectral Size	65536

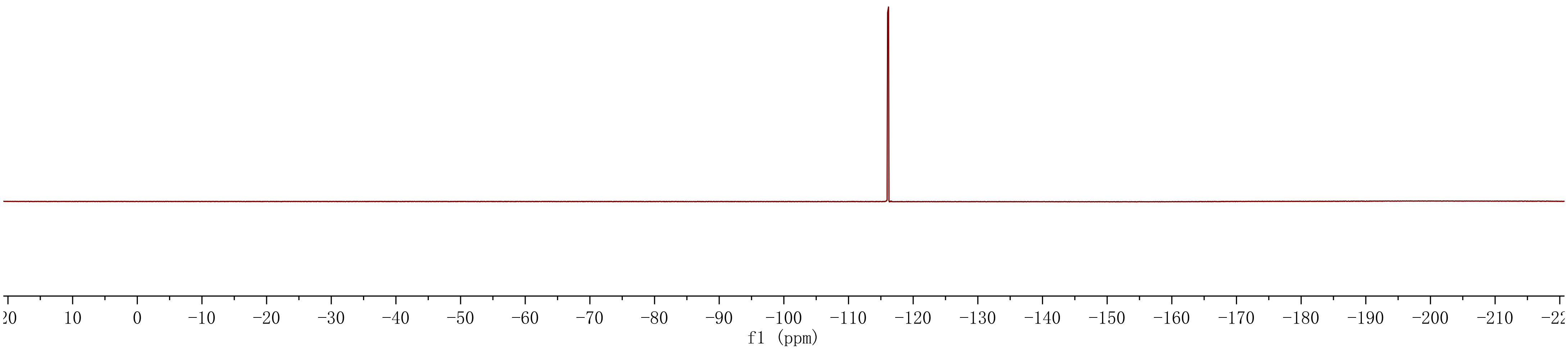

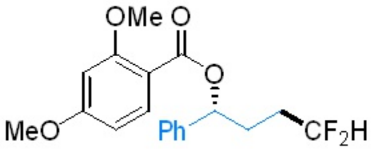

7

Parameter	Value
Title	qxk-124-f.3.fid
Origin	Bruker BioSpin GmbH
Owner	nmr
Instrument	AvanceNeo 400MHz
Solvent	CDCl3
Temperature	295.9
Pulse Sequence	zg
Experiment	1D
Number of Scans	16
Receiver Gain	101.0
Relaxation Delay	1.0000
Pulse Width	12.0000
Acquisition Time	0.7209
Acquisition Date	2025-02-20T13:11:31
Modification Date	2025-02-20T13:11:30
Spectrometer Frequency	376.51
Spectral Width	90909.1
Lowest Frequency	-83109.1
Nucleus	19F
Acquired Size	65536
Spectral Size	131072

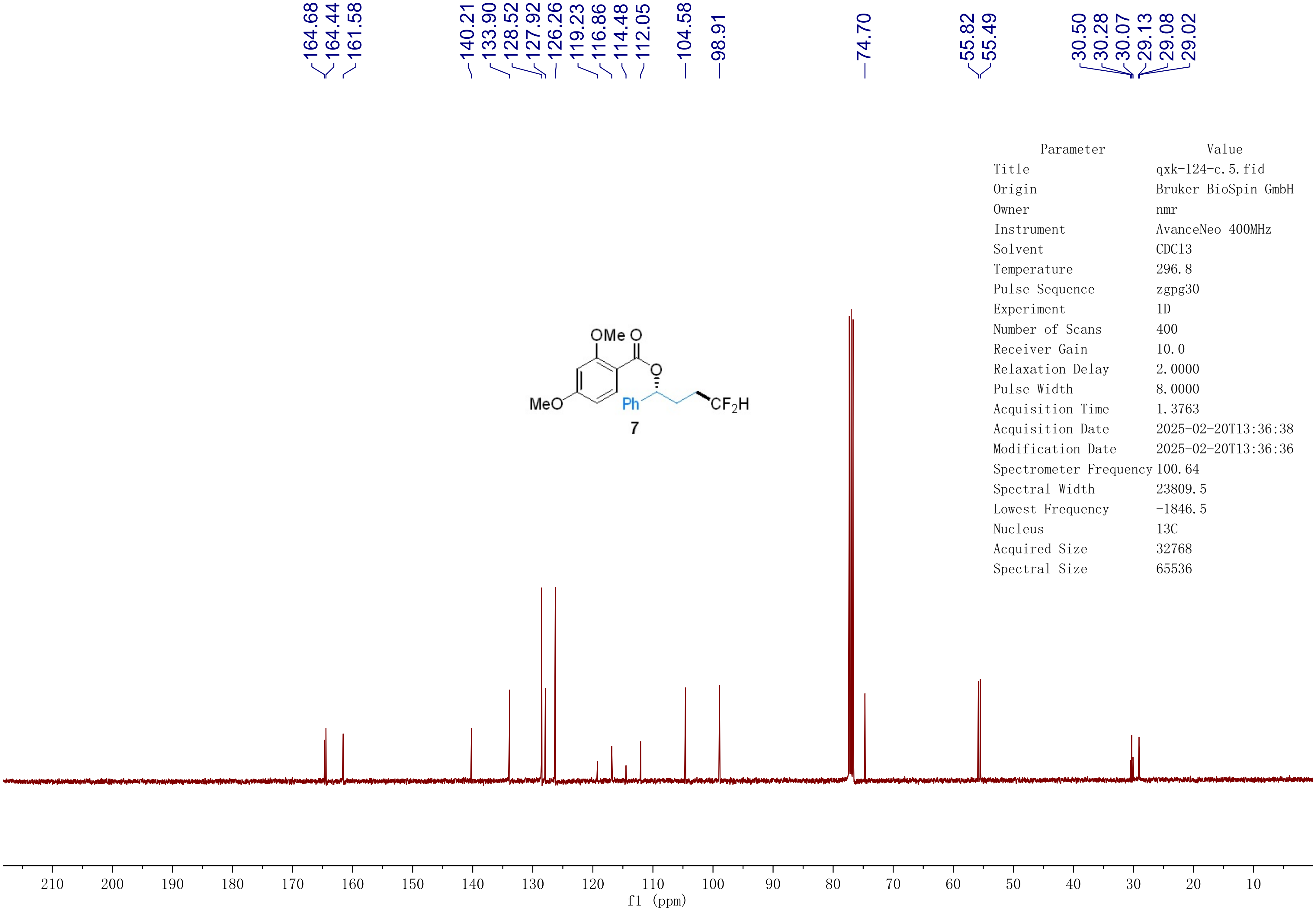

Parameter	Value
Title	qxk-124-c.5.fid
Origin	Bruker BioSpin GmbH
Owner	nmr
Instrument	AvanceNeo 400MHz
Solvent	CDCl3
Temperature	296.8
Pulse Sequence	zgpg30
Experiment	1D
Number of Scans	400
Receiver Gain	10.0
Relaxation Delay	2.0000
Pulse Width	8.0000
Acquisition Time	1.3763
Acquisition Date	2025-02-20T13:36:38
Modification Date	2025-02-20T13:36:36
Spectrometer Frequency	100.64
Spectral Width	23809.5
Lowest Frequency	-1846.5
Nucleus	13C
Acquired Size	32768
Spectral Size	65536

Parameter	Value
Title	qxk-210-2h.1.fid
Origin	Bruker BioSpin GmbH
Owner	nmr
Instrument	spect
Solvent	CDCl3
Temperature	292.2
Pulse Sequence	zg30
Experiment	1D
Number of Scans	16
Receiver Gain	228.0
Relaxation Delay	1.0000
Pulse Width	15.8000
Acquisition Time	3.9846
Acquisition Date	2025-05-18T13:24:14
Modification Date	2025-05-18T13:24:14
Spectrometer Frequency	400.13
Spectral Width	8223.7
Lowest Frequency	-1649.5
Nucleus	1H
Acquired Size	32768
Spectral Size	65536

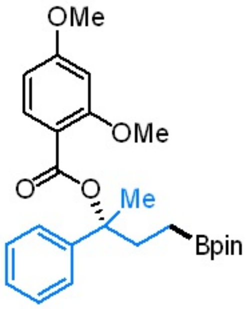

8

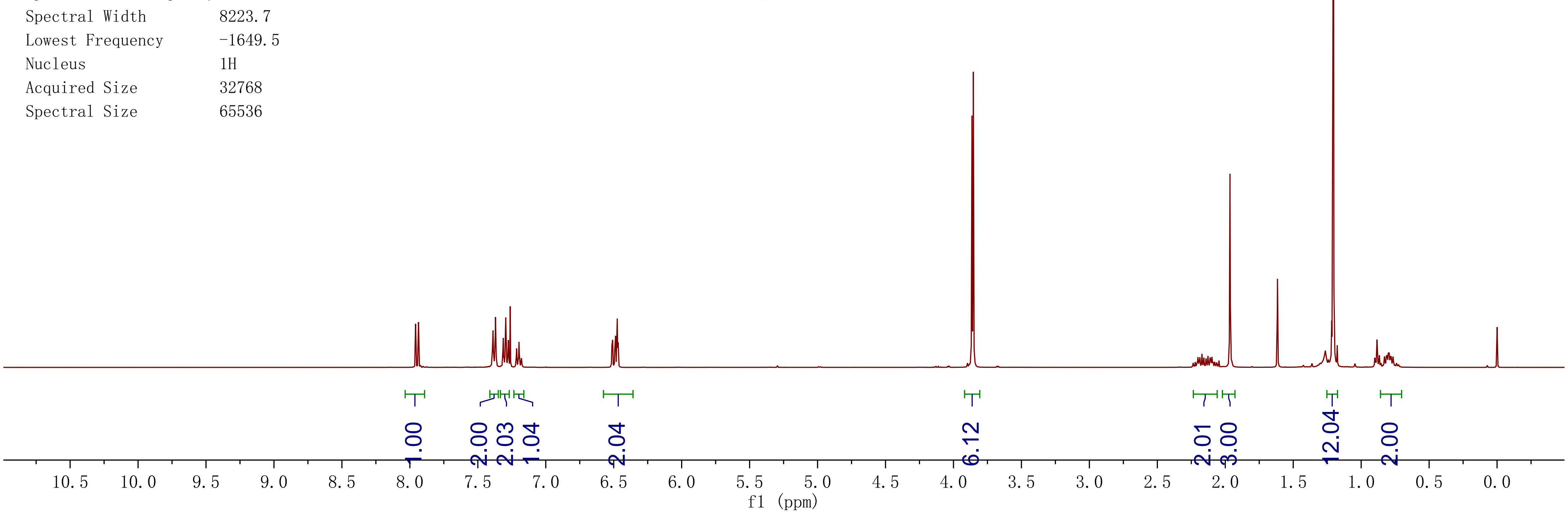

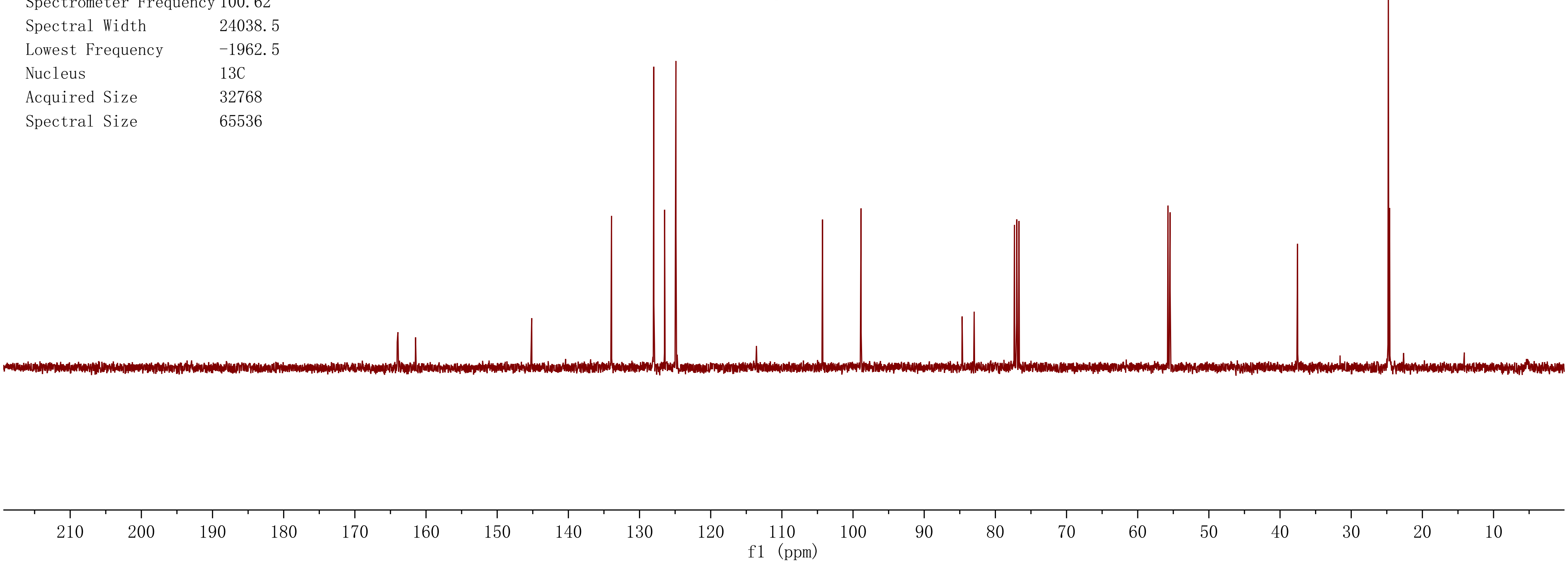

Parameter	Value
Title	qxk-210-2c.1.fid
Origin	Bruker BioSpin GmbH
Owner	nmr
Instrument	spect
Solvent	CDCl3
Temperature	292.8
Pulse Sequence	zgpg30
Experiment	1D
Number of Scans	368
Receiver Gain	22.6
Relaxation Delay	2.0000
Pulse Width	40.0000
Acquisition Time	1.3631
Acquisition Date	2025-05-18T13:51:02
Modification Date	2025-05-18T13:51:04
Spectrometer Frequency	100.62
Spectral Width	24038.5
Lowest Frequency	-1962.5
Nucleus	13C
Acquired Size	32768
Spectral Size	65536

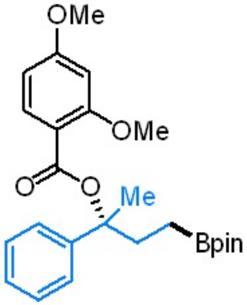

8

Parameter	Value
Title	qxk-189--a-3h.1.fid
Origin	Bruker BioSpin GmbH
Owner	nmr
Instrument	spect
Solvent	CDCl3
Temperature	293.0
Pulse Sequence	zg30
Experiment	1D
Number of Scans	16
Receiver Gain	114.0
Relaxation Delay	1.0000
Pulse Width	15.8000
Acquisition Time	3.9846
Acquisition Date	2025-05-12T22:26:58
Modification Date	2025-05-12T22:27:00
Spectrometer Frequency	400.13
Spectral Width	8223.7
Lowest Frequency	-1646.3
Nucleus	1H
Acquired Size	32768
Spectral Size	65536

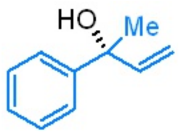

9

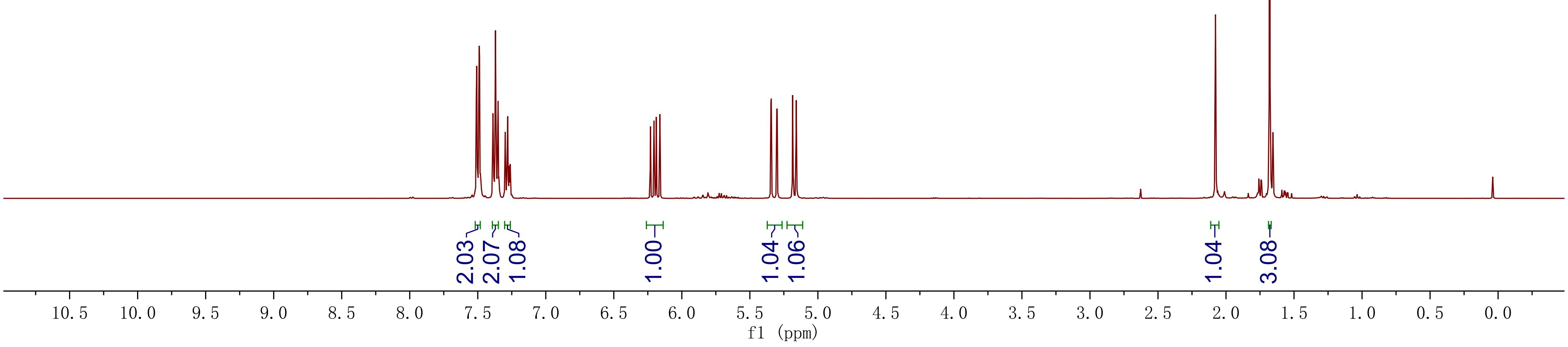

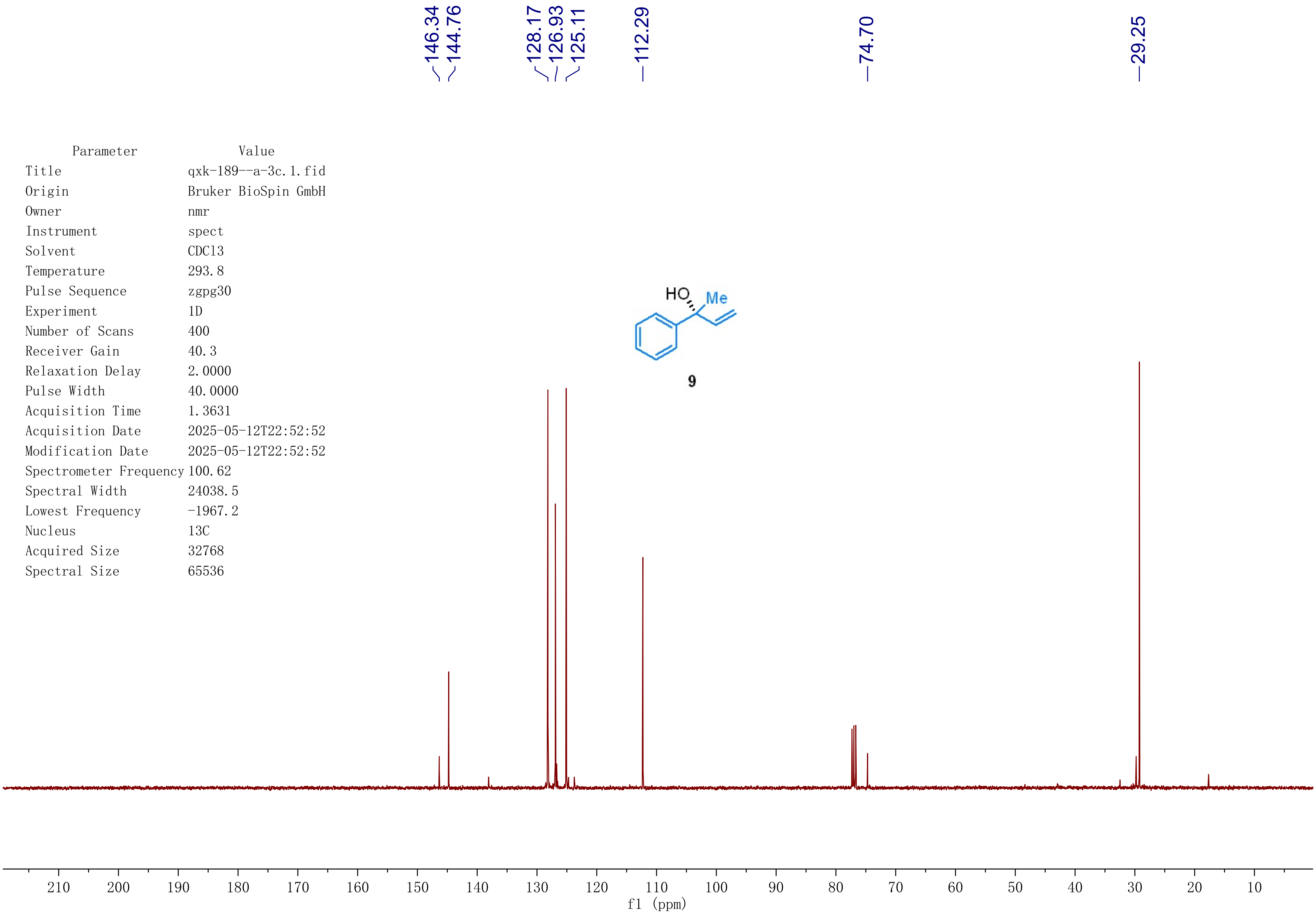

Parameter	Value
Title	qxk-189--a-3c.1.fid
Origin	Bruker BioSpin GmbH
Owner	nmr
Instrument	spect
Solvent	CDCl3
Temperature	293.8
Pulse Sequence	zgpg30
Experiment	1D
Number of Scans	400
Receiver Gain	40.3
Relaxation Delay	2.0000
Pulse Width	40.0000
Acquisition Time	1.3631
Acquisition Date	2025-05-12T22:52:52
Modification Date	2025-05-12T22:52:52
Spectrometer Frequency	100.62
Spectral Width	24038.5
Lowest Frequency	-1967.2
Nucleus	13C
Acquired Size	32768
Spectral Size	65536

Parameter	Value
Title	qxk-200.5.fid
Origin	Bruker BioSpin GmbH
Owner	nmr
Instrument	AvanceNeo 400MHz
Solvent	CDCl3
Temperature	298.3
Pulse Sequence	zg30
Experiment	1D
Number of Scans	16
Receiver Gain	101.0
Relaxation Delay	1.0000
Pulse Width	8.0000
Acquisition Time	3.9977
Acquisition Date	2025-05-15T01:04:38
Modification Date	2025-05-15T01:04:32
Spectrometer Frequency	400.18
Spectral Width	8196.7
Lowest Frequency	-1637.7
Nucleus	1H
Acquired Size	32768
Spectral Size	65536

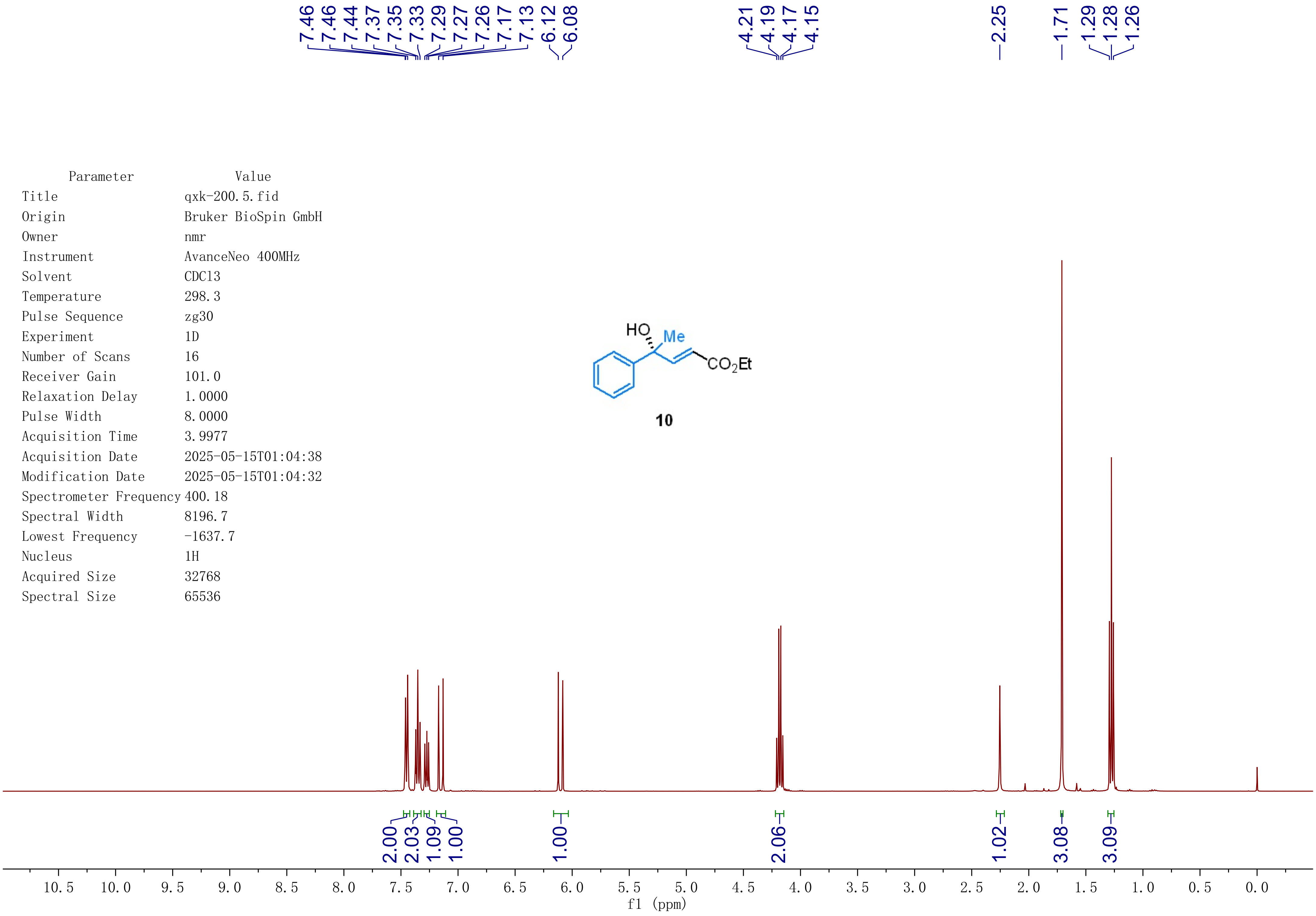

Parameter	Value
Title	qxk-200.6.fid
Origin	Bruker BioSpin GmbH
Owner	nmr
Instrument	AvanceNeo 400MHz
Solvent	CDCl3
Temperature	299.0
Pulse Sequence	zgpg30
Experiment	1D
Number of Scans	400
Receiver Gain	16.0
Relaxation Delay	2.0000
Pulse Width	8.0000
Acquisition Time	1.3763
Acquisition Date	2025-05-15T01:29:04
Modification Date	2025-05-15T01:28:58
Spectrometer Frequency	100.64
Spectral Width	23809.5
Lowest Frequency	-1847.1
Nucleus	13C
Acquired Size	32768
Spectral Size	65536

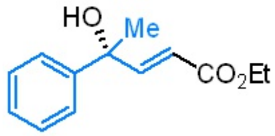

10

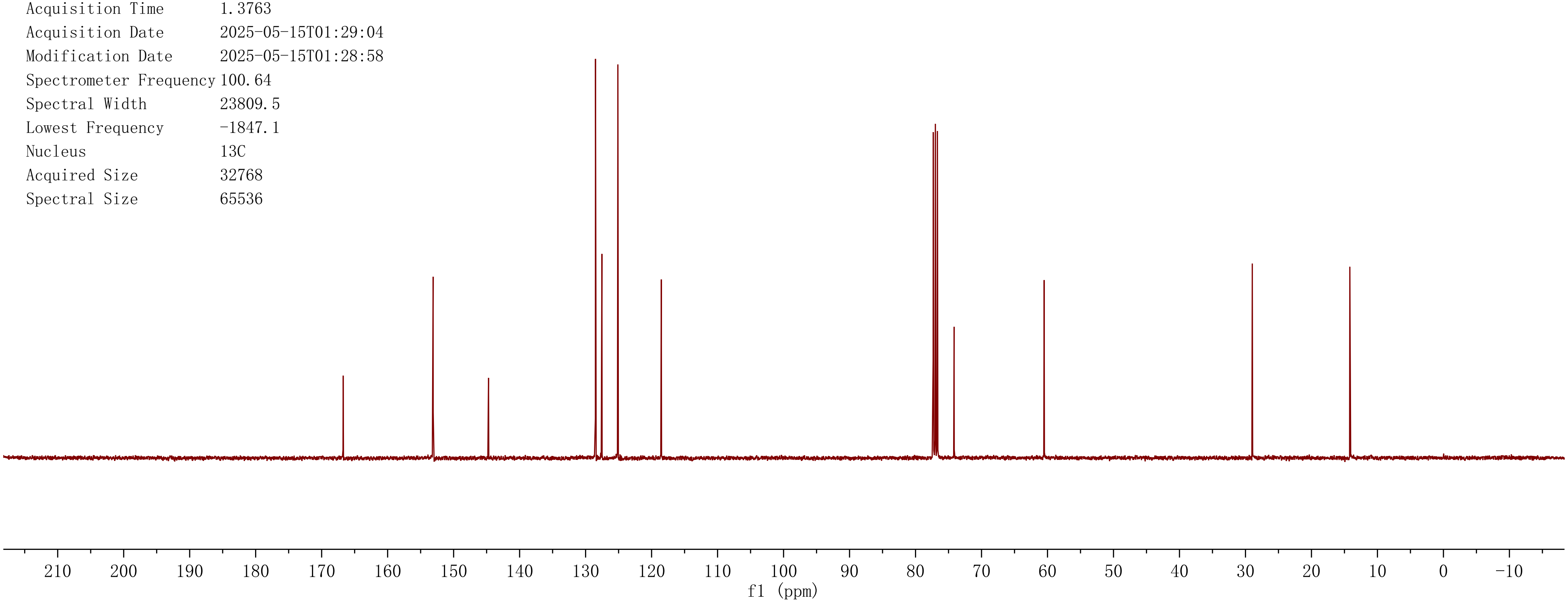

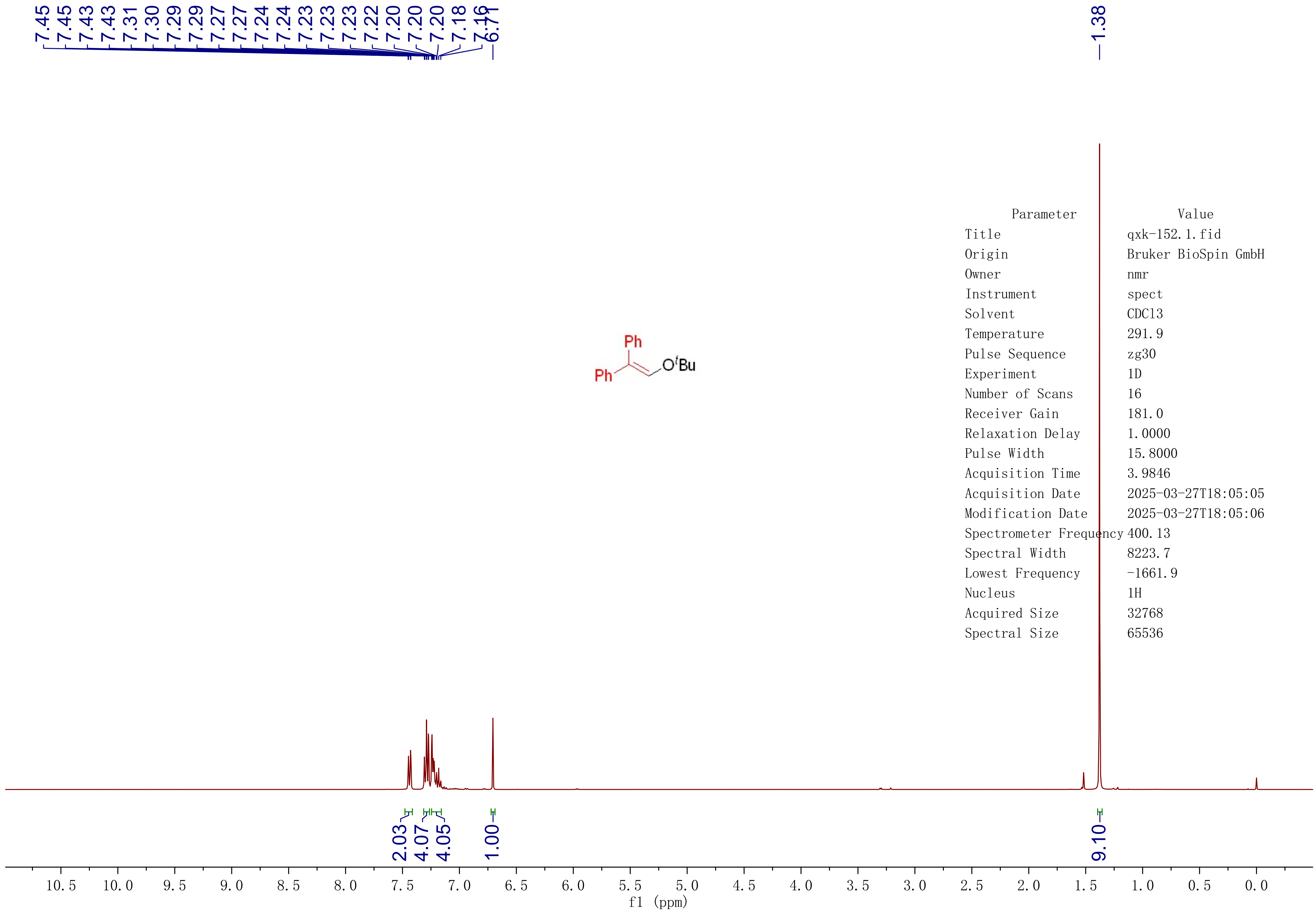

Parameter	Value
Title	qxk-152.1.fid
Origin	Bruker BioSpin GmbH
Owner	nmr
Instrument	spect
Solvent	CDCl3
Temperature	291.9
Pulse Sequence	zg30
Experiment	1D
Number of Scans	16
Receiver Gain	181.0
Relaxation Delay	1.0000
Pulse Width	15.8000
Acquisition Time	3.9846
Acquisition Date	2025-03-27T18:05:05
Modification Date	2025-03-27T18:05:06
Spectrometer Frequency	400.13
Spectral Width	8223.7
Lowest Frequency	-1661.9
Nucleus	1H
Acquired Size	32768
Spectral Size	65536

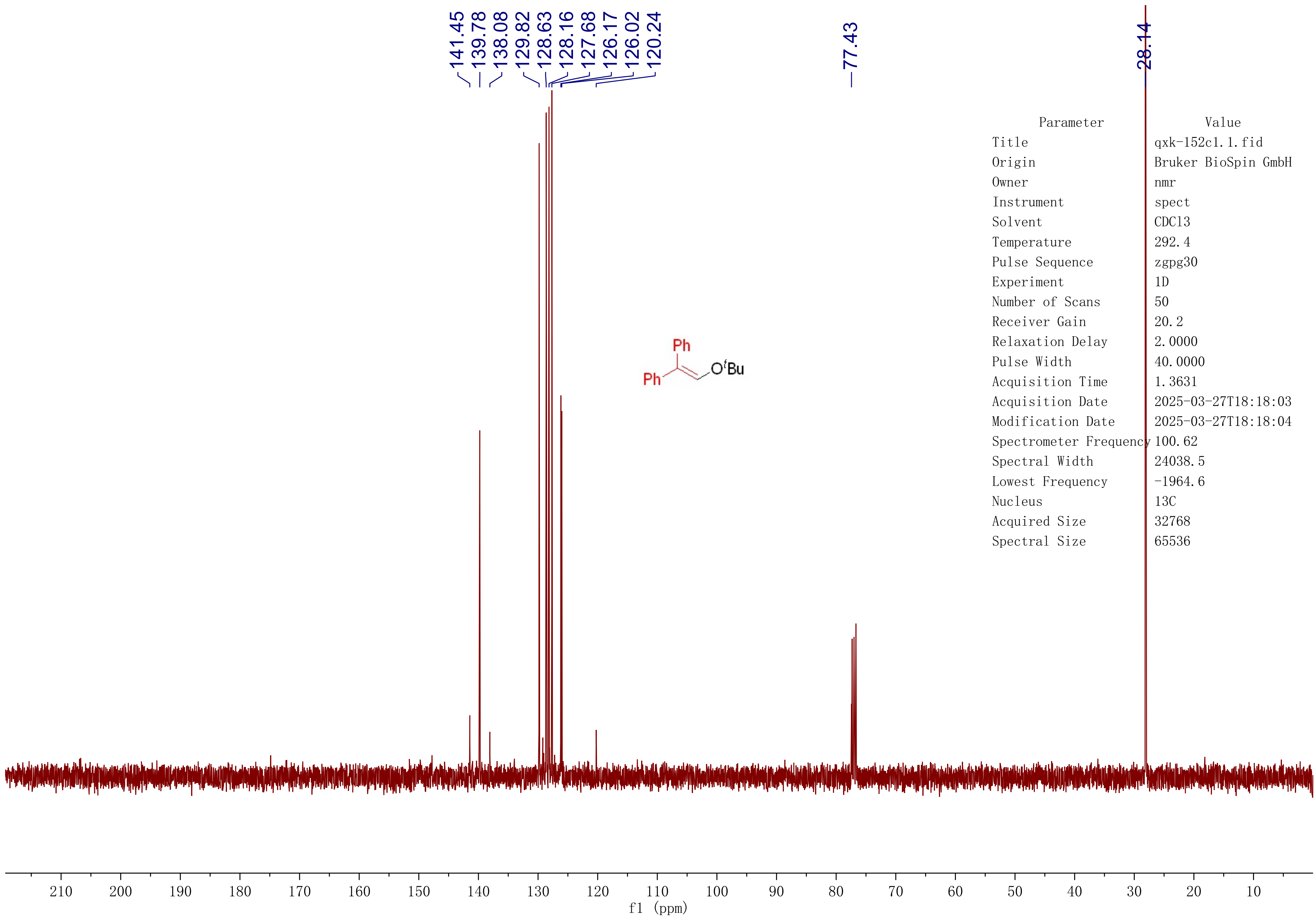

Parameter	Value
Title	qxk-152c1.1.fid
Origin	Bruker BioSpin GmbH
Owner	nmr
Instrument	spect
Solvent	CDCl3
Temperature	292.4
Pulse Sequence	zgpg30
Experiment	1D
Number of Scans	50
Receiver Gain	20.2
Relaxation Delay	2.0000
Pulse Width	40.0000
Acquisition Time	1.3631
Acquisition Date	2025-03-27T18:18:03
Modification Date	2025-03-27T18:18:04
Spectrometer Frequency	100.62
Spectral Width	24038.5
Lowest Frequency	-1964.6
Nucleus	13C
Acquired Size	32768
Spectral Size	65536

===

Acq. Operator : SYSTEM Seq. Line : 12
Sample Operator : SYSTEM
Acq. Instrument : HPLC Location : P1-F-07
Injection Date : 14/1/2025 7:45:23 pm Inj : 1
Inj Volume : 2.000 µl
Acq. Method : C:\Users\Public\Documents\ChemStation\1\Data\SUN\SUN 2025-01-14 16-10-18\AD3-20-20.M
Last changed : 20/12/2024 6:43:38 pm by SYSTEM
Analysis Method : C:\Users\Public\Documents\ChemStation\1\Data\SUN\SUN 2025-01-14 16-10-18\AD3-20-20.M (Sequence Method)
Last changed : 17/1/2025 8:50:21 pm by SYSTEM
(modified after loading)
Additional Info : Peak(s) manually integrated

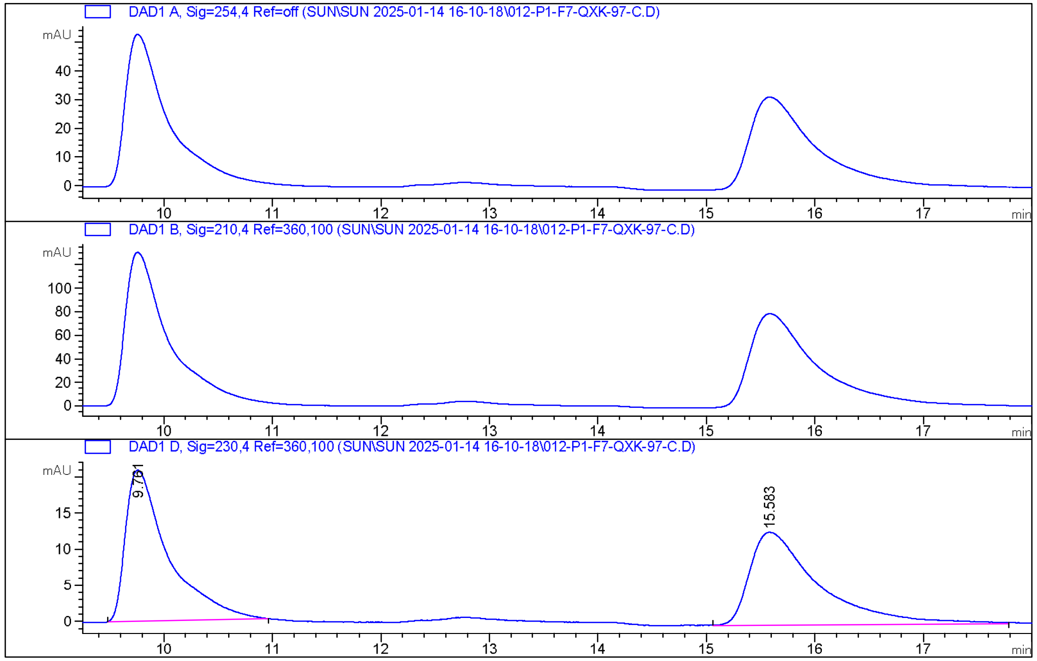

===

Area Percent Report

===

Sorted By : Signal
Multiplier : 1.0000
Dilution : 1.0000
Use Multiplier & Dilution Factor with ISTDs

Data File C:\Users\P...ChemStation\1\Data\SUN\SUN 2025-01-14 16-10-18\012-P1-F7-QXK-97-C.D
Sample Name: QXK-97-C

Signal 1: DAD1 A, Sig=254,4 Ref=off

Signal 2: DAD1 B, Sig=210,4 Ref=360,100

Signal 3: DAD1 D, Sig=230,4 Ref=360,100

Peak #	RetTime [min]	Type	Width [min]	Area [mAU*s]	Height [mAU]	Area %
1	9.761	MM R	0.4549	572.38165	20.97090	49.9569
2	15.583	MM R	0.7396	573.36993	12.92153	50.0431

Totals : 1145.75159 33.89243

===

*** End of Report ***

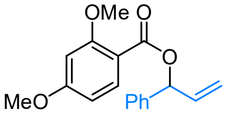

3a-Rac

===

Acq. Operator : SYSTEM Seq. Line : 2
Sample Operator : SYSTEM
Acq. Instrument : HPLC Location : P1-F-01
Injection Date : 1/1/2025 10:40:22 am Inj : 1
Inj Volume : 2.000 µl
Different Inj Volume from Sample Entry! Actual Inj Volume : 20.000 µl
Acq. Method : C:\Users\Public\Documents\ChemStation\1\Data\SUN\SUN 2025-01-01 10-27-09\AD3-20-20.M
Last changed : 20/12/2024 6:43:38 pm by SYSTEM
Analysis Method : C:\Users\Public\Documents\ChemStation\1\Data\SUN\SUN 2025-01-01 10-27-09\AD3-20-20.M (Sequence Method)
Last changed : 2/1/2025 10:42:32 am by SYSTEM
(modified after loading)
Additional Info : Peak(s) manually integrated

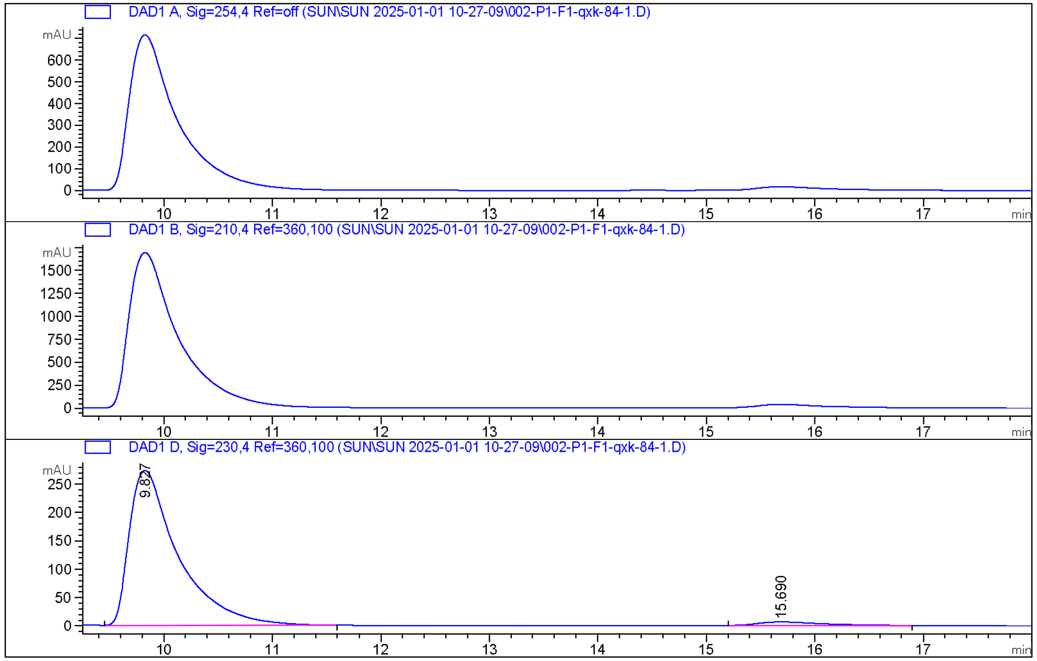

===
Area Percent Report
===

Sorted By : Signal
Multiplier : 1.0000
Dilution : 1.0000
Use Multiplier & Dilution Factor with ISTDs

Data File C:\Users\P...ChemStation\1\Data\SUN\SUN 2025-01-01 10-27-09\002-P1-F1-qxk-84-1.D
Sample Name: qxk-84-1

Signal 1: DAD1 A, Sig=254,4 Ref=off

Signal 2: DAD1 B, Sig=210,4 Ref=360,100

Signal 3: DAD1 D, Sig=230,4 Ref=360,100

Peak #	RetTime [min]	Type	Width [min]	Area [mAU*s]	Height [mAU]	Area %
1	9.827	BB	0.4519	8541.63770	273.43384	97.1949
2	15.690	BB	0.4804	246.51921	6.02395	2.8051

Totals : 8788.15691 279.45779

===
*** End of Report ***

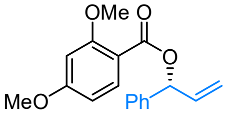

3a

===

Acq. Operator : SYSTEM Seq. Line : 4
Sample Operator : SYSTEM
Acq. Instrument : HPLC Location : P1-F-02
Injection Date : 19/11/2024 11:06:35 pm Inj : 1
Inj Volume : 2.000 µl
Different Inj Volume from Sample Entry! Actual Inj Volume : 20.000 µl
Acq. Method : C:\Users\Public\Documents\ChemStation\1\Data\SUN\SUN 2024-11-19 22-00-37\AD3-20-40.M
Last changed : 29/12/2022 10:50:52 pm by SYSTEM
Analysis Method : C:\Users\Public\Documents\ChemStation\1\Data\SUN\SUN 2024-11-19 22-00-37\AD3-20-40.M (Sequence Method)
Last changed : 2/1/2025 11:13:38 am by SYSTEM
(modified after loading)
Additional Info : Peak(s) manually integrated

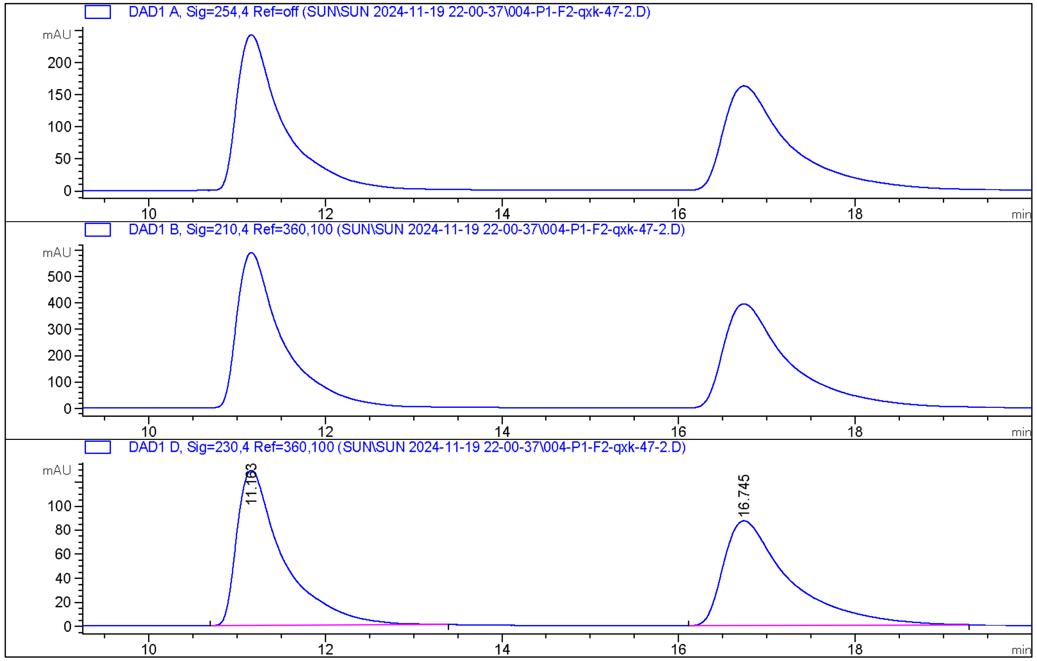

===

Area Percent Report

===

Sorted By : Signal
Multiplier : 1.0000
Dilution : 1.0000
Use Multiplier & Dilution Factor with ISTDs

Data File C:\Users\P...ChemStation\1\Data\SUN\SUN 2024-11-19 22-00-37\004-P1-F2-qxk-47-2.D
Sample Name: qxk-47-2

Signal 1: DAD1 A, Sig=254,4 Ref=off

Signal 2: DAD1 B, Sig=210,4 Ref=360,100

Signal 3: DAD1 D, Sig=230,4 Ref=360,100

Peak #	RetTime [min]	Type	Width [min]	Area [mAU*s]	Height [mAU]	Area %
1	11.163	BB	0.5132	4703.85547	128.92268	51.0582
2	16.745	BB	0.6122	4508.88574	86.99048	48.9418
Totals :				9212.74121	215.91316	

===

*** End of Report ***

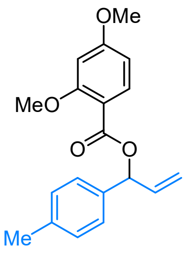

3b-Rac

Data File C:\Users\P...ChemStation\1\Data\SUN\SUN 2025-01-01 10-27-09\003-P1-F2-qxk-84-2.D
Sample Name: qxk-84-2

===

Acq. Operator : SYSTEM Seq. Line : 3
Sample Operator : SYSTEM
Acq. Instrument : HPLC Location : P1-F-02
Injection Date : 1/1/2025 11:01:32 am Inj : 1
Inj Volume : 2.000 µl
Different Inj Volume from Sample Entry! Actual Inj Volume : 20.000 µl
Acq. Method : C:\Users\Public\Documents\ChemStation\1\Data\SUN\SUN 2025-01-01 10-27-09\AD3-20-20.M
Last changed : 20/12/2024 6:43:38 pm by SYSTEM
Analysis Method : C:\Users\Public\Documents\ChemStation\1\Data\SUN\SUN 2025-01-01 10-27-09\AD3-20-20.M (Sequence Method)
Last changed : 2/1/2025 11:18:19 am by SYSTEM
(modified after loading)
Additional Info : Peak(s) manually integrated

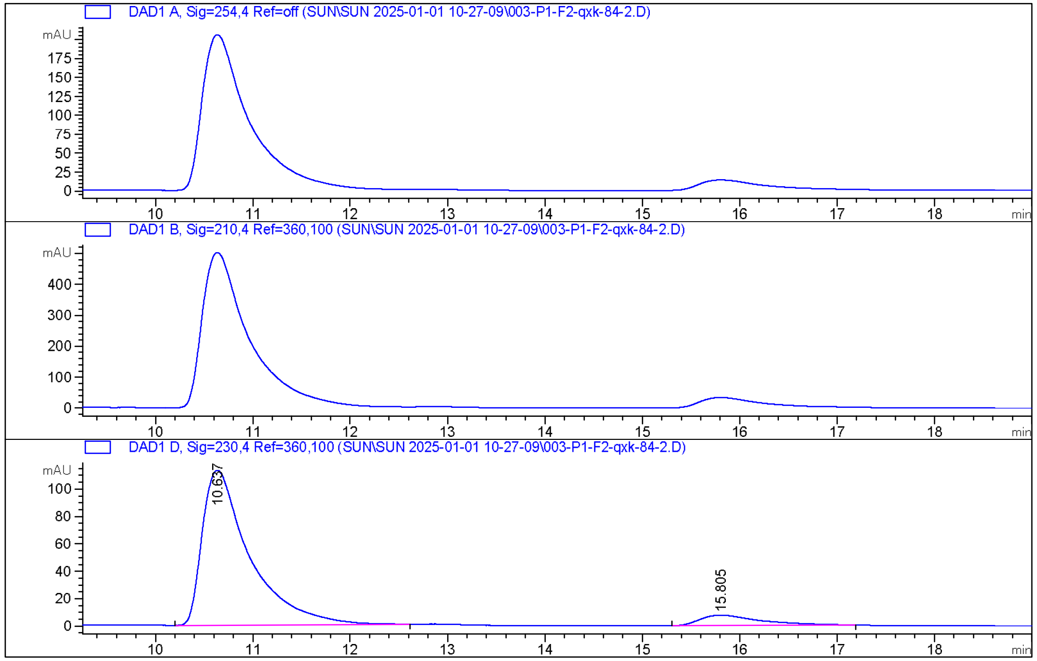

===

Area Percent Report

===

Sorted By : Signal
Multiplier : 1.0000
Dilution : 1.0000
Use Multiplier & Dilution Factor with ISTDs

Data File C:\Users\P...ChemStation\1\Data\SUN\SUN 2025-01-01 10-27-09\003-P1-F2-qxk-84-2.D
Sample Name: qxk-84-2

Signal 1: DAD1 A, Sig=254,4 Ref=off

Signal 2: DAD1 B, Sig=210,4 Ref=360,100

Signal 3: DAD1 D, Sig=230,4 Ref=360,100

Peak #	RetTime [min]	Type	Width [min]	Area [mAU*s]	Height [mAU]	Area %
1	10.637	BB	0.4778	3833.70605	113.14655	92.0499
2	15.805	BB	0.5093	331.10605	7.62971	7.9501

Totals : 4164.81210 120.77626

===

*** End of Report ***

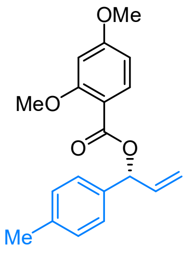

3b

===

```
Acq. Operator    : SYSTEM                      Seq. Line :    2
Sample Operator  : SYSTEM
Acq. Instrument  : HPLC                         Location :    P1-F-01
Injection Date   : 19/11/2024 10:13:56 pm            Inj :    1
                                              Inj Volume : 2.000 µl
Different Inj Volume from Sample Entry!  Actual Inj Volume : 20.000 µl
Acq. Method      : C:\Users\Public\Documents\ChemStation\1\Data\SUN\SUN 2024-11-19 22-00-37\AD3-
                   20-40.M
Last changed     : 29/12/2022 10:50:52 pm by SYSTEM
Analysis Method  : C:\Users\Public\Documents\ChemStation\1\Data\SUN\SUN 2024-11-19 22-00-37\AD3-
                   20-40.M (Sequence Method)
Last changed     : 2/1/2025 11:13:38 am by SYSTEM
                   (modified after loading)
Additional Info  : Peak(s) manually integrated
```

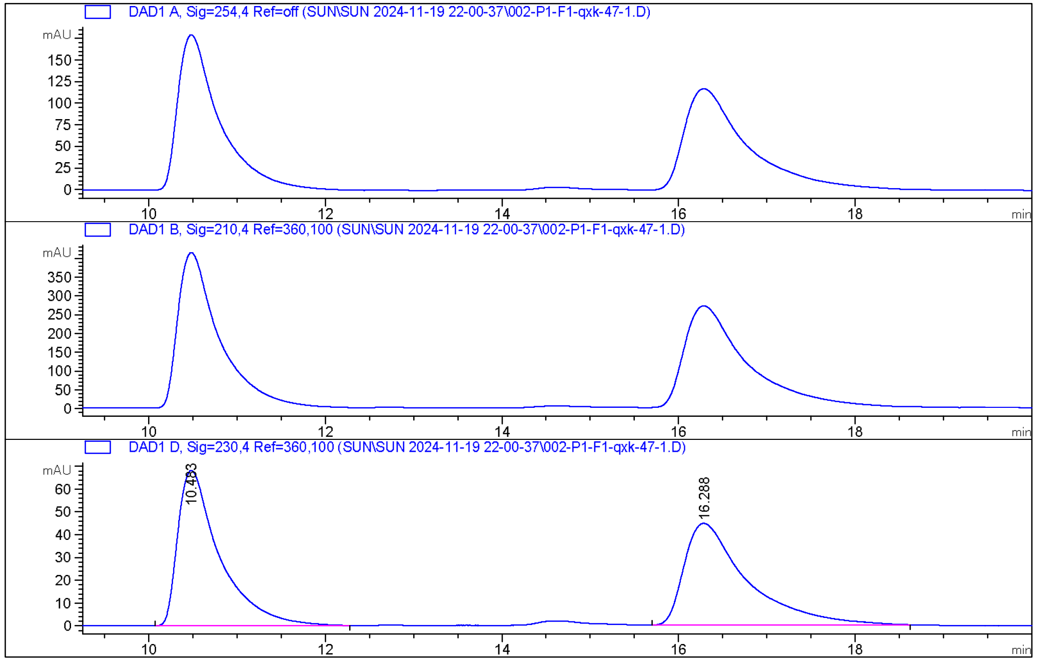

===
Area Percent Report
===

```
Sorted By             :      Signal
Multiplier            :      1.0000
Dilution              :      1.0000
Use Multiplier & Dilution Factor with ISTDs
```


Data File C:\Users\P...ChemStation\1\Data\SUN\SUN 2024-11-19 22-00-37\002-P1-F1-qxk-47-1.D
Sample Name: qxk-47-1

Signal 1: DAD1 A, Sig=254,4 Ref=off

Signal 2: DAD1 B, Sig=210,4 Ref=360,100

Signal 3: DAD1 D, Sig=230,4 Ref=360,100

Peak #	RetTime [min]	Type	Width [min]	Area [mAU*s]	Height [mAU]	Area %
1	10.483	BB	0.4490	2242.16699	68.21859	50.5458
2	16.288	BB	0.5832	2193.74780	44.57017	49.4542

Totals : 4435.91479 112.78876

===
*** End of Report ***

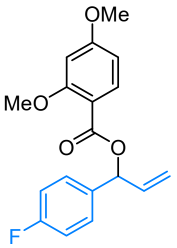

3c-Rac

Data File C:\Users\P...ChemStation\1\Data\SUN\SUN 2025-01-01 10-27-09\004-P1-F3-qxk-84-3.D
Sample Name: qxk-84-3

===

```
Acq. Operator   : SYSTEM                        Seq. Line :    4
Sample Operator : SYSTEM
Acq. Instrument : HPLC                           Location :    P1-F-03
Injection Date  : 1/1/2025 11:22:43 am                Inj :    1
                                               Inj Volume : 2.000 µl
Different Inj Volume from Sample Entry!  Actual Inj Volume : 20.000 µl
Acq. Method     : C:\Users\Public\Documents\ChemStation\1\Data\SUN\SUN 2025-01-01 10-27-09\AD3-
                  20-20.M
Last changed    : 20/12/2024 6:43:38 pm by SYSTEM
Analysis Method : C:\Users\Public\Documents\ChemStation\1\Data\SUN\SUN 2025-01-01 10-27-09\AD3-
                  20-20.M (Sequence Method)
Last changed    : 2/1/2025 4:58:14 pm by SYSTEM
                  (modified after loading)
Additional Info : Peak(s) manually integrated
```

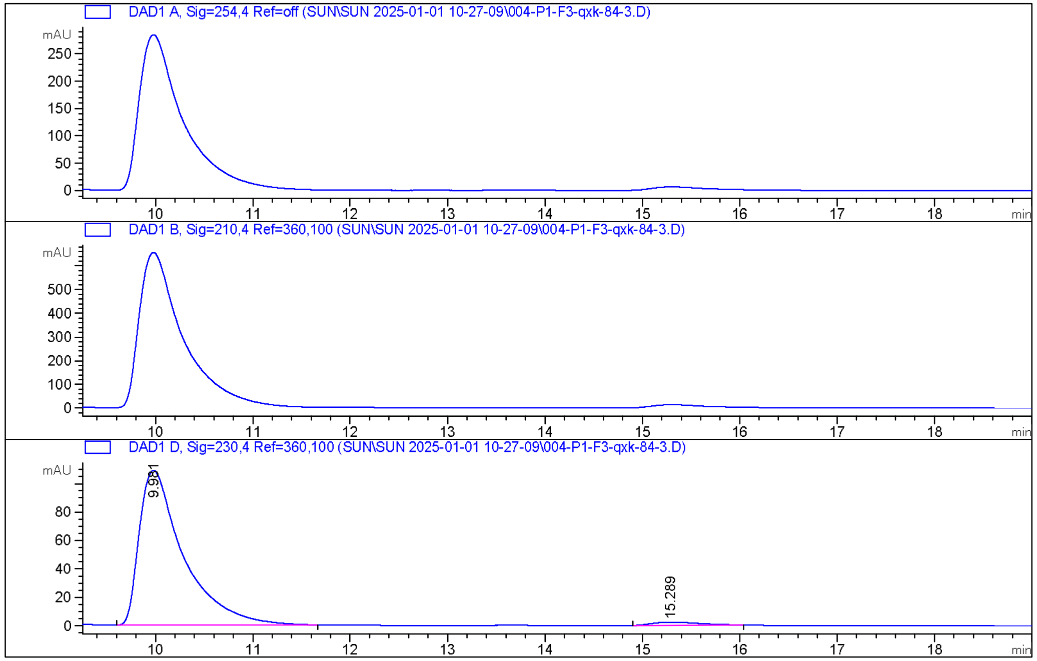

===

Area Percent Report

===

```
Sorted By             :      Signal
Multiplier            :      1.0000
Dilution              :      1.0000
Use Multiplier & Dilution Factor with ISTDs
```


Data File C:\Users\P...ChemStation\1\Data\SUN\SUN 2025-01-01 10-27-09\004-P1-F3-qxk-84-3.D
Sample Name: qxk-84-3

Signal 1: DAD1 A, Sig=254,4 Ref=off

Signal 2: DAD1 B, Sig=210,4 Ref=360,100

Signal 3: DAD1 D, Sig=230,4 Ref=360,100

Peak #	RetTime [min]	Type	Width [min]	Area [mAU*s]	Height [mAU]	Area %
1	9.981	BB	0.4485	3437.47241	109.12415	97.8840
2	15.289	BB	0.3940	74.30822	2.21979	2.1160

Totals : 3511.78063 111.34394

===

*** End of Report ***

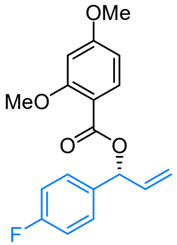

3c

Data File C:\Users\P...mStation\1\Data\SUN\SUN 2025-02-14 15-47-48\002-P1-F1-qxk-84-4rac.D
Sample Name: qxk-84-4rac

===

Acq. Operator : SYSTEM Seq. Line : 2
Sample Operator : SYSTEM
Acq. Instrument : HPLC Location : P1-F-01
Injection Date : 14/2/2025 4:01:24 pm Inj : 1
Inj Volume : 2.000 µl
Different Inj Volume from Sample Entry! Actual Inj Volume : 20.000 µl
Acq. Method : C:\Users\Public\Documents\ChemStation\1\Data\SUN\SUN 2025-02-14 15-47-48\AD3-20-20.M
Last changed : 20/12/2024 6:43:38 pm by SYSTEM
Analysis Method : C:\Users\Public\Documents\ChemStation\1\Data\SUN\SUN 2025-02-14 15-47-48\AD3-20-20.M (Sequence Method)
Last changed : 28/2/2025 4:08:07 pm by SYSTEM
(modified after loading)
Additional Info : Peak(s) manually integrated

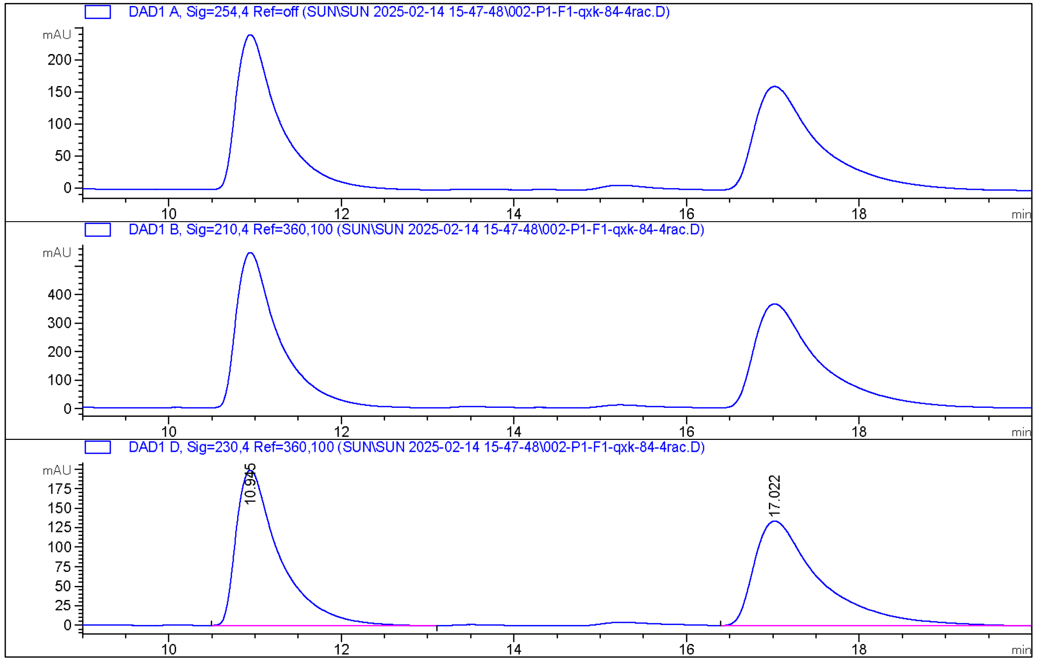

===

Area Percent Report

===

Sorted By : Signal
Multiplier : 1.0000
Dilution : 1.0000
Use Multiplier & Dilution Factor with ISTDs

Data File C:\Users\P...mStation\1\Data\SUN\SUN 2025-02-14 15-47-48\002-P1-F1-qxk-84-4rac.D
Sample Name: qxk-84-4rac

Signal 1: DAD1 A, Sig=254,4 Ref=off

Signal 2: DAD1 B, Sig=210,4 Ref=360,100

Signal 3: DAD1 D, Sig=230,4 Ref=360,100

Peak #	RetTime [min]	Type	Width [min]	Area [mAU*s]	Height [mAU]	Area %
1	10.945	BB	0.5020	6957.63086	198.70746	49.5012
2	17.022	BBA	0.6426	7097.85889	133.75645	50.4988

Totals : 1.40555e4 332.46391

===

*** End of Report ***

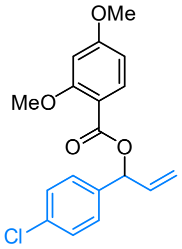

3d-Rac

===

```
Acq. Operator   : SYSTEM                        Seq. Line :    5
Sample Operator : SYSTEM
Acq. Instrument : HPLC                           Location :   P1-F-04
Injection Date  : 1/1/2025 11:44:17 am                Inj :    1
                                               Inj Volume : 2.000 µl
Different Inj Volume from Sample Entry!  Actual Inj Volume : 20.000 µl
Acq. Method     : C:\Users\Public\Documents\ChemStation\1\Data\SUN\SUN 2025-01-01 10-27-09\AD3-
                  20-20.M
Last changed    : 20/12/2024 6:43:38 pm by SYSTEM
Analysis Method : C:\Users\Public\Documents\ChemStation\1\Data\SUN\SUN 2025-01-01 10-27-09\AD3-
                  20-20.M (Sequence Method)
Last changed    : 28/2/2025 4:10:00 pm by SYSTEM
                  (modified after loading)
Additional Info : Peak(s) manually integrated
```

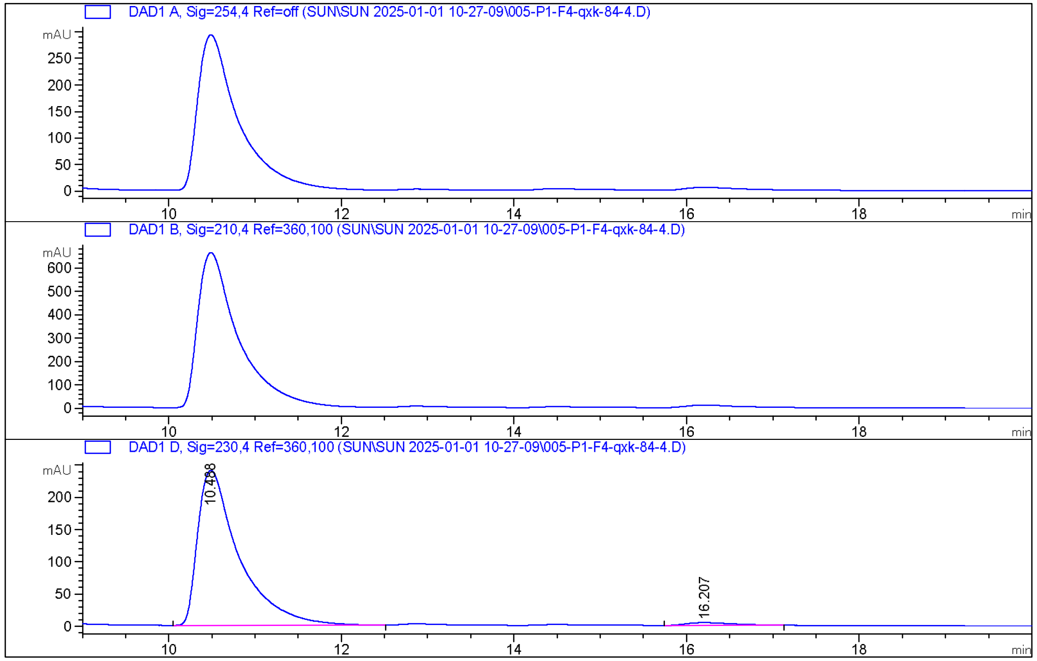

===

Area Percent Report

===

```
Sorted By             :      Signal
Multiplier            :      1.0000
Dilution              :      1.0000
Use Multiplier & Dilution Factor with ISTDs
```


Signal 1: DAD1 A, Sig=254,4 Ref=off

Signal 2: DAD1 B, Sig=210,4 Ref=360,100

Signal 3: DAD1 D, Sig=230,4 Ref=360,100

Peak #	RetTime [min]	Type	Width [min]	Area [mAU*s]	Height [mAU]	Area %
1	10.488	BB	0.4809	8015.73291	240.77151	98.0405
2	16.207	BB	0.4471	160.20404	4.21787	1.9595

Totals : 8175.93695 244.98939

===

*** End of Report ***

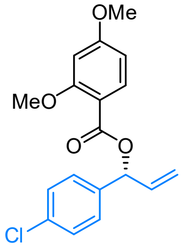

3d

Data File C:\Users\P...mStation\1\Data\SUN\SUN 2024-12-19 11-22-39\004-P1-D3-QXK-76-3RAC.D
Sample Name: QXK-76-3RAC

===

Acq. Operator : SYSTEM Seq. Line : 4
Sample Operator : SYSTEM
Acq. Instrument : HPLC Location : P1-D-03
Injection Date : 19/12/2024 12:18:14 pm Inj : 1
Inj Volume : 2.000 µl
Different Inj Volume from Sample Entry! Actual Inj Volume : 20.000 µl
Acq. Method : C:\Users\Public\Documents\ChemStation\1\Data\SUN\SUN 2024-12-19 11-22-39\AD3-20-20.M
Last changed : 29/11/2022 12:09:46 pm by SYSTEM
Analysis Method : C:\Users\Public\Documents\ChemStation\1\Data\SUN\SUN 2024-12-19 11-22-39\AD3-20-20.M (Sequence Method)
Last changed : 2/1/2025 11:16:13 am by SYSTEM
(modified after loading)
Additional Info : Peak(s) manually integrated

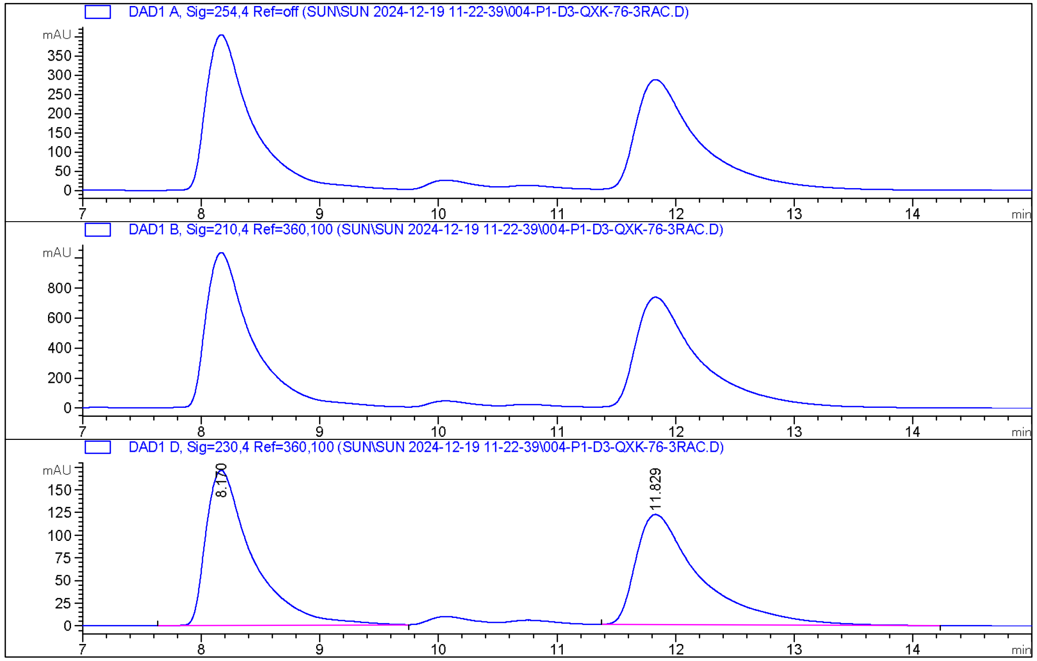

===

Area Percent Report

===

Sorted By : Signal
Multiplier : 1.0000
Dilution : 1.0000
Use Multiplier & Dilution Factor with ISTDs

Data File C:\Users\P...mStation\1\Data\SUN\SUN 2024-12-19 11-22-39\004-P1-D3-QXK-76-3RAC.D
Sample Name: QXK-76-3RAC

Signal 1: DAD1 A, Sig=254,4 Ref=off

Signal 2: DAD1 B, Sig=210,4 Ref=360,100

Signal 3: DAD1 D, Sig=230,4 Ref=360,100

Peak #	RetTime [min]	Type	Width [min]	Area [mAU*s]	Height [mAU]	Area %
1	8.170	BB	0.3966	4671.28711	171.31917	50.5024
2	11.829	BB	0.5277	4578.35400	121.46503	49.4976

Totals : 9249.64111 292.78420

===

*** End of Report ***

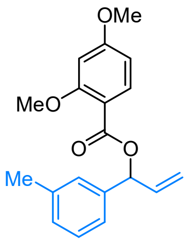

3e-Rac

Data File C:\Users\P...ChemStation\1\Data\SUN\SUN 2025-01-01 13-17-07\085-P1-F1-QXK-84-5.D
Sample Name: QXK-84-5

===

Acq. Operator : SYSTEM Seq. Line : 85
Sample Operator : SYSTEM
Acq. Instrument : HPLC Location : P1-F-01
Injection Date : 2/1/2025 2:49:29 pm Inj : 1
Inj Volume : 2.000 µl
Different Inj Volume from Sample Entry! Actual Inj Volume : 20.000 µl
Acq. Method : C:\Users\Public\Documents\ChemStation\1\Data\SUN\SUN 2025-01-01 13-17-07\AD3-20-20.M
Last changed : 20/12/2024 6:43:38 pm by SYSTEM
Analysis Method : C:\Users\Public\Documents\ChemStation\1\Data\SUN\SUN 2025-01-01 13-17-07\AD3-20-20.M (Sequence Method)
Last changed : 2/1/2025 4:54:28 pm by SYSTEM
(modified after loading)
Additional Info : Peak(s) manually integrated

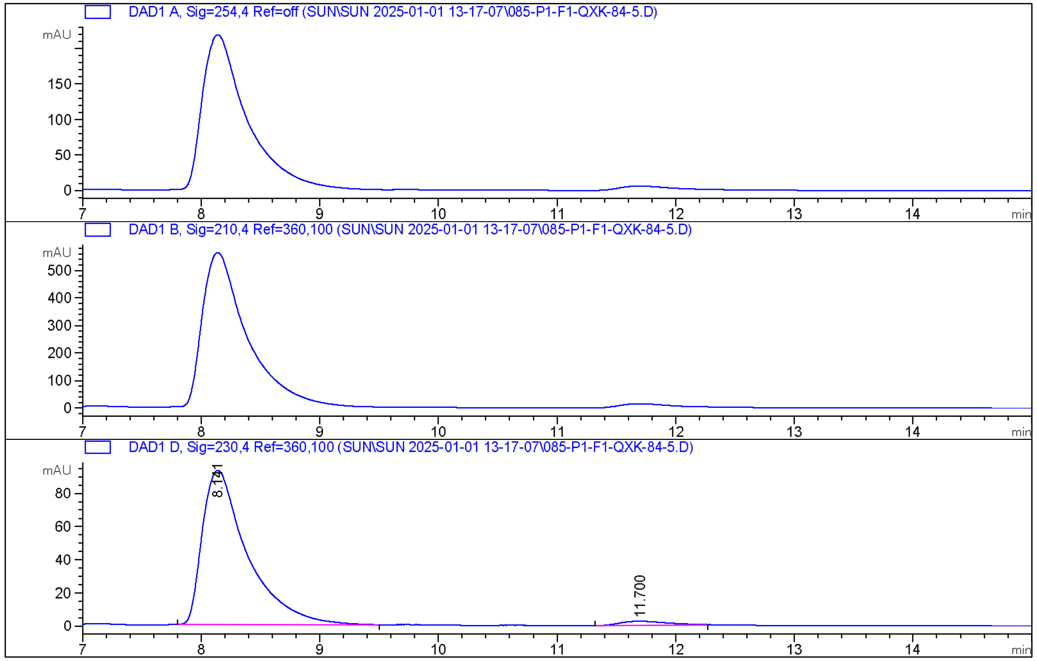

===

Area Percent Report

===

Sorted By : Signal
Multiplier : 1.0000
Dilution : 1.0000
Use Multiplier & Dilution Factor with ISTDs

Data File C:\Users\P...ChemStation\1\Data\SUN\SUN 2025-01-01 13-17-07\085-P1-F1-QXK-84-5.D
Sample Name: QXK-84-5

Signal 1: DAD1 A, Sig=254,4 Ref=off

Signal 2: DAD1 B, Sig=210,4 Ref=360,100

Signal 3: DAD1 D, Sig=230,4 Ref=360,100

Peak #	RetTime [min]	Type	Width [min]	Area [mAU*s]	Height [mAU]	Area %
1	8.141	BB	0.3818	2448.70752	92.97112	97.4350
2	11.700	BB	0.3215	64.46318	2.37992	2.5650

Totals : 2513.17070 95.35104

===

*** End of Report ***

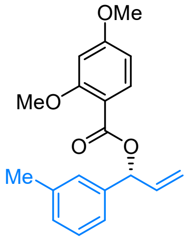

3e

===

Acq. Operator : SYSTEM Seq. Line : 52
Acq. Instrument : LC1260 Location : P2-D-01
Injection Date : 3/2/2025 3:23:21 AM Inj : 1
Inj Volume : 5.000 µl
Different Inj Volume from Sample Entry! Actual Inj Volume : 20.000 µl
Acq. Method : C:\Users\Public\Documents\ChemStation\1\Data\SUN\SUN 2025-03-01 09-55-58\AD3-20-30.M
Last changed : 4/1/2024 7:51:19 PM by SYSTEM
Analysis Method : C:\Users\Public\Documents\ChemStation\1\Data\SUN\SUN 2025-03-01 09-55-58\AD3-20-30.M (Sequence Method)
Last changed : 3/2/2025 2:31:56 PM by SYSTEM
(modified after loading)
Additional Info : Peak(s) manually integrated

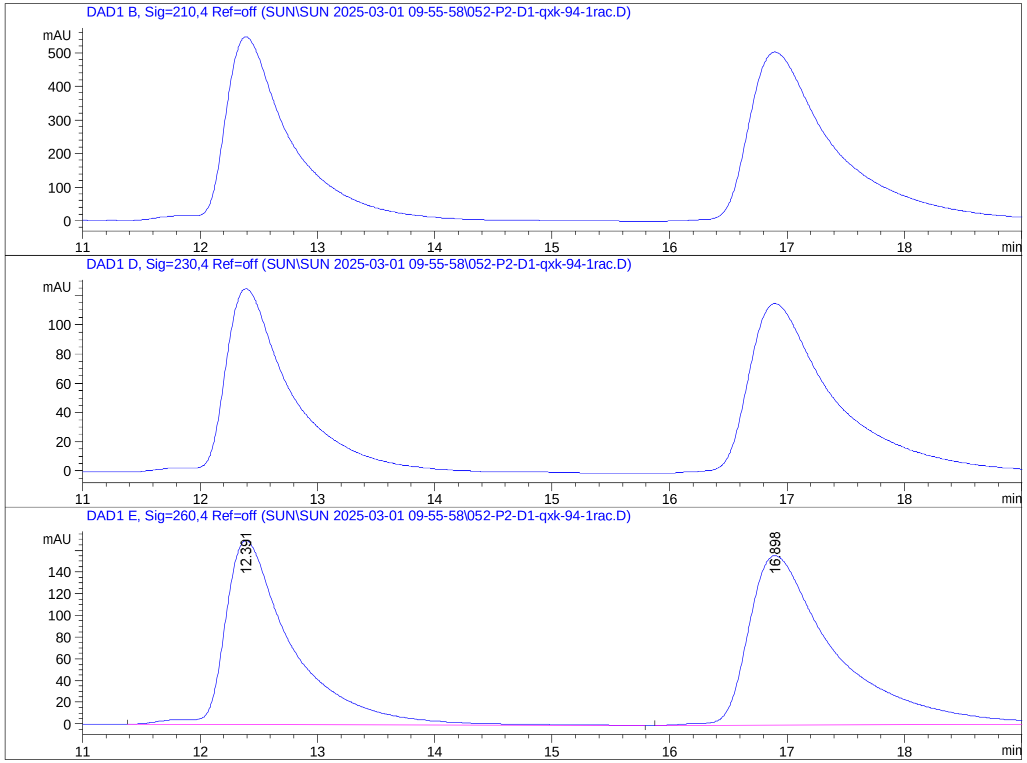

===

Area Percent Report

===

Sorted By : Signal
Multiplier : 1.0000
Dilution : 1.0000
Use Multiplier & Dilution Factor with ISTDs

Data File C:\Users\P...mStation\1\Data\SUN\SUN 2025-03-01 09-55-58\052-P2-D1-qxk-94-1rac.D
Sample Name: qxk-94-1rac

Signal 1: DAD1 B, Sig=210,4 Ref=off

Signal 2: DAD1 D, Sig=230,4 Ref=off

Signal 3: DAD1 E, Sig=260,4 Ref=off

Peak #	RetTime [min]	Type	Width [min]	Area [mAU*s]	Height [mAU]	Area %
1	12.391	BB	0.5650	6672.17676	169.58603	45.1327
2	16.898	BB	0.7442	8111.28711	156.10005	54.8673

Totals : 1.47835e4 325.68608

===

*** End of Report ***

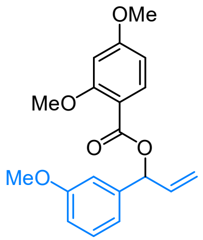

3f-Rac

===

Acq. Operator : SYSTEM Seq. Line : 19
Sample Operator : SYSTEM
Acq. Instrument : HPLC Location : P1-E-01
Injection Date : 16/1/2025 4:33:20 pm Inj : 1
Inj Volume : 2.000 µl
Different Inj Volume from Sample Entry! Actual Inj Volume : 20.000 µl
Acq. Method : C:\Users\Public\Documents\ChemStation\1\Data\SUN\SUN 2025-01-16 10-32-15\AD3-20-30.M
Last changed : 18/8/2022 9:21:31 pm by SYSTEM
Analysis Method : C:\Users\Public\Documents\ChemStation\1\Data\SUN\SUN 2025-01-16 10-32-15\AD3-20-30.M (Sequence Method)
Last changed : 28/2/2025 4:01:31 pm by SYSTEM
(modified after loading)
Additional Info : Peak(s) manually integrated

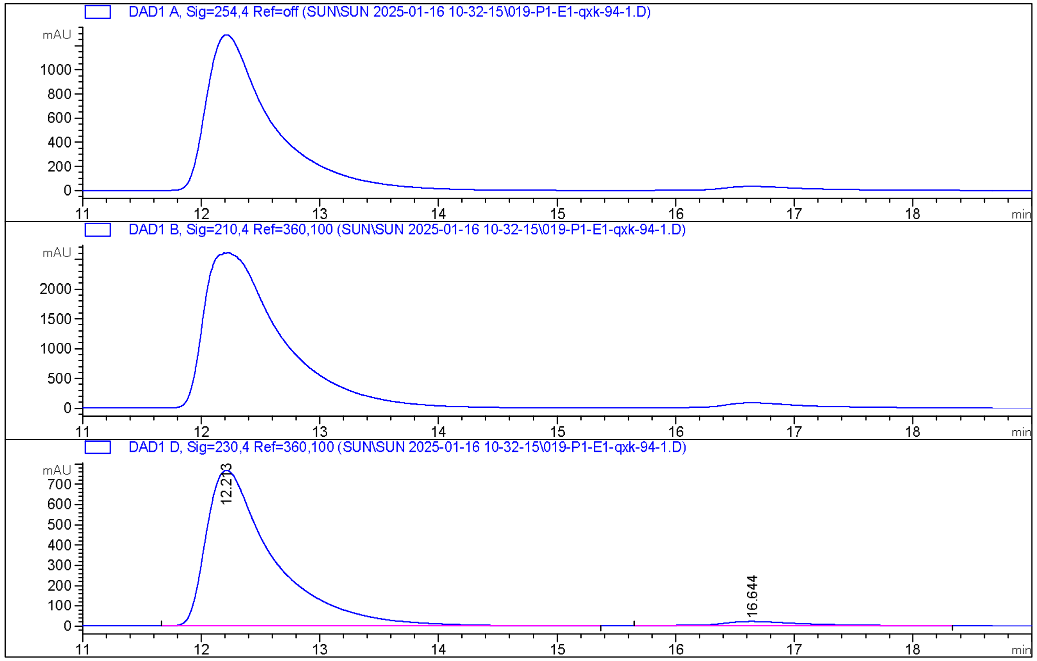

===

Area Percent Report

===

Sorted By : Signal
Multiplier : 1.0000
Dilution : 1.0000
Use Multiplier & Dilution Factor with ISTDs

Data File C:\Users\P...ChemStation\1\Data\SUN\SUN 2025-01-16 10-32-15\019-P1-E1-qxk-94-1.D
Sample Name: qxk-94-1

Signal 1: DAD1 A, Sig=254,4 Ref=off

Signal 2: DAD1 B, Sig=210,4 Ref=360,100

Signal 3: DAD1 D, Sig=230,4 Ref=360,100

Peak #	RetTime [min]	Type	Width [min]	Area [mAU*s]	Height [mAU]	Area %
1	12.213	BB	0.5624	3.01346e4	767.78644	96.5850
2	16.644	BB	0.5861	1065.49463	21.30164	3.4150

Totals : 3.12001e4 789.08808

===

*** End of Report ***

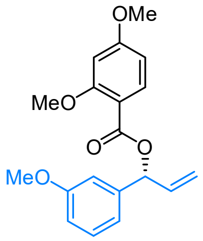

3f

Data File C:\Users\P...mStation\1\Data\SUN\SUN 2025-02-14 15-47-48\004-P1-F2-qxk-84-6rac.D
Sample Name: qxk-84-6rac

===

Acq. Operator : SYSTEM Seq. Line : 4
Sample Operator : SYSTEM
Acq. Instrument : HPLC Location : P1-F-02
Injection Date : 14/2/2025 4:34:36 pm Inj : 1
Inj Volume : 2.000 µl
Different Inj Volume from Sample Entry! Actual Inj Volume : 20.000 µl
Acq. Method : C:\Users\Public\Documents\ChemStation\1\Data\SUN\SUN 2025-02-14 15-47-48\AD3-20-20.M
Last changed : 20/12/2024 6:43:38 pm by SYSTEM
Analysis Method : C:\Users\Public\Documents\ChemStation\1\Data\SUN\SUN 2025-02-14 15-47-48\AD3-20-20.M (Sequence Method)
Last changed : 28/2/2025 4:11:02 pm by SYSTEM
(modified after loading)
Additional Info : Peak(s) manually integrated

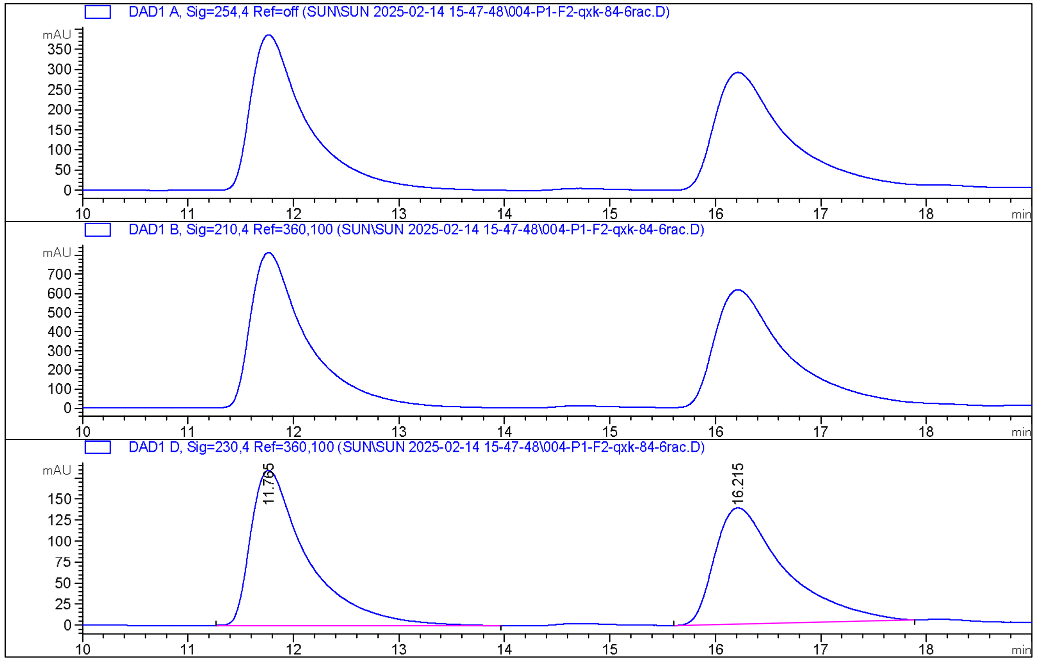

===

Area Percent Report

===

Sorted By : Signal
Multiplier : 1.0000
Dilution : 1.0000
Use Multiplier & Dilution Factor with ISTDs

Data File C:\Users\P...mStation\1\Data\SUN\SUN 2025-02-14 15-47-48\004-P1-F2-qxk-84-6rac.D
Sample Name: qxk-84-6rac

Signal 1: DAD1 A, Sig=254,4 Ref=off

Signal 2: DAD1 B, Sig=210,4 Ref=360,100

Signal 3: DAD1 D, Sig=230,4 Ref=360,100

Peak #	RetTime [min]	Type	Width [min]	Area [mAU*s]	Height [mAU]	Area %
1	11.765	BB	0.5190	6849.76953	184.78423	51.8936
2	16.215	BB	0.6120	6349.87354	138.21751	48.1064

Totals : 1.31996e4 323.00174

===

*** End of Report ***

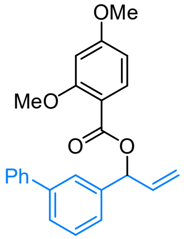

3g-Rac

Data File C:\Users\P...ChemStation\1\Data\SUN\SUN 2025-01-01 13-17-07\086-P1-F2-QXK-84-6.D
Sample Name: QXK-84-6

===

Acq. Operator : SYSTEM Seq. Line : 86
Sample Operator : SYSTEM
Acq. Instrument : HPLC Location : P1-F-02
Injection Date : 2/1/2025 3:10:40 pm Inj : 1
Inj Volume : 2.000 µl
Different Inj Volume from Sample Entry! Actual Inj Volume : 20.000 µl
Acq. Method : C:\Users\Public\Documents\ChemStation\1\Data\SUN\SUN 2025-01-01 13-17-07\AD3-20-20.M
Last changed : 20/12/2024 6:43:38 pm by SYSTEM
Analysis Method : C:\Users\Public\Documents\ChemStation\1\Data\SUN\SUN 2025-01-01 13-17-07\AD3-20-20.M (Sequence Method)
Last changed : 2/1/2025 5:01:56 pm by SYSTEM
(modified after loading)
Additional Info : Peak(s) manually integrated

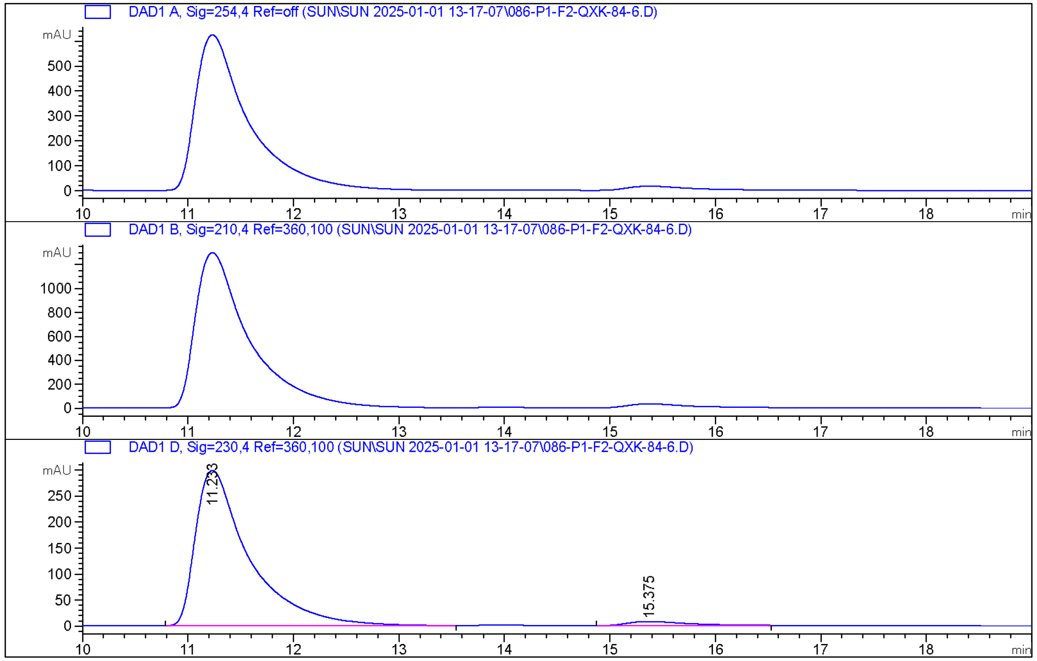

===

Area Percent Report

===

Sorted By : Signal
Multiplier : 1.0000
Dilution : 1.0000
Use Multiplier & Dilution Factor with ISTDs

Data File C:\Users\P...ChemStation\1\Data\SUN\SUN 2025-01-01 13-17-07\086-P1-F2-QXK-84-6.D
Sample Name: QXK-84-6

Signal 1: DAD1 A, Sig=254,4 Ref=off

Signal 2: DAD1 B, Sig=210,4 Ref=360,100

Signal 3: DAD1 D, Sig=230,4 Ref=360,100

Peak #	RetTime [min]	Type	Width [min]	Area [mAU*s]	Height [mAU]	Area %
1	11.233	BB	0.5083	1.05433e4	298.72797	97.2188
2	15.375	BB	0.4571	301.62164	7.74899	2.7812

Totals : 1.08449e4 306.47695

===

*** End of Report ***

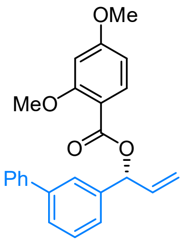

3g

===

Acq. Operator : SYSTEM Seq. Line : 6
Sample Operator : SYSTEM
Acq. Instrument : HPLC Location : P1-F-03
Injection Date : 14/2/2025 5:07:21 pm Inj : 1
Inj Volume : 2.000 µl
Different Inj Volume from Sample Entry! Actual Inj Volume : 20.000 µl
Acq. Method : C:\Users\Public\Documents\ChemStation\1\Data\SUN\SUN 2025-02-14 15-47-48\AD3-20-20.M
Last changed : 20/12/2024 6:43:38 pm by SYSTEM
Analysis Method : C:\Users\Public\Documents\ChemStation\1\Data\SUN\SUN 2025-02-14 15-47-48\AD3-20-20.M (Sequence Method)
Last changed : 28/2/2025 4:13:42 pm by SYSTEM
(modified after loading)
Additional Info : Peak(s) manually integrated

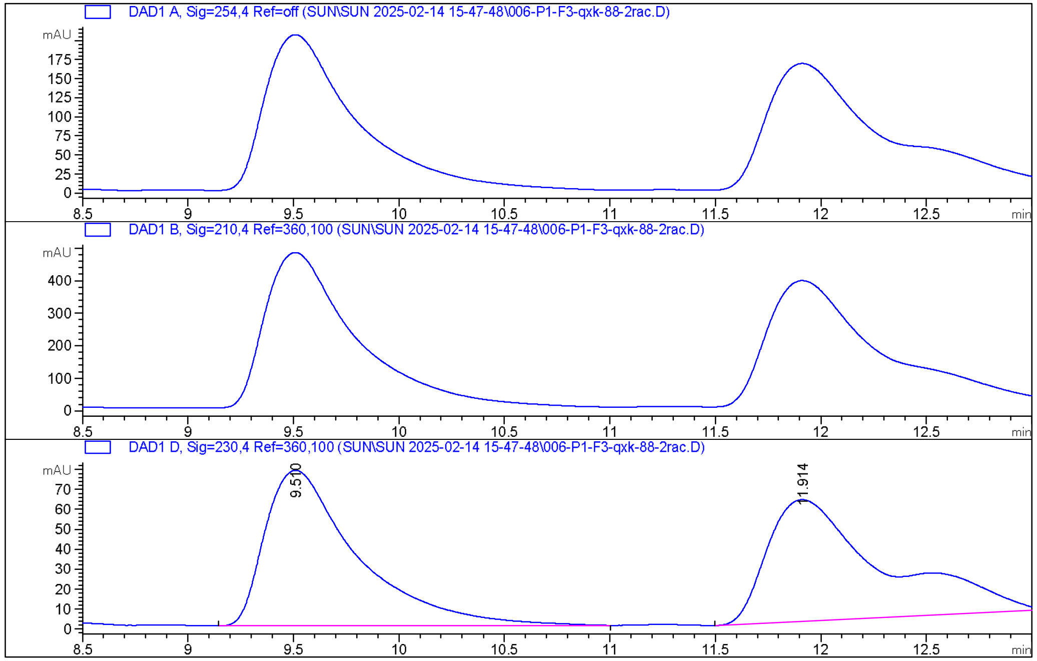

===

Area Percent Report

===

Sorted By : Signal
Multiplier : 1.0000
Dilution : 1.0000
Use Multiplier & Dilution Factor with ISTDs

Data File C:\Users\P...mStation\1\Data\SUN\SUN 2025-02-14 15-47-48\006-P1-F3-qxk-88-2rac.D
Sample Name: qxk-88-2rac

Signal 1: DAD1 A, Sig=254,4 Ref=off

Signal 2: DAD1 B, Sig=210,4 Ref=360,100

Signal 3: DAD1 D, Sig=230,4 Ref=360,100

Peak #	RetTime [min]	Type	Width [min]	Area [mAU*s]	Height [mAU]	Area %
1	9.510	BB	0.4260	2367.66846	78.03595	50.7195
2	11.914	MM R	0.6284	2300.49194	61.01480	49.2805
Totals :				4668.16040	139.05075	

===

*** End of Report ***

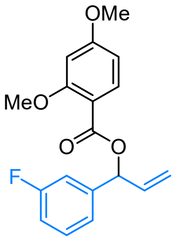

3h-Rac

===

```
Acq. Operator   : SYSTEM                        Seq. Line :   3
Sample Operator : SYSTEM
Acq. Instrument : HPLC                           Location :   P1-F-02
Injection Date  : 9/1/2025 3:59:32 pm                 Inj :   1
                                               Inj Volume : 2.000 µl
Different Inj Volume from Sample Entry!  Actual Inj Volume : 10.000 µl
Acq. Method     : C:\Users\Public\Documents\ChemStation\1\Data\SUN\SUN 2025-01-09 15-25-10\AD3-
                  20-20.M
Last changed    : 20/12/2024 6:43:38 pm by SYSTEM
Analysis Method : C:\Users\Public\Documents\ChemStation\1\Data\SUN\SUN 2025-01-09 15-25-10\AD3-
                  20-20.M (Sequence Method)
Last changed    : 11/1/2025 4:30:57 pm by SYSTEM
                  (modified after loading)
Additional Info : Peak(s) manually integrated
```

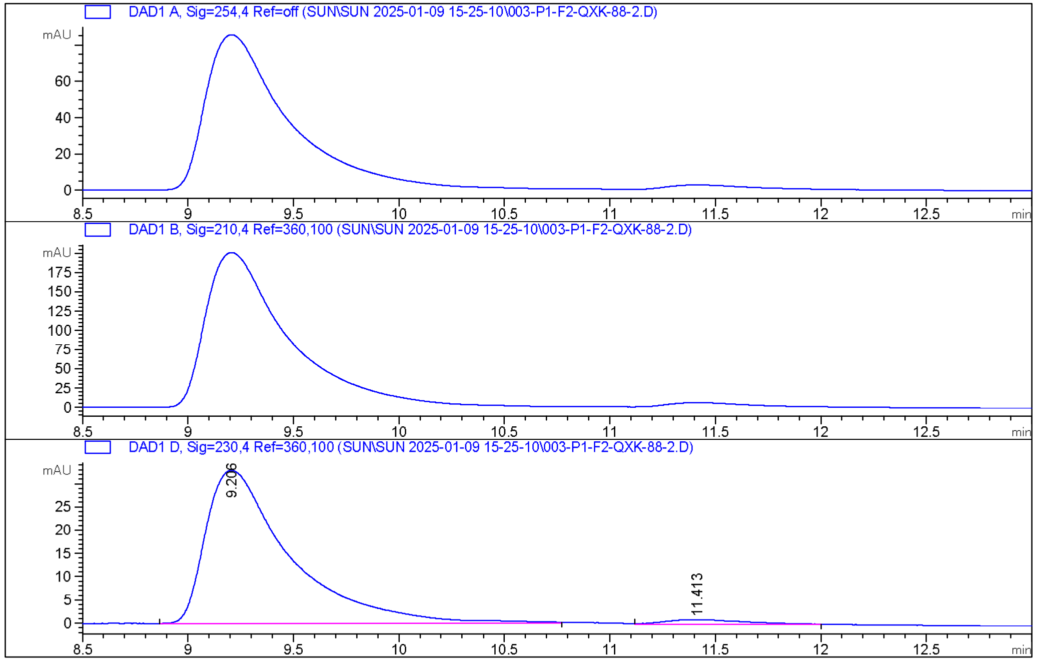

===
Area Percent Report
===

```
Sorted By             :      Signal
Multiplier            :      1.0000
Dilution              :      1.0000
Use Multiplier & Dilution Factor with ISTDs
```


Data File C:\Users\P...ChemStation\1\Data\SUN\SUN 2025-01-09 15-25-10\003-P1-F2-QXK-88-2.D
Sample Name: QXK-88-2

Signal 1: DAD1 A, Sig=254,4 Ref=off

Signal 2: DAD1 B, Sig=210,4 Ref=360,100

Signal 3: DAD1 D, Sig=230,4 Ref=360,100

Peak #	RetTime [min]	Type	Width [min]	Area [mAU*s]	Height [mAU]	Area %
1	9.206	MM R	0.4628	918.19989	33.06670	96.8705
2	11.413	MM R	0.4542	29.66337	1.08848	3.1295

Totals : 947.86326 34.15518

===
*** End of Report ***

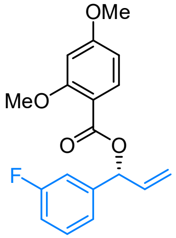

3h

===

Acq. Operator : SYSTEM Seq. Line : 2
Sample Operator : SYSTEM
Acq. Instrument : HPLC Location : P1-D-01
Injection Date : 19/12/2024 11:35:54 am Inj : 1
Inj Volume : 2.000 µl
Different Inj Volume from Sample Entry! Actual Inj Volume : 20.000 µl
Acq. Method : C:\Users\Public\Documents\ChemStation\1\Data\SUN\SUN 2024-12-19 11-22-39\AD3-20-20.M
Last changed : 29/11/2022 12:09:46 pm by SYSTEM
Analysis Method : C:\Users\Public\Documents\ChemStation\1\Data\SUN\SUN 2024-12-19 11-22-39\AD3-20-20.M (Sequence Method)
Last changed : 7/1/2025 9:15:59 pm by SYSTEM
(modified after loading)
Additional Info : Peak(s) manually integrated

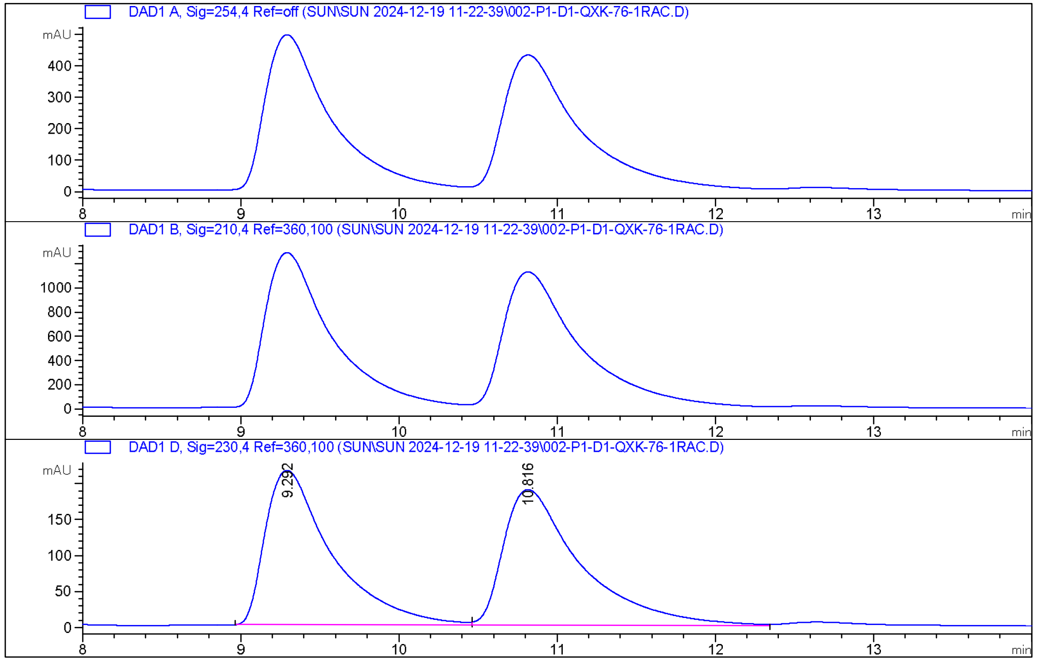

===
Area Percent Report
===

Sorted By : Signal
Multiplier : 1.0000
Dilution : 1.0000
Use Multiplier & Dilution Factor with ISTDs

Data File C:\Users\P...mStation\1\Data\SUN\SUN 2024-12-19 11-22-39\002-P1-D1-QXK-76-1RAC.D
Sample Name: QXK-76-1RAC

Signal 1: DAD1 A, Sig=254,4 Ref=off

Signal 2: DAD1 B, Sig=210,4 Ref=360,100

Signal 3: DAD1 D, Sig=230,4 Ref=360,100

Peak #	RetTime [min]	Type	Width [min]	Area [mAU*s]	Height [mAU]	Area %
1	9.292	MF R	0.4892	6302.58594	214.72107	49.4540
2	10.816	FM R	0.5690	6441.75879	188.69547	50.5460

Totals : 1.27443e4 403.41653

===
*** End of Report ***

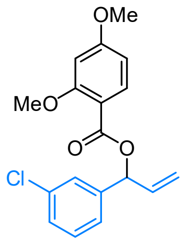

3i-Rac

===

Acq. Operator : SYSTEM Seq. Line : 2
Sample Operator : SYSTEM
Acq. Instrument : HPLC Location : P1-F-01
Injection Date : 9/1/2025 3:38:28 pm Inj : 1
Inj Volume : 2.000 µl
Different Inj Volume from Sample Entry! Actual Inj Volume : 10.000 µl
Acq. Method : C:\Users\Public\Documents\ChemStation\1\Data\SUN\SUN 2025-01-09 15-25-10\AD3-20-20.M
Last changed : 20/12/2024 6:43:38 pm by SYSTEM
Analysis Method : C:\Users\Public\Documents\ChemStation\1\Data\SUN\SUN 2025-01-09 15-25-10\AD3-20-20.M (Sequence Method)
Last changed : 11/1/2025 4:28:45 pm by SYSTEM
(modified after loading)
Additional Info : Peak(s) manually integrated

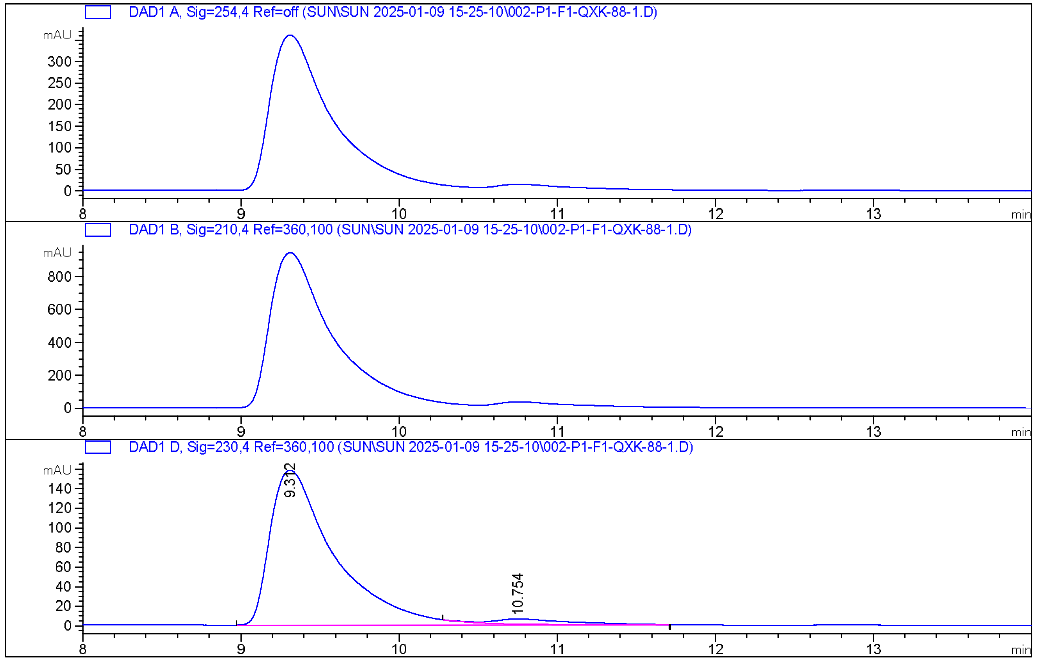

===

Area Percent Report

===

Sorted By : Signal
Multiplier : 1.0000
Dilution : 1.0000
Use Multiplier & Dilution Factor with ISTDs

Data File C:\Users\P...ChemStation\1\Data\SUN\SUN 2025-01-09 15-25-10\002-P1-F1-QXK-88-1.D
Sample Name: QXK-88-1

Signal 1: DAD1 A, Sig=254,4 Ref=off

Signal 2: DAD1 B, Sig=210,4 Ref=360,100

Signal 3: DAD1 D, Sig=230,4 Ref=360,100

Peak #	RetTime [min]	Type	Width [min]	Area [mAU*s]	Height [mAU]	Area %
1	9.312	BV R	0.4083	4490.34863	158.10561	96.2423
2	10.754	VB E	0.3850	175.32324	5.35516	3.7577

Totals : 4665.67188 163.46077

===

*** End of Report ***

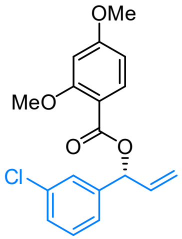

3i

===

```
Acq. Operator   : SYSTEM                      Seq. Line :    5
Sample Operator : SYSTEM
Acq. Instrument : HPLC                         Location :   P1-D-04
Injection Date  : 19/12/2024 12:39:38 pm            Inj :    1
                                             Inj Volume : 2.000 µl
Different Inj Volume from Sample Entry!  Actual Inj Volume : 20.000 µl
Acq. Method     : C:\Users\Public\Documents\ChemStation\1\Data\SUN\SUN 2024-12-19 11-22-39\AD3-
                  20-20.M
Last changed    : 29/11/2022 12:09:46 pm by SYSTEM
Analysis Method : C:\Users\Public\Documents\ChemStation\1\Data\SUN\SUN 2024-12-19 11-22-39\AD3-
                  20-20.M (Sequence Method)
Last changed    : 7/1/2025 9:17:35 pm by SYSTEM
                  (modified after loading)
Additional Info : Peak(s) manually integrated
```

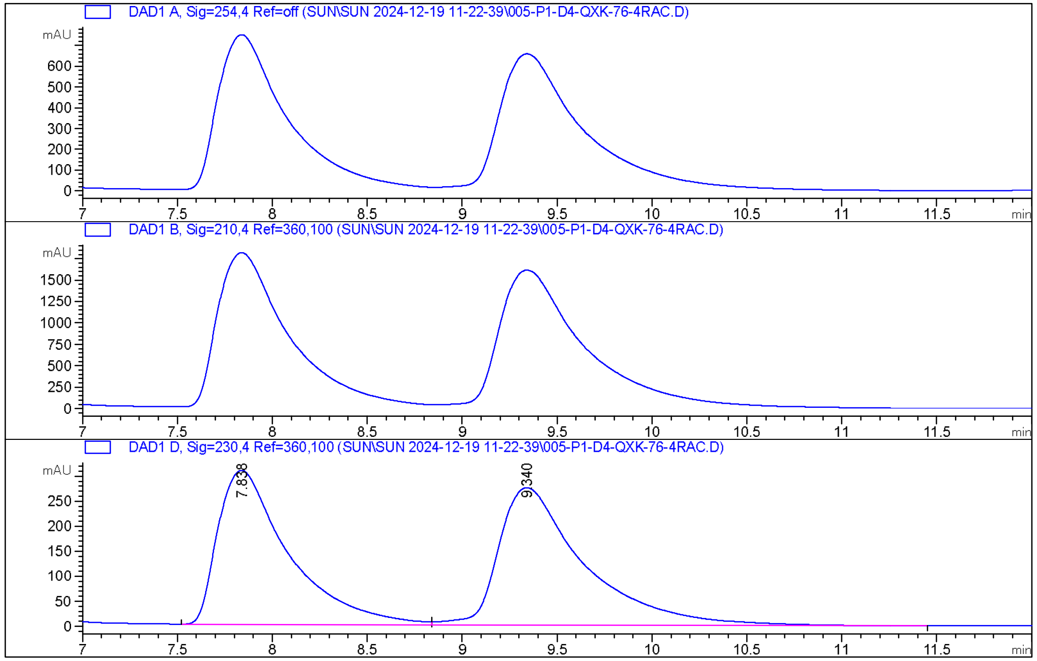

===

Area Percent Report

===

```
Sorted By          :     Signal
Multiplier         :     1.0000
Dilution           :     1.0000
Use Multiplier & Dilution Factor with ISTDs
```


Signal 1: DAD1 A, Sig=254,4 Ref=off

Signal 2: DAD1 B, Sig=210,4 Ref=360,100

Signal 3: DAD1 D, Sig=230,4 Ref=360,100

Peak #	RetTime [min]	Type	Width [min]	Area [mAU*s]	Height [mAU]	Area %
1	7.838	VV	0.3803	7978.98926	307.92505	48.1158
2	9.340	VB	0.4521	8603.90820	274.15912	51.8842

Totals : 1.65829e4 582.08417

===

*** End of Report ***

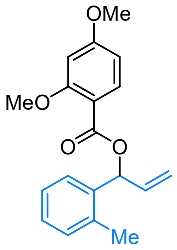

3j-Rac

===

Acq. Operator : SYSTEM Seq. Line : 4
Sample Operator : SYSTEM
Acq. Instrument : HPLC Location : P1-F-03
Injection Date : 9/1/2025 4:20:37 pm Inj : 1
Inj Volume : 2.000 µl
Different Inj Volume from Sample Entry! Actual Inj Volume : 10.000 µl
Acq. Method : C:\Users\Public\Documents\ChemStation\1\Data\SUN\SUN 2025-01-09 15-25-10\AD3-20-20.M
Last changed : 20/12/2024 6:43:38 pm by SYSTEM
Analysis Method : C:\Users\Public\Documents\ChemStation\1\Data\SUN\SUN 2025-01-09 15-25-10\AD3-20-20.M (Sequence Method)
Last changed : 11/1/2025 4:32:05 pm by SYSTEM
(modified after loading)
Additional Info : Peak(s) manually integrated

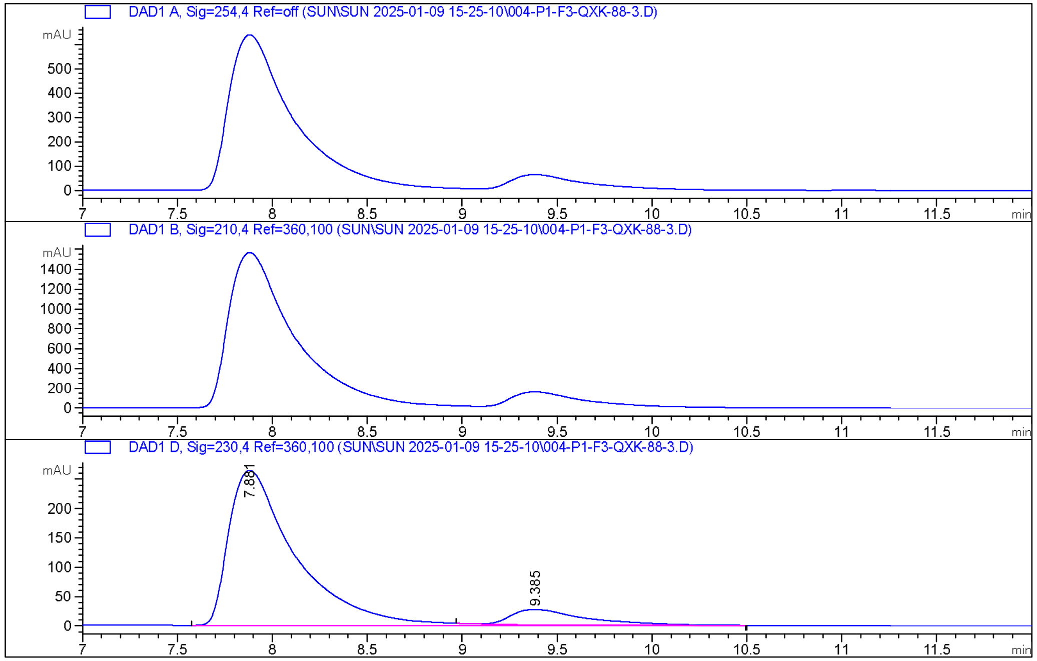

===

Area Percent Report

===

Sorted By : Signal
Multiplier : 1.0000
Dilution : 1.0000
Use Multiplier & Dilution Factor with ISTDs

Data File C:\Users\P...ChemStation\1\Data\SUN\SUN 2025-01-09 15-25-10\004-P1-F3-QXK-88-3.D
Sample Name: QXK-88-3

Signal 1: DAD1 A, Sig=254,4 Ref=off

Signal 2: DAD1 B, Sig=210,4 Ref=360,100

Signal 3: DAD1 D, Sig=230,4 Ref=360,100

Peak #	RetTime [min]	Type	Width [min]	Area [mAU*s]	Height [mAU]	Area %
1	7.881	BV R	0.3569	6479.66064	264.42621	90.0629
2	9.385	VB E	0.3642	714.93372	26.50661	9.9371

Totals : 7194.59436 290.93282

===
*** End of Report ***

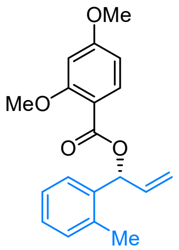

3j

===

Acq. Operator : SYSTEM Seq. Line : 6
Acq. Instrument : LC1260 Location : P2-F-05
Injection Date : 12/19/2024 8:15:29 PM Inj : 1
Inj Volume : 5.000 µl
Different Inj Volume from Sample Entry! Actual Inj Volume : 20.000 µl
Acq. Method : C:\Users\Public\Documents\ChemStation\1\Data\SUN\SUN 2024-12-19 17-58-10\IC3-20-30.M
Last changed : 4/1/2024 7:53:22 PM by SYSTEM
Analysis Method : C:\Users\Public\Documents\ChemStation\1\Data\SUN\SUN 2024-12-19 17-58-10\IC3-20-30.M (Sequence Method)
Last changed : 1/7/2025 8:36:39 PM by SYSTEM
(modified after loading)
Additional Info : Peak(s) manually integrated

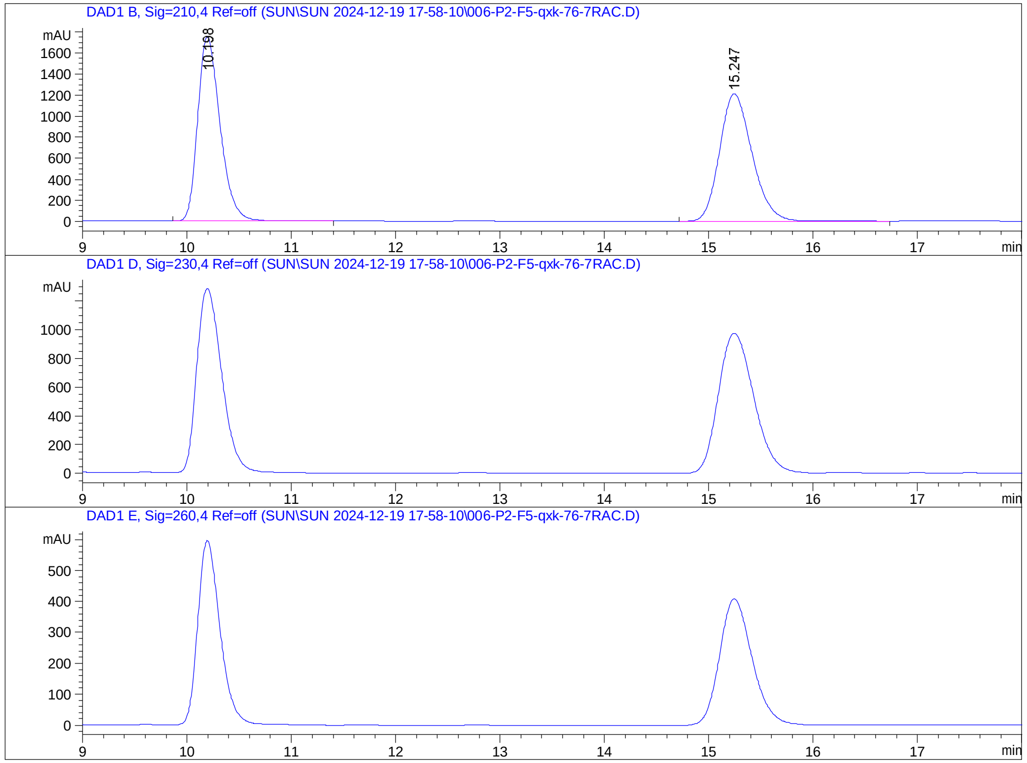

===

Area Percent Report

===

Sorted By : Signal
Multiplier : 1.0000
Dilution : 1.0000
Use Multiplier & Dilution Factor with ISTDs

Data File C:\Users\P...mStation\1\Data\SUN\SUN 2024-12-19 17-58-10\006-P2-F5-qxk-76-7RAC.D
Sample Name: qxk-76-7RAC

Signal 1: DAD1 B, Sig=210,4 Ref=off

Peak #	RetTime [min]	Type	Width [min]	Area [mAU*s]	Height [mAU]	Area %
1	10.198	BB	0.2307	2.62284e4	1751.65125	49.8724
2	15.247	BB R	0.3353	2.63626e4	1210.23608	50.1276

Totals : 5.25910e4 2961.88733

Signal 2: DAD1 D, Sig=230,4 Ref=off

Signal 3: DAD1 E, Sig=260,4 Ref=off

===

*** End of Report ***

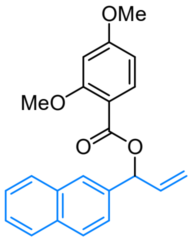

3k-Rac

===

Acq. Operator : SYSTEM Seq. Line : 47
Acq. Instrument : LC1260 Location : P1-F-06
Injection Date : 1/2/2025 12:55:38 AM Inj : 1
Inj Volume : 5.000 µl
Different Inj Volume from Sample Entry! Actual Inj Volume : 20.000 µl
Acq. Method : C:\Users\Public\Documents\ChemStation\1\Data\SUN\SUN 2025-01-01 13-44-33\IC3-20-30.M
Last changed : 4/1/2024 7:53:22 PM by SYSTEM
Analysis Method : C:\Users\Public\Documents\ChemStation\1\Data\SUN\SUN 2025-01-01 13-44-33\IC3-20-30.M (Sequence Method)
Last changed : 1/7/2025 8:43:30 PM by SYSTEM
(modified after loading)
Additional Info : Peak(s) manually integrated

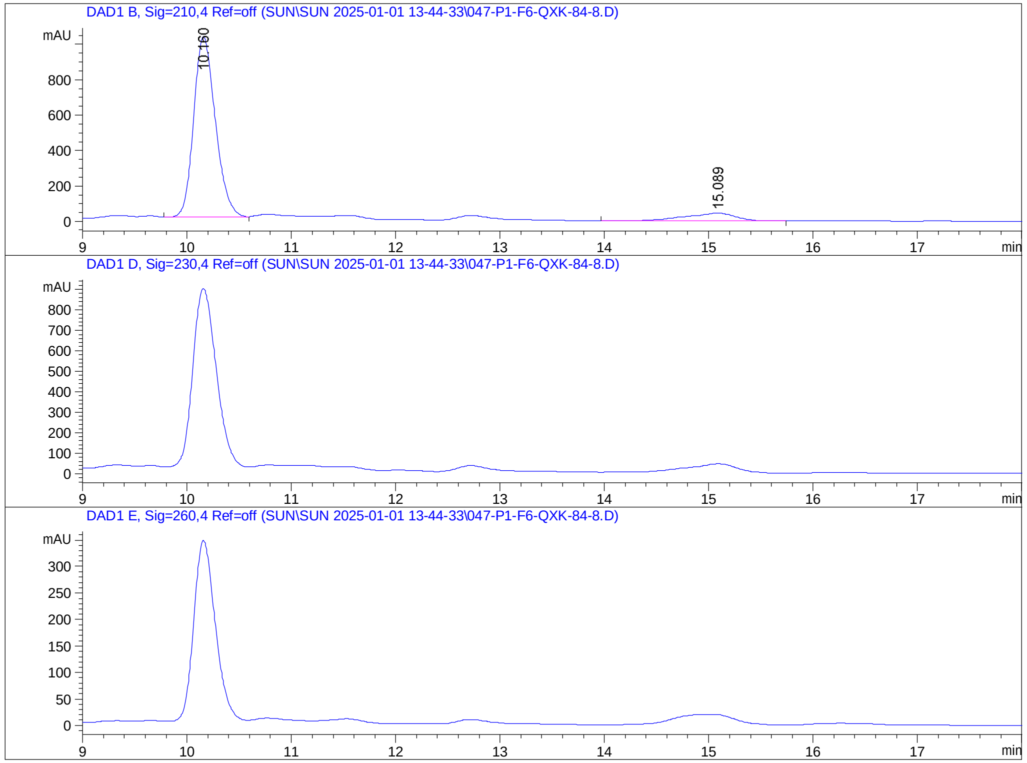

===

Area Percent Report

===

Sorted By : Signal
Multiplier : 1.0000
Dilution : 1.0000
Use Multiplier & Dilution Factor with ISTDs

Data File C:\Users\P...ChemStation\1\Data\SUN\SUN 2025-01-01 13-44-33\047-P1-F6-QXK-84-8.D
Sample Name: QXK-84-8

Signal 1: DAD1 B, Sig=210,4 Ref=off

Peak #	RetTime [min]	Type	Width [min]	Area [mAU*s]	Height [mAU]	Area %
1	10.160	MM R	0.2415	1.47570e4	1018.33594	90.6518
2	15.089	BB	0.4773	1521.76978	44.24553	9.3482
Totals :				1.62788e4	1062.58147	

Signal 2: DAD1 D, Sig=230,4 Ref=off

Signal 3: DAD1 E, Sig=260,4 Ref=off

===

*** End of Report ***

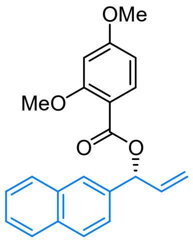

3k

Data File C:\Users\P...mStation\1\Data\SUN\SUN 2025-01-08 20-03-36\003-P1-F2-qxk-88-5rac.D
Sample Name: qxk-88-5rac

===

```
Acq. Operator   : SYSTEM                        Seq. Line :   3
Sample Operator : SYSTEM
Acq. Instrument : HPLC                           Location :   P1-F-02
Injection Date  : 8/1/2025 8:57:52 pm                 Inj :   1
                                               Inj Volume : 2.000 µl
Different Inj Volume from Sample Entry!  Actual Inj Volume : 20.000 µl
Acq. Method     : C:\Users\Public\Documents\ChemStation\1\Data\SUN\SUN 2025-01-08 20-03-36\AD3-
                  01-40.M
Last changed    : 7/10/2022 10:49:42 pm by SYSTEM
Analysis Method : C:\Users\Public\Documents\ChemStation\1\Data\SUN\SUN 2025-01-08 20-03-36\AD3-
                  01-40.M (Sequence Method)
Last changed    : 9/1/2025 2:58:45 pm by SYSTEM
                  (modified after loading)
Additional Info : Peak(s) manually integrated
```

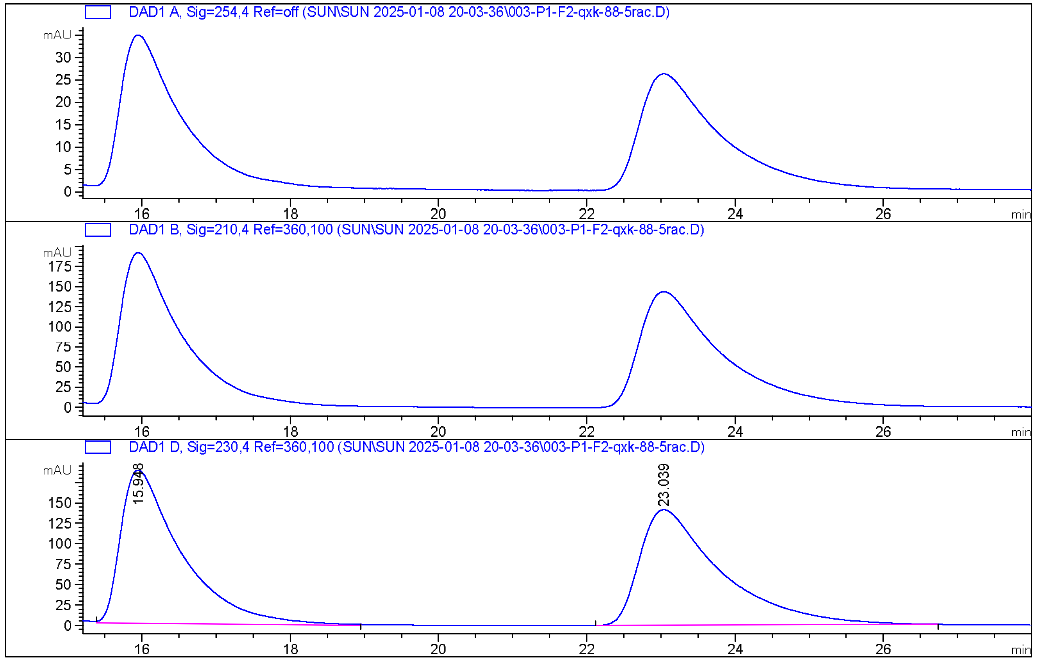

===
Area Percent Report
===

```
Sorted By             :      Signal
Multiplier            :      1.0000
Dilution              :      1.0000
Use Multiplier & Dilution Factor with ISTDs
```


Data File C:\Users\P...mStation\1\Data\SUN\SUN 2025-01-08 20-03-36\003-P1-F2-qxk-88-5rac.D
Sample Name: qxk-88-5rac

Signal 1: DAD1 A, Sig=254,4 Ref=off

Signal 2: DAD1 B, Sig=210,4 Ref=360,100

Signal 3: DAD1 D, Sig=230,4 Ref=360,100

Peak #	RetTime [min]	Type	Width [min]	Area [mAU*s]	Height [mAU]	Area %
1	15.948	MM R	0.9493	1.06826e4	187.54852	49.7550
2	23.039	BB	0.9146	1.07878e4	141.55721	50.2450

Totals : 2.14705e4 329.10573

===
*** End of Report ***

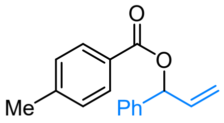

3l-Rac

===

Acq. Operator : SYSTEM Seq. Line : 2
Sample Operator : SYSTEM
Acq. Instrument : HPLC Location : P1-F-01
Injection Date : 8/1/2025 8:16:32 pm Inj : 1
Inj Volume : 2.000 µl
Different Inj Volume from Sample Entry! Actual Inj Volume : 20.000 µl
Acq. Method : C:\Users\Public\Documents\ChemStation\1\Data\SUN\SUN 2025-01-08 20-03-36\AD3-01-40.M
Last changed : 7/10/2022 10:49:42 pm by SYSTEM
Analysis Method : C:\Users\Public\Documents\ChemStation\1\Data\SUN\SUN 2025-01-08 20-03-36\AD3-01-40.M (Sequence Method)
Last changed : 9/1/2025 2:58:45 pm by SYSTEM
(modified after loading)
Additional Info : Peak(s) manually integrated

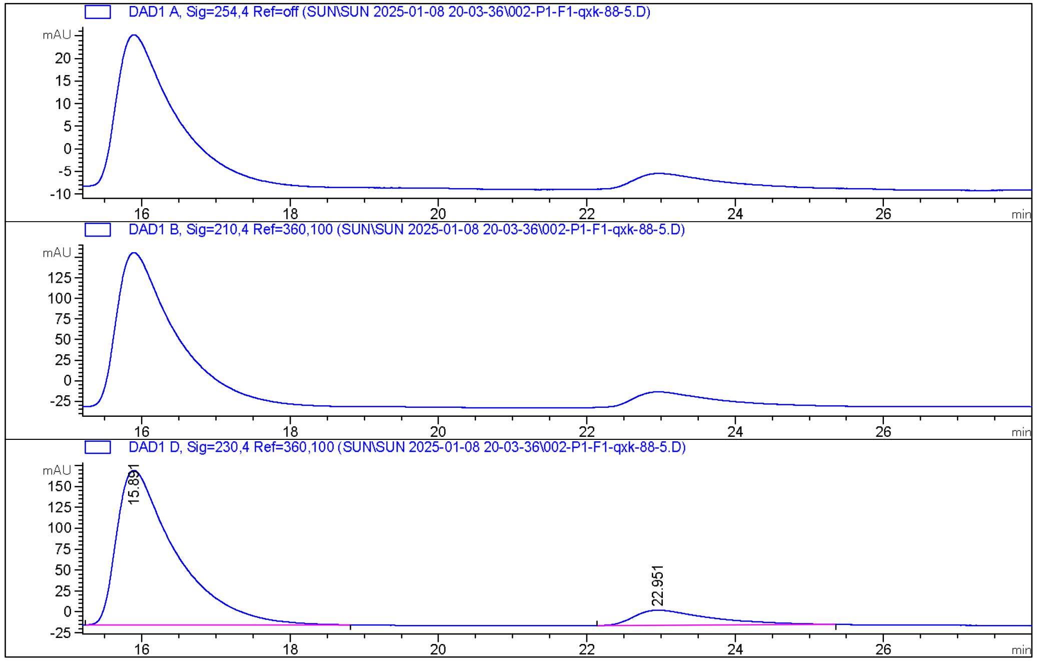

===
Area Percent Report
===

Sorted By : Signal
Multiplier : 1.0000
Dilution : 1.0000
Use Multiplier & Dilution Factor with ISTDs

Data File C:\Users\P...ChemStation\1\Data\SUN\SUN 2025-01-08 20-03-36\002-P1-F1-qxk-88-5.D
Sample Name: qxk-88-5

Signal 1: DAD1 A, Sig=254,4 Ref=off

Signal 2: DAD1 B, Sig=210,4 Ref=360,100

Signal 3: DAD1 D, Sig=230,4 Ref=360,100

Peak #	RetTime [min]	Type	Width [min]	Area [mAU*s]	Height [mAU]	Area %
1	15.891	BB	0.7438	1.02498e4	184.88519	88.8060
2	22.951	BB	0.8404	1291.99329	17.97937	11.1940

Totals : 1.15418e4 202.86456

===
*** End of Report ***

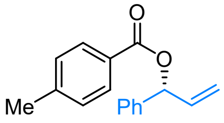

3l

Data File C:\Users\P...Station\1\Data\SUN\SUN 2024-11-20 17-21-08\039-P1-E4-qxk-46-4-rac.D
Sample Name: qxk-46-4-rac

===

```
Acq. Operator   : SYSTEM                        Seq. Line :  39
Sample Operator : SYSTEM
Acq. Instrument : HPLC                           Location :   P1-E-04
Injection Date  : 21/11/2024 4:28:48 am               Inj :   1
                                               Inj Volume : 2.000 µl
Different Inj Volume from Sample Entry!  Actual Inj Volume : 20.000 µl
Acq. Method     : C:\Users\Public\Documents\ChemStation\1\Data\SUN\SUN 2024-11-20 17-21-08\AD3-
                  02-40.M
Last changed    : 11/3/2023 2:01:17 pm by SYSTEM
Analysis Method : C:\Users\Public\Documents\ChemStation\1\Data\SUN\SUN 2024-11-20 17-21-08\AD3-
                  02-40.M (Sequence Method)
Last changed    : 7/1/2025 9:27:35 pm by SYSTEM
                  (modified after loading)
Additional Info : Peak(s) manually integrated
```

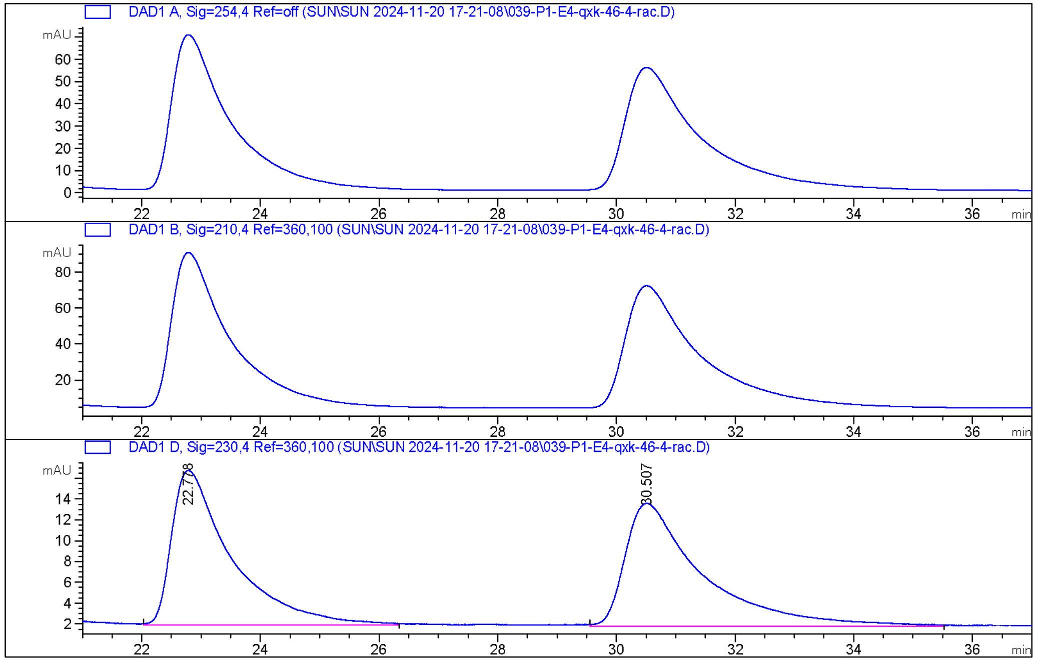

===
Area Percent Report
===

```
Sorted By              :      Signal
Multiplier             :      1.0000
Dilution               :      1.0000
Use Multiplier & Dilution Factor with ISTDs
```


Data File C:\Users\P...Station\1\Data\SUN\SUN 2024-11-20 17-21-08\039-P1-E4-qxk-46-4-rac.D
Sample Name: qxk-46-4-rac

Signal 1: DAD1 A, Sig=254,4 Ref=off

Signal 2: DAD1 B, Sig=210,4 Ref=360,100

Signal 3: DAD1 D, Sig=230,4 Ref=360,100

Peak #	RetTime [min]	Type	Width [min]	Area [mAU*s]	Height [mAU]	Area %
1	22.778	MM R	1.1630	1037.49023	14.86860	49.1476
2	30.507	MM R	1.5111	1073.47656	11.83968	50.8524

Totals : 2110.96680 26.70829

===
*** End of Report ***

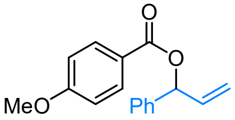

3m-Rac

Data File C:\Users\P...ChemStation\1\Data\SUN\SUN 2025-01-09 15-25-10\006-P1-F4-QXK-88-6.D
Sample Name: QXK-88-6

===

```
Acq. Operator   : SYSTEM                        Seq. Line :   6
Sample Operator : SYSTEM
Acq. Instrument : HPLC                           Location :   P1-F-04
Injection Date  : 9/1/2025 4:53:04 pm                 Inj :   1
                                               Inj Volume : 2.000 µl
Different Inj Volume from Sample Entry!  Actual Inj Volume : 10.000 µl
Acq. Method     : C:\Users\Public\Documents\ChemStation\1\Data\SUN\SUN 2025-01-09 15-25-10\AD3-
                  02-40.M
Last changed    : 11/3/2023 2:01:17 pm by SYSTEM
Analysis Method : C:\Users\Public\Documents\ChemStation\1\Data\SUN\SUN 2025-01-09 15-25-10\AD3-
                  02-40.M (Sequence Method)
Last changed    : 11/1/2025 4:34:28 pm by SYSTEM
                  (modified after loading)
Additional Info : Peak(s) manually integrated
```

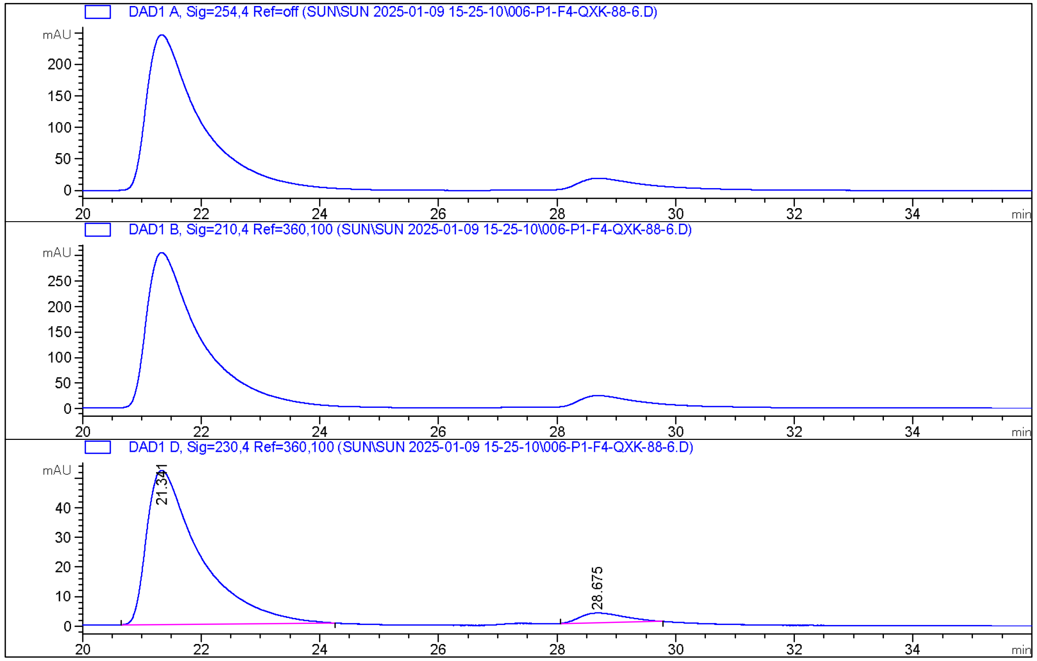

===

Area Percent Report

===

```
Sorted By             :      Signal
Multiplier            :      1.0000
Dilution              :      1.0000
Use Multiplier & Dilution Factor with ISTDs
```


Data File C:\Users\P...ChemStation\1\Data\SUN\SUN 2025-01-09 15-25-10\006-P1-F4-QXK-88-6.D
Sample Name: QXK-88-6

Signal 1: DAD1 A, Sig=254,4 Ref=off

Signal 2: DAD1 B, Sig=210,4 Ref=360,100

Signal 3: DAD1 D, Sig=230,4 Ref=360,100

Peak #	RetTime [min]	Type	Width [min]	Area [mAU*s]	Height [mAU]	Area %
1	21.341	BB	0.7353	3246.96069	52.15977	94.8466
2	28.675	BB	0.6151	176.41959	3.35684	5.1534

Totals : 3423.38028 55.51662

===

*** End of Report ***

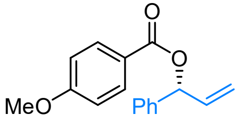

3m

Data File C:\Users\P...tation\1\Data\SUN\SUN 2025-01-06 10-32-44\002-P1-F2-qxk-91-1rac-1.D
Sample Name: qxk-91-1rac-1

===

Acq. Operator : SYSTEM Seq. Line : 2
Sample Operator : SYSTEM
Acq. Instrument : HPLC Location : P1-F-02
Injection Date : 6/1/2025 10:46:08 am Inj : 1
Inj Volume : 2.000 µl
Different Inj Volume from Sample Entry! Actual Inj Volume : 20.000 µl
Acq. Method : C:\Users\Public\Documents\ChemStation\1\Data\SUN\SUN 2025-01-06 10-32-44\AD3-05-30.M
Last changed : 3/9/2022 11:16:06 pm by SYSTEM
Analysis Method : C:\Users\Public\Documents\ChemStation\1\Data\SUN\SUN 2025-01-06 10-32-44\AD3-05-30.M (Sequence Method)
Last changed : 7/1/2025 9:43:54 pm by SYSTEM
(modified after loading)
Additional Info : Peak(s) manually integrated

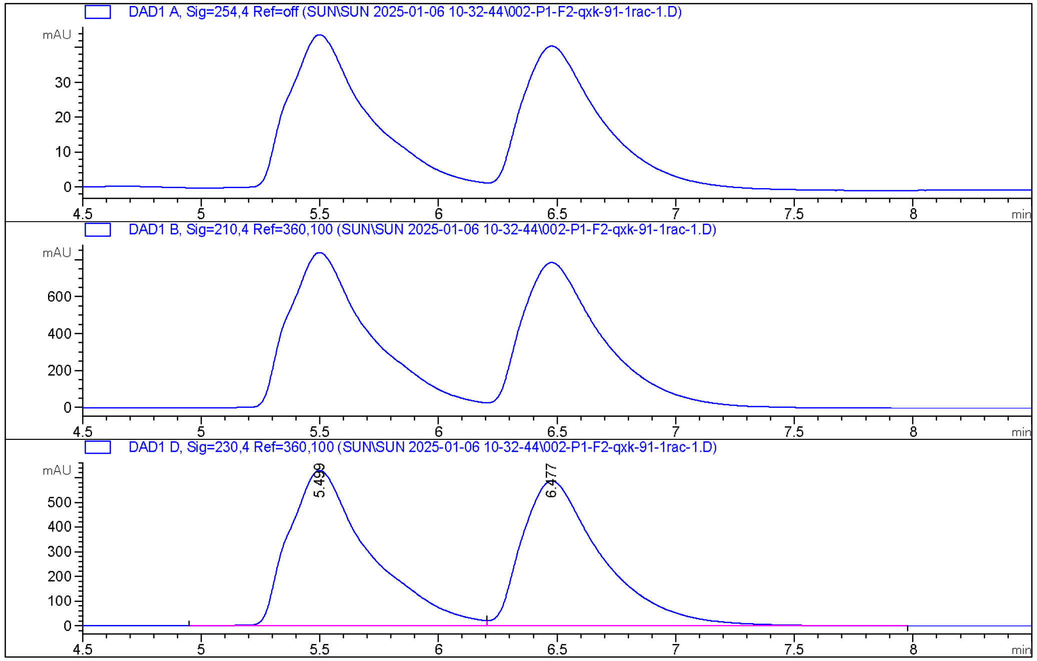

===

Area Percent Report

===

Sorted By : Signal
Multiplier : 1.0000
Dilution : 1.0000
Use Multiplier & Dilution Factor with ISTDs

Signal 1: DAD1 A, Sig=254,4 Ref=off

Signal 2: DAD1 B, Sig=210,4 Ref=360,100

Signal 3: DAD1 D, Sig=230,4 Ref=360,100

Peak #	RetTime [min]	Type	Width [min]	Area [mAU*s]	Height [mAU]	Area %
1	5.499	BV	0.3255	1.48786e4	630.16010	51.8572
2	6.477	VB	0.3501	1.38129e4	587.83191	48.1428

Totals : 2.86914e4 1217.99200

===

*** End of Report ***

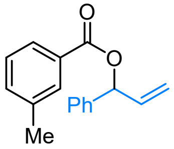

3n-Rac

===

```
Acq. Operator   : SYSTEM                        Seq. Line :   2
Sample Operator : SYSTEM
Acq. Instrument : HPLC                           Location :   P1-F-04
Injection Date  : 15/1/2025 9:32:17 pm                Inj :   1
                                               Inj Volume : 2.000 µl
Different Inj Volume from Sample Entry!  Actual Inj Volume : 20.000 µl
Acq. Method     : C:\Users\Public\Documents\ChemStation\1\Data\SUN\SUN 2025-01-15 21-18-53\AD3-
                  05-30.M
Last changed    : 3/9/2022 11:16:06 pm by SYSTEM
Analysis Method : C:\Users\Public\Documents\ChemStation\1\Data\SUN\SUN 2025-01-15 21-18-53\AD3-
                  05-30.M (Sequence Method)
Last changed    : 16/1/2025 10:07:43 am by SYSTEM
                  (modified after loading)
Additional Info : Peak(s) manually integrated
```

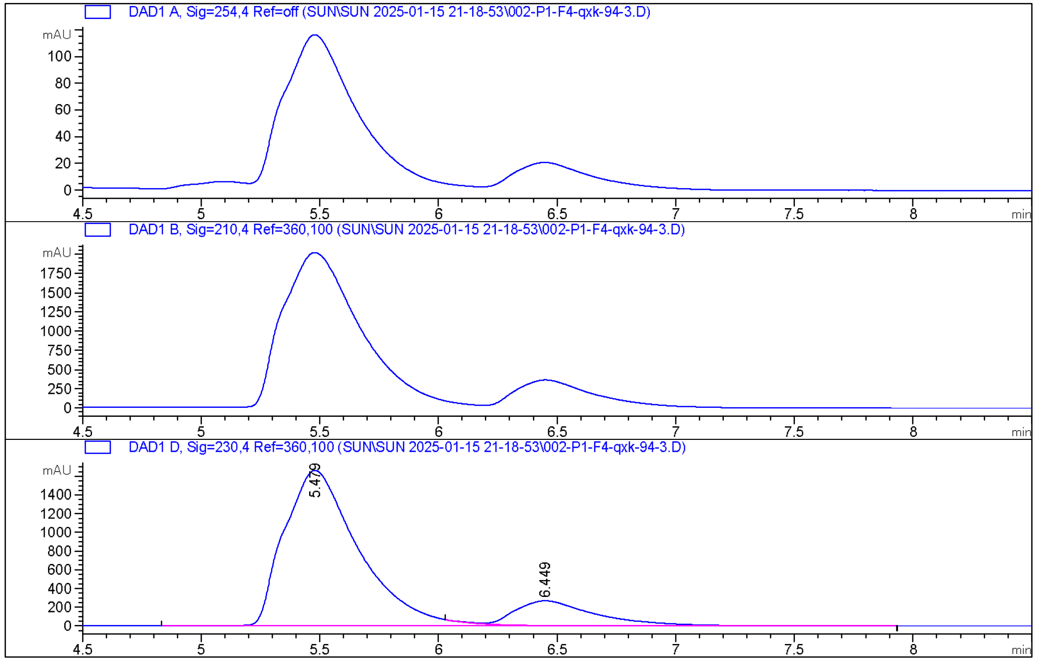

===
Area Percent Report
===

```
Sorted By             :      Signal
Multiplier            :      1.0000
Dilution              :      1.0000
Use Multiplier & Dilution Factor with ISTDs
```


Data File C:\Users\P...ChemStation\1\Data\SUN\SUN 2025-01-15 21-18-53\002-P1-F4-qxk-94-3.D
Sample Name: qxk-94-3

Signal 1: DAD1 A, Sig=254,4 Ref=off

Signal 2: DAD1 B, Sig=210,4 Ref=360,100

Signal 3: DAD1 D, Sig=230,4 Ref=360,100

Peak #	RetTime [min]	Type	Width [min]	Area [mAU*s]	Height [mAU]	Area %
1	5.479	BV R	0.3042	3.63704e4	1662.40723	85.4126
2	6.449	VB E	0.3519	6211.59277	264.08124	14.5874

Totals : 4.25820e4 1926.48846

===
*** End of Report ***

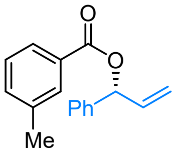

3n

Data File C:\Users\P...Station\1\Data\SUN\SUN 2024-11-15 20-39-14\008-P1-A4-qxk-40-4-rac.D
Sample Name: qxk-40-4-rac

===

```
Acq. Operator   : SYSTEM                        Seq. Line :    8
Sample Operator : SYSTEM
Acq. Instrument : HPLC                           Location :   P1-A-04
Injection Date  : 15/11/2024 10:39:10 pm              Inj :    1
                                               Inj Volume : 2.000 µl
Different Inj Volume from Sample Entry!  Actual Inj Volume : 20.000 µl
Acq. Method     : C:\Users\Public\Documents\ChemStation\1\Data\SUN\SUN 2024-11-15 20-39-14\AD3-
                  01-20.M
Last changed    : 27/7/2023 8:11:05 pm by SYSTEM
Analysis Method : C:\Users\Public\Documents\ChemStation\1\Data\SUN\SUN 2024-11-15 20-39-14\AD3-
                  01-20.M (Sequence Method)
Last changed    : 7/1/2025 9:31:58 pm by SYSTEM
                  (modified after loading)
Additional Info : Peak(s) manually integrated
```

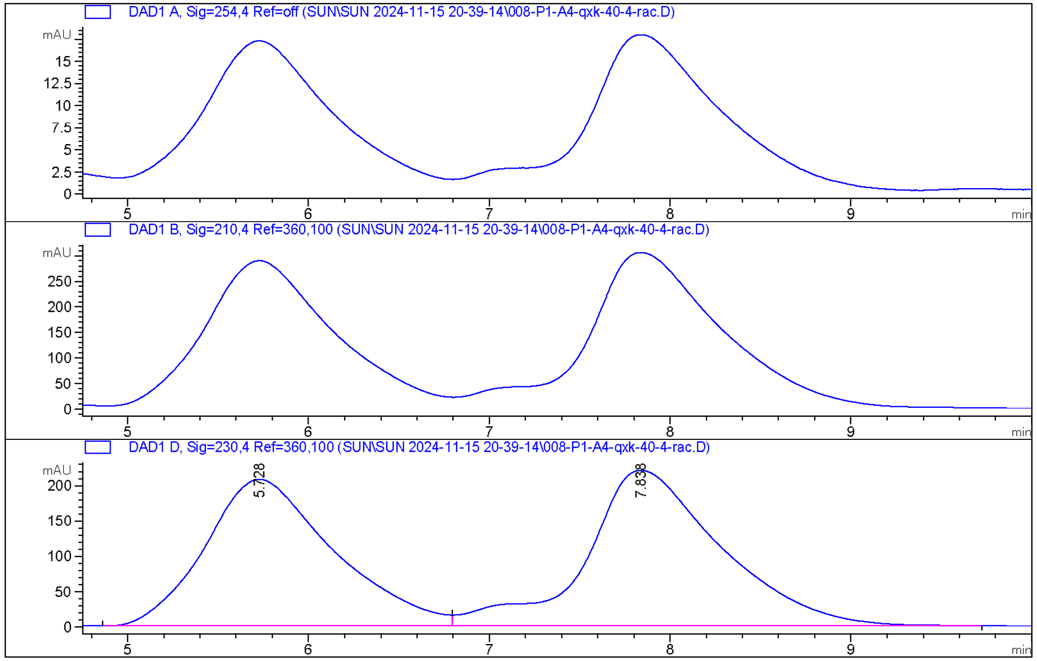

===
Area Percent Report
===

```
Sorted By             :      Signal
Multiplier            :      1.0000
Dilution              :      1.0000
Use Multiplier & Dilution Factor with ISTDs
```


Data File C:\Users\P...Station\1\Data\SUN\SUN 2024-11-15 20-39-14\008-P1-A4-qxk-40-4-rac.D
Sample Name: qxk-40-4-rac

Signal 1: DAD1 A, Sig=254,4 Ref=off

Signal 2: DAD1 B, Sig=210,4 Ref=360,100

Signal 3: DAD1 D, Sig=230,4 Ref=360,100

Peak #	RetTime [min]	Type	Width [min]	Area [mAU*s]	Height [mAU]	Area %
1	5.728	BV	0.6870	1.02309e4	207.41252	47.3072
2	7.838	VB	0.7133	1.13956e4	220.00677	52.6928

Totals : 2.16264e4 427.41930

===
*** End of Report ***

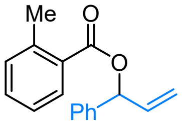

3o-Rac

===

Acq. Operator : SYSTEM Seq. Line : 48
Sample Operator : SYSTEM
Acq. Instrument : HPLC Location : P1-F-02
Injection Date : 7/1/2025 11:23:39 pm Inj : 1
Inj Volume : 2.000 µl
Different Inj Volume from Sample Entry! Actual Inj Volume : 20.000 µl
Acq. Method : C:\Users\Public\Documents\ChemStation\1\Data\SUN\SUN 2025-01-07 09-42-35\AD3-01-10.M
Last changed : 8/10/2022 11:58:49 am by SYSTEM
Analysis Method : C:\Users\Public\Documents\ChemStation\1\Data\SUN\SUN 2025-01-07 09-42-35\AD3-01-10.M (Sequence Method)
Last changed : 9/1/2025 3:07:30 pm by SYSTEM
(modified after loading)
Additional Info : Peak(s) manually integrated

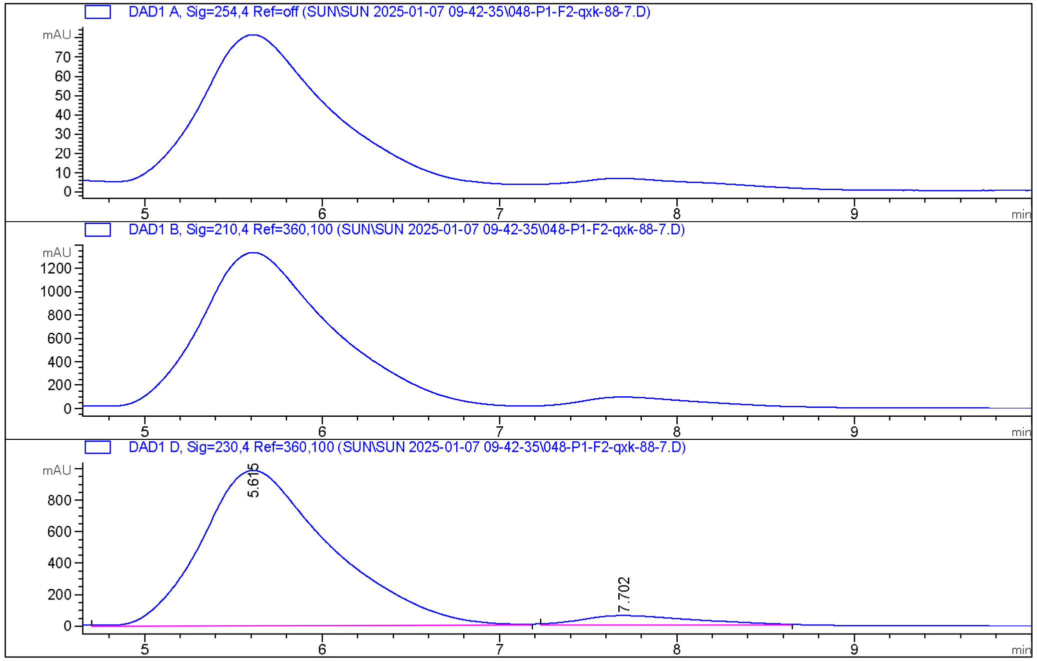

===

Area Percent Report

===

Sorted By : Signal
Multiplier : 1.0000
Dilution : 1.0000
Use Multiplier & Dilution Factor with ISTDs

Data File C:\Users\P...ChemStation\1\Data\SUN\SUN 2025-01-07 09-42-35\048-P1-F2-qxk-88-7.D
Sample Name: qxk-88-7

Signal 1: DAD1 A, Sig=254,4 Ref=off

Signal 2: DAD1 B, Sig=210,4 Ref=360,100

Signal 3: DAD1 D, Sig=230,4 Ref=360,100

Peak #	RetTime [min]	Type	Width [min]	Area [mAU*s]	Height [mAU]	Area %
1	5.615	MM R	0.8474	5.02477e4	988.21741	94.8831
2	7.702	MM R	0.7608	2709.78442	59.36576	5.1169

Totals : 5.29575e4 1047.58316

===

*** End of Report ***

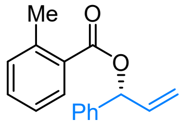

3o

===

```
Acq. Operator   : SYSTEM                        Seq. Line :   6
Sample Operator : SYSTEM
Acq. Instrument : HPLC                           Location :   P1-F-01
Injection Date  : 15/1/2025 11:07:06 pm               Inj :   1
                                               Inj Volume : 2.000 µl
Different Inj Volume from Sample Entry!  Actual Inj Volume : 20.000 µl
Acq. Method     : C:\Users\Public\Documents\ChemStation\1\Data\SUN\SUN 2025-01-15 21-18-53\AD3-
                  05-30.M
Last changed    : 3/9/2022 11:16:06 pm by SYSTEM
Analysis Method : C:\Users\Public\Documents\ChemStation\1\Data\SUN\SUN 2025-01-15 21-18-53\AD3-
                  05-30.M (Sequence Method)
Last changed    : 16/1/2025 10:11:54 am by SYSTEM
                  (modified after loading)
Additional Info : Peak(s) manually integrated
```

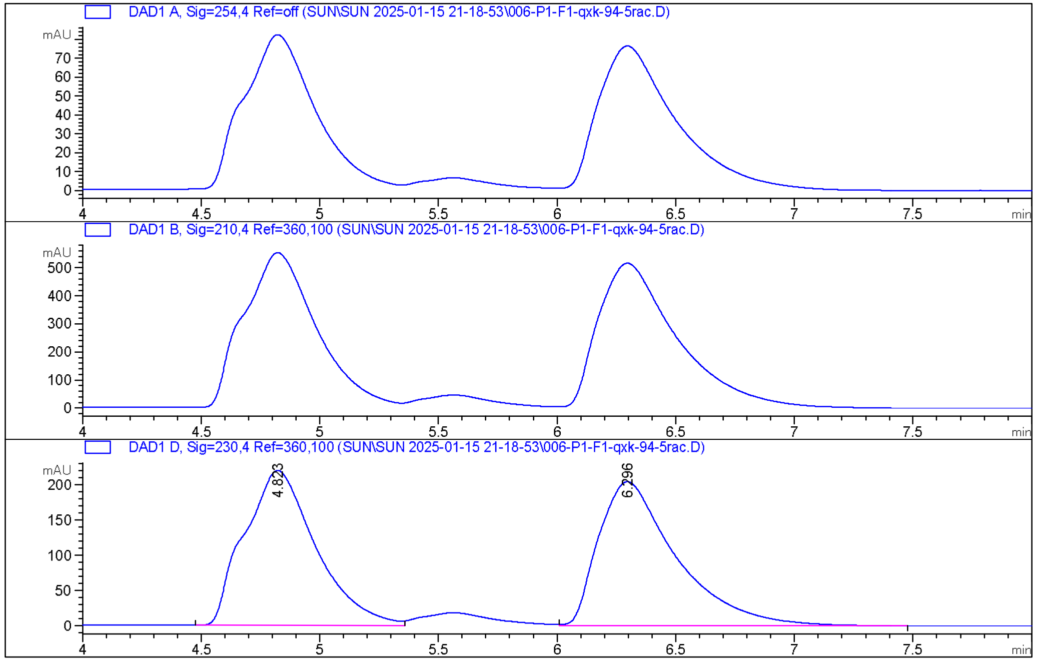

===
Area Percent Report
===

```
Sorted By              :      Signal
Multiplier             :      1.0000
Dilution               :      1.0000
Use Multiplier & Dilution Factor with ISTDs
```


Data File C:\Users\P...mStation\1\Data\SUN\SUN 2025-01-15 21-18-53\006-P1-F1-qxk-94-5rac.D
Sample Name: qxk-94-5rac

Signal 1: DAD1 A, Sig=254,4 Ref=off

Signal 2: DAD1 B, Sig=210,4 Ref=360,100

Signal 3: DAD1 D, Sig=230,4 Ref=360,100

Peak #	RetTime [min]	Type	Width [min]	Area [mAU*s]	Height [mAU]	Area %
1	4.823	MF R	0.3487	4599.62061	219.82562	49.7631
2	6.296	VB	0.3450	4643.42139	204.03688	50.2369
Totals :				9243.04199	423.86250	

===
*** End of Report ***

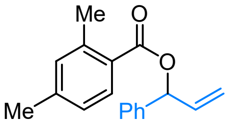

3p-Rac

Data File C:\Users\P...ChemStation\1\Data\SUN\SUN 2025-01-14 21-16-06\017-P1-F9-QXK-94-5.D
Sample Name: QXK-94-5

===

Acq. Operator : SYSTEM Seq. Line : 17
Sample Operator : SYSTEM
Acq. Instrument : HPLC Location : P1-F-09
Injection Date : 15/1/2025 3:17:37 am Inj : 1
Inj Volume : 2.000 µl
Different Inj Volume from Sample Entry! Actual Inj Volume : 20.000 µl
Acq. Method : C:\Users\Public\Documents\ChemStation\1\Data\SUN\SUN 2025-01-14 21-16-06\AD3-05-30.M
Last changed : 3/9/2022 11:16:06 pm by SYSTEM
Analysis Method : C:\Users\Public\Documents\ChemStation\1\Data\SUN\SUN 2025-01-14 21-16-06\AD3-05-30.M (Sequence Method)
Last changed : 16/1/2025 10:46:53 am by SYSTEM
(modified after loading)
Additional Info : Peak(s) manually integrated

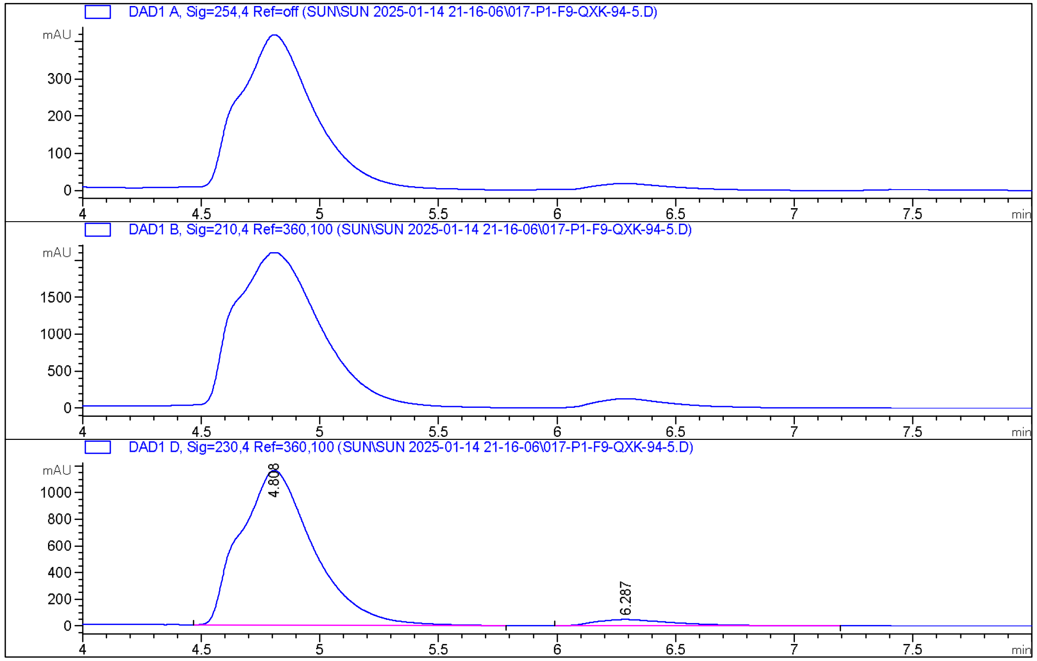

===

Area Percent Report

===

Sorted By : Signal
Multiplier : 1.0000
Dilution : 1.0000
Use Multiplier & Dilution Factor with ISTDs

Data File C:\Users\P...ChemStation\1\Data\SUN\SUN 2025-01-14 21-16-06\017-P1-F9-QXK-94-5.D
Sample Name: QXK-94-5

Signal 1: DAD1 A, Sig=254,4 Ref=off

Signal 2: DAD1 B, Sig=210,4 Ref=360,100

Signal 3: DAD1 D, Sig=230,4 Ref=360,100

Peak #	RetTime [min]	Type	Width [min]	Area [mAU*s]	Height [mAU]	Area %
1	4.808	BB	0.3022	2.47431e4	1160.94312	95.9594
2	6.287	BB	0.2992	1041.86841	46.80670	4.0406
Totals :				2.57850e4	1207.74982	

===

*** End of Report ***

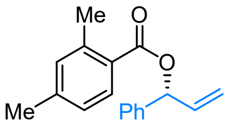

3p

Data File C:\Users\P...mStation\1\Data\SUN\SUN 2024-12-28 10-41-25\020-P1-E3-qxk-83-4rac.D
Sample Name: qxk-83-4rac

===

Acq. Operator : SYSTEM Seq. Line : 20
Sample Operator : SYSTEM
Acq. Instrument : HPLC Location : P1-E-03
Injection Date : 28/12/2024 4:16:08 pm Inj : 1
Inj Volume : 2.000 µl
Different Inj Volume from Sample Entry! Actual Inj Volume : 20.000 µl
Acq. Method : C:\Users\Public\Documents\ChemStation\1\Data\SUN\SUN 2024-12-28 10-41-25\AD3-01-20.M
Last changed : 27/7/2023 8:11:05 pm by SYSTEM
Analysis Method : C:\Users\Public\Documents\ChemStation\1\Data\SUN\SUN 2024-12-28 10-41-25\AD3-01-20.M (Sequence Method)
Last changed : 7/1/2025 9:34:28 pm by SYSTEM
(modified after loading)
Additional Info : Peak(s) manually integrated

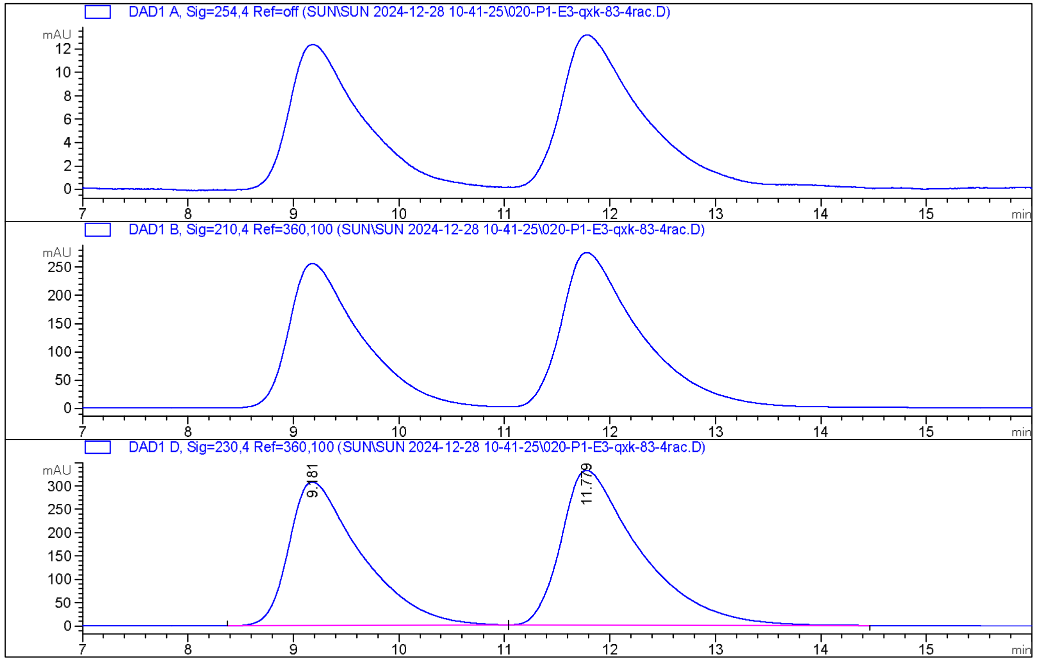

===

Area Percent Report

===

Sorted By : Signal
Multiplier : 1.0000
Dilution : 1.0000
Use Multiplier & Dilution Factor with ISTDs

Data File C:\Users\P...mStation\1\Data\SUN\SUN 2024-12-28 10-41-25\020-P1-E3-qxk-83-4rac.D
Sample Name: qxk-83-4rac

Signal 1: DAD1 A, Sig=254,4 Ref=off

Signal 2: DAD1 B, Sig=210,4 Ref=360,100

Signal 3: DAD1 D, Sig=230,4 Ref=360,100

Peak #	RetTime [min]	Type	Width [min]	Area [mAU*s]	Height [mAU]	Area %
1	9.181	BB	0.6342	1.46046e4	307.72403	45.9455
2	11.779	BB	0.7060	1.71822e4	331.59460	54.0545

Totals : 3.17869e4 639.31863

===

*** End of Report ***

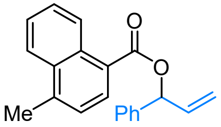

3q-Rac

Data File C:\Users\P...ChemStation\1\Data\SUN\SUN 2025-01-07 09-42-35\049-P1-F3-qxk-88-8.D
Sample Name: qxk-88-8

===

Acq. Operator : SYSTEM Seq. Line : 49
Sample Operator : SYSTEM
Acq. Instrument : HPLC Location : P1-F-03
Injection Date : 7/1/2025 11:34:53 pm Inj : 1
Inj Volume : 2.000 µl
Different Inj Volume from Sample Entry! Actual Inj Volume : 20.000 µl
Acq. Method : C:\Users\Public\Documents\ChemStation\1\Data\SUN\SUN 2025-01-07 09-42-35\AD3-01-20.M
Last changed : 27/7/2023 8:11:05 pm by SYSTEM
Analysis Method : C:\Users\Public\Documents\ChemStation\1\Data\SUN\SUN 2025-01-07 09-42-35\AD3-01-20.M (Sequence Method)
Last changed : 9/1/2025 3:09:19 pm by SYSTEM
(modified after loading)
Additional Info : Peak(s) manually integrated

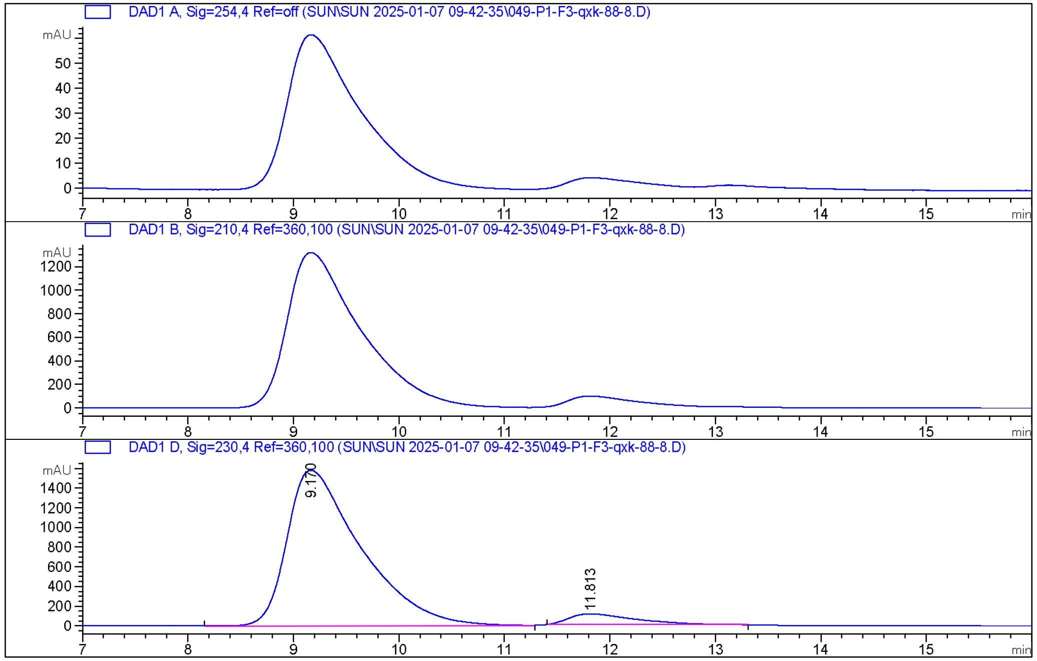

===

Area Percent Report

===

Sorted By : Signal
Multiplier : 1.0000
Dilution : 1.0000
Use Multiplier & Dilution Factor with ISTDs

Data File C:\Users\P...ChemStation\1\Data\SUN\SUN 2025-01-07 09-42-35\049-P1-F3-qxk-88-8.D
Sample Name: qxk-88-8

Signal 1: DAD1 A, Sig=254,4 Ref=off

Signal 2: DAD1 B, Sig=210,4 Ref=360,100

Signal 3: DAD1 D, Sig=230,4 Ref=360,100

Peak #	RetTime [min]	Type	Width [min]	Area [mAU*s]	Height [mAU]	Area %
1	9.170	MM R	0.8163	7.75912e4	1584.17358	94.2791
2	11.813	MM R	0.7205	4708.23486	108.91082	5.7209

Totals : 8.22994e4 1693.08440

===

*** End of Report ***

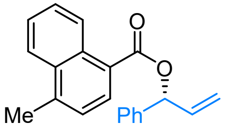

3q

===

Acq. Operator : SYSTEM Seq. Line : 3
Sample Operator : SYSTEM
Acq. Instrument : HPLC Location : P1-F-02
Injection Date : 12/5/2025 9:45:27 pm Inj : 1
Inj Volume : 2.000 µl
Different Inj Volume from Sample Entry! Actual Inj Volume : 20.000 µl
Acq. Method : C:\Users\Public\Documents\ChemStation\1\Data\SUN\SUN 2025-05-12 21-00-53\ID3-10-30.M
Last changed : 16/8/2022 10:05:02 am by SYSTEM
Analysis Method : C:\Users\Public\Documents\ChemStation\1\Data\SUN\SUN 2025-05-12 21-00-53\ID3-10-30.M (Sequence Method)
Last changed : 17/5/2025 4:07:52 pm by SYSTEM
(modified after loading)
Additional Info : Peak(s) manually integrated

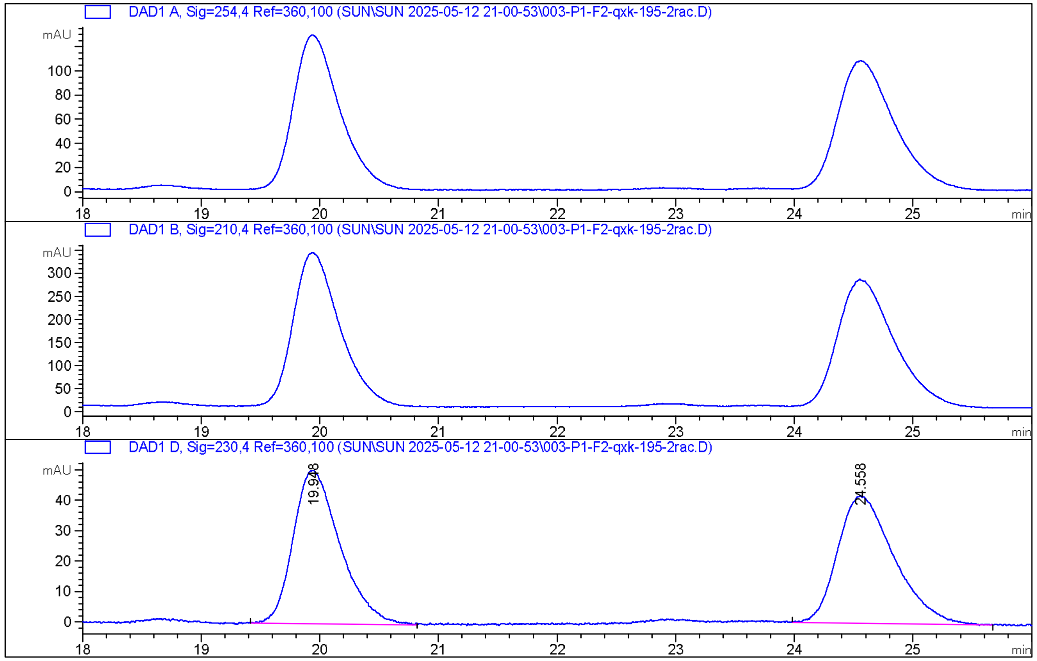

===

Area Percent Report

===

Sorted By : Signal
Multiplier : 1.0000
Dilution : 1.0000
Use Multiplier & Dilution Factor with ISTDs

Data File C:\Users\P...Station\1\Data\SUN\SUN 2025-05-12 21-00-53\003-P1-F2-qxk-195-2rac.D
Sample Name: qxk-195-2rac

Signal 1: DAD1 A, Sig=254,4 Ref=360,100

Signal 2: DAD1 B, Sig=210,4 Ref=360,100

Signal 3: DAD1 D, Sig=230,4 Ref=360,100

Peak #	RetTime [min]	Type	Width [min]	Area [mAU*s]	Height [mAU]	Area %
1	19.948	MM R	0.4581	1389.83813	50.56574	50.1129
2	24.558	MM R	0.5498	1383.57666	41.94281	49.8871
Totals :				2773.41479	92.50855	

===

*** End of Report ***

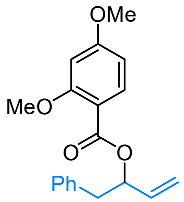

3r-Rac

Data File C:\Users\P...hemStation\1\Data\SUN\SUN 2025-05-16 18-38-04\015-P1-E2-qxk-195-2.D
Sample Name: qxk-195-2

===

Acq. Operator : SYSTEM Seq. Line : 15
Sample Operator : SYSTEM
Acq. Instrument : HPLC Location : P1-E-02
Injection Date : 16/5/2025 10:26:13 pm Inj : 1
Inj Volume : 2.000 µl
Different Inj Volume from Sample Entry! Actual Inj Volume : 20.000 µl
Acq. Method : C:\Users\Public\Documents\ChemStation\1\Data\SUN\SUN 2025-05-16 18-38-04\ID3-10-30.M
Last changed : 16/8/2022 10:05:02 am by SYSTEM
Analysis Method : C:\Users\Public\Documents\ChemStation\1\Data\SUN\SUN 2025-05-16 18-38-04\ID3-10-30.M (Sequence Method)
Last changed : 17/5/2025 4:05:30 pm by SYSTEM
(modified after loading)
Additional Info : Peak(s) manually integrated

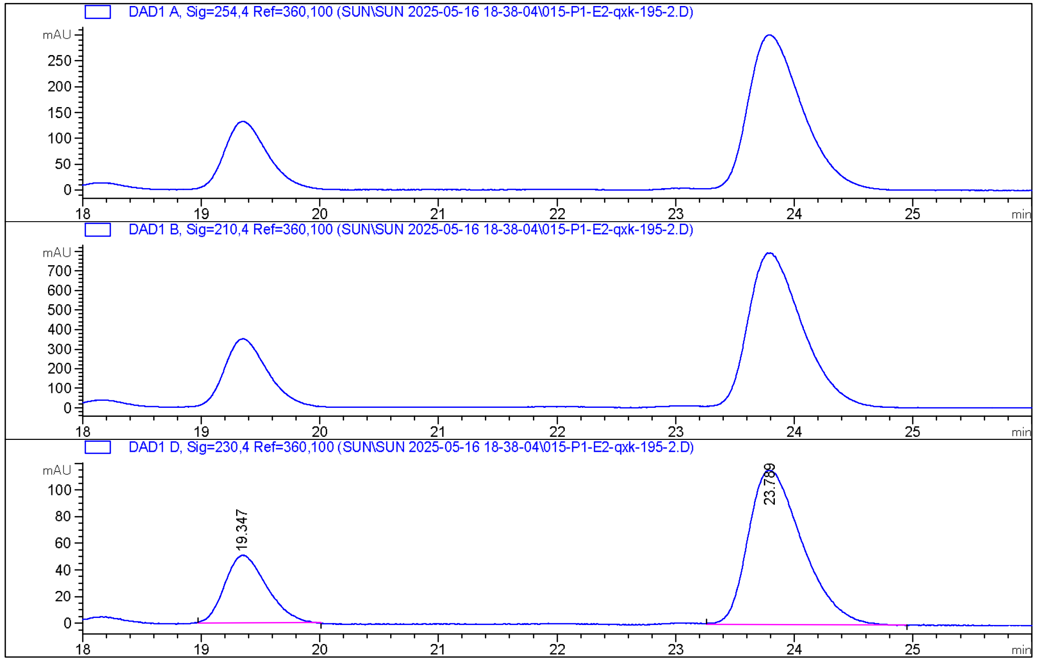

===

Area Percent Report

===

Sorted By : Signal
Multiplier : 1.0000
Dilution : 1.0000
Use Multiplier & Dilution Factor with ISTDs

Data File C:\Users\P...hemStation\1\Data\SUN\SUN 2025-05-16 18-38-04\015-P1-E2-qxk-195-2.D
Sample Name: qxk-195-2

Signal 1: DAD1 A, Sig=254,4 Ref=360,100

Signal 2: DAD1 B, Sig=210,4 Ref=360,100

Signal 3: DAD1 D, Sig=230,4 Ref=360,100

Peak #	RetTime [min]	Type	Width [min]	Area [mAU*s]	Height [mAU]	Area %
1	19.347	MM R	0.4080	1245.73022	50.88183	25.3016
2	23.789	MM R	0.5286	3677.78687	115.95383	74.6984

Totals : 4923.51709 166.83567

===

*** End of Report ***

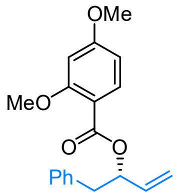

3r

===

Acq. Operator : SYSTEM Seq. Line : 2
Sample Operator : SYSTEM
Acq. Instrument : HPLC Location : P1-F-01
Injection Date : 12/5/2025 9:14:14 pm Inj : 1
Inj Volume : 2.000 µl
Different Inj Volume from Sample Entry! Actual Inj Volume : 20.000 µl
Acq. Method : C:\Users\Public\Documents\ChemStation\1\Data\SUN\SUN 2025-05-12 21-00-53\IC3-10-30.M
Last changed : 19/8/2022 10:41:24 pm by SYSTEM
Analysis Method : C:\Users\Public\Documents\ChemStation\1\Data\SUN\SUN 2025-05-12 21-00-53\IC3-10-30.M (Sequence Method)
Last changed : 16/5/2025 9:00:34 pm by SYSTEM
(modified after loading)
Additional Info : Peak(s) manually integrated

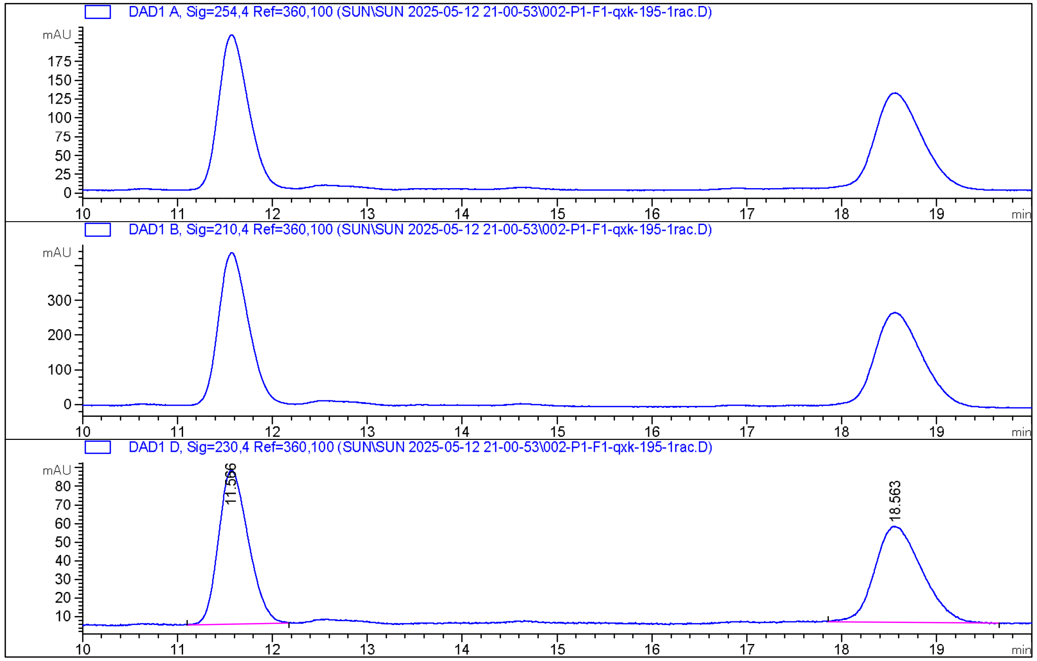

===
Area Percent Report
===

Sorted By : Signal
Multiplier : 1.0000
Dilution : 1.0000
Use Multiplier & Dilution Factor with ISTDs

Data File C:\Users\P...Station\1\Data\SUN\SUN 2025-05-12 21-00-53\002-P1-F1-qxk-195-1rac.D
Sample Name: qxk-195-1rac

Signal 1: DAD1 A, Sig=254,4 Ref=360,100

Signal 2: DAD1 B, Sig=210,4 Ref=360,100

Signal 3: DAD1 D, Sig=230,4 Ref=360,100

Peak #	RetTime [min]	Type	Width [min]	Area [mAU*s]	Height [mAU]	Area %
1	11.566	VV R	0.2590	1806.67639	82.64378	50.2729
2	18.563	MM R	0.5773	1787.06445	51.59245	49.7271
Totals :				3593.74084	134.23624	

===
*** End of Report ***

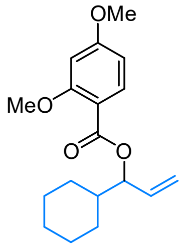

3s-Rac

===

```
Acq. Operator   : SYSTEM                        Seq. Line :    4
Sample Operator : SYSTEM
Acq. Instrument : HPLC                           Location :    P1-E-01
Injection Date  : 15/5/2025 3:12:14 pm                Inj :    1
                                               Inj Volume : 2.000 µl
Different Inj Volume from Sample Entry!  Actual Inj Volume : 10.000 µl
Acq. Method     : C:\Users\Public\Documents\ChemStation\1\Data\SUN\SUN 2025-05-15 14-17-27\IC3-
                  10-30.M
Last changed    : 19/8/2022 10:41:24 pm by SYSTEM
Analysis Method : C:\Users\Public\Documents\ChemStation\1\Data\SUN\SUN 2025-05-15 14-17-27\IC3-
                  10-30.M (Sequence Method)
Last changed    : 16/5/2025 9:05:38 pm by SYSTEM
                  (modified after loading)
Additional Info : Peak(s) manually integrated
```

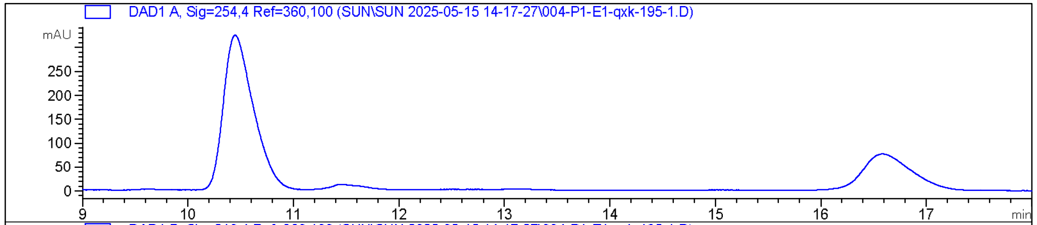

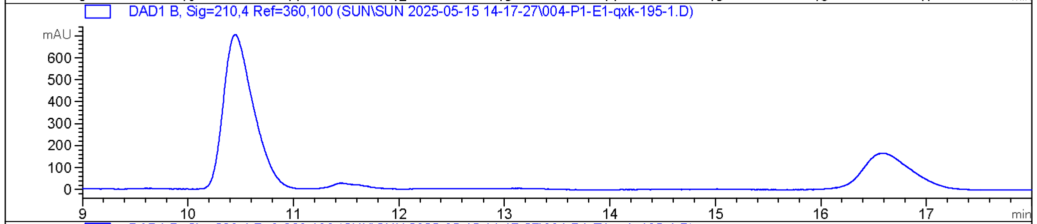

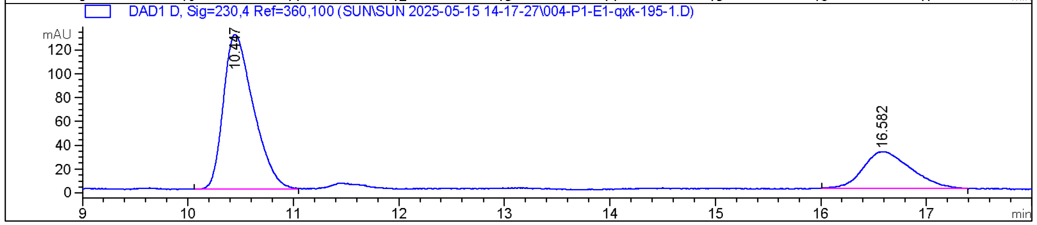

===

Area Percent Report

===

```
Sorted By             :      Signal
Multiplier            :      1.0000
Dilution              :      1.0000
Use Multiplier & Dilution Factor with ISTDs
```


Data File C:\Users\P...hemStation\1\Data\SUN\SUN 2025-05-15 14-17-27\004-P1-E1-qxk-195-1.D
Sample Name: qxk-195-1

Signal 1: DAD1 A, Sig=254,4 Ref=360,100

Signal 2: DAD1 B, Sig=210,4 Ref=360,100

Signal 3: DAD1 D, Sig=230,4 Ref=360,100

Peak #	RetTime [min]	Type	Width [min]	Area [mAU*s]	Height [mAU]	Area %
1	10.447	VV R	0.2362	2568.96704	129.90587	72.5549
2	16.582	MM R	0.5255	971.75378	30.82261	27.4451

Totals : 3540.72083 160.72848

===

*** End of Report ***

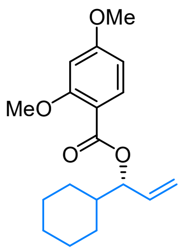

3s

```
=====================================================================
Acq. Operator   : SYSTEM                        Seq. Line :  18
                                                 Location :   P1-F-02
Injection Date  : 2/14/2025 5:33:51 PM                Inj :   1
Acq. Method     : IC3-20-30.M
Analysis Method : C:\Users\Public\Documents\ChemStation\1\Data\SUN\SUN 2025-02-14 13-13-28
                  \IC3-20-30.M (Sequence Method)
Last changed    : 2/28/2025 3:56:12 PM by SYSTEM
                  (modified after loading)
Additional Info : Peak(s) manually integrated
```

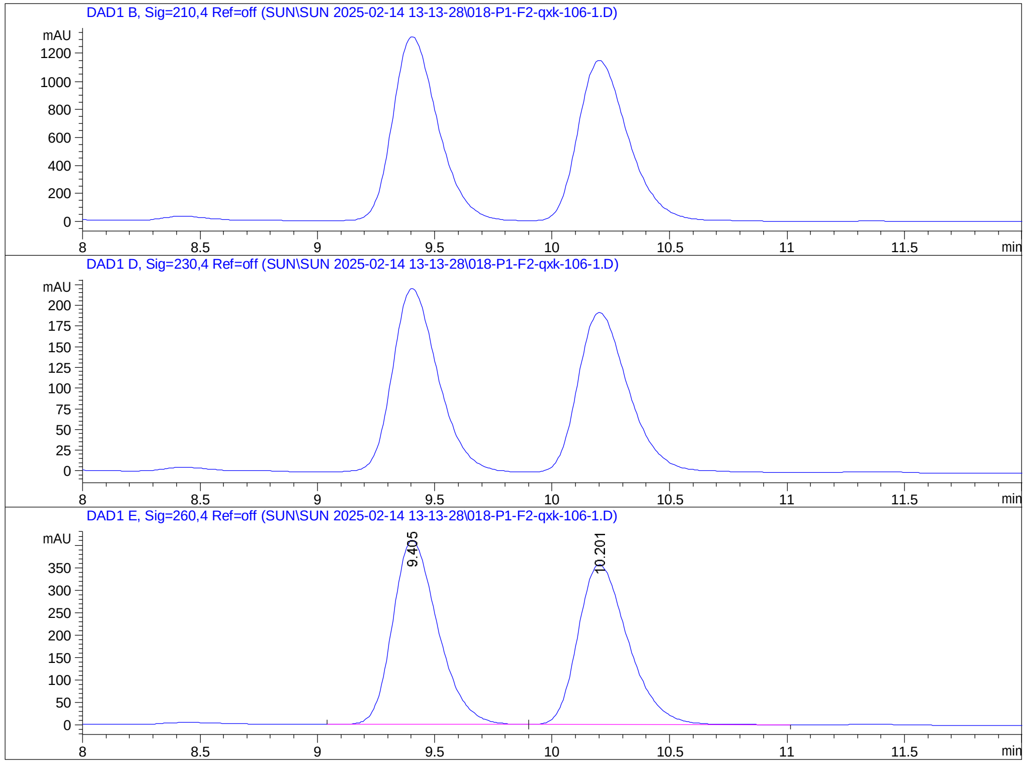


```
=====================================================================
                         Area Percent Report
=====================================================================

Sorted By             :      Signal
Multiplier            :      1.0000
Dilution              :      1.0000
Use Multiplier & Dilution Factor with ISTDs
```


Data File C:\Users\P...hemStation\1\Data\SUN\SUN 2025-02-14 13-13-28\018-P1-F2-qxk-106-1.D
Sample Name: qxk-106-1

Signal 1: DAD1 B, Sig=210,4 Ref=off

Signal 2: DAD1 D, Sig=230,4 Ref=off

Signal 3: DAD1 E, Sig=260,4 Ref=off

Peak #	RetTime [min]	Type	Width [min]	Area [mAU*s]	Height [mAU]	Area %
1	9.405	BB	0.2068	5503.69482	409.53000	50.9781
2	10.201	BB	0.2291	5292.50635	356.60925	49.0219

Totals : 1.07962e4 766.13925

```
=====================================================================
                          *** End of Report ***
```

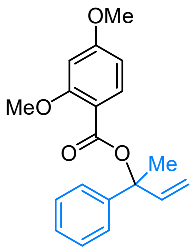

3t-Rac

===

```
Acq. Operator   : SYSTEM                        Seq. Line :    2
Acq. Instrument : LC1260                         Location :   P1-F-01
Injection Date  : 1/23/2025 2:57:34 PM                Inj :    1
                                               Inj Volume : 5.000 µl
Different Inj Volume from Sample Entry!  Actual Inj Volume : 20.000 µl
Acq. Method     : C:\Users\Public\Documents\ChemStation\1\Data\SUN\SUN 2025-01-23 14-44-42
                  \IC3-20-30.M
Last changed    : 4/1/2024 7:53:22 PM by SYSTEM
Analysis Method : C:\Users\Public\Documents\ChemStation\1\Data\SUN\SUN 2025-01-23 14-44-42
                  \IC3-20-30.M (Sequence Method)
Last changed    : 1/24/2025 12:03:38 PM by SYSTEM
                  (modified after loading)
Additional Info : Peak(s) manually integrated
```

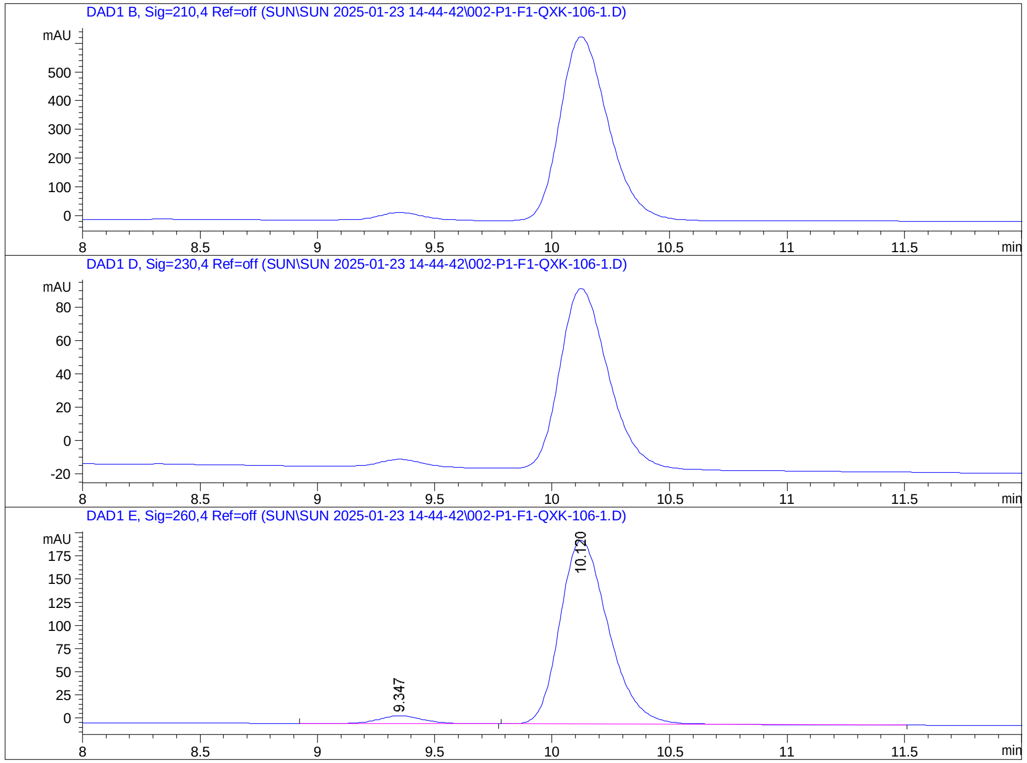

===
Area Percent Report
===

```
Sorted By             :      Signal
Multiplier            :      1.0000
Dilution              :      1.0000
Use Multiplier & Dilution Factor with ISTDs
```


Data File C:\Users\P...hemStation\1\Data\SUN\SUN 2025-01-23 14-44-42\002-P1-F1-QXK-106-1.D
Sample Name: QXK-106-1

Signal 1: DAD1 B, Sig=210,4 Ref=off

Signal 2: DAD1 D, Sig=230,4 Ref=off

Signal 3: DAD1 E, Sig=260,4 Ref=off

Peak #	RetTime [min]	Type	Width [min]	Area [mAU*s]	Height [mAU]	Area %
1	9.347	BB	0.2050	114.79300	8.53168	3.8877
2	10.120	BB	0.2213	2837.91260	197.95398	96.1123

Totals : 2952.70560 206.48565

===
*** End of Report ***

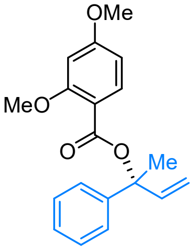

3t

===

Acq. Operator : SYSTEM Seq. Line : 10
Sample Operator : SYSTEM
Acq. Instrument : HPLC Location : P1-F-05
Injection Date : 14/2/2025 6:13:21 pm Inj : 1
Inj Volume : 2.000 µl
Different Inj Volume from Sample Entry! Actual Inj Volume : 20.000 µl
Acq. Method : C:\Users\Public\Documents\ChemStation\1\Data\SUN\SUN 2025-02-14 15-47-48\AD3-20-20.M
Last changed : 20/12/2024 6:43:38 pm by SYSTEM
Analysis Method : C:\Users\Public\Documents\ChemStation\1\Data\SUN\SUN 2025-02-14 15-47-48\AD3-20-20.M (Sequence Method)
Last changed : 28/2/2025 4:18:24 pm by SYSTEM
(modified after loading)
Additional Info : Peak(s) manually integrated

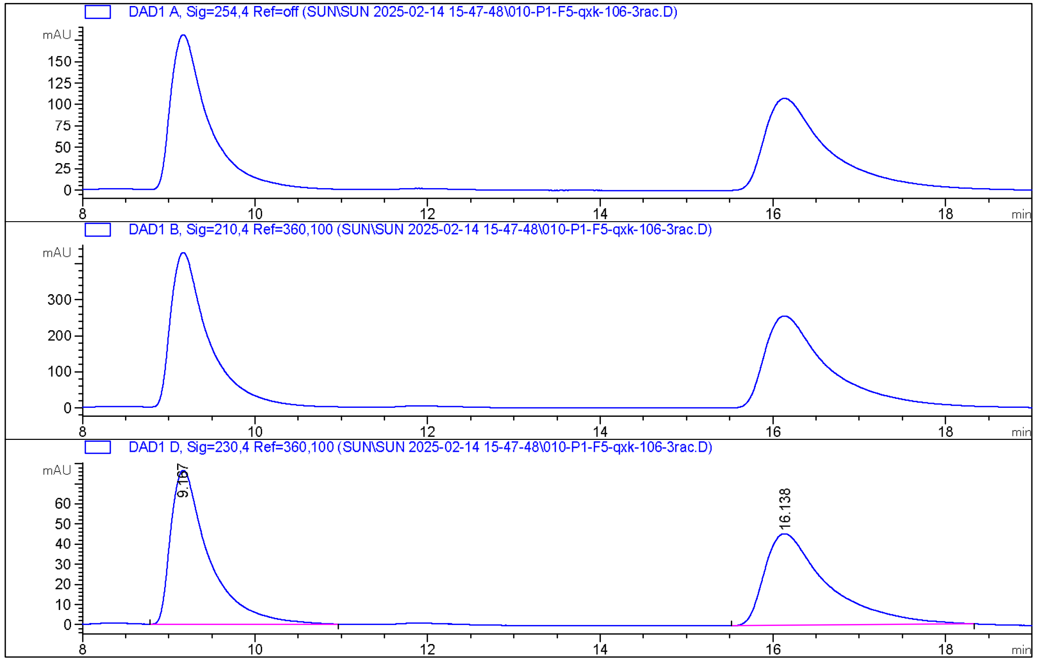

===

Area Percent Report

===

Sorted By : Signal
Multiplier : 1.0000
Dilution : 1.0000
Use Multiplier & Dilution Factor with ISTDs

Data File C:\Users\P...Station\1\Data\SUN\SUN 2025-02-14 15-47-48\010-P1-F5-qxk-106-3rac.D
Sample Name: qxk-106-3rac

Signal 1: DAD1 A, Sig=254,4 Ref=off

Signal 2: DAD1 B, Sig=210,4 Ref=360,100

Signal 3: DAD1 D, Sig=230,4 Ref=360,100

Peak #	RetTime [min]	Type	Width [min]	Area [mAU*s]	Height [mAU]	Area %
1	9.167	BB	0.4301	2355.71118	76.63108	50.4736
2	16.138	BB	0.5945	2311.50513	45.62934	49.5264
Totals :				4667.21631	122.26042	

===

*** End of Report ***

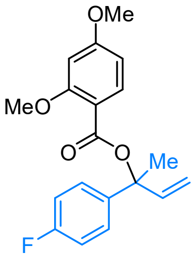

3u-Rac

Data File C:\Users\P...hemStation\1\Data\SUN\SUN 2025-01-23 14-16-33\010-P1-E2-qxk-106-3.D
Sample Name: qxk-106-3

===

Acq. Operator : SYSTEM   Seq. Line : 10
Sample Operator : SYSTEM
Acq. Instrument : HPLC   Location : P1-E-02
Injection Date : 23/1/2025 5:19:16 pm   Inj : 1
Inj Volume : 2.000 µl
Different Inj Volume from Sample Entry! Actual Inj Volume : 20.000 µl
Acq. Method : C:\Users\Public\Documents\ChemStation\1\Data\SUN\SUN 2025-01-23 14-16-33\AD3-20-30.M
Last changed : 18/8/2022 9:21:31 pm by SYSTEM
Analysis Method : C:\Users\Public\Documents\ChemStation\1\Data\SUN\SUN 2025-01-23 14-16-33\AD3-20-30.M (Sequence Method)
Last changed : 28/2/2025 4:22:17 pm by SYSTEM
(modified after loading)
Additional Info : Peak(s) manually integrated

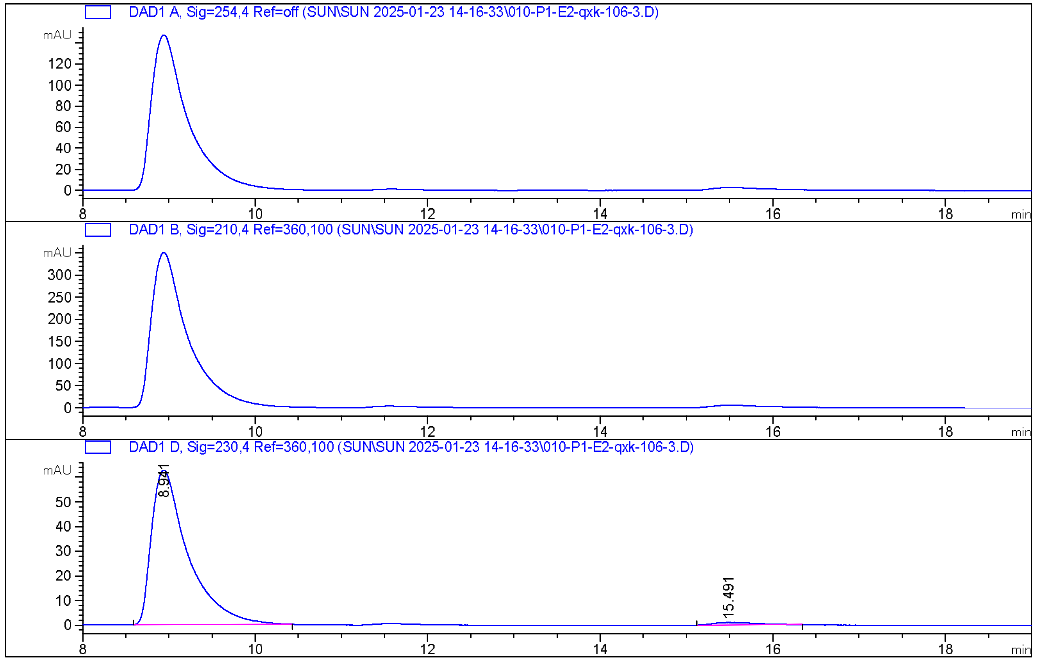

===

Area Percent Report

===

Sorted By : Signal
Multiplier : 1.0000
Dilution : 1.0000
Use Multiplier & Dilution Factor with ISTDs

Data File C:\Users\P...hemStation\1\Data\SUN\SUN 2025-01-23 14-16-33\010-P1-E2-qxk-106-3.D
Sample Name: qxk-106-3

Signal 1: DAD1 A, Sig=254,4 Ref=off

Signal 2: DAD1 B, Sig=210,4 Ref=360,100

Signal 3: DAD1 D, Sig=230,4 Ref=360,100

Peak #	RetTime [min]	Type	Width [min]	Area [mAU*s]	Height [mAU]	Area %
1	8.941	BB	0.4145	1848.00452	62.75930	98.1116
2	15.491	MM R	0.5622	35.57013	1.05442	1.8884

Totals : 1883.57465 63.81372

===

*** End of Report ***

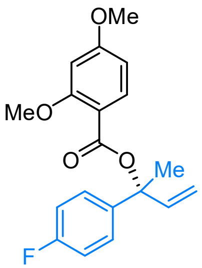

3u

===

Acq. Operator : SYSTEM Seq. Line : 8
Acq. Instrument : LC1260 Location : P1-F-07
Injection Date : 1/13/2025 2:13:41 PM Inj : 1
Inj Volume : 5.000 µl
Different Inj Volume from Sample Entry! Actual Inj Volume : 20.000 µl
Acq. Method : C:\Users\Public\Documents\ChemStation\1\Data\SUN\SUN 2025-01-13 10-53-31\IC3-20-30.M
Last changed : 4/1/2024 7:53:22 PM by SYSTEM
Analysis Method : C:\Users\Public\Documents\ChemStation\1\Data\SUN\SUN 2025-01-13 10-53-31\IC3-20-30.M (Sequence Method)
Last changed : 2/28/2025 3:58:40 PM by SYSTEM
(modified after loading)
Additional Info : Peak(s) manually integrated

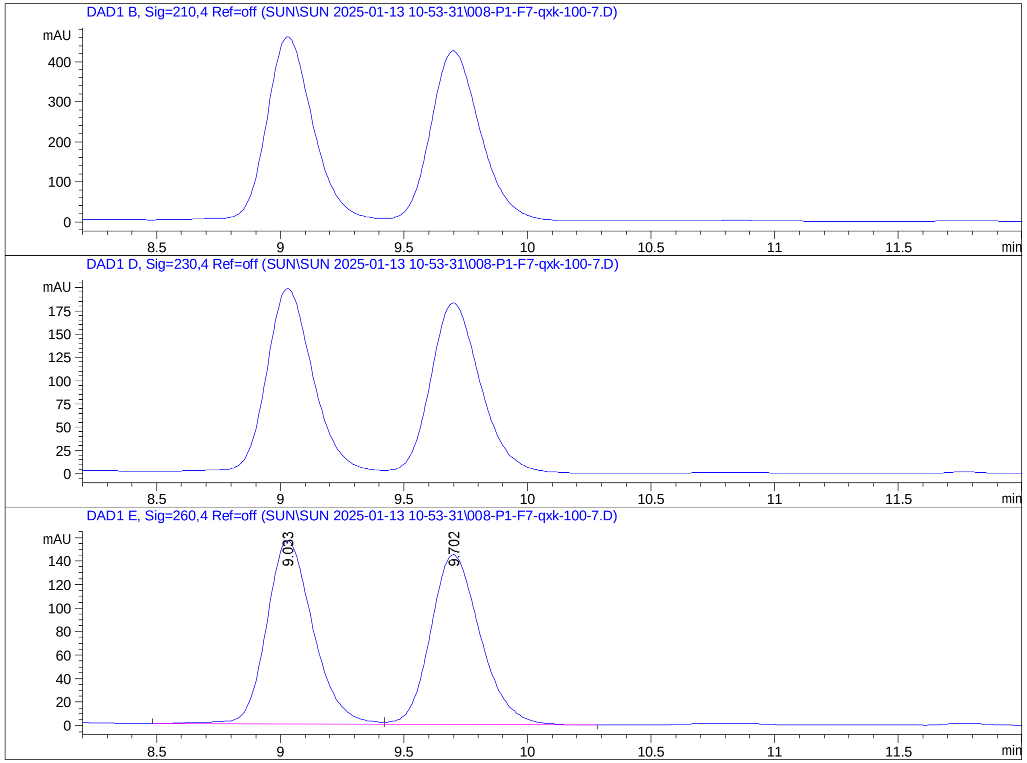

===

Area Percent Report

===

Sorted By : Signal
Multiplier : 1.0000
Dilution : 1.0000
Use Multiplier & Dilution Factor with ISTDs

Data File C:\Users\P...hemStation\1\Data\SUN\SUN 2025-01-13 10-53-31\008-P1-F7-qxk-100-7.D
Sample Name: qxk-100-7

Signal 1: DAD1 B, Sig=210,4 Ref=off

Signal 2: DAD1 D, Sig=230,4 Ref=off

Signal 3: DAD1 E, Sig=260,4 Ref=off

Peak #	RetTime [min]	Type	Width [min]	Area [mAU*s]	Height [mAU]	Area %
1	9.033	BV	0.2004	2039.50989	156.16853	50.3393
2	9.702	VB	0.2144	2012.01721	144.55605	49.6607

Totals : 4051.52710 300.72458

===

*** End of Report ***

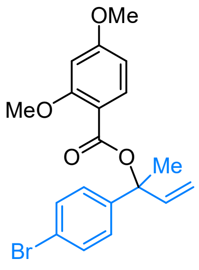

3v-Rac

Data File C:\Users\P...hemStation\1\Data\SUN\SUN 2025-01-23 14-44-42\003-P1-F2-QXK-106-4.D
Sample Name: QXK-106-4

===

Acq. Operator : SYSTEM Seq. Line : 3
Acq. Instrument : LC1260 Location : P1-F-02
Injection Date : 1/23/2025 3:28:42 PM Inj : 1
Inj Volume : 5.000 µl
Different Inj Volume from Sample Entry! Actual Inj Volume : 20.000 µl
Acq. Method : C:\Users\Public\Documents\ChemStation\1\Data\SUN\SUN 2025-01-23 14-44-42\IC3-20-30.M
Last changed : 4/1/2024 7:53:22 PM by SYSTEM
Analysis Method : C:\Users\Public\Documents\ChemStation\1\Data\SUN\SUN 2025-01-23 14-44-42\IC3-20-30.M (Sequence Method)
Last changed : 1/24/2025 12:05:09 PM by SYSTEM
(modified after loading)
Additional Info : Peak(s) manually integrated

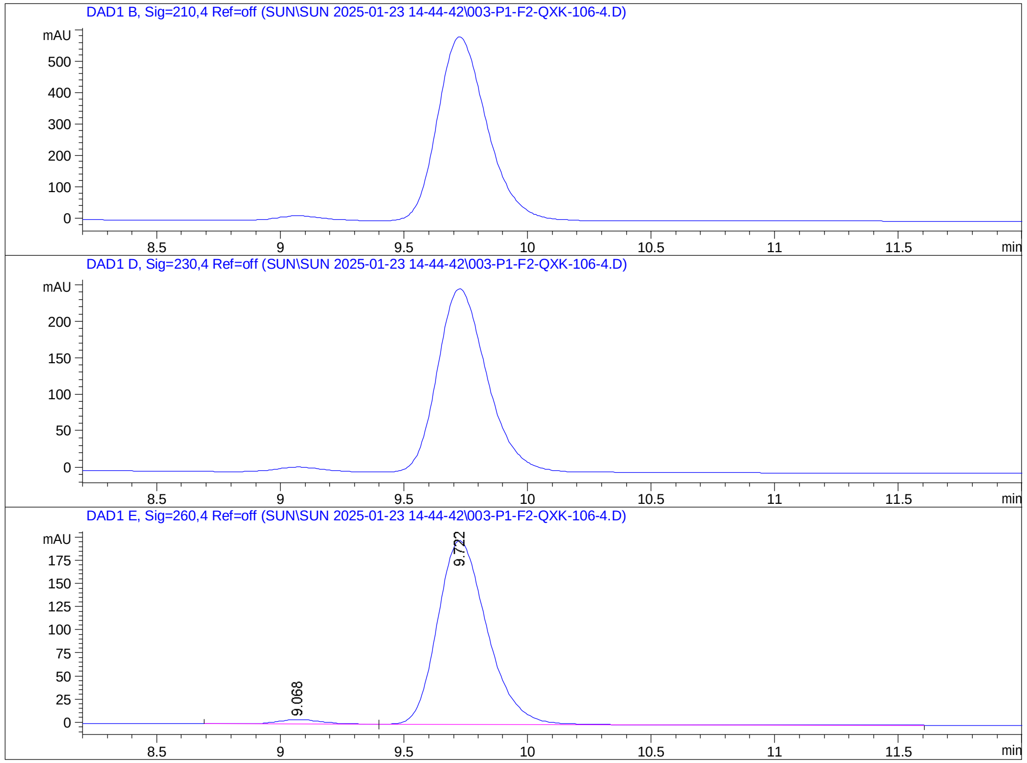

===
Area Percent Report
===

Sorted By : Signal
Multiplier : 1.0000
Dilution : 1.0000
Use Multiplier & Dilution Factor with ISTDs

Data File C:\Users\P...hemStation\1\Data\SUN\SUN 2025-01-23 14-44-42\003-P1-F2-QXK-106-4.D
Sample Name: QXK-106-4

Signal 1: DAD1 B, Sig=210,4 Ref=off

Signal 2: DAD1 D, Sig=230,4 Ref=off

Signal 3: DAD1 E, Sig=260,4 Ref=off

Peak #	RetTime [min]	Type	Width [min]	Area [mAU*s]	Height [mAU]	Area %
1	9.068	BB	0.1978	63.41973	4.93846	2.2448
2	9.722	BB	0.2140	2761.74878	198.85391	97.7552

Totals : 2825.16851 203.79237

===
*** End of Report ***

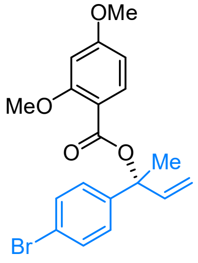

3v

===

Acq. Operator : SYSTEM Seq. Line : 4
Sample Operator : SYSTEM
Acq. Instrument : HPLC Location : P1-F-03
Injection Date : 12/5/2025 10:17:09 pm Inj : 1
Inj Volume : 2.000 µl
Different Inj Volume from Sample Entry! Actual Inj Volume : 20.000 µl
Acq. Method : C:\Users\Public\Documents\ChemStation\1\Data\SUN\SUN 2025-05-12 21-00-53\IC3-10-30.M
Last changed : 19/8/2022 10:41:24 pm by SYSTEM
Analysis Method : C:\Users\Public\Documents\ChemStation\1\Data\SUN\SUN 2025-05-12 21-00-53\IC3-10-30.M (Sequence Method)
Last changed : 16/5/2025 9:03:04 pm by SYSTEM
(modified after loading)
Additional Info : Peak(s) manually integrated

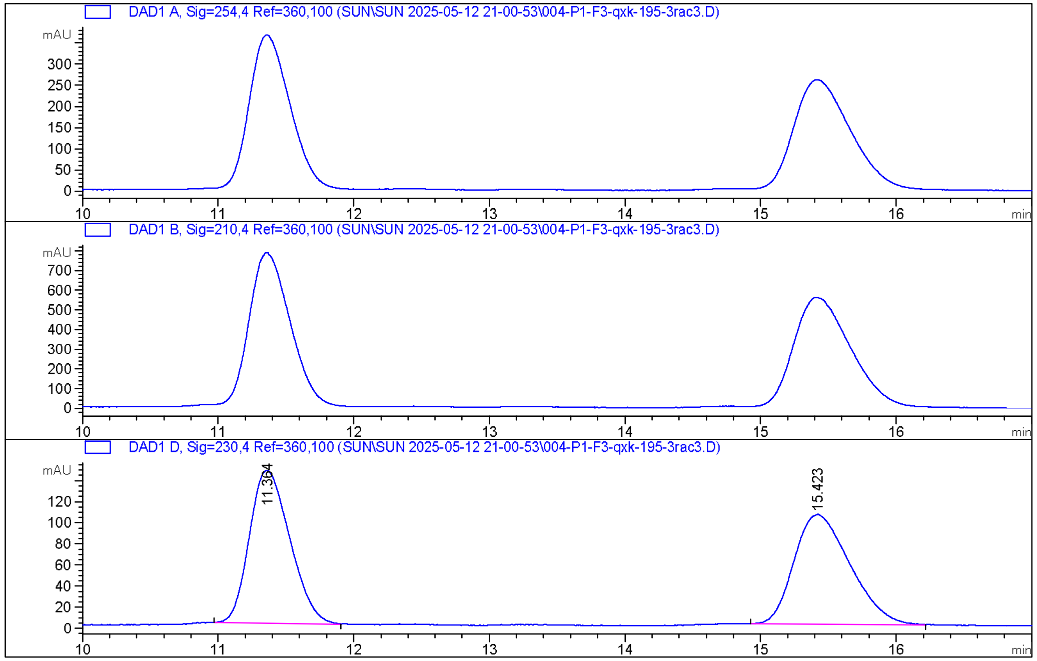

===
Area Percent Report
===

Sorted By : Signal
Multiplier : 1.0000
Dilution : 1.0000
Use Multiplier & Dilution Factor with ISTDs

Data File C:\Users\P...tation\1\Data\SUN\SUN 2025-05-12 21-00-53\004-P1-F3-qxk-195-3rac3.D
Sample Name: qxk-195-3rac3

Signal 1: DAD1 A, Sig=254,4 Ref=360,100

Signal 2: DAD1 B, Sig=210,4 Ref=360,100

Signal 3: DAD1 D, Sig=230,4 Ref=360,100

Peak #	RetTime [min]	Type	Width [min]	Area [mAU*s]	Height [mAU]	Area %
1	11.364	VV R	0.2483	3004.47559	144.95532	50.2019
2	15.423	VV R	0.3379	2980.30493	104.12953	49.7981

Totals : 5984.78052 249.08485

===
*** End of Report ***

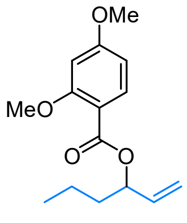

Rac

===

Acq. Operator : SYSTEM Seq. Line : 6
Sample Operator : SYSTEM
Acq. Instrument : HPLC Location : P1-E-03
Injection Date : 15/5/2025 4:14:24 pm Inj : 1
Inj Volume : 2.000 µl
Different Inj Volume from Sample Entry! Actual Inj Volume : 10.000 µl
Acq. Method : C:\Users\Public\Documents\ChemStation\1\Data\SUN\SUN 2025-05-15 14-17-27\IC3-10-30.M
Last changed : 19/8/2022 10:41:24 pm by SYSTEM
Analysis Method : C:\Users\Public\Documents\ChemStation\1\Data\SUN\SUN 2025-05-15 14-17-27\IC3-10-30.M (Sequence Method)
Last changed : 16/5/2025 9:11:20 pm by SYSTEM
(modified after loading)
Additional Info : Peak(s) manually integrated

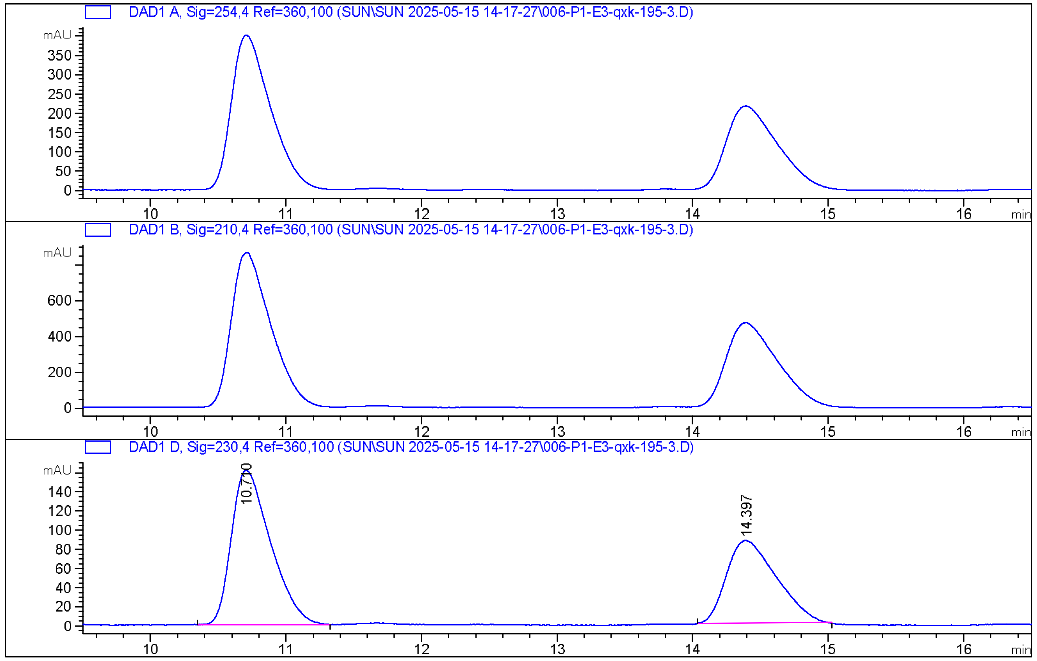

===
Area Percent Report
===

Sorted By : Signal
Multiplier : 1.0000
Dilution : 1.0000
Use Multiplier & Dilution Factor with ISTDs

Data File C:\Users\P...hemStation\1\Data\SUN\SUN 2025-05-15 14-17-27\006-P1-E3-qxk-195-3.D
Sample Name: qxk-195-3

Signal 1: DAD1 A, Sig=254,4 Ref=360,100

Signal 2: DAD1 B, Sig=210,4 Ref=360,100

Signal 3: DAD1 D, Sig=230,4 Ref=360,100

Peak #	RetTime [min]	Type	Width [min]	Area [mAU*s]	Height [mAU]	Area %
1	10.710	VV R	0.2504	3236.16797	161.38506	59.4265
2	14.397	MM R	0.4260	2209.49829	86.43795	40.5735
Totals :				5445.66626	247.82301	

===
*** End of Report ***

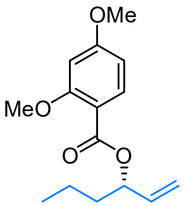

===

```
Acq. Operator    : SYSTEM                        Seq. Line :  21
Sample Operator  : SYSTEM
Acq. Instrument  : HPLC                           Location :   P1-F-02
Injection Date   : 16/1/2025 5:15:35 pm                Inj :   1
                                               Inj Volume : 2.000 µl
Different Inj Volume from Sample Entry!  Actual Inj Volume : 20.000 µl
Acq. Method      : C:\Users\Public\Documents\ChemStation\1\Data\SUN\SUN 2025-01-16 10-32-15\OD3-
                   05-30.M
Last changed     : 21/12/2024 9:30:27 pm by SYSTEM
Analysis Method  : C:\Users\Public\Documents\ChemStation\1\Data\SUN\SUN 2025-01-16 10-32-15\OD3-
                   05-30.M (Sequence Method)
Last changed     : 17/1/2025 8:45:37 pm by SYSTEM
                   (modified after loading)
Additional Info  : Peak(s) manually integrated
```

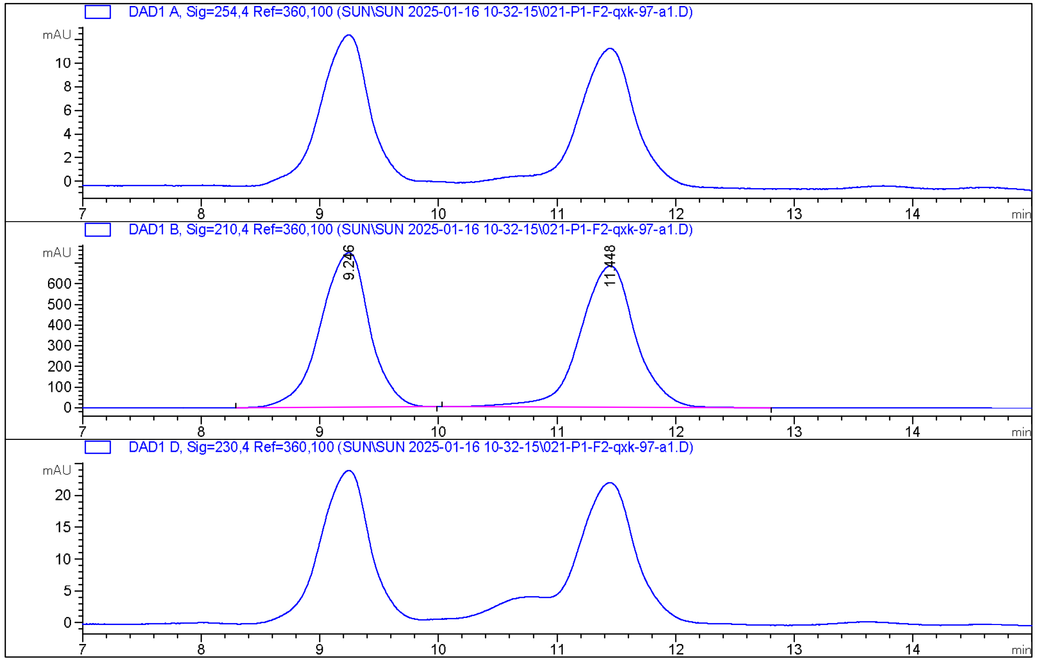

===

Area Percent Report

===

```
Sorted By             :      Signal
Multiplier            :      1.0000
Dilution              :      1.0000
Use Multiplier & Dilution Factor with ISTDs
```


Data File C:\Users\P...hemStation\1\Data\SUN\SUN 2025-01-16 10-32-15\021-P1-F2-qxk-97-a1.D
Sample Name: qxk-97-a1

Signal 1: DAD1 A, Sig=254,4 Ref=360,100

Signal 2: DAD1 B, Sig=210,4 Ref=360,100

Peak #	RetTime [min]	Type	Width [min]	Area [mAU*s]	Height [mAU]	Area %
1	9.246	BB	0.4393	2.14368e4	747.04004	49.2038
2	11.448	BB	0.4590	2.21306e4	683.95398	50.7962
Totals :				4.35674e4	1430.99402	

Signal 3: DAD1 D, Sig=230,4 Ref=360,100

===

*** End of Report ***

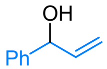

4-Rac

===

Acq. Operator : SYSTEM Seq. Line : 14
Acq. Instrument : LC1260 Location : P1-F-05
Injection Date : 3/6/2025 10:16:01 PM Inj : 1
Inj Volume : 5.000 µl
Different Inj Volume from Sample Entry! Actual Inj Volume : 20.000 µl
Acq. Method : C:\Users\Public\Documents\ChemStation\1\Data\SUN\SUN 2025-03-06 15-59-17\OD3-05-30.M
Last changed : 9/11/2024 5:02:48 PM by SYSTEM
Analysis Method : C:\Users\Public\Documents\ChemStation\1\Data\SUN\SUN 2025-03-06 15-59-17\OD3-05-30.M (Sequence Method)
Last changed : 3/13/2025 10:20:25 AM by SYSTEM
(modified after loading)
Additional Info : Peak(s) manually integrated

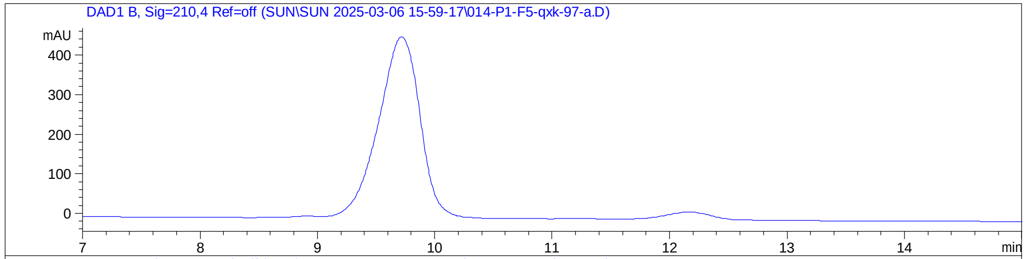

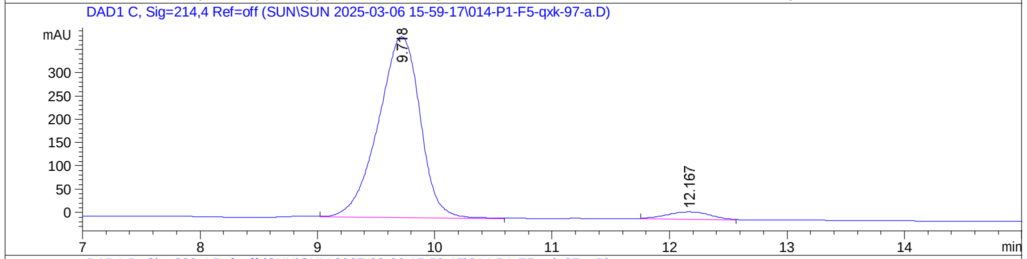

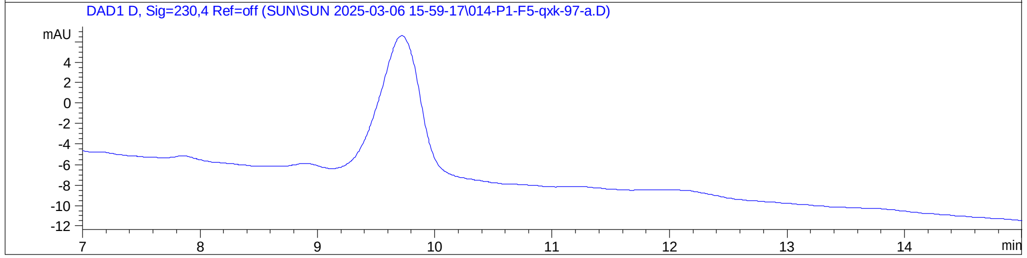

===

Area Percent Report

===

Sorted By : Signal
Multiplier : 1.0000
Dilution : 1.0000
Use Multiplier & Dilution Factor with ISTDs

Data File C:\Users\P...ChemStation\1\Data\SUN\SUN 2025-03-06 15-59-17\014-P1-F5-qxk-97-a.D
Sample Name: qxk-97-a

Signal 1: DAD1 B, Sig=210,4 Ref=off

Signal 2: DAD1 C, Sig=214,4 Ref=off

Peak #	RetTime [min]	Type	Width [min]	Area [mAU*s]	Height [mAU]	Area %
1	9.718	MM R	0.4127	9629.87402	388.89355	95.9216
2	12.167	MM R	0.4259	409.44067	16.02215	4.0784

Totals : 1.00393e4 404.91571

Signal 3: DAD1 D, Sig=230,4 Ref=off

===

*** End of Report ***

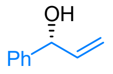

4

===

Acq. Operator : SYSTEM Seq. Line : 21
Acq. Instrument : LC1260 Location : P2-E-02
Injection Date : 2/28/2025 2:12:27 PM Inj : 1
Inj Volume : 5.000 µl
Different Inj Volume from Sample Entry! Actual Inj Volume : 20.000 µl
Acq. Method : C:\Users\Public\Documents\ChemStation\1\Data\SUN\SUN 2025-02-28 09-30-03\AD3-30-50.M
Last changed : 4/17/2024 2:31:09 PM by SYSTEM
Analysis Method : C:\Users\Public\Documents\ChemStation\1\Data\SUN\SUN 2025-02-28 09-30-03\AD3-30-50.M (Sequence Method)
Last changed : 2/28/2025 4:00:34 PM by SYSTEM
(modified after loading)
Additional Info : Peak(s) manually integrated

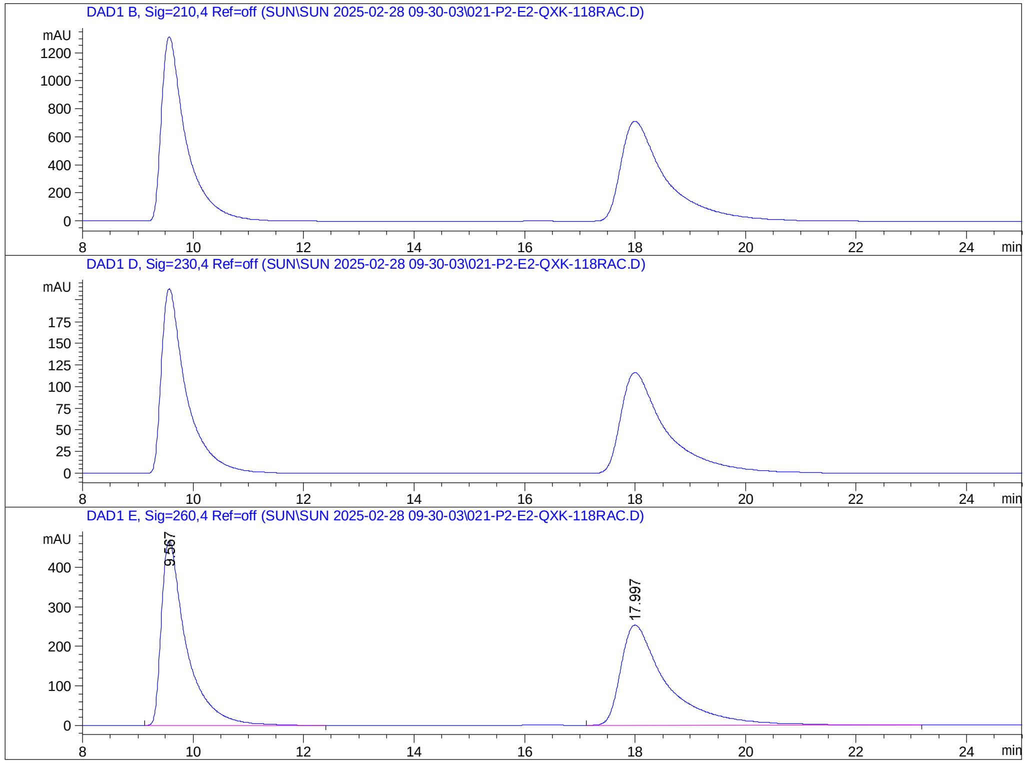

===
Area Percent Report
===

Sorted By : Signal
Multiplier : 1.0000
Dilution : 1.0000
Use Multiplier & Dilution Factor with ISTDs

Signal 1: DAD1 B, Sig=210,4 Ref=off

Signal 2: DAD1 D, Sig=230,4 Ref=off

Signal 3: DAD1 E, Sig=260,4 Ref=off

Peak #	RetTime [min]	Type	Width [min]	Area [mAU*s]	Height [mAU]	Area %
1	9.567	BB	0.4476	1.44735e4	466.98865	49.9899
2	17.997	BB	0.8237	1.44794e4	253.01590	50.0101

Totals : 2.89529e4 720.00455

===
*** End of Report ***

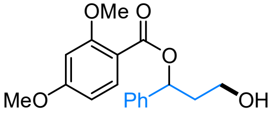

5-Rac

Data File C:\Users\P...\ChemStation\1\Data\SUN\SUN 2025-03-06 15-59-17\002-P1-F1-qxk-118.D
Sample Name: qxk-118

===

Acq. Operator : SYSTEM Seq. Line : 2
Acq. Instrument : LC1260 Location : P1-F-01
Injection Date : 3/6/2025 4:07:16 PM Inj : 1
Inj Volume : 5.000 µl
Different Inj Volume from Sample Entry! Actual Inj Volume : 20.000 µl
Acq. Method : C:\Users\Public\Documents\ChemStation\1\Data\SUN\SUN 2025-03-06 15-59-17\AD3-30-50.M
Last changed : 4/17/2024 2:31:09 PM by SYSTEM
Analysis Method : C:\Users\Public\Documents\ChemStation\1\Data\SUN\SUN 2025-03-06 15-59-17\AD3-30-50.M (Sequence Method)
Last changed : 3/13/2025 10:06:11 AM by SYSTEM
(modified after loading)
Additional Info : Peak(s) manually integrated

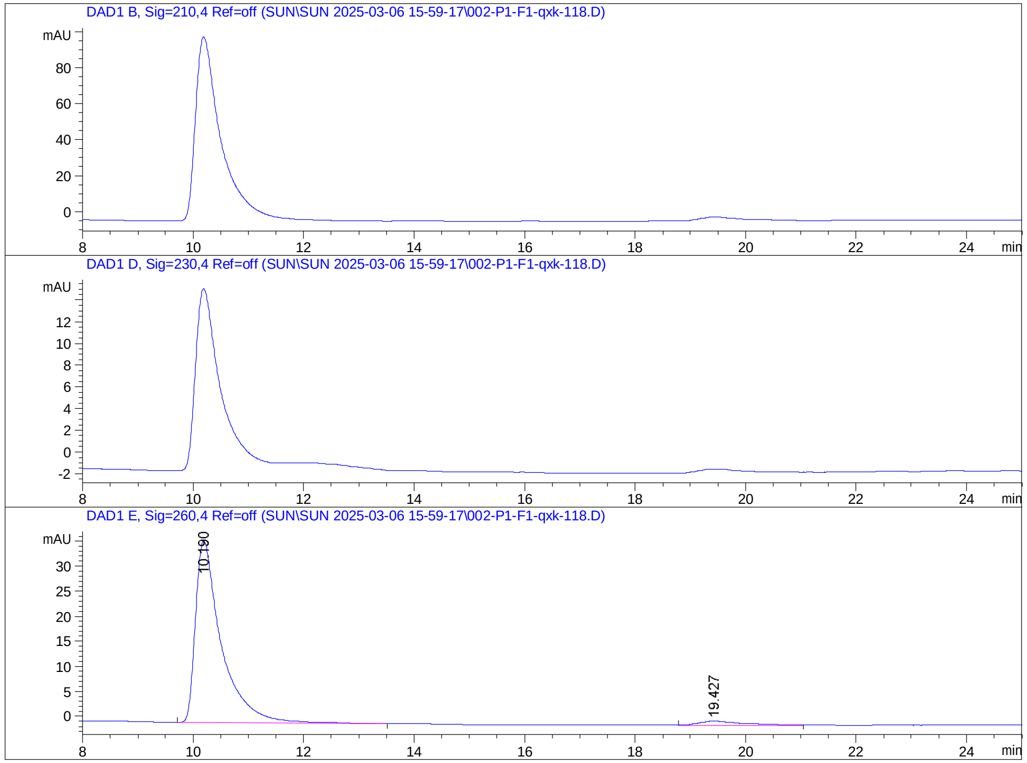

===

Area Percent Report

===

Sorted By : Signal
Multiplier : 1.0000
Dilution : 1.0000
Use Multiplier & Dilution Factor with ISTDs

Data File C:\Users\P...\ChemStation\1\Data\SUN\SUN 2025-03-06 15-59-17\002-P1-F1-qxk-118.D
Sample Name: qxk-118

Signal 1: DAD1 B, Sig=210,4 Ref=off

Signal 2: DAD1 D, Sig=230,4 Ref=off

Signal 3: DAD1 E, Sig=260,4 Ref=off

Peak #	RetTime [min]	Type	Width [min]	Area [mAU*s]	Height [mAU]	Area %
1	10.190	BB	0.4715	1188.15369	36.33092	96.2447
2	19.427	MM R	0.9771	46.35999	7.90748e-1	3.7553

Totals : 1234.51368 37.12167

===

*** End of Report ***

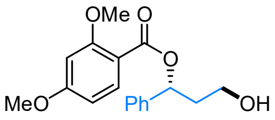

5

===

Acq. Operator : SYSTEM   Seq. Line : 12
Acq. Instrument : LC1260   Location : P1-F-01
Injection Date : 3/19/2025 12:54:15 PM   Inj : 1
Inj Volume : 5.000 µl
Different Inj Volume from Sample Entry! Actual Inj Volume : 20.000 µl
Acq. Method : C:\Users\Public\Documents\ChemStation\1\Data\SUN\SUN 2025-03-19 09-31-33 \IG3-30-50.M
Last changed : 4/19/2024 10:02:28 AM by SYSTEM
Analysis Method : C:\Users\Public\Documents\ChemStation\1\Data\SUN\SUN 2025-03-19 09-31-33 \IG3-30-50.M (Sequence Method)
Last changed : 3/20/2025 9:43:10 AM by SYSTEM
(modified after loading)
Additional Info : Peak(s) manually integrated

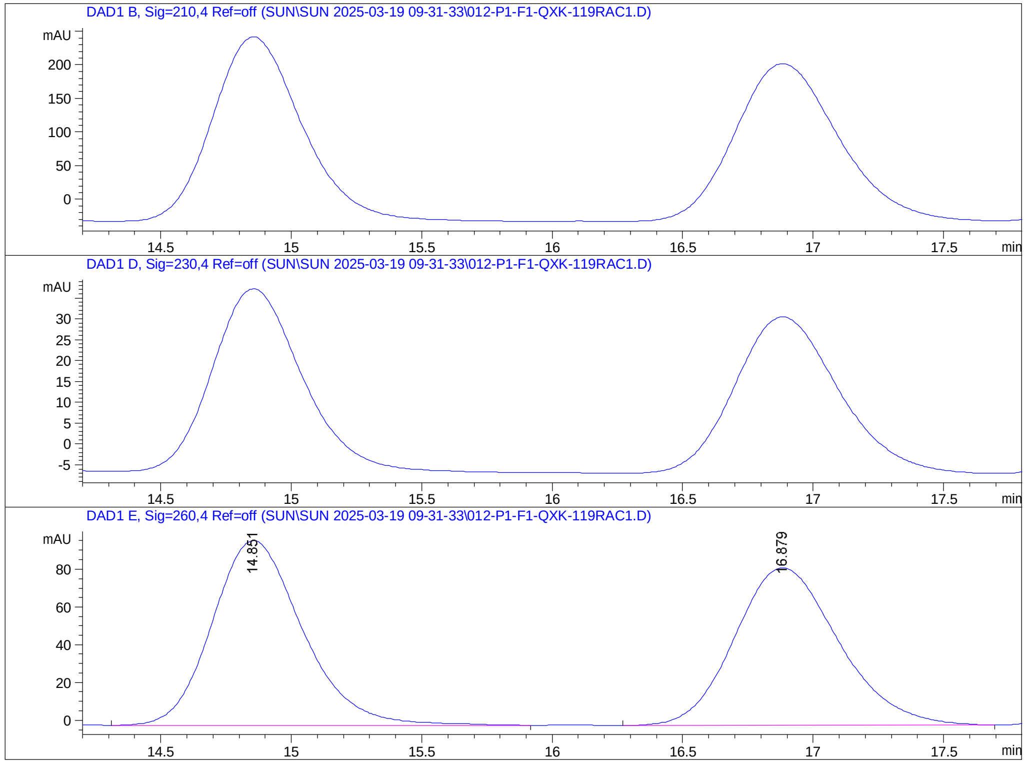

===

Area Percent Report

===

Sorted By : Signal
Multiplier : 1.0000
Dilution : 1.0000
Use Multiplier & Dilution Factor with ISTDs

Data File C:\Users\P...mStation\1\Data\SUN\SUN 2025-03-19 09-31-33\012-P1-F1-QXK-119RAC1.D
Sample Name: QXK-119RAC1

Signal 1: DAD1 B, Sig=210,4 Ref=off

Signal 2: DAD1 D, Sig=230,4 Ref=off

Signal 3: DAD1 E, Sig=260,4 Ref=off

Peak #	RetTime [min]	Type	Width [min]	Area [mAU*s]	Height [mAU]	Area %
1	14.851	BB	0.3701	2343.15649	97.58283	50.4653
2	16.879	BB	0.4285	2299.94458	83.21237	49.5347

Totals : 4643.10107 180.79520

===

*** End of Report ***

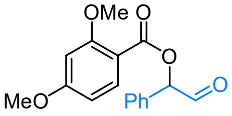

6-Rac

===

Acq. Operator : SYSTEM Seq. Line : 34
Acq. Instrument : LC1260 Location : P1-F-03
Injection Date : 3/19/2025 11:18:01 PM Inj : 1
Inj Volume : 5.000 µl
Different Inj Volume from Sample Entry! Actual Inj Volume : 20.000 µl
Acq. Method : C:\Users\Public\Documents\ChemStation\1\Data\SUN\SUN 2025-03-19 09-31-33\IG3-30-30.M
Last changed : 3/19/2025 8:15:45 PM by SYSTEM
Analysis Method : C:\Users\Public\Documents\ChemStation\1\Data\SUN\SUN 2025-03-19 09-31-33\IG3-30-30.M (Sequence Method)
Last changed : 3/20/2025 9:44:31 AM by SYSTEM
(modified after loading)
Additional Info : Peak(s) manually integrated

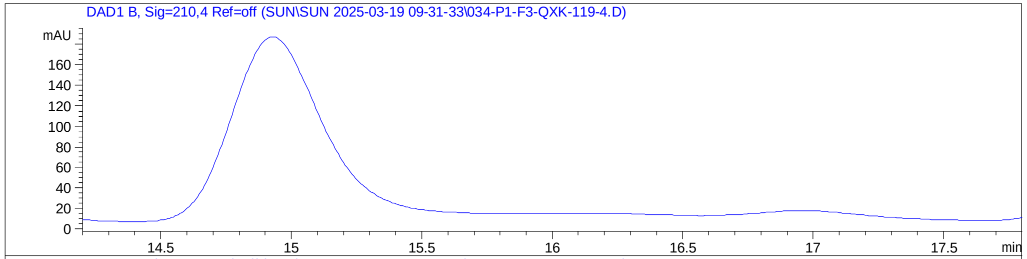

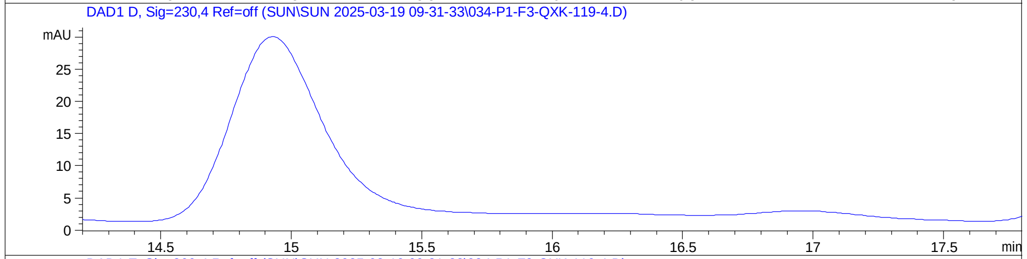

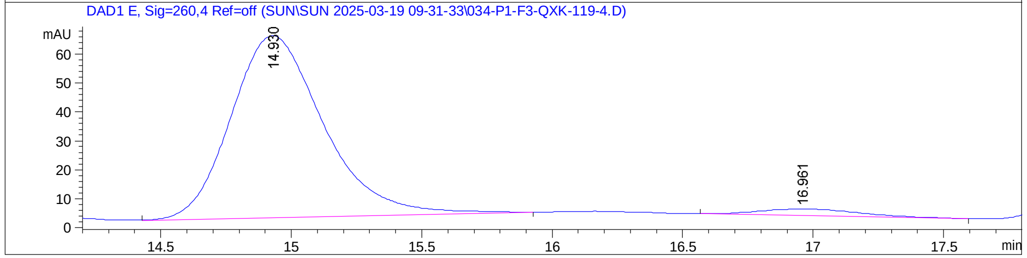

===

Area Percent Report

===

Sorted By : Signal
Multiplier : 1.0000
Dilution : 1.0000
Use Multiplier & Dilution Factor with ISTDs

Signal 1: DAD1 B, Sig=210,4 Ref=off

Signal 2: DAD1 D, Sig=230,4 Ref=off

Signal 3: DAD1 E, Sig=260,4 Ref=off

Peak #	RetTime [min]	Type	Width [min]	Area [mAU*s]	Height [mAU]	Area %
1	14.930	BB	0.3773	1547.68030	62.85179	96.1783
2	16.961	BB	0.4063	61.49739	2.32683	3.8217

Totals : 1609.17768 65.17862

===

*** End of Report ***

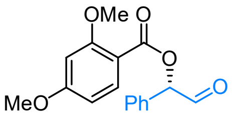

6

===

```
Acq. Operator   : SYSTEM                          Seq. Line :    2
Acq. Instrument : LC1260                           Location :   P2-F-01
Injection Date  : 3/13/2025 10:21:33 AM                 Inj :    1
                                                 Inj Volume : 5.000 µl
Different Inj Volume from Sample Entry!  Actual Inj Volume : 20.000 µl
Acq. Method     : C:\Users\Public\Documents\ChemStation\1\Data\SUN\SUN 2025-03-13 10-08-37
                  \AD3-20-30.M
Last changed    : 4/1/2024 7:51:19 PM by SYSTEM
Analysis Method : C:\Users\Public\Documents\ChemStation\1\Data\SUN\SUN 2025-03-13 10-08-37
                  \AD3-20-30.M (Sequence Method)
Last changed    : 3/13/2025 10:53:03 AM by SYSTEM
                  (modified after loading)
Additional Info : Peak(s) manually integrated
```

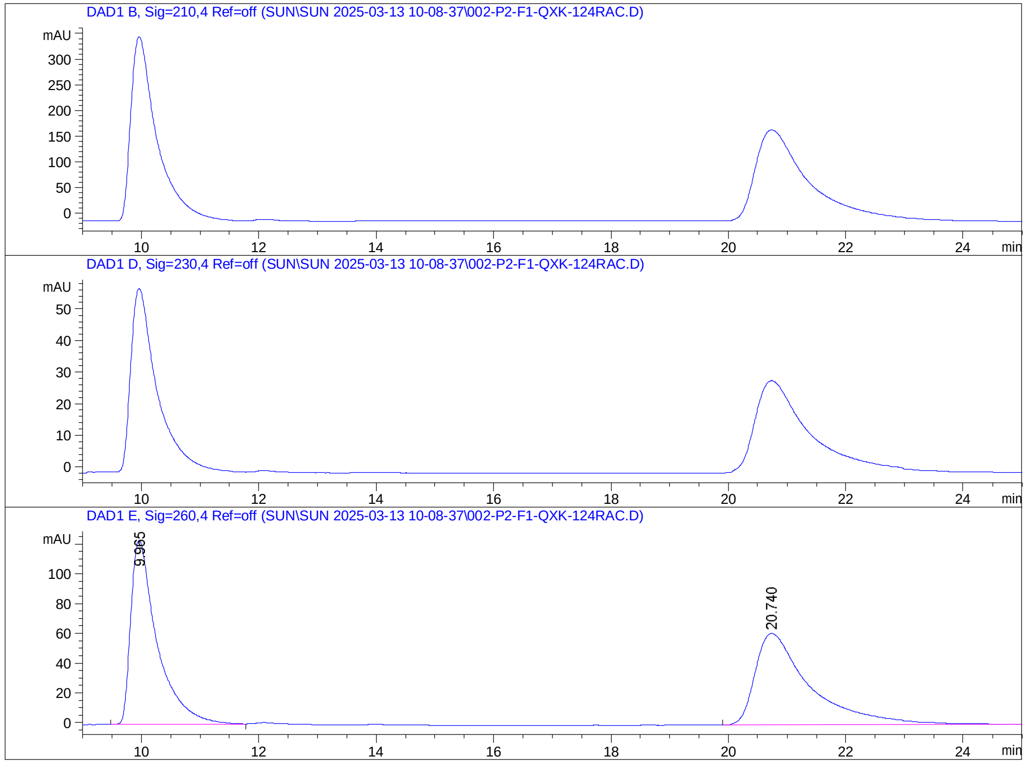

===

Area Percent Report

===

```
Sorted By             :      Signal
Multiplier            :      1.0000
Dilution              :      1.0000
Use Multiplier & Dilution Factor with ISTDs
```


Data File C:\Users\P...emStation\1\Data\SUN\SUN 2025-03-13 10-08-37\002-P2-F1-QXK-124RAC.D
Sample Name: QXK-124RAC

Signal 1: DAD1 B, Sig=210,4 Ref=off

Signal 2: DAD1 D, Sig=230,4 Ref=off

Signal 3: DAD1 E, Sig=260,4 Ref=off

Peak #	RetTime [min]	Type	Width [min]	Area [mAU*s]	Height [mAU]	Area %
1	9.965	BB	0.4558	3848.47607	123.40225	49.2082
2	20.740	BB	0.9212	3972.33105	61.36645	50.7918

Totals : 7820.80713 184.76870

===

*** End of Report ***

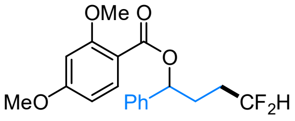

7-Rac

===

Acq. Operator : SYSTEM Seq. Line : 2
Acq. Instrument : LC1260 Location : P1-F-05
Injection Date : 3/7/2025 6:42:16 PM Inj : 1
Inj Volume : 5.000 µl
Different Inj Volume from Sample Entry! Actual Inj Volume : 30.000 µl
Acq. Method : C:\Users\Public\Documents\ChemStation\1\Data\SUN\SUN 2025-03-07 18-29-09\AD3-20-30.M
Last changed : 4/1/2024 7:51:19 PM by SYSTEM
Analysis Method : C:\Users\Public\Documents\ChemStation\1\Data\SUN\SUN 2025-03-07 18-29-09\AD3-20-30.M (Sequence Method)
Last changed : 3/7/2025 8:52:34 PM by SYSTEM
(modified after loading)
Additional Info : Peak(s) manually integrated

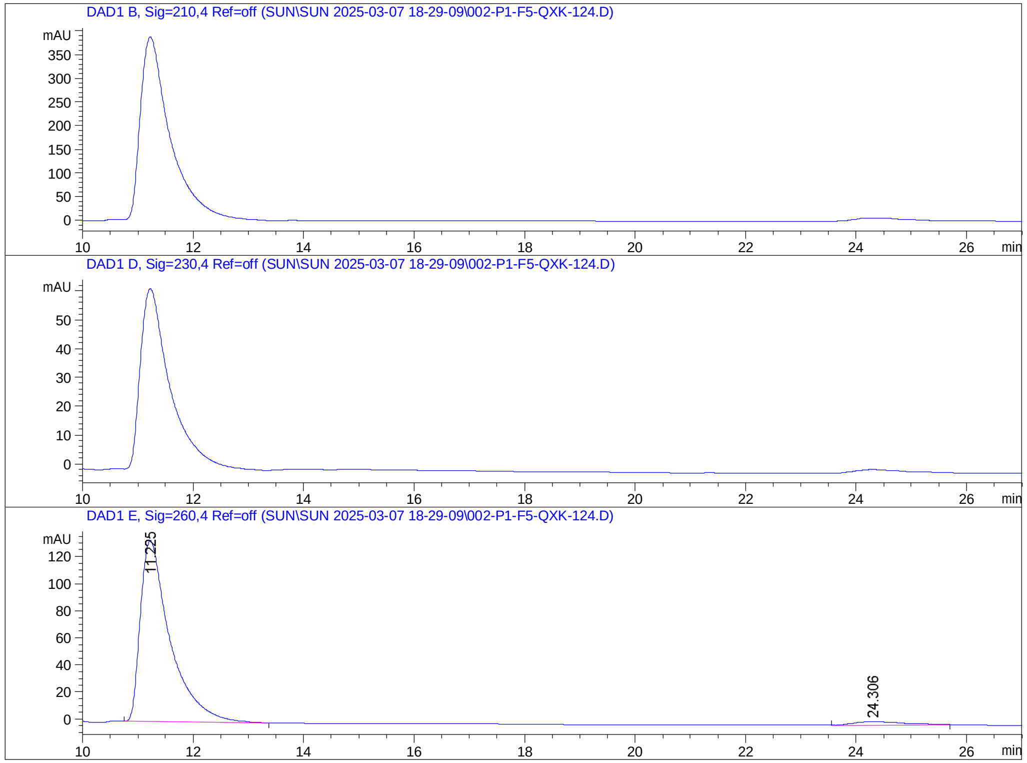

===

Area Percent Report

===

Sorted By : Signal
Multiplier : 1.0000
Dilution : 1.0000
Use Multiplier & Dilution Factor with ISTDs

Signal 1: DAD1 B, Sig=210,4 Ref=off

Signal 2: DAD1 D, Sig=230,4 Ref=off

Signal 3: DAD1 E, Sig=260,4 Ref=off

Peak #	RetTime [min]	Type	Width [min]	Area [mAU*s]	Height [mAU]	Area %
1	11.225	BB	0.5504	5004.52637	133.79146	96.6705
2	24.306	MM R	1.0714	172.36526	2.68123	3.3295

Totals : 5176.89163 136.47269

===

*** End of Report ***

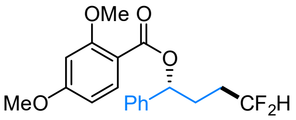

7

Data File C:\Users\P...Station\1\Data\SUN\SUN 2025-05-18 15-16-41\002-P1-F1-qxk-210-2rac.D
Sample Name: qxk-210-2rac

===

```
Acq. Operator   : SYSTEM                        Seq. Line :   2
Sample Operator : SYSTEM
Acq. Instrument : HPLC                           Location :   P1-F-01
Injection Date  : 18/5/2025 3:30:03 pm                Inj :   1
                                               Inj Volume : 2.000 µl
Different Inj Volume from Sample Entry!  Actual Inj Volume : 10.000 µl
Acq. Method     : C:\Users\Public\Documents\ChemStation\1\Data\SUN\SUN 2025-05-18 15-16-41\ID3-
                  20-30.M
Last changed    : 21/9/2022 10:08:04 am by SYSTEM
Analysis Method : C:\Users\Public\Documents\ChemStation\1\Data\SUN\SUN 2025-05-18 15-16-41\ID3-
                  20-30.M (Sequence Method)
Last changed    : 23/5/2025 10:29:15 am by SYSTEM
                  (modified after loading)
Additional Info : Peak(s) manually integrated
```

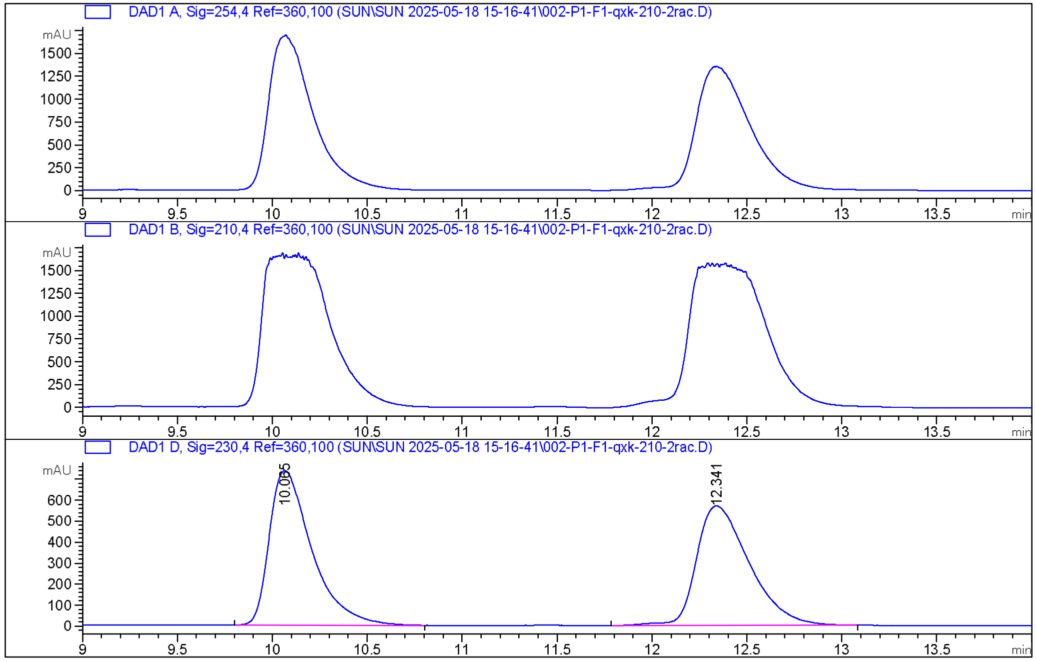

===

Area Percent Report

===

```
Sorted By          :      Signal
Multiplier         :      1.0000
Dilution           :      1.0000
Use Multiplier & Dilution Factor with ISTDs
```


Data File C:\Users\P...Station\1\Data\SUN\SUN 2025-05-18 15-16-41\002-P1-F1-qxk-210-2rac.D
Sample Name: qxk-210-2rac

Signal 1: DAD1 A, Sig=254,4 Ref=360,100

Signal 2: DAD1 B, Sig=210,4 Ref=360,100

Signal 3: DAD1 D, Sig=230,4 Ref=360,100

Peak #	RetTime [min]	Type	Width [min]	Area [mAU*s]	Height [mAU]	Area %
1	10.065	VV R	0.2147	1.14635e4	738.26990	50.9798
2	12.341	VV R	0.2285	1.10228e4	570.39642	49.0202
Totals :				2.24863e4	1308.66632	

===

*** End of Report ***

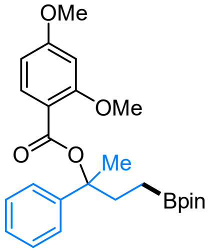

8-Rac

===

Acq. Operator : SYSTEM Seq. Line : 20
Sample Operator : SYSTEM
Acq. Instrument : HPLC Location : P1-F-01
Injection Date : 19/5/2025 5:09:32 pm Inj : 1
Inj Volume : 2.000 µl
Different Inj Volume from Sample Entry! Actual Inj Volume : 20.000 µl
Acq. Method : C:\Users\Public\Documents\ChemStation\1\Data\SUN\SUN 2025-05-19 13-08-21\ID3-20-30.M
Last changed : 21/9/2022 10:08:04 am by SYSTEM
Analysis Method : C:\Users\Public\Documents\ChemStation\1\Data\SUN\SUN 2025-05-19 13-08-21\ID3-20-30.M (Sequence Method)
Last changed : 23/5/2025 10:32:15 am by SYSTEM
(modified after loading)
Additional Info : Peak(s) manually integrated

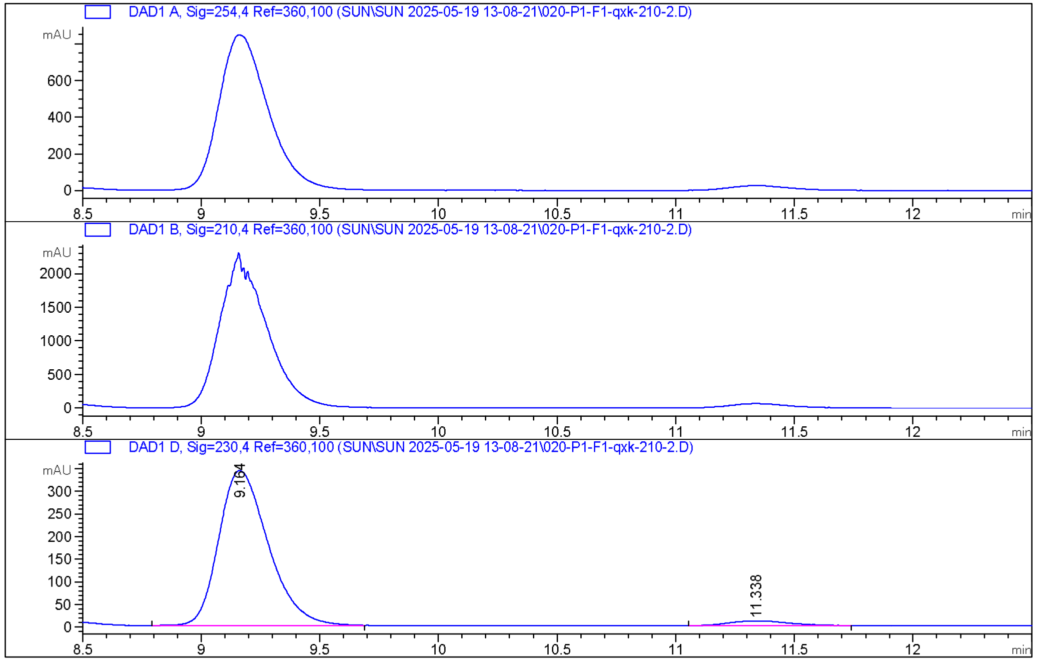

===

Area Percent Report

===

Sorted By : Signal
Multiplier : 1.0000
Dilution : 1.0000
Use Multiplier & Dilution Factor with ISTDs

Data File C:\Users\P...hemStation\1\Data\SUN\SUN 2025-05-19 13-08-21\020-P1-F1-qxk-210-2.D
Sample Name: qxk-210-2

Signal 1: DAD1 A, Sig=254,4 Ref=360,100

Signal 2: DAD1 B, Sig=210,4 Ref=360,100

Signal 3: DAD1 D, Sig=230,4 Ref=360,100

Peak #	RetTime [min]	Type	Width [min]	Area [mAU*s]	Height [mAU]	Area %
1	9.164	VV R	0.1954	4971.24219	343.06094	96.3655
2	11.338	MM R	0.2866	187.49648	10.90328	3.6345

Totals : 5158.73866 353.96422

===

*** End of Report ***

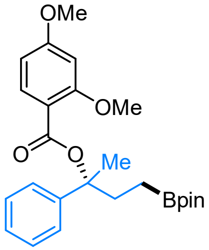

8

===

Acq. Operator : SYSTEM Seq. Line : 81
Sample Operator : SYSTEM
Acq. Instrument : HPLC Location : P1-E-03
Injection Date : 23/5/2025 11:33:44 am Inj : 1
Inj Volume : 2.000 µl
Different Inj Volume from Sample Entry! Actual Inj Volume : 20.000 µl
Acq. Method : C:\Users\Public\Documents\ChemStation\1\Data\SUN\SUN 2025-05-22 11-00-10\OJ3-10-20.M
Last changed : 7/10/2022 10:20:38 am by SYSTEM
Analysis Method : C:\Users\Public\Documents\ChemStation\1\Data\SUN\SUN 2025-05-22 11-00-10\OJ3-10-20.M (Sequence Method)
Last changed : 23/5/2025 3:29:40 pm by SYSTEM
(modified after loading)
Additional Info : Peak(s) manually integrated

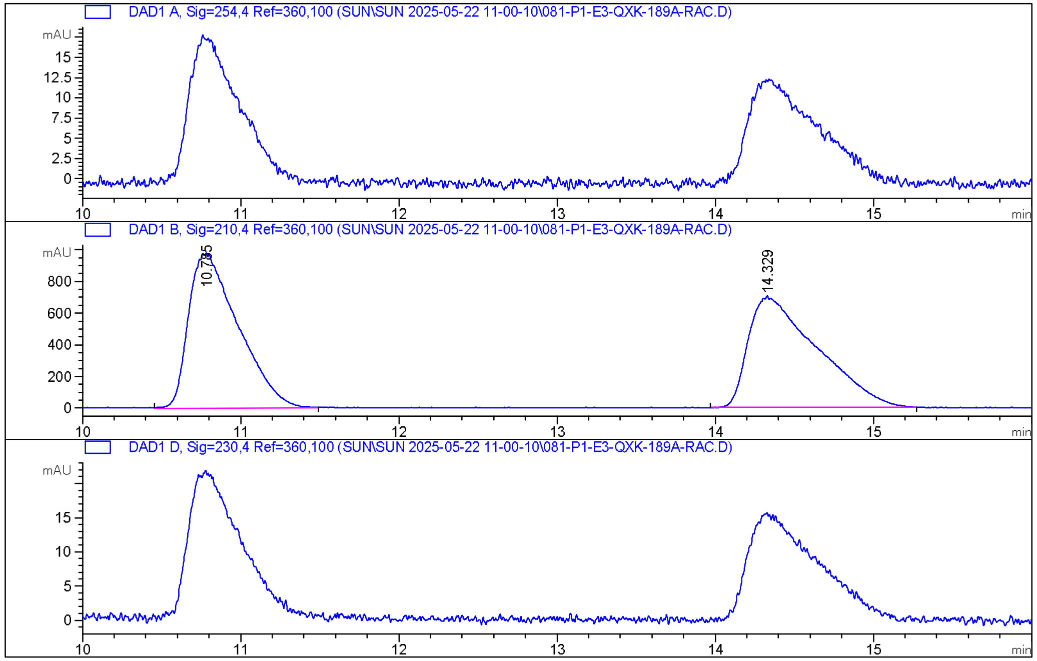

===
Area Percent Report
===

Sorted By : Signal
Multiplier : 1.0000
Dilution : 1.0000
Use Multiplier & Dilution Factor with ISTDs

Data File C:\Users\P...Station\1\Data\SUN\SUN 2025-05-22 11-00-10\081-P1-E3-QXK-189A-RAC.D
Sample Name: QXK-189A-RAC

Signal 1: DAD1 A, Sig=254,4 Ref=360,100

Signal 2: DAD1 B, Sig=210,4 Ref=360,100

Peak #	RetTime [min]	Type	Width [min]	Area [mAU*s]	Height [mAU]	Area %
1	10.785	MM R	0.3606	2.12746e4	983.32813	50.2840
2	14.329	MM R	0.4984	2.10343e4	703.42896	49.7160
Totals :				4.23089e4	1686.75708	

Signal 3: DAD1 D, Sig=230,4 Ref=360,100

===
*** End of Report ***

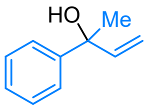

9-Rac

Data File C:\Users\P...hemStation\1\Data\SUN\SUN 2025-05-23 14-05-52\003-P1-E1-QXK-189A2.D
Sample Name: QXK-189A2

===

```
Acq. Operator   : SYSTEM                     Seq. Line :   3
Sample Operator : SYSTEM
Acq. Instrument : HPLC                        Location :   P1-E-01
Injection Date  : 23/5/2025 2:40:17 pm             Inj :   1
                                            Inj Volume : 2.000 µl
Different Inj Volume from Sample Entry!  Actual Inj Volume : 5.000 µl
Acq. Method     : C:\Users\Public\Documents\ChemStation\1\Data\SUN\SUN 2025-05-23 14-05-52\OJ3-
                  10-20.M
Last changed    : 7/10/2022 10:20:38 am by SYSTEM
Analysis Method : C:\Users\Public\Documents\ChemStation\1\Data\SUN\SUN 2025-05-23 14-05-52\OJ3-
                  10-20.M (Sequence Method)
Last changed    : 23/5/2025 3:31:49 pm by SYSTEM
                  (modified after loading)
Additional Info : Peak(s) manually integrated
```

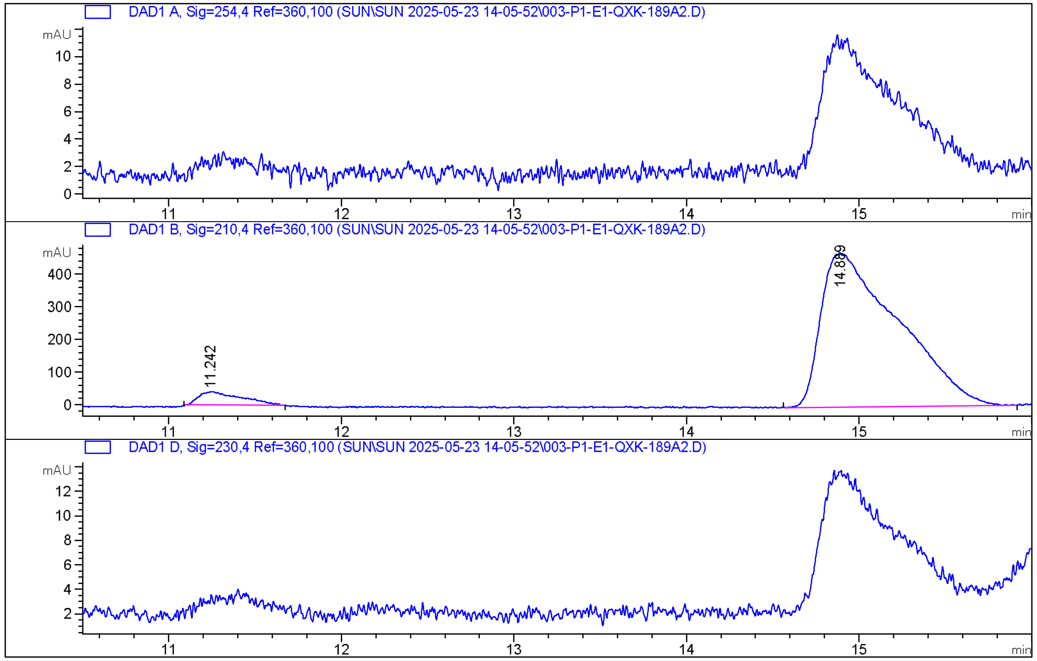

===
Area Percent Report
===

```
Sorted By             :      Signal
Multiplier            :      1.0000
Dilution              :      1.0000
Use Multiplier & Dilution Factor with ISTDs
```


Data File C:\Users\P...hemStation\1\Data\SUN\SUN 2025-05-23 14-05-52\003-P1-E1-QXK-189A2.D
Sample Name: QXK-189A2

Signal 1: DAD1 A, Sig=254,4 Ref=360,100

Signal 2: DAD1 B, Sig=210,4 Ref=360,100

Peak #	RetTime [min]	Type	Width [min]	Area [mAU*s]	Height [mAU]	Area %
1	11.242	MM R	0.2848	693.76740	40.59356	4.4879
2	14.889	MM R	0.5176	1.47648e4	475.38147	95.5121
Totals :				1.54585e4	515.97503	

Signal 3: DAD1 D, Sig=230,4 Ref=360,100

===
*** End of Report ***

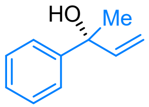

9

===

```
Acq. Operator   : SYSTEM                    Seq. Line :   5
Sample Operator : SYSTEM
Acq. Instrument : HPLC                       Location :   P1-F-03
Injection Date  : 18/5/2025 4:43:34 pm            Inj :   1
                                           Inj Volume : 2.000 µl
Different Inj Volume from Sample Entry!  Actual Inj Volume : 20.000 µl
Acq. Method     : C:\Users\Public\Documents\ChemStation\1\Data\SUN\SUN 2025-05-18 15-16-41\OD3-
                  05-30.M
Last changed    : 21/12/2024 9:30:27 pm by SYSTEM
Analysis Method : C:\Users\Public\Documents\ChemStation\1\Data\SUN\SUN 2025-05-18 15-16-41\OD3-
                  05-30.M (Sequence Method)
Last changed    : 23/5/2025 10:35:39 am by SYSTEM
                  (modified after loading)
Additional Info : Peak(s) manually integrated
```

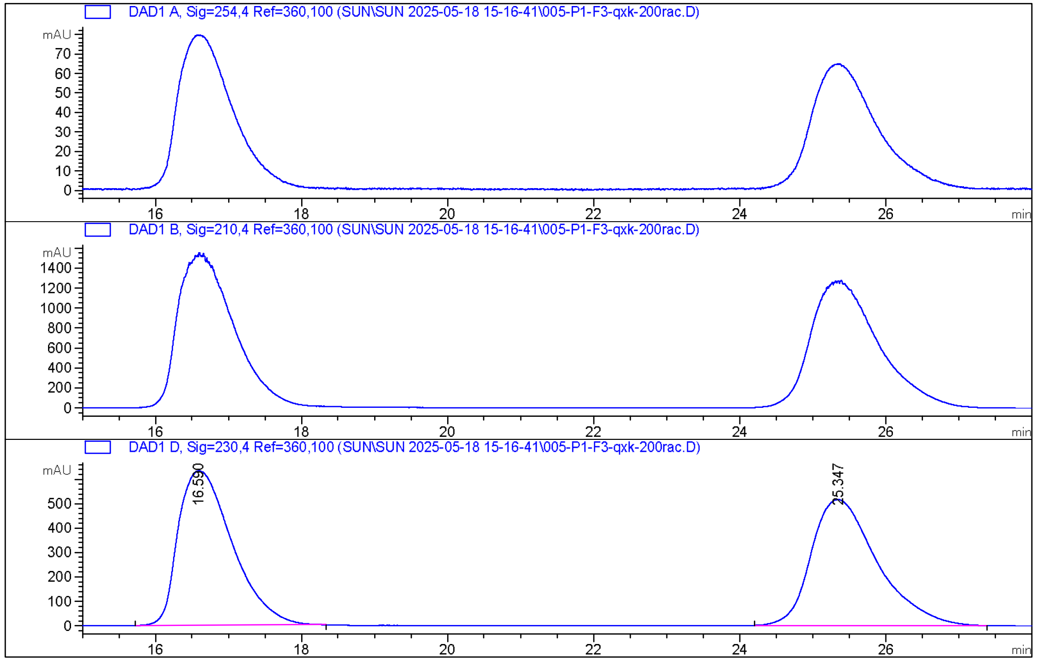

===

Area Percent Report

===

```
Sorted By             :      Signal
Multiplier            :      1.0000
Dilution              :      1.0000
Use Multiplier & Dilution Factor with ISTDs
```


Data File C:\Users\P...emStation\1\Data\SUN\SUN 2025-05-18 15-16-41\005-P1-F3-qxk-200rac.D
Sample Name: qxk-200rac

Signal 1: DAD1 A, Sig=254,4 Ref=360,100

Signal 2: DAD1 B, Sig=210,4 Ref=360,100

Signal 3: DAD1 D, Sig=230,4 Ref=360,100

Peak #	RetTime [min]	Type	Width [min]	Area [mAU*s]	Height [mAU]	Area %
1	16.590	MM R	0.8548	3.26239e4	636.10760	50.0703
2	25.347	MM R	1.0484	3.25323e4	517.17853	49.9297

Totals : 6.51562e4 1153.28613

===

*** End of Report ***

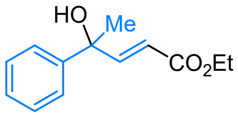

10-Rac

Data File C:\Users\P...hemStation\1\Data\SUN\SUN 2025-05-22 11-00-10\032-P1-F3-QXK-200-3.D
Sample Name: QXK-200-3

===

Acq. Operator : SYSTEM Seq. Line : 32
Sample Operator : SYSTEM
Acq. Instrument : HPLC Location : P1-F-03
Injection Date : 22/5/2025 8:55:18 pm Inj : 1
Inj Volume : 2.000 µl
Different Inj Volume from Sample Entry! Actual Inj Volume : 10.000 µl
Acq. Method : C:\Users\Public\Documents\ChemStation\1\Data\SUN\SUN 2025-05-22 11-00-10\OD3-05-30.M
Last changed : 21/12/2024 9:30:27 pm by SYSTEM
Analysis Method : C:\Users\Public\Documents\ChemStation\1\Data\SUN\SUN 2025-05-22 11-00-10\OD3-05-30.M (Sequence Method)
Last changed : 23/5/2025 10:37:41 am by SYSTEM
(modified after loading)
Additional Info : Peak(s) manually integrated

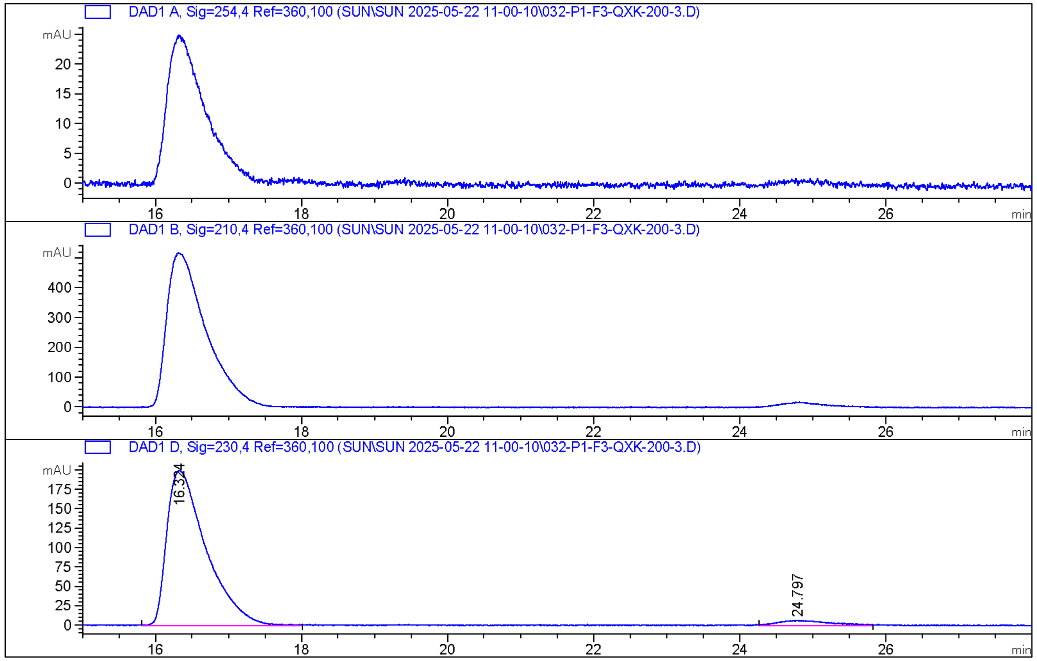

===

Area Percent Report

===

Sorted By : Signal
Multiplier : 1.0000
Dilution : 1.0000
Use Multiplier & Dilution Factor with ISTDs

Data File C:\Users\P...hemStation\1\Data\SUN\SUN 2025-05-22 11-00-10\032-P1-F3-QXK-200-3.D
Sample Name: QXK-200-3

Signal 1: DAD1 A, Sig=254,4 Ref=360,100

Signal 2: DAD1 B, Sig=210,4 Ref=360,100

Signal 3: DAD1 D, Sig=230,4 Ref=360,100

Peak #	RetTime [min]	Type	Width [min]	Area [mAU*s]	Height [mAU]	Area %
1	16.324	MM R	0.6229	7487.86963	200.34441	95.9661
2	24.797	MM R	0.7740	314.74734	6.77725	4.0339

Totals : 7802.61697 207.12165

===

*** End of Report ***

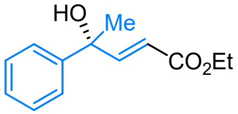

10
